# Training student volunteers as community resource navigators to address patients' social needs: A curriculum toolkit

**DOI:** 10.3389/fpubh.2022.966872

**Published:** 2022-09-20

**Authors:** Diwas Gautam, Sahil Sandhu, Kate Kutzer, Lillian Blanchard, Jacqueline Xu, Veronica Sotelo Munoz, Erika Dennis, Connor Drake, Carolyn Crowder, Howard Eisenson, Janet Prvu Bettger

**Affiliations:** ^1^Trinity College of Arts & Sciences, Duke University, Durham, NC, United States; ^2^Sanford School of Public Policy, Duke University, Durham, NC, United States; ^3^Duke University School of Nursing, Durham, NC, United States; ^4^Department of Population Health Sciences, Duke University School of Medicine, Durham, NC, United States; ^5^Lincoln Community Health Center, Durham, NC, United States; ^6^Department of Orthopaedic Surgery, Duke University School of Medicine, Durham, NC, United States

**Keywords:** social determinants of health (MeSH), social needs, primary care (MeSH), academic-community partnership, curriculum-undergraduate and postgraduate

## Abstract

**Introduction:**

Few resources are available to train students to provide patients assistance for obtaining needed community-based services. This toolkit outlines a curriculum to train student volunteers to become “community resource navigators” to serve patients *via* telephone at partner health sites.

**Methods:**

University students co-designed the Help Desk navigator program and training for volunteer navigators as part of an academic-community partnership with a local Federally Qualified Health Center (FQHC). The multi-modal curricula consisted of five components: didactic instruction on social determinants of health and program logistics, mock patient calls and documentation, observation of experienced navigator interaction with patients, supervised calls with real patients, and homework assignments. In 2020, training materials were adapted for virtual delivery due to the COVID-19 pandemic. Trainees completed a survey after completion to provide qualitative feedback on the training and preparedness.

**Results:**

The training was offered for the first cohort of 11 student volunteer navigators in 2019, revised and then offered for 13 undergraduate and nursing students over 6 weeks in 2020. In the training evaluation, trainees described the new knowledge and skills gained from the training, the long-term benefits toward their educational and professional career goals, and helpful interactive delivery of the training. Trainees also highlighted areas for improvement, including more time learning about community resources and practicing challenging patient conversations.

**Conclusions:**

Our peer-to-peer, multi-modal training prepares student volunteers to become community resource navigators. Student, eager for meaningful clinical experiences, are an untapped resource that can help patients with their social needs.

## Introduction

According to the World Health Organization, the social determinants of health (SDOH) are “the conditions in which people are born, grow, work, live and age, and the wider set of forces and systems shaping the conditions of daily life” (1). The United States Healthy People 2030 initiative has prioritized improving SDOH in their overarching objectives to improve health and wellbeing nationwide. Specifically, they organize SDOH into five broad categories: economic stability, education, social and community context, health and health care, and neighborhood and built environment (2). Downstream consequences of SDOH include patient-level social needs, such as food insecurity, transportation barriers, social isolation, and housing instability (3). These social needs are major drivers of health and health disparities (4). In response, multiple professional organizations across medical specialties, government agencies, and the National Academy of Medicine have recommended healthcare providers identify and address patients' social needs (5–8). Unfortunately, community-based healthcare organizations may lack the capacity to fully address these needs. Through academic-community partnerships, the health sector can leverage student volunteers as an untapped resource to improve integrated health and social care. In return, students build valuable inter- and intraprofessional competencies in a meaningful experiential learning opportunity with exposure to the relationship between SDOH and health outcomes.

Despite growing evidence on the feasibility and effectiveness of volunteer models that engage students to address social needs in the clinical setting (9–12), most studies do not provide full details of the content and delivery of the training program needed for replication (13). The purpose of this paper is to disseminate a structured curriculum for an expert-informed, peer-to- peer training model that can be adopted and adapted by other programs to train undergraduate and pre-licensure health professions students to become volunteer “community resource navigators.” Our multimodal structured trainings, originally developed through an academic-community partnership, can be used to equip student volunteers with the knowledge and skills necessary to help patients in accessing resources to meet their unmet social needs.

## Methods and pedagogical framework

### Program background

In 2018, four university students and a professor partnered with the chief medical officer and director of behavioral health at a Federally Qualified Health Center (FQHC) in Durham, North Carolina to co-develop and implement a “Help Desk” volunteer program. In this model, case managers on the FQHC's behavioral health team screen clinic patients for social needs using the Protocol for Responding to and Assessing Patients' Assets, Risks, and Experiences (PRAPARE) screening tool and refer them to resources internal to the FQHC or to community-based resources that address unmet social needs such as food, transportation, and housing (14). Student volunteer “community resource navigators” follow-up with patients telephonically in English or Spanish, both 2 weeks and 4 weeks after their initial visit. Their goal is to help patients overcome potential barriers and encourage the initiation of services or uptake of community resources.

This ongoing academic-community partnership, now in its fourth year, was originally supported through a university-wide initiative to support interdisciplinary research teams of students, faculty, and community partners tackling complex societal challenges. Between spring 2018 to present (fall 2021), key phases of the partnership included (i) a 3-month engagement period among partners to discuss the target patient population and opportunities for students to complement existing social care efforts, (ii) a six-month planning period to develop navigator workflows, trainings, resource directory, and data infrastructure, (iii) a two-month pilot, and (iv) over 2 years of program implementation, maintenance, and scale-up as part of routine clinical care. Full program details are described elsewhere (14–17). The program continues with annual appointments of new student leadership who recruit, train, and manage a cohort of peers who volunteer as navigators for healthcare partners as part of a university student organization.

### Curriculum development, learning objectives, and learning areas

Our curriculum aims to equip volunteers with the knowledge and skills needed to telephonically motivate patients to connect to community resources, as recommended by their case manager, and if necessary, to work with patients to identify alternative resources. Helping patients manage their social needs requires students to understand community resources, communicate effectively and compassionately, and tailor support to respond to individual circumstances.

Curriculum development was guided by the literature (18–20) and by partners and advisors with expertise in community-based health care delivery, general and pediatric medicine, behavioral health, health services and implementation research, academic curriculum development, and clinician education. Our student team actively engaged FQHC leadership and case managers to identify the essential knowledge and skills to support their patients as navigators. Additionally, we consulted similar social needs volunteer programs to learn about their process for experiential training components (10, 21).

**Figure 1:**
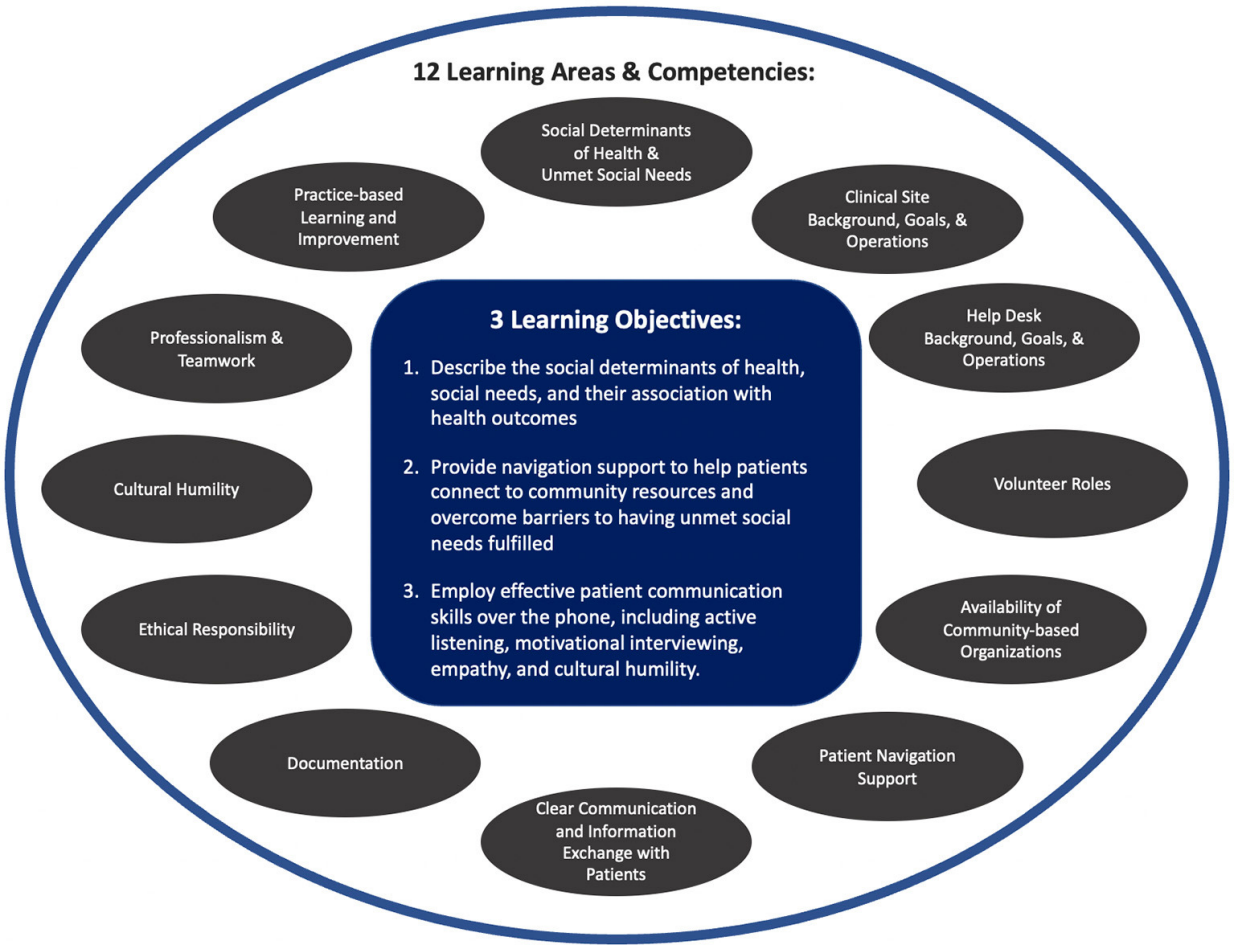
Help desk learning objectives and learning areas.

The student leadership team created a set of three overarching learning objectives, 12 learning areas (Figure 1), and 54 specific learning outcomes (Appendix 1). Since targeted trainees included pre-medical students, we also aligned our training's areas and specific materials with the core pre-professional competencies outlined by Association of American Medical Colleges (AAMC) (22). Our curriculum and program develops 12 of the 15 AAMC competencies, including service orientation, social skills, cultural competence, teamwork, oral communication, ethical responsibility to self and others, reliability and dependability, resilience and adaptability, capacity for improvement, critical thinking, written communication, and human behavior. Our curriculum also aligns more broadly with essential learning objectives for liberal arts education defined by American Association of Colleges and Universities (e.g., personal and social responsibility, integrative and applied learning) and leverages multiple evidence-based, high-impact practices shown to be associated with higher levels of learning success (e.g., service-learning, community-based learning) (23, 24).

## Learning environment and pedagogical format

### Volunteer recruitment

Target learners include medical, nursing, undergraduate and graduate students in any area of study. To advertise the training program and opportunity, we reached out to pre-health and health professions student organizations, cultural student groups, and academic programs. We assessed applicants' demonstrated interest in SDOH, previous clinical and volunteer experiences, and ability to relate to the diverse patient population served by our health care partner. We also conducted interviews and mock patient calls to assess applicants' oral communication skills and ability to demonstrate empathy over the phone. Those interested in serving Spanish-speaking patients were cleared for Spanish language fluency by a native Spanish speaker on the student leadership team. A sample of our recruitment materials is available in Appendix 2.

#### Training implementation

The training was first delivered in 2019 by the four program's developers and experienced student navigators (SS, LB, JX, VSM) for a cohort of 11 student volunteers. Over the 2019–2020 implementation year, the curriculum was refined and then delivered by three Help Desk program leads and experienced student navigators (DG, KK, SS) for our second cohort of thirteen students to volunteer in 2020–2021. Demographic characteristics of our trainees are presented in Table 1.

**Table 1:** Student volunteer characteristics.

**Characteristics**	**Number of volunteers** ** (*N* = 24)**
**Race/ethnicity**	
Asian	8
Hispanic	8
White	7
Black	1
**Gender**	
Female	18
Male	6
**Student level**	
**Undergraduate**	17
2nd year undergraduate	*(4)*
3rd year undergraduate	*(8)*
4th year undergraduate	*(5)*
Graduate or professional	7
**Degrees/programs**	
Natural sciences (e.g., Biology, Neuroscience)	9
Social sciences (e.g., Public Policy, Psychology)	8
Health professions (Medicine, Nursing)	5
Mathematics or computer science	2
**Career trajectories of graduated students**	
Health professions students (e.g., medicine, dentistry, clinical psychology)	15
Resident physician	1
Licensed nurse	3
Health policy research	1
Other	1
Average semesters volunteered	2.5

**Figure 2:**
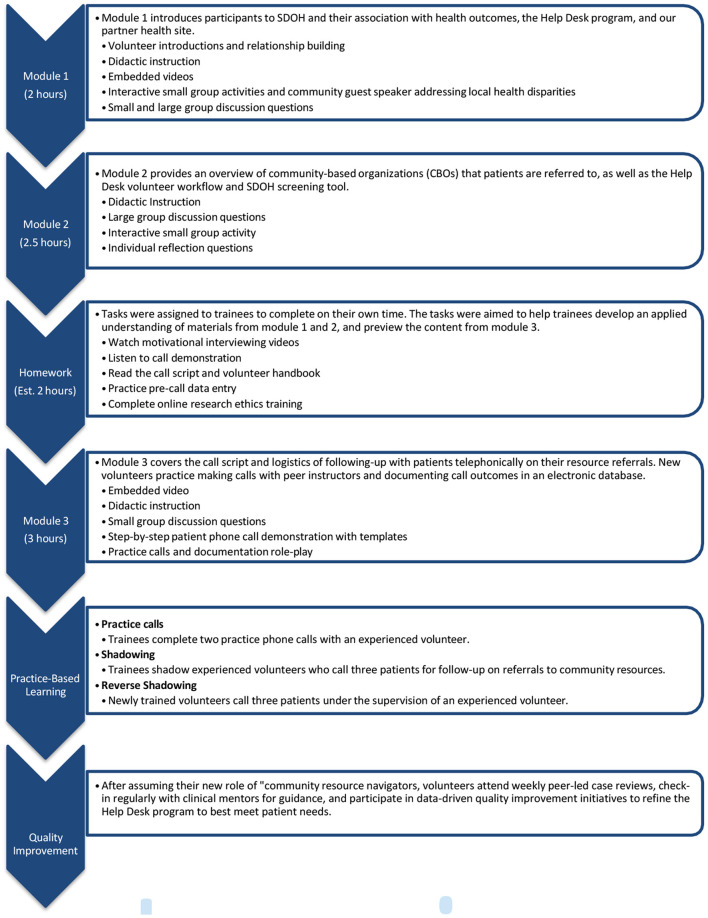
Help desk training overview.

Volunteer recruitment, selection, onboarding, and training took 12 weeks. The training program consisted of five components: didactic instruction on SDOH and program logistics, homework assignments, mock patient calls and documentation, observation of experienced navigators with patients, and supervised calls with actual patients. Appendix 3 outlines the full training overview and detailed timeline. The didactic component consisted of three facilitated, 2–3 h- long modules conducted over three different sessions (Figure 2). Help Desk student leads facilitated all three didactic sessions. The facilitator's guide is in Appendix 4.

*Module 1* aims to address the first learning objective: *describe the social determinants of health and how they affect health outcomes*. The module included PowerPoint presentations (Appendix 5) on SDOH, an overview of interventions to address social needs in clinic settings and the history and goals of our local Help Desk program. Learners also participated in an interactive activity to explore data on disparities in health and social needs in the local community (activity handout in Appendix 6).

*Module 2* aims to address the learning objective: *provide navigation support to help patients connect to community resources and overcome barriers*. The module included a PowerPoint presentation (Appendix 7) on the Help Desk program workflow, a pre-recorded video demonstrating documentation of patient data into our electronic REDCap database, and an interactive activity to learn about community-based organizations using our program's community resource directory (activity handout in Appendix 8). Using the twenty-four most common resource referrals from our data (18), learners worked in assigned groups to explore a resource (e.g., local food pantry) and present to the whole cohort on: (i) how each resource specifically addresses a patient's need (ii) the target population, and (iii) the best way to access each resource.

*Module 3* aims to address the learning objective: *demonstrate effective patient communication skills over the phone, including active listening, motivational interviewing, empathy, and cultural humility*. This module included a PowerPoint presentation (Appendix 9) that addressed the goals of follow-up calls, and further discussed motivational interviewing strategies, the call script, call logistics, and data entry. The session concluded with a peer-to-peer mock call activity (activity handout in Appendix 10). Between modules 2 and 3, learners completed homework activities that aligned with both module's learning objectives. Activities included reviewing the volunteer handbook and script, watching a video demonstration of a typical Help Desk call and a YouTube video about motivational interviewing (25), and completing a reflection exercise with questions aimed to improve retention and understanding.

All three didactic modules were conducted over 1 week in three separate sessions. Session one and two lasted 2 h each, and session three lasted 3 h. For the first cohort in 2019, didactic training components were conducted in-person in a conference room at the university using handouts and slideshow presentations were displayed with projectors. For our second cohort of students in 2020, all didactic components of the training were conducted virtually *via* Zoom due to the COVID-19 pandemic. Handouts could be viewed on the volunteers' personal computers and the student leadership team used the share screen function in Zoom to deliver didactic instruction. Group interactive activities were facilitated using Zoom breakout rooms. There were no prerequisites for the didactic modules.

Following all three sessions, learners completed two practice calls with a student facilitator who offered feedback on community resource fit, motivational interviewing technique, and conversational flow. Similarly to didactic training, these practice sessions were conducted in a conference room for the first cohort and *via* Zoom in breakout rooms for the second cohort. Appendix 11 includes materials for the practice call, such as patient scenarios, fake completed social needs screening assessments, and telephone scripts. Learners then completed on-boarding requirements for our partner health site (e.g., background check, reviewing HIPAA and protected health information fact sheets). The first cohort completed the health site specific onboarding in-person at the FQHC in a single day. However, the second cohort completed this onboarding remotely by reviewing these materials due to the pandemic. Subsequent cohorts have returned to completing the partner health site specific orientations in person, however; all other parts of training have remained virtual for convenience and costs.

Once approved, learners shadowed current resource navigators in completing three patient calls before calling three patients under the supervision of an experienced volunteer. The first cohort completed shadowing and reverse shadowing calls on the university campus in the designated Help Desk call room. Having students call patients from a secure office on the main university campus rather than traveling to the FQHC prevented transportation challenges for students and enabled our team to overcome the lack of physical space at the FQHC to accommodate a student office on-site. The second cohort completed patient calls *via* Zoom with the calls on speaker phone so both the volunteer in training and the student facilitator could hear the call. Facilitator guides were created for the practice calls (Appendix 12), shadowing (Appendix 13), and reverse shadowing (Appendix 14). During practice calls and reverse shadowing experiences, experienced navigators completed a fidelity checklist to track progress (Appendix 15). Once learners completed the full training and became real-world “Help Desk” volunteers, they further refined their skills through continuing education activities, including weekly peer-led case reviews, and tested revisions to their workflows and role as part of continuous quality improvement processes.

#### Program and curriculum evaluation

We evaluated our curriculum and program across five domains, and these are reflected in our program's logic model (15). In brief, these include student experience of the training program, student knowledge and skills gained from the training, student growth from participating in the program, student-led dissemination efforts, and student volunteer effectiveness for patient reach and service connection.

#### Student experience with the training program

We conducted a qualitative evaluation of our curriculum for its second implementation with the 2020–2021 cohort. We asked learners to complete a survey with five open-ended reflection questions (Appendix 16). The evaluation methodology and structure were adapted from an interprofessional training program for medical students to address health disparities (26). The survey allowed the students to reflect on the strengths and weaknesses of the training and what they learned from it. We administered the survey 3 weeks after the conclusion of training when learners have had experience making several calls on their own.

Three team members (DG, KK, and SS) reviewed survey responses and conducted a thematic content analysis (27). First, we created preliminary codes *via* an inductive approach. Second, we grouped our preliminary codes into broader themes and subthemes, with representative quotations. Given our training targeted a small group of learners in the first two first years, our qualitative methodology enabled us to gather more robust and concrete feedback to drive improvement efforts for future years (28). We received open-ended evaluation survey responses from 10 of the 2020–2021 learners (nine undergraduates and one nursing student).

#### Student knowledge and skills gained from the training

The same qualitative survey and thematic content analysis described above was also used to assess student acceptability of the training program.

#### Student growth from program participation

Given the long-term student outcome for our program was to prepare students as contributing members of the public health system, we measured the number of students who pursued healthcare and health-related professions after graduating.

#### Student-led dissemination efforts

Help Desk volunteers collected patient-reported outcomes and experience data through follow-up calls in an effort to improve patient experience with primary care at the partner FQHC and to contribute to the evidence base for social interventions. Measures for dissemination of our program include the number of student-led peer-reviewed publications and posters/presentations at state, national, and international conferences.

#### Student volunteer effectiveness for patient reach and service connection

Measures for volunteer effectiveness include (1) number of patients referred to follow-up who were successfully reached *via* phone by student volunteers; (2) number of patients who attempted to contact a referred resource; and (3) number of patients who successfully connected with a referred resource.

## Results

### Student experience with the training program

Learners described the strengths and weaknesses of how the training components were delivered. They highlighted what was helpful, what could be improve upon, and gave helpful insights about the virtual nature of the training program. Representative quotes are presented in Table 3.

#### Utility of interactive and practice-based modules

Learners enjoyed the team-based, active learning components of the training. Activities such as researching local resources and practice calls with experienced volunteers helped to increase volunteer comfort working with patients and replicate real life conditions. Learners also shared the benefits of learning from previous Help Desk student volunteers.

#### Opportunities for improvement

Learners consistently described two areas for improvement: more time learning about community resources and practicing challenging patient conversations. Learners felt that our module on community resources felt rushed and hoped to learn more about specific eligibility requirements of resources and services for specific populations (e.g., Spanish-speaking patients). Learners also desired more “off-the-script” practice calls that had more curve balls, and specific training on strategies to handle difficult situations.

#### Virtual training

Learners felt that the virtual delivery of the training was smooth and did not negatively affect their ability to develop necessary skills and knowledge. Learners shared that utilizing breakout rooms in Zoom facilitated engagement among peers. Additionally, learners found recordings and availability of asynchronous materials useful in their learning.

### Student knowledge and skills gained from the training program

Learners described the knowledge and skills they gained to prepare them to become community resource navigators. They also identified how they could translate what they learned to their future educational and professional careers. Representative quotes are presented in Table 2.

**Table 2:** Representative quotes of learner feedback on student experience and delivery of training components.

**Subtheme**	**Quote**
Utility of interactive and practice-based modules	• “The fact that we researched most of the resources individually during training was also very helpful.” • “Practice calls, shadowing, and reverse shadowing allowed me to feel comfortable when I made calls on my own as I felt like I had already interacted with patients before in a controlled environment when it was better to make mistakes.” • “Each time I completed a practice call I felt considerably more confident and comfortable both with the script and with my ability to communicate with patients.”
Opportunities for improvement	• “I wish we had spent a bit more time on learning the exact parameters of the most common referrals. By that I mean how reachable those referrals are, which ones can be relied upon, and which ones have a tendency to be less helpful.” • “The only topic I wished we covered more is practicing scenarios with our peers when there was an abnormal situation such as the patient picking up the call in the hospital.” • “Having more practice with strictly motivational interviewing situations and having direct feedback would be very helpful...I feel like it's such a valuable tool, but it's a skill, and skills take time to develop.”
Virtual training	• “I don't think that the virtual training negatively impacted my experience primarily because since this volunteering is remote all of the practice was also remote meaning that it was very similar to what I am doing with directly volunteering. While direct interaction with people is always better than zoom I believe that the training could still be conducted over zoom in the future after COVID and the effectiveness will still be the same.” • “Being virtual was pretty good for the setup we had. It made it really easy to share the screen and see what was going on. The breakout rooms were good because they let us practice looking things up in smaller groups and helped us stay engaged.” • “I also benefited greatly from watching demonstrations of how to use certain resources, such as the End Hunger Durham food resource map. I felt that one place for improvement is that the specific discussion questions asked could have been a bit more tailored to align with the most common questions patients ask about these resources.”

**Table 3:** Representative quotes on knowledge gained and skills developed by learners.

**Subtheme**	**Quote**
New knowledge about social determinants of health and the local community	• “I learned a lot about the Durham community… my biggest takeaway is my new knowledge of the community organizations (especially food pantries) that are available to Durham residents.” • “The training program did a great job of emphasizing and educating volunteers about which social determinants of health the Durham community faces.”
Improved interpersonal and communication skills	• “I will definitely use the phone skills and motivational interviewing techniques to help patients discover their own reasons for making healthy changes...As a nurse, it is important to make sure my patients will be able to follow through with their wellness goals and treatment, so this has been eye opening to different barriers to care that I did not previously consider.” • “I think one of the biggest takeaways was improving my ability to adapt a set script to a patient's individual needs so that their experience is more personalized and they feel heard… [another] takeaway was practicing empathy without letting my feelings overwhelm me.” • “It's very frustrating not being able to help people experiencing exceedingly stressful circumstances. However, for that reason, I have found it very valuable to put into practice the emotional support skills we learned.”
Applying knowledge and skills to future educational and professional career	• “I enjoyed case review because it adds a team aspect to the program. I love how we can discuss the patients, and potential resource referrals to help our patients. This is an aspect that I think connects to working with a team of medical professionals in the future.” • “I hope to become a physician in the future and communication with patients while empathizing and informing them about tough subjects is a crucial part of the job. Thus, the skills garnered about having tough conversations as well as the tough conversations I have had with patients have allowed me to be better prepared in that regard for my future career.”

#### New knowledge about social determinants of health and the local community

Learners were motivated by their improved knowledge of social determinants of health, the distribution of social risk factors in the local community, and resources available to support patients' social needs.

#### Improved interpersonal and communication skills

Learners valued their training in patient- provider communication, active listening, and empathy. In particular, they appreciated learning about motivational interviewing. Given some learners had little prior experience working directly with patients, they described the importance of practicing how to multitask during a patient call (e.g., listening to patients, navigating the script and resource information, and documenting the call), adapting the script when needed, and managing difficult conversations.

#### Applying knowledge and skills to future educational and professional career

Learners described how the training prepared them not just as community resource navigators, but as future physicians, nurses, and other health professionals.

### Student growth from program participation

The Help Desk program has supported students in pursuing healthcare and health-related professions (19 of 20 volunteers who have already graduated from Duke University). Fifteen pre-health students of the Help Desk program have successfully matriculated into a health professions graduate program (e.g., medical, dental, clinical psychology), four graduate students who were already in health professions schools have successfully become registered nurses or resident physicians, and one student has successfully pursued a career in health policy research (Table 1).

### Student-led dissemination efforts

Our work has resulted in four student-led, peer-reviewed publications and eight presentations at state, national, international conferences focused on the development and implementation of the Help Desk model, adaptations of the program during the pandemic, evaluating factors associated with a successful patient referral, and patient-reported barriers to accessing referred resources (15–17, 29).

### Student volunteer effectiveness for patient reach and service connection

Between March 2019 and December 2020, student volunteers called 791 patients and successfully reached 501 (78%) of patients referred to follow-up by their case manager (17). Within 4 weeks of the initial referral, 63.3% of patients had at attempted to contact at least referred resource and 32.7% had started services with 1 or more of their referred resources (17).

## Discussion

Our curriculum trains student volunteers to become “community resource navigators” to serve patients with health-related social needs, such as food insecurity and housing instability, at partner health care sites *via* telephone. The curriculum uses a peer-to peer model and multimodal approach that includes didactic instruction, interactive activities, homework activities, peer shadowing, and supervised phone calls with clinic patients. Trainees reported gaining the specific knowledge and skills needed to help patients connect to community resources. Our curriculum's hands-on approach which provided trainees repeated opportunities to practice their role was crucial for students to develop the necessary confidence and competence.

Our curriculum is unique in the scope and types of learners targeted. While other published SDOH education materials have focused on medical students or residents (25–30), our curriculum focused on training an interprofessional group of students, that included undergraduate and graduate students. To our knowledge, we are the first group to develop a service-learning program aimed to assist patients with social needs, expand capacity of health services, and include learning outcomes intentionally designed to align with the AAMC's “Core Competencies for Incoming Medical Students.” Our curriculum emphasized fundamental patient-provider communication skills, such as motivational interviewing and active listening, in addition to SDOH content and service orientation. We encourage the medical education community to consider how they can extend their research and education initiatives to engage the pipeline of college students interested in the health professions.

The strengths of our program include its multi-modal, virtual delivery that allowed for both synchronous and asynchronous learning. Previous curricula have focused on educating students broadly on SDOH and their impacts on health, screening for social needs, and physician advocacy (26, 27). In contrast, our curriculum specifically trains students to help patients navigate community resources to address social needs. Students shadowed and practiced patient phone calls with experienced student navigators, and conducted supervised calls with clinic patients.

Centered on an engagement opportunity outside of the classroom and traditional academic curricula, our program focuses on preparing students to immediately work with patients in a volunteer capacity through a service-learning program developed through an academic- community partnership.

Our qualitative analysis of post-training survey revealed positive feedback from students and will inform future curricular design. Consistent with findings from evaluations of other SDOH curriculum, students described they gained knowledge of social determinants of health and health disparities of the local community, and skills to address social needs (30, 31). Unique to our study, likely due to our focus on preprofessional learners, students reported their development of broader interpersonal and communication skills needed to become future physicians and health professionals. Survey data also highlighted opportunities for curricular improvements: in future iterations, we will spend more time discussing local community resources and will develop more difficult patient scenarios for practice calls.

### Supporting successful sustainability and scalability

Since the original two cohorts, we have trained an additional 64 students to become Help Desk navigators and currently have 43 active volunteers as of Summer 2022. In addition to continue to support our original FQHC partner, we have adapted our training materials and workflows to serve other clinical sites in Durham, North Carolina including our own institution's Emergency Department and pediatrics clinic. In fall 2022, we are also planning to expand our program to serve additional service lines in our institution's health system in partnership with our health system's population health management office. Recent adaptations to our training and service model include deploying students to move beyond follow-up calls to also screen patients for social needs and to make resource referrals using a new, state-wide electronic referral platform for health and social services.

While this paper focuses of disseminating curricular components our structured training program, there are remaining research opportunities to evaluate the implementation and effectiveness of our training approach. First, administering pre-post surveys to students, with survey items mapped to our curricular learning objectives and learning areas, can provide quantitative evidence of training effectiveness and better identify curricular gaps. Second, we can assess the volunteer characteristics associated with better patient outcomes and satisfaction (e.g., undergraduate vs. health professions graduate students; year in school; degree program; previous volunteer experience; length of engagement; racial, ethnic, and language concordance between navigator and patient). Third, we can compare the effectiveness of a trained student workforce in supporting patients' social needs to other healthcare staff. For example, we are currently conducting a study using a factorial design to evaluate the effectiveness of providing social care in primary care across social workers, community health workers, and student volunteer navigators.

As other institutions consider replicating or adapting our curriculum and broader program components, there are a variety of lessons learned that may be helpful to consider. First, implementation requires an existing infrastructure for students to volunteer with a partner healthcare organization to provide direct service to patients. Approaches may include academic-community research projects, service-learning models integrated into undergraduate coursework, expansion of applied interprofessional education programs to include undergraduate students, or student-led service organizations funded by the university. Across models, strong student leadership and faculty oversight are important to create and support the student workforce. For example, more recently our program has transitioned into an official student organization with a robust organizational structure. Leadership roles include program coordinators, specific site coordinators, recruitment and training leads, communication officer, treasurer, liaisons with community-based organizations, director of programming for continuing education, and a secretary.

While our peer-to-peer training model relies on current navigators to recruit and train the next volunteer cohort, others institutions launching their own program should consider having academic or clinical partners leading the trainings until a student-led program is fully in operation. In particular, having sufficient trainers to supervise the applied, experiential components of the training (e.g., practice calls with other students using a fidelity checklist) is crucial. For successful implementation beyond the initial training, we recommend that navigators be continuously trained on communication and community resources throughout their tenure. Local services and eligibility change, and these nuances can dramatically affect patient access and the ability to have needs met.

This toolkit provides educators, community partners, and clinical leadership the resources to train students to function as volunteers in program to address social needs. Our curriculum demonstrates the feasibility and opportunity to include university students interested in health professions in interprofessional education activities, while increasing clinic capacity to support patients' unmet social needs through a trained volunteer workforce.

## Data availability statement

The original contributions presented in the study are included in the article/Supplementary material, further inquiries can be directed to the corresponding author/s.

## Author contributions

DG, SS, and KK drafted the manuscript. All authors revised the manuscript critically for important intellectual content, approved of the version of the manuscript to be published, and were involved in the conception and implementation of the project.

## Funding

Funding was provided by the Duke University Bass Connections Program (Durham, NC, USA).

## Conflict of interest

The authors declare that the research was conducted in the absence of any commercial or financial relationships that could be construed as a potential conflict of interest.

## Publisher's note

All claims expressed in this article are solely those of the authors and do not necessarily represent those of their affiliated organizations, or those of the publisher, the editors and the reviewers. Any product that may be evaluated in this article, or claim that may be made by its manufacturer, is not guaranteed or endorsed by the publisher.
