# Supplementary material for: Training student volunteers as community resource navigators to address patients' social needs: A curriculum toolkit
Source: Front Public Health. 2022 Sep 20;10:966872. doi: 10.3389/fpubh.2022.966872 (PMC9531674; doi:10.3389/fpubh.2022.966872)
Supplement: Supplementary file 1 [file Data_Sheet_1.zip › Data Sheet 10.docx]

**Facilitator guide for mock call**

**Mock call 1**

1. Start with the trainee who was assigned patient 2 for preview:

Sample patient 1 information:

Background: A 36 year old woman living with her husband and three children. Her children’s school is closed and they are in need of food.

Referrals: SNAP, Durham FEAST

Situation: SNAP: used, not satisfied. Hasn’t connected to Durham FEAST

1. Help the trainee set up their desktop
   1. REDCap, Script, CBO directory
      1. Remind them that usually, we will use google voice, however, this time we will be just practicing over zoom
2. Help the trainee review their referrals
3. Facilitator should act as the patient and respond to trainee as they mock the call
   1. Try to give general responses. However, acting patients are encouraged to deviate from the script to simulate real life conditions.
4. After the call, while they are doing post-call data entry, go through the fidelity checklist (see appendix O)
5. Once a trainee finishes post-call data entry, check their data entry and give them overall feedback.
6. Ask the second trainee who was listening during call with patient 1 about their thoughts and observations.
   1. Ask how the call went for them
   2. Tell them what they did well and how they can improve

**Mock call 2**

1. Repeat steps 1-7 for trainee who previewed patient 2.

Sample patient 2 information:

Background: Joseph McPhee is a hard of hearing 77 year old man living with his wife. He is in need of medicine but doesn’t have insurance.

Referrals: Senior PharmAssist; LCHC Transportation

Situation:

1. He is a little confused and can’t hear very well
2. SPA: He is confused, hasn’t heard of it.
3. Transportation: Used it, gave it at 10/10 for both.
