# Supplementary material for: Training student volunteers as community resource navigators to address patients' social needs: A curriculum toolkit
Source: Front Public Health. 2022 Sep 20;10:966872. doi: 10.3389/fpubh.2022.966872 (PMC9531674; doi:10.3389/fpubh.2022.966872)
Supplement: Supplementary file 1 [file Data_Sheet_1.zip › Data Sheet 11.docx]

**Practice Materials**

**Scenarios for role play practice calls**

| **Green Flag: Business as Usual.**  **Luisa Esquivel is a 42-year-old woman living with her husband and two children. She lost her job and applied for a stimulus check but hasn’t gotten it yet. She is financially struggling and is needing food, medication assistance, and employment resources.**  **Referrals:**   - **Food Pantries** - **NC MedAssist** - **Employment**   **Situation:**  **First attempt - Answers, is driving, needs to reschedule to tomorrow (but just call right) back)**  **Food: Hasn’t gone, afraid of COVID (2)**  **NC Med: In the process of completing the application, most important (1)**  **Asks about why she hasn’t received her stimulus check yet**  **CM talked about giving her employment resources but she forgets what they talked about**  **COVID Qs, you haven’t really been staying up to date and aren’t familiar with any of the questions** |
| --- |

| **Green Flag:**  **Andrew Hutchinson is an anxious 30-year-old man who works as a part-time waiter and doesn’t have housing. He feels like everything is fine because he can get by living in his car and is reluctant to accept help.**  **Referrals:**   - **SNAP (2)** - **Reinvestment Partners Durham Rental Guide (3)** - **DSS Durham Housing (3)** - **Durham Financial Assistance Handout (1)** - **Lincoln Behavioral Health (4)**   **Situation:**  **1. SNAP: CM completed app, still waiting**  **2. Durham Rental Guide: Hasn’t connected, intimidated by the form**  **3. DSS: Doesn’t feel like he needs it.**  **4. Financial Assistance Handout: Used the executive Order 118**  **5. Talked to CM for a bit over the phone on his initial call but doesn’t want more help**  **COVID Qs: Is pretty informed, listens to the radio in his car** |
| --- |

| **Green Flag:**  **Brianna Rainey is a 38-year-old black woman who struggles with diabetes and is receiving dental care from Lincoln. She previously used the Lincoln voucher but is now referred to NC MedAssist. She received a dental exam from Candace but wants to see her summary before seeing a specialist. She has been out marching all week and is emotionally strained from what is happening regarding the BLM Movement.**  **Referrals:**   - **Food Pantries (2) - used** - **NC MedAssist (1) - not started** - **Financial Assistance (3) - not started**   **Situation:**  **1. Food Pantries (2) - Last call she was referred to Mt. Level Missionary and Greater Orange Grove Baptist Church for food pantries. She gave it a 10/10.**  **2. NC MedAssist (1) - She is in extreme need of paying for her medications but hasn’t completed her application. Nekoba invited her to come fill it out at Lincoln last week but she hasn’t had the chance because she was busy. She hasn’t started the application.**  **3. Financial Assistance (3) - She hasn’t reached out because Nekoba has notified her that none of the resources are offering money.** |
| --- |

**New Patient Follow-up Script Week 2**

- Script is a suggested flow and may vary by patient and patient responses.
- Start with a smile!
- Motivate, inform, and loop back to case manager if necessary

**Voicemail (to set up on Help Desk phones)**

Hello! You’ve reached the Help Desk with Lincoln Community Health Center. If this is a medical emergency, please hang up immediately and dial 911. If you are calling regarding an urgent matter related to your community resource referral, please contact your care team at Lincoln Community Health Center directly at _______. If you are calling to follow-up on a missed phone call, thank you for your call; we’re sorry we missed you! Please leave a voicemail with your name and call-back number at the tone. We will return your call within a week and look forward to speaking soon.

**First Voicemail (to leave on patient phones)**

Hi, I’m calling from Lincoln Community Health Center. My name is _____, and I am a Community Resource Navigator. I just wanted to follow up with you about some resources you were referred to. I’ll plan to call back some time within the week from this phone number! Sorry we didn’t reach you this time. Thanks so much, bye.

**Second Voicemail (to leave on patient phones)**

Hi, I’m calling from Lincoln Community Health Center. My name is _____, and I am a Community Resource Navigator. I just wanted to follow up with you about some resources you were referred to. I’ll plan to call back some time within the week from this phone number! But please feel free to leave me a voicemail or text with your name [pause] and availability [pause]. My number is _______‬. Again, I’m_____ at (phone number)‬. Sorry we weren’t able to reach you at this time. Thanks so much, bye.

**Third Voicemail (to leave on patient phones)**

Hi, I’m calling from Lincoln Community Health Center. My name is _____, and I am a Community Resource Navigator. I just wanted to follow up with you about some resources you were referred to. I’m sorry I haven’t been able to reach you. Please feel free / You’re still welcome to leave a voicemail or text with your name [pause] and availability [pause] at ________‬. Again, that’s (phone number)‬. Otherwise, feel free to reach me through Lincoln in the future! Thanks so much, bye.

1. **Hi, this is _______. Am I speaking with _______?**
2. **Hi, I’m volunteering with Lincoln Community Health Center. My role is to help patients work with community resources for things like [food, financial assistance, housing, etc].**

**I know you had a recent phone call check-in with a case manager from Lincoln [Candace/ D’Nicolole/ Diana/ Nekoba], and I’d like to follow-up on how things are going. I also want to be sure you have important information on the coronavirus. Is now a good time for a brief call?**

1. **Thank you for your time Ms./Mr._________! I’ll start with a few questions. And of course, the information we discuss will be kept confidential.**

**[Your case manager] told me that you were given some resources or information regarding [______specific resource________], [food], [employment resources], [managing stress]...**

***[If 1 referral, SKIP]***

1. **Of your referrals, which one of these do you feel is the most important to you?**

*[pending patient response]*

1. **Ok, let's talk about that one. Were you able to call or connect with _____________?**

**[If no]**

| **Was there a specific barrier or reason that kept you from connecting?**  *[record reason in RedCAP]*  **[If the patient references COVID19, use MI]**  **Yes, that makes a lot of sense. I’ll be sure to share some important information with you about COVID19 before we finish.**  **I know this has been a difficult time for everyone. Social distancing or staying at home is a smart decision, but it’s also good to make sure you’re doing what you can to take care of yourself.**  **Are you still interested in connecting with [the service]?**   - ***[if no]***   ***Ok, I completely understand. I respect those needs and your priorities during this time. (Use MI where appropriate)***   - ***[If yes]*** - **Ok, maybe we can work together to find a way to safely access this resource.**   **→ *[In the information you give, provide up-to-date knowledge, assess the urgency and risk of accessing that resource.]***  ***[If application based]***  *[Provide initial information/description about the resource]*  **I would recommend that you send in your application either through the internet or by mail instead of in-person to minimize risk.**  **(can you remind me, did you CM help you complete the application, mail you the application to complete, or expect you to fill out the application on your own?**   - **If no assistance, potentially use more in my basket service for patients** - **Can always go in person to the DSS** - ***provide application website, phone number for mail-based applications***   ***[If appointment-based or typically in person]***  **Based on the circumstances, this resource is currently *(research CBO)***   - **in full operation** - ***- Please call ahead before arriving at in-person referrals to ensure the operation status has not changed.***    - **Ex: “The organization is currently asking people to call ahead before coming to their appointments to be informed of their new protocol for coming in. They want to know if you are experiencing any symptoms. Although they are in full operation, I would advise practicing every precaution to avoid public areas, stay at least 6 feet away from people, and to avoid touching frequently touched surfaces. Wash your hands frequently, and avoid touching your face.”** - **in altered operation**   - **Ex: “The organization is now switching to online services”**   - **Ex: “The organization is now making people remain in their cars to receive food from the foodbank”**   - **Ex: “Because there are reduced workers, the organization has changed their hours”** - **closed**   - **unfortunately, we will have to navigate another way to access what you need. Let me look through our directory to see if there is maybe another resource you could use.**   ***[If handout]***   - ***As question what they know first*** - ***Ex: I know your CM might have referred you to some FA resources, did you receive an information in the mail or did you talk about this resource with you CM previously*** - **Ex: “Luckily, this service is done over the phone, so you can proceed as usual, but their hours have changed”** - **Ex: “If you refer to your handout, you can access the resource over the internet”**   ***[Continue through all other referrals from CM by order of importance to the patient.]***  **Now, of your other referrals [list the other referrals], which one is the next most important?**  ***[Repeat step 5]***  **Great. Before we end the call, I would like to provide some information about the coronavirus that the Lincoln team and I believe would be helpful for you. *→ COVID*** |
| --- |

***[if yes]***

| **Ease of Use**  **Thanks for sharing. It sounds like [__show that you were listening__].**  **I’m curious to learn how easy it was to use the resource. On a scale from 1-10, 10 being the easiest, how easy was it to use/access [the service]?**  ***[if anything less than 10]*:**  i. I’m interested in your experience. What led you to score this a # = [highly/not] of being useful?  ii. What would have made this experience a 10?    **Utility**  **Thanks for sharing. I understand it was [validate previous response].Now I’m interested to see how useful it was. Once you actually got [the service], on a scale from 1-10, 10 being the most useful, how useful was the service?**  ***[if anything less than 10]*:**  i. I’m interested in your experience. What led you to score this a # = [highly/not] of being useful?  ii. What would have made this experience a 10?  ***[Continue through all other referrals from CM by order of importance to the patient.]***  **Now, of your other referrals [list the other referrals], which one is the next most important?**  ***[Repeat step 5]*** |
| --- |

*** * * * * * * * * ***

***[Transition to COVID19 Information]***

1. **Thanks for your input. Next, our team at Lincoln really wants to make sure that all our patients have the most recent information about COVID-19. I would love to talk a little about that with you given there has been some misinformation going around. So have you been staying up to date about the current outbreak?**

*[pending patient response, show active listening Ex: It really is an unbelievable time****.****]*

**COVID19 Questions**

| **General tips for asking questions about knowledge**  **[If they are wrong about something, kindly correct them]**   - - ex:   - ex: Oh that’s close, but the main ones are actually   **[Segue into each topic, give plenty of positive affirmation when correct, delicately and kindly correct them if wrong]**   - ex: Yes, that’s great. Tell me more about… [insert question] - ex: Ok, sounds good. Let’s talk through…[insert question] - ex: That’s almost correct. Just one detail is that. Can you tell me other [continue prompting the question].   **[Other transitions]**   - I’m glad we talked about that. Maybe we can discuss [this] now. - Besides “Ok, good” or “Ok, great” or “Sounds good” maybe “yeah, those are on the right track”   **[Avoid]**   - Repetition, “I understand”, sometimes things are always “good” or “great”, stay away from evaluative language like “that’s correct” |
| --- |

1. **I’m curious to hear what you’ve learned about the coronavirus so far.**
   1. **I was wondering, if you or a loved one were to show any of the three main symptoms of COVID, which are fever, cough, and shortness of breath, do you have a plan on what you would do?**
      1. Call your doctor: ask what they advise and let them know you have symptoms.

- Stay at home except to get medical care.
- Continue to practice social distancing, but now within your home.

***[document in RedCAP which ones they get correct.]***

***Yes, those are good options. The best practice that I would recommend if you or a loved one becomes ill is to call your doctor. While on the phone, ask what they advise and let them know you have symptoms. Lincoln has advised their patients to call their appointment line at (919) 956-4034. Second, stay at home except to get medical care. Third, continue to practice social distancing. This means avoiding close contact with people at home and sharing personal items. Some additional information that you should know, if you have an appointment (either at Lincoln or somewhere else), it is important to call ahead before going and confirm their protocol. Finally, if you have a medical emergency, be sure to call 911.***

- 1. **So, next I was wondering, what are the main steps you have been taking to protect yourself and others against the spread of the coronavirus?**
  - Practice the 3 Ws:
    - Wear: a mask while in public
    - Wait: at least 6 feet away while social distancing and stay at home
    - Wash: your hands and other surfaces frequently
      - after blowing your nose, coughing, or sneezing; going to the bathroom; and before eating or preparing food
  - Don’t touch your face: eyes, mouth, and nose

***[document in RedCAP which ones they get correct. Fill in missing info]***

***Yes, I find it easiest to remember using the 3 Ws…***

***Optional: Also, there is a texting service from the state of North Carolina that will give you updates and important information. Would you be interesting in receiving text updates:***

***[pending patient respond]***

***All you have to do is text COVIDNC in all caps to 898211 and they will send you information updates.***

***I’m glad we talked about that.***

1. **Alright, do you have any other questions that I can help answer?**
2. **I know we discussed a few things just now. *[give a brief summary of call].* Does this sound correct to you?**
3. **Based on our conversation, where would you like to go from here, in terms of connecting with [service]?**

*[Reflect response about next steps]*

1. **Based on our routine follow up, I think it would be helpful for us to talk again in the next 2 weeks, just to see how things are going and check in on [______________]. Can I call you back again in 2 weeks around this time?**
2. **Thank you so much for your time today Mr./Ms. ___________. I look forward to following back with you in the coming 2 weeks. Have a nice day! Good-bye!**

**New Patient Follow-up Script Week 4**

- Script is a suggested flow and may vary by patient and patient responses.
- Start with a smile!
- Motivate, information giving, and loop back to a case manager

**Voicemail (to set up on Help Desk phones)**

Hello! You’ve reached the Help Desk with Lincoln Community Health Center. If this is a medical emergency, please hang up immediately and dial 911. If you are calling regarding an urgent matter related to your community resource referral, please contact your care team at Lincoln Community Health Center directly at_________. If you are calling to follow-up on a missed phone call, thank you for your call; we’re sorry we missed you! Please leave a voicemail with your name and call-back number at the tone. We will return your call within a week and look forward to speaking soon.

**First Voicemail (to leave on patient phones)**

Hi, I’m calling from Lincoln Community Health Center. My name is _____, and I am a Community Resource Navigator. I just wanted to follow up on our call about 2 weeks ago. I’ll plan to call back some time within the week from this phone number! Sorry I didn’t reach you this time. Thanks so much, bye.

**Second Voicemail (to leave on patient phones)**

Hi, I’m calling from Lincoln Community Health Center. My name is _____, and I am a Community Resource Navigator. I just wanted to follow up with you on our call about 2 weeks ago related to community resources you were referred to. I’ll plan to call back some time within the week from this phone number! But please feel free to leave me a voicemail or text with your name [pause] and availability [pause]. My number is ______. Again, I’m_____ at (phone number). Sorry I didn’t reach you but I’d love to hear from you. Thanks so much, bye.

**Third Voicemail (to leave on patient phones)**

Hi, I’m calling from Lincoln Community Health Center. My name is _____, and I am a Community Resource Navigator. I just wanted to follow up with you on our call about 2 weeks ago related to community resources you were referred to. I’m sorry I haven’t been able to reach you. You’re still welcome to leave a voicemail or text with your name [pause] and availability [pause] at________. Again, that’s (phone number). Otherwise, feel free to reach me through Lincoln in the future! Thanks so much, bye.

**Modified Voicemail (if did not reach on week 2)**

Hi, I’m calling from Lincoln Community Health Center. My name is _____, and I am a Community Resource Navigator. I just wanted to follow up on resources you were referred to. Sorry I still haven’t been able to reach you! I’ll plan to call back some time within the week from this phone number! Thanks so much, bye.

1. **Hi, this is _______. Am I speaking with______?**
2. **Hi, it’s nice to speak with you again. I’m calling from Lincoln Community Health Center and wanted to follow-up on our [chat or voicemail] we had about 2 weeks ago related to [free medication program, food stamps, etc.]. I’m curious to learn more about how things went for you and would love to follow-up. Is now a good time for a brief call?**
3. **Thank you for your time Ms./Mr._________! I’ll start with a few questions. And of course, the information we discuss will be kept confidential.**

***[If 1 referral, SKIP]***

**Last time we talked about [housing and transportation, etc], and you mentioned ______ was most important. I believe we discussed _________ as your next steps. I’d love to hear more about how that went for you!**

*[pending patient response]*

**Were you able to visit or connect with_____________? Have you tried _________?**

***[if patient responds no]***

Are you still interested in connecting with [the service]? I recall last time you mentioned _______.

***[if yes interested, see prompts below]***

Were there any reasons you weren’t able to connect with [the service]?

OR Is there anything we can do together that can assist your efforts?

OR What would you like to get out of an agency referral?

- How do you usually [find solutions to this barrier]? (ie find transportation)
- Can you give me a minute or so to look up that information for you? /

Is it ok if I call you back in a few minutes to pull up [this information] for you?

- - - Do you have a pen and paper available?
    - Would you want to read back the phone number to me to double-check?

***[if not interested, see prompts below]***

Was there anything that made you interested in the initial referral? I’m wondering if anything changed for you that made you change your mind about [the resource].

- I know that the care team at Lincoln thought you would benefit from [this service.]
- Is there anything you’d be willing to do about _____?
- Is there anything you imagine that might change your mind?
- Do you think there’s a reason in the future that might change your mind?
- We understand. If you ever change your mind, feel free to connect with us via Lincoln. We’re always open to listening and working together with folks on their health-related goals.

***[if patient responds with yes, was able to visit/connect with service; collect Ease of Use/Utility]***

**Ease of Use**

Thanks for sharing. What I’m hearing is you were able to get [______]. On a scale from 1-10, 10 being the easiest, how easy was it to use/access [the service]?

(easiest to access) 10 9 8 7 6 5 4 3 2 1

***[if anything less than 10]*:**

i. I’m interested in your experience. What led you to score this a # = [highly/not] of being easy to access?

ii. What would have made this experience a 10?

**Utility**

Thanks for sharing. So glad you were able to connect I understand it was [validate previous response]. Once you actually got [the service], on a scale from 1-10, 10 being the most useful, how useful was the service?

(most useful) 10 9 8 7 6 5 4 3 2 1

***[if anything less than 10]*:**

ii. I’m interested in your experience. What led you to score this a # = [highly/not] of being useful?

ii. What would have made this experience a 10?

***[for multiple referrals]*:**

**5. I know you were also referred to but didn’t connect with [_____], [_____] the last time we talked. I just wanted to be able to share with our team at Lincoln, since then, were you be able to connect with any of [these additional services]?**

***[if patient responds with yes]***

[return to Ease of Use and Utility Questions]

***[if patient responds with no]***

i. We want to be most helpful to you, do you still want to talk about [these other services]?

ii. Is that something I can assist you with?

**6. I’m glad we talked about that. Do you have any questions about [any of your resources]?**

**7. *[if you reached patient at week 2]***

**Last time we talked about the current coronavirus outbreak and you were able to answer some questions about it for me. I just want to remind you again to continue practicing the 3 Ws of “Wear” a mask when out in public, “Wait” at least 6 feet away from others, and “Wash” your hands and frequently touched surfaces on a regular basis. Finally, be sure to call your doctor if you experience coronavirus symptoms. How do you feel about all of this? Do you feel comfortable about taking these steps?**

**7. I know we discussed a couple things just now. [This is what I’ve learned]…do you have any questions about your resources, the coronavirus, or anything else I can help you with?**

**8. Based off our conversation, where would you like to go from here regarding connecting to resources?**

**9. <Reflect response about next steps> Based on our routine follow up, we currently don’t have any other follow-ups scheduled. However, you’re always welcome to reach our team by calling the Lincoln main line and speaking with Behavioral Health.**

**11. Thank you so much for your time today Mr./Ms. ___________. It was wonderful getting to speak with you. Have a nice day! Good-bye!**
