# Supplementary material for: Training student volunteers as community resource navigators to address patients' social needs: A curriculum toolkit
Source: Front Public Health. 2022 Sep 20;10:966872. doi: 10.3389/fpubh.2022.966872 (PMC9531674; doi:10.3389/fpubh.2022.966872)
Supplement: Supplementary file 1 [file Data_Sheet_1.zip › Data Sheet 12.docx]

**Guide for facilitating practice call**

1. Before each practice session, facilitators should read through the patient scenarios to familiarize themselves. Three patient scenarios are given at the end of this document.
2. Facilitators should first help the trainee set up their desktop
   1. REDCap, Script, CBO directory and other potential tabs
3. Facilitators should also review the patient referrals with trainees before starting practice call.
4. One the trainee’s first attempt, pick up the phone but tell trainee you need to reschedule to a better time.
5. After the trainee has successfully rescheduled, have them attempt the call again. In the 2nd attempt, follow the script.
6. After the call, while the trainee is doing post-call data entry, go through the fidelity checklist (appendix K) according to their call performance and write feedback as necessary.
7. Once the trainee completes the post call data entry, check their entry and ask how the call went.
8. Give constructive feedback on their calls.
   1. Tell them what they did well and how they can improve

**Patient scenarios for practice call**

| **Green Flag: Business as Usual.**  **Luisa Esquivel is a 42-year-old woman living with her husband and two children. She lost her job and applied for a stimulus check but hasn’t gotten it yet. She is financially struggling and is needing food, medication assistance, and employment resources.**  **Referrals:**   - **Food Pantries** - **NC MedAssist** - **Employment**   **Situation:**  **First attempt - Answers, is driving, needs to reschedule to tomorrow (but just call right) back)**  **Food: Hasn’t gone, afraid of COVID (2)**  **NC Med: In the process of completing the application, most important (1)**  **Asks about why she hasn’t received her stimulus check yet**  **CM talked about giving her employment resources but she forgets what they talked about**  **COVID Qs, you haven’t really been staying up to date and aren’t familiar with any of the questions** |
| --- |

| **Green Flag:**  **Andrew Hutchinson is an anxious 30-year-old man who works as a part-time waiter and doesn’t have housing. He feels like everything is fine because he can get by living in his car and is reluctant to accept help.**  **Referrals:**   - **SNAP (2)** - **Reinvestment Partners Durham Rental Guide (3)** - **DSS Durham Housing (3)** - **Durham Financial Assistance Handout (1)** - **Lincoln Behavioral Health (4)**   **Situation:**  **1. SNAP: CM completed app, still waiting**  **2. Durham Rental Guide: Hasn’t connected, intimidated by the form**  **3. DSS: Doesn’t feel like he needs it.**  **4. Financial Assistance Handout: Used the executive Order 118**  **5. Talked to CM for a bit over the phone on his initial call but doesn’t want more help**  **COVID Qs: Is pretty informed, listens to the radio in his car** |
| --- |

| **Green Flag:**  **Brianna Rainey is a 38-year-old black woman who struggles with diabetes and is receiving dental care from Lincoln. She previously used the Lincoln voucher but is now referred to NC MedAssist. She received a dental exam from Candace but wants to see her summary before seeing a specialist. She has been out marching all week and is emotionally strained from what is happening regarding the BLM Movement.**  **Referrals:**   - **Food Pantries (2) - used** - **NC MedAssist (1) - not started** - **Financial Assistance (3) - not started**   **Situation:**  **1. Food Pantries (2) - Last call she was referred to Mt. Level Missionary and Greater Orange Grove Baptist Church for food pantries. She gave it a 10/10.**  **2. NC MedAssist (1) - She is in extreme need of paying for her medications but hasn’t completed her application. Nekoba invited her to come fill it out at Lincoln last week but she hasn’t had the chance because she was busy. She hasn’t started the application.**  **3. Financial Assistance (3) - She hasn’t reached out because Nekoba has notified her that none of the resources are offering money.** |
| --- |
