# Supplementary material for: Training student volunteers as community resource navigators to address patients' social needs: A curriculum toolkit
Source: Front Public Health. 2022 Sep 20;10:966872. doi: 10.3389/fpubh.2022.966872 (PMC9531674; doi:10.3389/fpubh.2022.966872)
Supplement: Supplementary file 1 [file Data_Sheet_1.zip › Data Sheet 13.docx]

**Instructions for facilitating virtual shadowing experience**

**During call shift**

1. Facilitators should Join the meeting from both your computer and phone.
2. Mute on on the zoom app.
3. Screen share your desktop lay out and walk through your general pre-call workflow to the new volunteers
4. When you are ready to call, leave the meeting from your computer, but stay on your phone.
5. Before you call the patient, remind the new volunteers about the three questions in during the observation.
6. Call the patient from your computer via Google Voice.
7. After the call, discuss these 3 questions with the volunteer:
   1. How well do I use motivational interviewing techniques?
   2. 2) How well do I communicate specific information about the resource? And
   3. 3) How was my tone and how often did I vary from the script?
8. Go over documentation.
