# Supplementary material for: Training student volunteers as community resource navigators to address patients' social needs: A curriculum toolkit
Source: Front Public Health. 2022 Sep 20;10:966872. doi: 10.3389/fpubh.2022.966872 (PMC9531674; doi:10.3389/fpubh.2022.966872)
Supplement: Supplementary file 1 [file Data_Sheet_1.zip › Data Sheet 14.docx]

**Instructions for facilitating reverse shadowing**

1. Discuss referrals with the new volunteer
2. Have the new volunteer go over the patient and outline of the call
3. Open fidelity checklist
4. Make a copy of the sheet and title it the ‘new volunteer’s name fidelity checklist reverse shadowing 1’
5. Save the new sheet on the folder titled “reverse shadowing fidelity checklist”
6. Check the fidelity checklist as they call and talk with patients
7. After the call, have the new volunteer document and check their documentation
8. Go over the call and suggest any feedback or tips.
9. Follow these steps for all three calls
