# Supplementary material for: Training student volunteers as community resource navigators to address patients' social needs: A curriculum toolkit
Source: Front Public Health. 2022 Sep 20;10:966872. doi: 10.3389/fpubh.2022.966872 (PMC9531674; doi:10.3389/fpubh.2022.966872)
Supplement: Supplementary file 1 [file Data_Sheet_1.zip › Data Sheet 16.docx]

*Appendix 16*

**Community Resource Navigator Training Evaluation**

**Volunteer Information**

1. Which school are you in? Please circle one.

Trinity College of Arts and Sciences

Pratt School of Engineering

School of Nursing

1. What is your major or program of study? ________________
2. When is your expected graduation year. Please circle one. 2021, 2022, 2023, 2024.

**Long Answer Questions**

*Please respond to the following questions in 3-6 sentences. Please be specific and answer in complete sentences.*

1. Describe your key takeaways from the training program, including new knowledge, skills, or personal reflections.
2. How do you see yourself using what you’ve learned during training in your future educational and professional career, if at all?
3. To what extent did the training prepare you, or not, to provide navigation support to help patients connect to community resources. You might comment on your ability and confidence to use the call script, the resource directory, enter and document data, communicate with patients and peers, etc.
4. Briefly list the topics you wish we covered or spent more time on.
5. Due to the COVID-19 pandemic, our community resource training was entirely virtual. Which components of the virtual training were particularly helpful and why? Which components of training could be improved in the future and how?
