# Supplementary material for: Training student volunteers as community resource navigators to address patients' social needs: A curriculum toolkit
Source: Front Public Health. 2022 Sep 20;10:966872. doi: 10.3389/fpubh.2022.966872 (PMC9531674; doi:10.3389/fpubh.2022.966872)
Supplement: Supplementary file 1 [file Data_Sheet_1.zip › Data Sheet 2.docx]

**Recruitment blurb that was sent to organizations or posted on social media**

Have your summer plans changed because of the COVID-19 pandemic? Would you like to help families in Durham connect to community resources during the Pandemic? Do you want an applied experience working with patients? Are you interested in the social determinants of health and health disparities?

Become a community resource navigator with the Help Desk team this summer! Help Desk is a student volunteer program in partnership with Lincoln Community Health Center in Durham. Our community resource navigators help patients over the phone to connect to local community resources that address non-medical needs, like food and housing insecurity.

Due to the COVID-19 pandemic, volunteers this summer will no longer be required to be in Durham over the summer and can fulfill their duties from their homes or current location.

**We are recruiting English and near-native/native Spanish speakers to serve our patients as a part-time volunteer position (5-7 hours/wk) this summer.**

**Community Resource Navigator Application**

*Note: Community Resource Navigators will be required to undergo a 30 minute interview as well as a background check and drug test administered by Lincoln before receiving an invitation to join the volunteer program.*

**Background**

First Name: Last Name: Preferred Name (optional):

Email: Cell Phone:

How did you first hear about the Community Resource Navigator program?

| Friend |  |
| --- | --- |
| Advisor/Professor |  |
| Organization (inc. email) |  |
| Other (please specify) |  |

**Education**

Graduation Year:

School (Trinity/Pratt):

Primary Major:

Secondary Major:

Minors, Certificates:

**Experience**

Have you previously volunteered with Lincoln Community Health Center (Y/N)?

Have you had any previous volunteer experience in a healthcare setting (Y/N)?

Have you had any previous volunteer experience (Y/N)?

Please list up to 5 previous Volunteer/Community Service experiences, ordered by significance of involvement.

| Volunteer Role Title (inc. Brief description of responsibilities) | Organization Name | Dates of Service |
| --- | --- | --- |
|  |  |  |
|  |  |  |
|  |  |  |
|  |  |  |
|  |  |  |

Please list any additional educational, personal, or professional experience that you would like us to consider in your volunteer application, including but not limited to social determinants of health/health systems coursework and Durham community volunteer work/advocacy):

|  |
| --- |

We are recruiting for both English and Spanish-speaking volunteers:

Can you speak fluently or read/write Spanish (Y/N)?

Are you a native speaker (Y/N)?

**Availability**

Note: All Community Resource Navigators will be expected to regularly commit a minimum 6 weeks of volunteering at 5-10 hours/week during the summer, including follow-up calls, data entry, and training meetings. Virtual training will be held on **insert training dates**

Are you able to attend the **insert training day and time**. (Y/N)?

Are you able to attend the **insert training day and time**. (Y/N)?

Are you able to attend the **insert training day and time**. (Y/N)?

Please complete the following table about your summer volunteering availability. We realize plans may change, so fill to the best of your ability and we can work out details later.

| Week | Week Availability (Y/N?) | Days of week & hours of day available | Notes about availability |
| --- | --- | --- | --- |
| 5/3 |  |  |  |
| 5/10 |  |  |  |
| 5/17 |  |  |  |
| 5/24 |  |  |  |
| 5/31 |  |  |  |
| 6/7 |  |  |  |
| 6/14 |  |  |  |
| 6/21 |  |  |  |
| 6/28 |  |  |  |
| 7/5 |  |  |  |
| 7/12 |  |  |  |
| 7/19 |  |  |  |
| 7/26 |  |  |  |
| 8/2 |  |  |  |
| 8/9 |  |  |  |
| 8/16 |  |  |  |
| 8/23 |  |  |  |

**Short Answer**

Please answer the following questions. Each response should be limited to 1-2 paragraphs.

1. Why do you want to become a Community Resource Navigator?
2. Why do you think you would make a good Community Resource Navigator? (Please include specific qualifications and experiences.)

**Resume**

**Please attach the most recent version of your resume with your application submission.**

Thank you for your interest in the Community Resource Navigator position at Lincoln Community Health Center! We look forward to reading your applications. Within 1 week of the written application deadline, we will be extending 30-minute interview invitations for select candidates. Please keep an eye on your email inbox! Final volunteer decisions will be made shortly after interviews.

**Screening Criteria**

When screening applicants, we kept in mind the following criteria before sending invitations to interview:

**Demonstrated Interest in SDoH and addressing health disparities**

We favored applicants who demonstrated interest and familiarity with SDoH/health disparities either through coursework or professional/ personal experiences.

**Demonstrated Dependability and Commitment**

We favored applicants who were able to demonstrate sustained commitment or follow-through with a previous extracurricular or service activity.

**Prior Experience Work with Patients (Especially Over the Phone)**

We looked for applicants who had previous experience working with patients, especially over the phone.

**Language**

Given that many of our patients indicate that Spanish is the language they are most comfortable with, we favored applicants with Spanish fluency.

**Able to relate to our patients**

Considering that our partner site, Lincoln Community Health Center, serves patients who are predominantly racial and ethnic minority and/or under the federal poverty level, we aimed to recruit volunteers who represent the population we serve.

**Interview**

During our virtual interview, we assessed the applicant's ability to communicate in a clear and concise manner, while also demonstrating empathy. For this we had the applicants practice a mock call, in which they help a fellow student connect to resources around campus. This helped us evaluate their ability to deviate from the script and provide tailored information based on an individual’s response and specific situation.

**Interview Questions**

1. **Intro:** Tell us a little bit about yourself?
2. **Intent**: What drew you to apply for this volunteering opportunity?
3. **Interest**: Can you tell us about your interests or previous experiences related to the social determinants of health or community health?
4. **Dependable/Commitment**: Can you describe a time in which you have demonstrated sustained commitment or follow-through with an extracurricular or service activity?
5. **Diversity/Adversit**y: The Lincoln patients we call are often uninsured, have incomes at or below the federal poverty level, are mostly racial and ethnic minorities, and face many difficult circumstances ranging from discrimination, poverty, food or housing insecurity, mental health issues, and difficulty accessing medical care. How have your personal or professional experiences prepared to work with or relate to this specific patient population?
6. **Empathy**: Can you describe a time when you’ve talked with someone who is stressed, emotional, or clearly going through a difficult time? What was your approach to responding to their concerns? It can be a close family or friend, a peer or co-worker, a client, or patient you’ve worked with.

**Logistics questions**

- Are you available for 8 hours of training week of 5/11? If so what times?
- What is your availability this summer? On your application it says for available XYZ times… is this still the case?
- Are you available in the fall to volunteer in the fall semester?

**We looked for the following skills during the mock call:**

1. Are they able to navigate the script?
2. Are they able to improvise?
3. Are they able to respond to veering off the script?
4. Are they able to sound genuine and not robotic?
5. Do they sound emphatic?
6. Do they sound nice over the phone?
7. Are they respectful?
8. Are they patient with you?
