# Supplementary material for: Training student volunteers as community resource navigators to address patients' social needs: A curriculum toolkit
Source: Front Public Health. 2022 Sep 20;10:966872. doi: 10.3389/fpubh.2022.966872 (PMC9531674; doi:10.3389/fpubh.2022.966872)
Supplement: Supplementary file 1 [file Data_Sheet_1.zip › Data Sheet 3.docx]

**Recommended Training Timeline and Agenda**

- **Module 1: Introduction to SDOH and health equity.** This module will introduce trainees to Social Determinants of Health (SDOH) and health equity, history of Help Desk intervention, and the Lincoln Community Health Center (LCHC). Total time: 2 hours.
  - Part 1: Welcome and introductions (15 minutes)
    - Facilitators and new volunteer introductions. (10 minutes)
    - Training overview and timeline. (3 minutes)
  - Part 2: Introduction to SDOH and health disparities in Durham (45 minutes)
    - Introduction to SDOH & health equity. (20 minutes)
    - Health disparities at a local level. (25 minutes)

***Water/bathroom break (5 min)***

- - Part 3: What is Help Desk, where do we work, and how do we fit in the whole care model? (30 minutes)
    - Interventions to address SDOH in the clinic setting (10 min)
    - Introductions to partner site: Lincoln Community Health Center (10 min)
    - Introduction to PRAPARE and Help Desk intervention (10 min)
- **Module 2: Needs, referrals, and resources.** This module will cover the types of community based resources (CBOs), how to navigate CBO directory, and introduce learners to the most referred resources during and before COVID-19 pandemic. Total time: 2.5 hours.
  - Part 1: Workflow and PRAPARE (20 minutes)
    - Nuances of the PRAPARE form (5 min)
      - Slideshow/example PRAPARE
    - Documentation process on REDCap (15 minutes)
      - Precall Data entry and scheduling demonstration video
  - Part 2: How resources vary. This part will cover what types of resources exist and the different ways patients and access them. (15 minutes)
    - Variety of ways people can access (application-based, warm handoff, telephonic, in-person) (10 minutes)
      - Brief overview of each type, how they generally work, timelines, and examples
    - CBO directory walkthrough: (5 minutes)
      - Trainees visit the directory and learn to navigate the directory and familiarize themselves with the format

***5 minute break***

- - Part 2: Deep dive into most popular resources. This activity will go deeper into some common resources and how to support patients who are referred to these. 1 hr 40 minutes)
    - Trainees research information about common referrals for each of six need domains in breakout rooms and present to the whole group.
  - Part 3: Overview of the homework (10 minutes)
    - Go over logistics of each of the homework materials and expectations for next module
- **Homework assignments** (Estimate total time 2 hr 35 minutes)
  - Motivational interviewing videos (youtube videos)
  - Listen to call demonstration (-30 minutes)
  - Read through the call script (~15 minutes)
  - Practice pre-call data entry (~10 minutes)
  - Skim volunteer handbook (~10 minutes)
  - CITI ethics training (~1 hour)
- **Module 3: Making the call.** This module will cover the logistics and language of making a call. Additionally, it will give trainees an opportunity to practice making calls and document it. Total time: 3 hours.
  - Part 1: Debrief on the homework. (15 minutes)
  - Part 2: Presentation on step-by-step instruction to setting up the call, purpose and goals of calling, and documentation. (45 minutes)

***5 minute break***

- - Part 3: Role play activity to practice calling and documentation ( 1 hour 50 minutes)
    - Breakout room groups with two new volunteers and 1 experienced volunteer.
      - Practice PRAPARE data entry (two PRAPAREs)
      - Call prep
        - Look at the directory/research resources
      - Two calls per new volunteer (1 difficult and 1 easy)
        - An Experienced volunteer will act as the patient, 1 new volunteer will observe, another will make the calls.
      - Call debrief with the bigger group
  - Part 3: Closing comments and next step (10 min)
    - Scheduling shadowing times and next steps

**Practice calls:** Trainees complete three practice calls with an experienced volunteer

- Trainee completes pre-call data entry for hypothetical patients
- Experienced volunteers act as patients
- Feedback and reflection

**Shadowing:** Trainees shadow experienced volunteers call three real patients

- Go over key points to look for during the call
- Experienced volunteer calls, trainee listens
- Debrief on the call going over the key points

**Reverse shadowing:** New volunteers calls three real patients under supervision of an experienced volunteer

- Go over the call and referrals together
- Newly trained volunteer calls patients
- Debrief on the call and provide feedback
