# Supplementary material for: Training student volunteers as community resource navigators to address patients' social needs: A curriculum toolkit
Source: Front Public Health. 2022 Sep 20;10:966872. doi: 10.3389/fpubh.2022.966872 (PMC9531674; doi:10.3389/fpubh.2022.966872)
Supplement: Supplementary file 1 [file Data_Sheet_1.zip › Data Sheet 4.docx]

Help Desk:

Training Undergraduate and Nursing Students to Become Community Resource Navigators


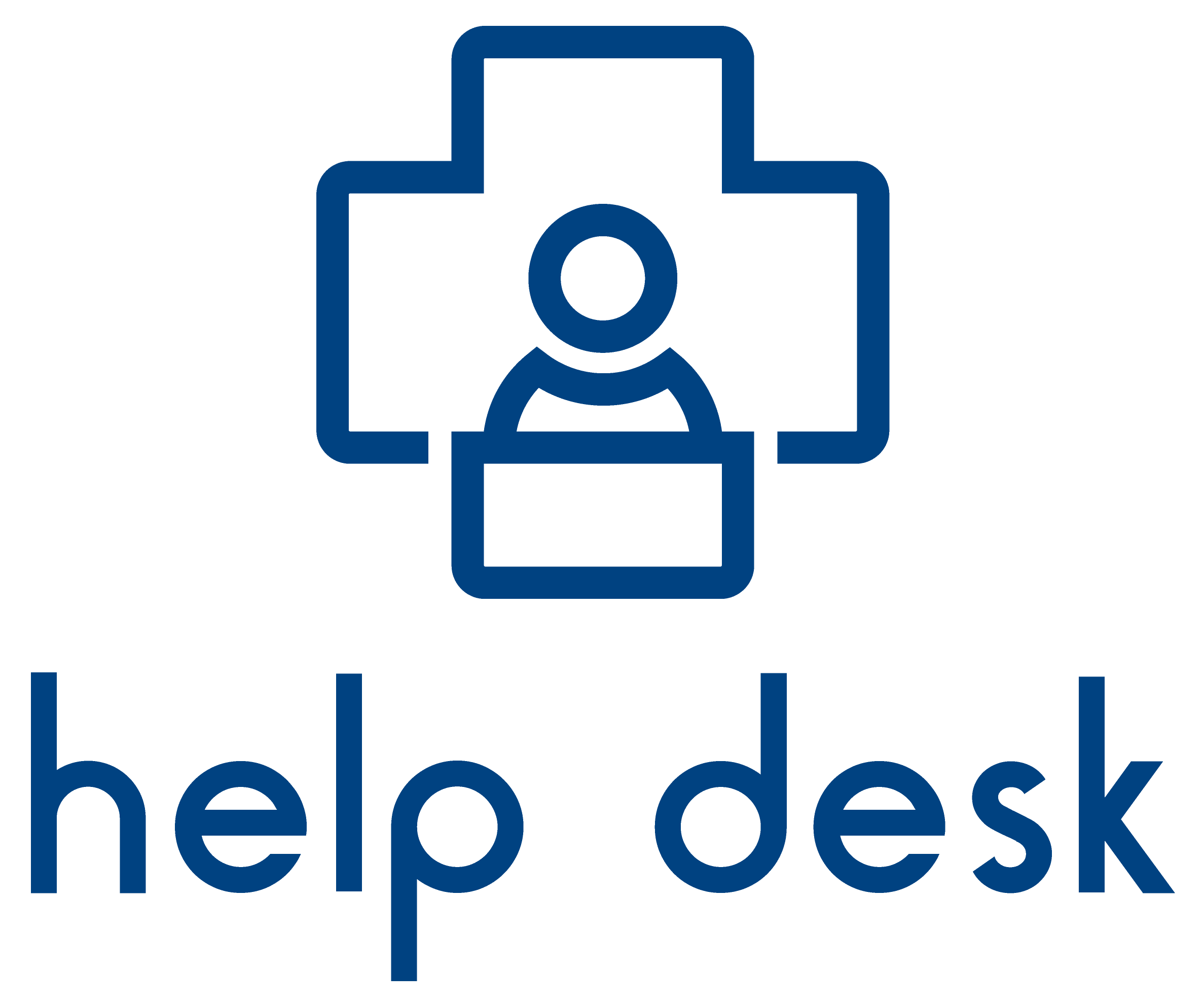


Facilitator Guides for Didactic Sessions

May 2020

Session 1: Social Determinants of Health and Health Equity

**Section 1: Intro to SDoH**

| Time: 20 minutes  Learning Objective**:**   - Define and give examples of health, community, and social determinants of health - Identify and describe unmet social needs - Describe how unmet social needs of the community needs can impact an individual’s personal health   Supplies:   - Slide 7-23 from Introduction to SDoH and Help Desk slide deck   Handout(s):   - None   Preparation:   - Review SDoH before hand - Watch the relevant videos that will be presented |
| --- |

1) SDoH (25 minutes)

- Slide 9:
  - Present slide in fullscreen
  - We just want to start the big picture question of what does the following sentence mean to you?
    - “Health starts in our homes, schools, and communities.”
    - This is a statement from the WHO, anyone have any thoughts on what this means to them?
  - Wait a few minutes to hear from the participants
- Slide 10:
  - WHO defines health as “A state of complete physical, mental, and social well-being and not merely the absence of disease or infirmity.
    - Continue onto next slide
- Slide 11:
  - And then they define Social Determinants of Health as:
    - **“The conditions in which people are born, grow, work, live, and age, and the wider set of forces and systems shaping the conditions of daily life. These forces and systems include economic policies and systems, development agendas, social norms, social policies and political systems”**
- Slide 12:
  - When we are talking about Social Determinants of Health there are six different categories:
    - Economic stability, Neighborhood and physical environment, Education, Food, Community and social context, and Health care systems. These six types of social determinants lead to health outcomes such as Mortality, Morbidity, Life Expectancy etc.
    - Can go more in depth on each of the categories if time permits.
- Slide 13:
  - Here is a more visual representation of an individual’s health is affected by social determinants. In the innermost circle we have the individual, their age, sex, and hereditary factors, outside it is their lifestyle, and then their social and community networks, and then factors such as education, employment and housing.
    - Provide specific examples if needed
- Slide 14:
  - Lot of new research shows that what goes into making an individual healthy actually doesn’t have a lot to do with healthcare.
  - We now know that only 20 percent of a person’s health and well-being is related to care and quality of Service.
  - The other 80 percent of health outcomes is actually driven by the physical environment, social determinants, and behavioral factors.
- Slide 15-18:
  - Let’s look closely at what this actually looks like for a patient.
  - Suppose Maria is a patient, who is probably low-income and does not have health insurance.
  - She is struggling to manage her diabetes and hypertension. In addition, she is a Spanish speaker, making it difficult for her to navigate the health system.
  - She also does not have access to reliable transportation, healthy foods, and stable housing.
  - All of these factors together make her more stressed and exacerbate her condition.
- Slide 19:
  - Lets differentiate between some key terms that often get misunderstood.
  - When we say Social Determinants of Health, we are often referring to upstream factors. These are community-level factors that affect everyone, these are neither negative or positive.
  - We do not say one has more social determinants of health than other, there are different factors that can be affecting one's health positively or negatively.
  - SDoH is a more broad term than social risk factor, which often refers to individual-level factors that could negatively affect a person’s health. This could be something like an individual’s status on food security.
  - We can identify one’s social risk factors on something like a social needs screening.
  - Social need depends on an individual’s preferences and priorities.
  - A screening tool could identify social risk factors, but patient might have preferences on which factors are actually “needs”
- Slide 20:
  - Here is a more visual representation of how social needs can be addressed at an individual and a community level.
  - More recently more and more people have grown interested in investing at a community level to bring change that can affect health in a community.
  - They are trying to look at the root causes of these disparities.
- Slide 21:
  - This is a video showing the kind of choices a low-income patient has to make when shopping.
  - Discuss, ask participants their thoughts on the video.
- Slide 22:
  - So how do SDoH and social needs impact health?
  - There are few mechanism that this could happen:
    - Social needs and directly impacts one’s health
      - Bad air pollution-worsening asthma
    - Unmet social needs can affect health related behaviors
      - If one lives in a neighborhood with a park-limited physical activity
      - Or does not have a nearby grocery store that limits options to get healthy foods
    - Lot of these unmet social needs combined lead to chronic stress that actually brings physiological changes at a molecular and cellular level.
- Slide 23:
  - Here is a schematic shown in one recent paper published. Here we see that how education affects health knowledge which affects diet and exercise habits, it can also affect type of work which determines if you are exposed to hazards or have health insurance etc.

**Section 2: Disparities at a Local Level**

| Time: 50 minutes  Learning Objective:   - Understand variations in the prevalence of health and social needs in local community   Supplies:   - Slide 24 & 25 from Introduction to SDoH and Help Desk slide deck - Durham Compass activity lesson plan   Handout(s):   - Durham Compass activity guide   Preparation:   - Research website/data that highlight the disparities in your local community - Explore: <https://compass.durhamnc.gov/en> |
| --- |

Durham Compass Activity

- This activity was designed to be specific to Durham, North Carolina. The details of the activity will not be applicable to sites outside of Durham. We recommend you identify a resource similar to Durham Compass in your community and use our guide as a template to design an activity specific to your community.
- Divide participants into group of 3 or 4 individuals
- If training students virtually, preset Zoom Breakout rooms.
- Send the activity guide with set by set instruction to each participant.

**Section 3: What is Help Desk**

| Time: 35 minutes  Learning Objective:   - Explain how health care organizations can assess and address their patients’ unmet social needs - Understand the history and goals of the local Help Desk Program   Supplies**:**   - Slide 27 to 64 from Introduction to SDoH and Help Desk slide deck   Handout(s):   - None   Preparation:   - Research website/data that highlight the disparities in your local community - Explore: <https://compass.durhamnc.gov/en> - Be familiar with recent updates on your community regarding health development - Be familiar with the history of your program |
| --- |

Role of Help Desk

- Slide 27:
  - Now we are going to learn about what health systems do to address the problems we have been discussing.
- Slide 28:
  - Recently there has been a big push on how health systems can address these problems. Here are some recent studies published that are evident of this.
    - Show a few recent articles and give a brief overview
- Slide 29:
  - Health care hasn’t always thought about social needs and the importance of integrating it into the delivery of health care.
  - A recent report from the National Academies of Sciences, Engineering, and Medicine highlighted what it means to integrate social and medical care.
- Slide 30:
  - The report highlighted five health care activities to better integrate social care: Awareness, Adjustment, Assistance, and Alignment, Advocacy.
  - Awareness: We need to know at the patient and community level, what do the needs look like.
  - Adjustment: Once you are aware of the need, you can adjust the mode of delivery to accommodate their specific needs. For example, if someone doesn’t have transportation, a telehealth visit maybe better.
  - Assistance: Giving them help for their needs. This would be like giving a transportation voucher to someone who doesn’t have access to a car.
  - Alignment: Align with social services, invest in community programs.
  - Advocacy: Advocating for laws to address social cares at a policy level. For example, speaking out for better housing policies.
- Slide 31:
  - A written description of the 5As we just talked about.
- Slide 32-38:
  - So what is Happening in NC?
  - **Slides discuss the changes brought on by NC-Care 360. Presenters should include information relevant to their state.**
- Slide 39:
  - How does this all relate to becoming a Help Desk Community Resource Navigator?
  - **Transition to discuss the history or motive of your specific program. **
- Slide 40-43:
  - **Describe the history specific to your program**
- Slide 44-45:
  - **Introduce your clinic partner site**
  - **Discuss their location, population they serve, history of the partnership, and workflow**
- Slide 50-64: Introduction to PRAPARE and Help Desk workflow
- Slide 50:
  - The case managers at Lincoln are screening patients for SDoH using The Protocol for Responding to and Assessing Patients’ Assets, Risks, and Experiences (PRAPARE).
  - PRAPARE is part of a national effort to help health centers and other providers screen for unmet social needs and SDoH.
- Slide 51:
  - **Example PRAPARE, briefly walk through**
  - In general case manager’s are screening patients for factors such as education, employment, insurance, income, stress etc using the PRAPARE form.
- Slide 52:
  - **Specific to Lincoln Community Health Center**
  - The PRAPARE tool is also on the EHR at Lincoln
- Slide 53:
  - Highlights a paper published by the Durham Help Desk describing how PRAPARE is utilized at Lincoln.
- Slide 54:
  - Role of Help Desk volunteer is to follow-up with patients after they are screened and are referred to community resources.
- Slide 55:
  - If we as students are able to follow-up after the referral, we hope to improve understanding of the referral and build self-efficacy and increase the use of community resources.
  - We hope as a community resource navigator you will also develop your communication skills and knowledge on social determinants of health to better prepare yourself to contribute to the community health system.
- Slide 56:
  - As a volunteer, you will follow-up with patients to assess the success of connection with community-based resources
  - You will provide information to patients to troubleshoot reported barriers and problems
  - Identify gaps in local community
  - And maintain a updated community resource directory
- Slide 57-59:
  - You will ask if the patient was able to connect to the resource and assess the ease of use and usefulness of the resource.
  - If they haven’t, identify barriers and help troubleshoot
- Slide 60-63:
  - **Help Desk Data, present data specific to your program if available.**
  - **Common referrals, needs are good to show**
- Slide 64-67:
  - Closing remarks and overview of the upcoming training.

Session 2: Needs, Referrals, and Resources

**Section 1: Workflow and PRAPARE**

| Time: 20 minutes  Learning Objective:   - Learn how to complete pre-call data entry   Supplies:   - PRAPARE and CBO directory training slide deck - Entering data on PRAPARE demonstration video   Handout(s):   - None   Preparation:   - Prepare a data entry demonstration video if your documentation process varies |
| --- |

1) Workflow and PRAPARE (20 minutes)

- Slide 7-10:
  - This is what the PRAPARE form that case managers at Lincoln use to screen patients for needs and refer them to resources
- Slide 11:
  - **The specifics of this presentation may vary as different programs have different workflow and setting**
  - Here is general PRAPARE workflow:
  - Case managers at Lincoln complete PRAPARE forms with patients, either in person or over phone.
  - A designated case manager scans and uploads the completed PRAPARE to Dukebox
  - Then a volunteer coordinator assigns data entry of PRAPARE to an individual volunteer
- Slide 12:
  - **The specifics of this presentation may vary as different programs have different workflow and setting**
  - Now, your role is to open Google Sheets to check which patients you are assigned to enter onto REDCap
  - Go to Dukebox and open the file (DO NOT DOWNLOAD the forms to your computer). Make the window smaller and shift it to one side of your screen
  - Open REDCap on another window. Make the new smaller and shift it to the other side of your screen
  - Create a New Record ID on REDCap
  - Enter patient information in the appropriate boxes.
- Slide 14:
  - Lets watch a demonstration video showing this workflow in action
  - Example video on Appendix 4C

**Section 2: How Resources Vary**

| Time: 15 minutes  Learning Objective:   - Describe Community Based Organizations - Distinguish ways in which resources and referrals vary - Become comfortable navigating the CBO directory   Supplies:   - PRAPARE and CBO directory training slide deck - CBO directory training activity guide   Handout(s):   - None   Preparation:   - Compile a resource directory of local organizations, services, and resources - If available, collect information on most common referrals |
| --- |

How Resources Vary

- Slide 16:
  - Here is the flowchart showing most common referrals
- Slide 17:
  - The patients we follow-up from Help Desk are often referred to various community based resources.
  - These resources vary, some are internal to Lincoln, some are external
  - Some are governmental programs, some are small local-non-profits
  - Sometimes patients are referred to one specific resource and sometimes they are given a list of resources.
  - ***Explain how resources vary in your site***
- Slide 18:
  - ***Ten seconds thought on how resources vary**
  - Given this variation in resource types, how would that change the way to follow-up with a patient about their referral?
  - What information would you want to know before you called a patient?
  - ***Pose these questions to the group, give a few seconds to think about it and hear their thoughts***
- Slide 20-27:
  - These slides cover different ways patients can access resources in Durham.
  - In Durham, we identified four main ways patients can access: Choosing from a list, applications, phone call, and in-person.
  - For each way, we describe how it was happening before the COVID-19 pandemic, and how it has changed during the pandemic. Additionally, we provide examples of some community resources that offer their assistance via the specific way
- Slide 29-31:
  - Exploring the directory and becoming familiar with its format
  - ***The directory and its format will vary from site to site***
- Slide 32:
  - Send links to the directory to participants.
  - Give them 3-5 minutes to students to familiarize themselves with the directory
  - After exploration, ask participants how the format of directory could be improved and what is good as it is.
- Slide 33:
  - Update on Durham directory going online

**Section 3: Deep Dive into Most Common Needs and Referrals**

| Time: 1 hour 40 minutes  Learning Objective:   - Recognize some of patients most common needs - Have general knowledge about the commonly referred resources   Supplies:   - PRAPARE and CBO directory training slide deck - CBO directory training activity guide   Handout(s):   - Appendix 4B: CBO directory training activity guide   Preparation:   - Identify most common needs - Identify top referrals to address a specific need - Divide participants into group of 3-4 and assign a referral to each group for each need domain |
| --- |

Deep Dive into most common needs and referrals:

*******These set of slides go hand in hand with the CBO directory training guide***

*** The details of the needs, resources are specific to Lincoln Community Health Center and Durham, North Carolina. Please use these slides as templates to include details specific to your site***

- Slide 35-65:
  - Activity to dive deep into the specific resources in the directory
  - We identified eight most common needs of the referred patients
  - We presented basic overview of the resources
  - Identified four resources in the directory that address the specific need and assigned a group of 3-4 students to a specific resource
  - In breakout rooms, students research the resource and present to the whole group.
  - Refer to the CBO training activity lesson plan
- Slide 66:
  - We described some referrals that are most common during the COVID-19 pandemic
- Slide 68:
  - Go over homework and to-dos
