# Supplementary material for: Training student volunteers as community resource navigators to address patients' social needs: A curriculum toolkit
Source: Front Public Health. 2022 Sep 20;10:966872. doi: 10.3389/fpubh.2022.966872 (PMC9531674; doi:10.3389/fpubh.2022.966872)
Supplement: Supplementary file 1 [file Data_Sheet_1.zip › Data Sheet 5.PPTX]

## Slide 1
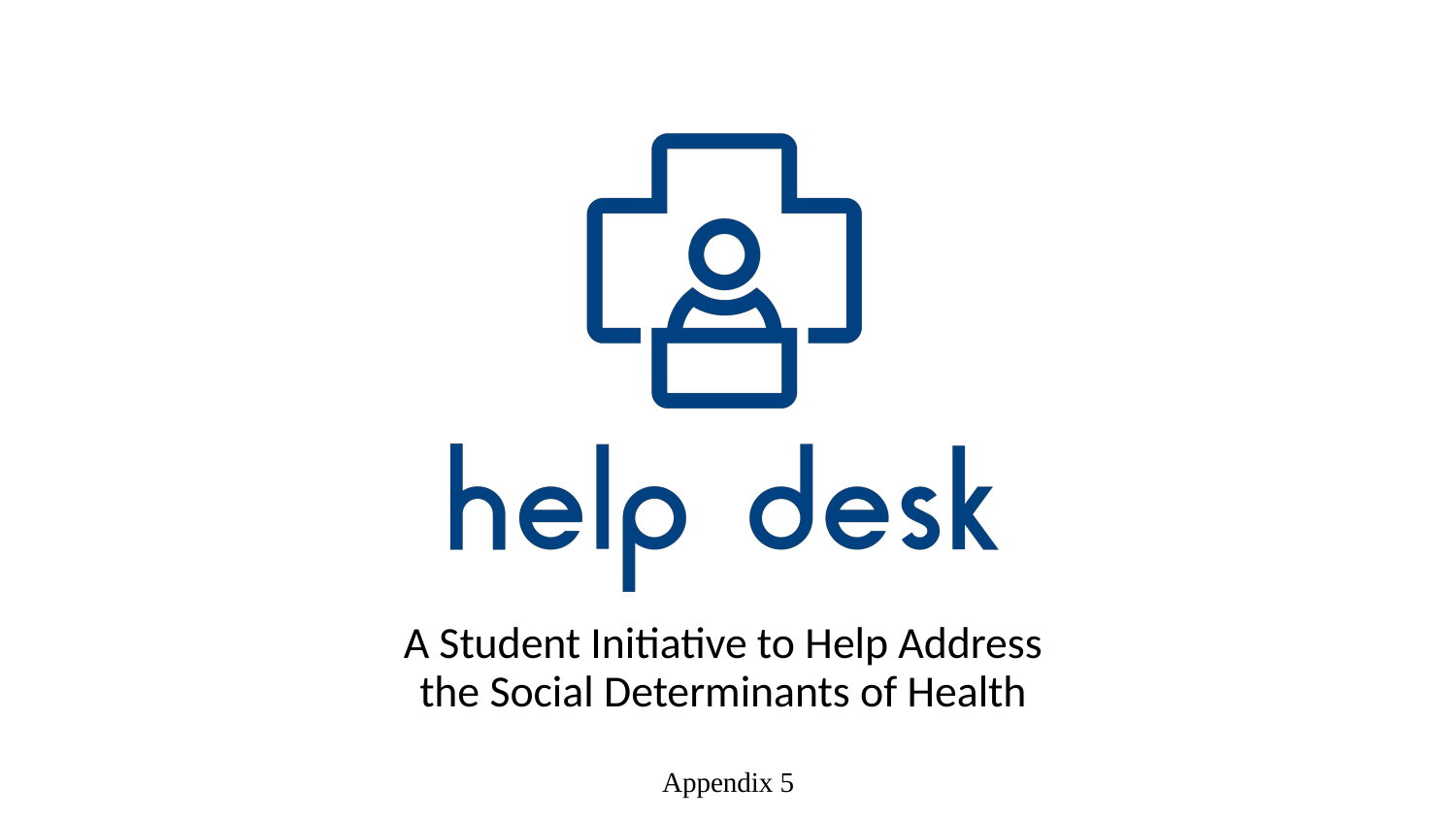

A Student Initiative to Help Address
 the Social Determinants of Health
Appendix 5

## Slide 2
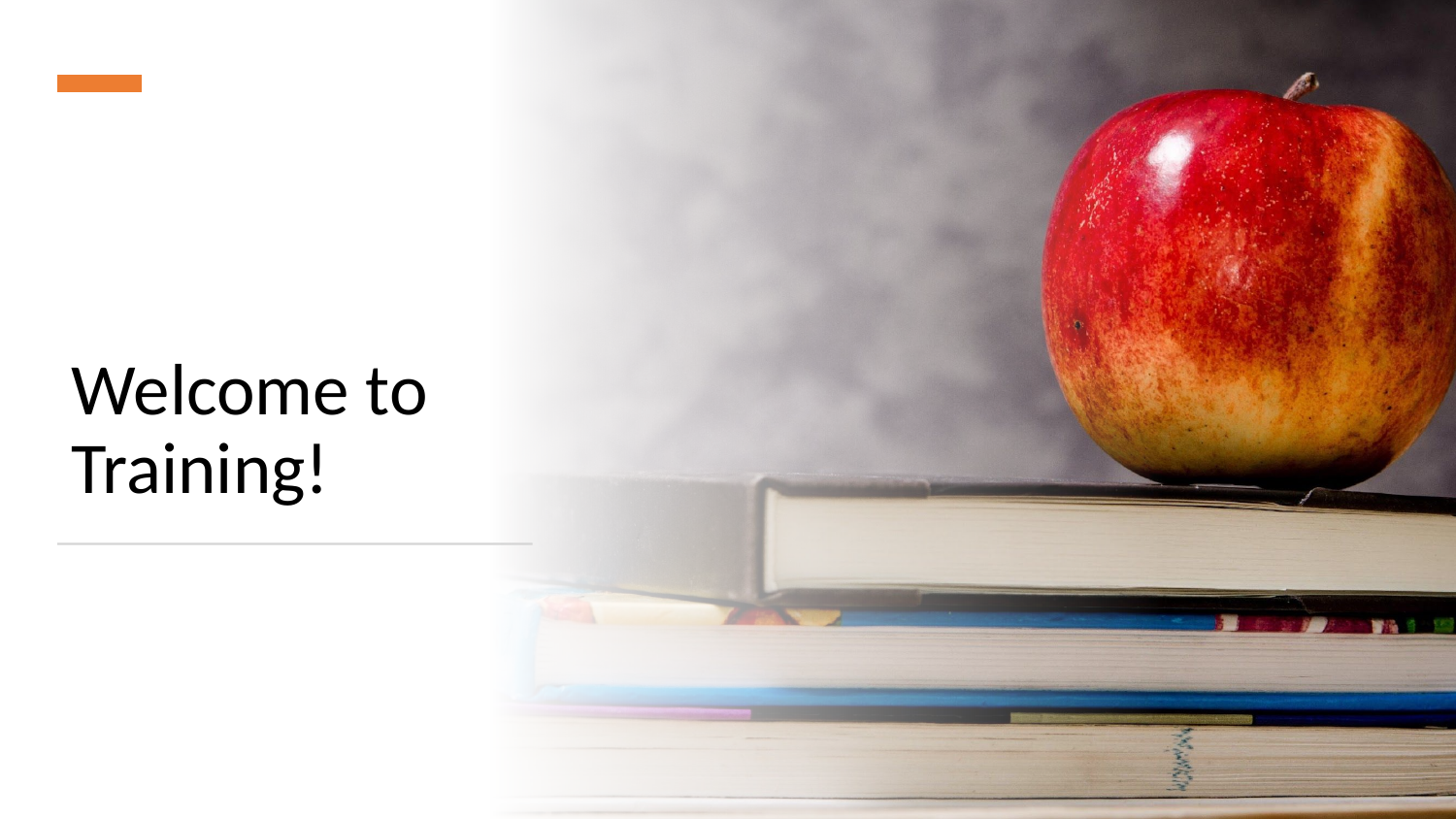

# Welcome to Training!

## Slide 3
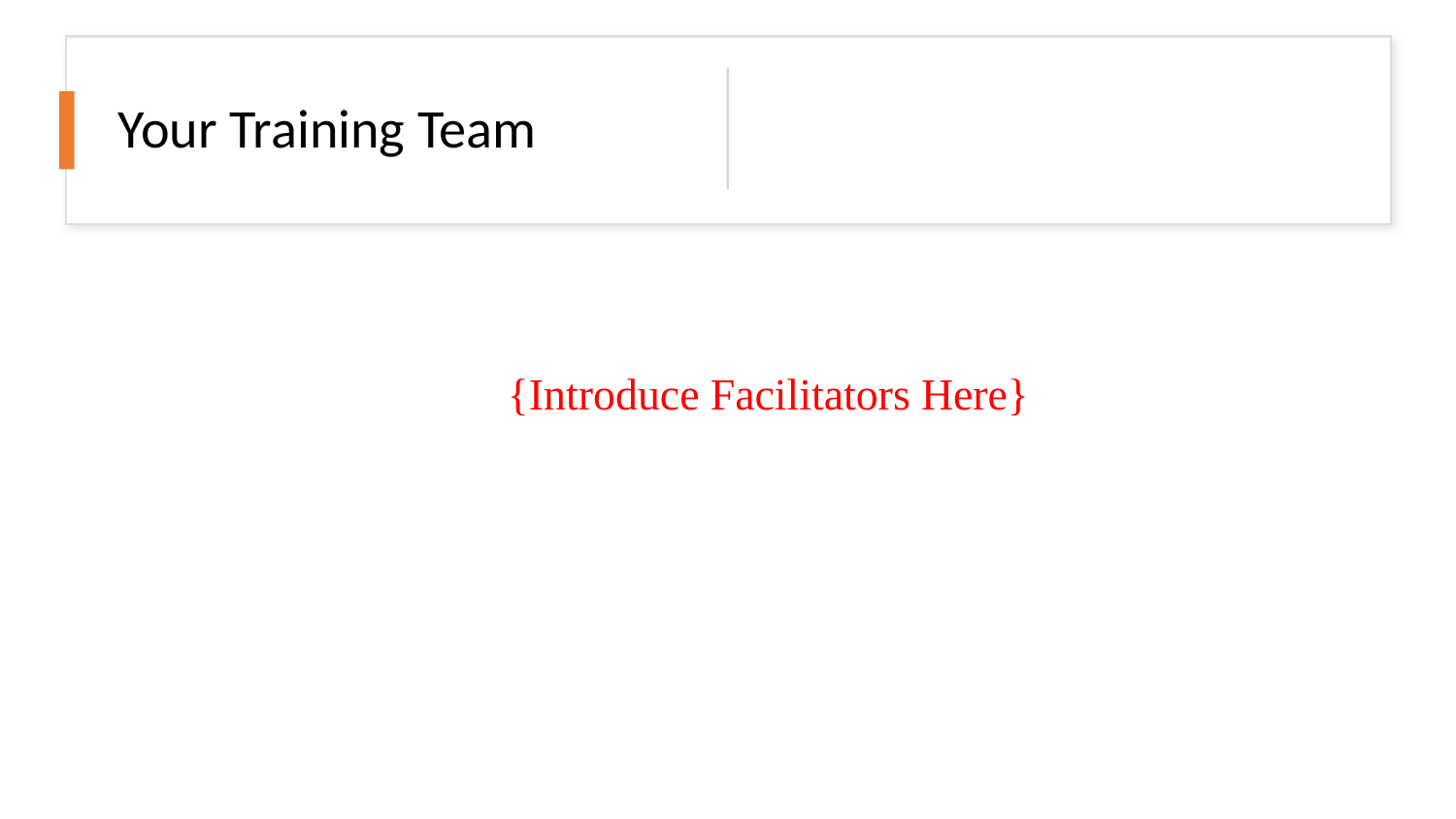

# Your Training Team
{Introduce Facilitators Here}

## Slide 4
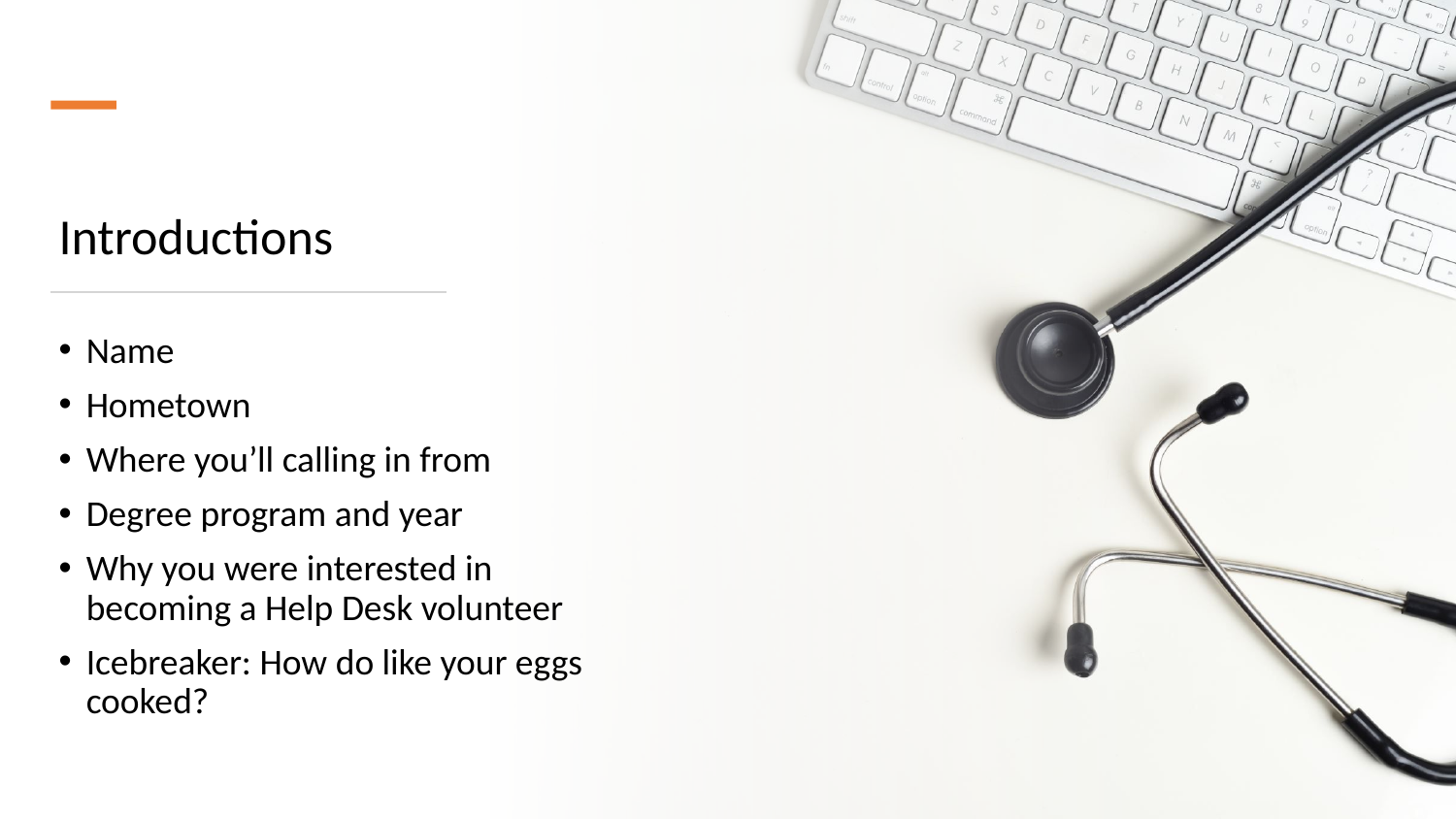

# Introductions
Name
Hometown
Where you’ll calling in from
Degree program and year
Why you were interested in becoming a Help Desk volunteer
Icebreaker: How do like your eggs cooked?

## Slide 5
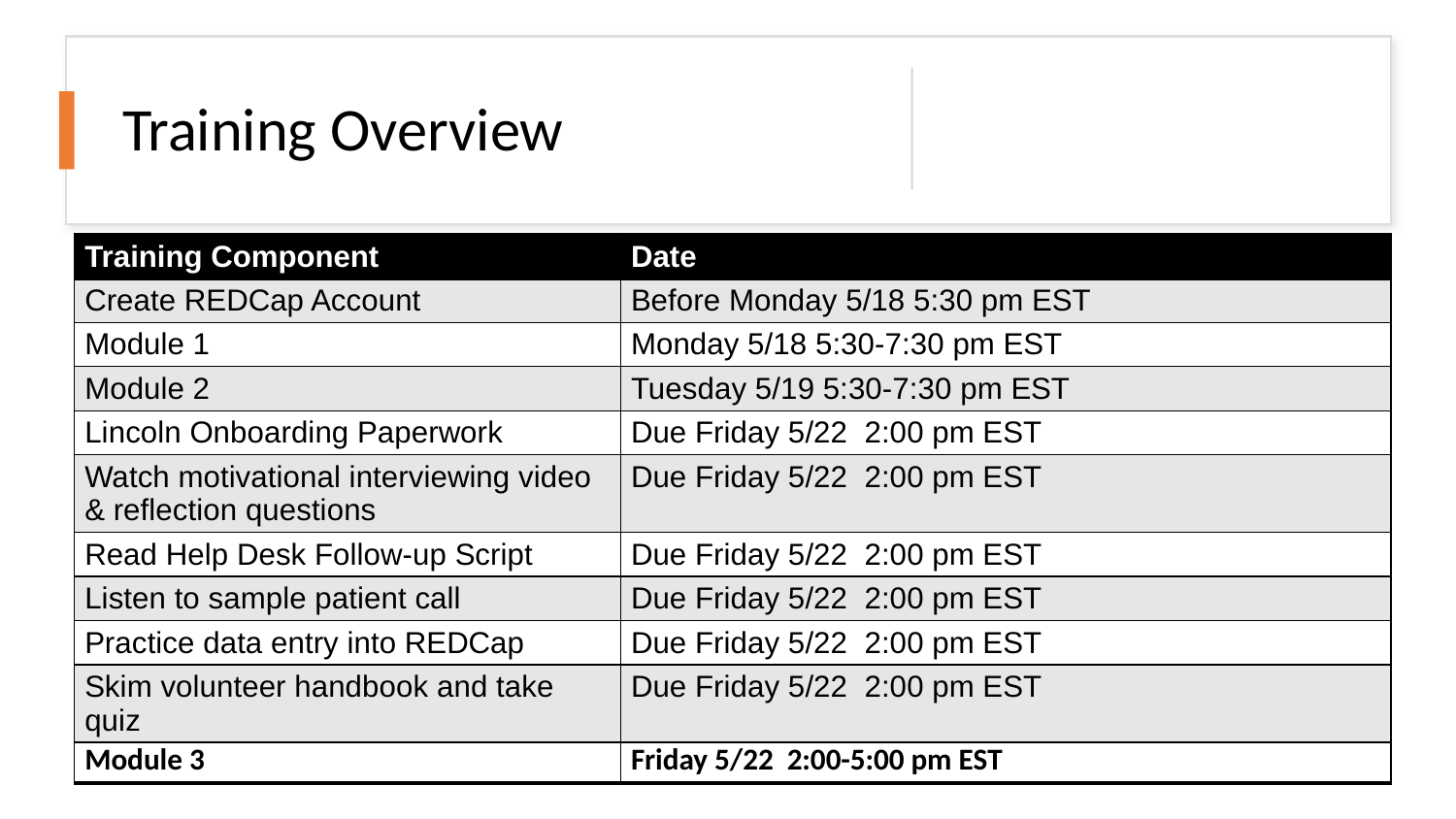

# Training Overview
| Training Component | Date |
| --- | --- |
| Create REDCap Account | Before Monday 5/18 5:30 pm EST |
| Module 1 | Monday 5/18 5:30-7:30 pm EST |
| Module 2 | Tuesday 5/19 5:30-7:30 pm EST |
| Lincoln Onboarding Paperwork | Due Friday 5/22 2:00 pm EST |
| Watch motivational interviewing video & reflection questions | Due Friday 5/22 2:00 pm EST |
| Read Help Desk Follow-up Script | Due Friday 5/22 2:00 pm EST |
| Listen to sample patient call | Due Friday 5/22 2:00 pm EST |
| Practice data entry into REDCap | Due Friday 5/22 2:00 pm EST |
| Skim volunteer handbook and take quiz | Due Friday 5/22 2:00 pm EST |
| Module 3 | Friday 5/22 2:00-5:00 pm EST |

## Slide 6
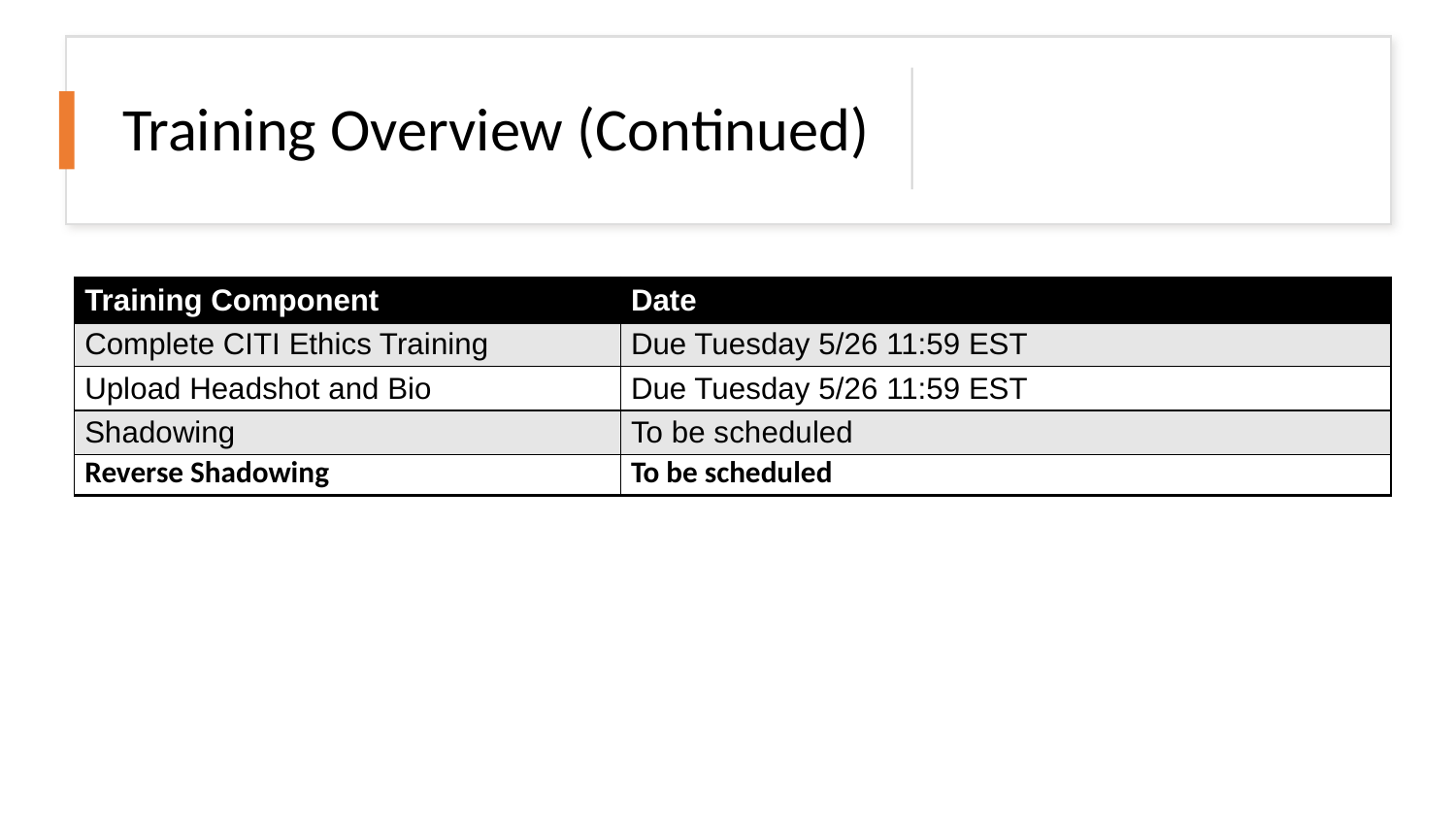

# Training Overview (Continued)
| Training Component | Date |
| --- | --- |
| Complete CITI Ethics Training | Due Tuesday 5/26 11:59 EST |
| Upload Headshot and Bio | Due Tuesday 5/26 11:59 EST |
| Shadowing | To be scheduled |
| Reverse Shadowing | To be scheduled |

## Slide 7
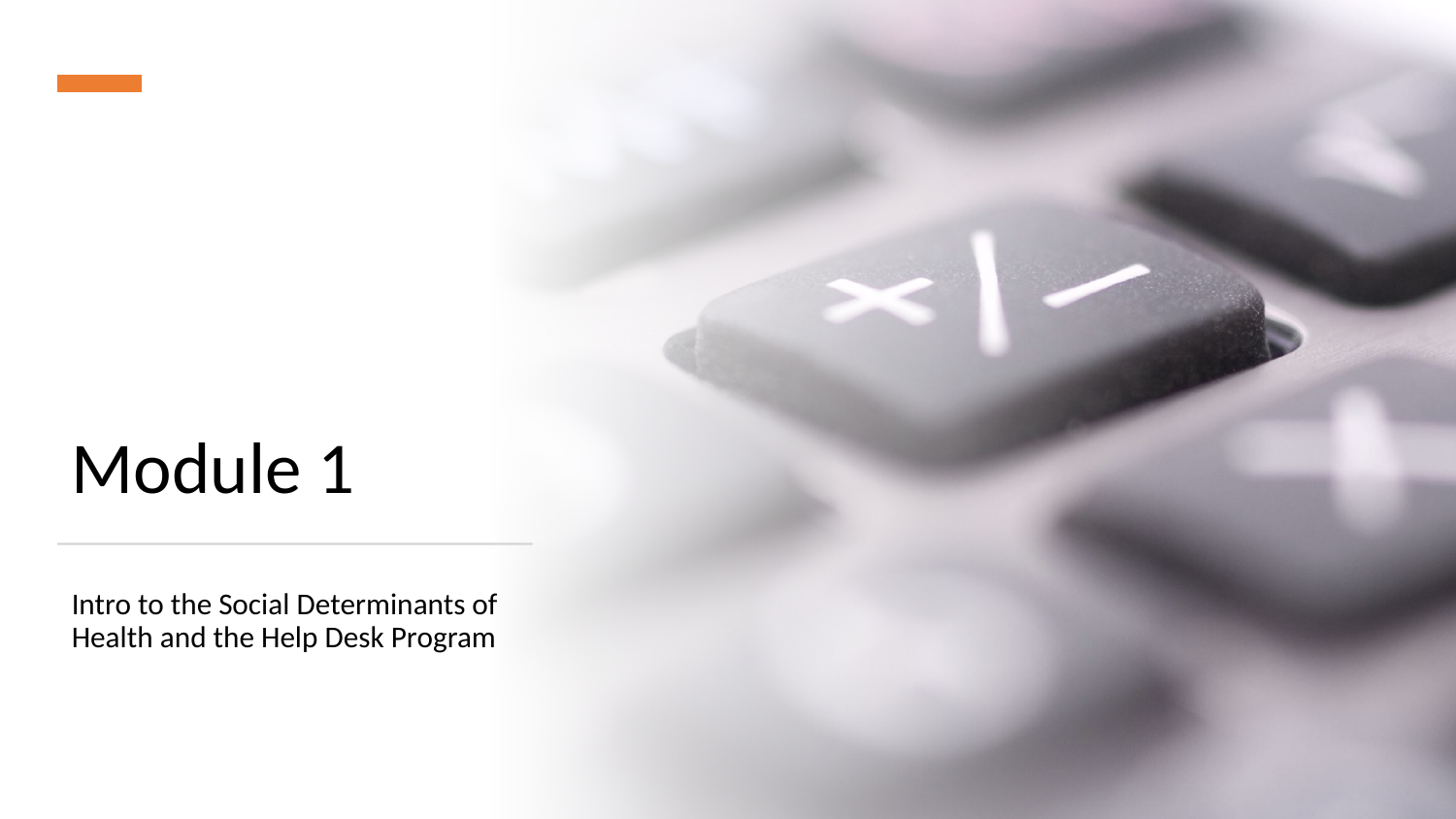

# Module 1
Intro to the Social Determinants of Health and the Help Desk Program

## Slide 8
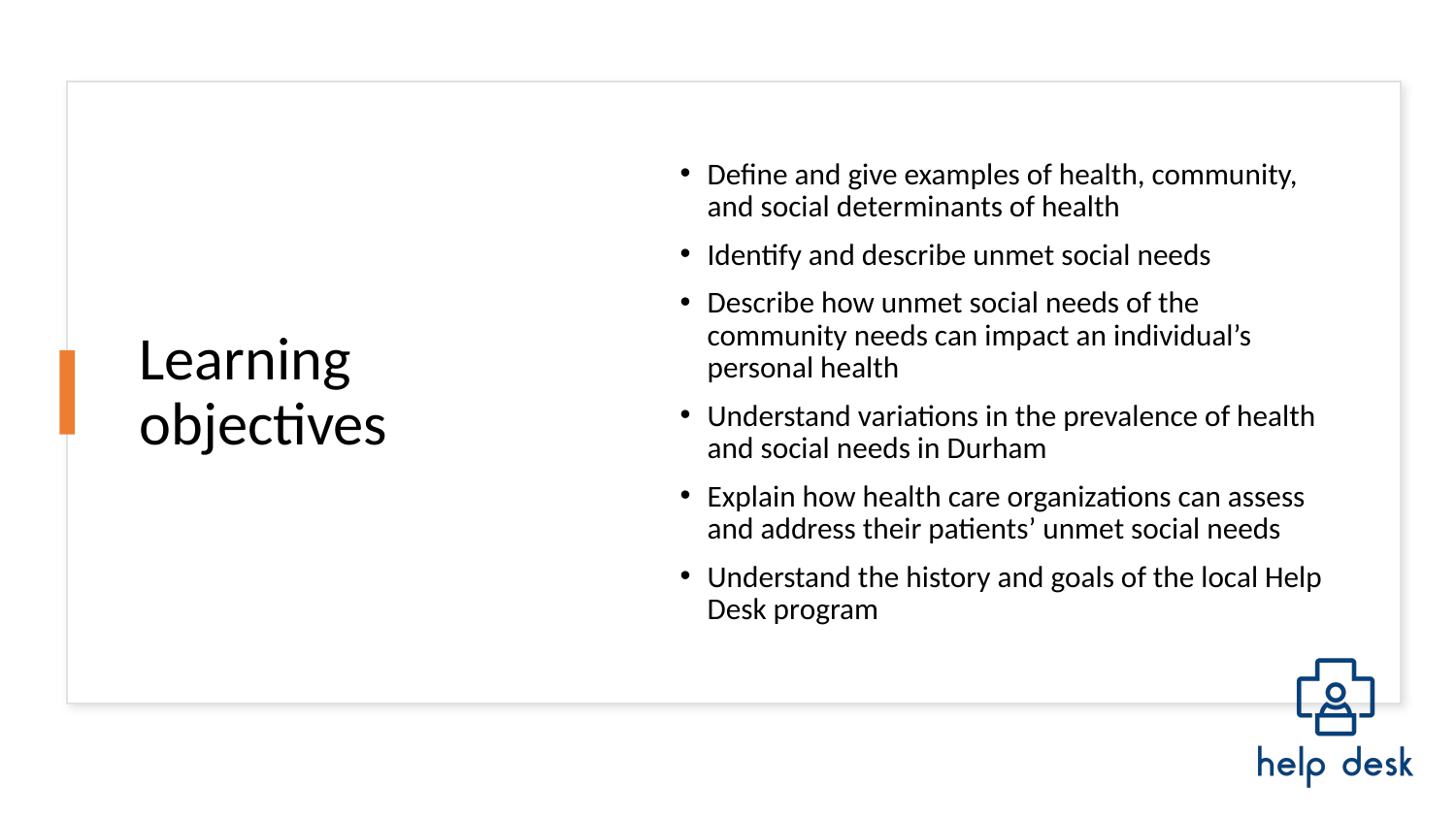

# Learning objectives
Define and give examples of health, community, and social determinants of health
Identify and describe unmet social needs
Describe how unmet social needs of the community needs can impact an individual’s personal health
Understand variations in the prevalence of health and social needs in Durham
Explain how health care organizations can assess and address their patients’ unmet social needs
Understand the history and goals of the local Help Desk program

## Slide 9
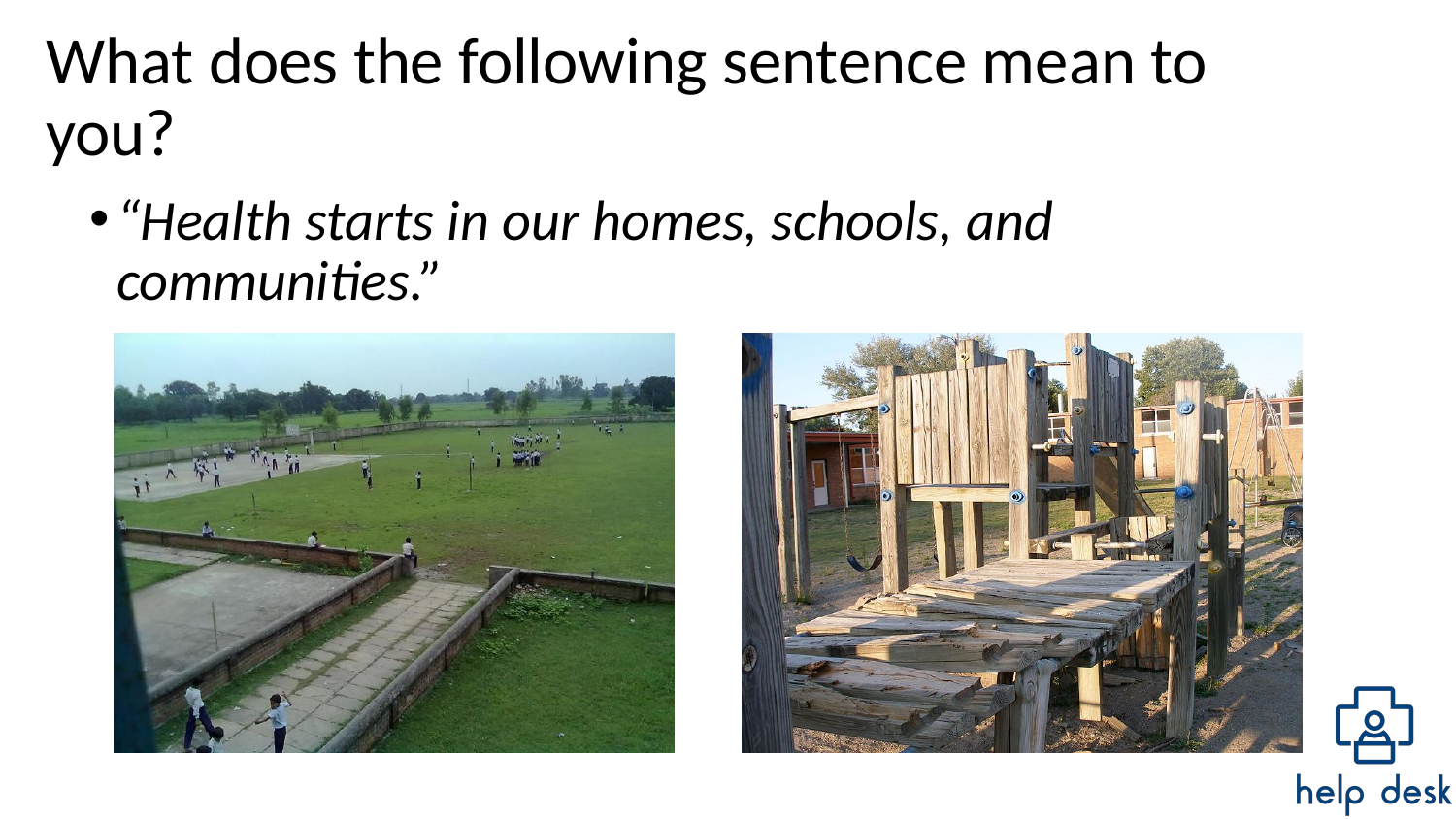

# What does the following sentence mean to you?
“Health starts in our homes, schools, and communities.”

## Slide 10
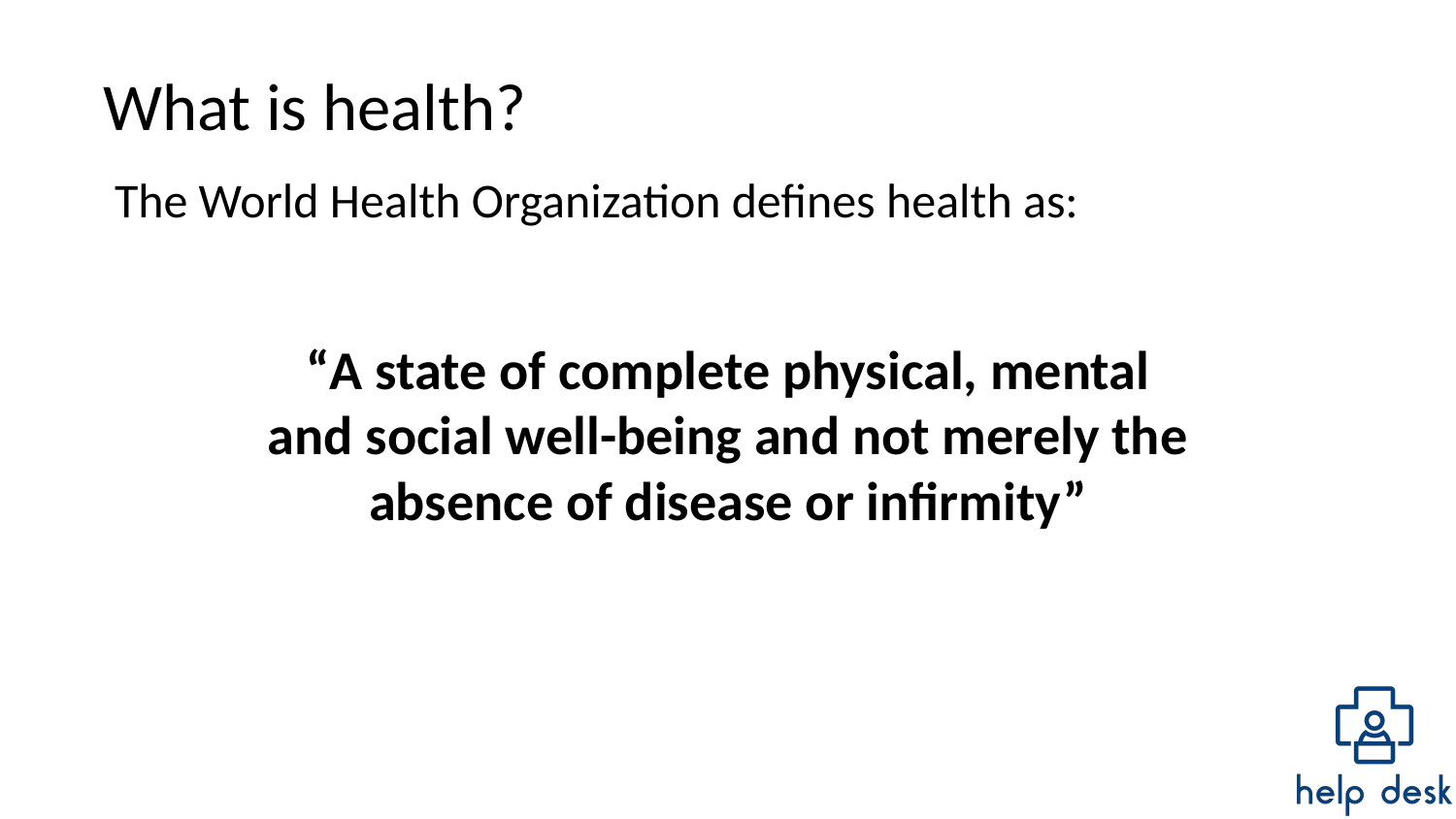

# What is health?
The World Health Organization defines health as:
“A state of complete physical, mental and social well-being and not merely the absence of disease or infirmity”

## Slide 11
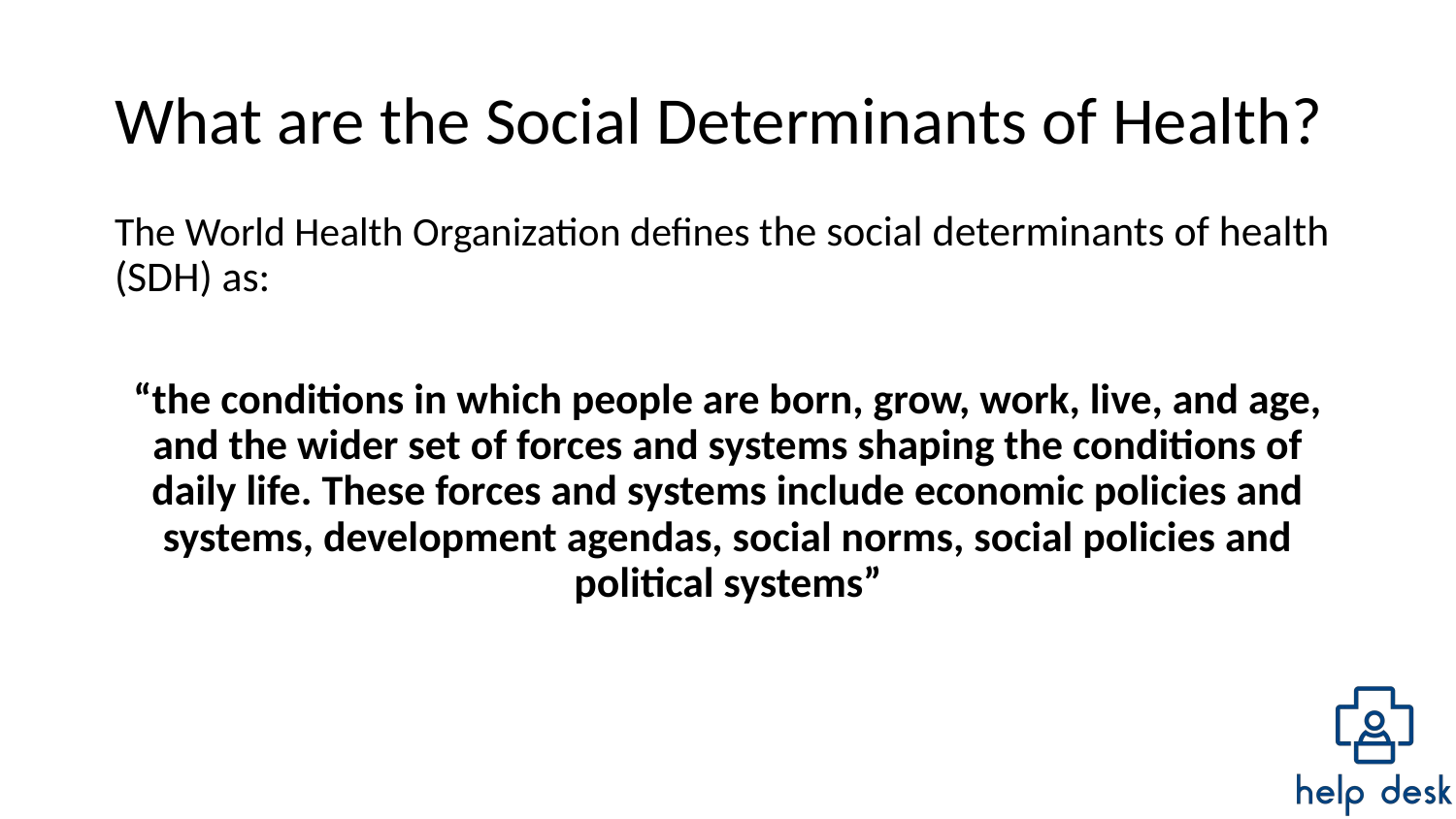

# What are the Social Determinants of Health?
The World Health Organization defines the social determinants of health (SDH) as:
“the conditions in which people are born, grow, work, live, and age, and the wider set of forces and systems shaping the conditions of daily life. These forces and systems include economic policies and systems, development agendas, social norms, social policies and political systems”

## Slide 12
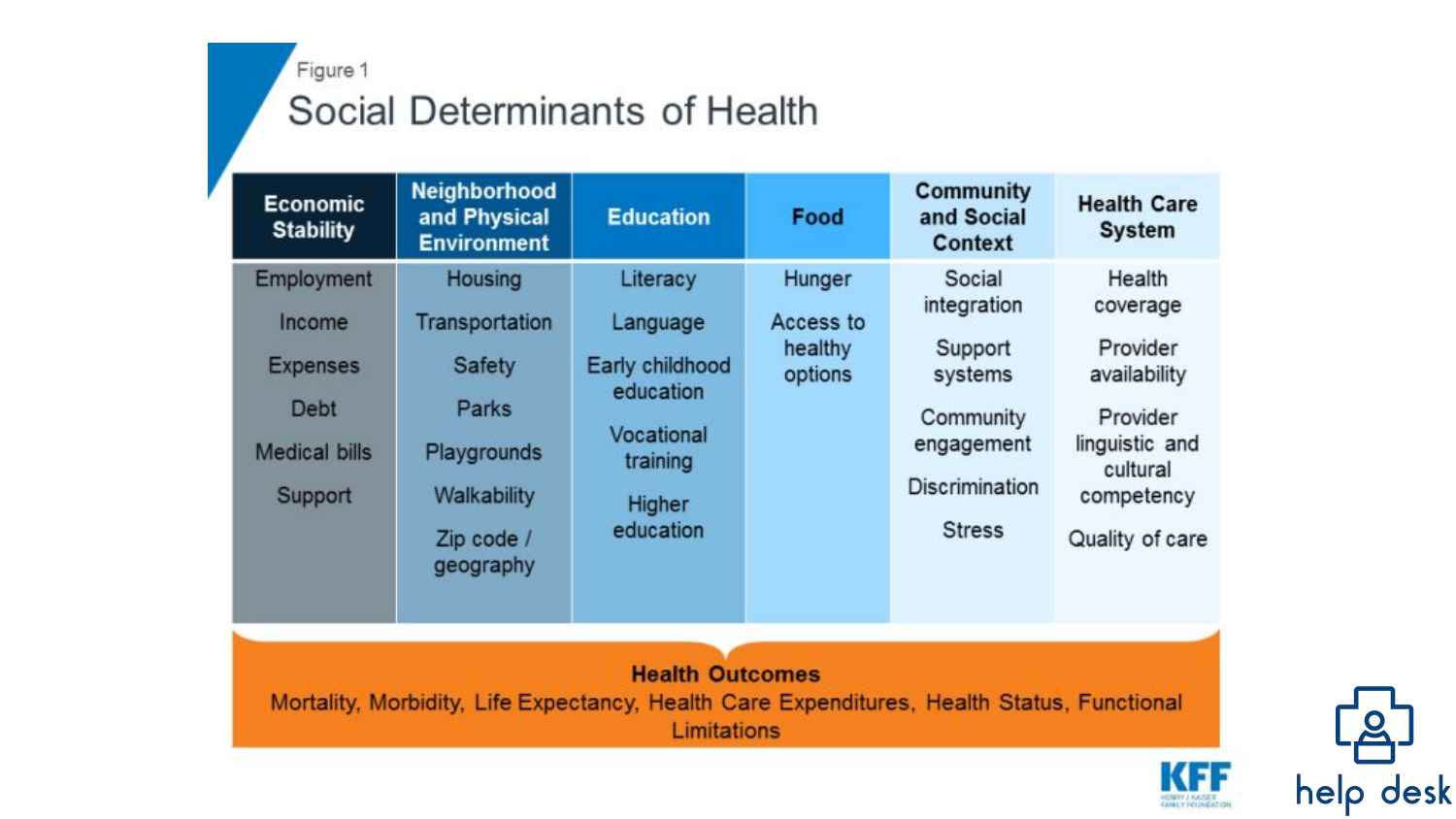

#

## Slide 13
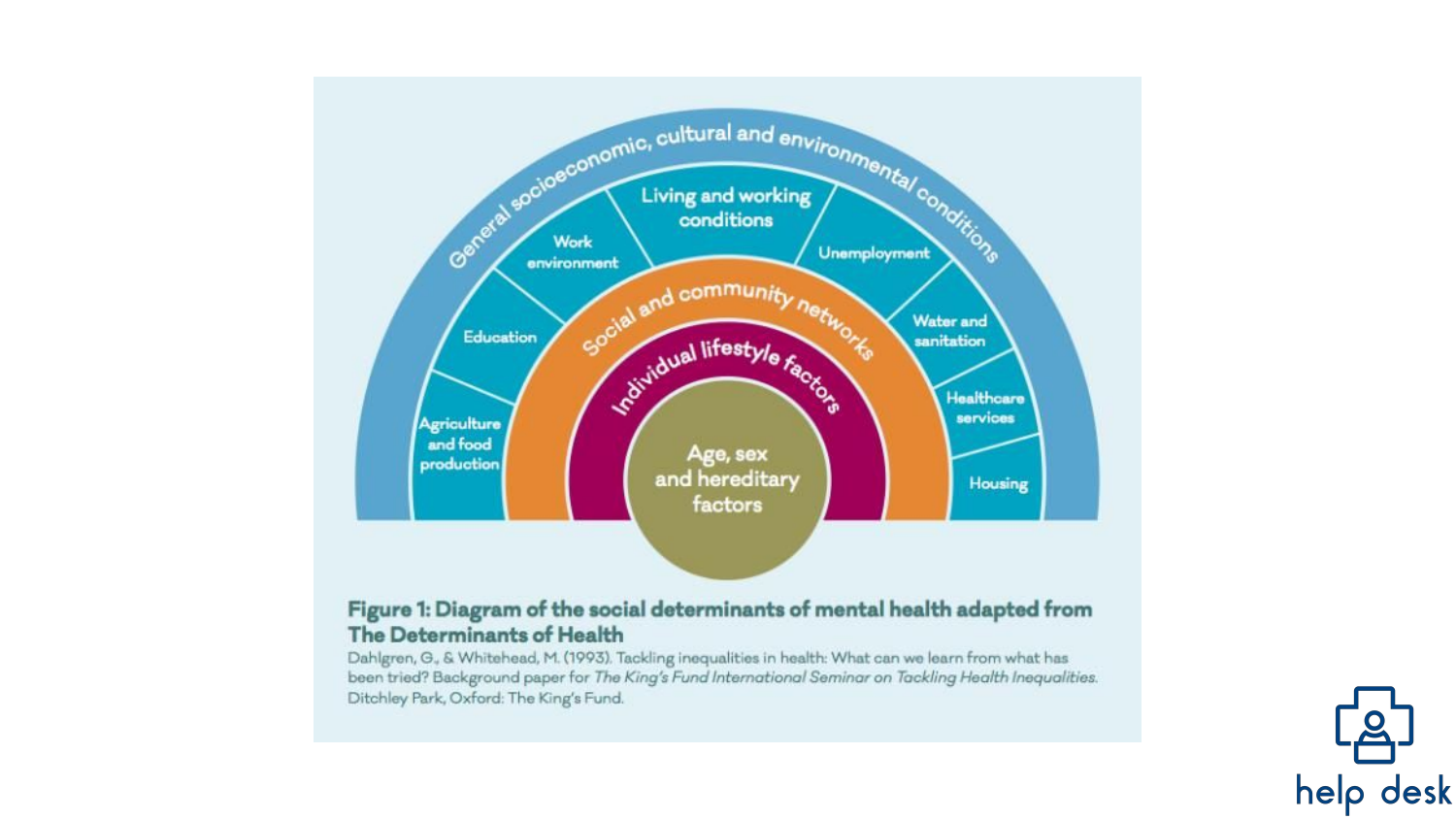

#

## Slide 14
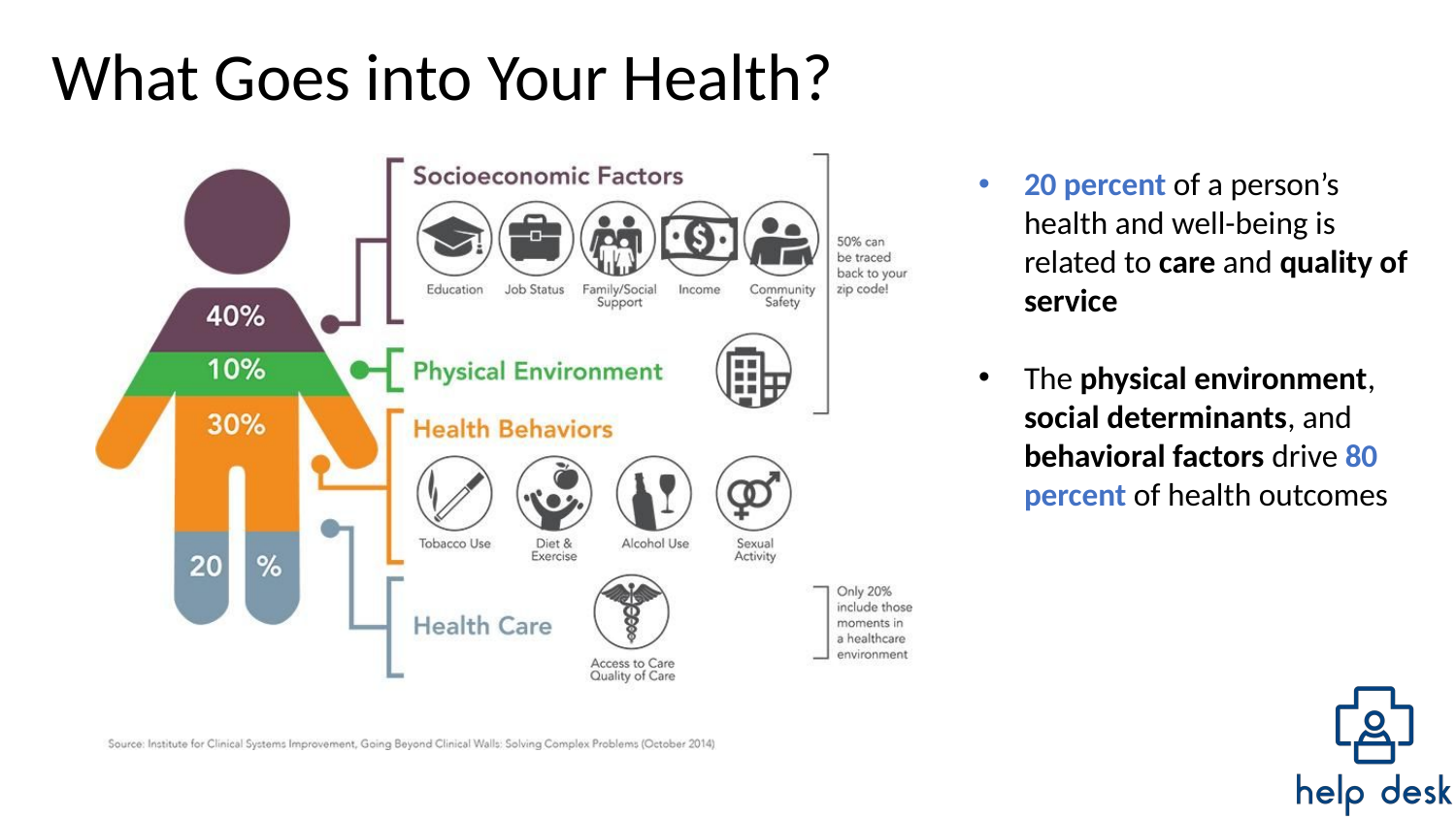

# What Goes into Your Health?
20 percent of a person’s health and well-being is related to care and quality of service
The physical environment, social determinants, and behavioral factors drive 80 percent of health outcomes

## Slide 15
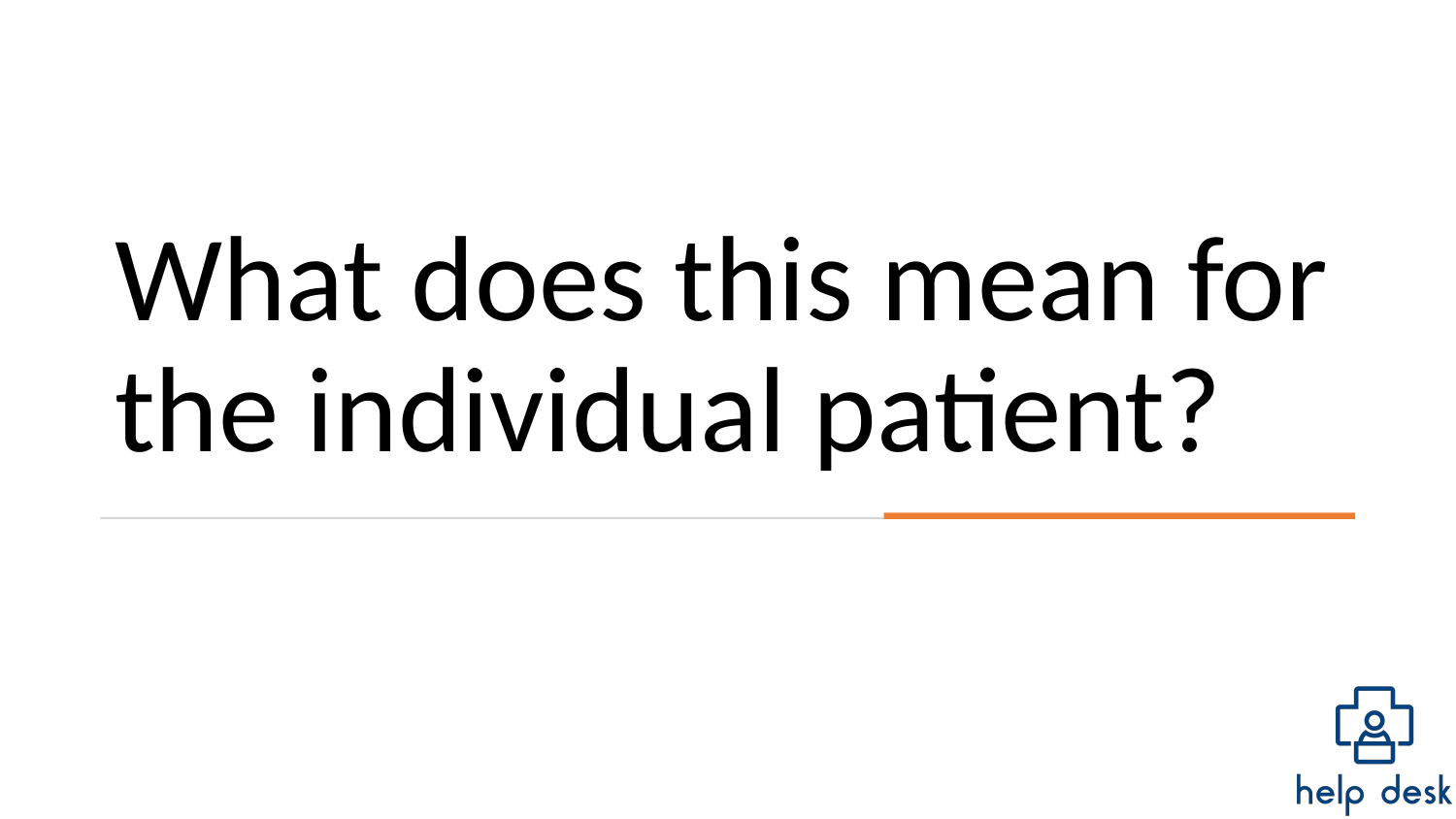

# What does this mean for the individual patient?

## Slide 16
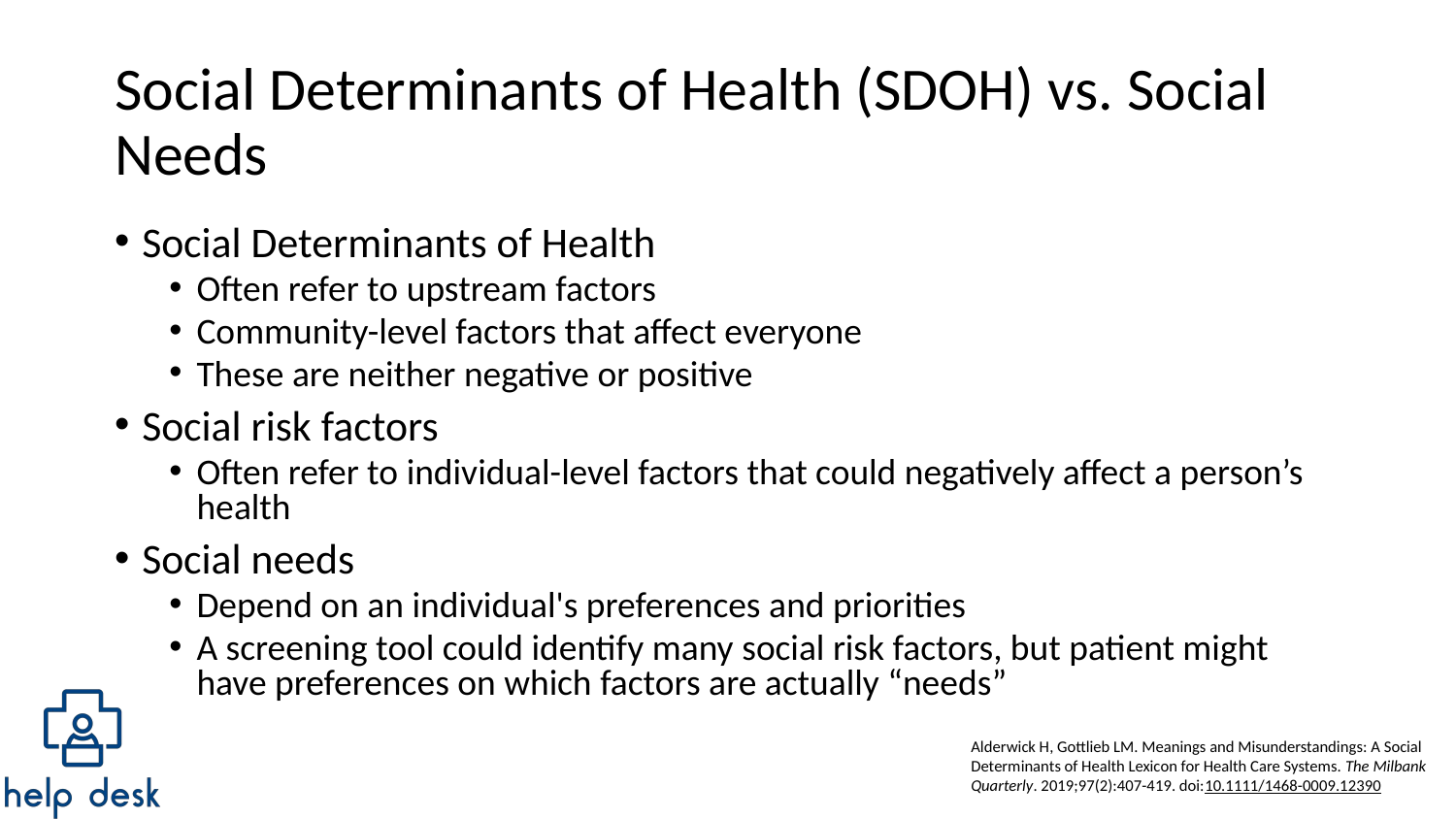

# Social Determinants of Health (SDOH) vs. Social Needs
Social Determinants of Health
Often refer to upstream factors
Community-level factors that affect everyone
These are neither negative or positive
Social risk factors
Often refer to individual-level factors that could negatively affect a person’s health
Social needs
Depend on an individual's preferences and priorities
A screening tool could identify many social risk factors, but patient might have preferences on which factors are actually “needs”
Alderwick H, Gottlieb LM. Meanings and Misunderstandings: A Social Determinants of Health Lexicon for Health Care Systems. The Milbank Quarterly. 2019;97(2):407-419. doi:10.1111/1468-0009.12390

## Slide 17
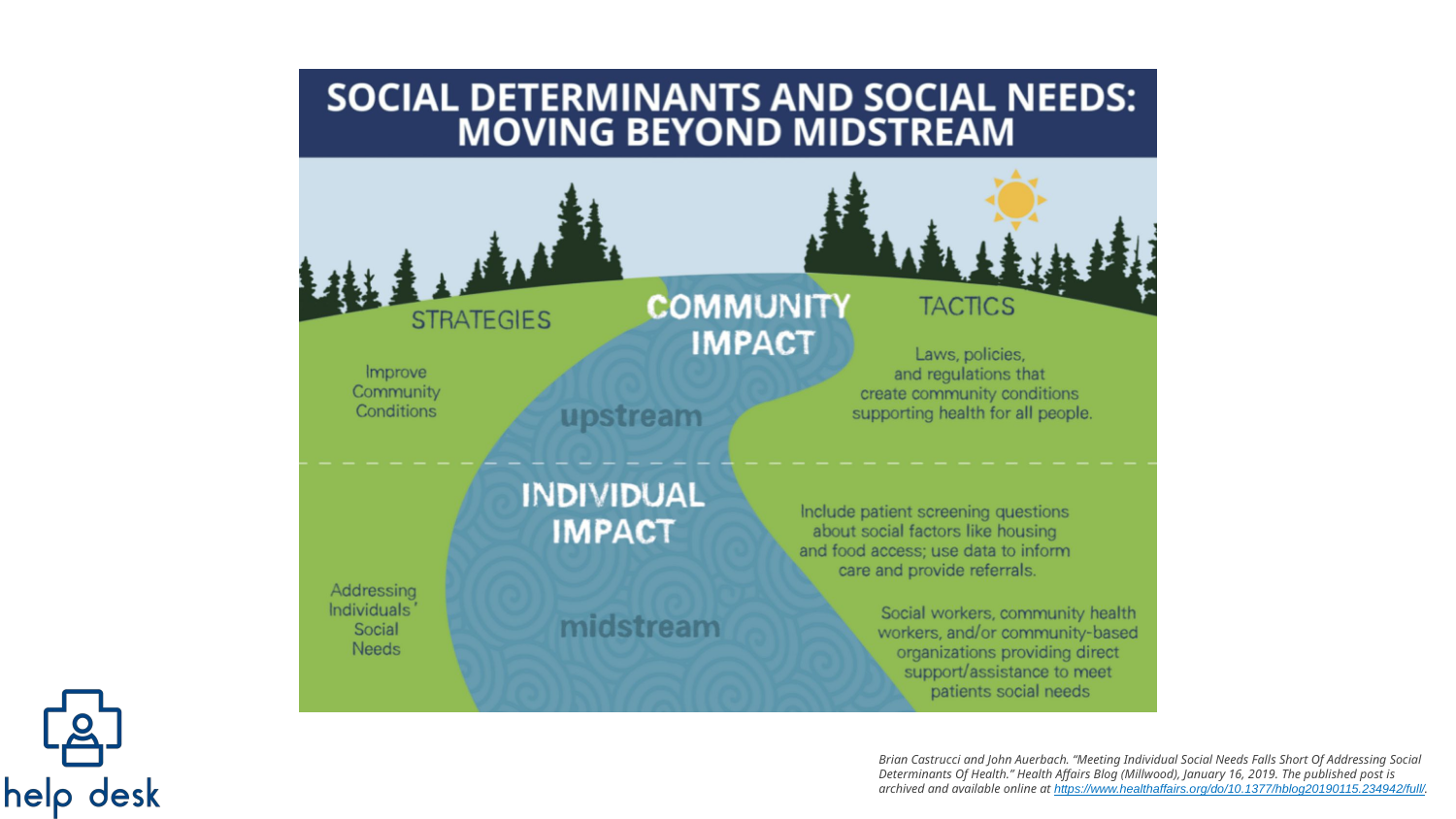

Brian Castrucci and John Auerbach. “Meeting Individual Social Needs Falls Short Of Addressing Social Determinants Of Health.” Health Affairs Blog (Millwood), January 16, 2019. The published post is archived and available online at https://www.healthaffairs.org/do/10.1377/hblog20190115.234942/full/.

## Slide 18
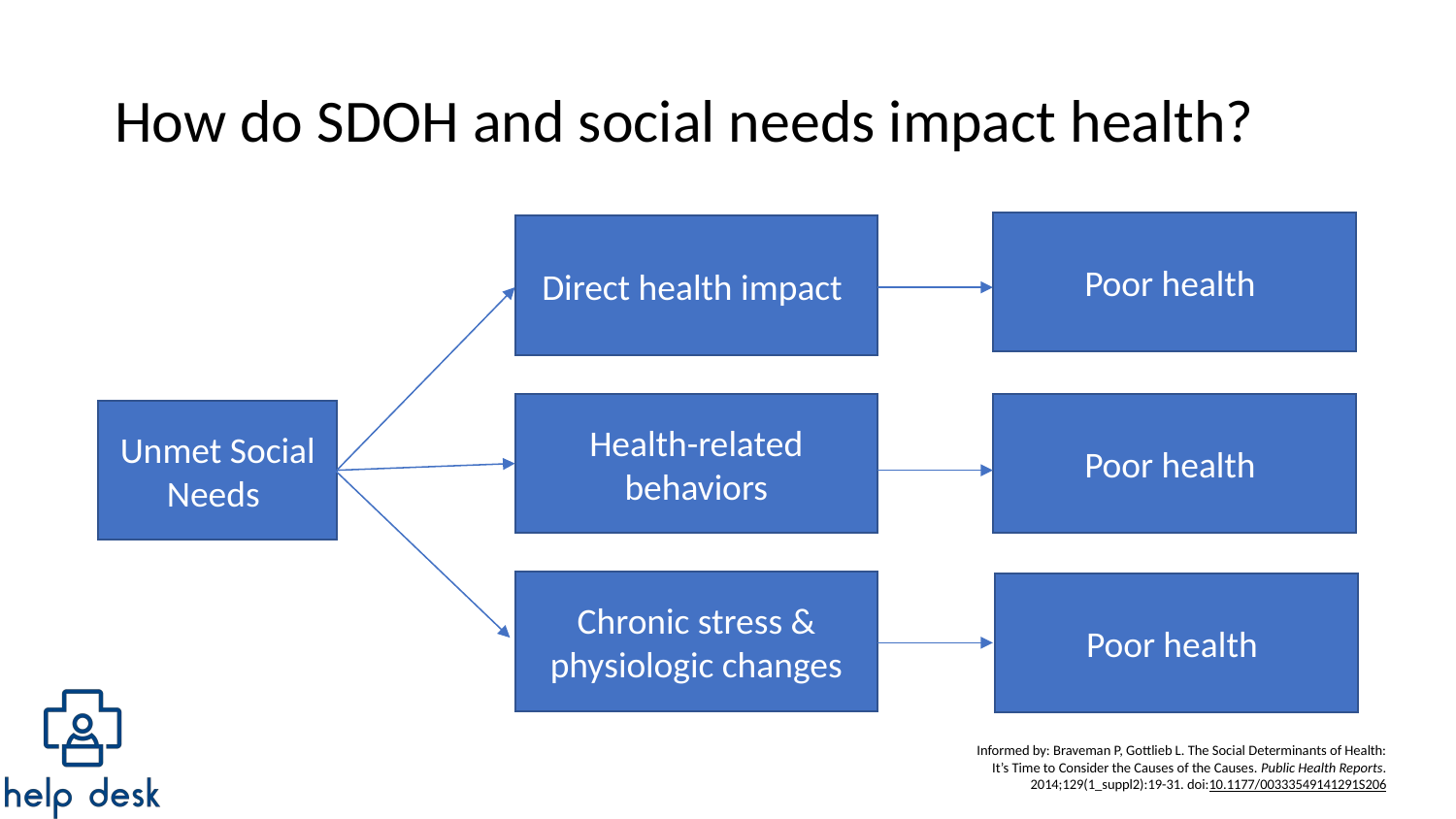

# How do SDOH and social needs impact health?
Poor health
Direct health impact
Health-related behaviors
Poor health
Unmet Social Needs
Chronic stress & physiologic changes
Poor health
Informed by: Braveman P, Gottlieb L. The Social Determinants of Health: It’s Time to Consider the Causes of the Causes. Public Health Reports. 2014;129(1_suppl2):19-31. doi:10.1177/00333549141291S206

## Slide 19
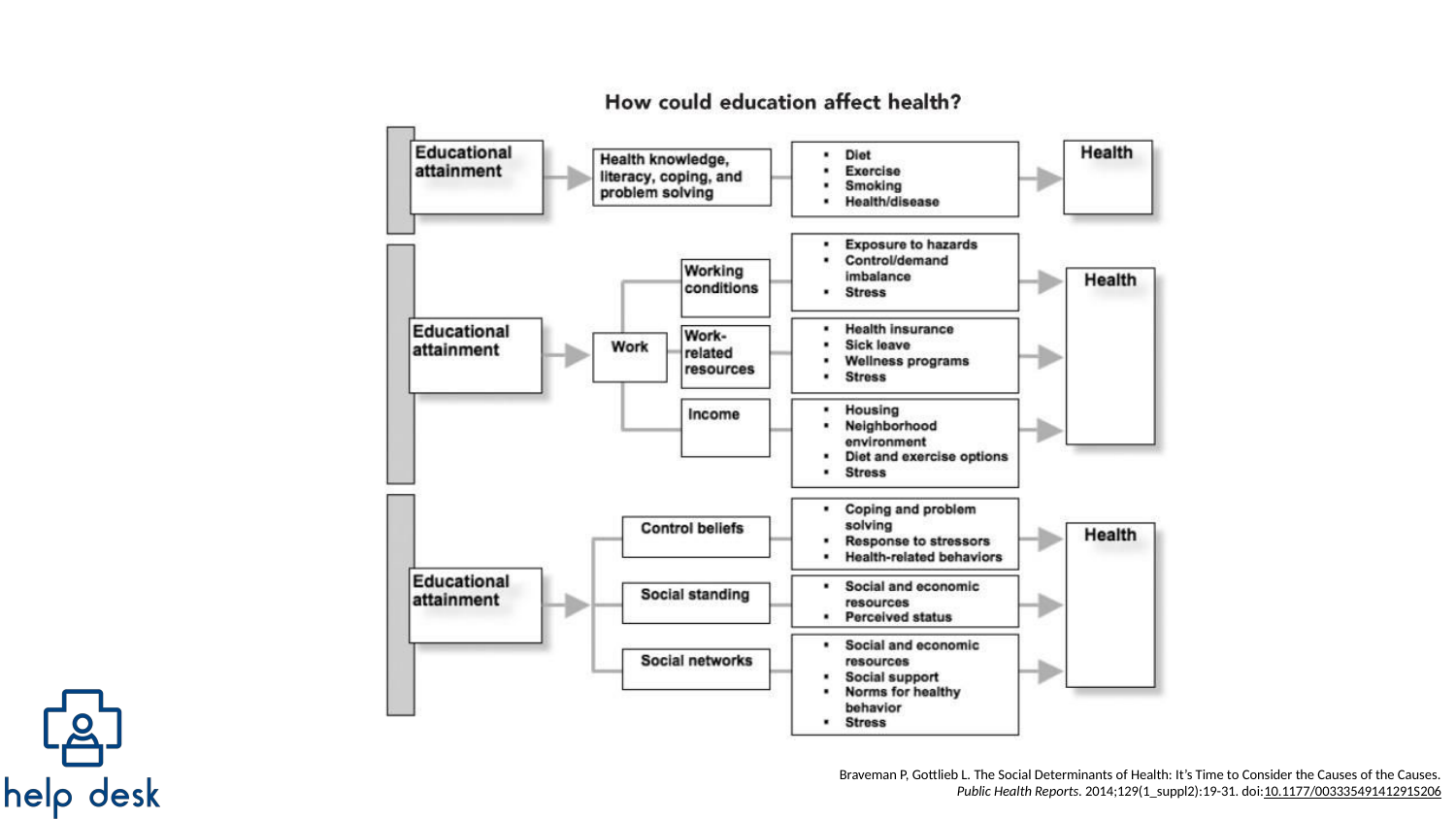

#
Braveman P, Gottlieb L. The Social Determinants of Health: It’s Time to Consider the Causes of the Causes. Public Health Reports. 2014;129(1_suppl2):19-31. doi:10.1177/00333549141291S206

## Slide 20
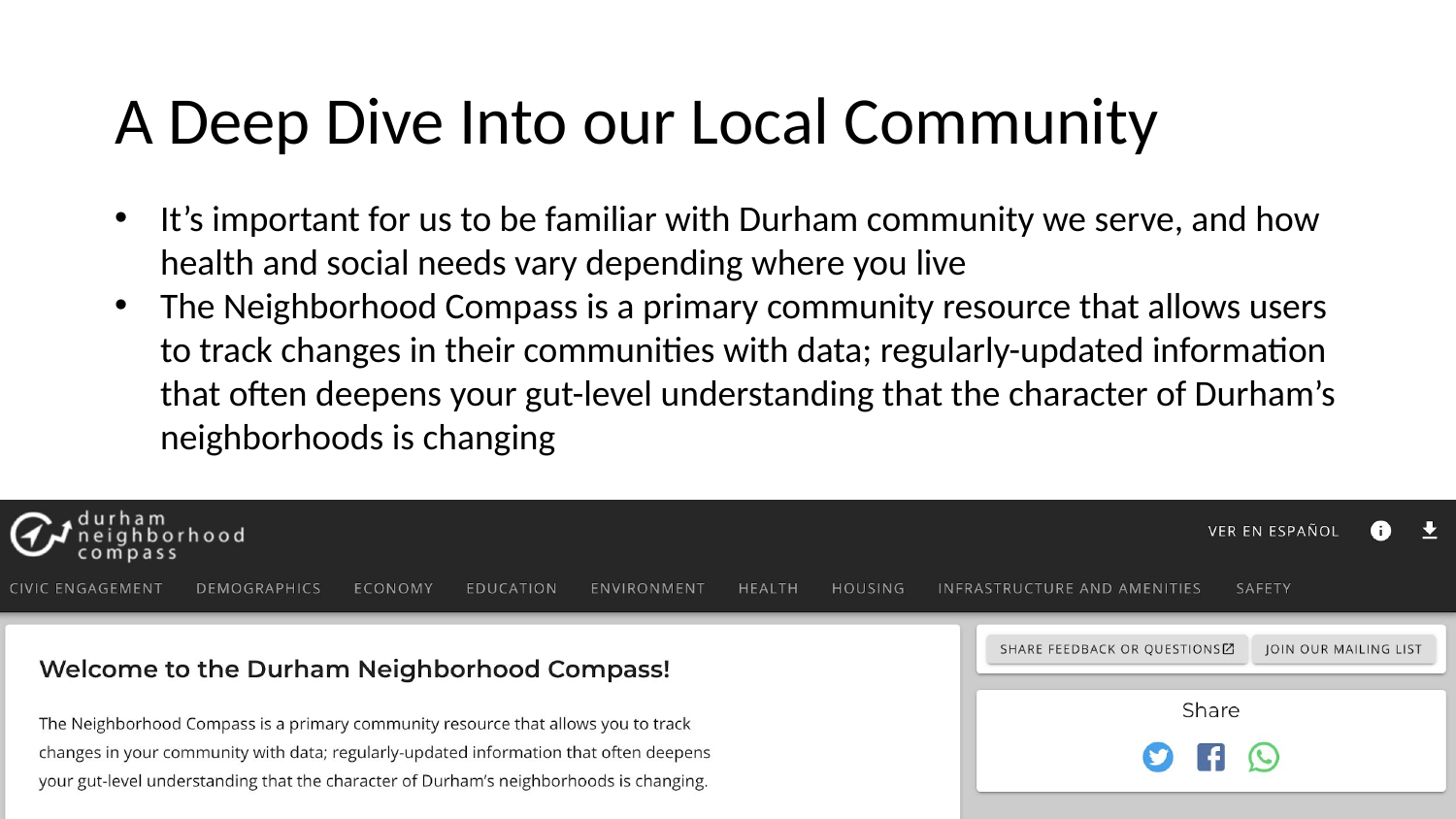

# A Deep Dive Into our Local Community
It’s important for us to be familiar with Durham community we serve, and how health and social needs vary depending where you live
The Neighborhood Compass is a primary community resource that allows users to track changes in their communities with data; regularly-updated information that often deepens your gut-level understanding that the character of Durham’s neighborhoods is changing

## Slide 21
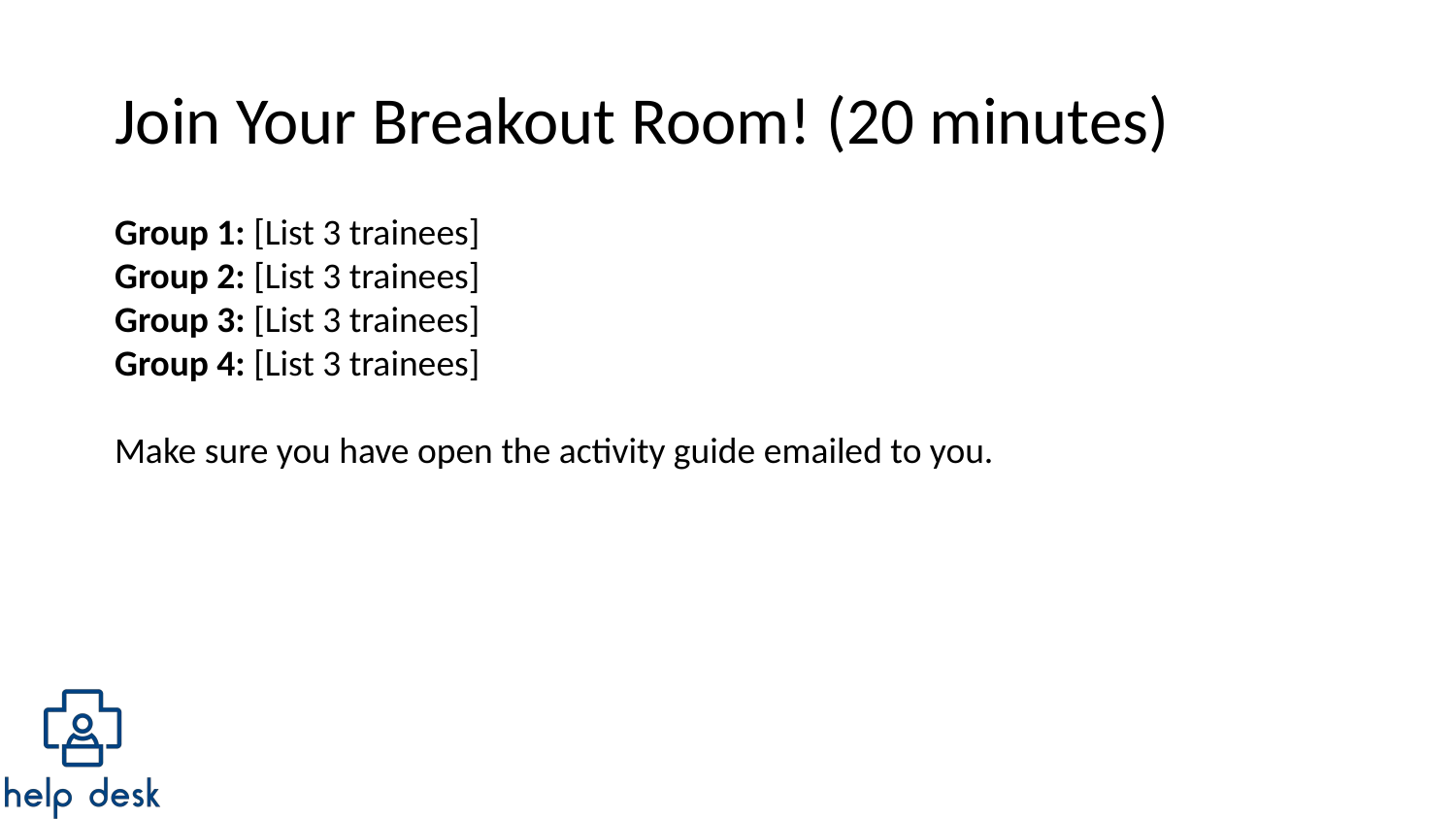

# Join Your Breakout Room! (20 minutes)
Group 1: [List 3 trainees]
Group 2: [List 3 trainees]
Group 3: [List 3 trainees]
Group 4: [List 3 trainees]
Make sure you have open the activity guide emailed to you.

## Slide 22
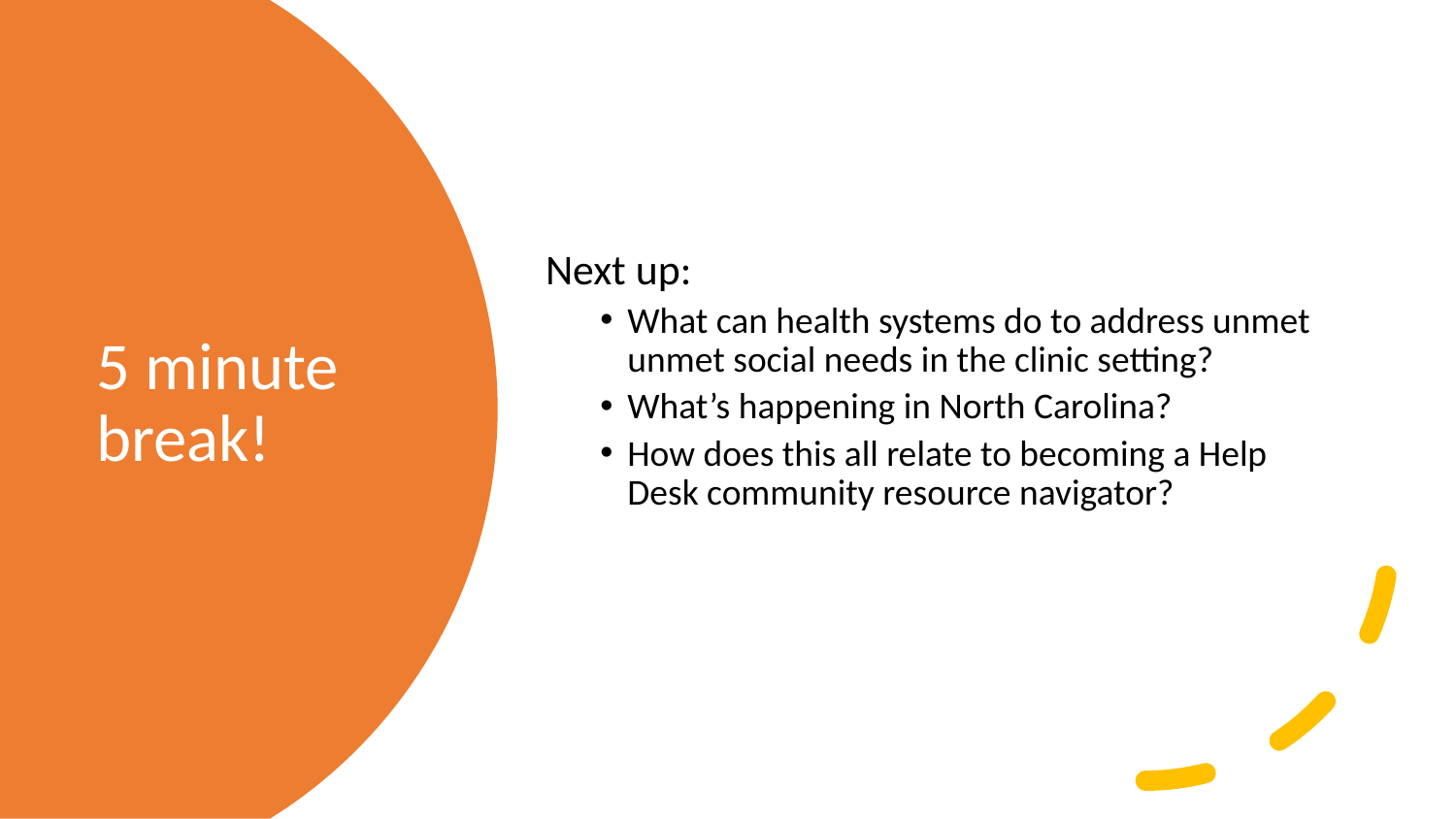

Next up:
What can health systems do to address unmet unmet social needs in the clinic setting?
What’s happening in North Carolina?
How does this all relate to becoming a Help Desk community resource navigator?
# 5 minute break!

## Slide 23
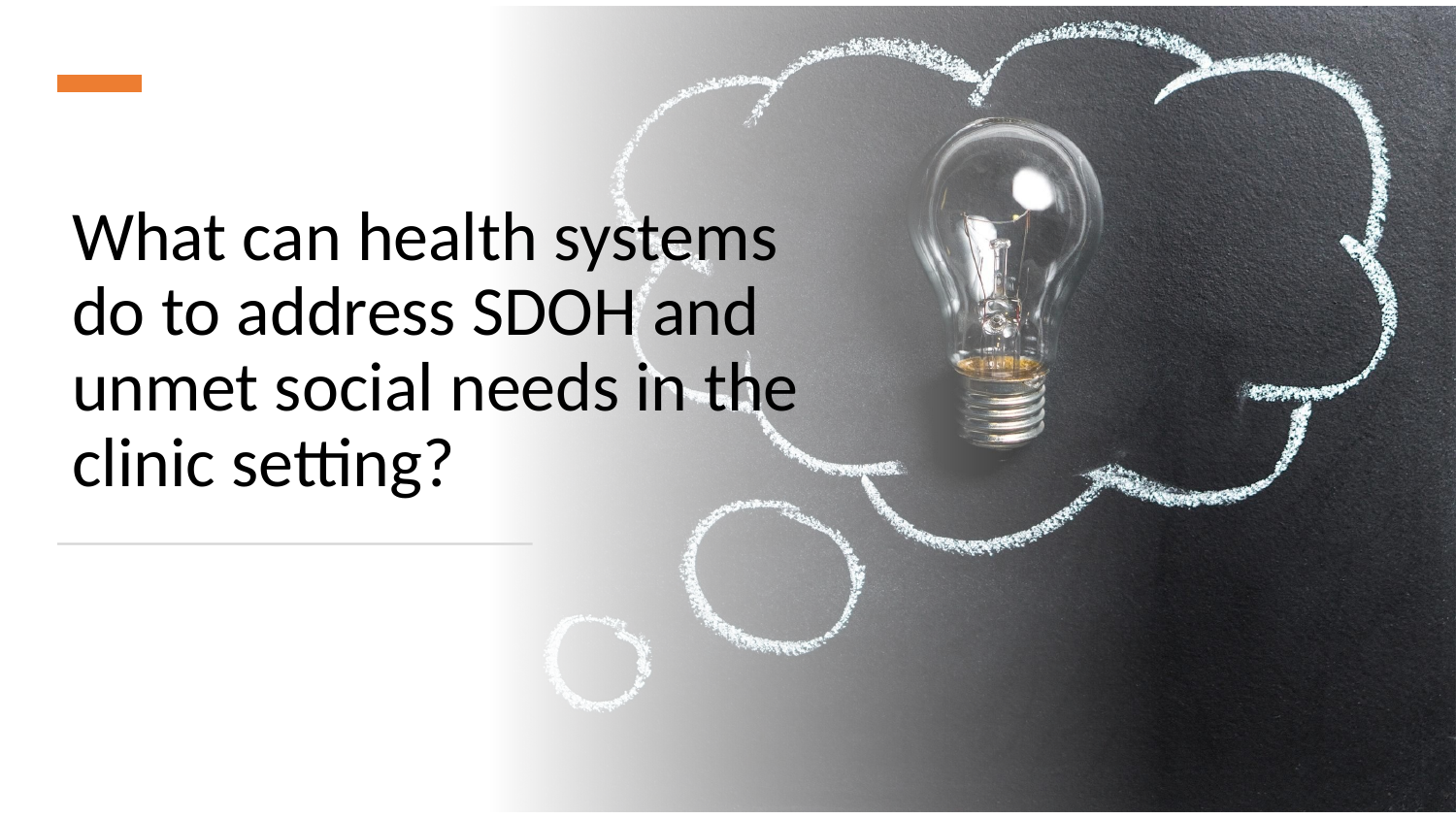

# What can health systems do to address SDOH and unmet social needs in the clinic setting?

## Slide 24
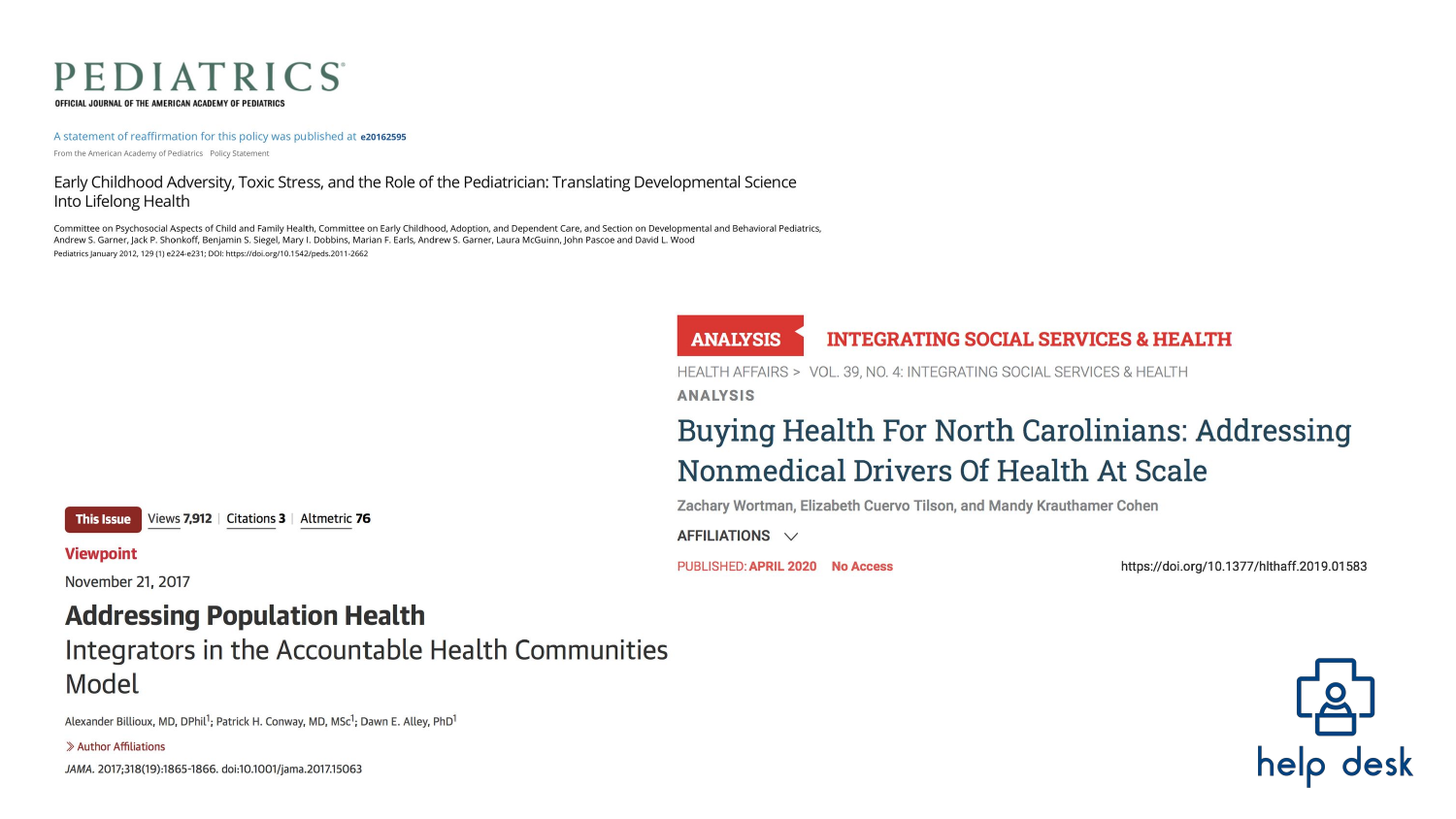

## Slide 25
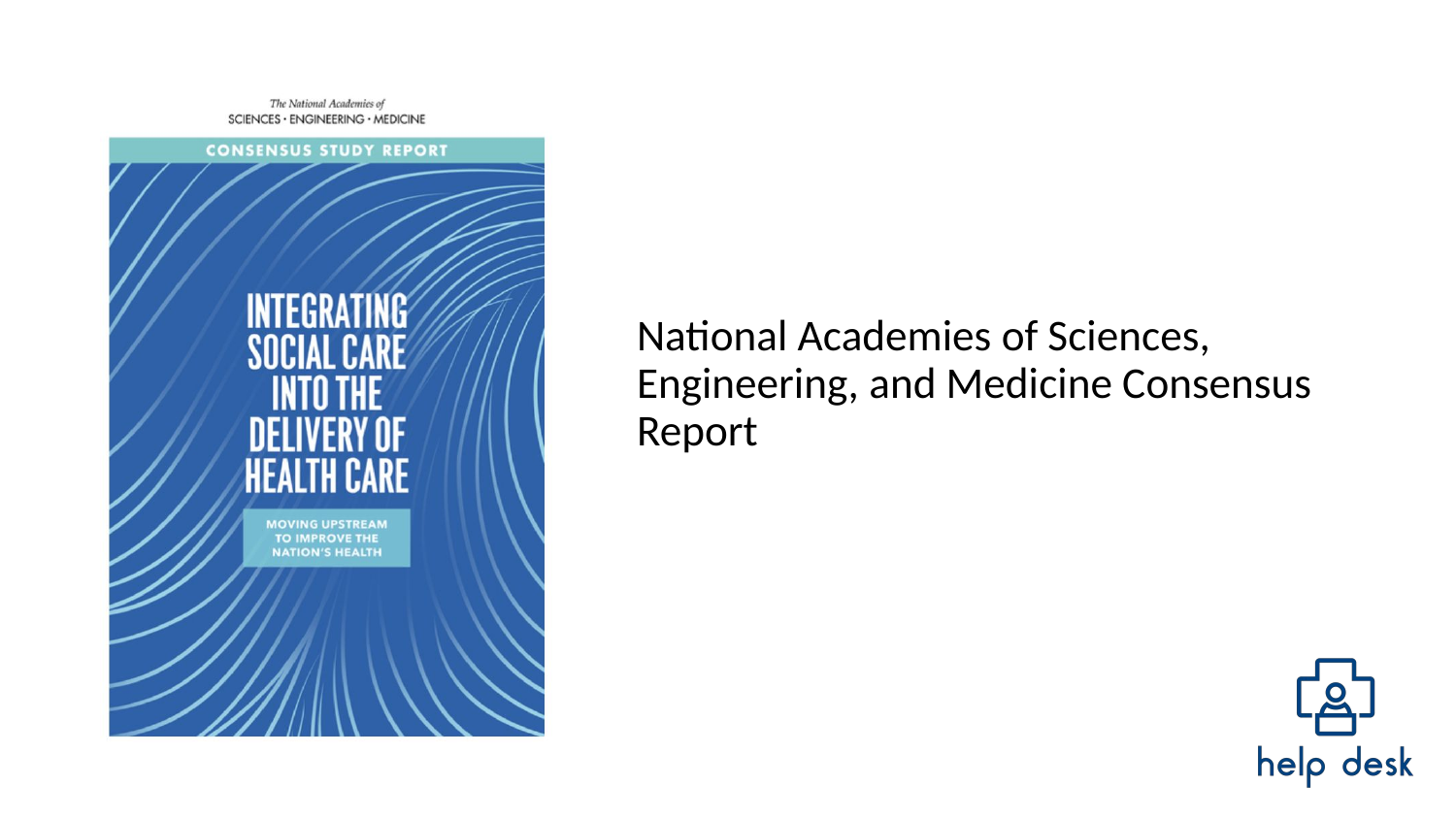

# National Academies of Sciences, Engineering, and Medicine Consensus Report

## Slide 26
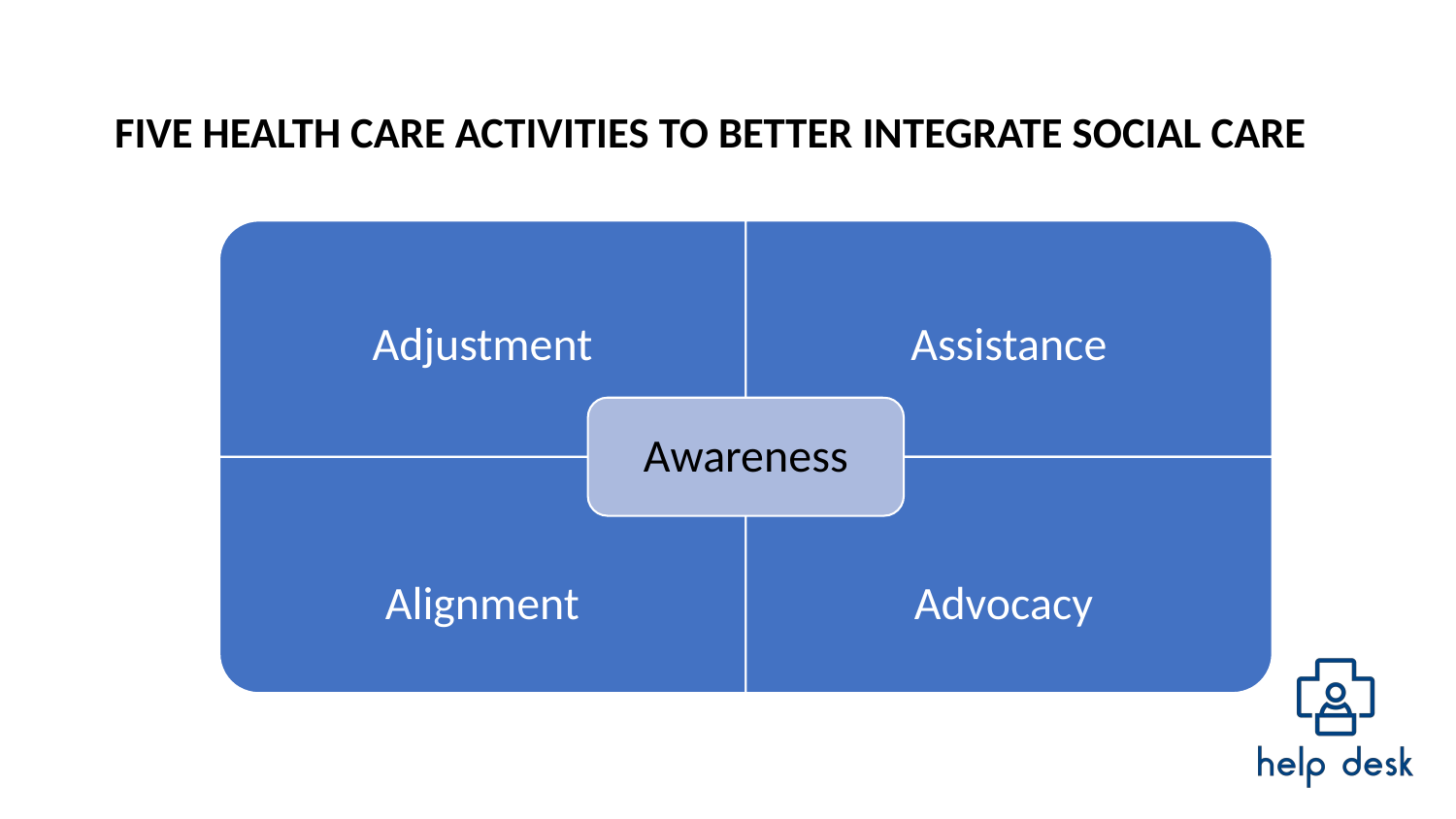

# FIVE HEALTH CARE ACTIVITIES TO BETTER INTEGRATE SOCIAL CARE
Adjustment
Assistance
Awareness
Alignment
Advocacy

## Slide 27
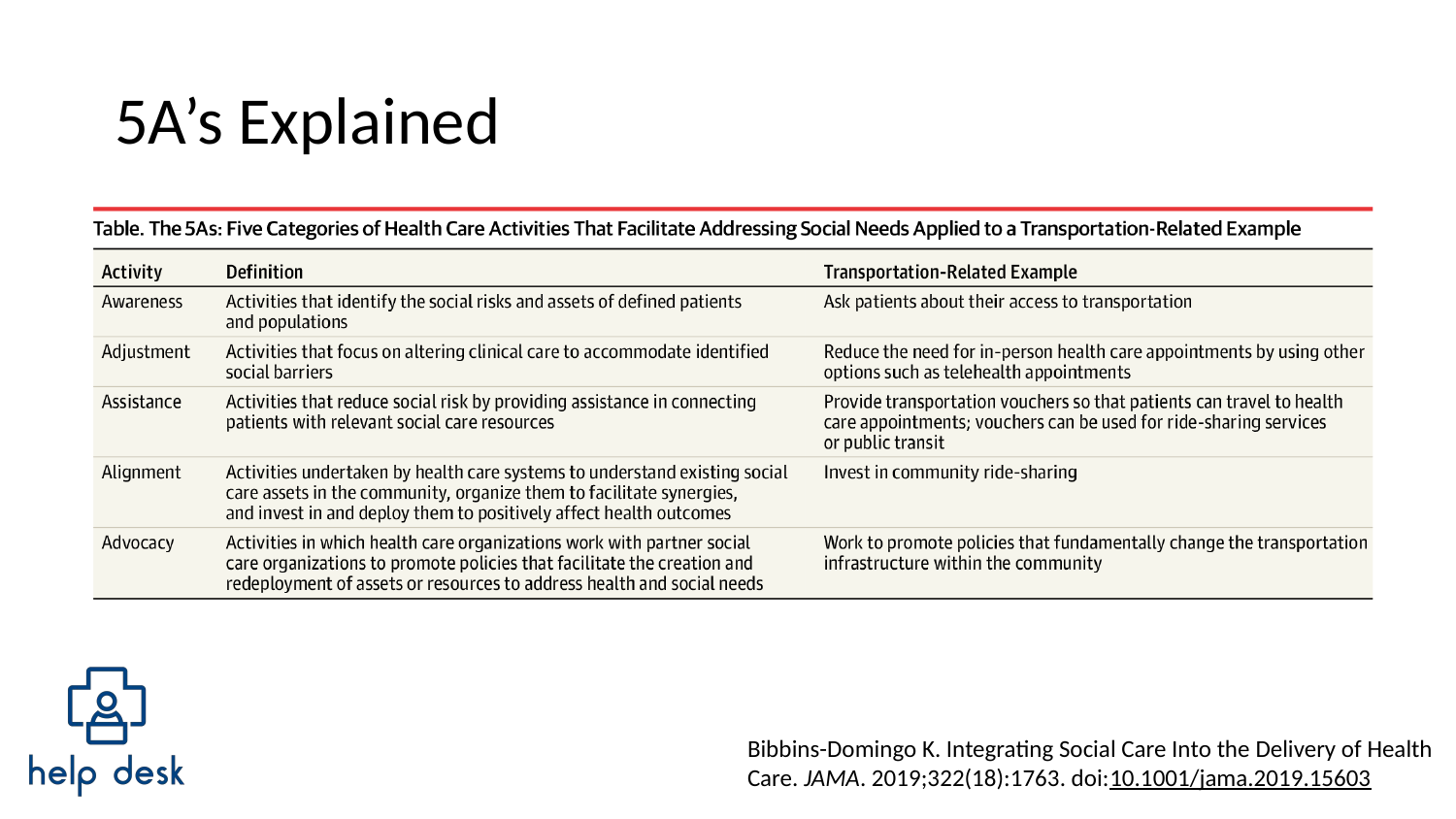

# 5A’s Explained
Bibbins-Domingo K. Integrating Social Care Into the Delivery of Health Care. JAMA. 2019;322(18):1763. doi:10.1001/jama.2019.15603

## Slide 28
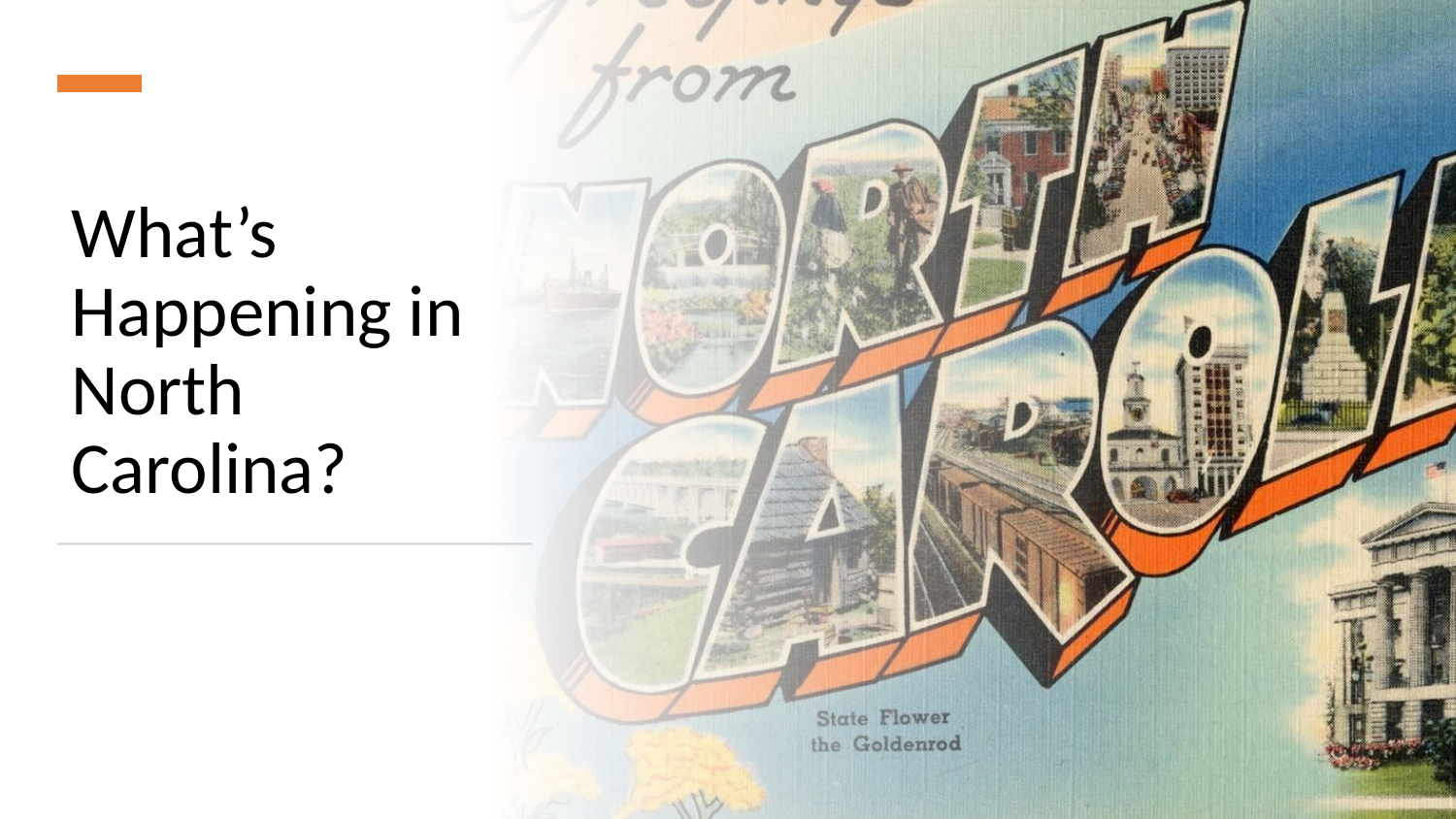

# What’s Happening in North Carolina?

## Slide 29
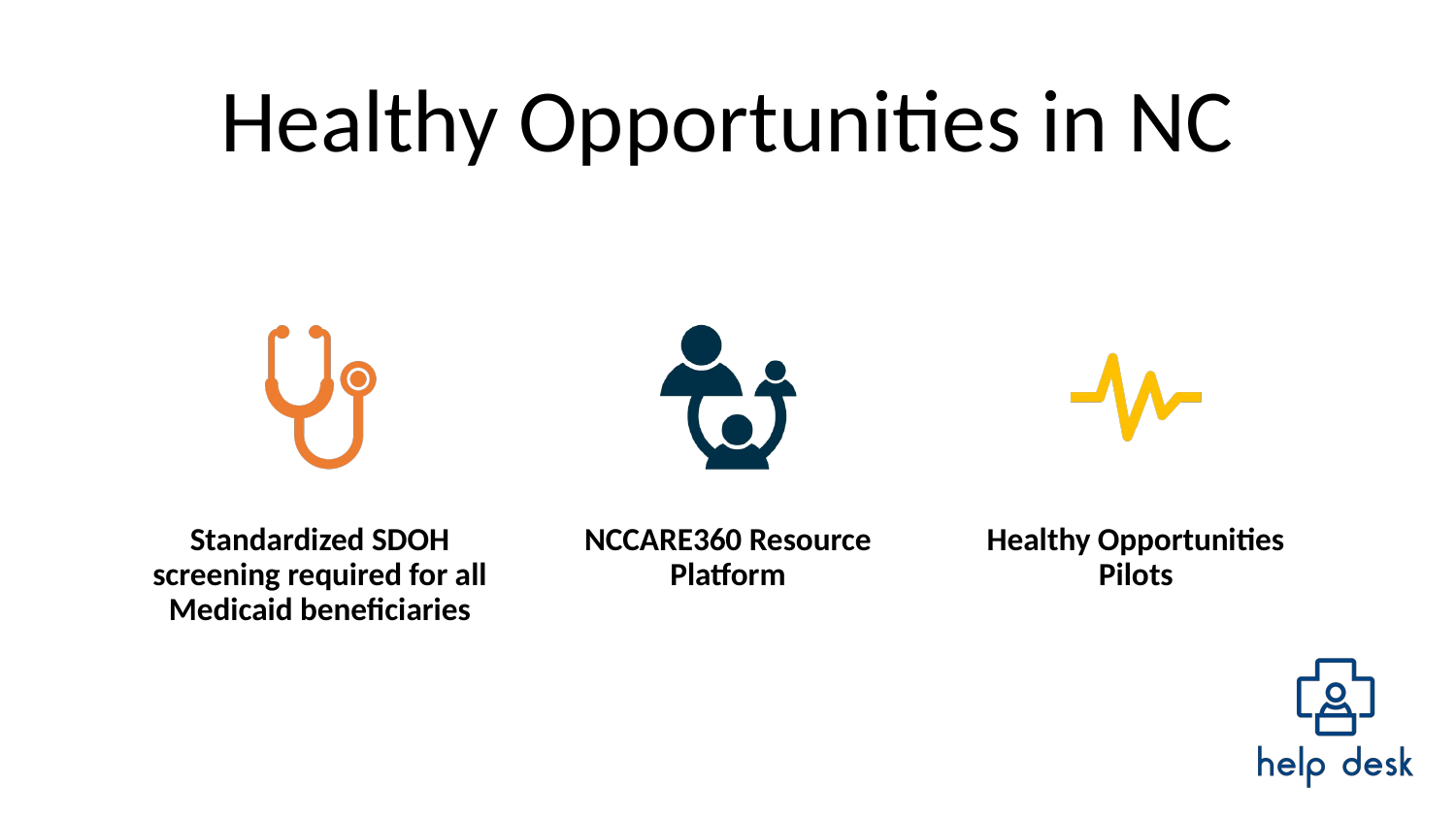

Healthy Opportunities in NC
Standardized SDOH screening required for all Medicaid beneficiaries
NCCARE360 Resource Platform
Healthy Opportunities Pilots

## Slide 30
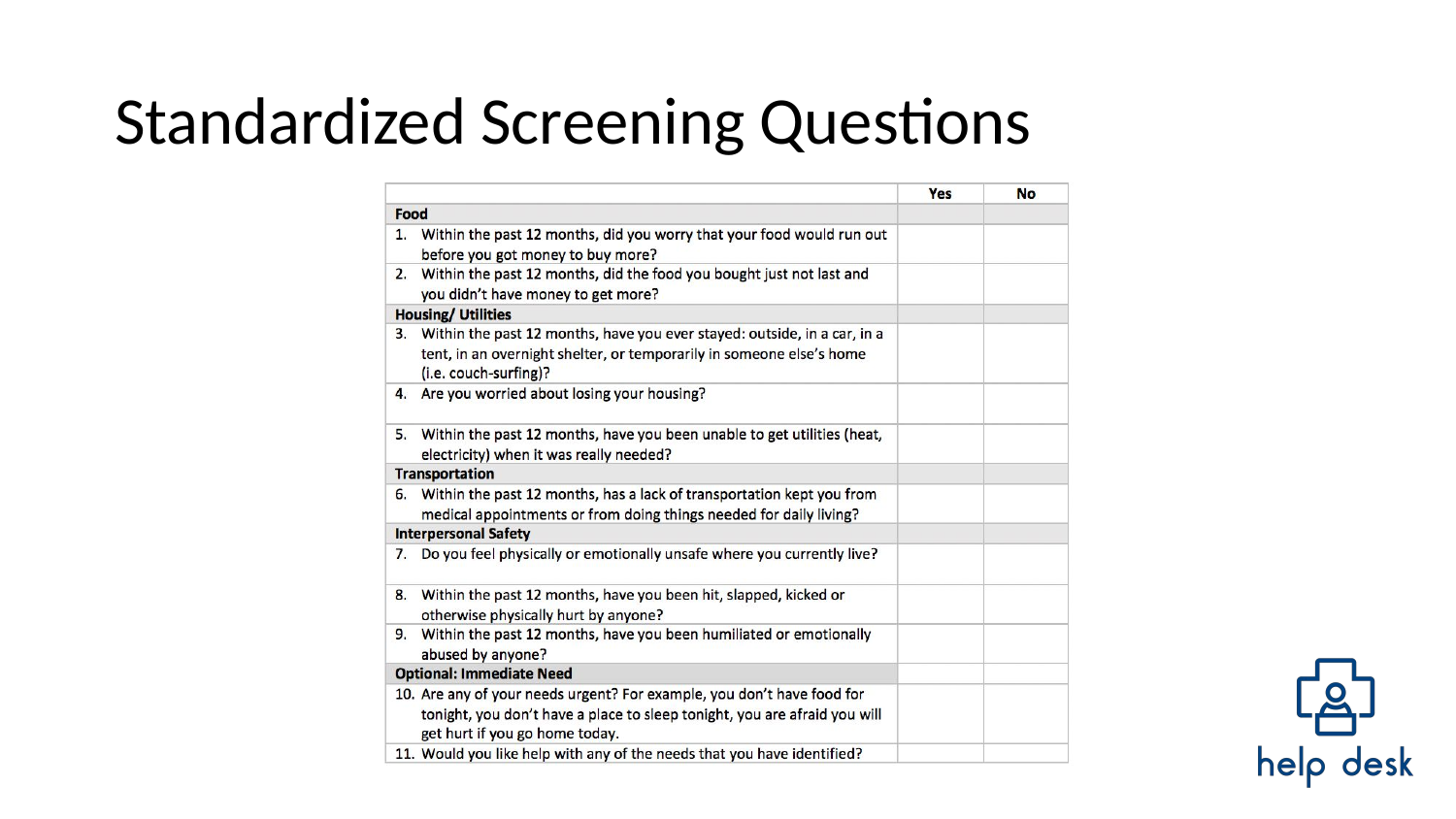

# Standardized Screening Questions

## Slide 31
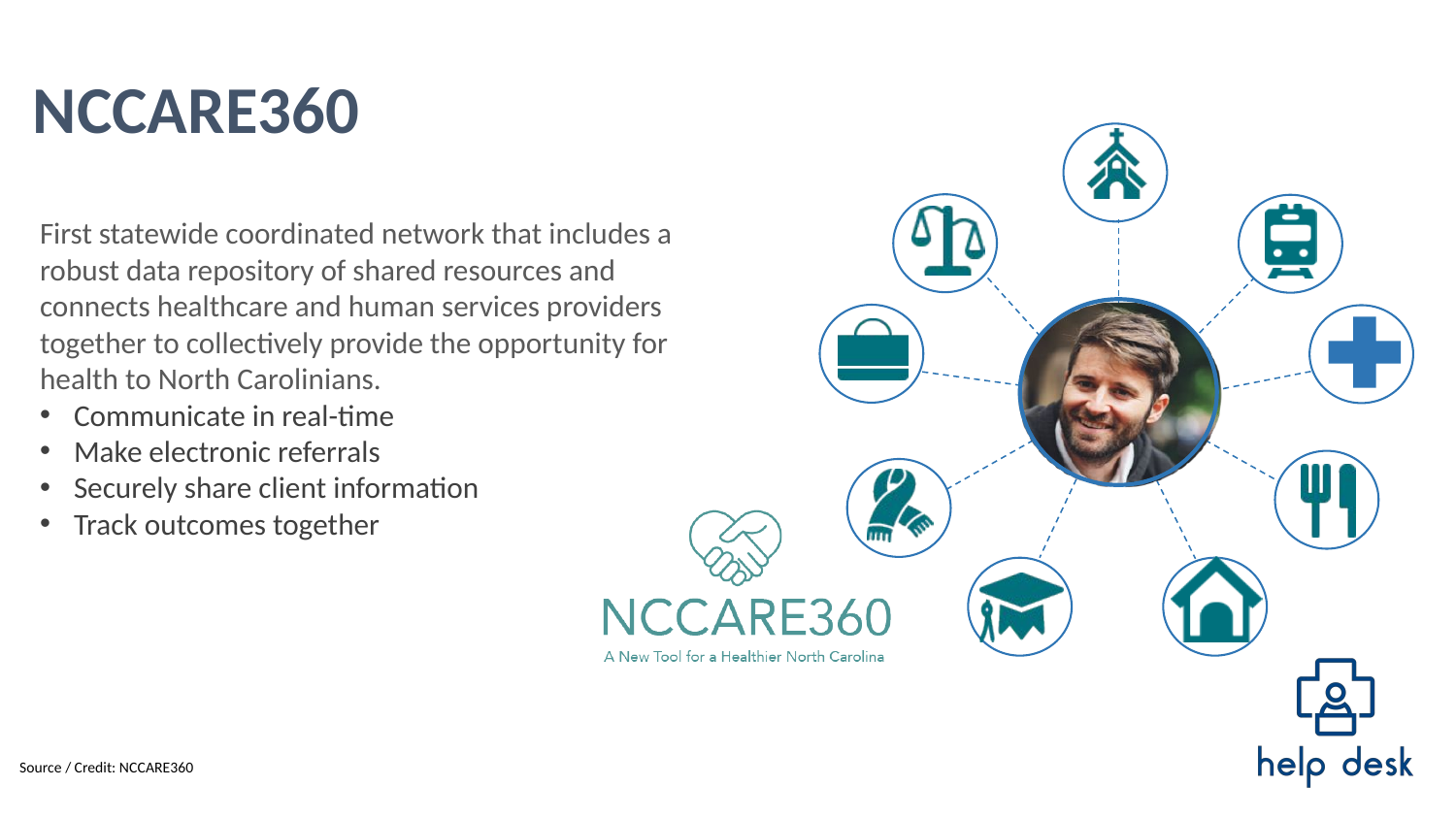

NCCARE360
First statewide coordinated network that includes a robust data repository of shared resources and connects healthcare and human services providers together to collectively provide the opportunity for health to North Carolinians.
Communicate in real-time
Make electronic referrals
Securely share client information
Track outcomes together
# V. Social Determinants of Health
Source / Credit: NCCARE360

## Slide 32
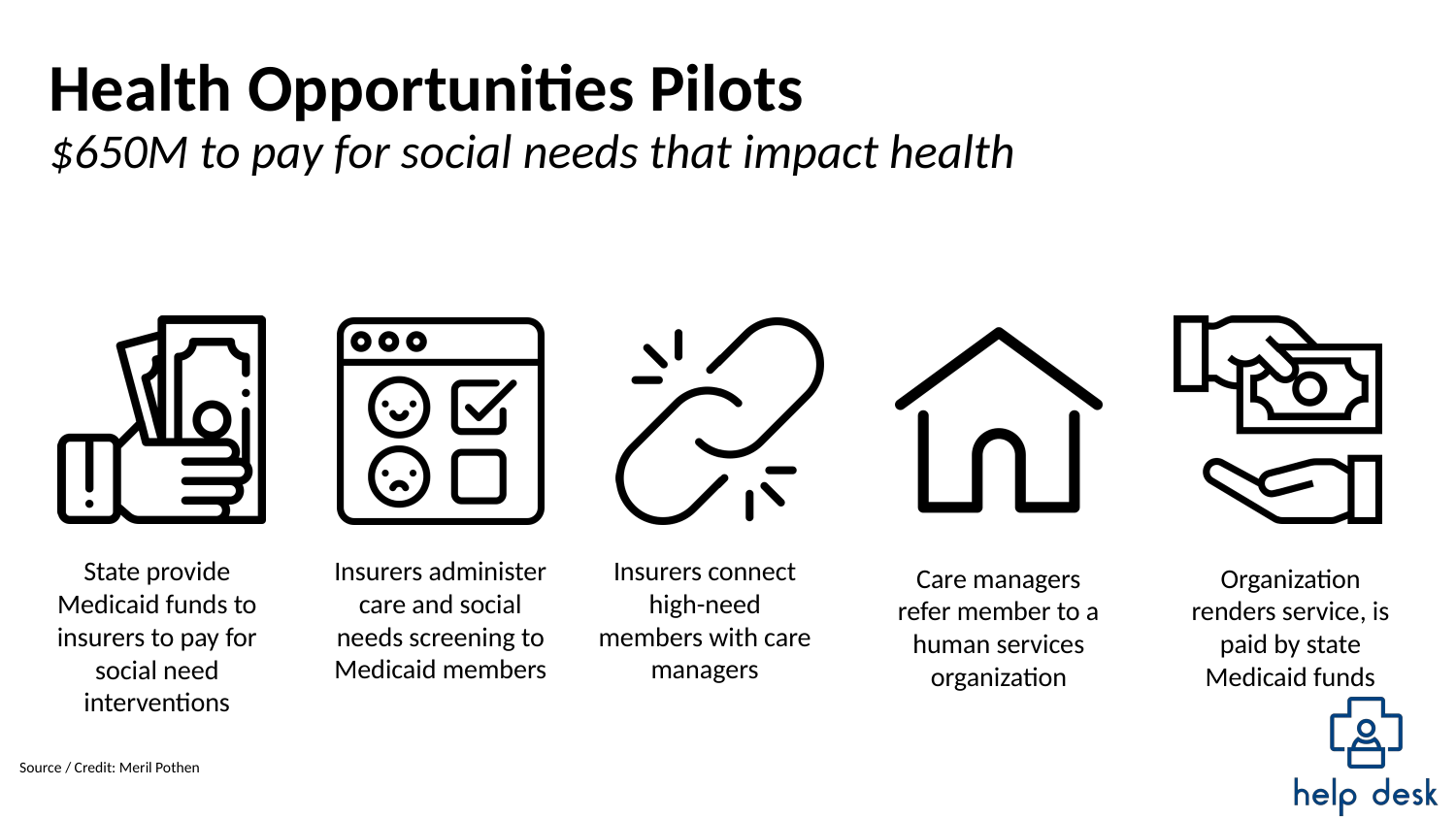

# Health Opportunities Pilots $650M to pay for social needs that impact health
Insurers administer care and social needs screening to Medicaid members
Insurers connect high-need members with care managers
State provide Medicaid funds to insurers to pay for social need interventions
Care managers refer member to a human services organization
Organization renders service, is paid by state Medicaid funds
Source / Credit: Meril Pothen

## Slide 33
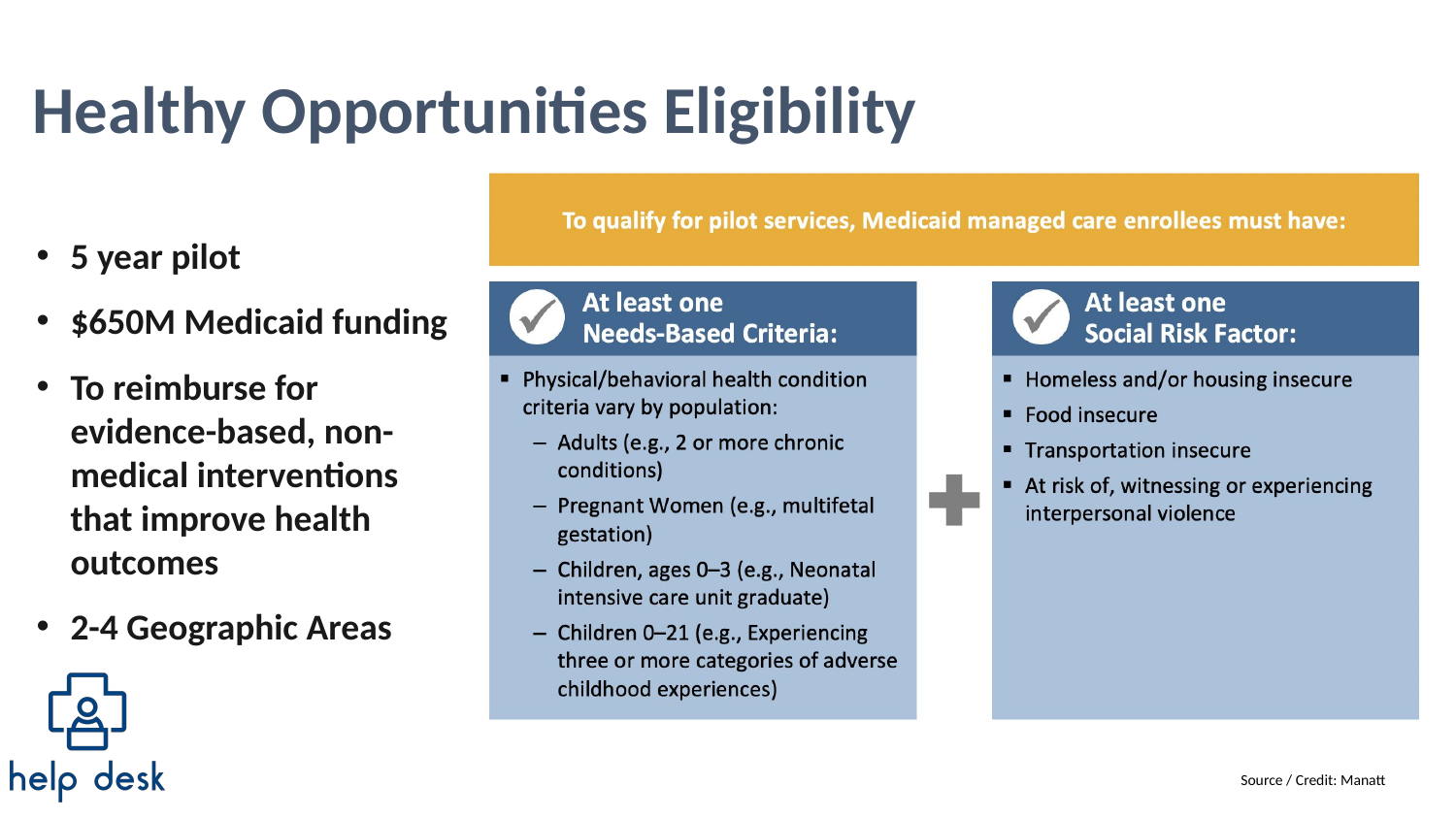

Healthy Opportunities Eligibility
5 year pilot
$650M Medicaid funding
To reimburse for evidence-based, non-medical interventions that improve health outcomes
2-4 Geographic Areas
# V. Social Determinants of Health
Source / Credit: Manatt

## Slide 34
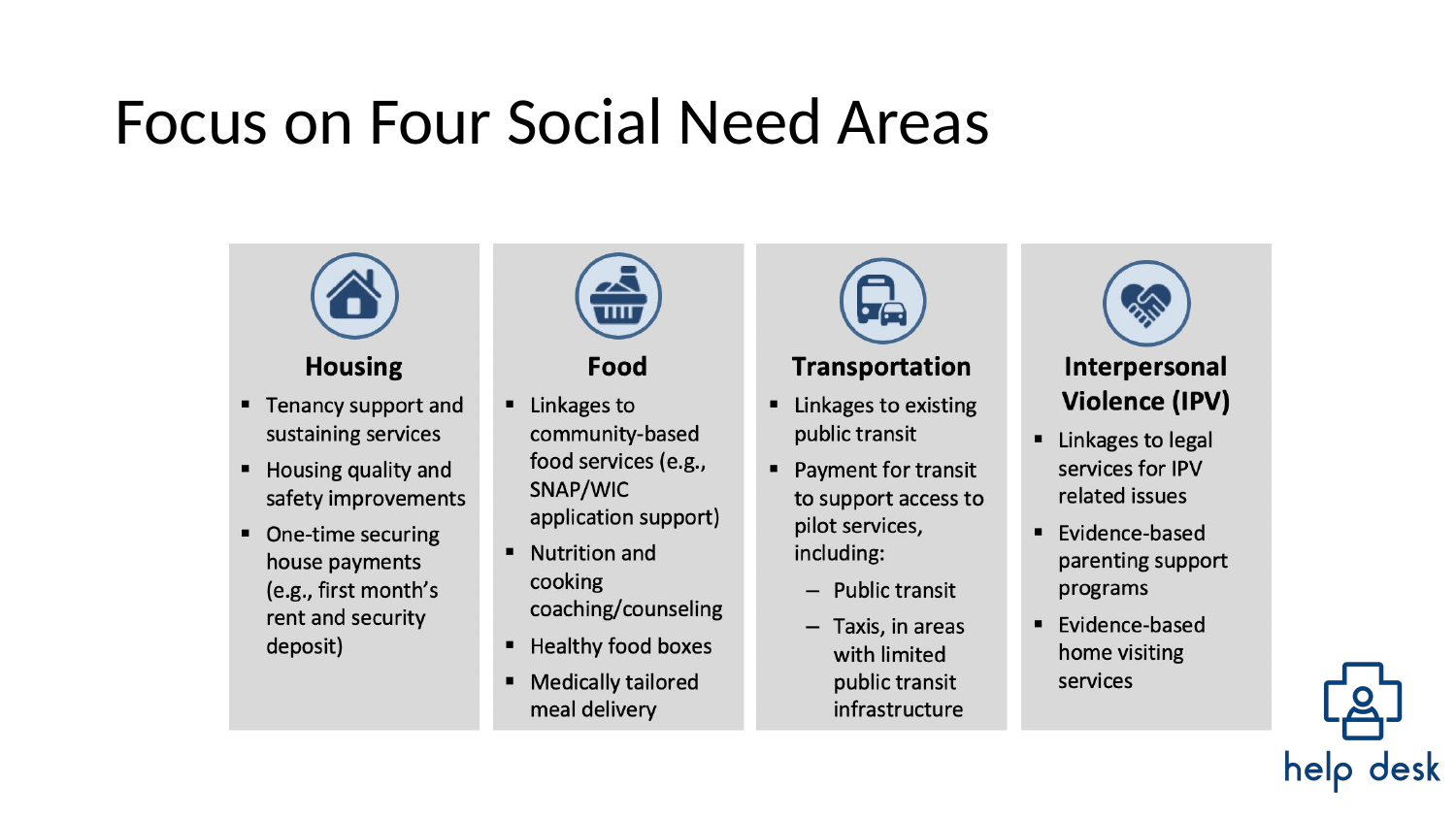

# Focus on Four Social Need Areas

## Slide 35
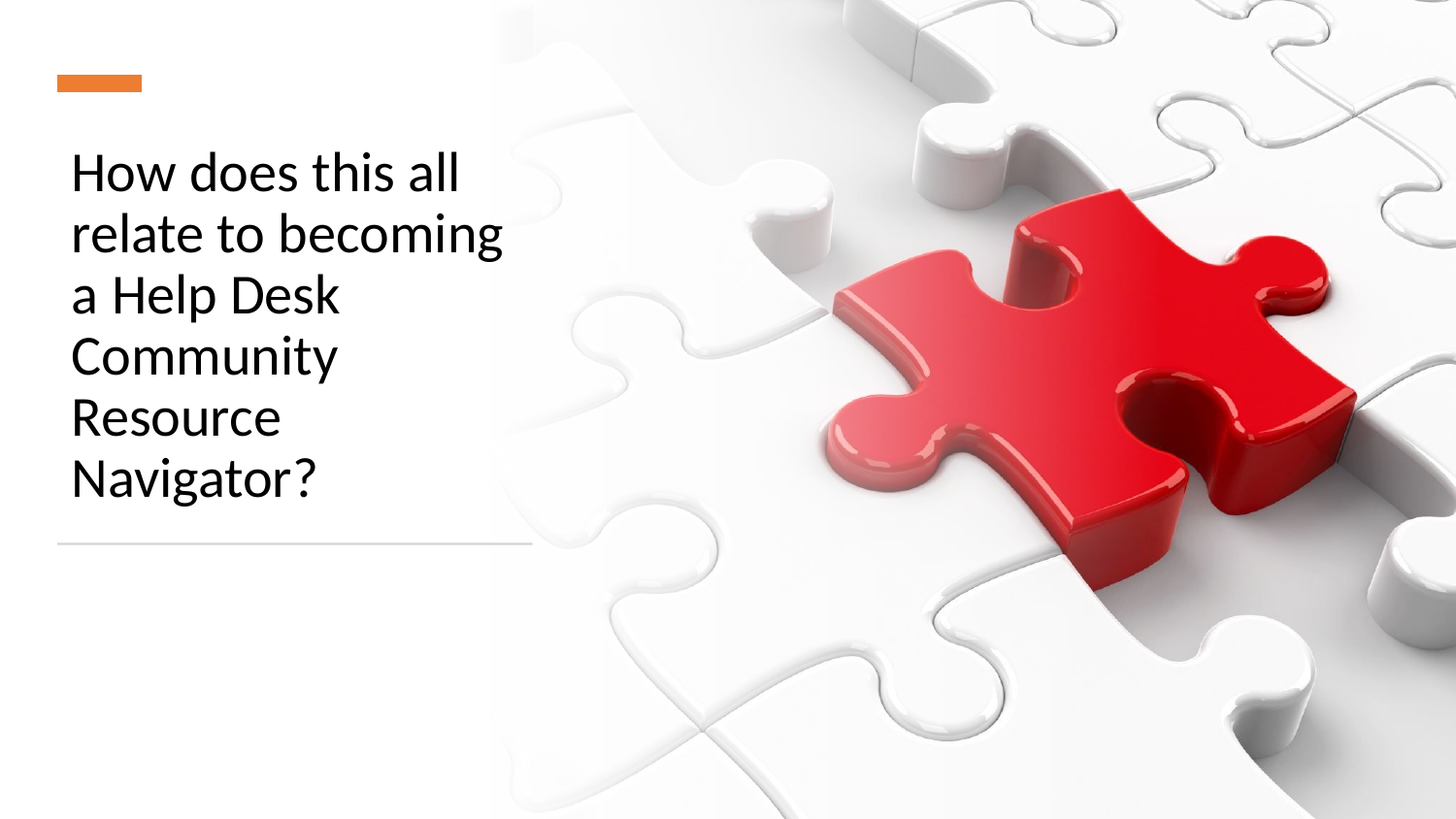

# How does this all relate to becoming a Help Desk Community Resource Navigator?

## Slide 36
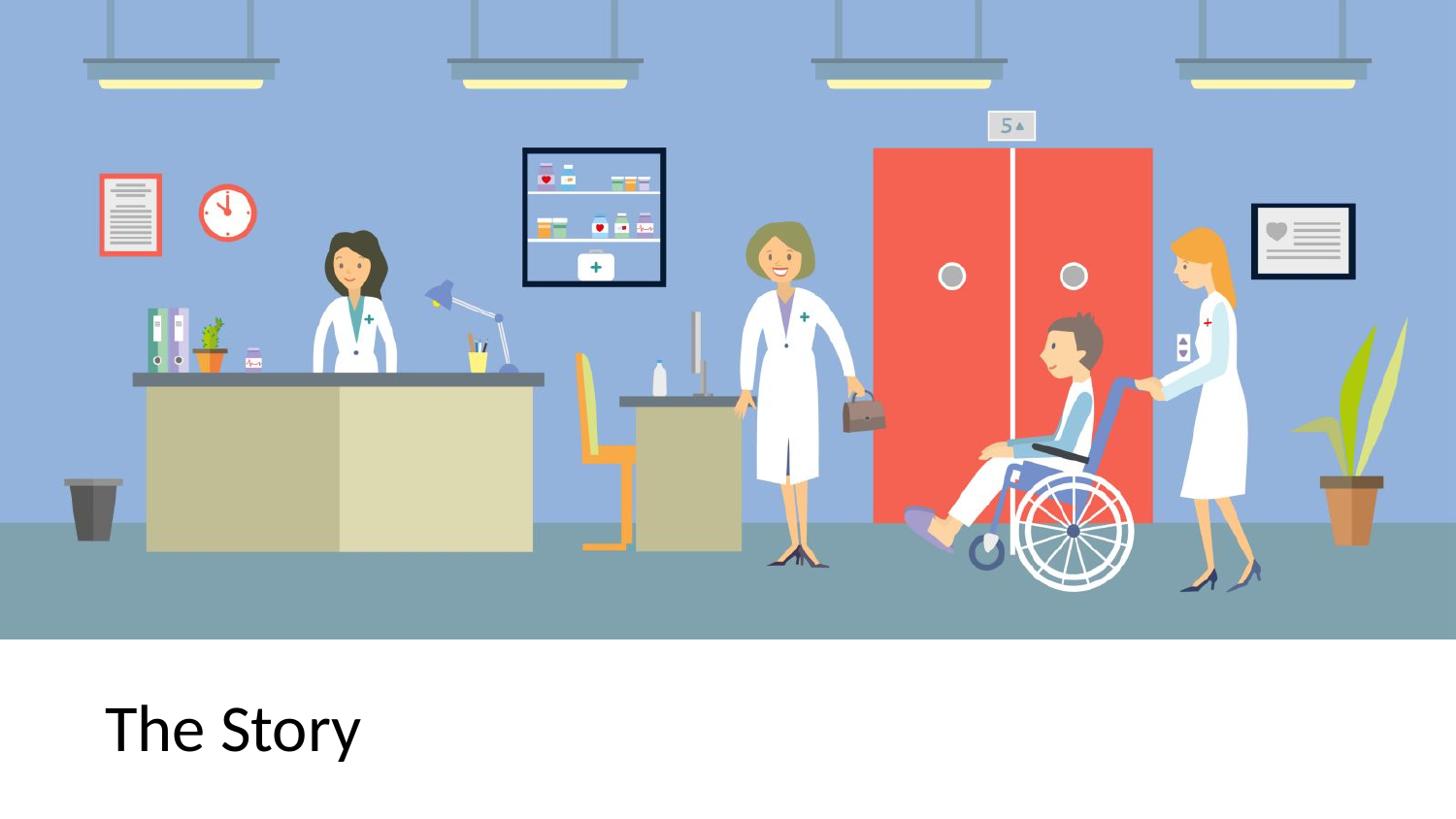

# The Story

## Slide 37
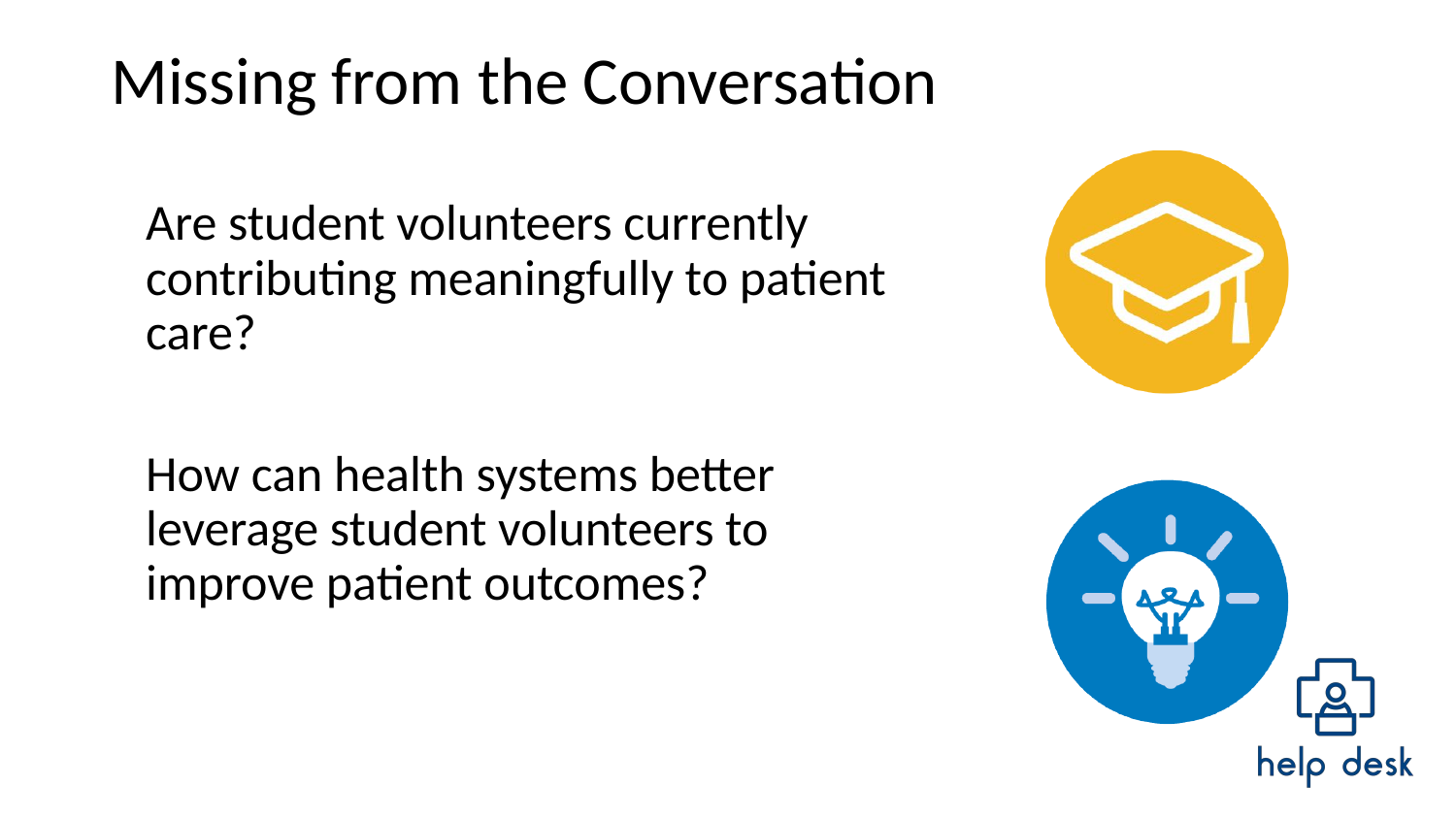

# Missing from the Conversation
Are student volunteers currently contributing meaningfully to patient care?
How can health systems better leverage student volunteers to improve patient outcomes?

## Slide 38
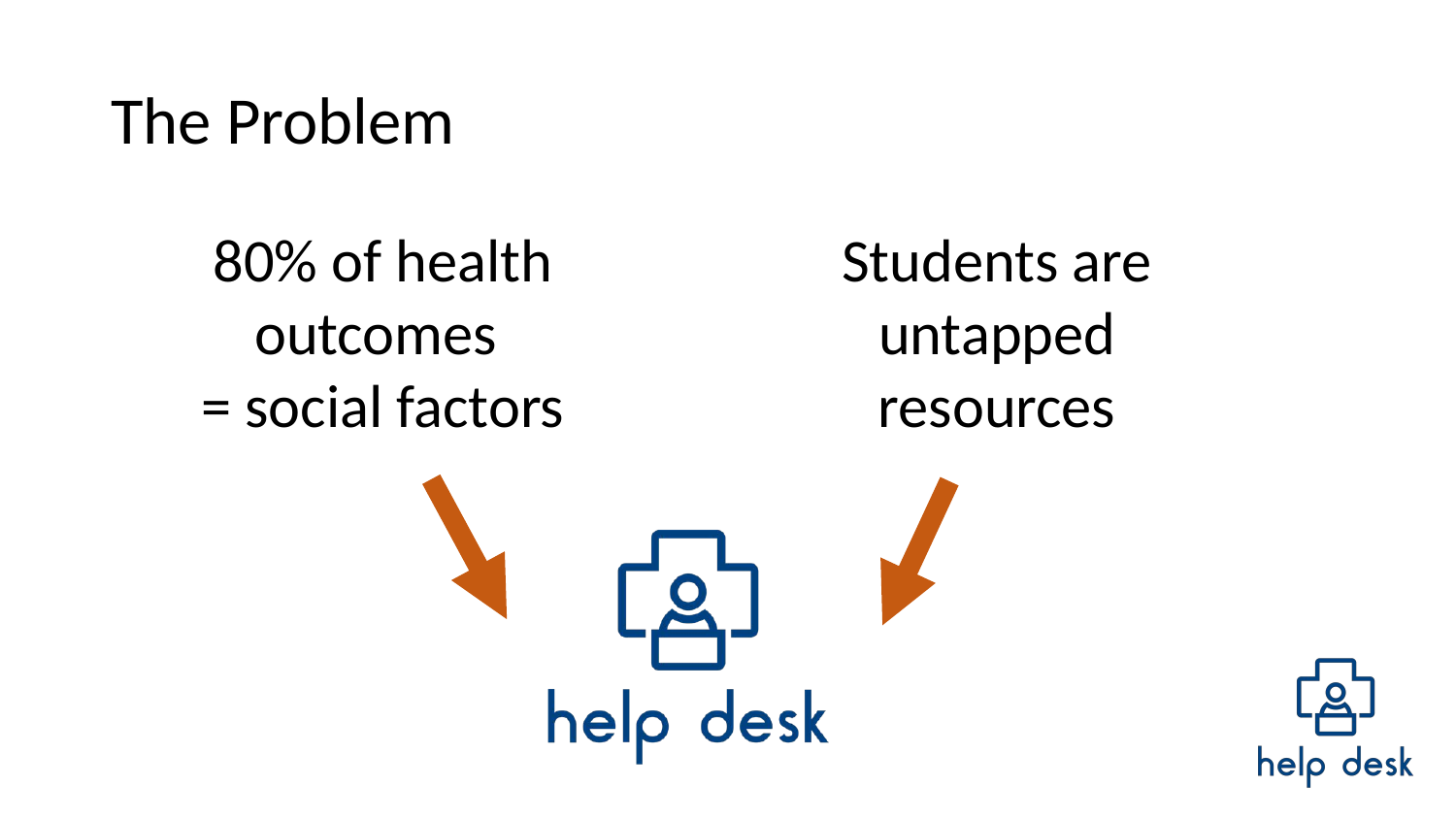

# The Problem
80% of health outcomes
= social factors
Students are untapped resources

## Slide 39
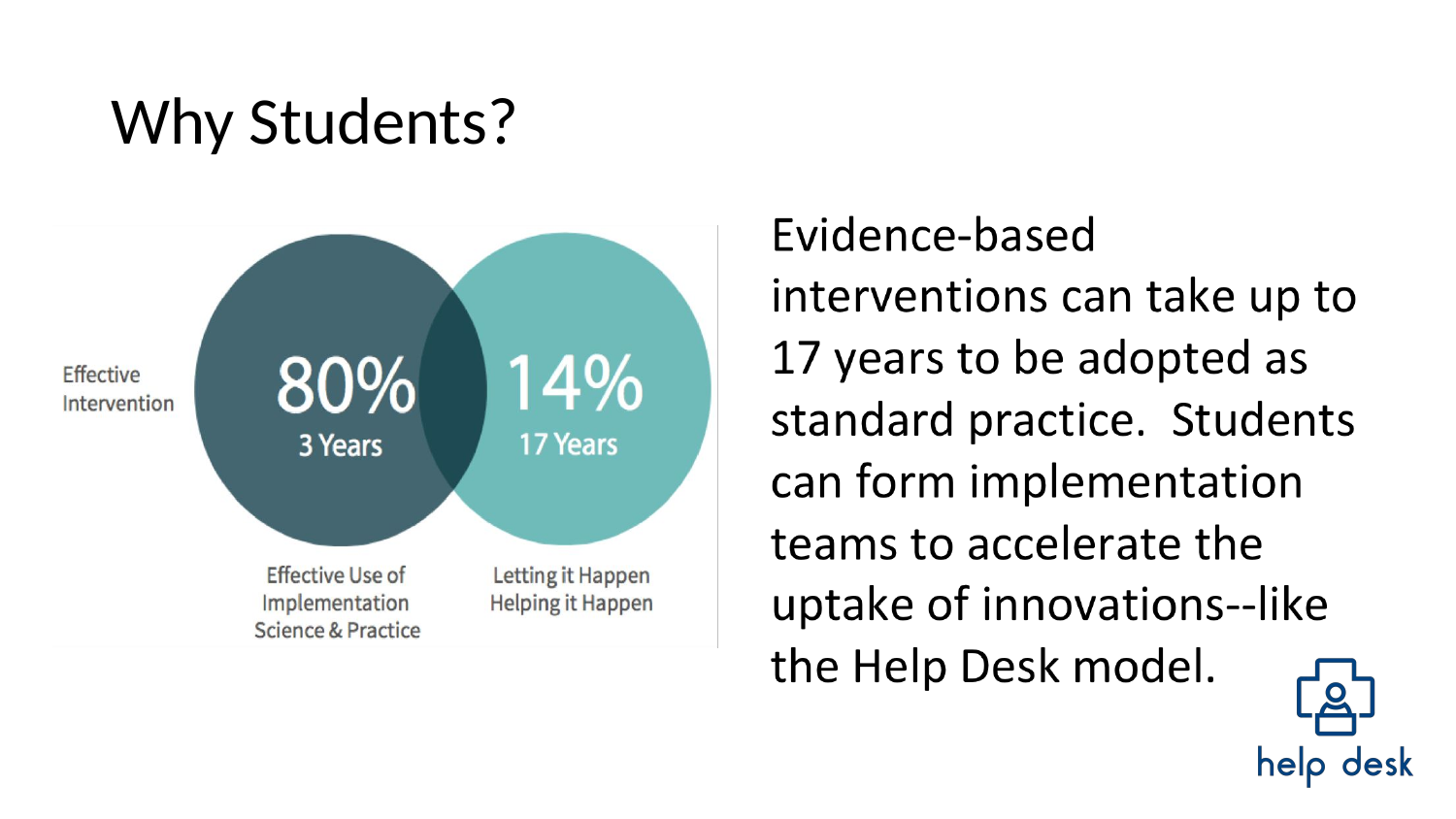

# Why Students?

## Slide 40
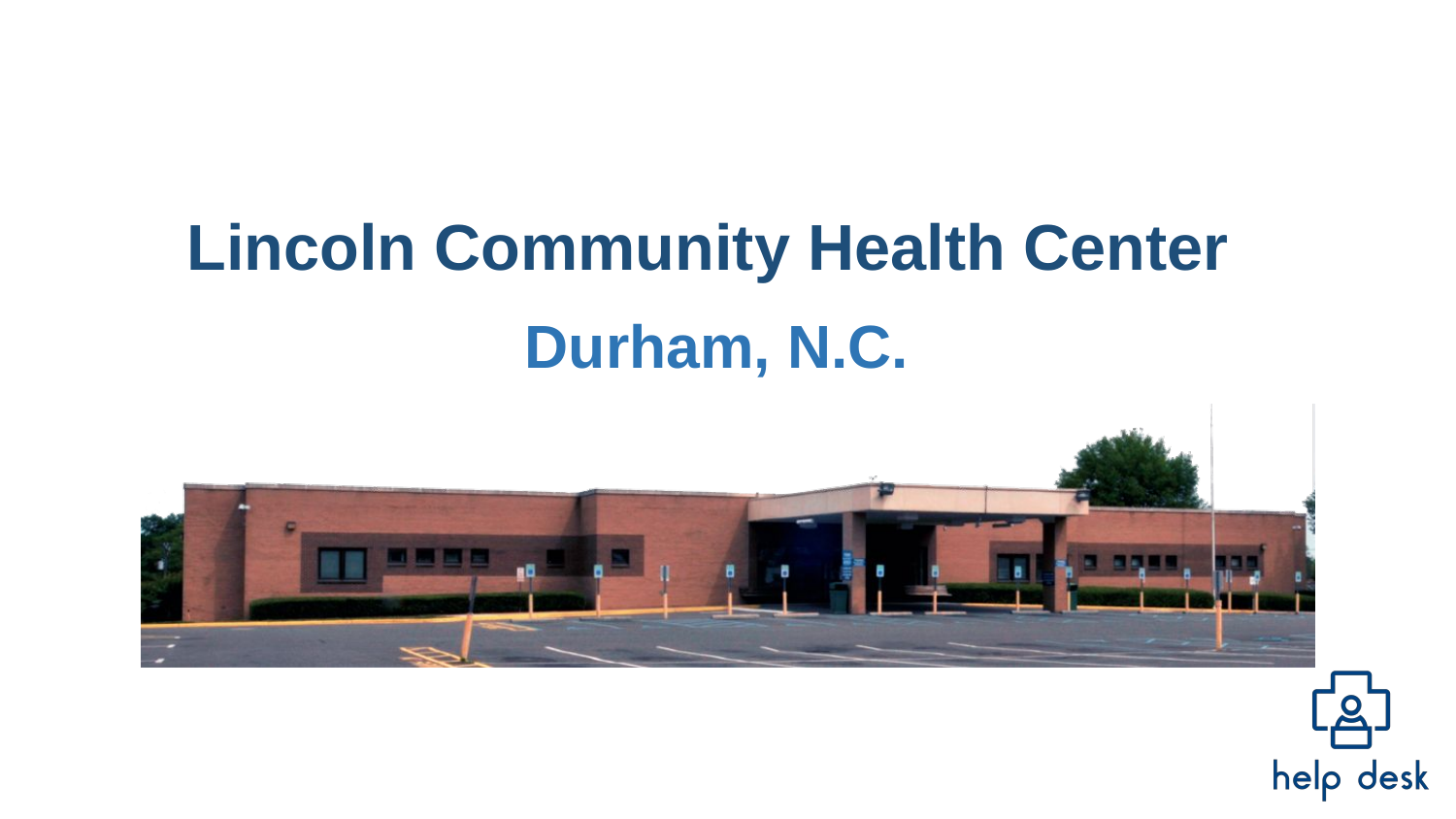

Lincoln Community Health Center
Durham, N.C.

## Slide 41
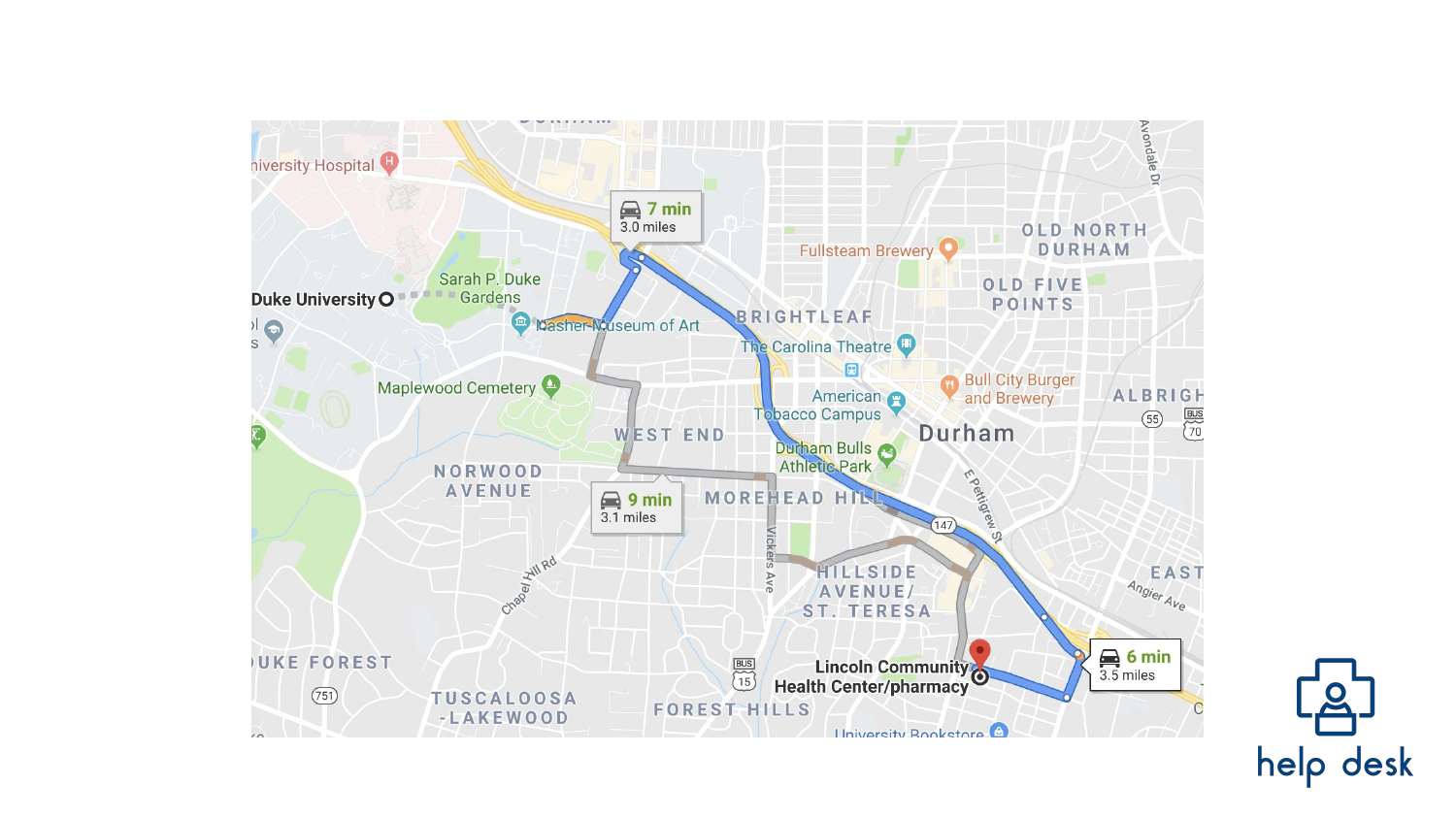

## Slide 42
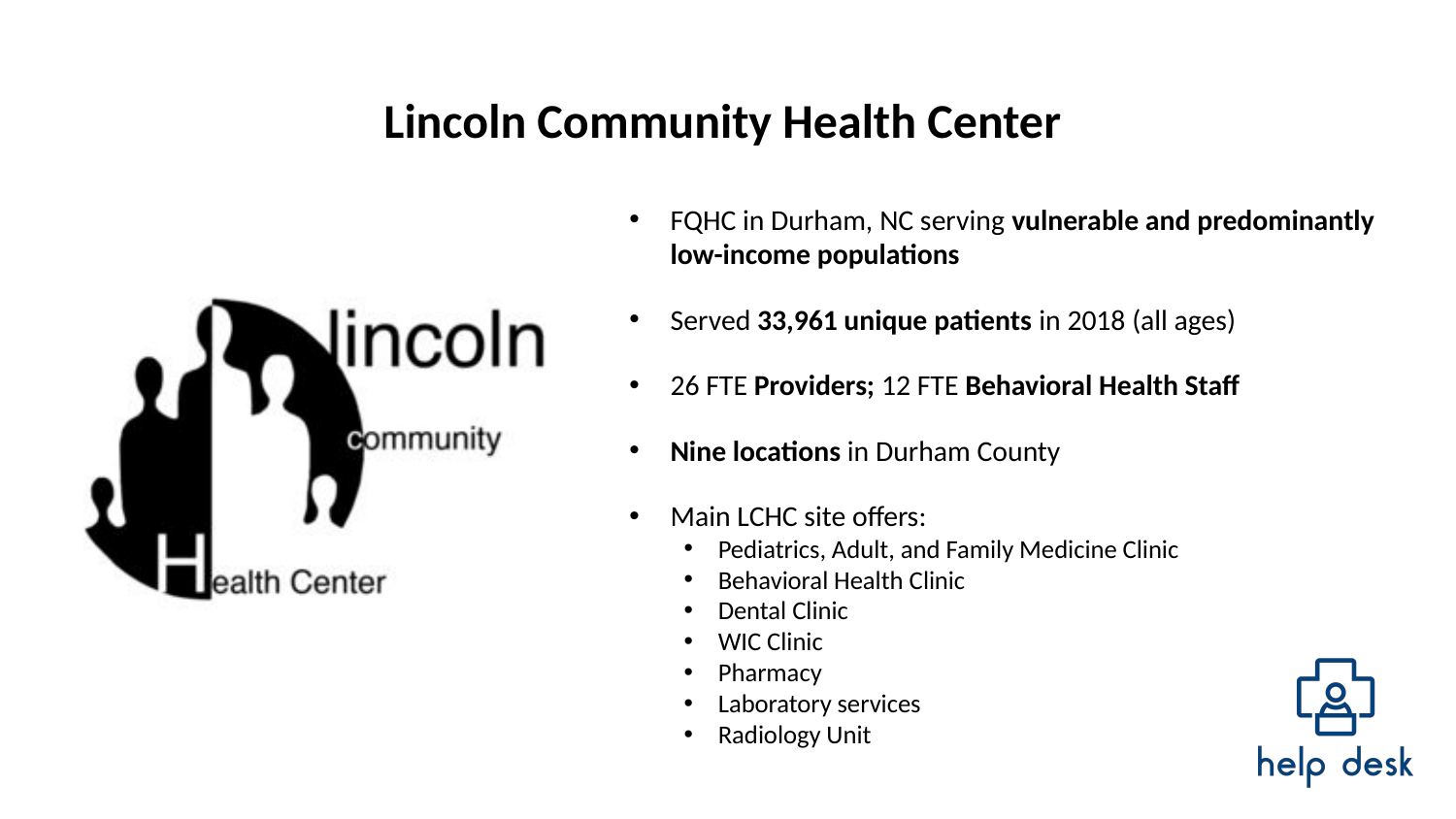

# Lincoln Community Health Center
FQHC in Durham, NC serving vulnerable and predominantly low-income populations
Served 33,961 unique patients in 2018 (all ages)
26 FTE Providers; 12 FTE Behavioral Health Staff
Nine locations in Durham County
Main LCHC site offers:
Pediatrics, Adult, and Family Medicine Clinic
Behavioral Health Clinic
Dental Clinic
WIC Clinic
Pharmacy
Laboratory services
Radiology Unit

## Slide 43
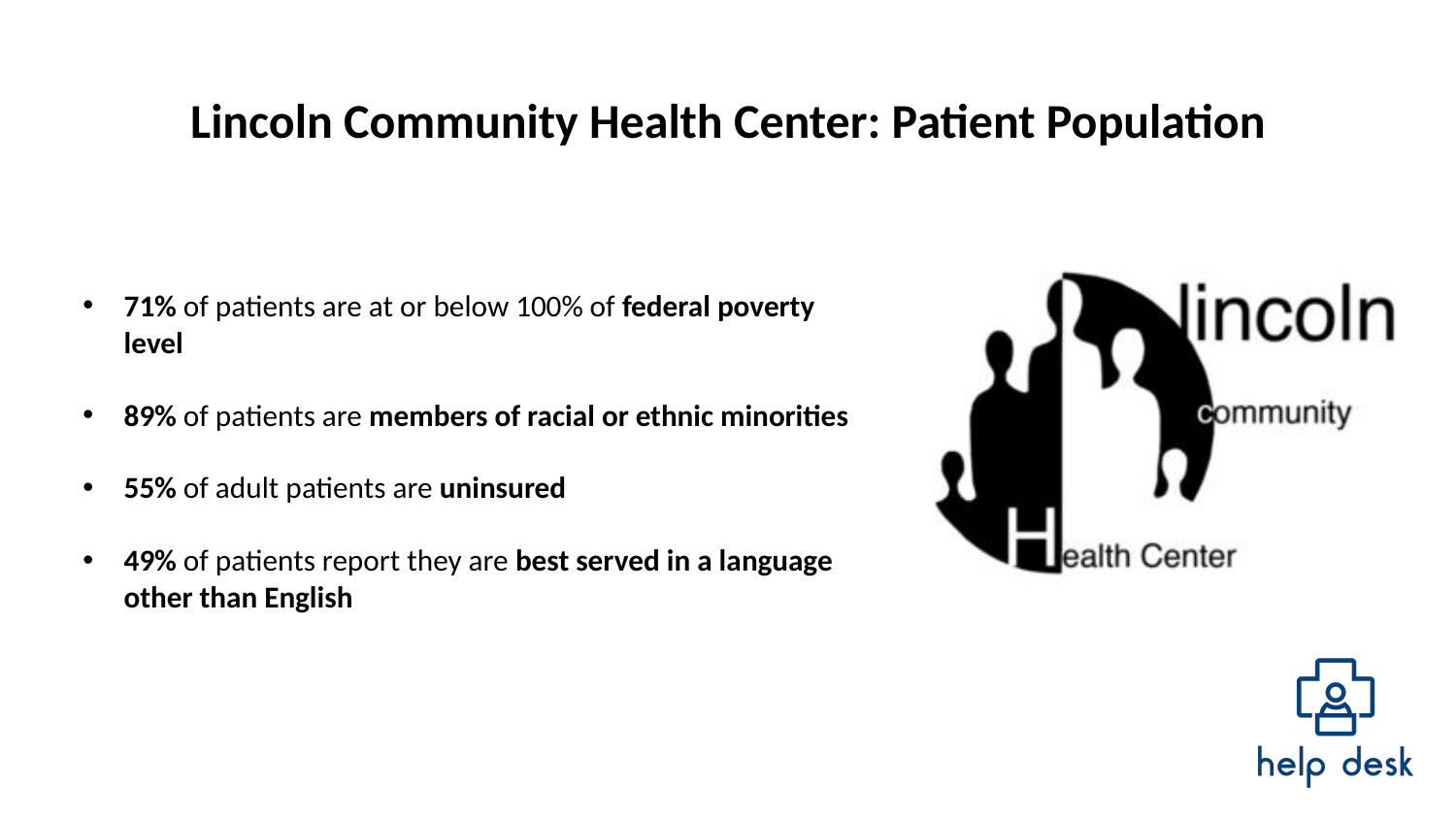

# Lincoln Community Health Center: Patient Population
71% of patients are at or below 100% of federal poverty level
89% of patients are members of racial or ethnic minorities
55% of adult patients are uninsured
49% of patients report they are best served in a language other than English

## Slide 44
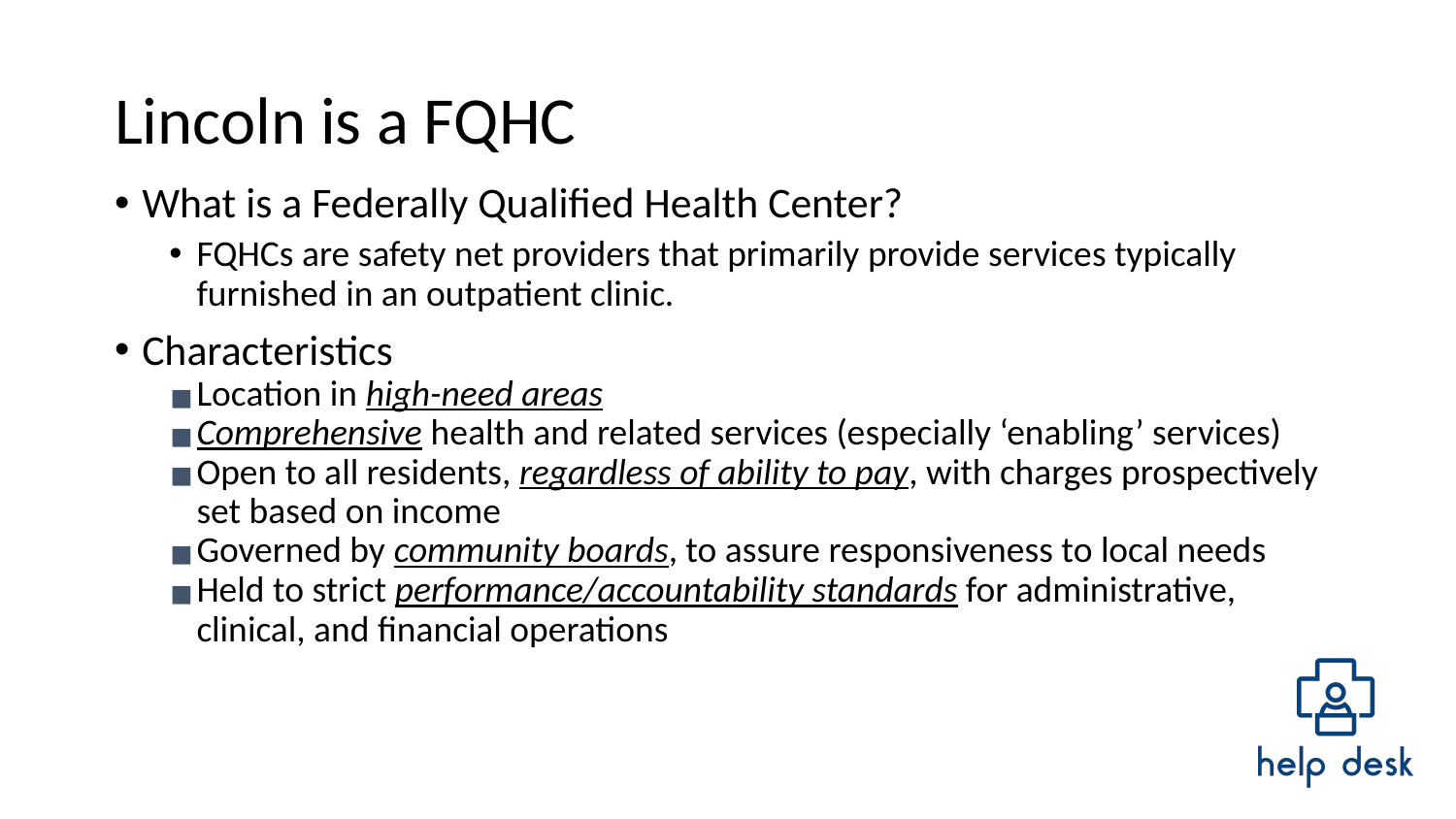

# Lincoln is a FQHC
What is a Federally Qualified Health Center?
FQHCs are safety net providers that primarily provide services typically furnished in an outpatient clinic.
Characteristics
Location in high-need areas
Comprehensive health and related services (especially ‘enabling’ services)
Open to all residents, regardless of ability to pay, with charges prospectively set based on income
Governed by community boards, to assure responsiveness to local needs
Held to strict performance/accountability standards for administrative, clinical, and financial operations

## Slide 45
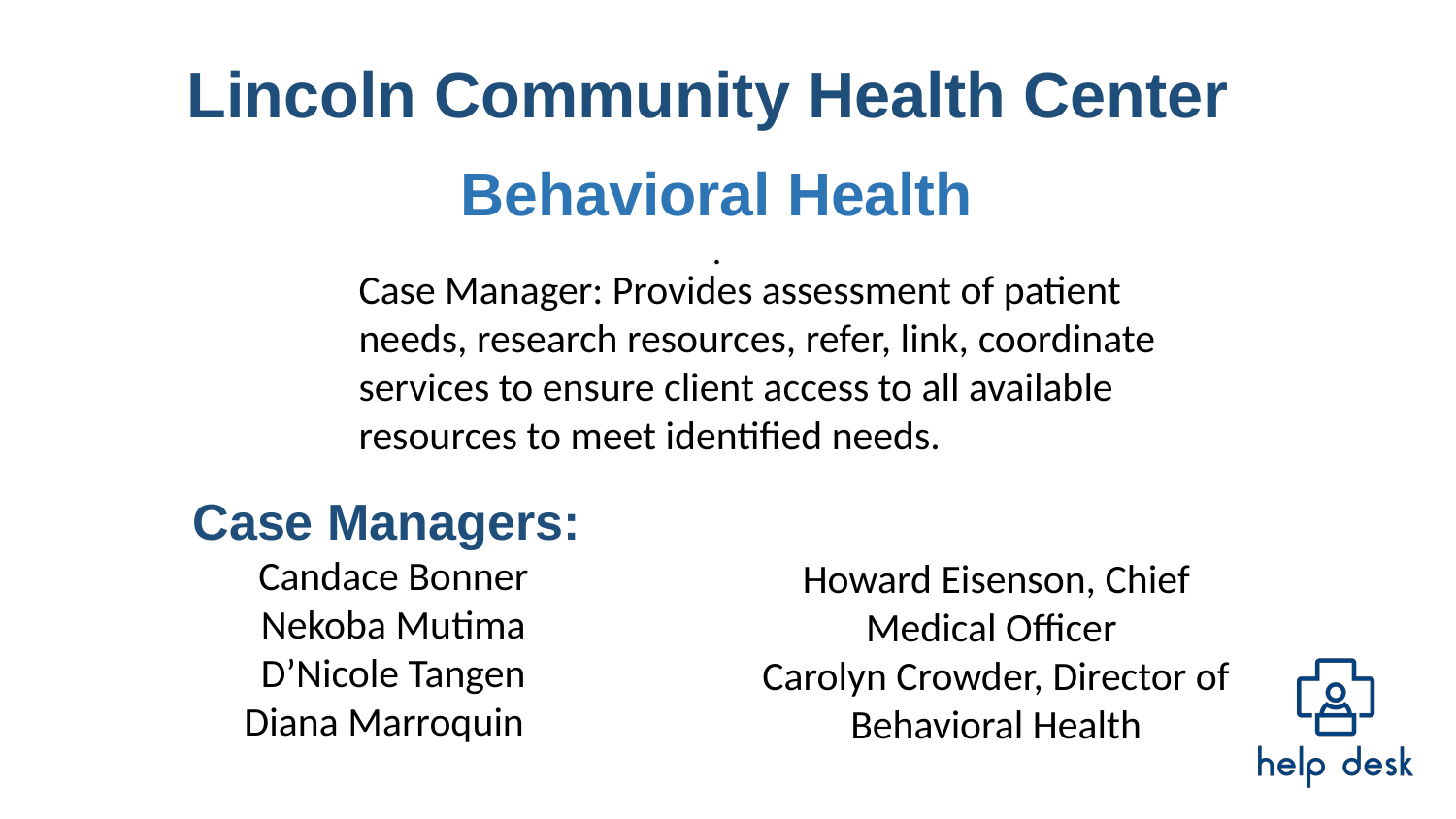

Lincoln Community Health Center
Behavioral Health
.
Case Manager: Provides assessment of patient needs, research resources, refer, link, coordinate services to ensure client access to all available resources to meet identified needs.
Case Managers:
Candace Bonner
Nekoba Mutima
D’Nicole Tangen
Diana Marroquin
Howard Eisenson, Chief Medical Officer
Carolyn Crowder, Director of Behavioral Health

## Slide 46
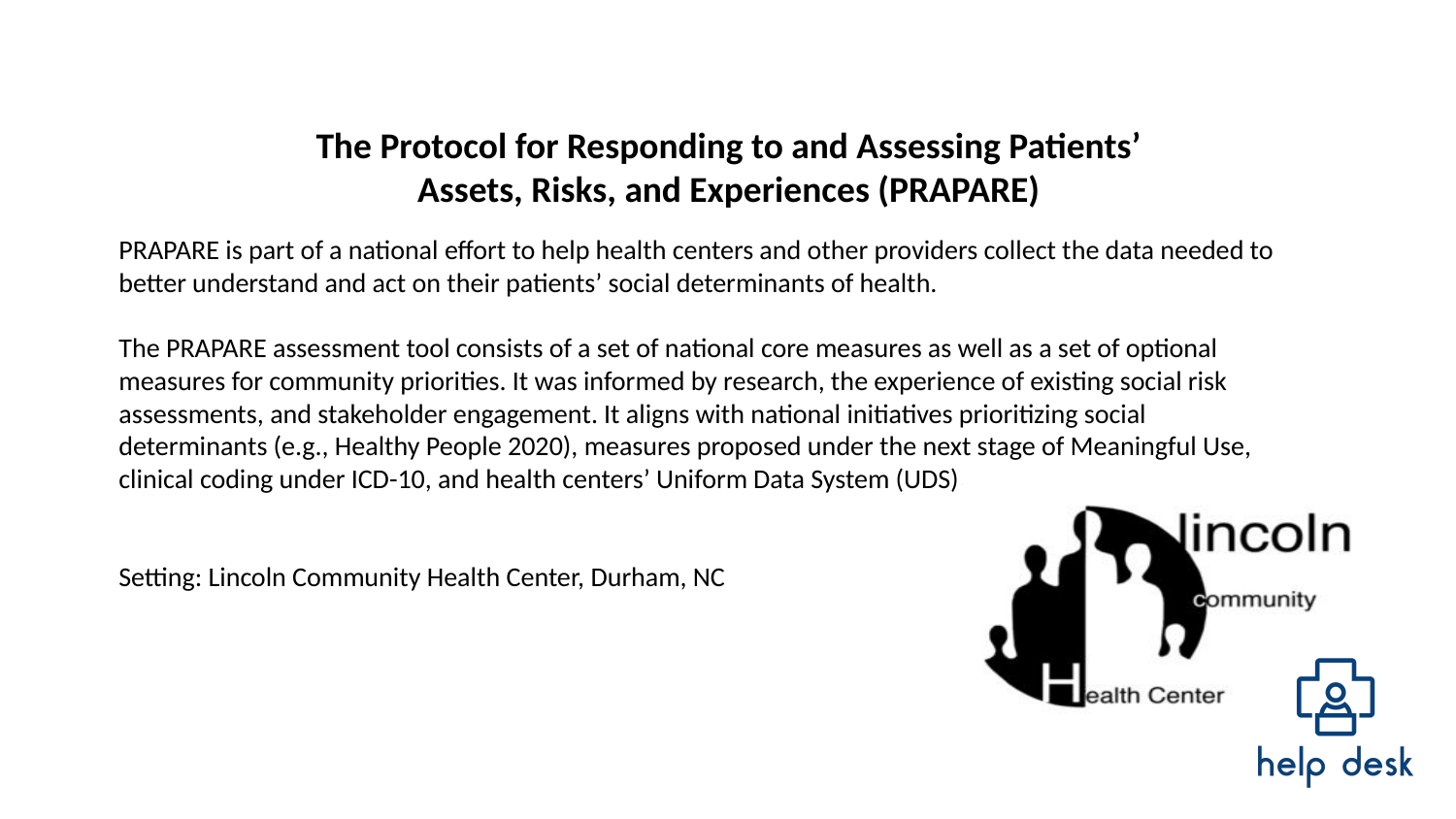

The Protocol for Responding to and Assessing Patients’ Assets, Risks, and Experiences (PRAPARE)
PRAPARE is part of a national effort to help health centers and other providers collect the data needed to better understand and act on their patients’ social determinants of health.
The PRAPARE assessment tool consists of a set of national core measures as well as a set of optional measures for community priorities. It was informed by research, the experience of existing social risk assessments, and stakeholder engagement. It aligns with national initiatives prioritizing social determinants (e.g., Healthy People 2020), measures proposed under the next stage of Meaningful Use, clinical coding under ICD-10, and health centers’ Uniform Data System (UDS).
Setting: Lincoln Community Health Center, Durham, NC

## Slide 47
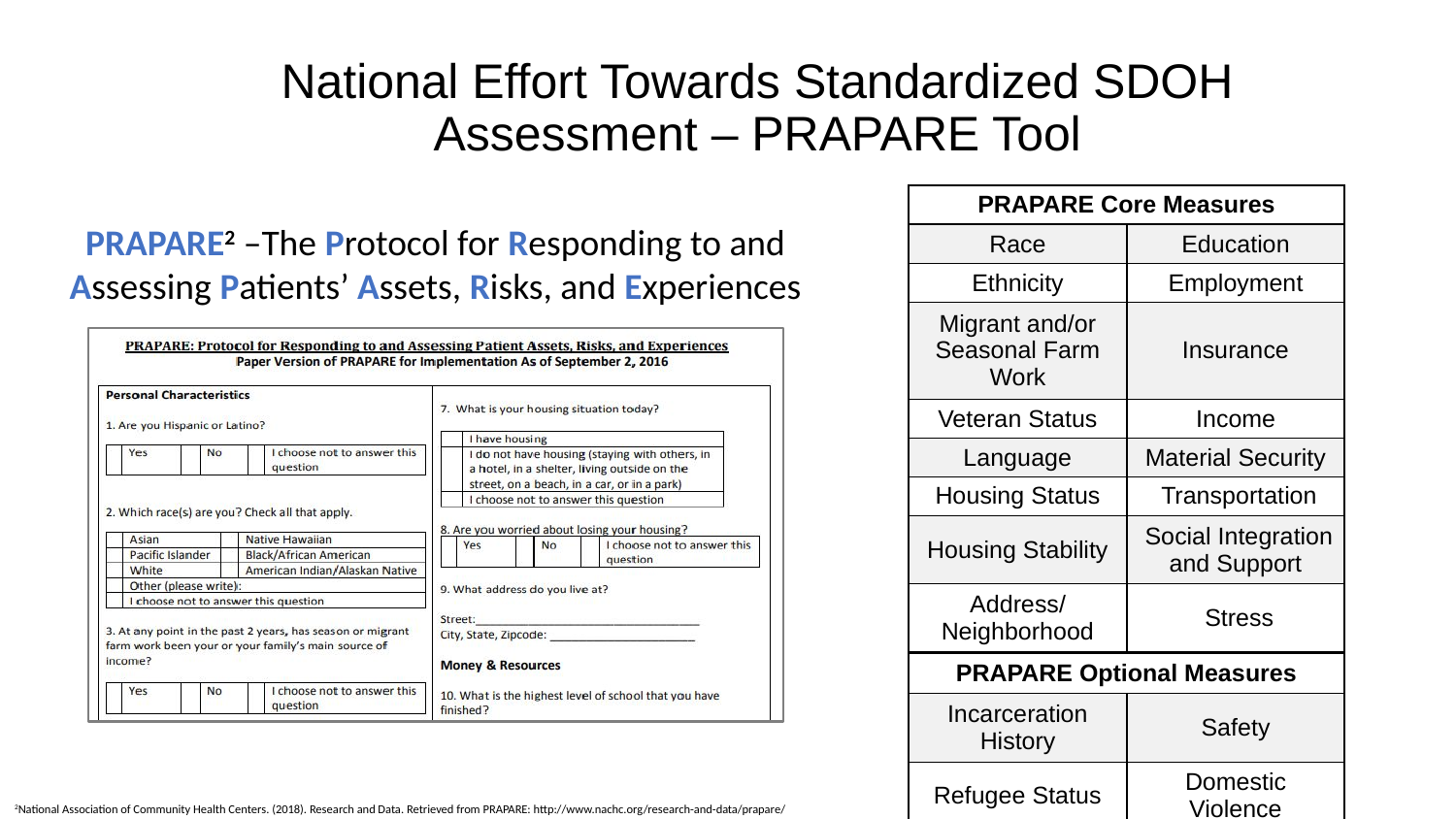

# National Effort Towards Standardized SDOH Assessment – PRAPARE Tool
| PRAPARE Core Measures | |
| --- | --- |
| Race | Education |
| Ethnicity | Employment |
| Migrant and/or Seasonal Farm Work | Insurance |
| Veteran Status | Income |
| Language | Material Security |
| Housing Status | Transportation |
| Housing Stability | Social Integration and Support |
| Address/ Neighborhood | Stress |
PRAPARE2 –The Protocol for Responding to and Assessing Patients’ Assets, Risks, and Experiences
| PRAPARE Optional Measures | |
| --- | --- |
| Incarceration History | Safety |
| Refugee Status | Domestic Violence |
2National Association of Community Health Centers. (2018). Research and Data. Retrieved from PRAPARE: http://www.nachc.org/research-and-data/prapare/

## Slide 48
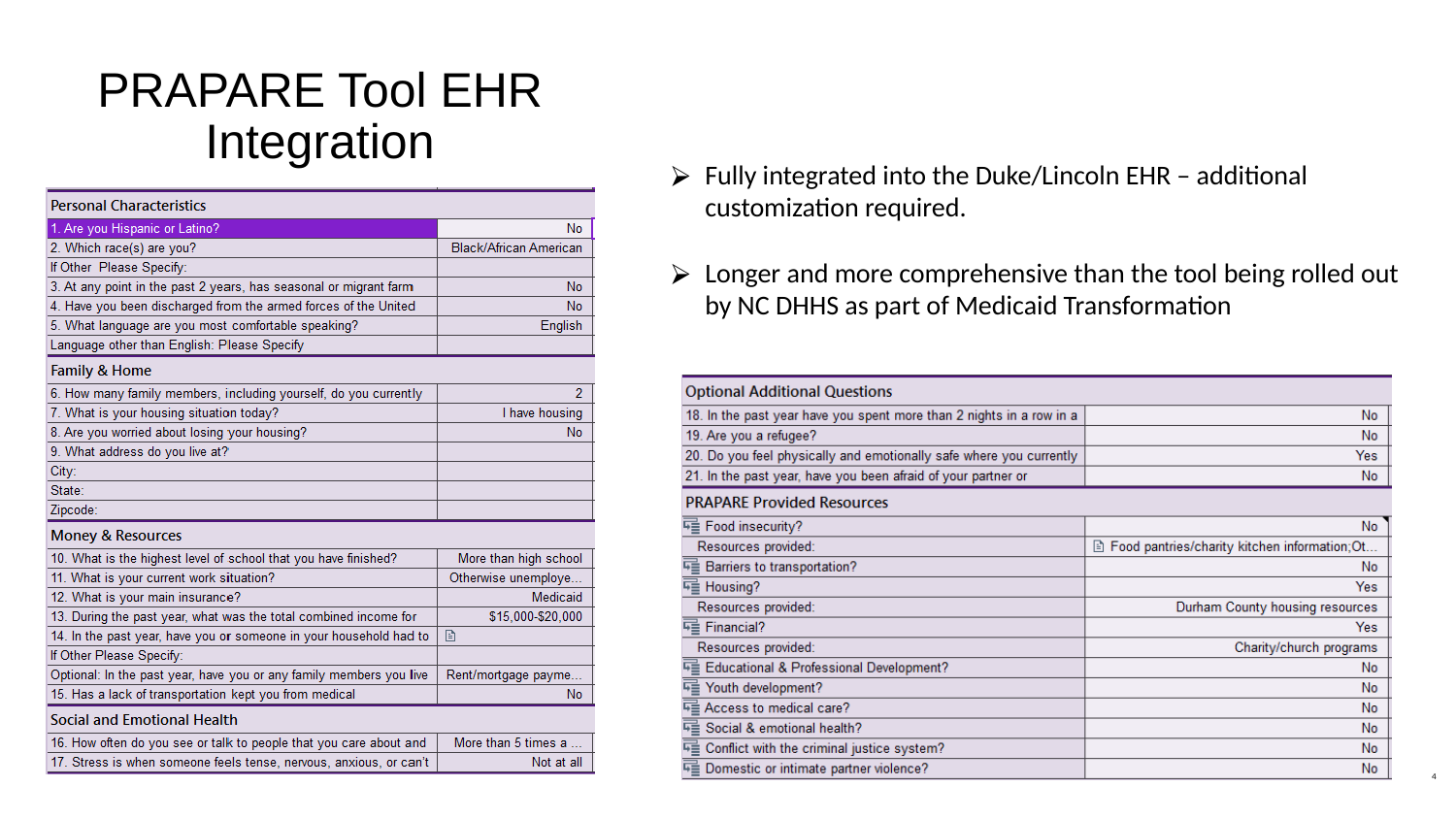

# PRAPARE Tool EHR Integration
Fully integrated into the Duke/Lincoln EHR – additional customization required.
Longer and more comprehensive than the tool being rolled out by NC DHHS as part of Medicaid Transformation
4

## Slide 49
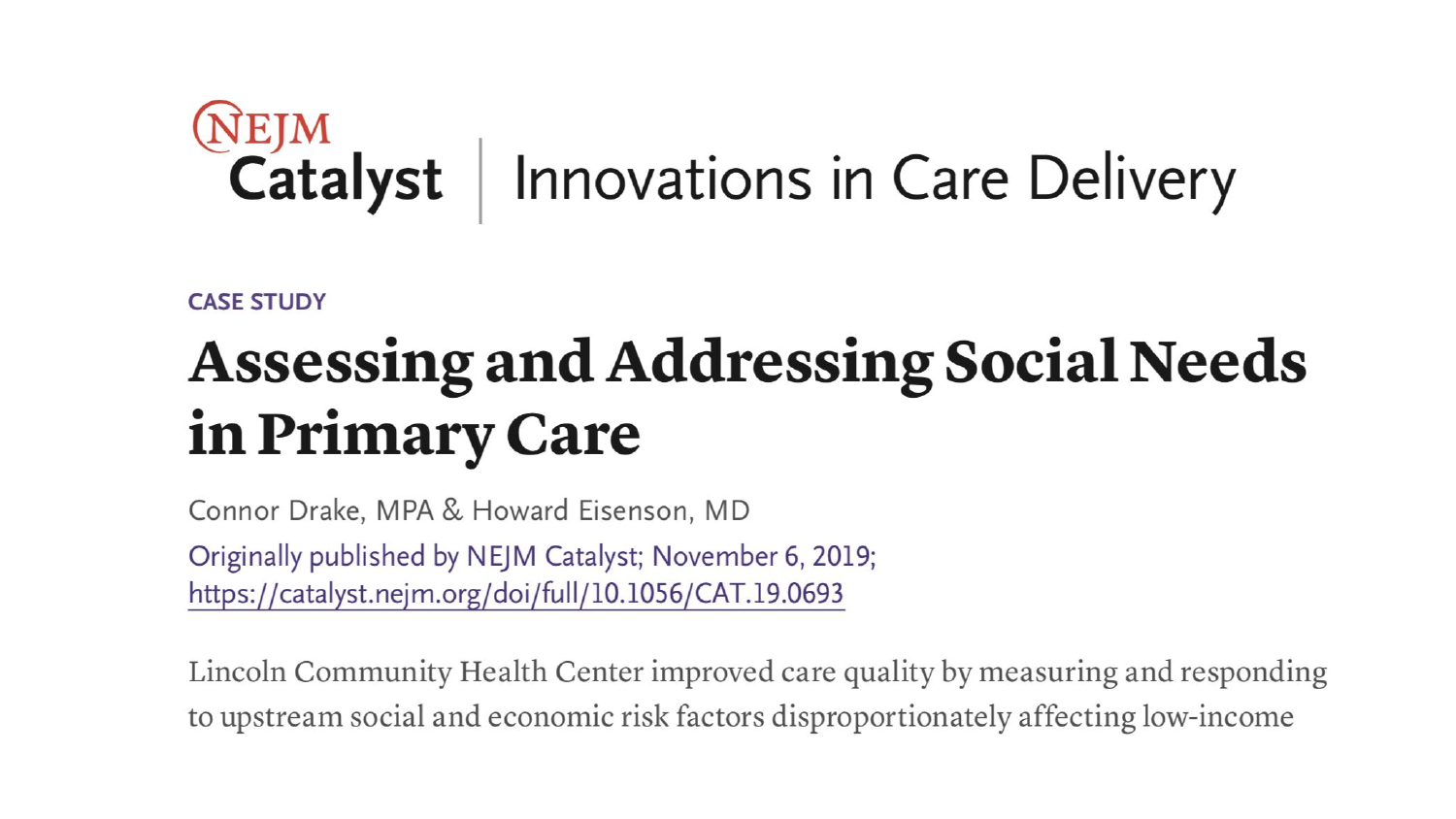

## Slide 50
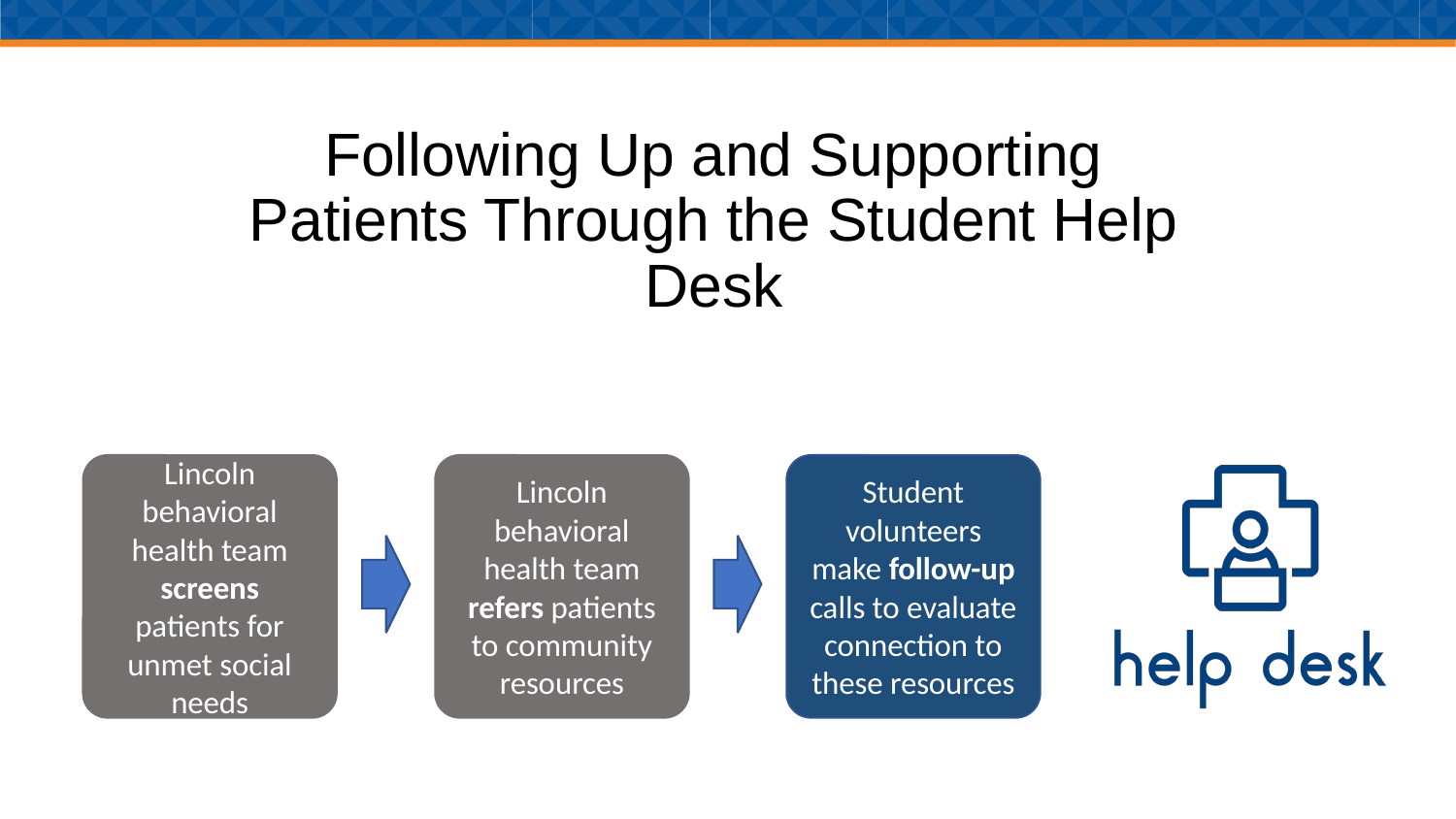

# Following Up and Supporting Patients Through the Student Help Desk
Lincoln behavioral health team screens patients for unmet social needs
Lincoln behavioral health team refers patients to community resources
Student volunteers make follow-up calls to evaluate connection to these resources

## Slide 51
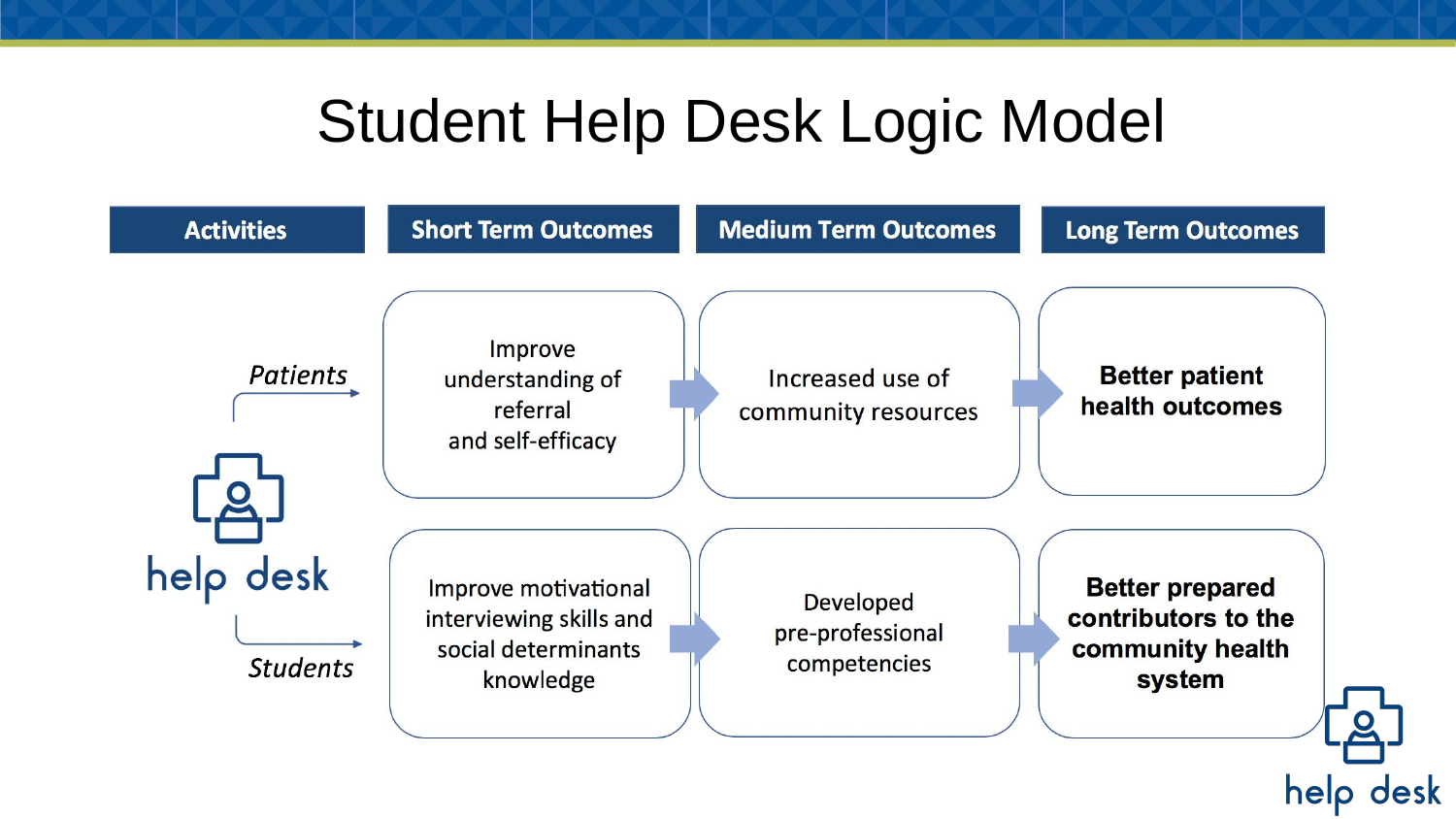

Student Help Desk Logic Model

## Slide 52
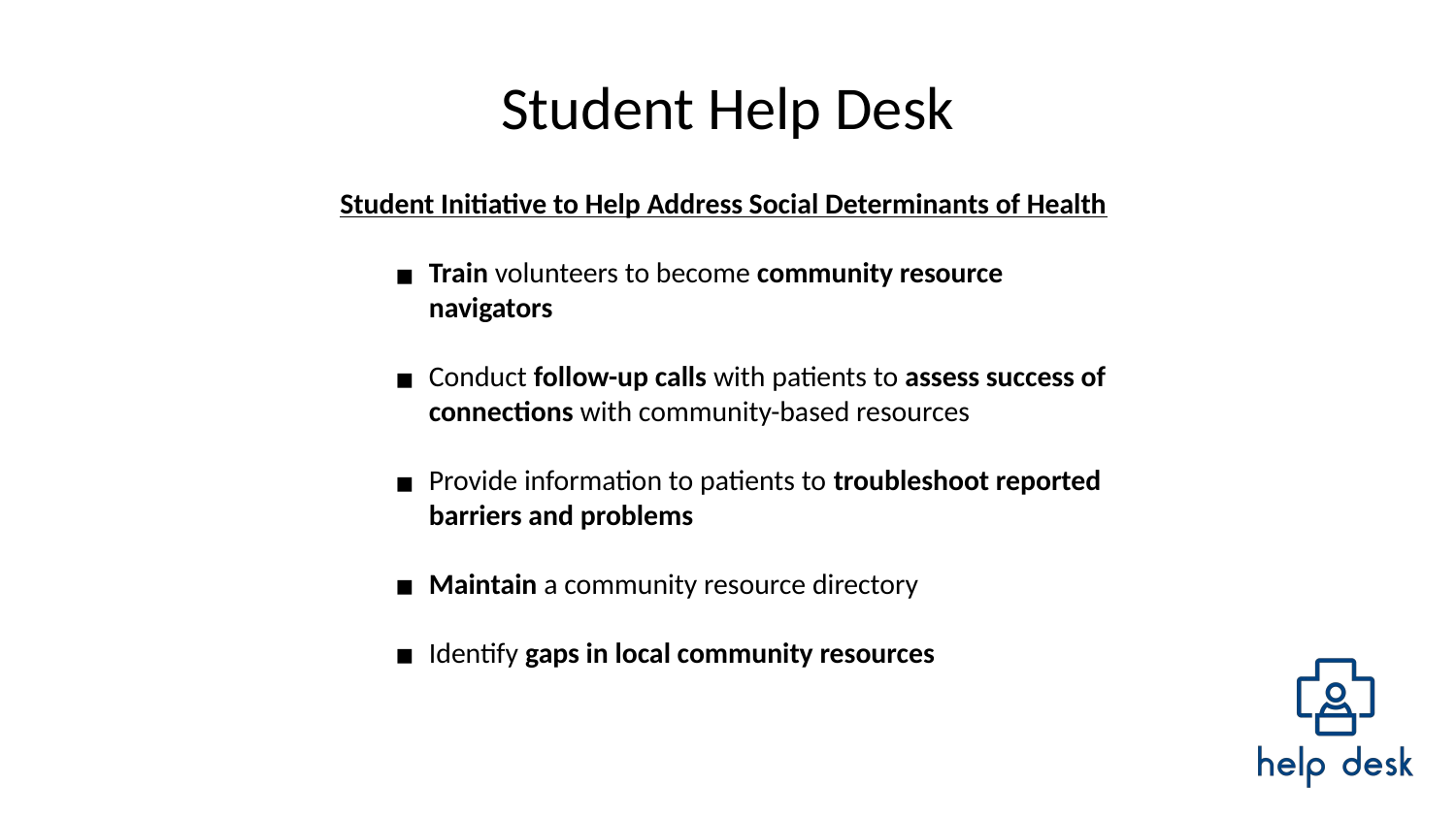

Student Help Desk
Student Initiative to Help Address Social Determinants of Health
Train volunteers to become community resource navigators
Conduct follow-up calls with patients to assess success of connections with community-based resources
Provide information to patients to troubleshoot reported barriers and problems
Maintain a community resource directory
Identify gaps in local community resources

## Slide 53
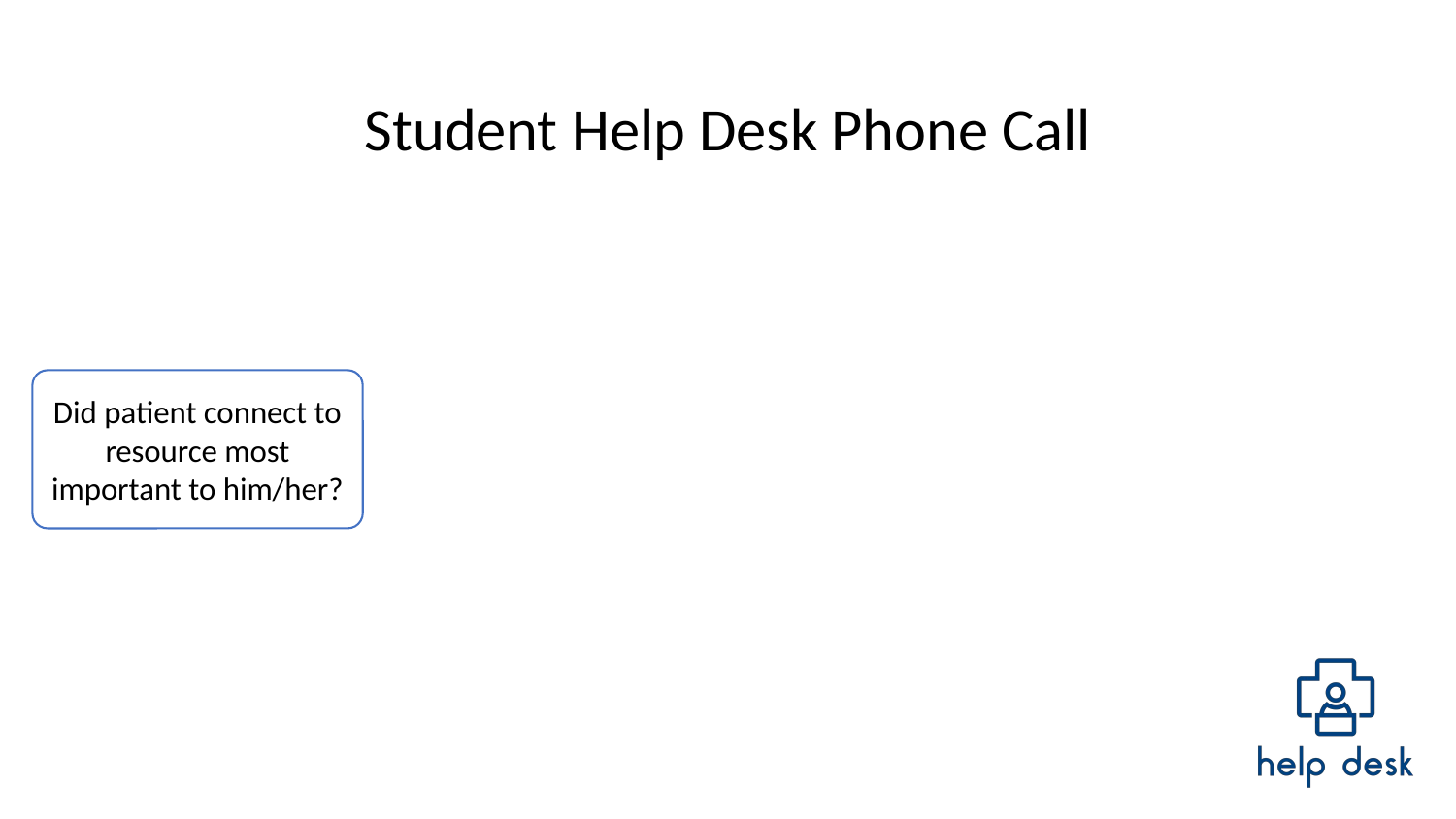

Student Help Desk Phone Call
Did patient connect to resource most important to him/her?

## Slide 54
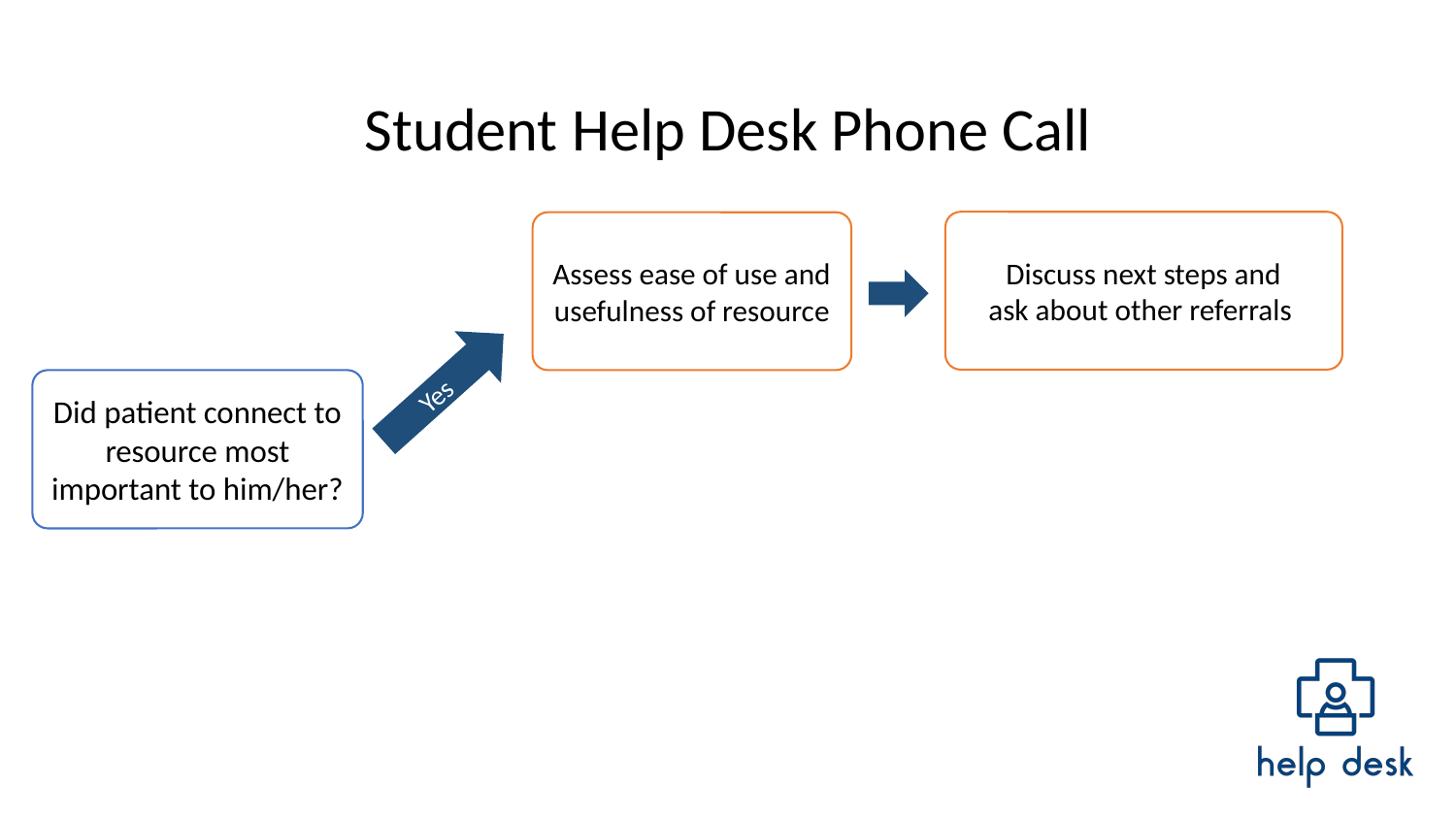

Student Help Desk Phone Call
Discuss next steps and
ask about other referrals
Assess ease of use and usefulness of resource
Yes
Did patient connect to resource most important to him/her?

## Slide 55
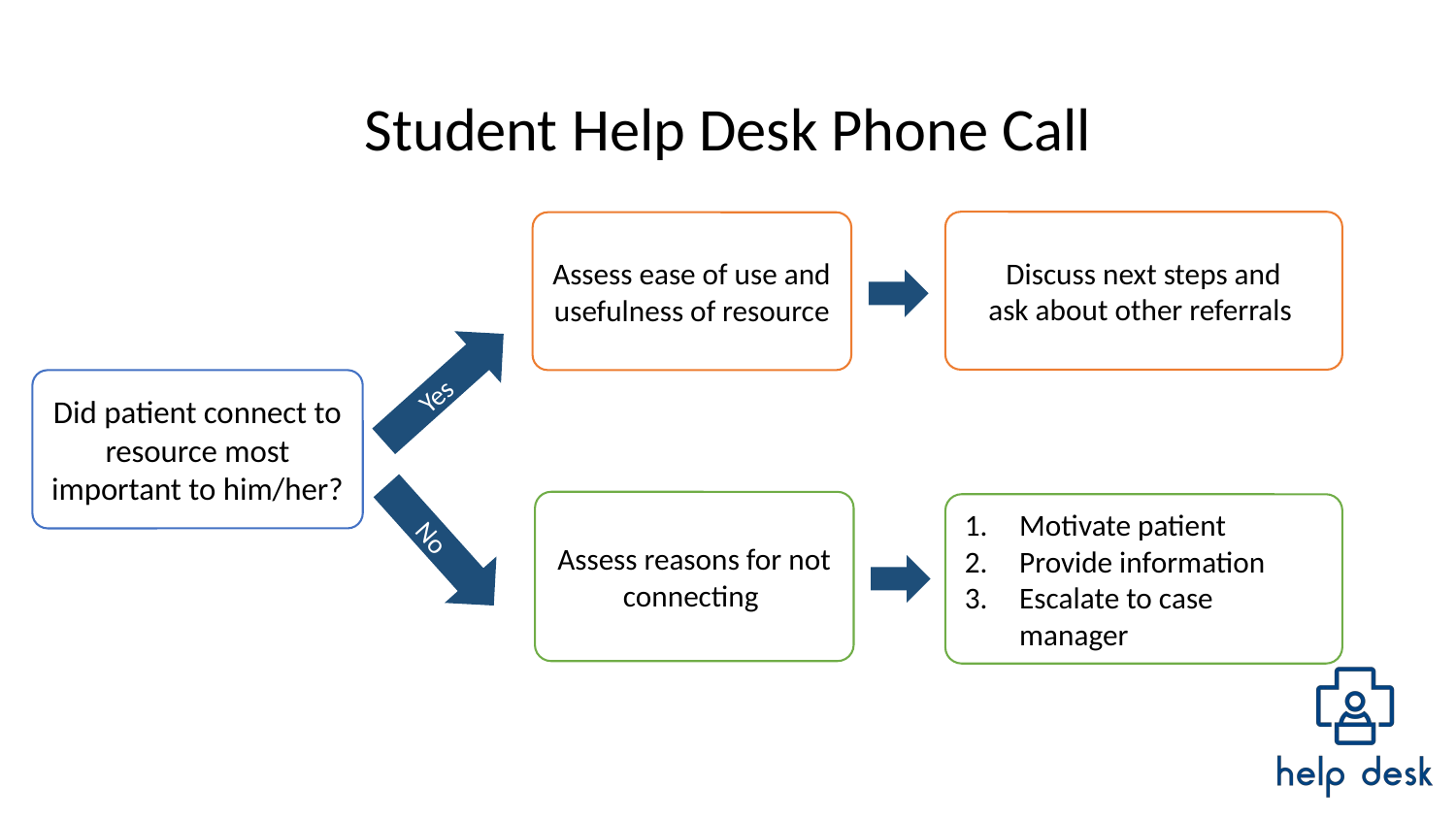

Student Help Desk Phone Call
Discuss next steps and
ask about other referrals
Assess ease of use and usefulness of resource
Yes
Did patient connect to resource most important to him/her?
Assess reasons for not connecting
Motivate patient
Provide information
Escalate to case manager
No

## Slide 56
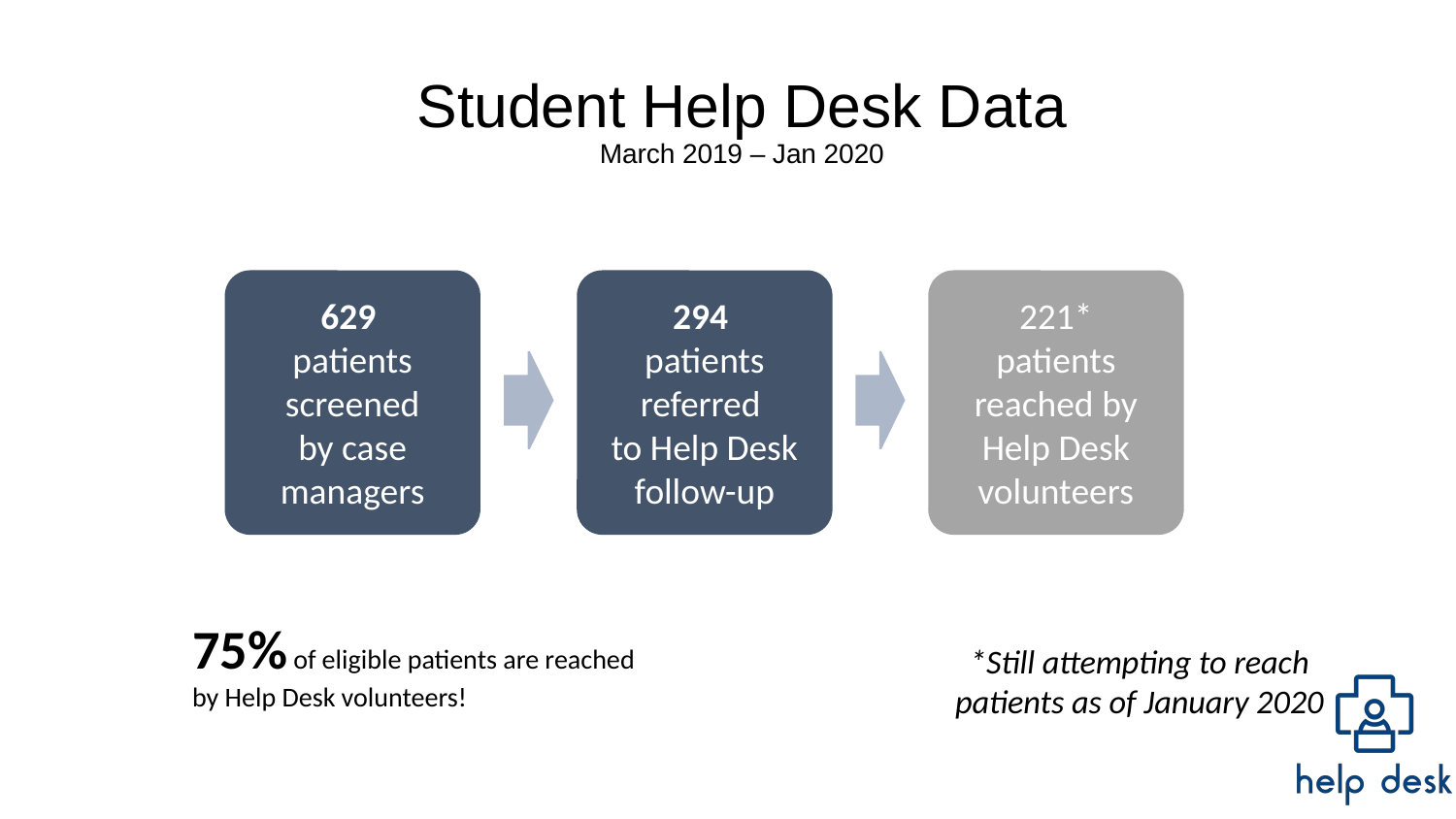

Student Help Desk Data
March 2019 – Jan 2020
629
patients screened
by case managers
294
patients referred
to Help Desk follow-up
221*
patients reached by Help Desk volunteers
75% of eligible patients are reached by Help Desk volunteers!
*Still attempting to reach patients as of January 2020

## Slide 57
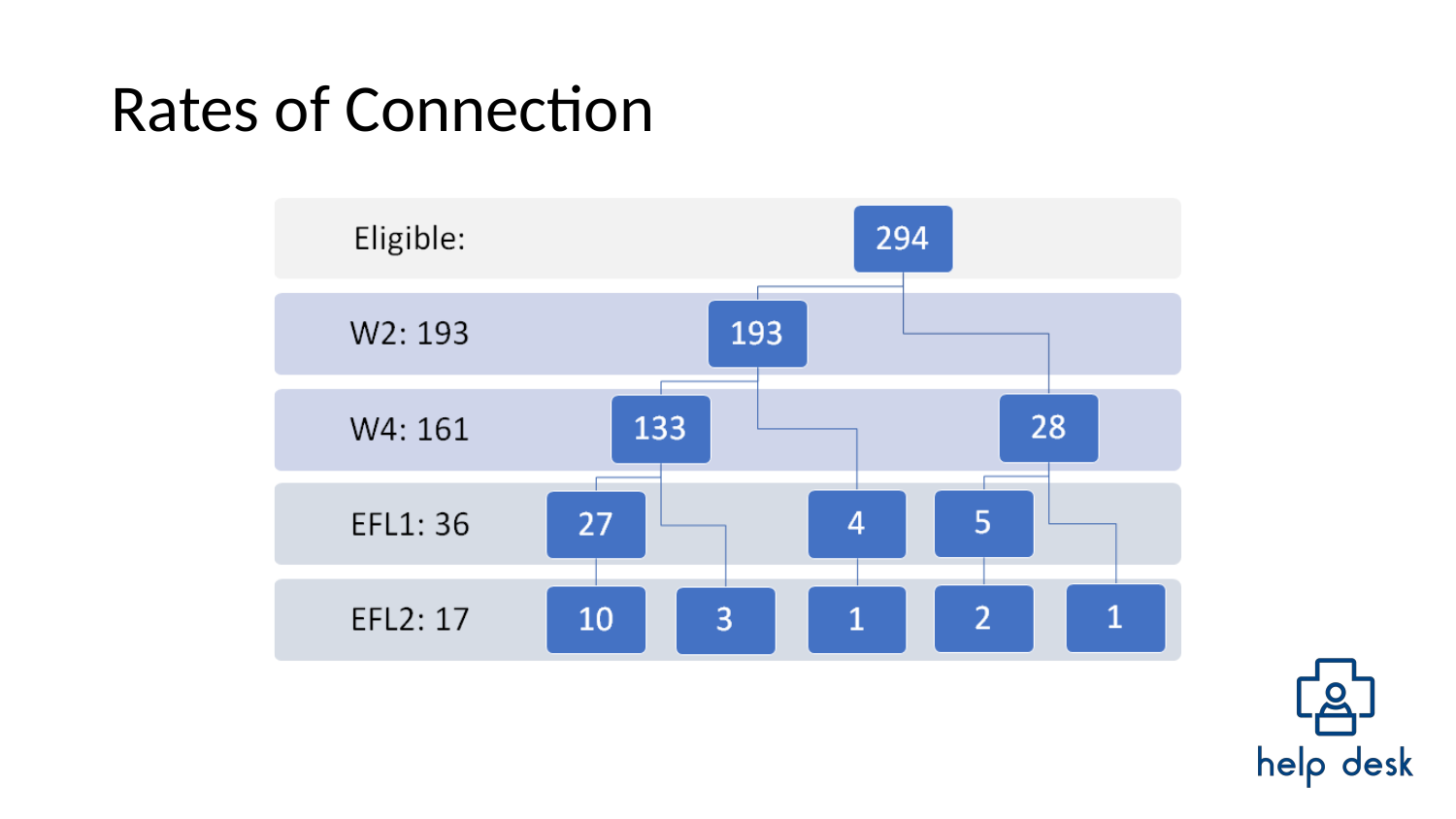

# Rates of Connection

## Slide 58
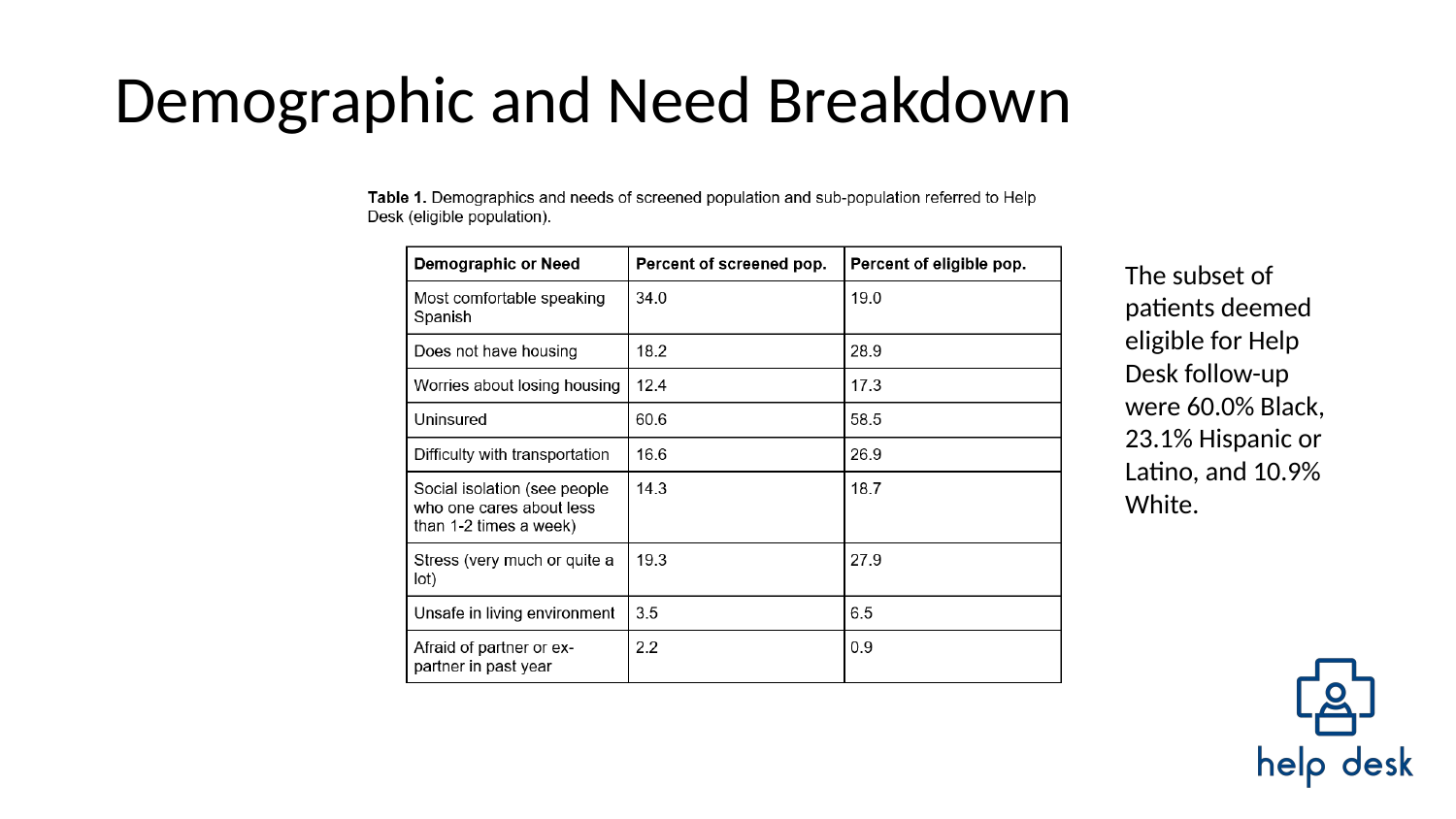

# Demographic and Need Breakdown
The subset of patients deemed eligible for Help Desk follow-up were 60.0% Black, 23.1% Hispanic or Latino, and 10.9% White.

## Slide 59
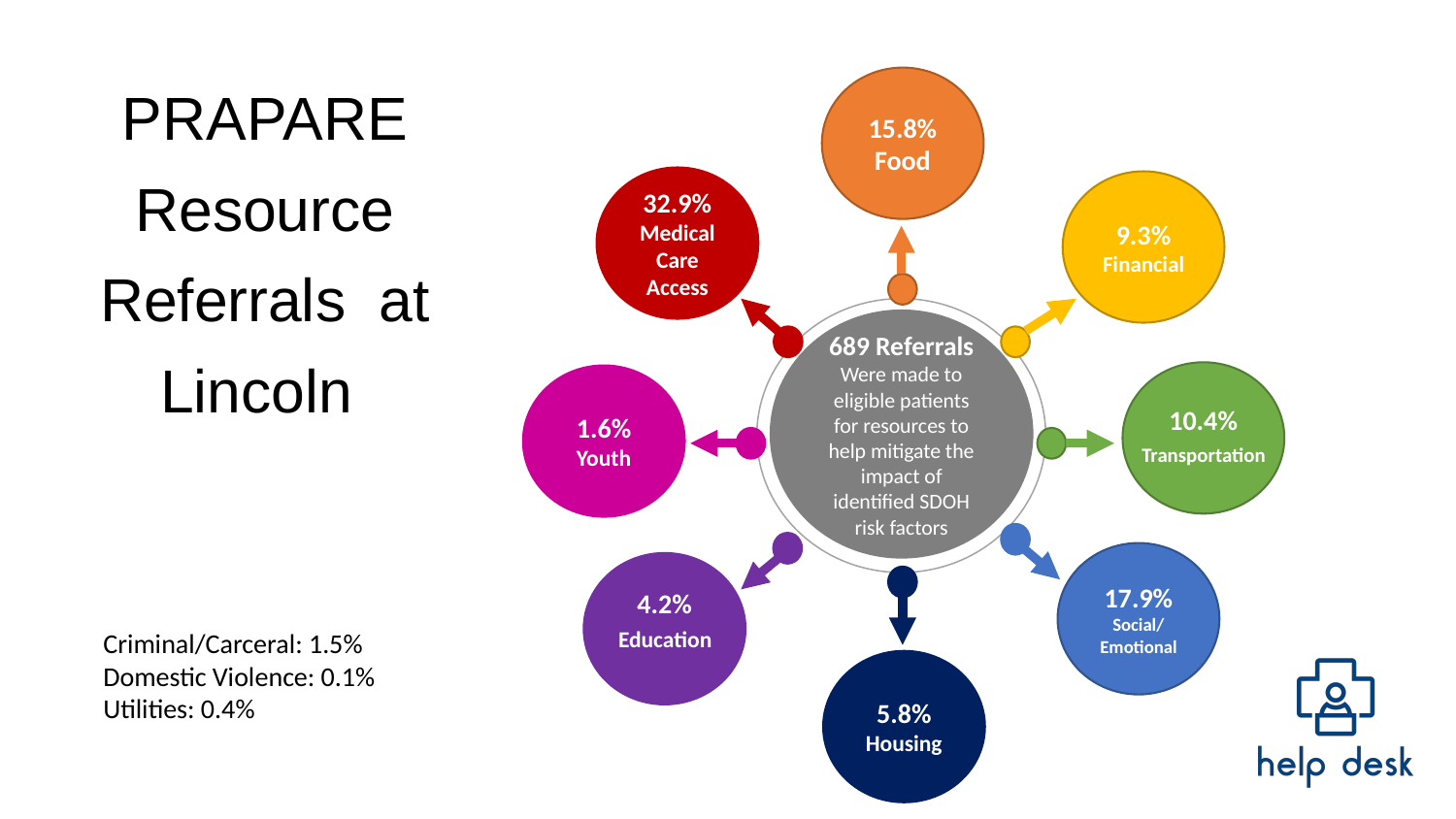

PRAPARE
Resource Referrals at Lincoln
15.8% Food
32.9% Medical Care Access
9.3% Financial
689 Referrals
Were made to eligible patients for resources to help mitigate the impact of identified SDOH risk factors
10.4%
1.6% Youth
Transportation
17.9% Social/
Emotional
4.2%
Education
Criminal/Carceral: 1.5%
Domestic Violence: 0.1%
Utilities: 0.4%
5.8% Housing

## Slide 60
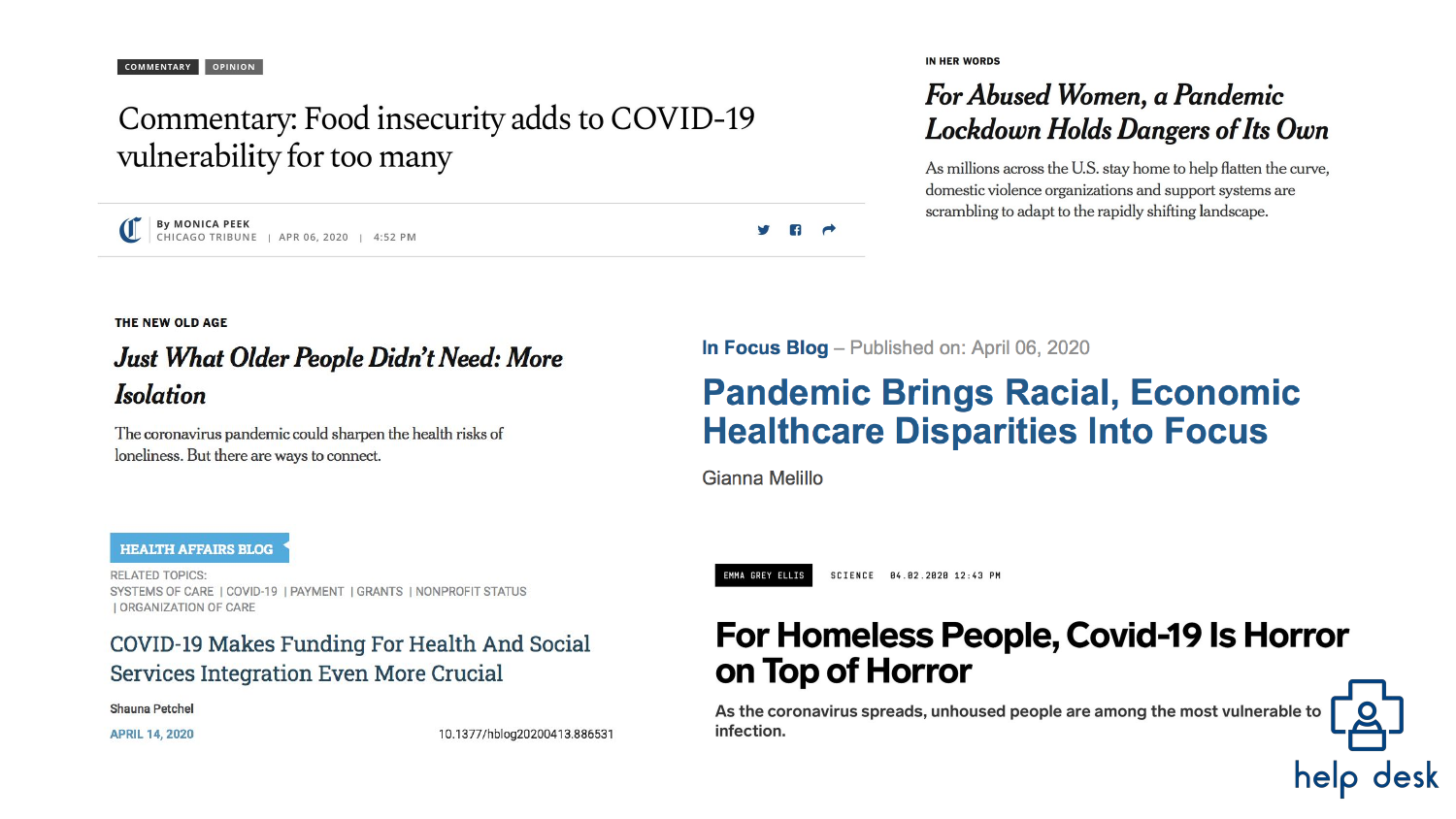

## Slide 61
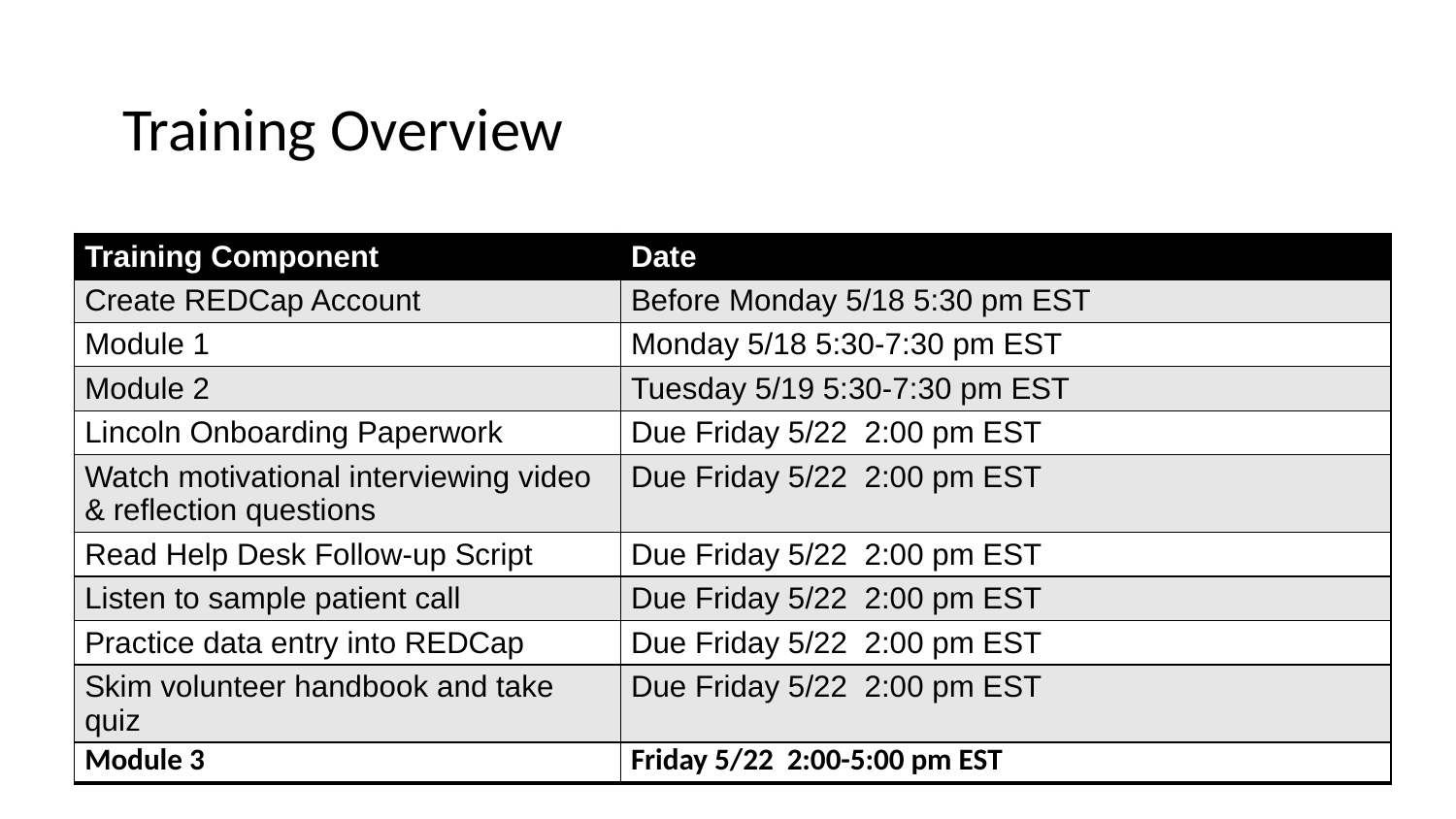

# Training Overview
| Training Component | Date |
| --- | --- |
| Create REDCap Account | Before Monday 5/18 5:30 pm EST |
| Module 1 | Monday 5/18 5:30-7:30 pm EST |
| Module 2 | Tuesday 5/19 5:30-7:30 pm EST |
| Lincoln Onboarding Paperwork | Due Friday 5/22 2:00 pm EST |
| Watch motivational interviewing video & reflection questions | Due Friday 5/22 2:00 pm EST |
| Read Help Desk Follow-up Script | Due Friday 5/22 2:00 pm EST |
| Listen to sample patient call | Due Friday 5/22 2:00 pm EST |
| Practice data entry into REDCap | Due Friday 5/22 2:00 pm EST |
| Skim volunteer handbook and take quiz | Due Friday 5/22 2:00 pm EST |
| Module 3 | Friday 5/22 2:00-5:00 pm EST |

## Slide 62
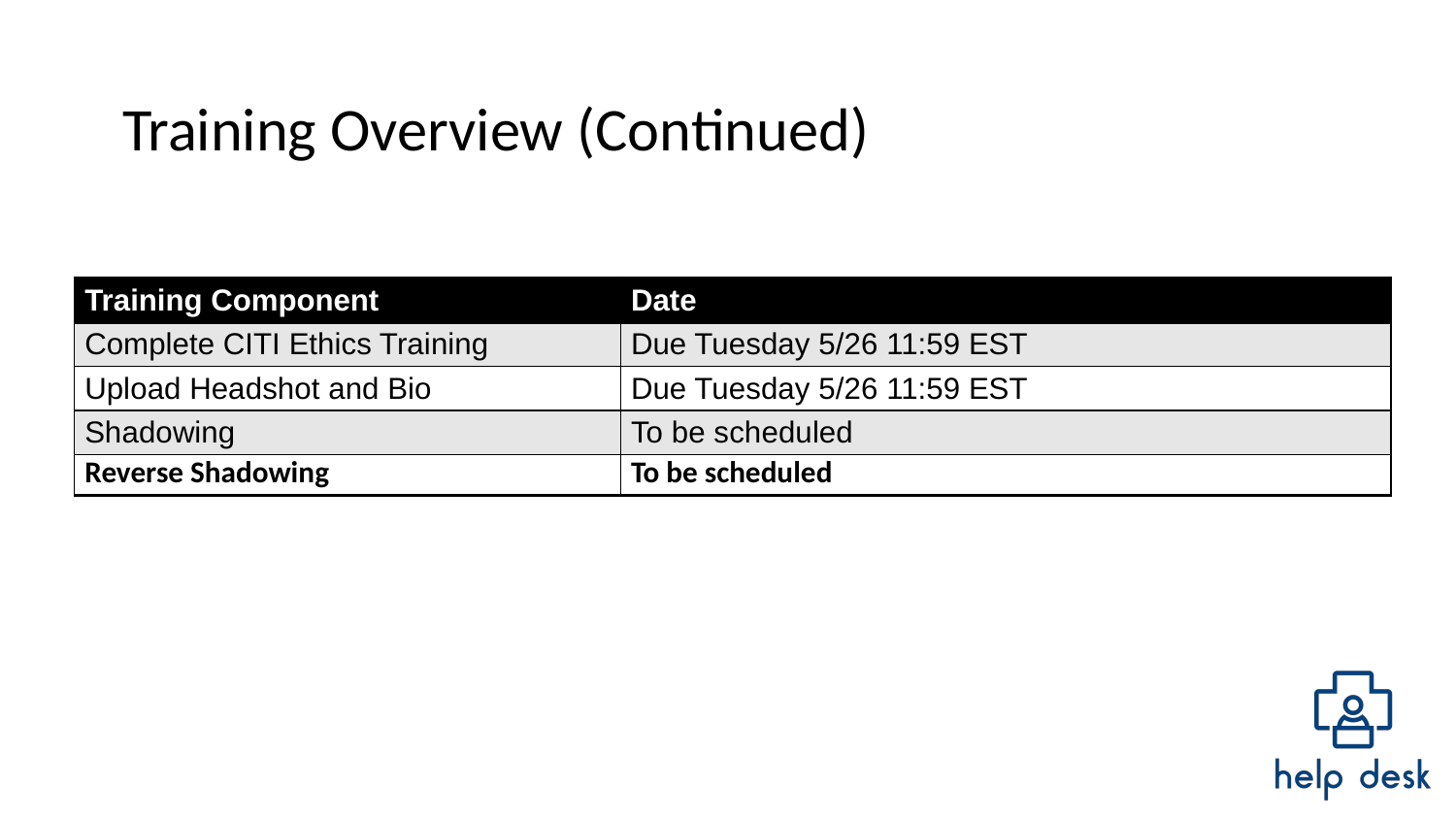

# Training Overview (Continued)
| Training Component | Date |
| --- | --- |
| Complete CITI Ethics Training | Due Tuesday 5/26 11:59 EST |
| Upload Headshot and Bio | Due Tuesday 5/26 11:59 EST |
| Shadowing | To be scheduled |
| Reverse Shadowing | To be scheduled |
