# Supplementary material for: Training student volunteers as community resource navigators to address patients' social needs: A curriculum toolkit
Source: Front Public Health. 2022 Sep 20;10:966872. doi: 10.3389/fpubh.2022.966872 (PMC9531674; doi:10.3389/fpubh.2022.966872)
Supplement: Supplementary file 1 [file Data_Sheet_1.zip › Data Sheet 6.docx]

**Durham Neighborhood Compass Activity**

**Disclaimer: This activity was designed for Durham North Carolina, using local data and resources. When replicating to a clinical site outside of Durham, the general format of the activity can be followed, but specific resources and data should be adapted to fit the local community.**

**Participant Instruction**

The goal of this activity is to learn more about Durham. Volunteer trainees will be split up into breakout rooms.

1. Introduce yourself to your group!

Group 1: [List names here]

Group 2: [List names here]

Group 3: [List names here]

Group 4: [List names here]

1. Designate a group note taker who can report back what they discussed to the broader team.
2. Take a moment to read the “Background on Durham, North Carolina” below and answer the guiding questions as a group.

**PART 1 (5 minutes)**

**Background on Durham, North Carolina**

*From: L E Boulware, G B Harris, P Harewood, F F Johnson, P Maxson, N Bhavsar, S S Blackwelder, S S Poley, K Arnold, B Akindele, J Ferranti, M Lyn, Democratizing health system data to impact social and environmental health contexts: a novel collaborative community data-sharing model, Journal of Public Health.*

- North Carolina ranks 36^th^ for overall health nationally and 38^th^ for premature death.
- Within North Carolina, Durham County is the sixth largest (312,000 residents) county.
- The County is ethnically and racially diverse (14% Hispanic/Latino, 38% Black, 42% White and 6% other races/ethnicities) and socioeconomically diverse (16% below poverty line, a median income of $54,000 and 20% above 100,000 annual income).
- Unlike most counties in North Carolina, Durham County is comprised primarily of a single city, Durham, which is home to a diverse economy featuring academic centers, health care, technology and a number of other sectors.
- Durham fares worse than the state overall on a number of health indicators, such as child mortality, percent uninsured, health risk from air pollutants, food insecurity, and consumption of fruits and vegetables.
- Durham ranks 38th among North Carolina’s 100 counties for social and economic factors, including a homicide rate almost twice that of the state.
- Durham also has health inequities (e.g. rate of low birth weight being 7% for Whites and 13% for Blacks).
- A significant proportion (7%) of Durham’s children are uninsured, and 24% of households with children have incomes below the poverty level. Within these households, 65% are enrolled in Medicaid and more than half receive food assistance through the Supplemental Nutrition Assistance Program or the Supplemental Nutrition Program for Women, Infants and Children. In 2018, 59% of children in Durham County qualified for free or reduced lunch.

**Guiding questions**

- How do these statistics relate to your own knowledge and personal experiences living in Durham?
- Do any of these statistics surprise you? Why?
- What are some limitations of this data?
- What do you think might be some of the reasons that explain Durham’s health indicators relative to the state? What about the health and social inequities that exist within the county?

**PART 2 (7 minutes)**

**Background on Durham Neighborhood Compass**

- Durham city and County stakeholders have developed the Durham Neighborhood Compass, a website designed to openly share data on community characteristics with Durham residents.
- The Durham Neighborhood Compass surfaces data on a number of community characteristics with relevance to health outcomes including data on educational attainment, income distribution, housing prices, crime rates, travel routes, school quality, grocery store proximity, etc., all at the neighborhood level.
- Data aggregated from locally generated data from government operations, data from commonly available census and other state/federal sources, and health data from Duke Health and Lincoln Community Health Center. The table below from the Boulware article shows a sampling of where data for the Durham Neighborhood Compass comes from.

**
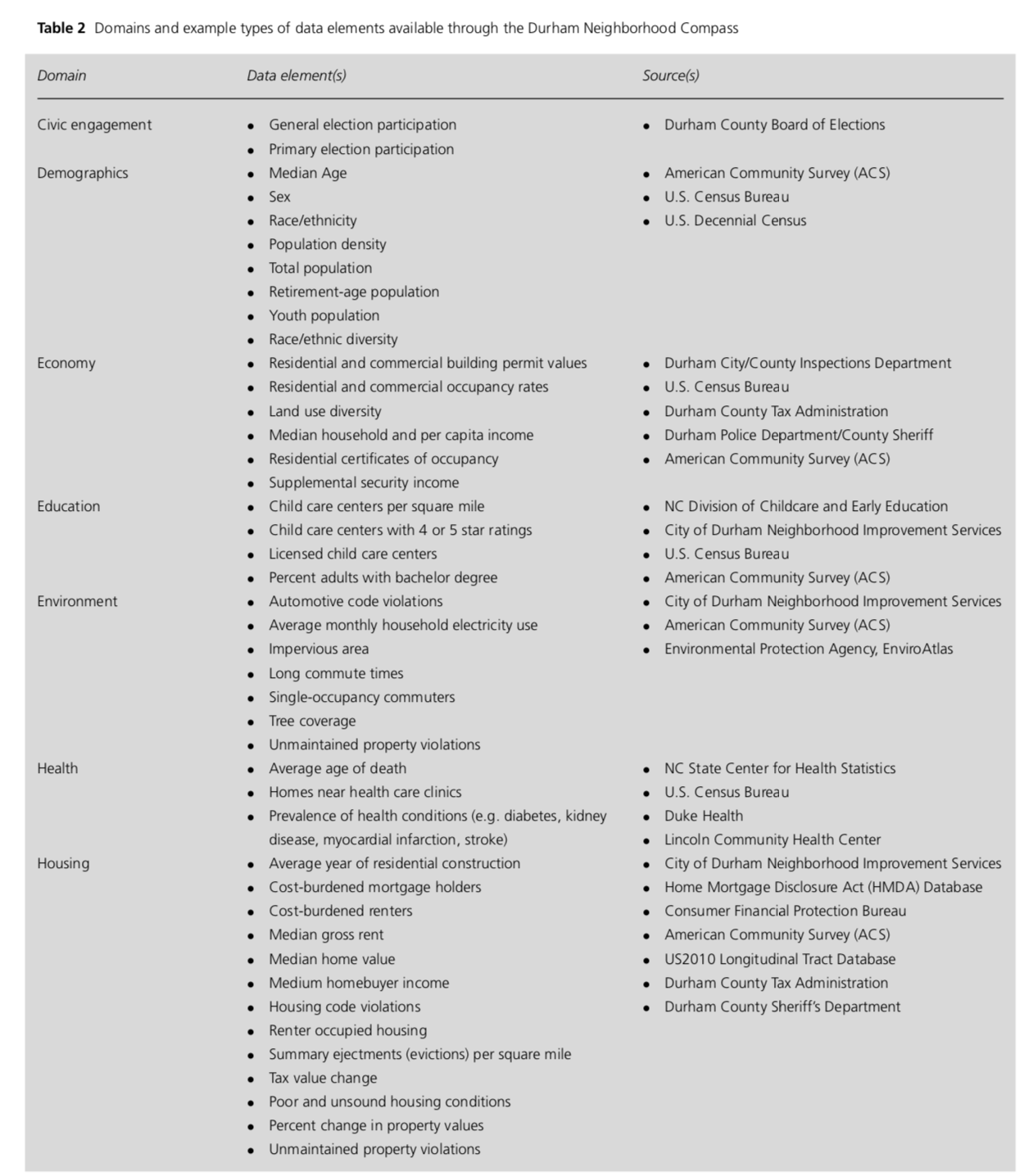
**

**Now it’s your turn to use the Durham Neighborhood Compass!**

1. Open the link: <https://compass.durhamnc.gov/en> (**link relevant to local community should be used**)
2. Take a few minutes to explore the website
   1. Health Data
      1. Click the “Health” tab
      2. Choose a measure of interest (e.g. average age of death, chronic kidney disease, diabetes, etc.). Use your “mouse” to hover over different census block groups
      3. Guiding question:
         1. **What trends and variation by geography do you see in the measures you selected?**
   2. Social Data
      1. Click “Economy” or “Housing” or “Infrastructure and Amenities”
      2. Choose a measure of interest
      3. Guiding question:
         1. **What trends and variation do you see in the measures you selected? How do they compare to trends in the “health” measures from the last step of the activity?**
         2. **How might different stakeholders use this data? For example, you might consider County Health Department, health centers, non-profits, policymakers, researchers)**

**Part 3: Durham Community Health Indicators Projects (7 minutes)**

A complementary website is the Durham Community Health Indicators Project: <https://health.dataworks-nc.org/en/about> (**local datasite should be used if applicable**)

This project is a partnership of Durham County Public Health, Lincoln Community Health, and Duke's Center for Community and Population Health Improvement. DataWorks receives health information from the Public Health Department for use in the Neighborhood Compass. We have developed this site as another way to learn about what influences health in our neighborhoods.

**Step 1: Open the Website** - <https://health.dataworks-nc.org/en/about> (**local datasite should be used if applicable**)

**Step 2: Press “locations” in the right-hand corner and search the location of your assigned group (**Clinical sites outside of Durham, NC should adjust the locations as needed.**)**

Group 1:

1301 Fayetteville St, Durham, NC 27707

(This is where Lincoln Community Health Center is located)

Group 2:

401 N. Driver Street, Durham, NC 27703

(This is where Lincoln Community Health Center’s satellite clinic Holton Wellness Center is located)

Group 3:

1313 Halley Street, Suite 137, Durham, NC 27707

(This is where Lincoln Community Health Center’s satellite clinic Lyon Park is located)

Group 4:

Watts-Hillandale, NC School of Science and Math

(This is close to East Campus)

**Step 3: One person in the group should share their screen and have the indicators site and this tables on this page side by side as shown below:**

**
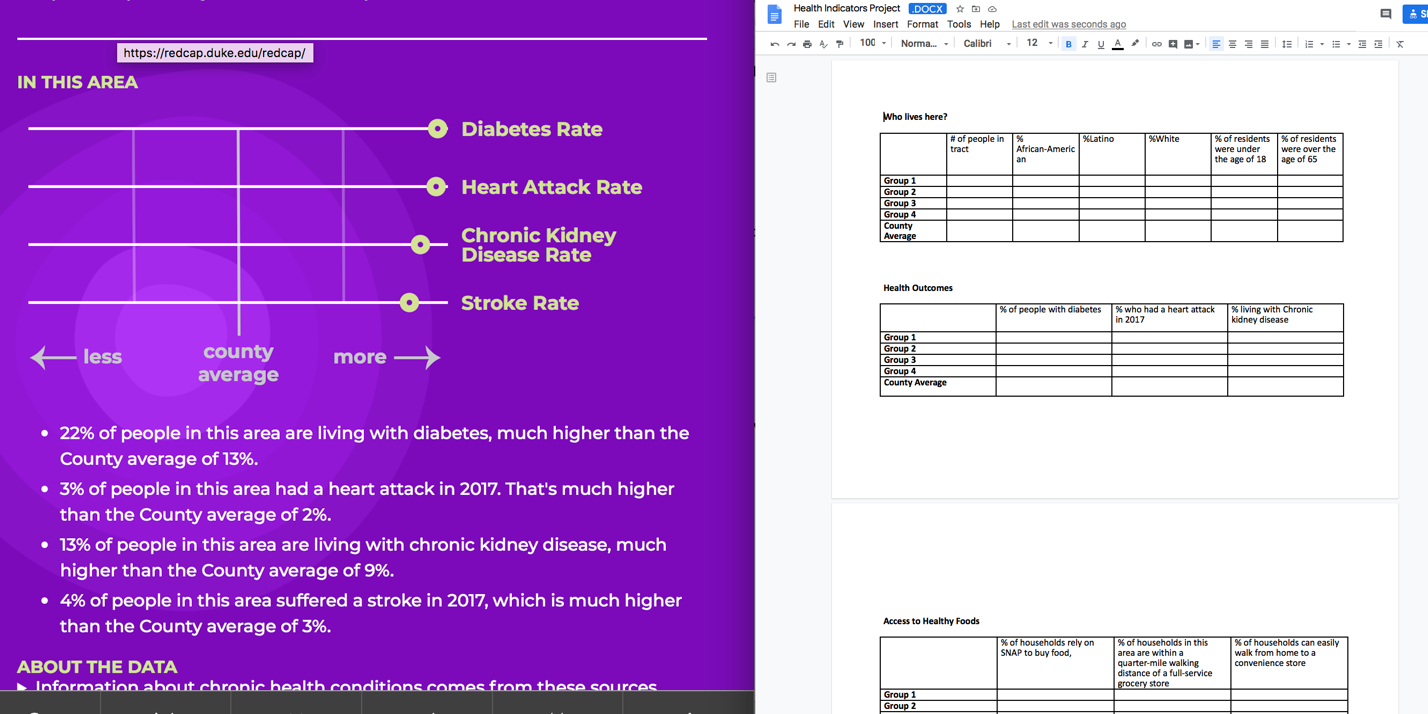
**

**Step 4: Your group should complete the table on the next page together. Throughout the process, hypothesize mechanisms in which various social factors could influence a person’s health. Make notes on what’s surprising, some common trends, etc. What assets do you have in your neighborhoods?**

**Who lives here?**

|  | # of people in tract | % African-American | %Latino | %White | % of residents were under the age of 18 | % of residents were over the age of 65 |
| --- | --- | --- | --- | --- | --- | --- |
| **Group 1** |  |  |  |  |  |  |
| **Group 2** |  |  |  |  |  |  |
| **Group 3** |  |  |  |  |  |  |
| **Group 4** |  |  |  |  |  |  |
| **County Average** |  |  |  |  |  |  |

**Health Outcomes**

|  | % of people with diabetes | % who had a heart attack in 2017 | % living with Chronic kidney disease |
| --- | --- | --- | --- |
| **Group 1** |  |  |  |
| **Group 2** |  |  |  |
| **Group 3** |  |  |  |
| **Group 4** |  |  |  |
| **County Average** |  |  |  |

**Access to Healthy Foods**

|  | % of households rely on SNAP to buy food | % of households in this area are within a quarter-mile walking distance of a full-service grocery store | % of households can easily walk from home to a convenience store |
| --- | --- | --- | --- |
| **Group 1** |  |  |  |
| **Group 2** |  |  |  |
| **Group 3** |  |  |  |
| **Group 4** |  |  |  |
| **County Average** |  |  |  |

**Access to Physical Activity**

|  | % of households in this area live near a park or rec center | Traffic exposure | % of roads in this area have sidewalks |
| --- | --- | --- | --- |
| **Group 1** |  |  |  |
| **Group 2** |  |  |  |
| **Group 3** |  |  |  |
| **Group 4** |  |  |  |
| **County Average** |  |  |  |

**Environmental stress factors**

|  | % Cost burdened residents | # of drug arrests per square mile | # of eviction notices per square mile | # of households are located near sources of air pollution |
| --- | --- | --- | --- | --- |
| **Group 1** |  |  |  |  |
| **Group 2** |  |  |  |  |
| **Group 3** |  |  |  |  |
| **Group 4** |  |  |  |  |
| **County Average** |  |  |  |  |

**Class**

|  | % of residents are working in service industry jobs | % of residents are living in poverty |
| --- | --- | --- |
| **Group 1** |  |  |
| **Group 2** |  |  |
| **Group 3** |  |  |
| **Group 4** |  |  |
| **County Average** |  |  |
