# Supplementary material for: Training student volunteers as community resource navigators to address patients' social needs: A curriculum toolkit
Source: Front Public Health. 2022 Sep 20;10:966872. doi: 10.3389/fpubh.2022.966872 (PMC9531674; doi:10.3389/fpubh.2022.966872)
Supplement: Supplementary file 1 [file Data_Sheet_1.zip › Data Sheet 8.docx]

**Deep Dive into the Common Needs and Resources**

**Disclaimer: These resources are specific to clinic sites at Durham, North Carolina. Other clinical sites adapting this model should use this model as a template and include resources relevant to that site. However, general format of the activity is replicable**

**Breakout groups:**

Group 1: Participant names

Group 2: Participant names

Group 3: Participant names

Group 4: Participant names

**Activity 1: Directory walkthrough (5-7 minutes)**

- Goal: Familiarize yourself with the format of the directory.
- The links below take you to the directory. Each tab on the bottom is a different need domain. This directory is constantly updated by a team of 20-30 volunteers so the most recent information is reflected.
- Links to the directory:
  - English: **Insert link to directory here**
  - Spanish: **Insert link to directory here**
- As you explore think about:
  - What is one thing you like in the format of the directory?
  - What is one thing that could be improved?

**Activity 2: Deep dive into the resources**

- General flow of the activity**:**
  - Learn about the need domain→ research assigned resource → Report out to the whole group→ discuss with experienced volunteers→ learn about a new need domain..

- Goal: Learn more about the commonly referred resources.

- Description: For each need domain, four commonly referred resources are identified. Each breakout group will research one assigned resource using the directory or any other sources and report out to the whole group.
- In breakout rooms, each person should pull up the resource directory on their computer. One person may consider sharing their screen. Take 5-6 minutes to find your resource in the directory and be prepared to report on the following to the whole group:
  - How does this resource specifically address a patient’s need (e.g. what services are provided)
  - Which populations do they serve?
  - Best way to access the resource
  - Languages
  - What additional info would you like to know about this resource?

**We will repeat this activity for all 6 need domains**:

Food, Housing, Medical Care, Finances, Behavioral and Mental Health, and Transportation.

Below are the assigned resources for each group.

**Food Insecurity:**

| Food Pantries Handout (on directory look for Durham Area Food Resources Locator) | Participant names |
| --- | --- |
| SNAP | Participant names |
| More in my Basket | Participant names |
| Durham FEAST (both pick-up and delivery) | Participant names |

**Housing:**

| Durham Rental Guide (see link below) | Participant names |
| --- | --- |
| Durham Housing Authority | Participant names |
| Catholic Charities of Durham | Participant names |
| Durham Rescue Mission | Participant names |

**Medical Care:**

| Senior PharmAssist (prescription drugs tab in the CBO directory and the medical care tab!) | Participant names |
| --- | --- |
| Project Access | Participant names |
| NC MedAssist | Participant names |
| Lincoln Medication Voucher (prescription drugs tab in the CBO directory) | Participant names |

**Finances:**

| Unemployment Benefits | Participant names |
| --- | --- |
| Durham Department of Social Services (rent/utilities assistance) from handout | Participant names |
| Salvation Army (from handout) | Participant names |
| Urban Ministries (from handout) | Participant names |

**Behavioral and Mental Health:**

| Carolina Outreach | Participant names |
| --- | --- |
| Alliance Behavioral Health | Participant names |
| LCHC Behavioral Health | Participant names |
| Freedom House | Participant names |

**Transportation:**

| LCHC Transportation Slip | Participant names |
| --- | --- |
| Medicaid Van | Participant names |
| Go Durham Access Van | Participant names |
| Public Transportation (Durham Bus System) | Participant names |
