# Supplementary material for: Training student volunteers as community resource navigators to address patients' social needs: A curriculum toolkit
Source: Front Public Health. 2022 Sep 20;10:966872. doi: 10.3389/fpubh.2022.966872 (PMC9531674; doi:10.3389/fpubh.2022.966872)
Supplement: Supplementary file 1 [file Data_Sheet_1.zip › Data Sheet 7.PPTX]

## Slide 1
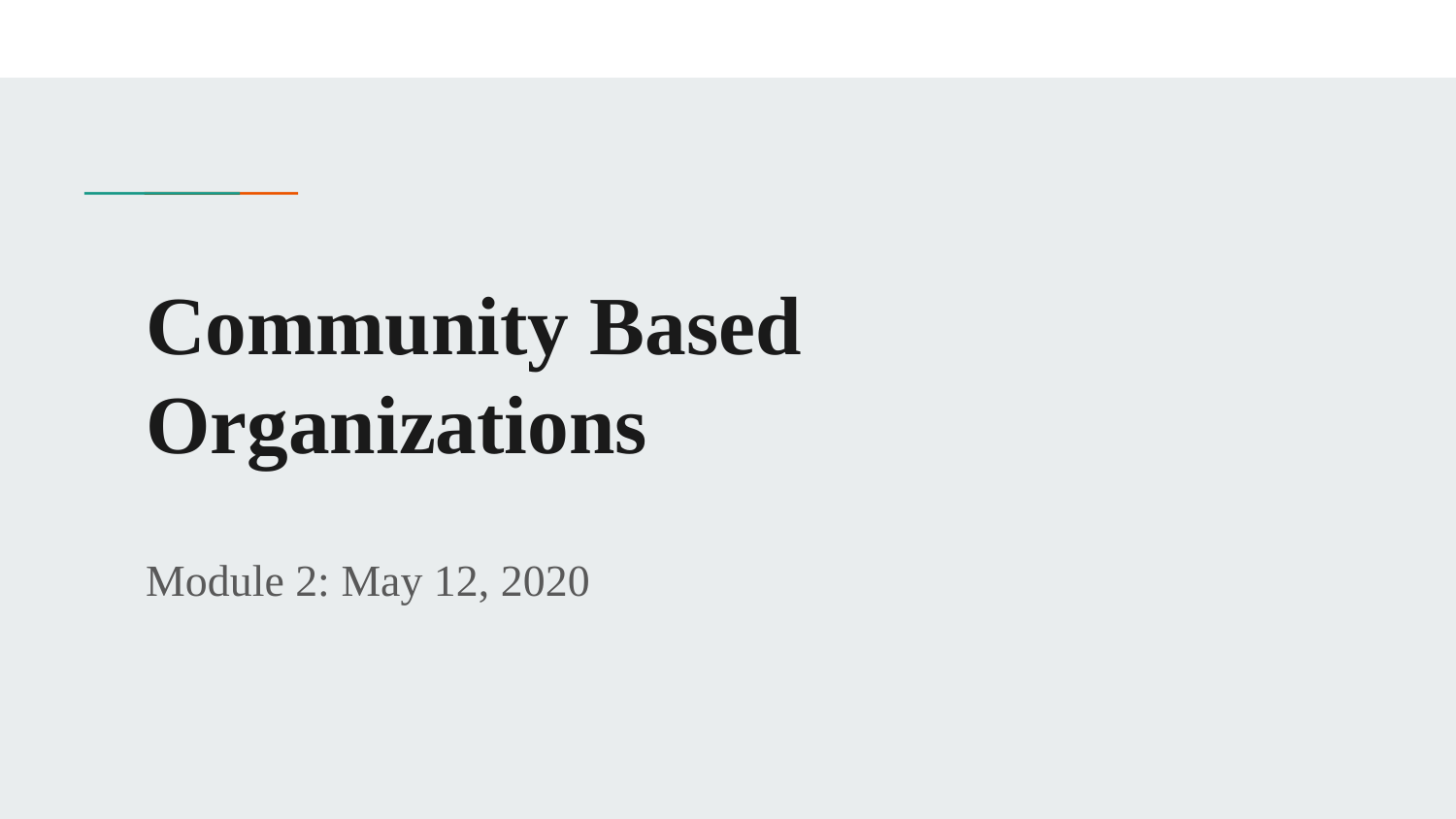

# Community Based Organizations
Module 2: May 12, 2020

## Slide 2
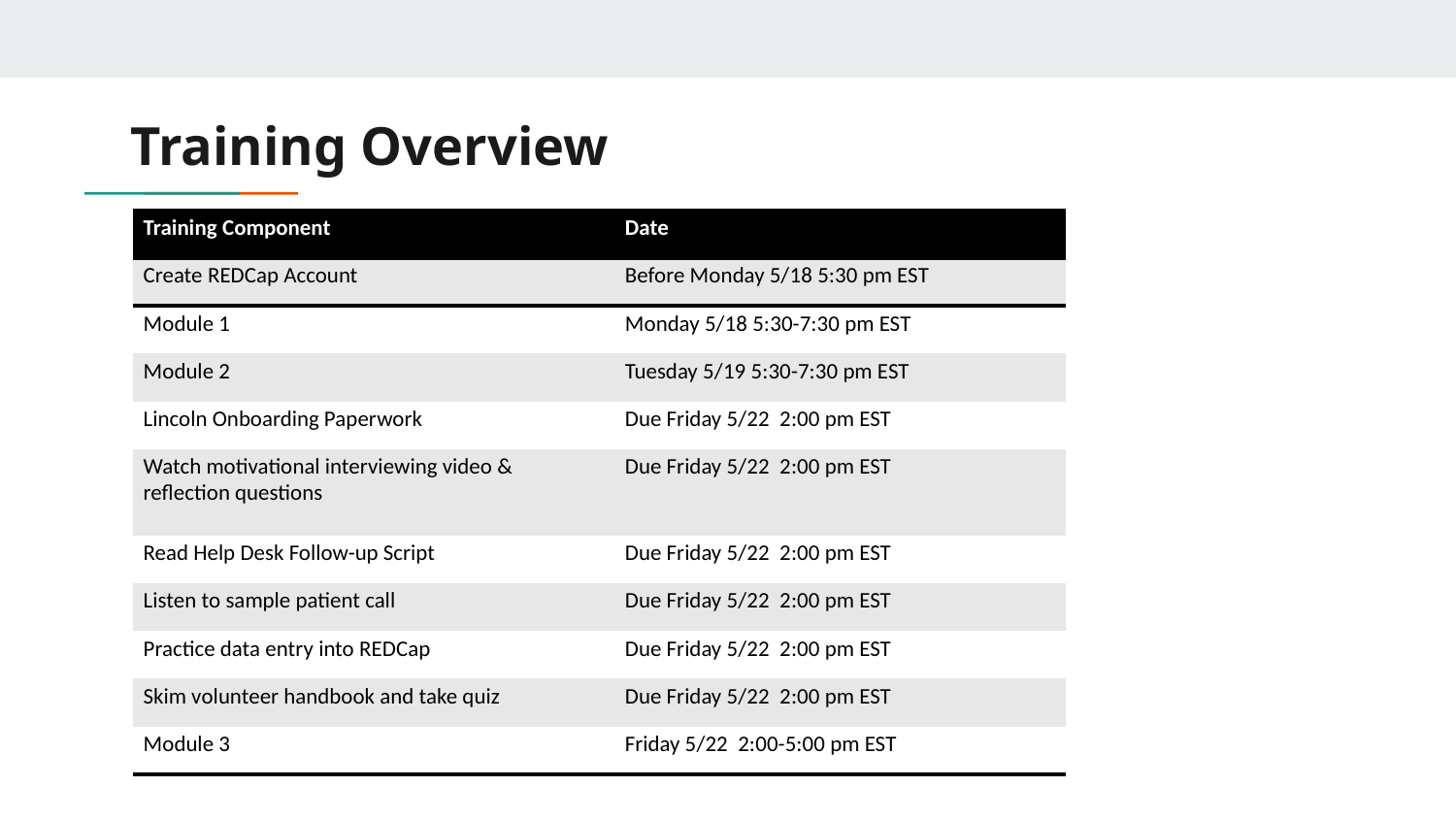

# Training Overview
| Training Component | Date |
| --- | --- |
| Create REDCap Account | Before Monday 5/18 5:30 pm EST |
| Module 1 | Monday 5/18 5:30-7:30 pm EST |
| Module 2 | Tuesday 5/19 5:30-7:30 pm EST |
| Lincoln Onboarding Paperwork | Due Friday 5/22 2:00 pm EST |
| Watch motivational interviewing video & reflection questions | Due Friday 5/22 2:00 pm EST |
| Read Help Desk Follow-up Script | Due Friday 5/22 2:00 pm EST |
| Listen to sample patient call | Due Friday 5/22 2:00 pm EST |
| Practice data entry into REDCap | Due Friday 5/22 2:00 pm EST |
| Skim volunteer handbook and take quiz | Due Friday 5/22 2:00 pm EST |
| Module 3 | Friday 5/22 2:00-5:00 pm EST |

## Slide 3
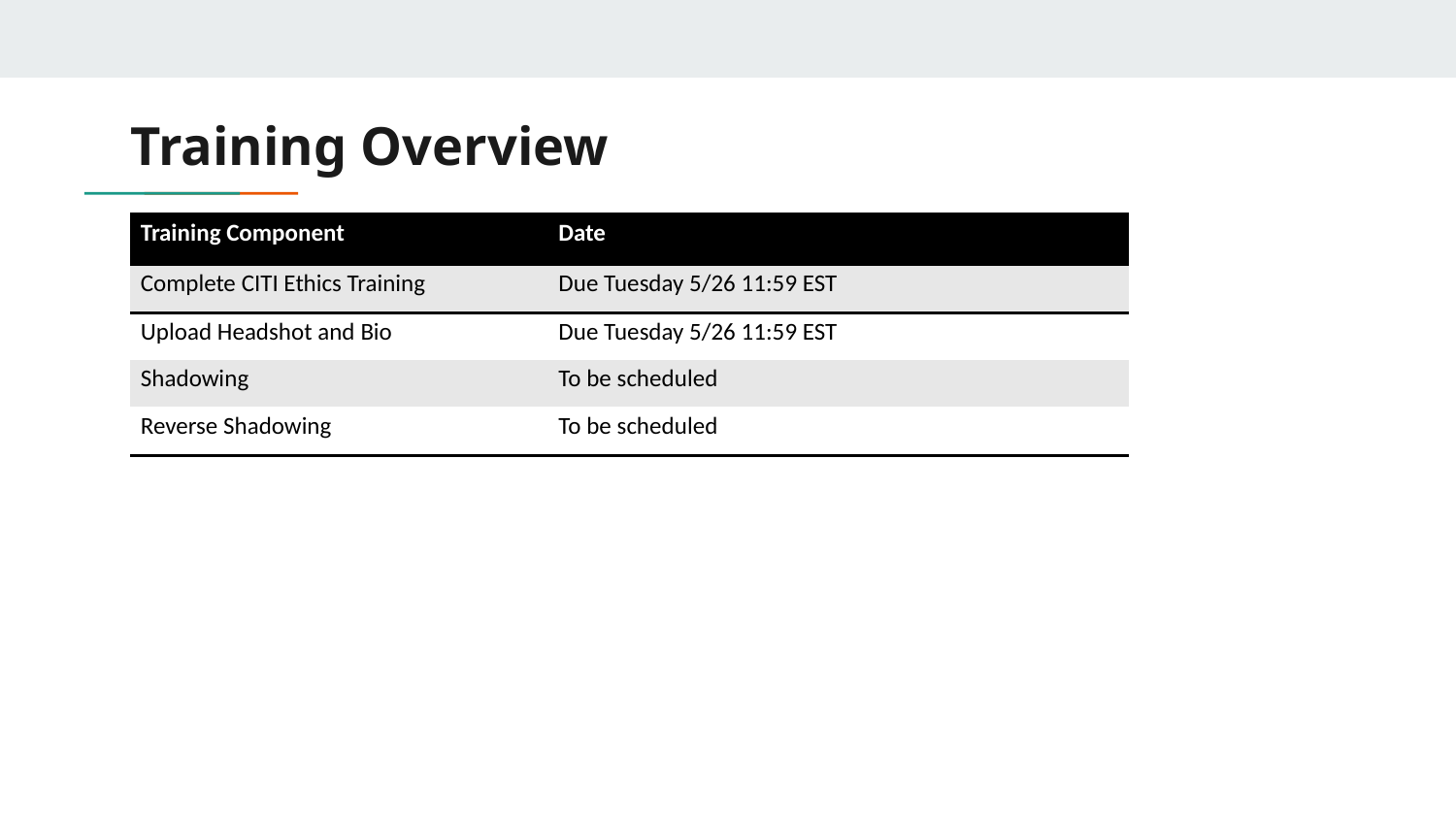

# Training Overview
| Training Component | Date |
| --- | --- |
| Complete CITI Ethics Training | Due Tuesday 5/26 11:59 EST |
| Upload Headshot and Bio | Due Tuesday 5/26 11:59 EST |
| Shadowing | To be scheduled |
| Reverse Shadowing | To be scheduled |

## Slide 4
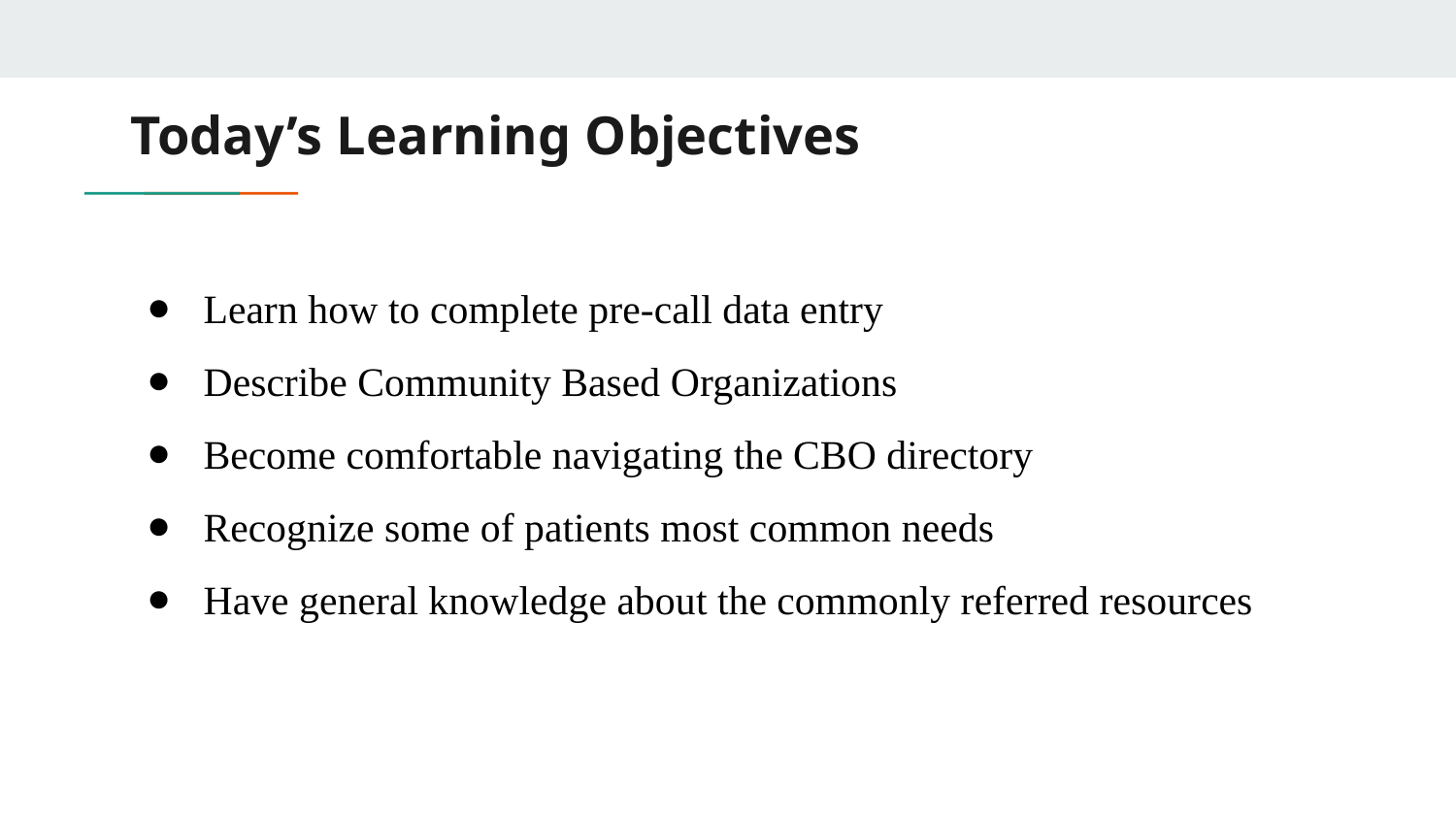

# Today’s Learning Objectives
Learn how to complete pre-call data entry
Describe Community Based Organizations
Become comfortable navigating the CBO directory
Recognize some of patients most common needs
Have general knowledge about the commonly referred resources

## Slide 5
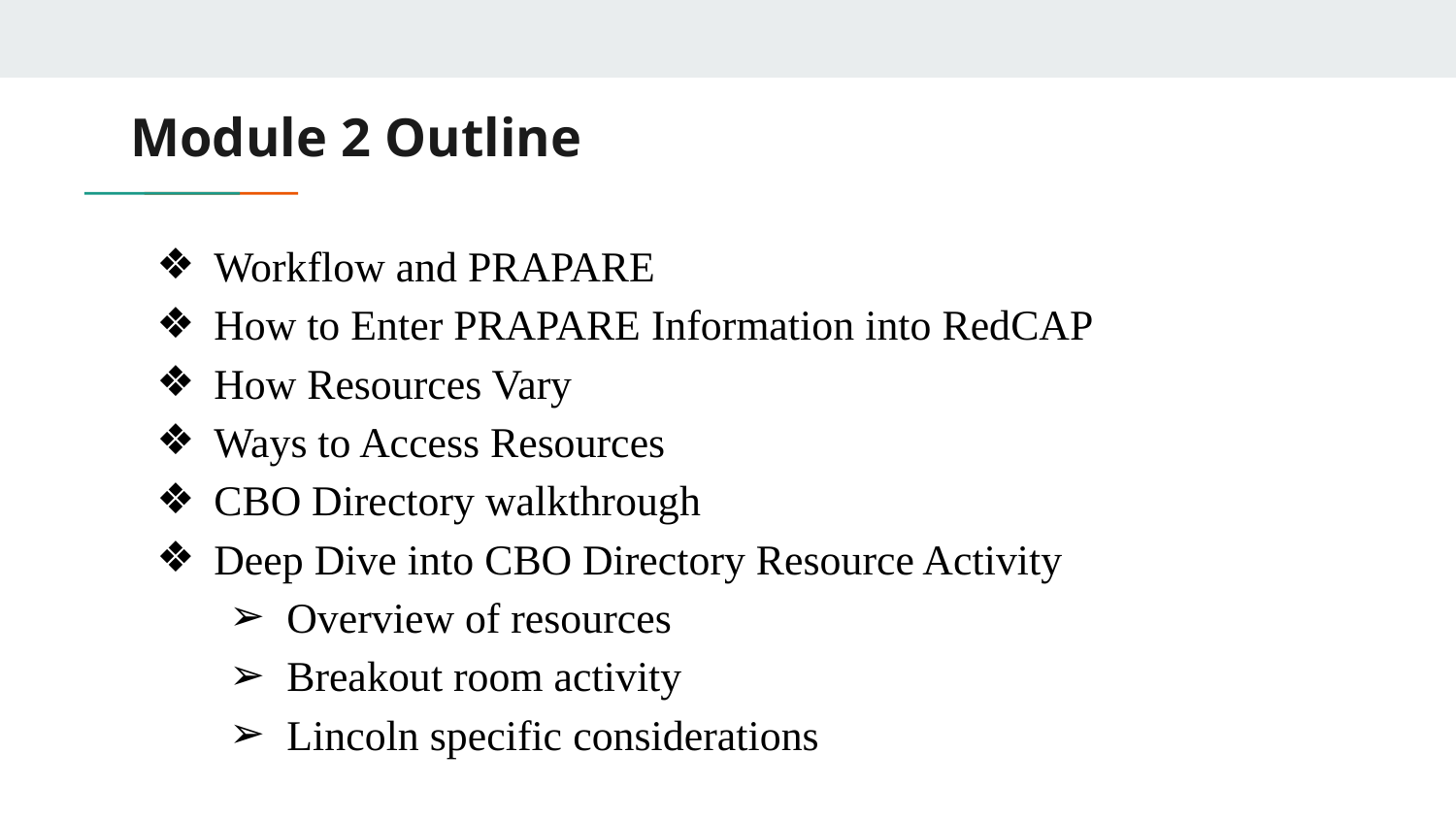

# Module 2 Outline
Workflow and PRAPARE
How to Enter PRAPARE Information into RedCAP
How Resources Vary
Ways to Access Resources
CBO Directory walkthrough
Deep Dive into CBO Directory Resource Activity
Overview of resources
Breakout room activity
Lincoln specific considerations

## Slide 6
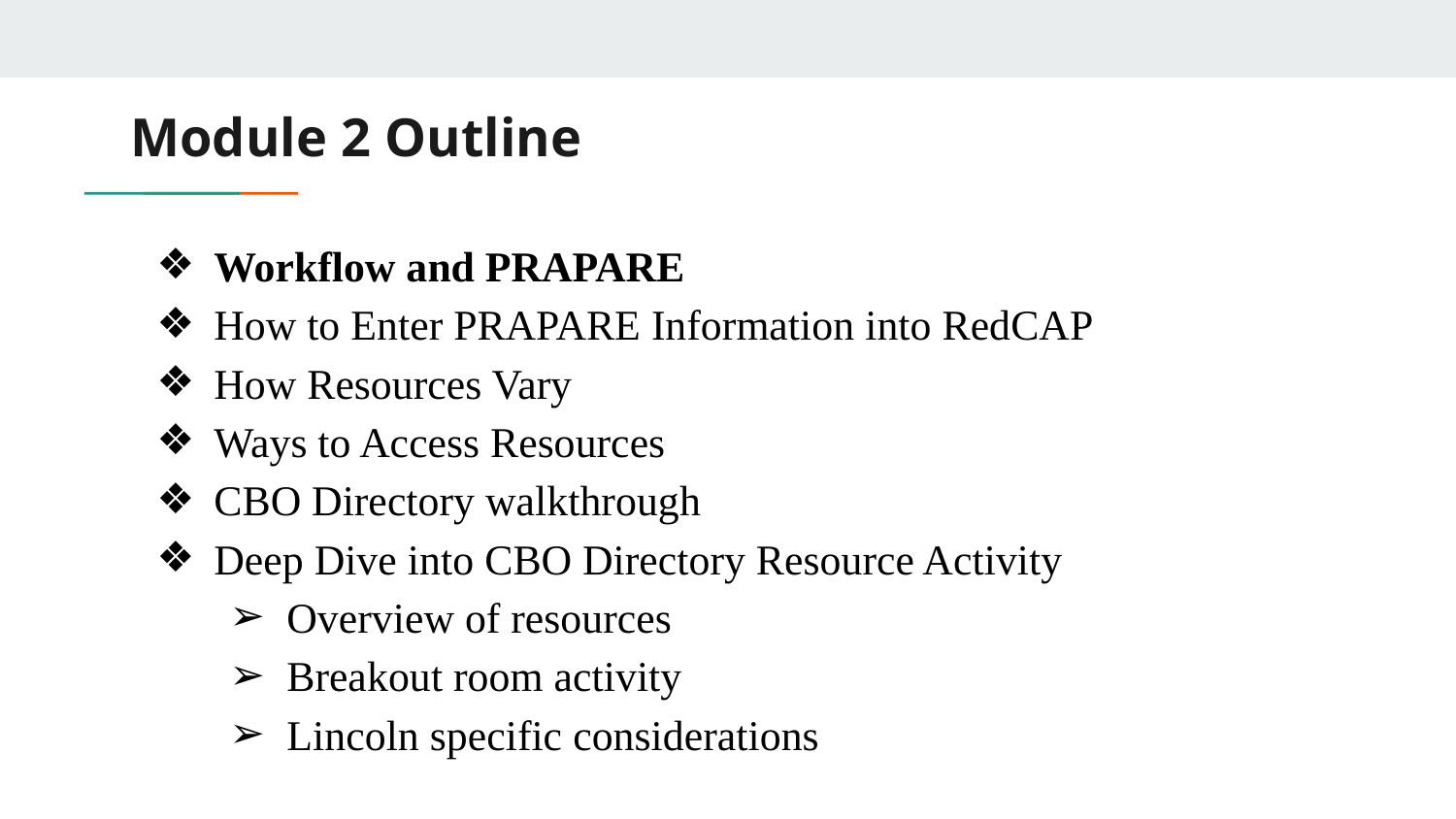

# Module 2 Outline
Workflow and PRAPARE
How to Enter PRAPARE Information into RedCAP
How Resources Vary
Ways to Access Resources
CBO Directory walkthrough
Deep Dive into CBO Directory Resource Activity
Overview of resources
Breakout room activity
Lincoln specific considerations

## Slide 7
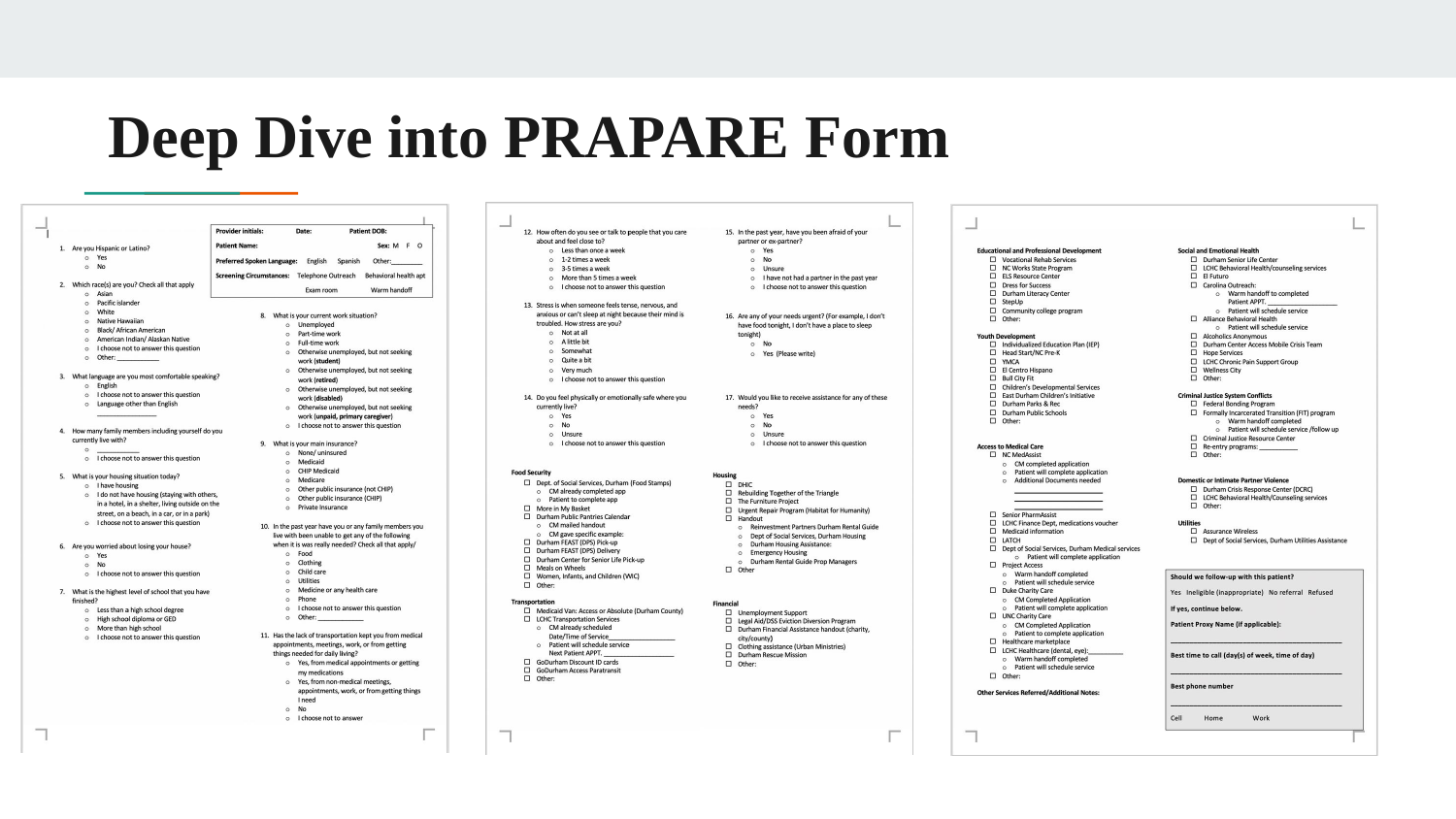

# Deep Dive into PRAPARE Form

## Slide 8
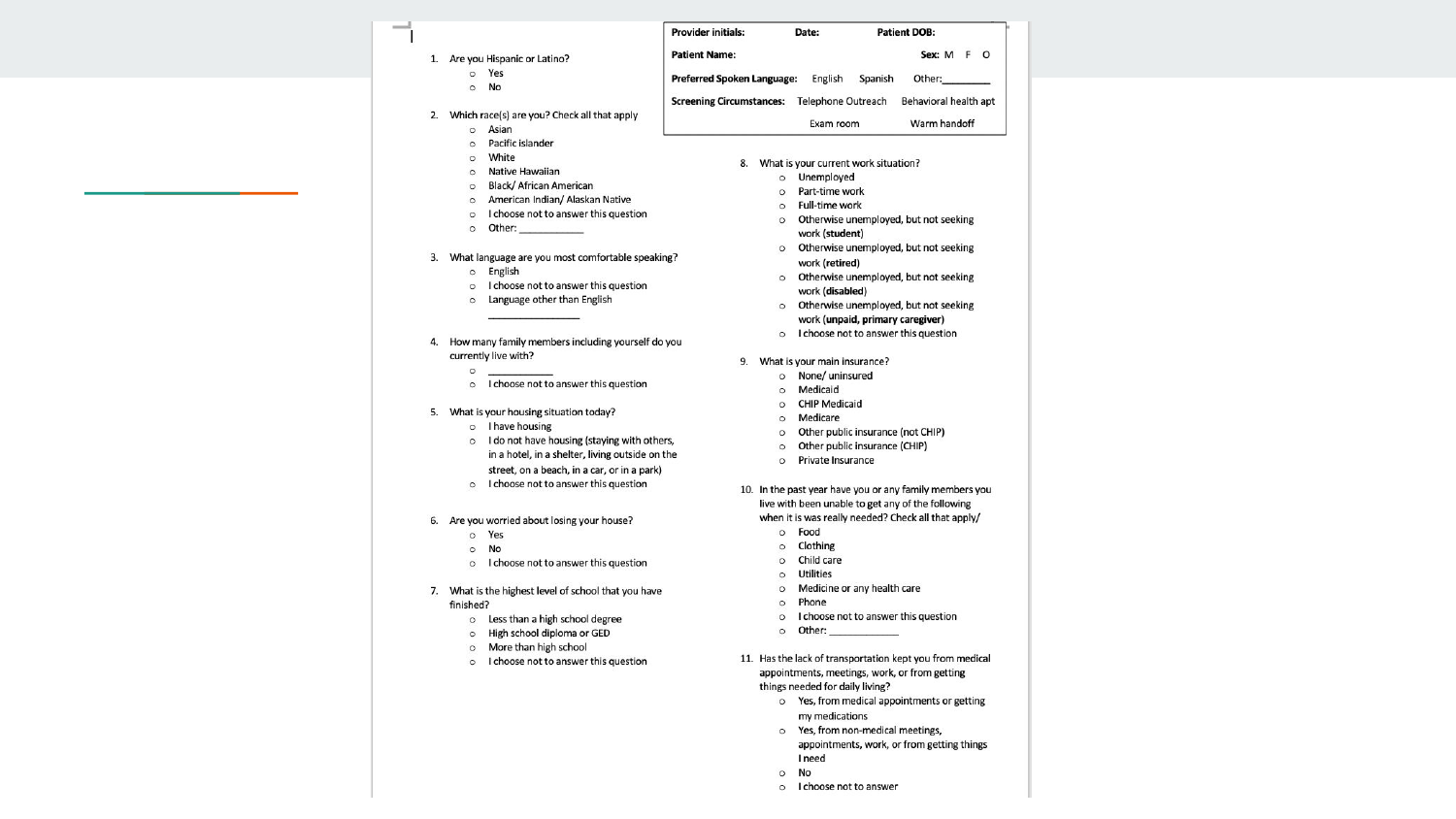

## Slide 9
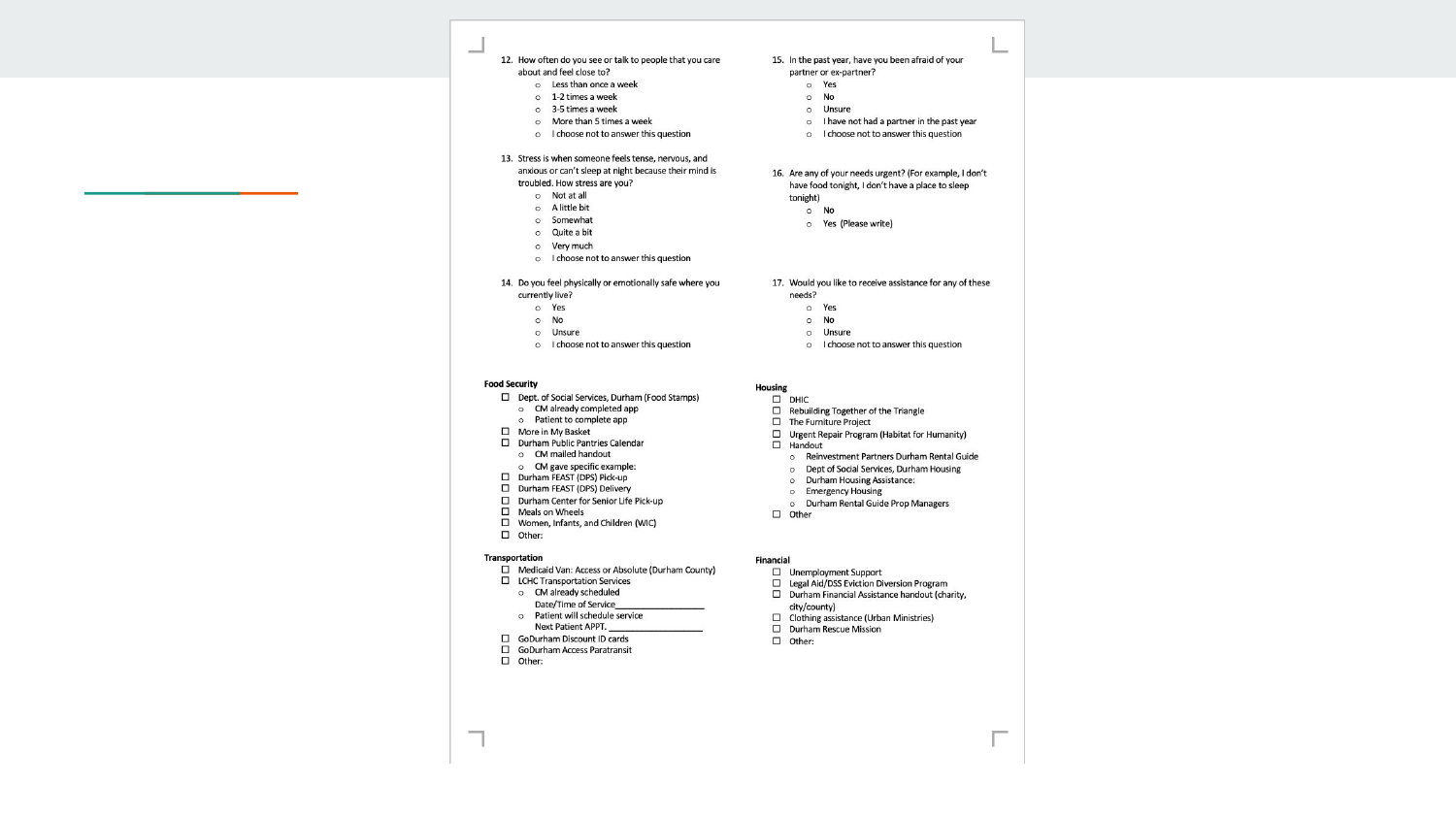

## Slide 10
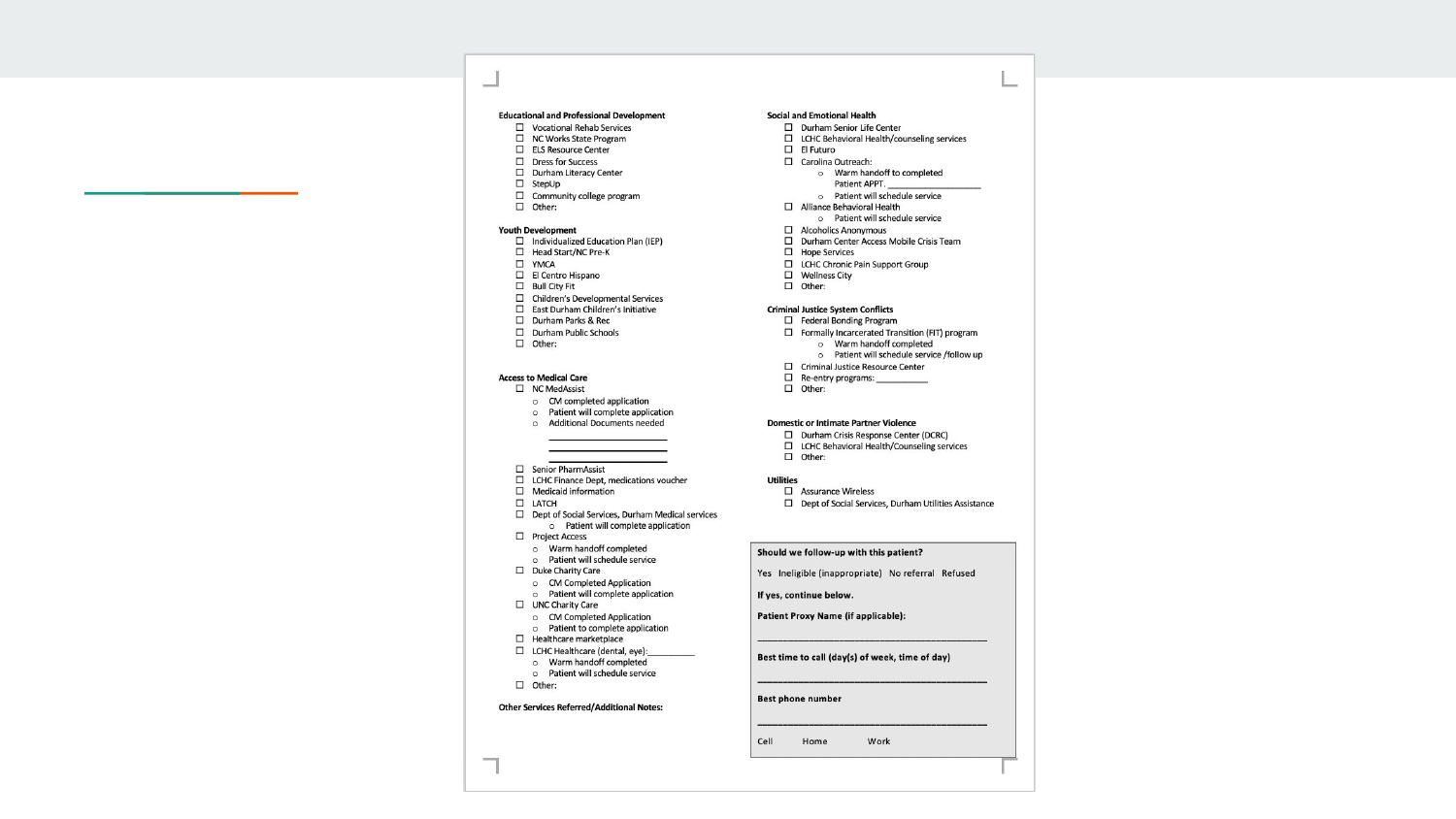

## Slide 11
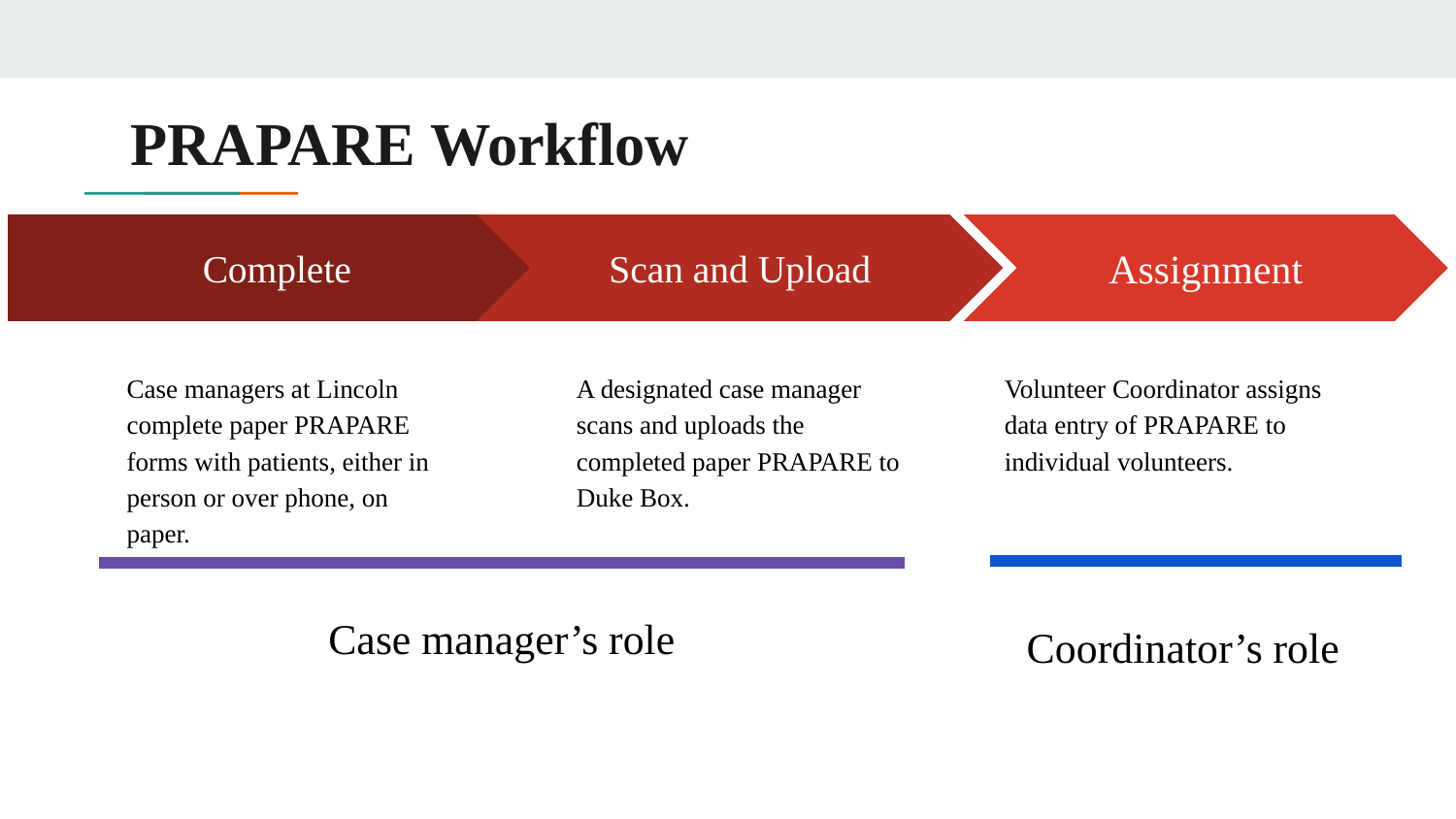

# PRAPARE Workflow
Scan and Upload
A designated case manager scans and uploads the completed paper PRAPARE to Duke Box.
Assignment
Volunteer Coordinator assigns data entry of PRAPARE to individual volunteers.
Complete
Case managers at Lincoln complete paper PRAPARE forms with patients, either in person or over phone, on paper.
Case manager’s role
Coordinator’s role

## Slide 12
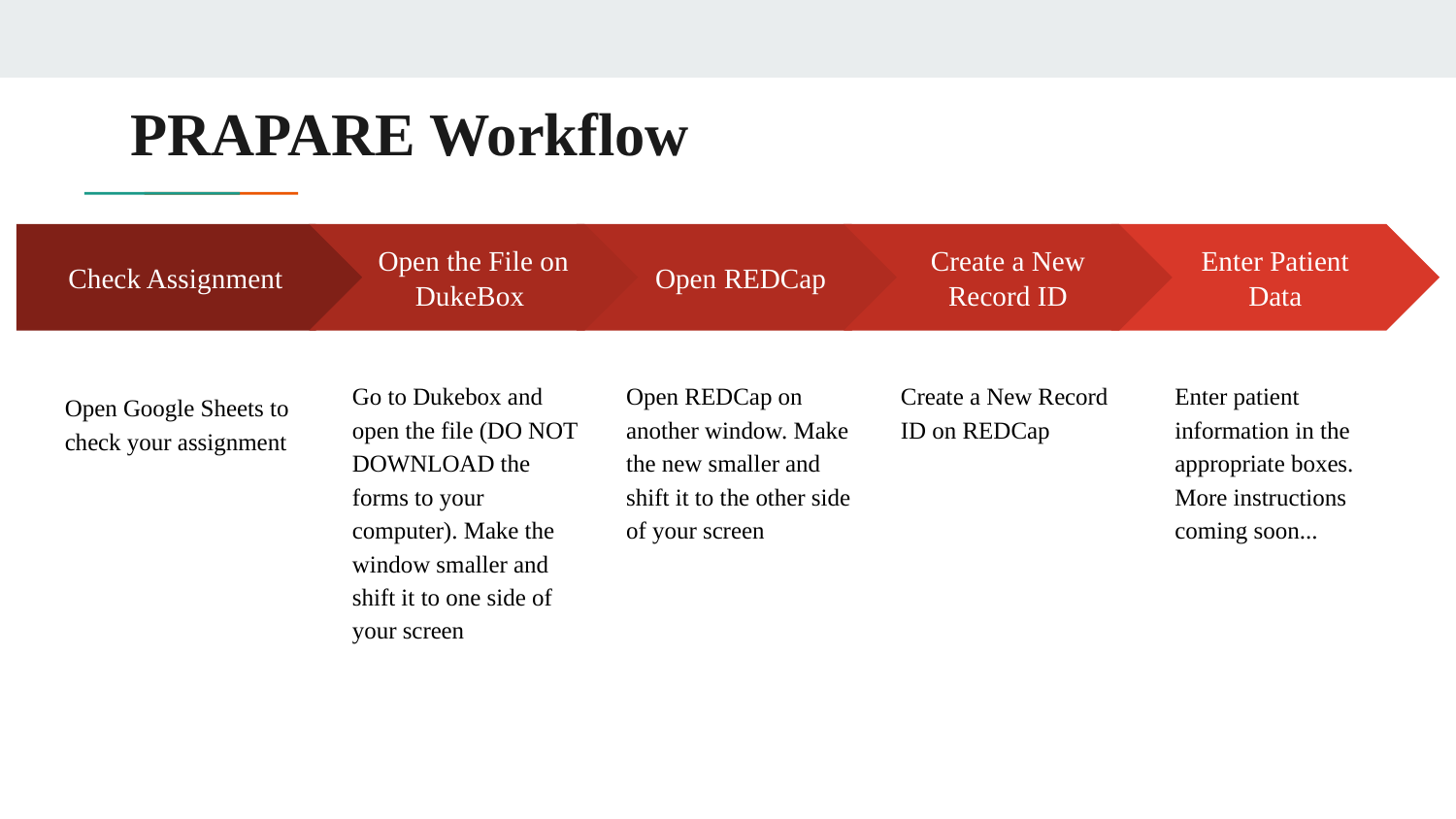

# PRAPARE Workflow
Open the File on DukeBox
Go to Dukebox and open the file (DO NOT DOWNLOAD the forms to your computer). Make the window smaller and shift it to one side of your screen
Open REDCap
Open REDCap on another window. Make the new smaller and shift it to the other side of your screen
Create a New Record ID
Create a New Record ID on REDCap
Enter Patient Data
Enter patient information in the appropriate boxes. More instructions coming soon...
Check Assignment
Open Google Sheets to check your assignment

## Slide 13
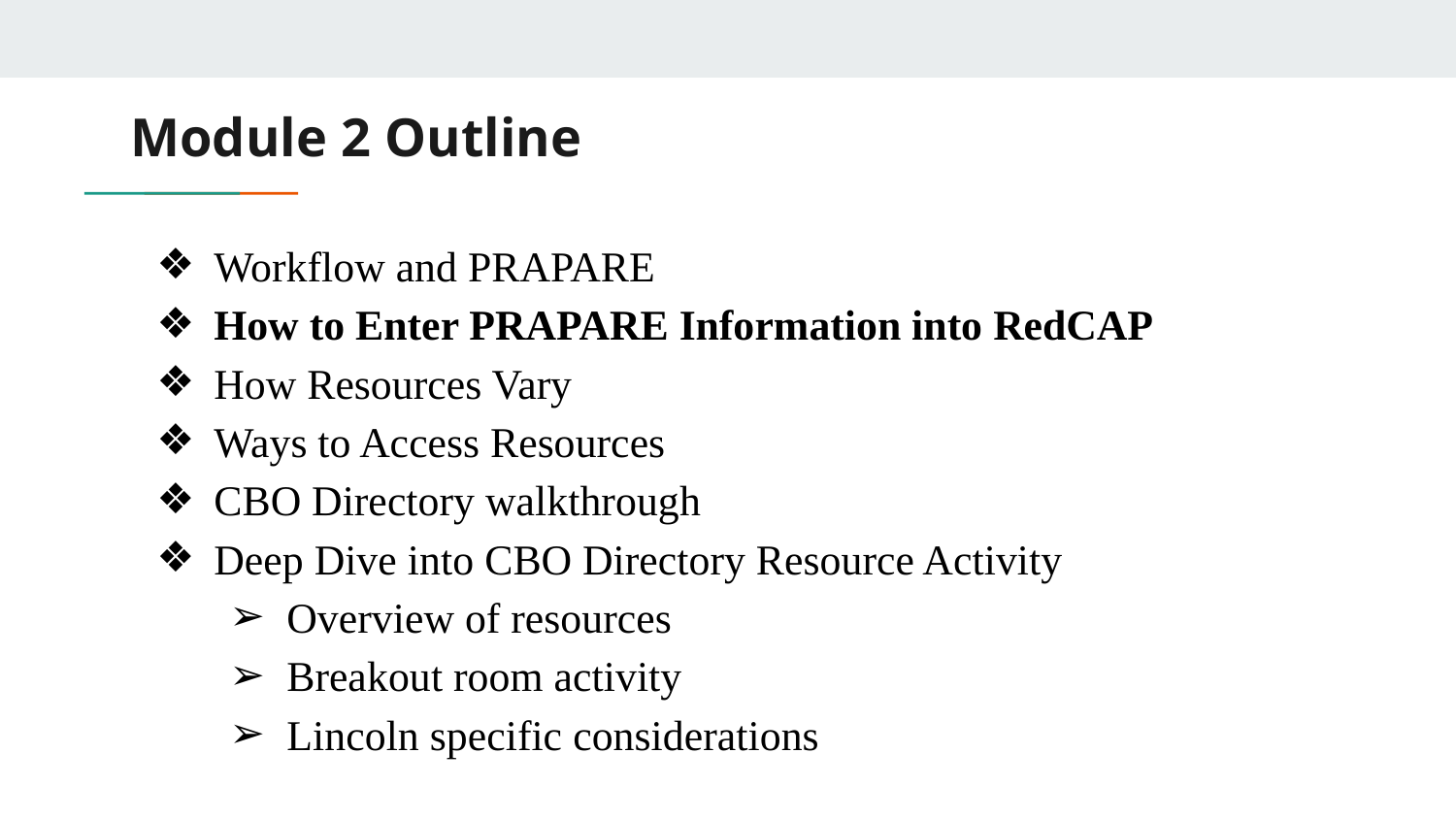

# Module 2 Outline
Workflow and PRAPARE
How to Enter PRAPARE Information into RedCAP
How Resources Vary
Ways to Access Resources
CBO Directory walkthrough
Deep Dive into CBO Directory Resource Activity
Overview of resources
Breakout room activity
Lincoln specific considerations

## Slide 14
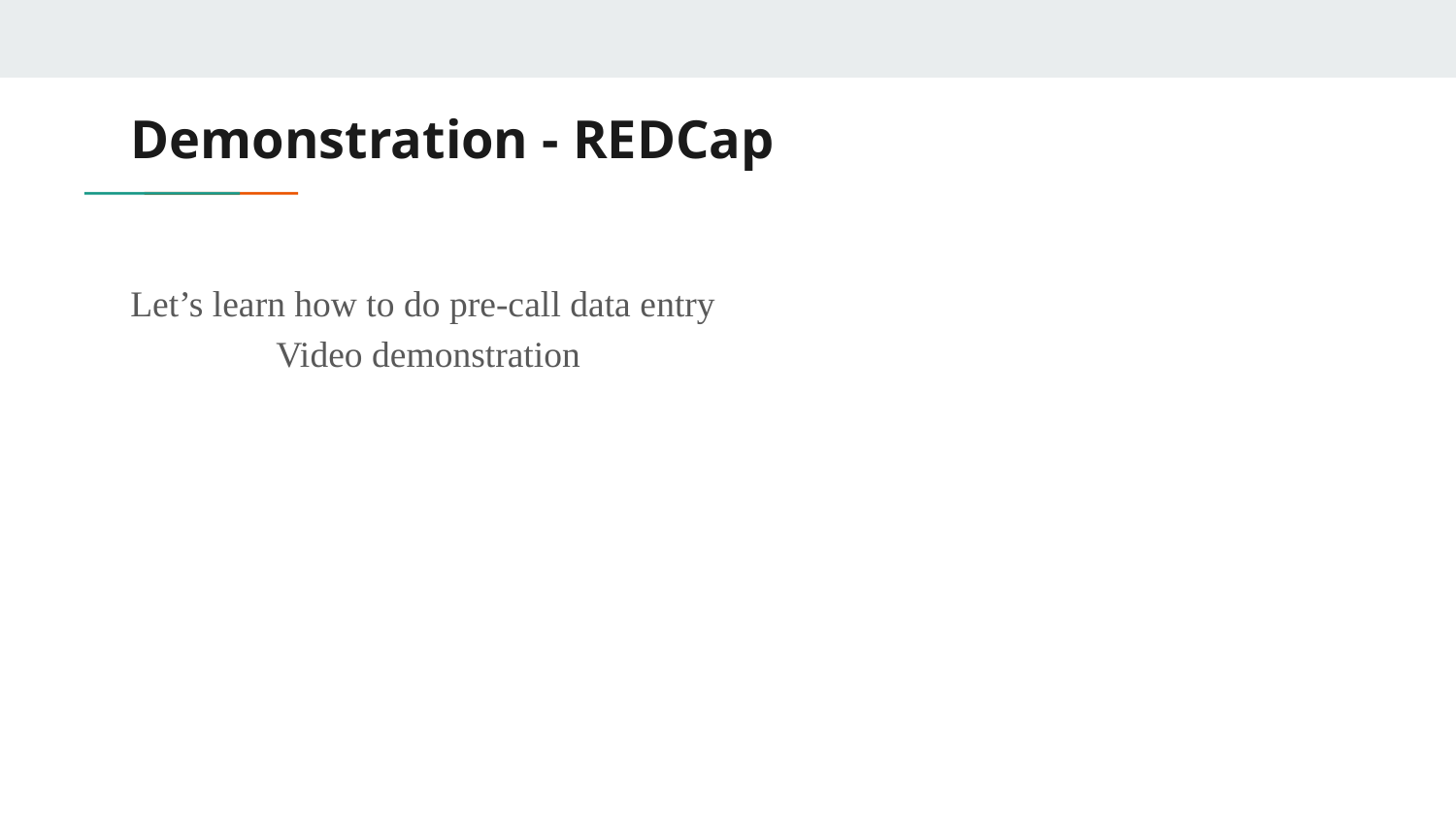

# Demonstration - REDCap
Let’s learn how to do pre-call data entry
	Video demonstration

## Slide 15
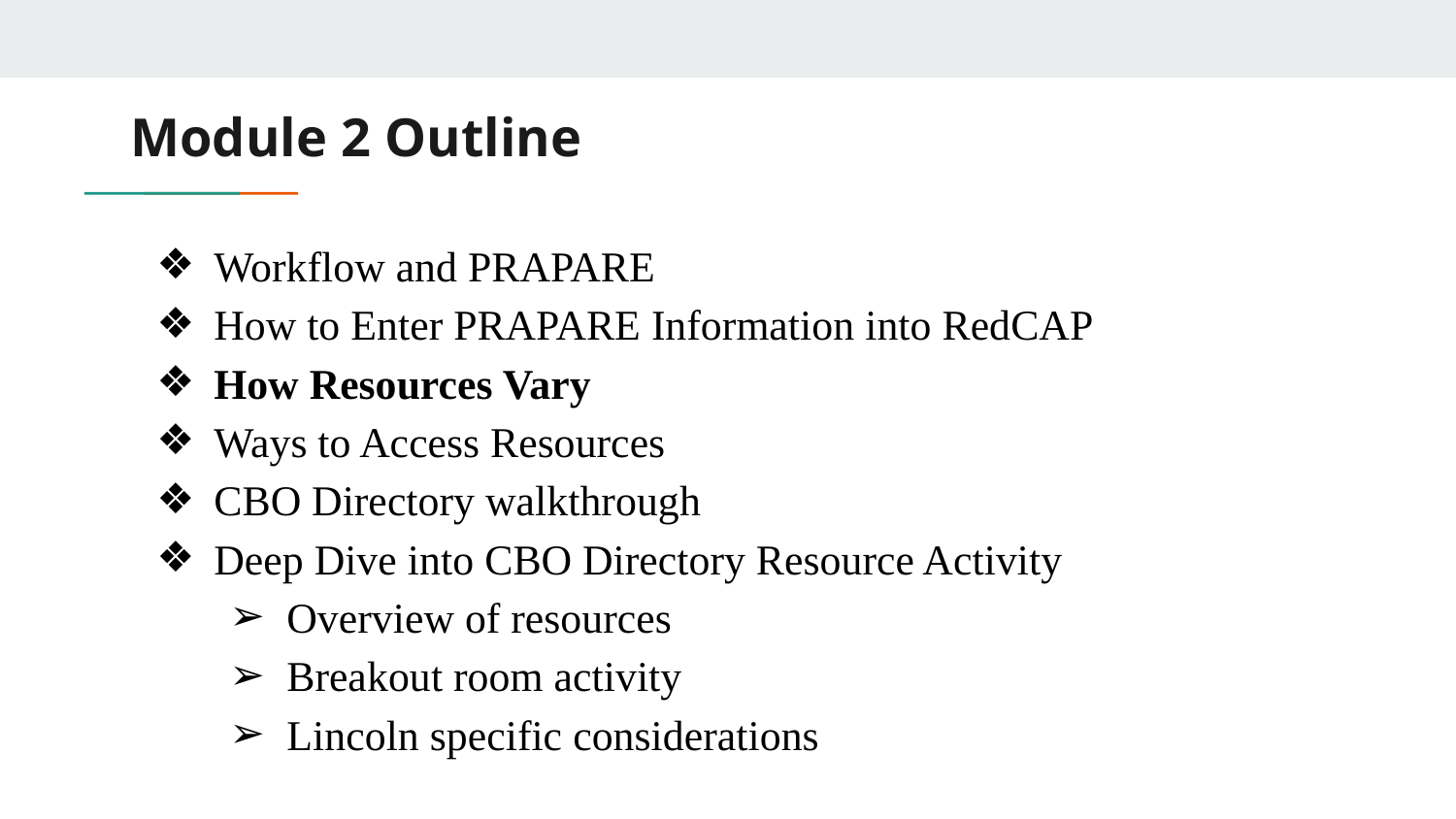

# Module 2 Outline
Workflow and PRAPARE
How to Enter PRAPARE Information into RedCAP
How Resources Vary
Ways to Access Resources
CBO Directory walkthrough
Deep Dive into CBO Directory Resource Activity
Overview of resources
Breakout room activity
Lincoln specific considerations

## Slide 16
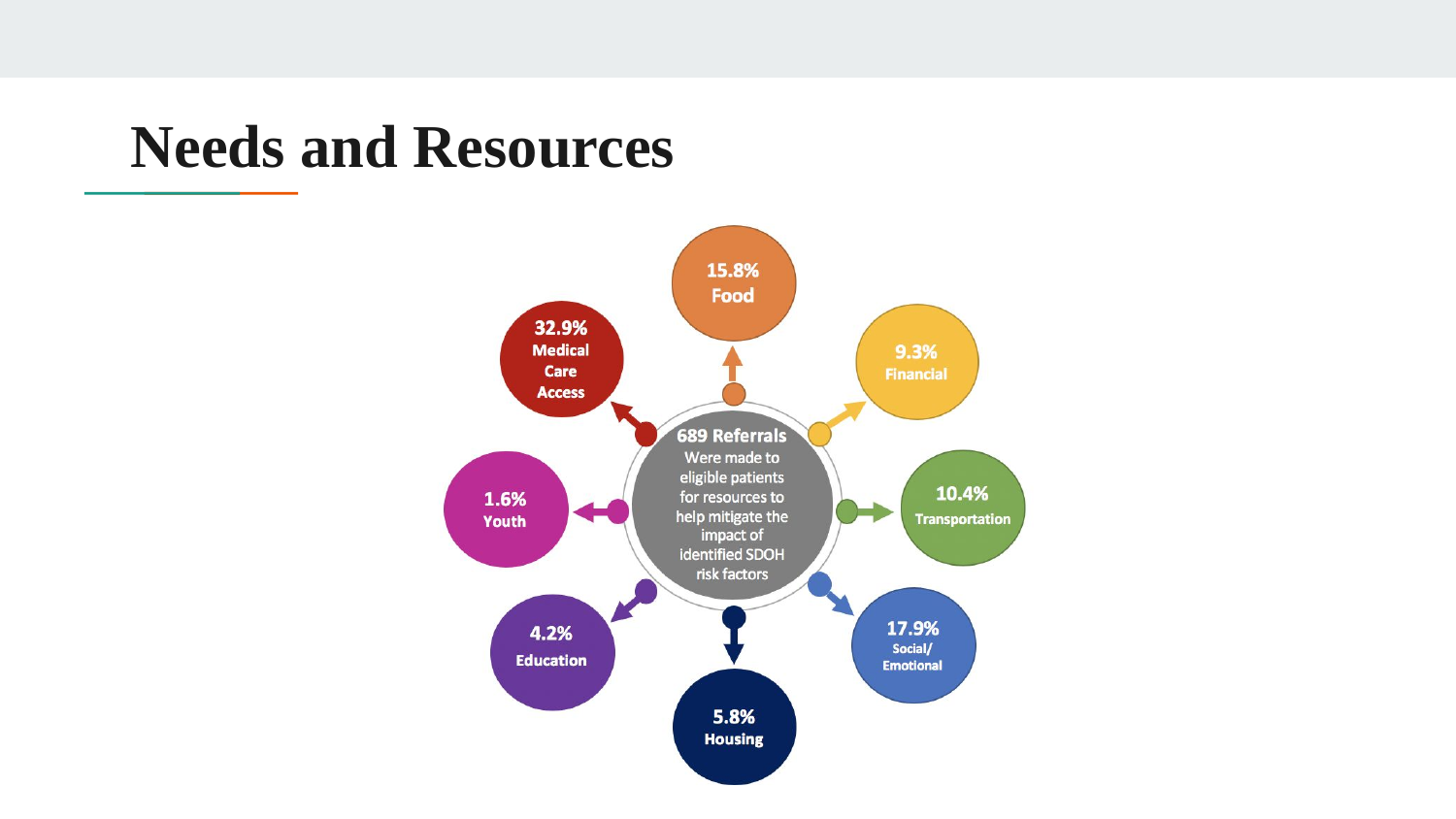

# Needs and Resources

## Slide 17
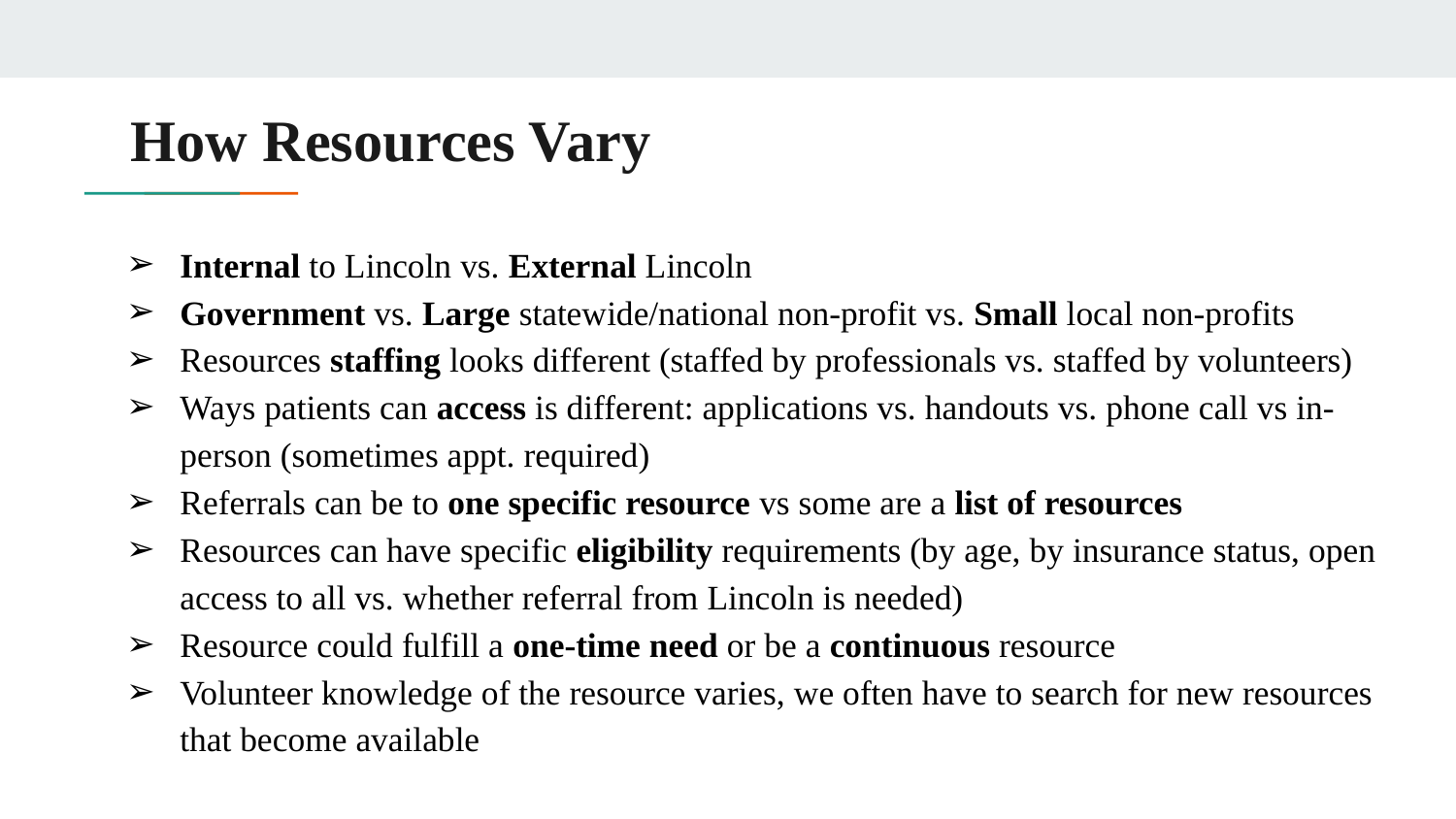

# How Resources Vary
Internal to Lincoln vs. External Lincoln
Government vs. Large statewide/national non-profit vs. Small local non-profits
Resources staffing looks different (staffed by professionals vs. staffed by volunteers)
Ways patients can access is different: applications vs. handouts vs. phone call vs in-person (sometimes appt. required)
Referrals can be to one specific resource vs some are a list of resources
Resources can have specific eligibility requirements (by age, by insurance status, open access to all vs. whether referral from Lincoln is needed)
Resource could fulfill a one-time need or be a continuous resource
Volunteer knowledge of the resource varies, we often have to search for new resources that become available

## Slide 18
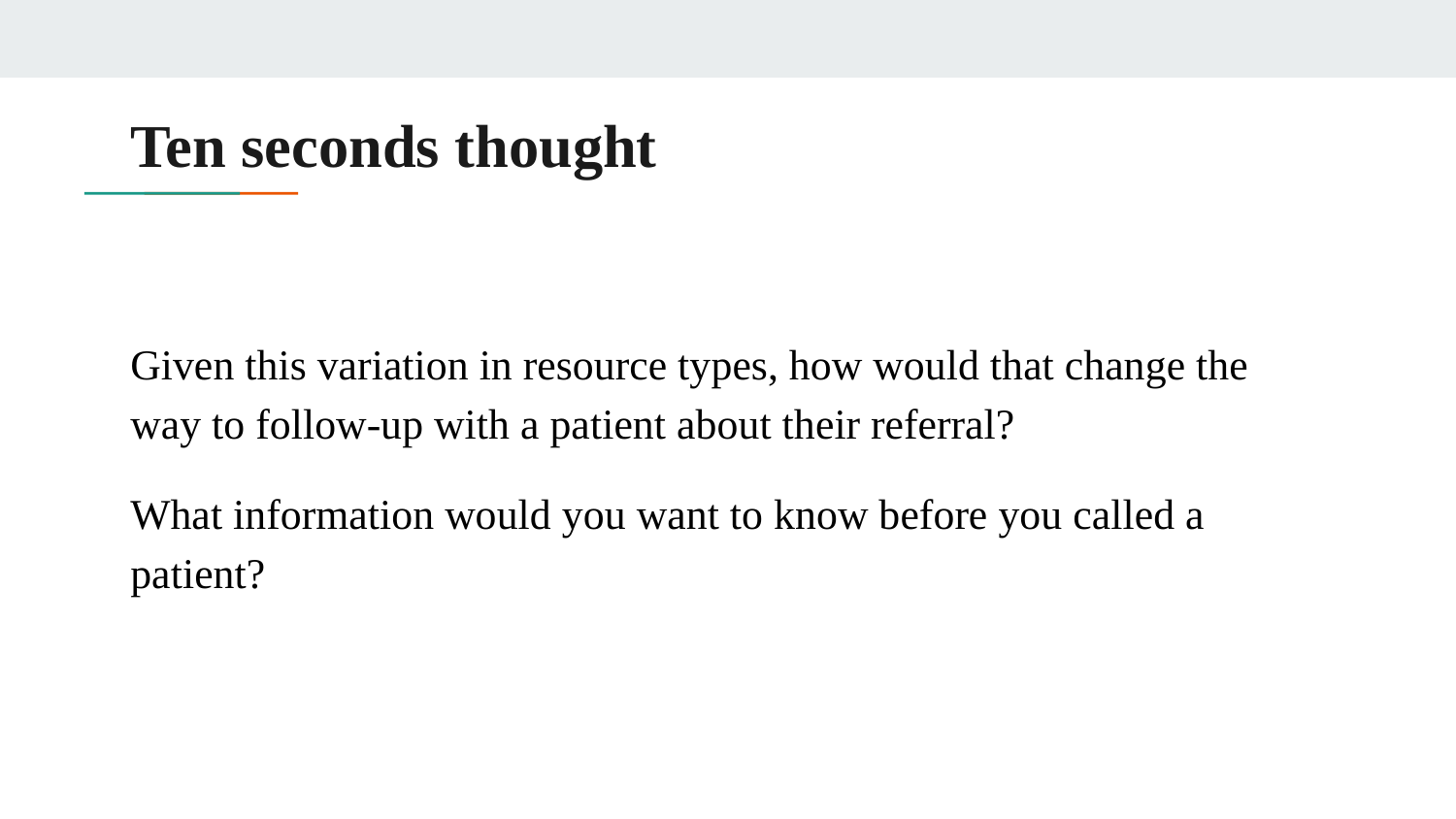

# Ten seconds thought
Given this variation in resource types, how would that change the way to follow-up with a patient about their referral?
What information would you want to know before you called a patient?

## Slide 19
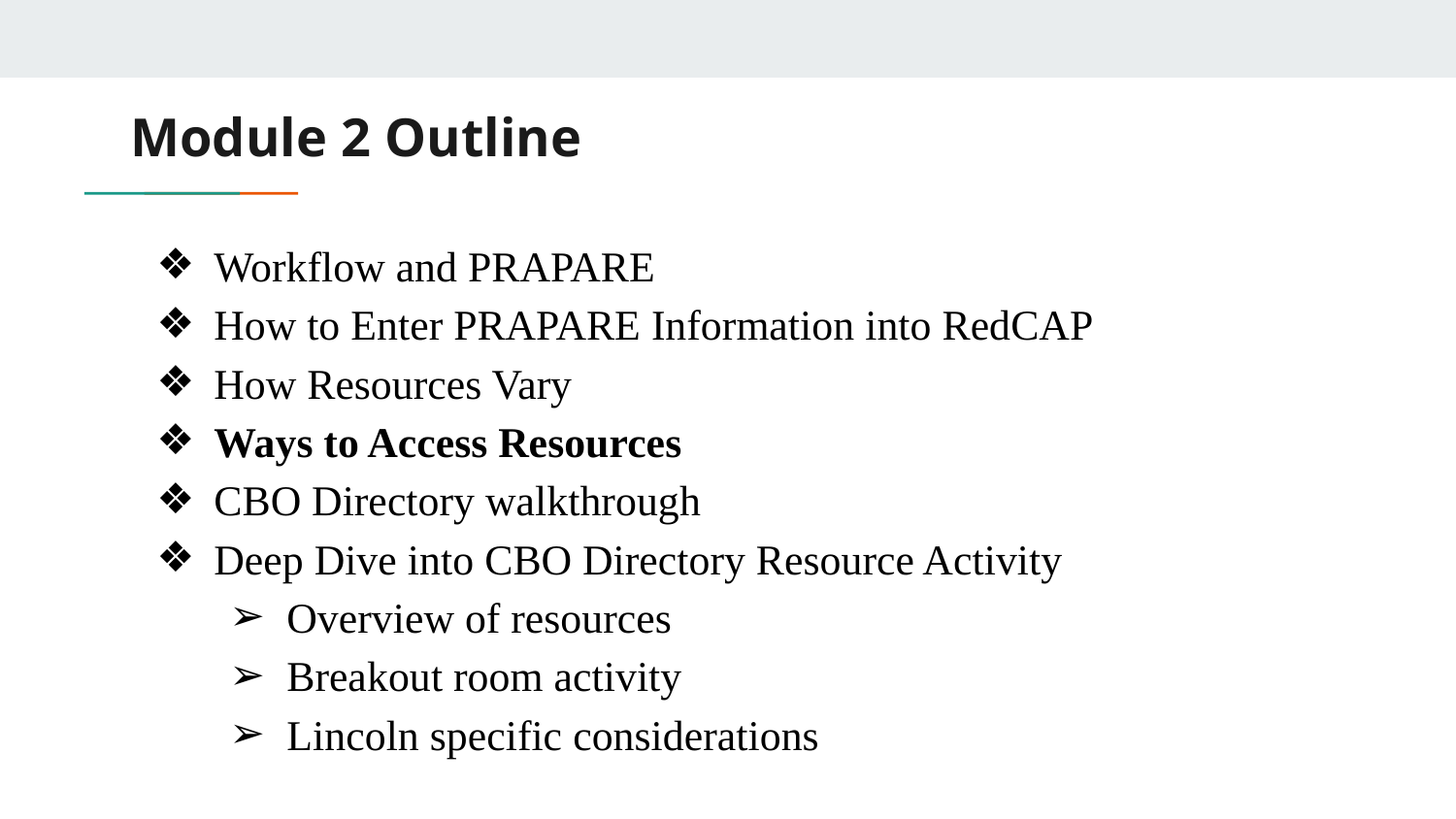

# Module 2 Outline
Workflow and PRAPARE
How to Enter PRAPARE Information into RedCAP
How Resources Vary
Ways to Access Resources
CBO Directory walkthrough
Deep Dive into CBO Directory Resource Activity
Overview of resources
Breakout room activity
Lincoln specific considerations

## Slide 20
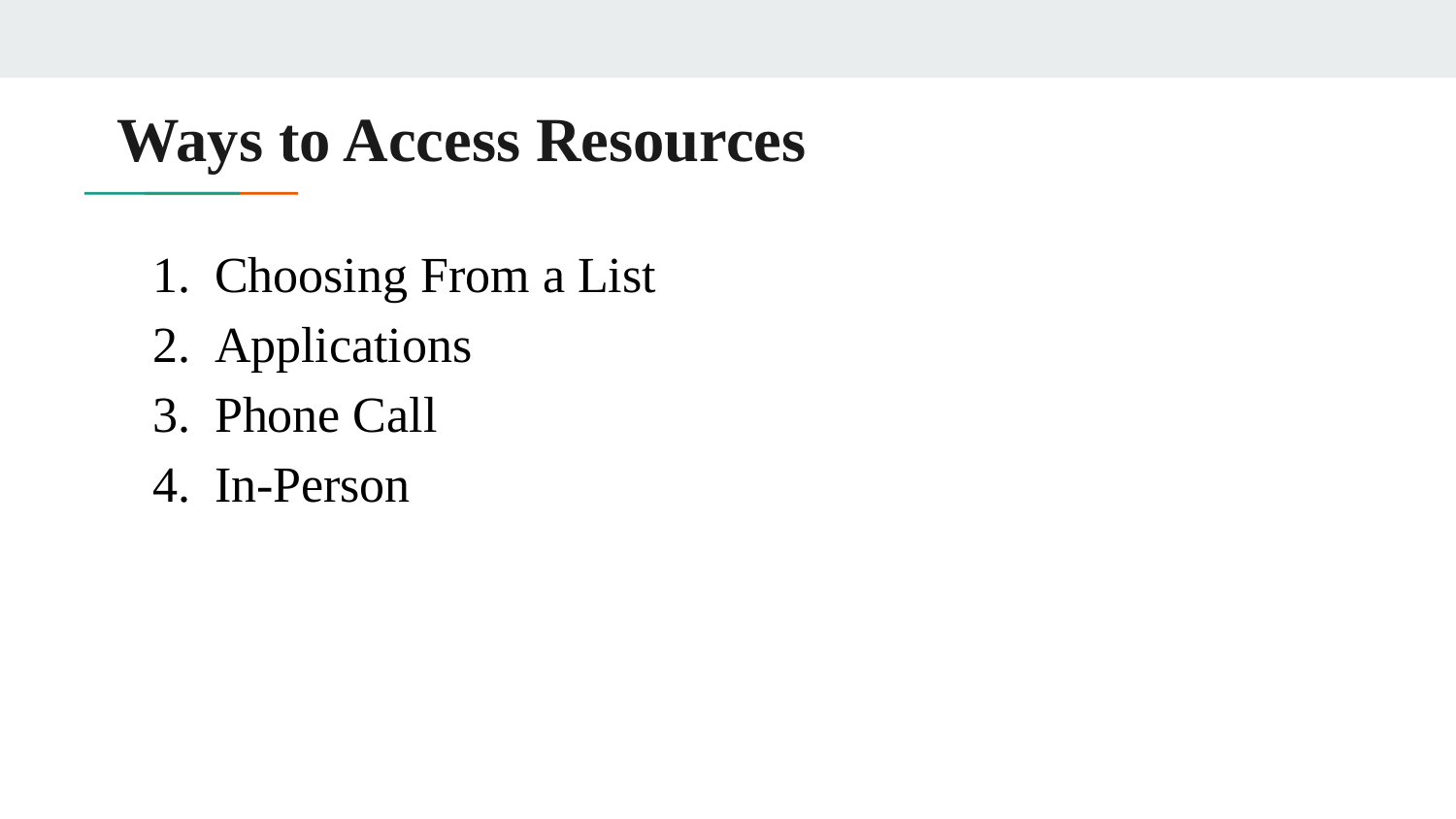

# Ways to Access Resources
Choosing From a List
Applications
Phone Call
In-Person

## Slide 21
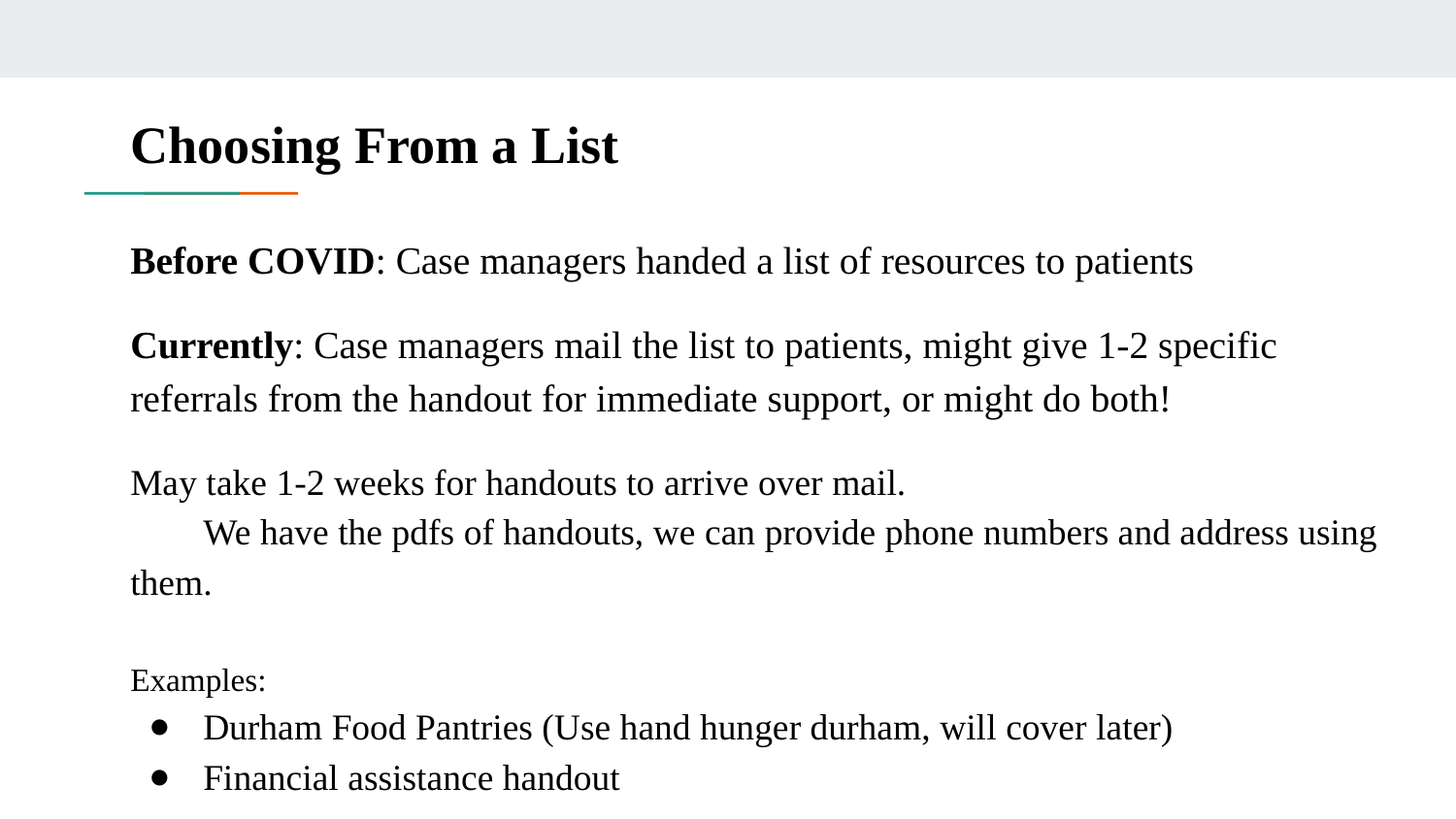

# Choosing From a List
Before COVID: Case managers handed a list of resources to patients
Currently: Case managers mail the list to patients, might give 1-2 specific referrals from the handout for immediate support, or might do both!
May take 1-2 weeks for handouts to arrive over mail.
We have the pdfs of handouts, we can provide phone numbers and address using them.
Examples:
Durham Food Pantries (Use hand hunger durham, will cover later)
Financial assistance handout

## Slide 22
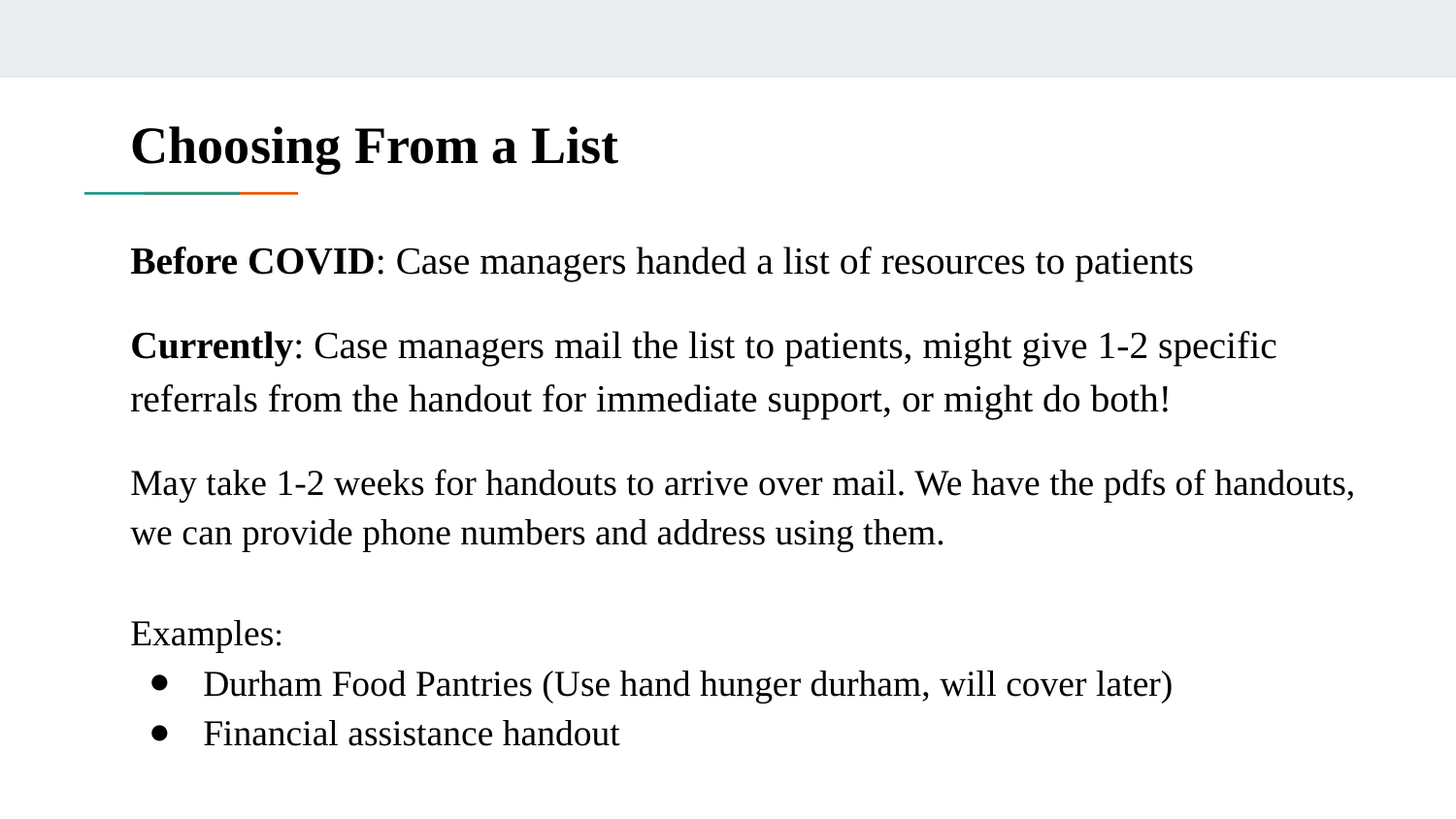

# Choosing From a List
Before COVID: Case managers handed a list of resources to patients
Currently: Case managers mail the list to patients, might give 1-2 specific referrals from the handout for immediate support, or might do both!
May take 1-2 weeks for handouts to arrive over mail. We have the pdfs of handouts, we can provide phone numbers and address using them.
Examples:
Durham Food Pantries (Use hand hunger durham, will cover later)
Financial assistance handout

## Slide 23
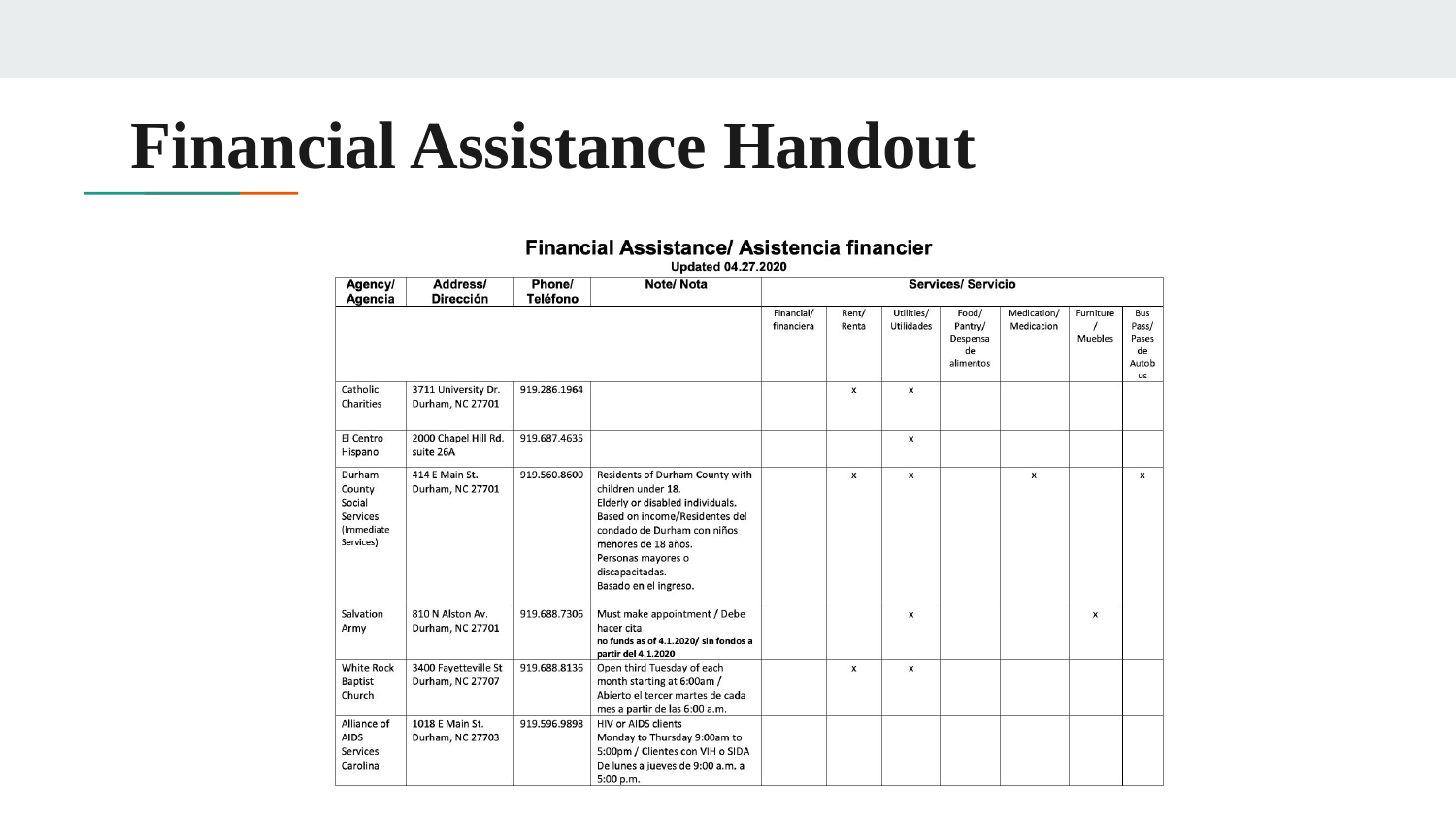

# Financial Assistance Handout

## Slide 24
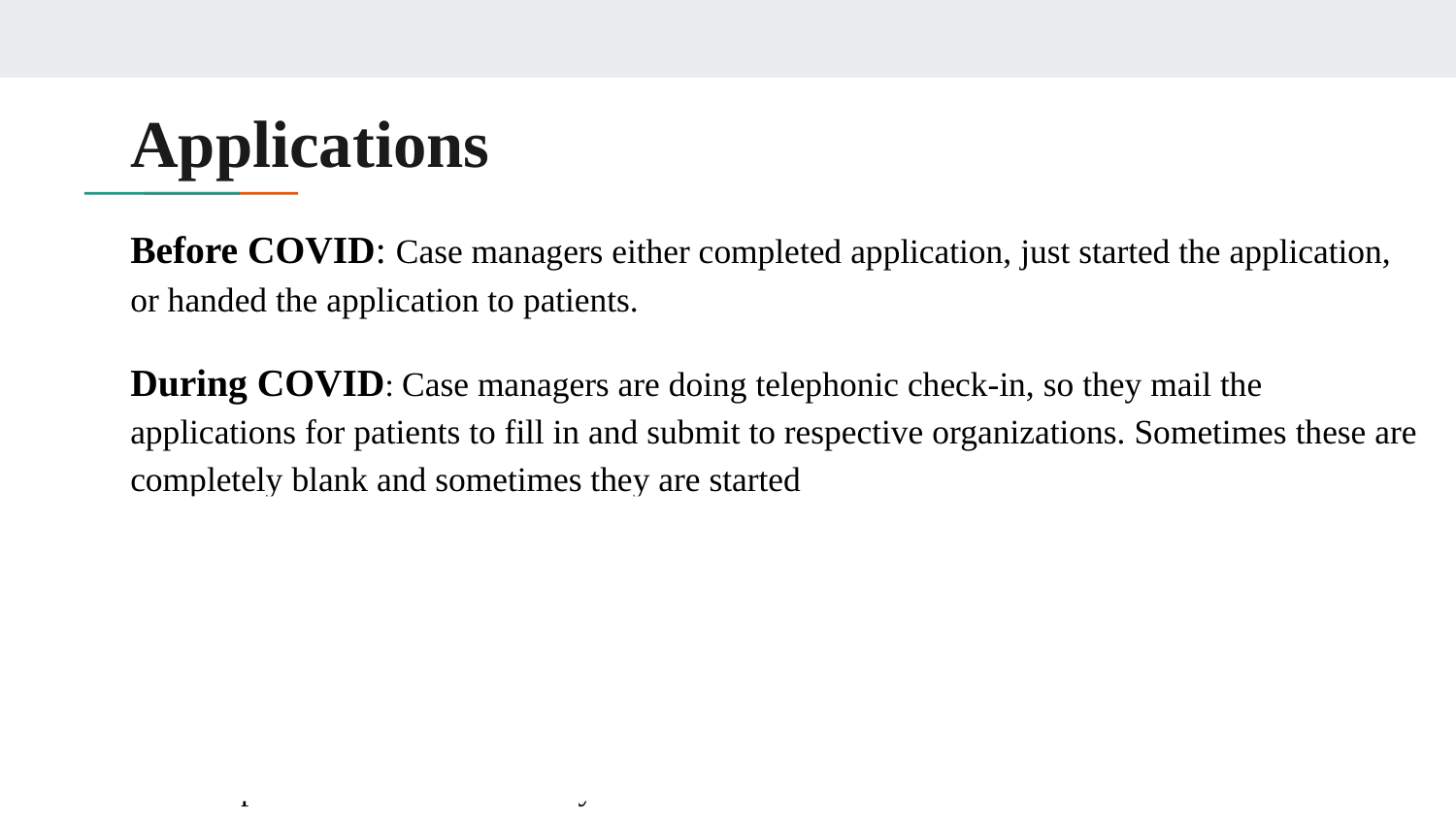

# Applications
Before COVID: Case managers either completed application, just started the application, or handed the application to patients.
During COVID: Case managers are doing telephonic check-in, so they mail the applications for patients to fill in and submit to respective organizations. Sometimes these are completely blank and sometimes they are started
Usually takes 2-4 weeks for approval
Ask if they received and submitted the app
Examples:
SNAP-Food stamps
Department of Social Security

## Slide 25
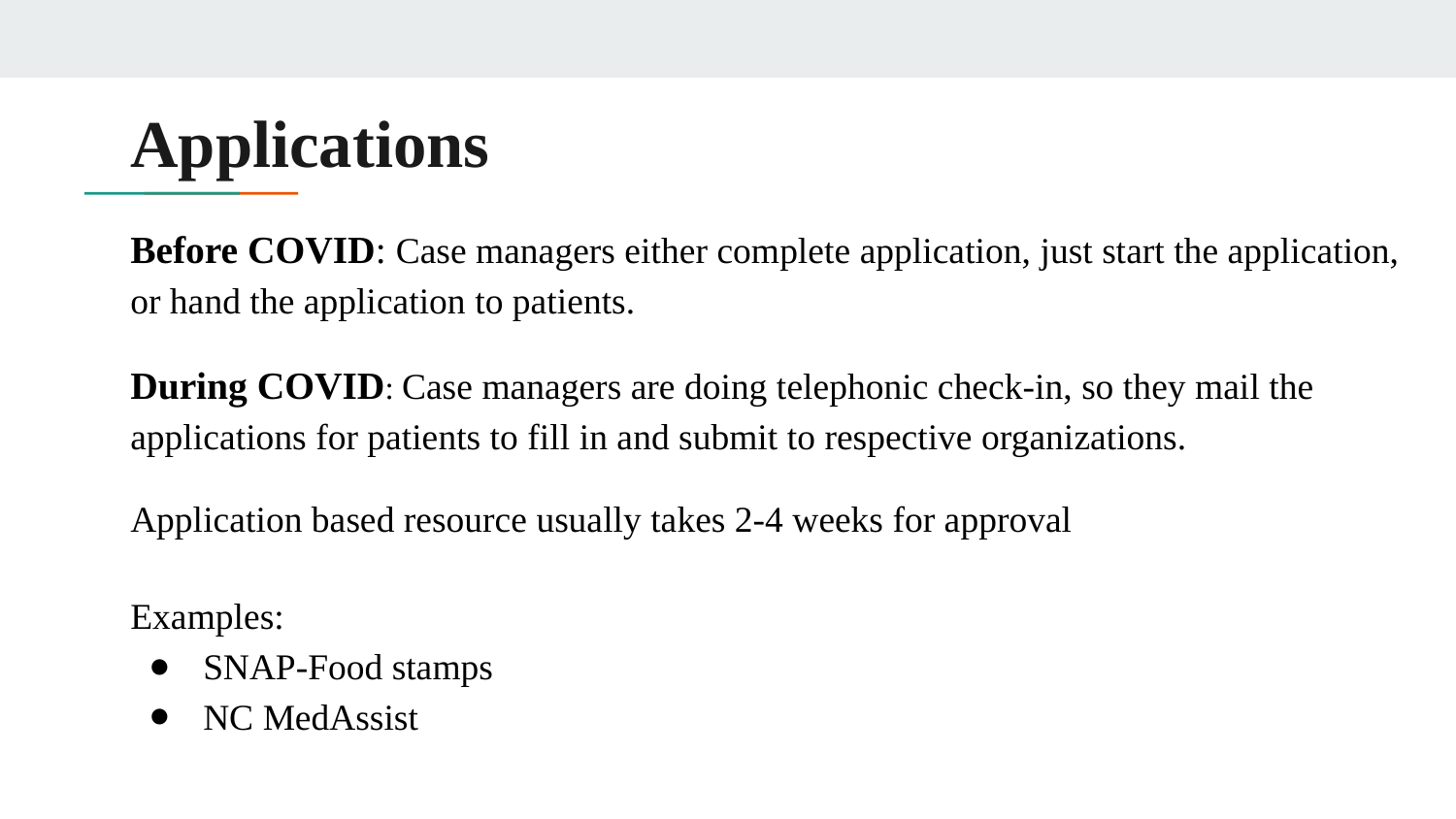

# Applications
Before COVID: Case managers either complete application, just start the application, or hand the application to patients.
During COVID: Case managers are doing telephonic check-in, so they mail the applications for patients to fill in and submit to respective organizations.
Application based resource usually takes 2-4 weeks for approval
Examples:
SNAP-Food stamps
NC MedAssist

## Slide 26
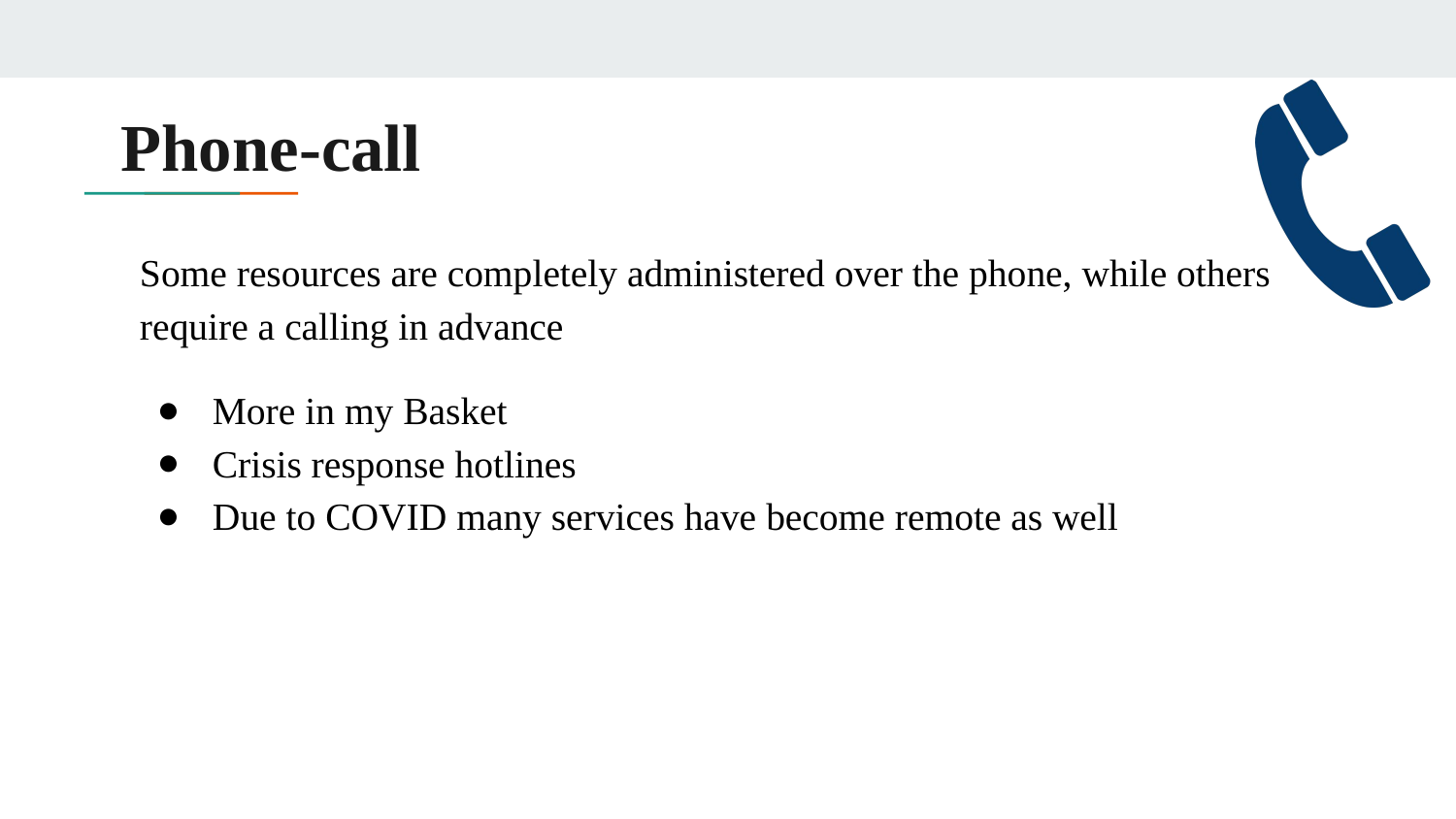

# Phone-call
Some resources are completely administered over the phone, while others require a calling in advance
More in my Basket
Crisis response hotlines
Due to COVID many services have become remote as well

## Slide 27
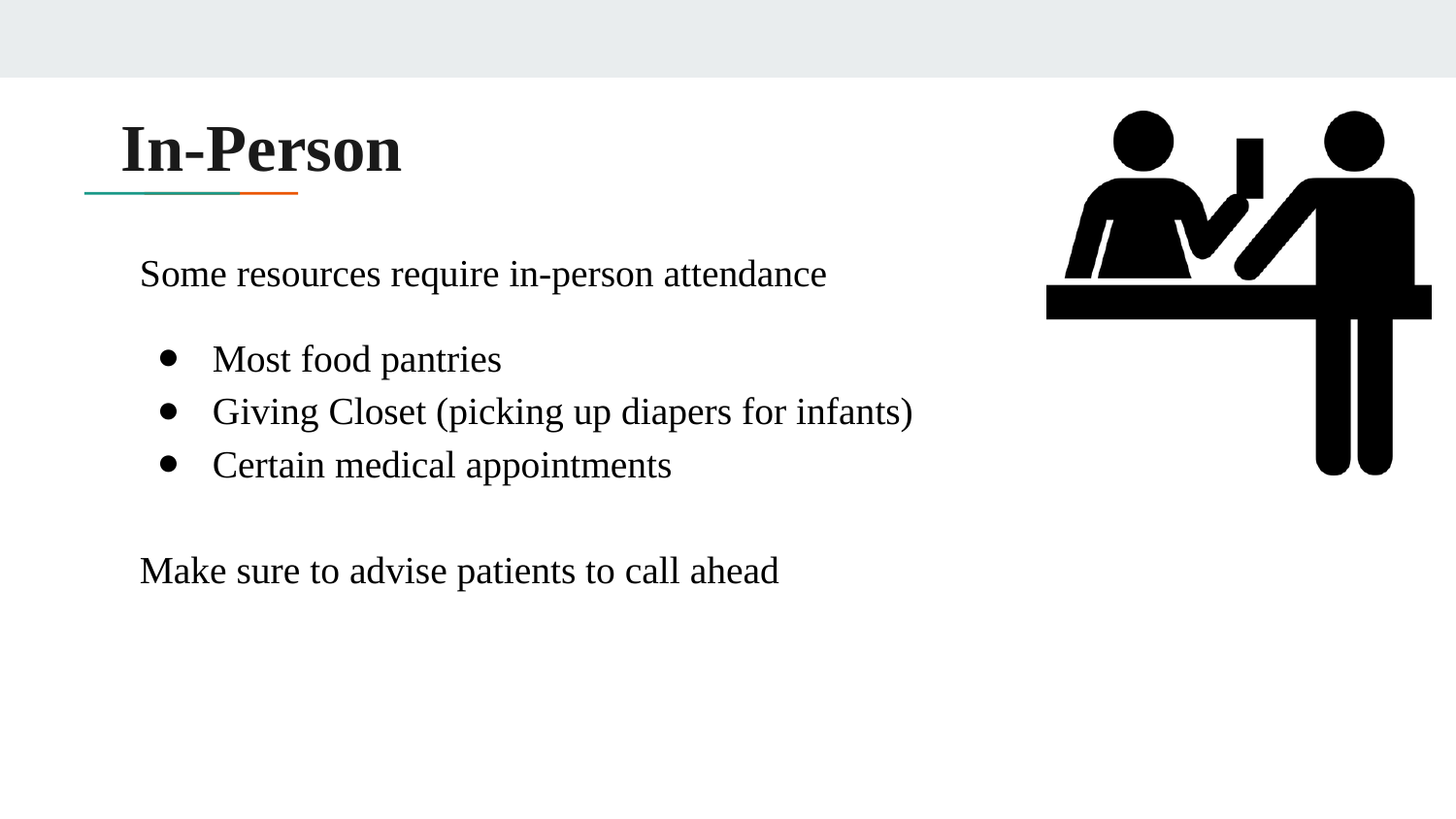

# In-Person
Some resources require in-person attendance
Most food pantries
Giving Closet (picking up diapers for infants)
Certain medical appointments
Make sure to advise patients to call ahead

## Slide 28
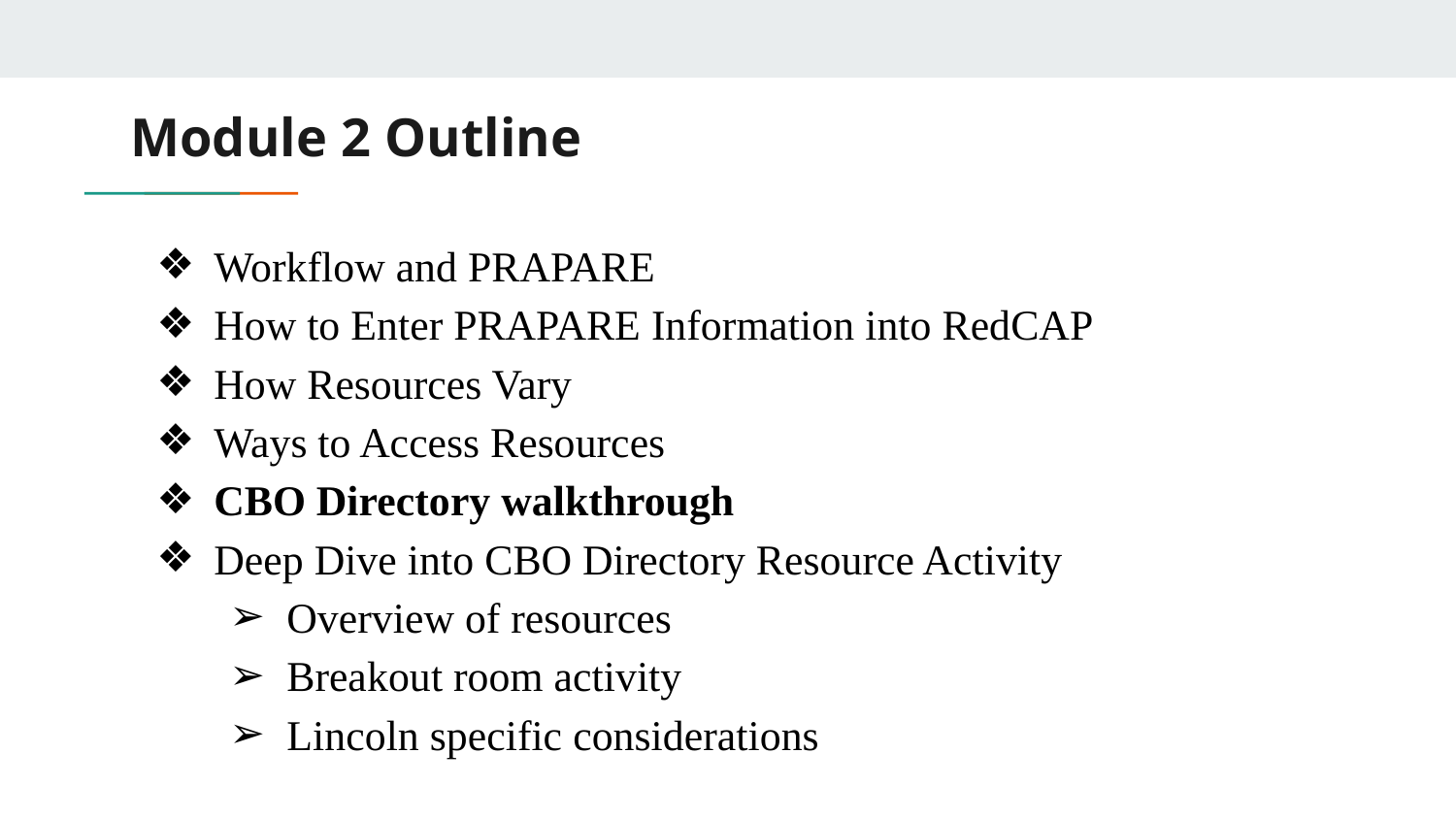

# Module 2 Outline
Workflow and PRAPARE
How to Enter PRAPARE Information into RedCAP
How Resources Vary
Ways to Access Resources
CBO Directory walkthrough
Deep Dive into CBO Directory Resource Activity
Overview of resources
Breakout room activity
Lincoln specific considerations

## Slide 29
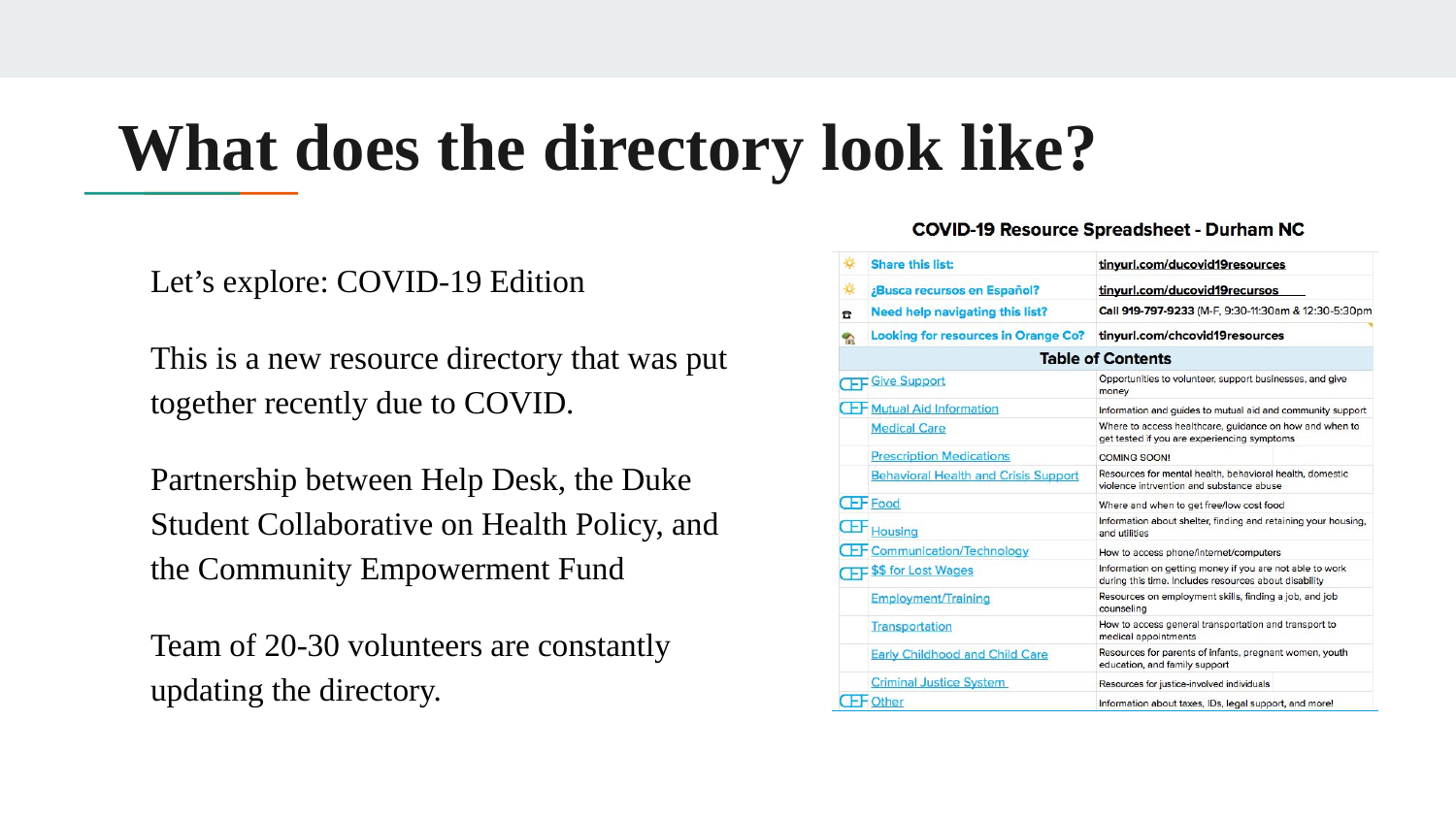

# What does the directory look like?
Let’s explore: COVID-19 Edition
This is a new resource directory that was put together recently due to COVID.
Partnership between Help Desk, the Duke Student Collaborative on Health Policy, and the Community Empowerment Fund
Team of 20-30 volunteers are constantly updating the directory.

## Slide 30
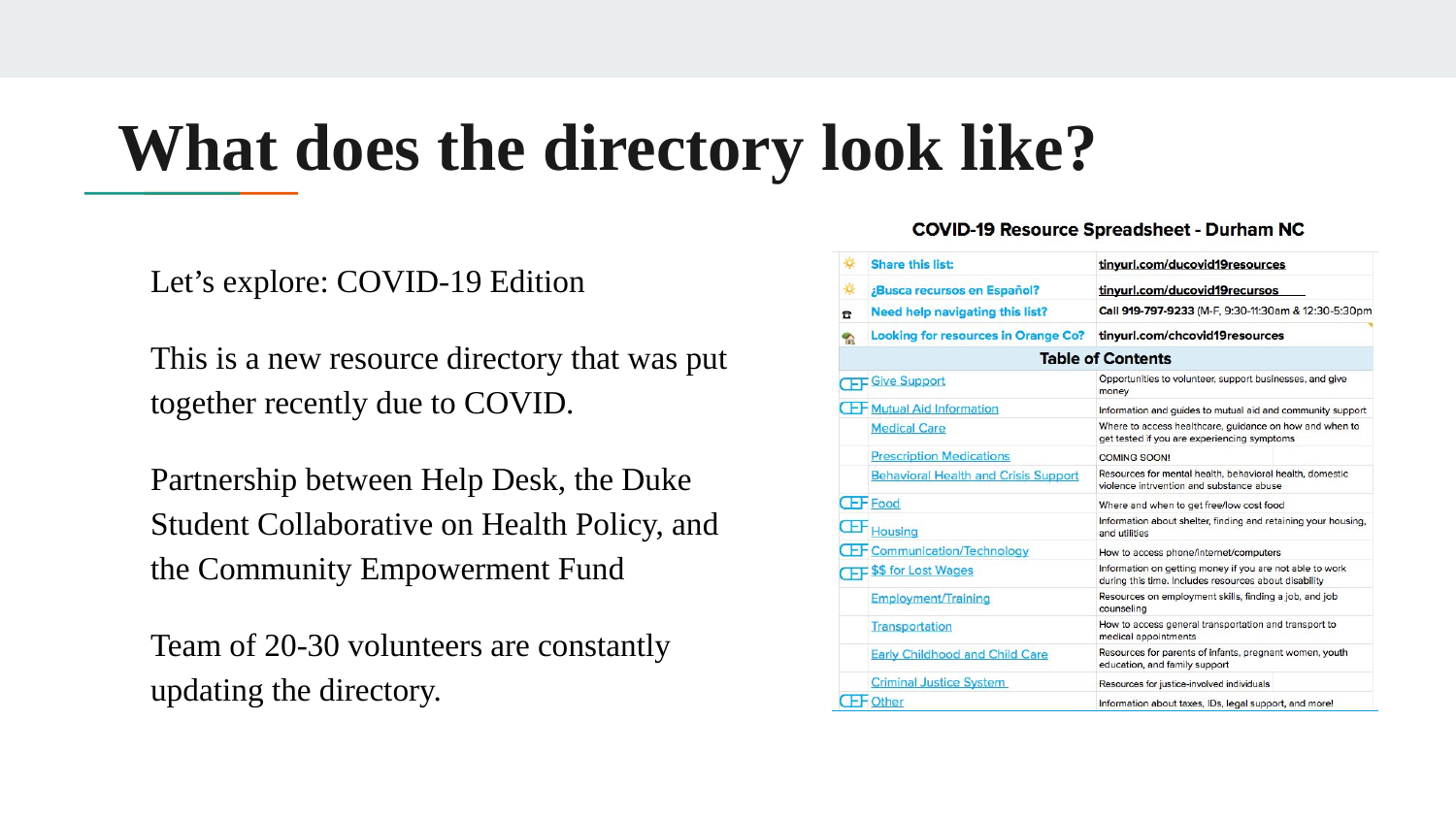

# What does the directory look like?
Let’s explore: COVID-19 Edition
This is a new resource directory that was put together recently due to COVID.
Partnership between Help Desk, the Duke Student Collaborative on Health Policy, and the Community Empowerment Fund
Team of 20-30 volunteers are constantly updating the directory.

## Slide 31
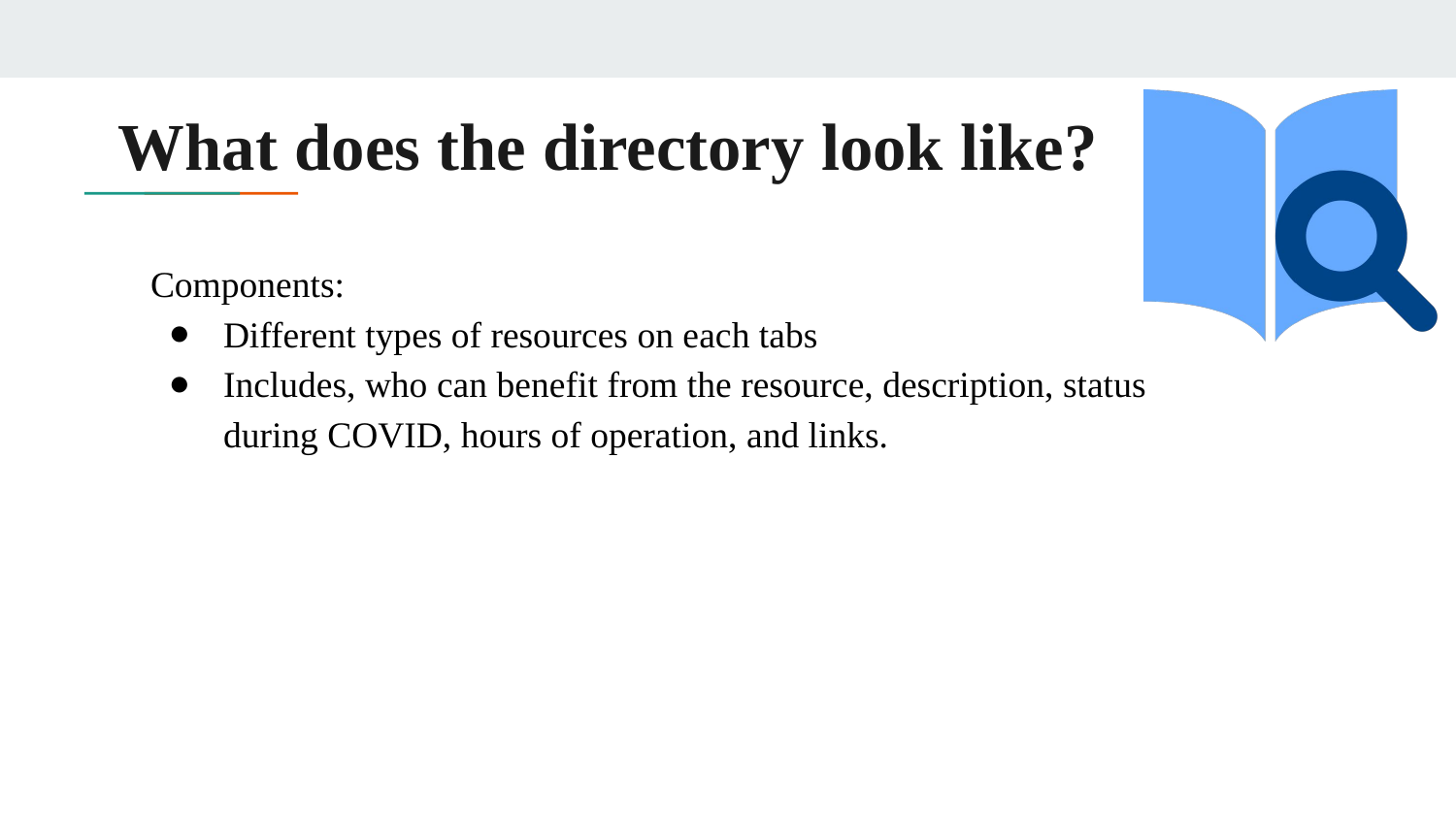

# What does the directory look like?
Components:
Different types of resources on each tabs
Includes, who can benefit from the resource, description, status during COVID, hours of operation, and links.

## Slide 32
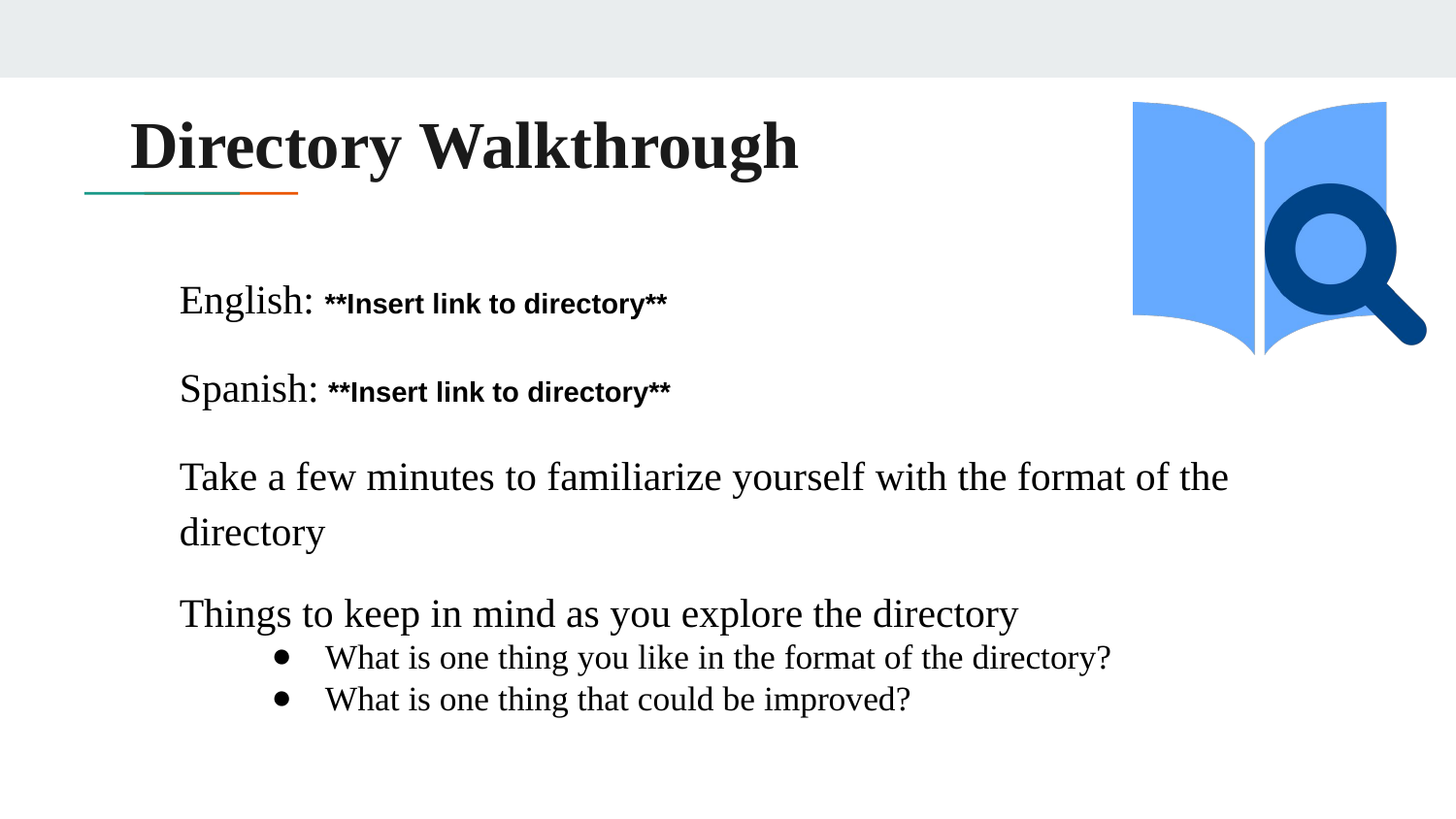

# Directory Walkthrough
English: **Insert link to directory**
Spanish: **Insert link to directory**
Take a few minutes to familiarize yourself with the format of the directory
Things to keep in mind as you explore the directory
What is one thing you like in the format of the directory?
What is one thing that could be improved?

## Slide 33
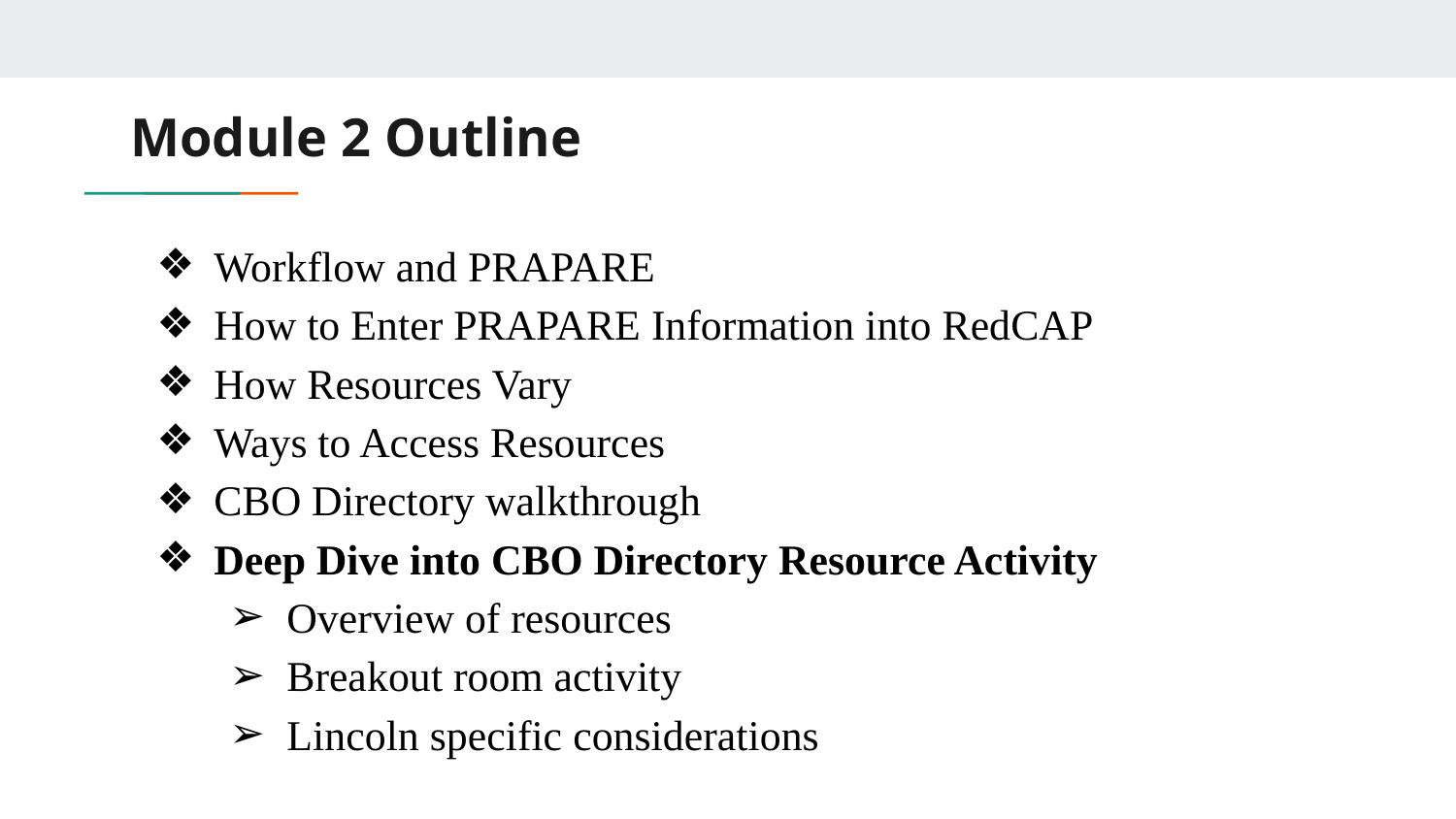

# Module 2 Outline
Workflow and PRAPARE
How to Enter PRAPARE Information into RedCAP
How Resources Vary
Ways to Access Resources
CBO Directory walkthrough
Deep Dive into CBO Directory Resource Activity
Overview of resources
Breakout room activity
Lincoln specific considerations

## Slide 34
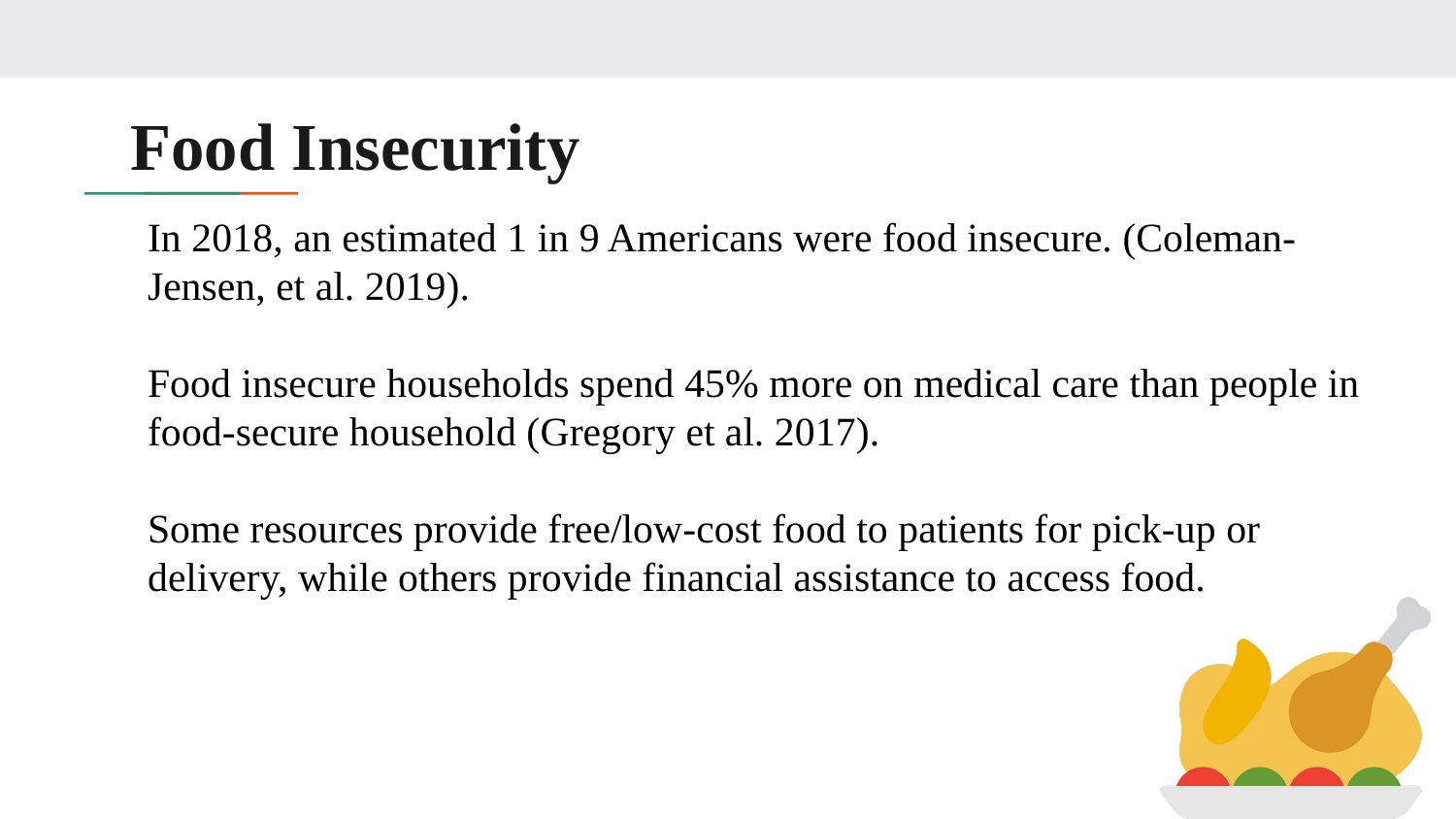

# Food Insecurity
In 2018, an estimated 1 in 9 Americans were food insecure. (Coleman-Jensen, et al. 2019).
Food insecure households spend 45% more on medical care than people in food-secure household (Gregory et al. 2017).
Some resources provide free/low-cost food to patients for pick-up or delivery, while others provide financial assistance to access food.

## Slide 35
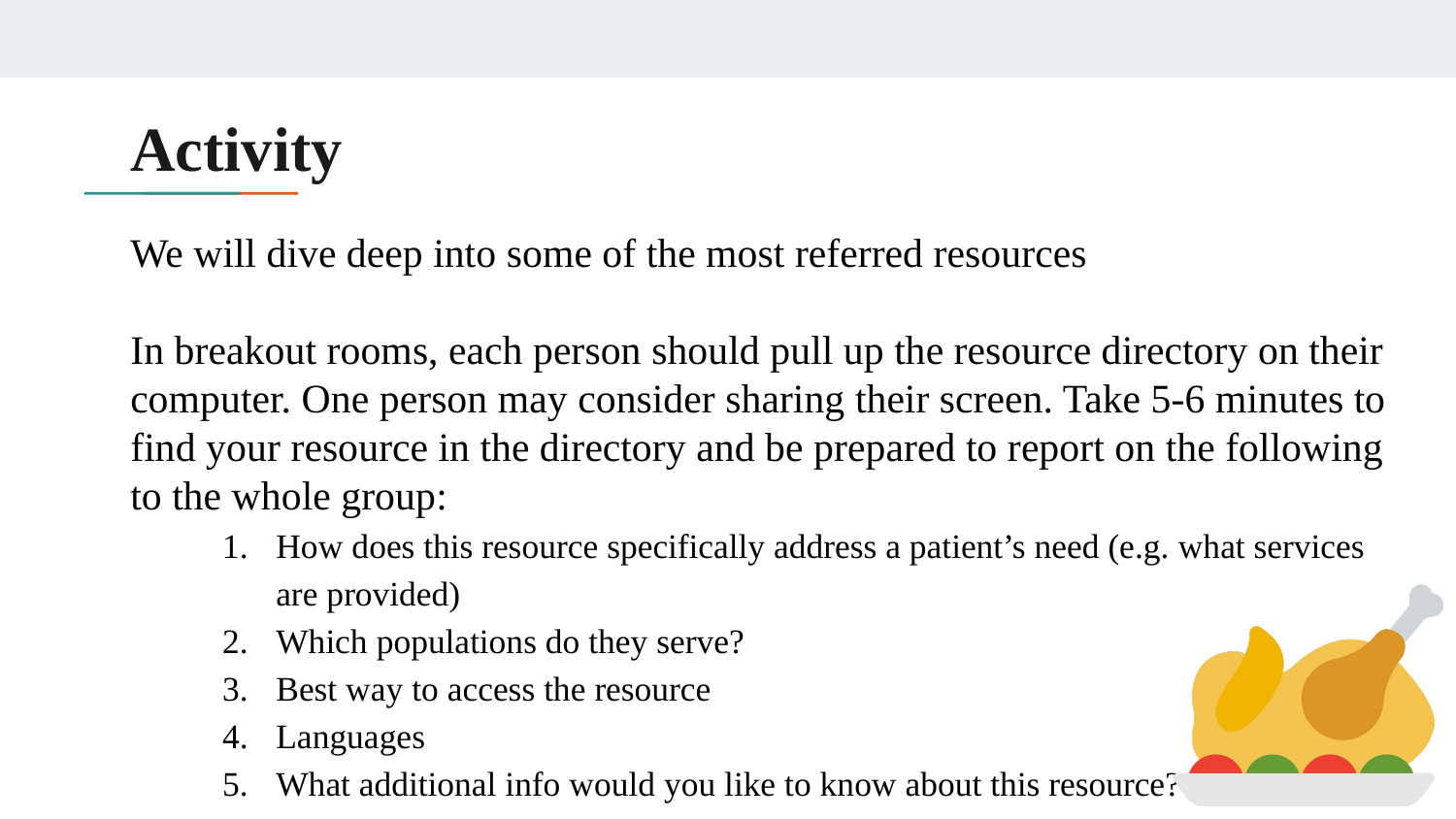

# Activity
We will dive deep into some of the most referred resources
In breakout rooms, each person should pull up the resource directory on their computer. One person may consider sharing their screen. Take 5-6 minutes to find your resource in the directory and be prepared to report on the following to the whole group:
How does this resource specifically address a patient’s need (e.g. what services are provided)
Which populations do they serve?
Best way to access the resource
Languages
What additional info would you like to know about this resource?

## Slide 36
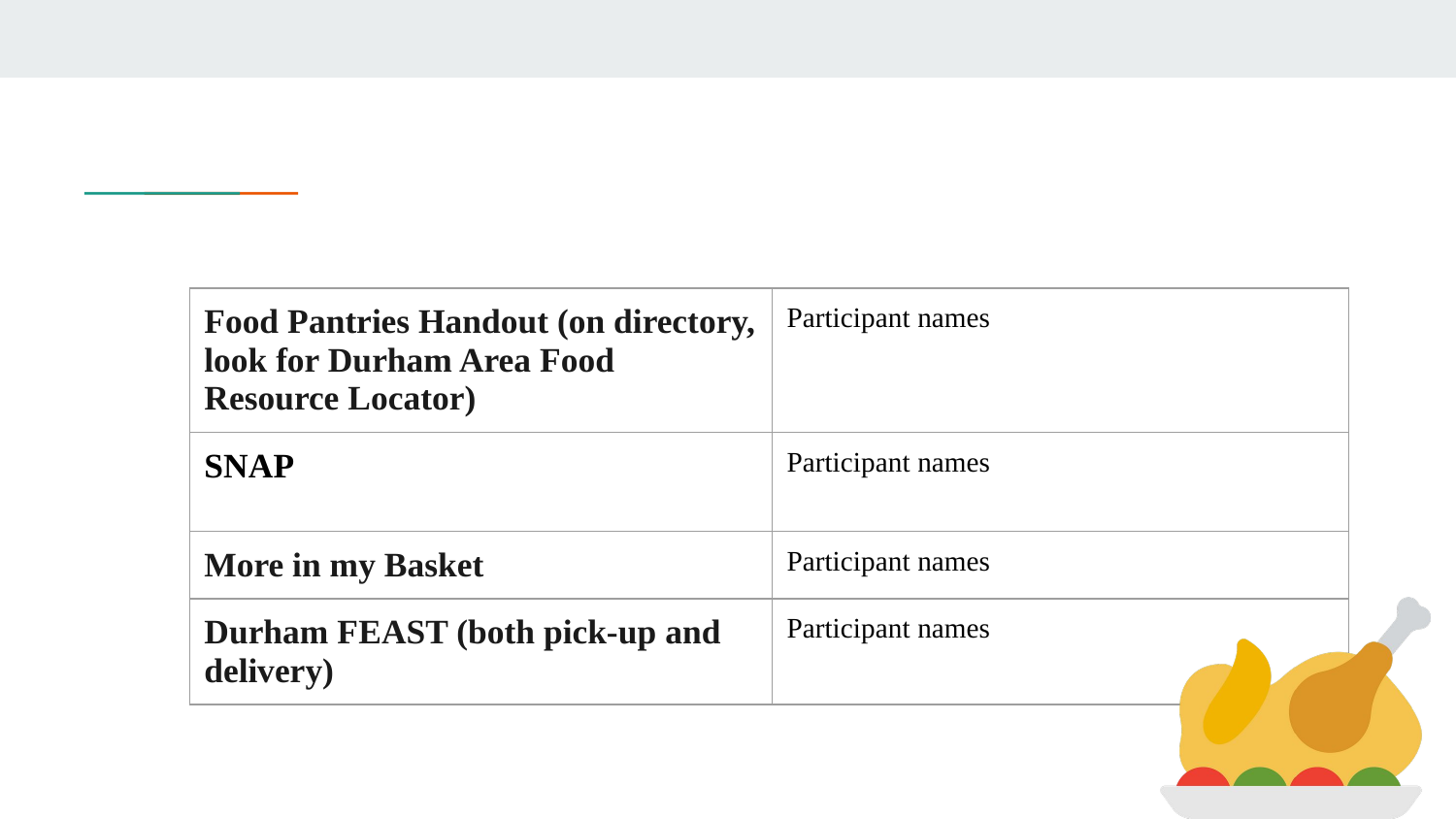

| Food Pantries Handout (on directory, look for Durham Area Food Resource Locator) | Participant names |
| --- | --- |
| SNAP | Participant names |
| More in my Basket | Participant names |
| Durham FEAST (both pick-up and delivery) | Participant names |

## Slide 37
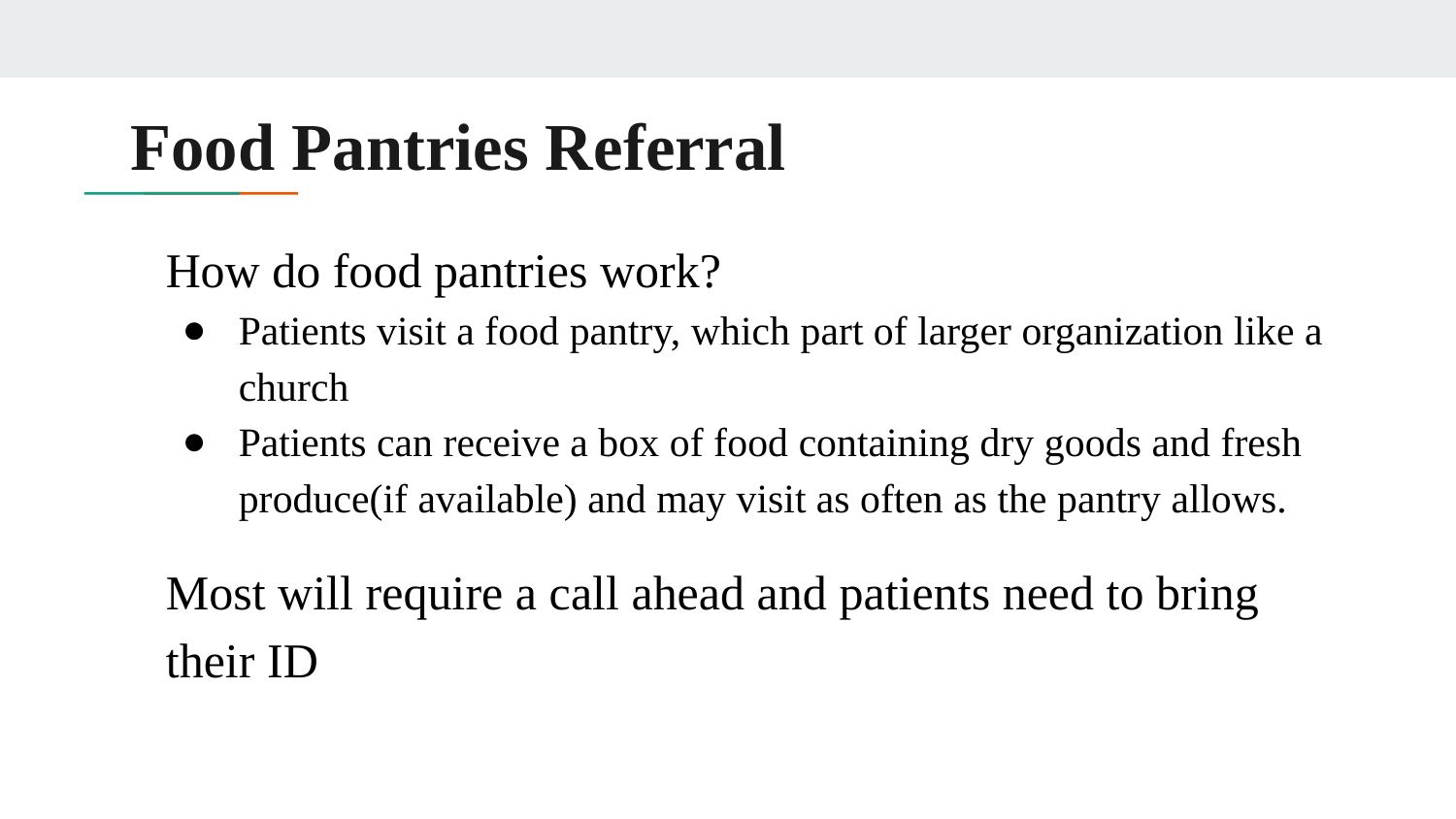

# Food Pantries Referral
How do food pantries work?
Patients visit a food pantry, which part of larger organization like a church
Patients can receive a box of food containing dry goods and fresh produce(if available) and may visit as often as the pantry allows.
Most will require a call ahead and patients need to bring their ID

## Slide 38
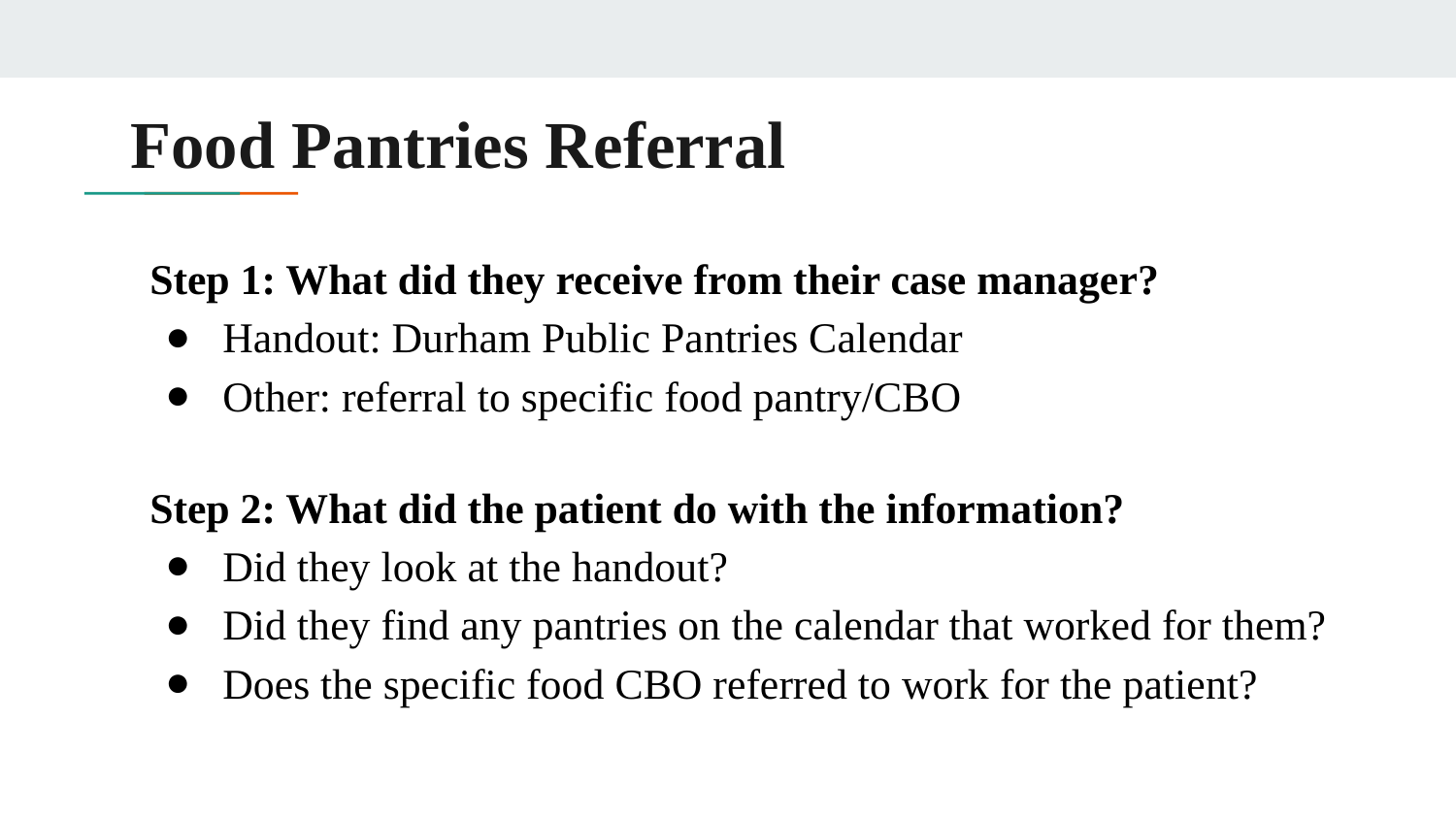

# Food Pantries Referral
Step 1: What did they receive from their case manager?
Handout: Durham Public Pantries Calendar
Other: referral to specific food pantry/CBO
Step 2: What did the patient do with the information?
Did they look at the handout?
Did they find any pantries on the calendar that worked for them?
Does the specific food CBO referred to work for the patient?

## Slide 39
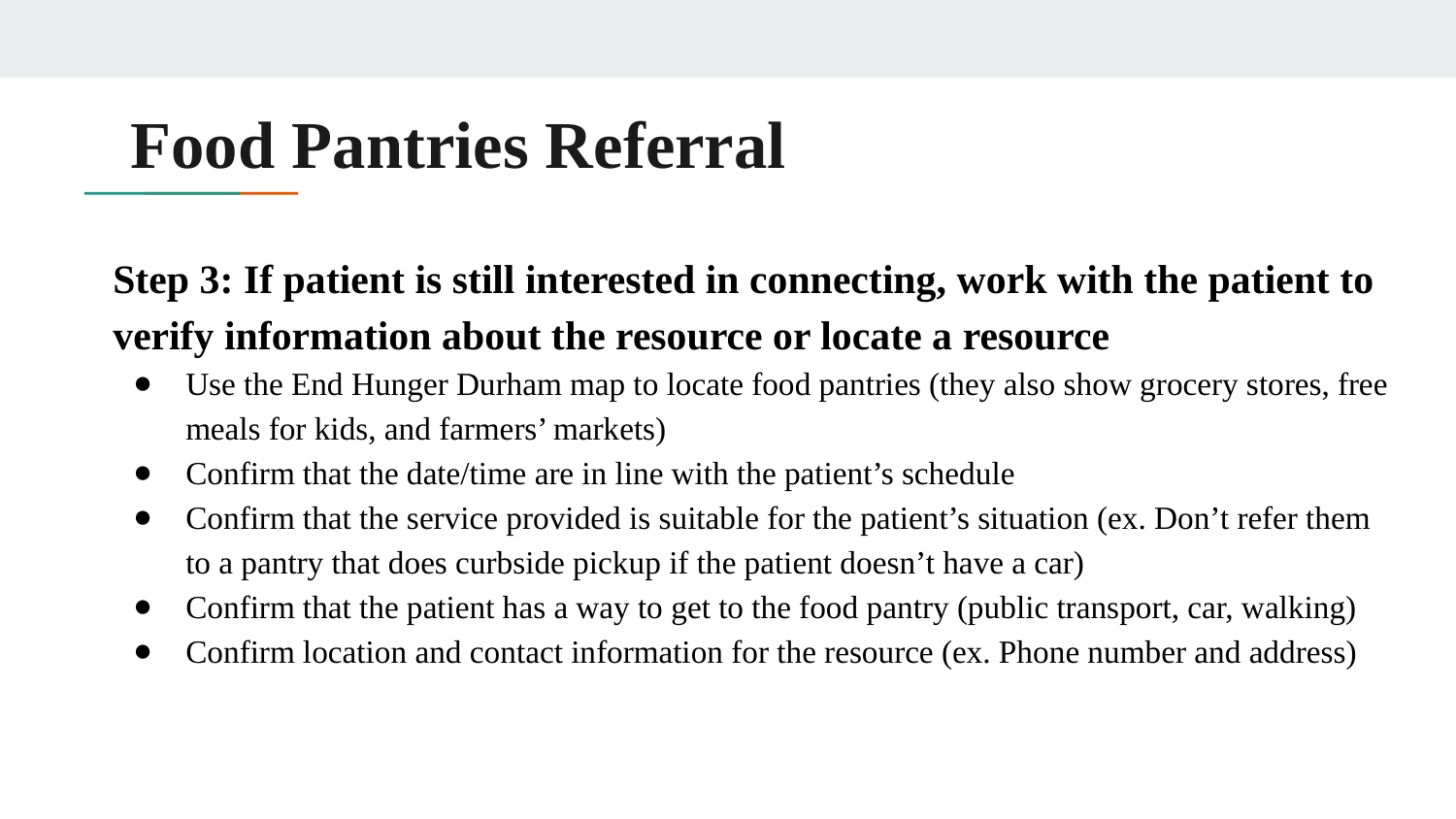

# Food Pantries Referral
Step 3: If patient is still interested in connecting, work with the patient to verify information about the resource or locate a resource
Use the End Hunger Durham map to locate food pantries (they also show grocery stores, free meals for kids, and farmers’ markets)
Confirm that the date/time are in line with the patient’s schedule
Confirm that the service provided is suitable for the patient’s situation (ex. Don’t refer them to a pantry that does curbside pickup if the patient doesn’t have a car)
Confirm that the patient has a way to get to the food pantry (public transport, car, walking)
Confirm location and contact information for the resource (ex. Phone number and address)

## Slide 40
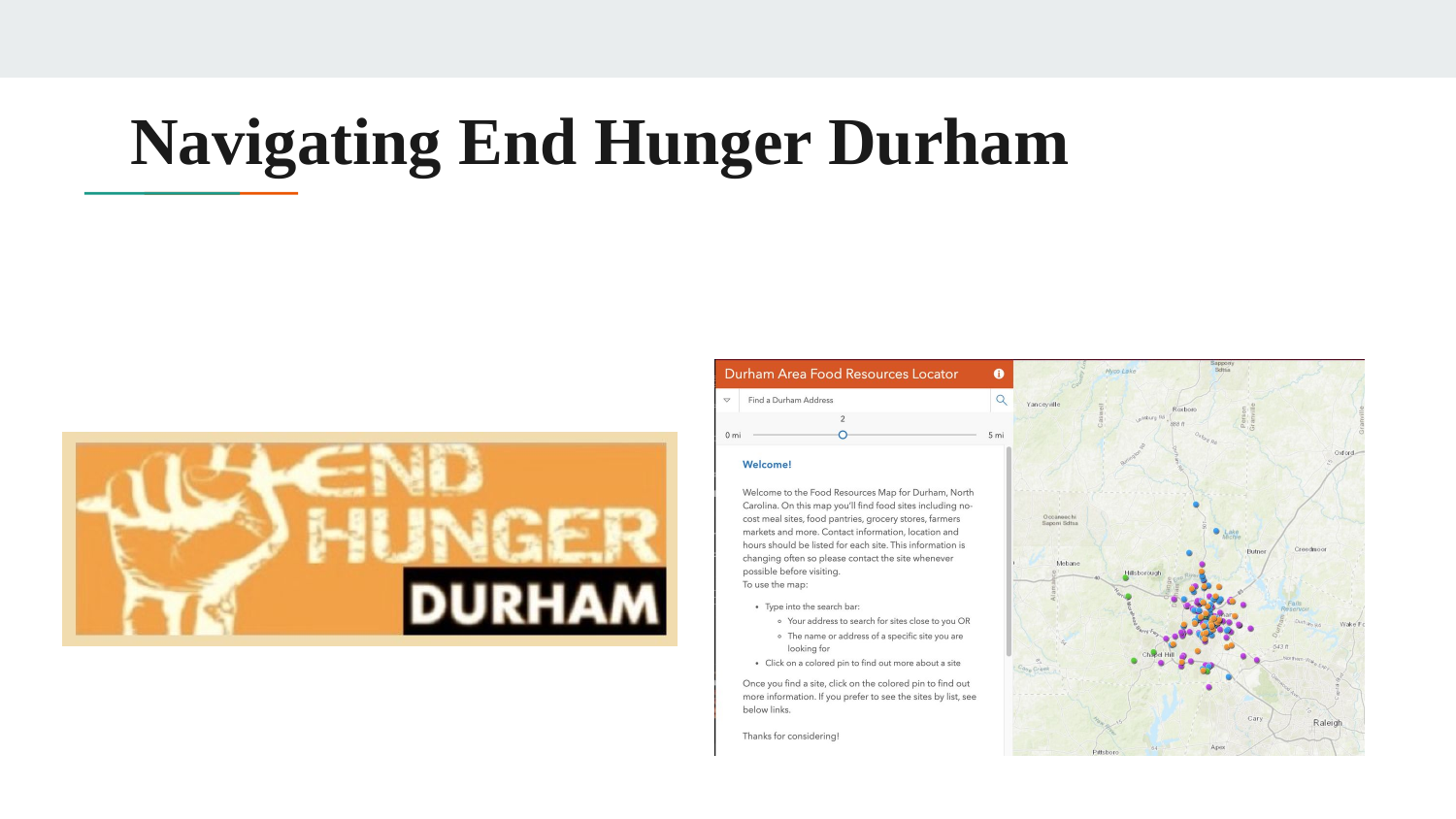

# Navigating End Hunger Durham

## Slide 41
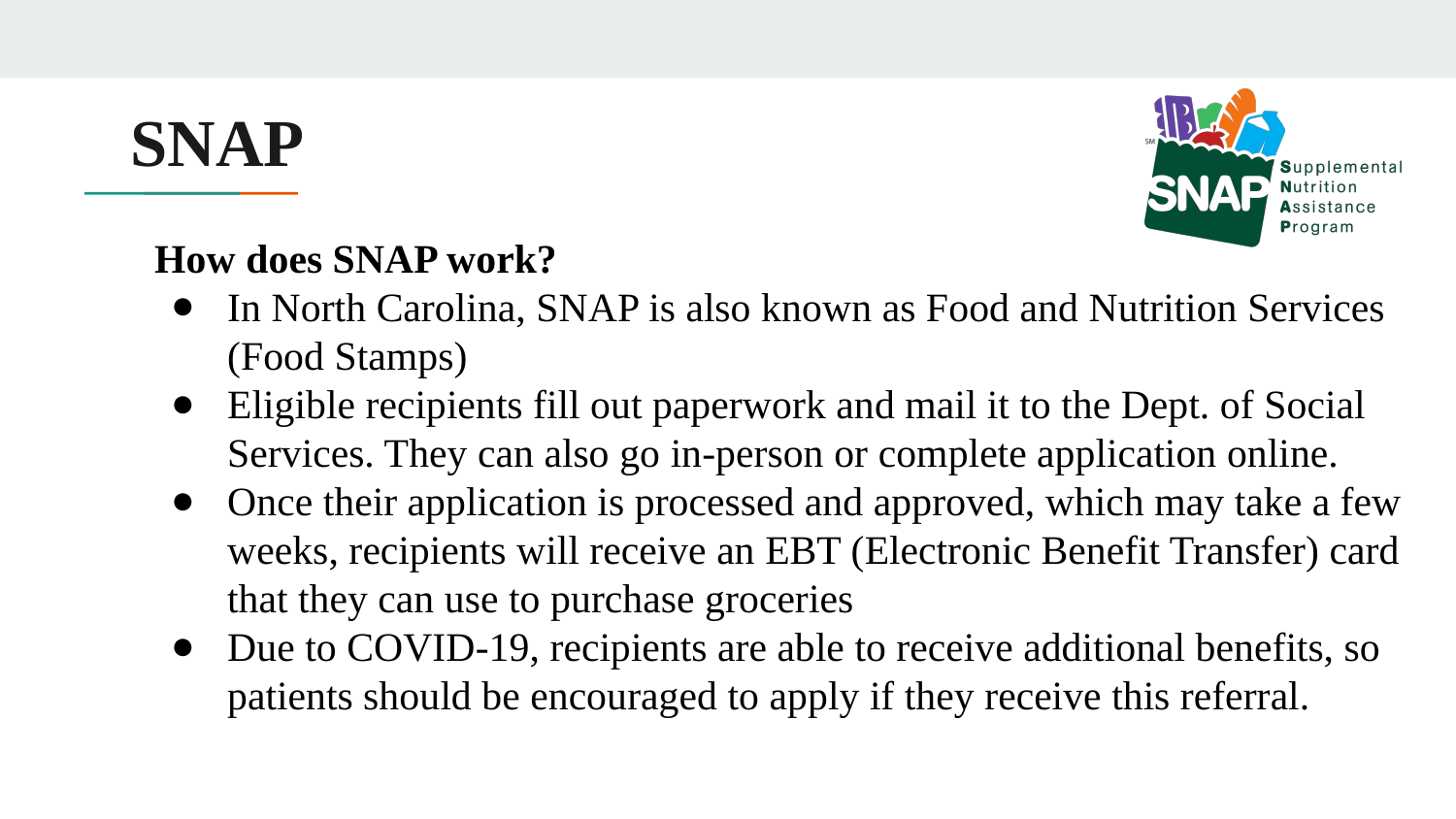

# SNAP
How does SNAP work?
In North Carolina, SNAP is also known as Food and Nutrition Services (Food Stamps)
Eligible recipients fill out paperwork and mail it to the Dept. of Social Services. They can also go in-person or complete application online.
Once their application is processed and approved, which may take a few weeks, recipients will receive an EBT (Electronic Benefit Transfer) card that they can use to purchase groceries
Due to COVID-19, recipients are able to receive additional benefits, so patients should be encouraged to apply if they receive this referral.

## Slide 42
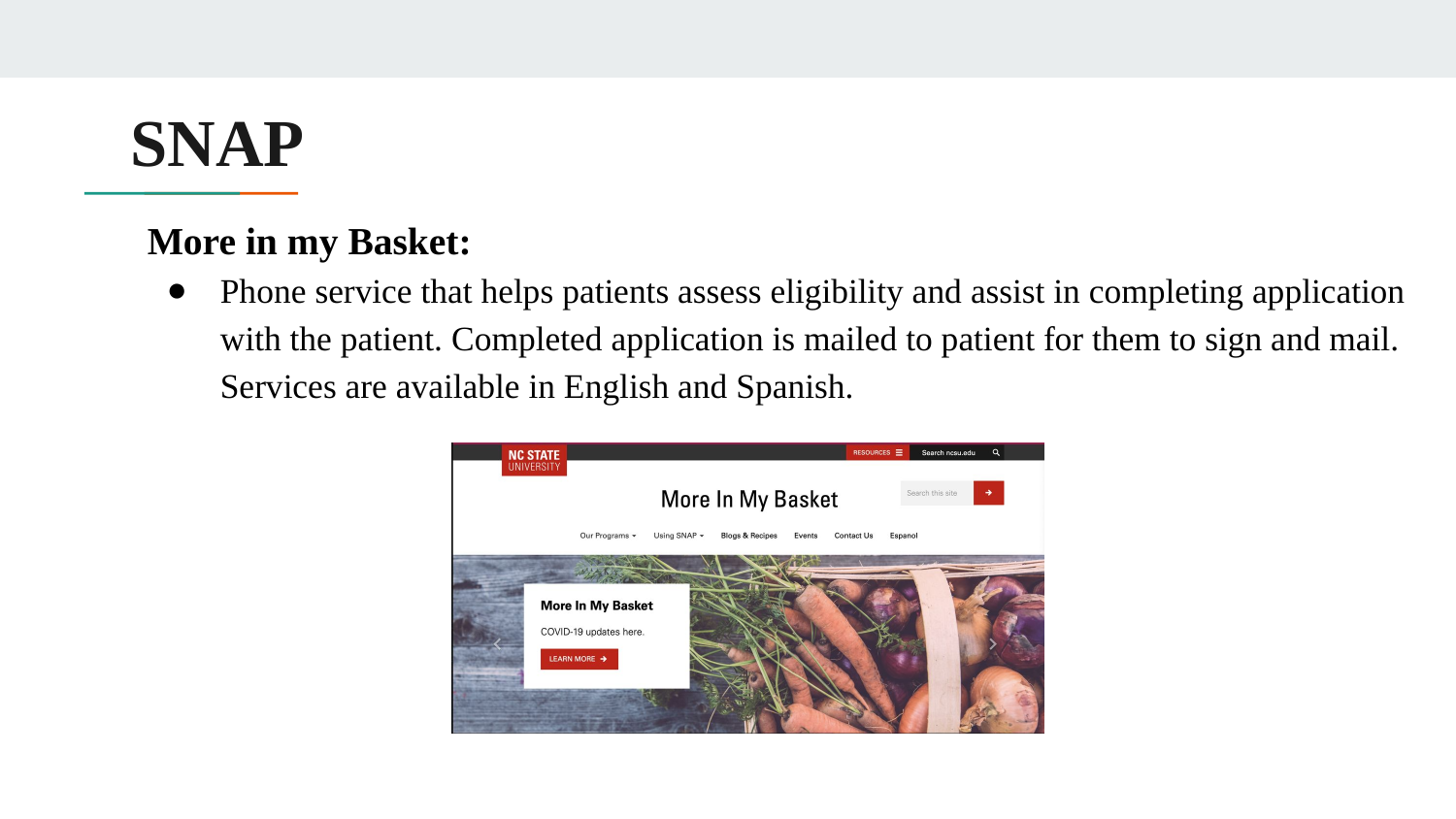

# SNAP
More in my Basket:
Phone service that helps patients assess eligibility and assist in completing application with the patient. Completed application is mailed to patient for them to sign and mail. Services are available in English and Spanish.

## Slide 43
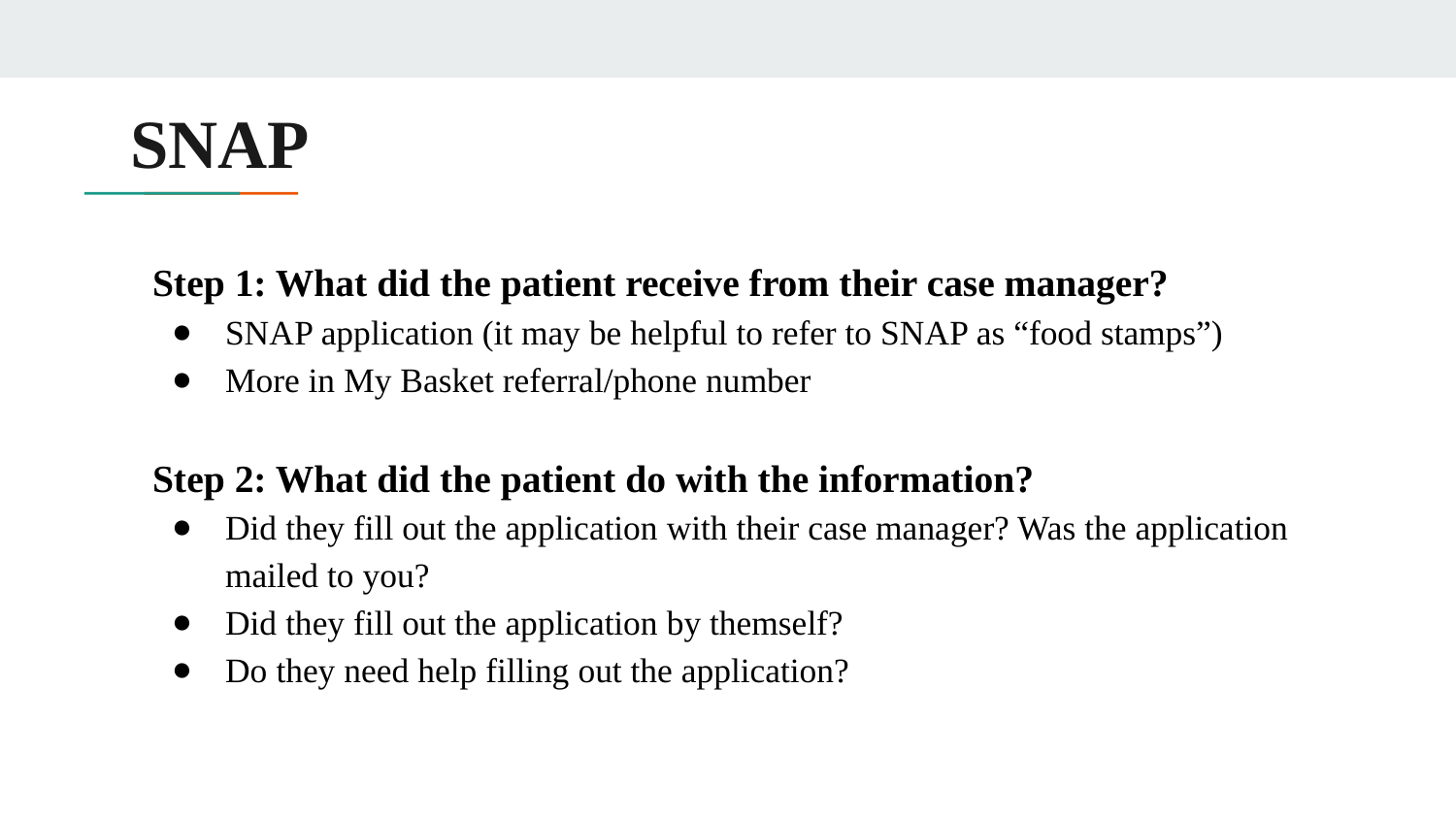

# SNAP
Step 1: What did the patient receive from their case manager?
SNAP application (it may be helpful to refer to SNAP as “food stamps”)
More in My Basket referral/phone number
Step 2: What did the patient do with the information?
Did they fill out the application with their case manager? Was the application mailed to you?
Did they fill out the application by themself?
Do they need help filling out the application?

## Slide 44
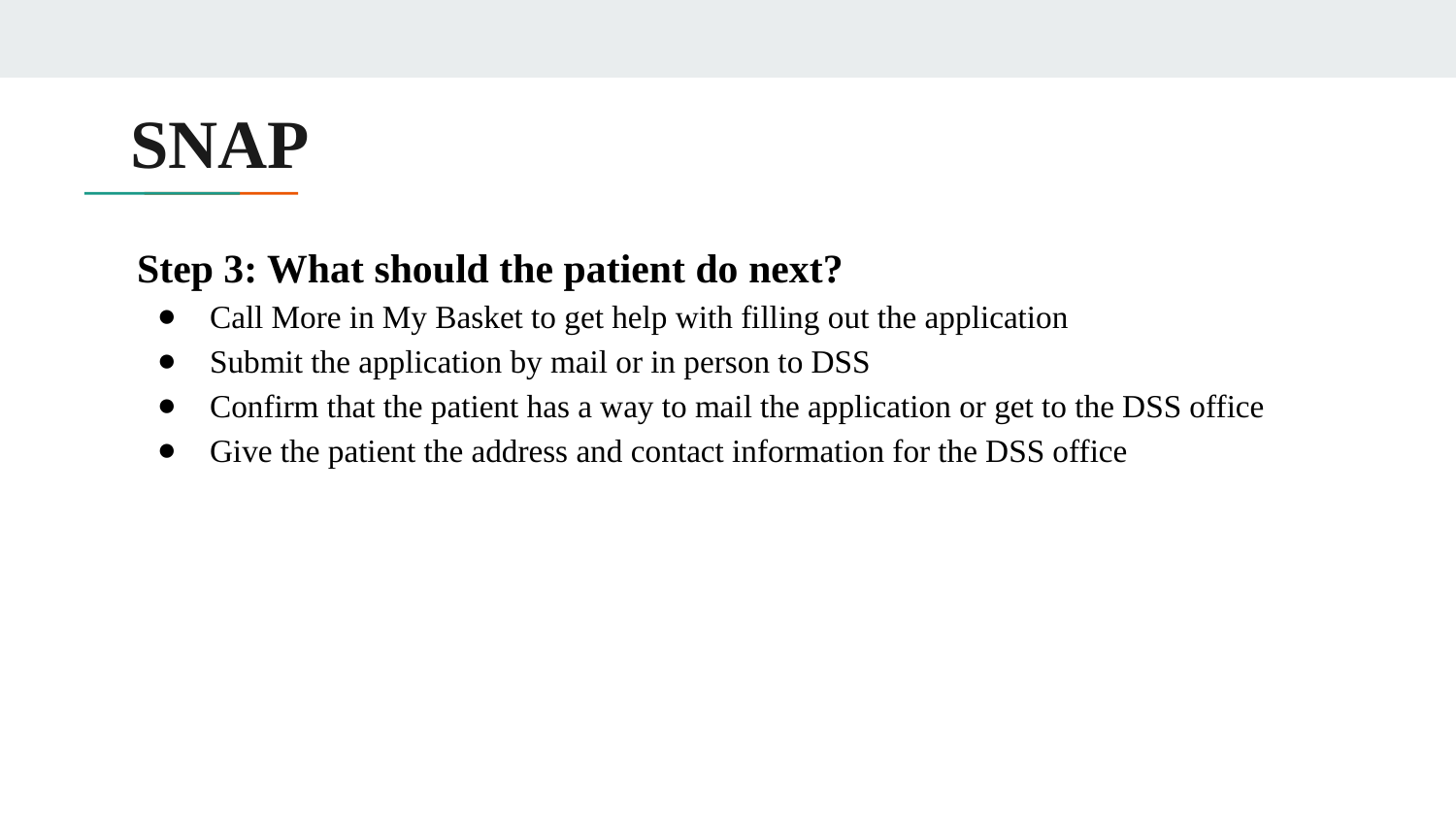

# SNAP
Step 3: What should the patient do next?
Call More in My Basket to get help with filling out the application
Submit the application by mail or in person to DSS
Confirm that the patient has a way to mail the application or get to the DSS office
Give the patient the address and contact information for the DSS office

## Slide 45
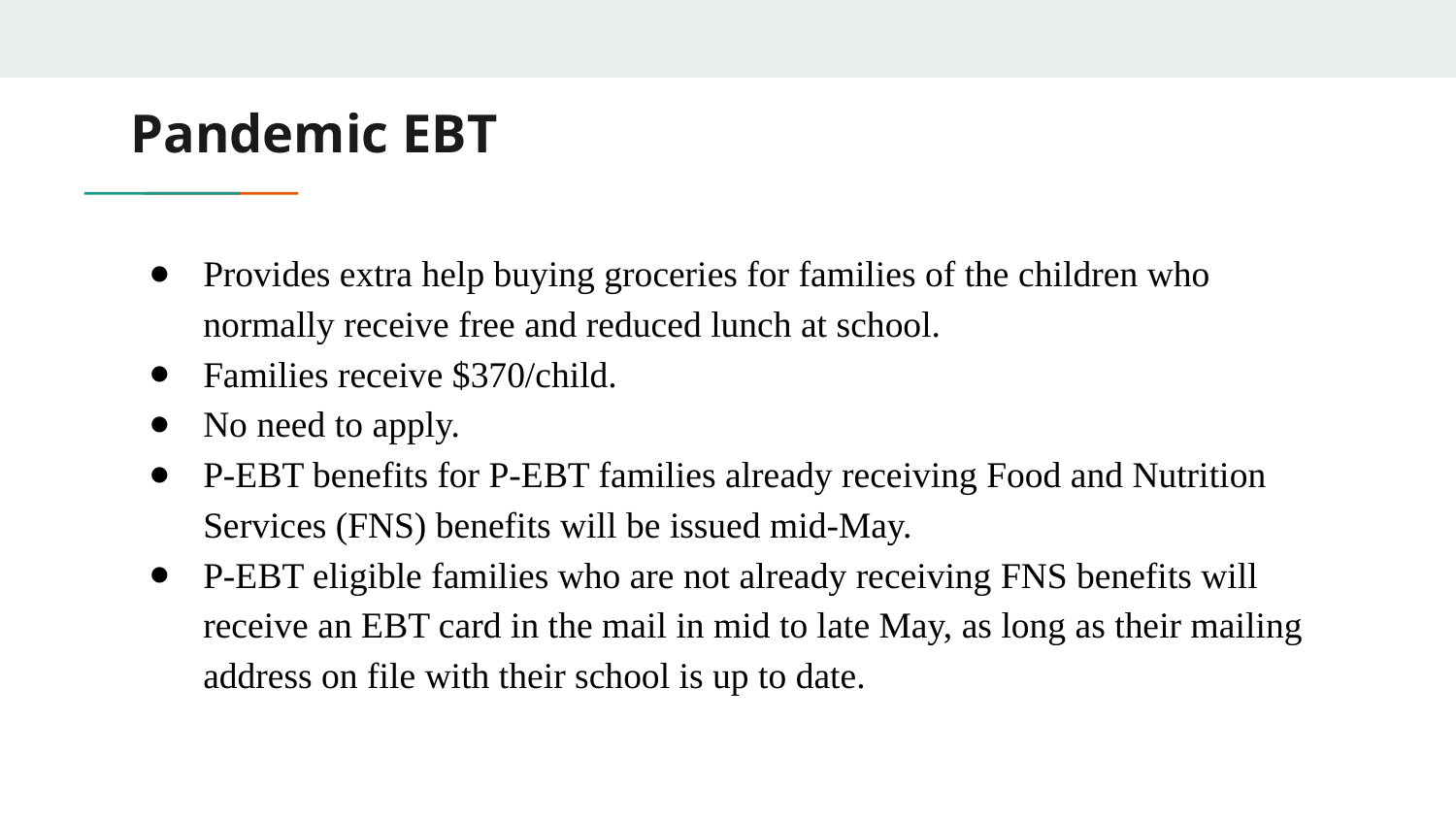

# Pandemic EBT
Provides extra help buying groceries for families of the children who normally receive free and reduced lunch at school.
Families receive $370/child.
No need to apply.
P-EBT benefits for P-EBT families already receiving Food and Nutrition Services (FNS) benefits will be issued mid-May.
P-EBT eligible families who are not already receiving FNS benefits will receive an EBT card in the mail in mid to late May, as long as their mailing address on file with their school is up to date.

## Slide 46
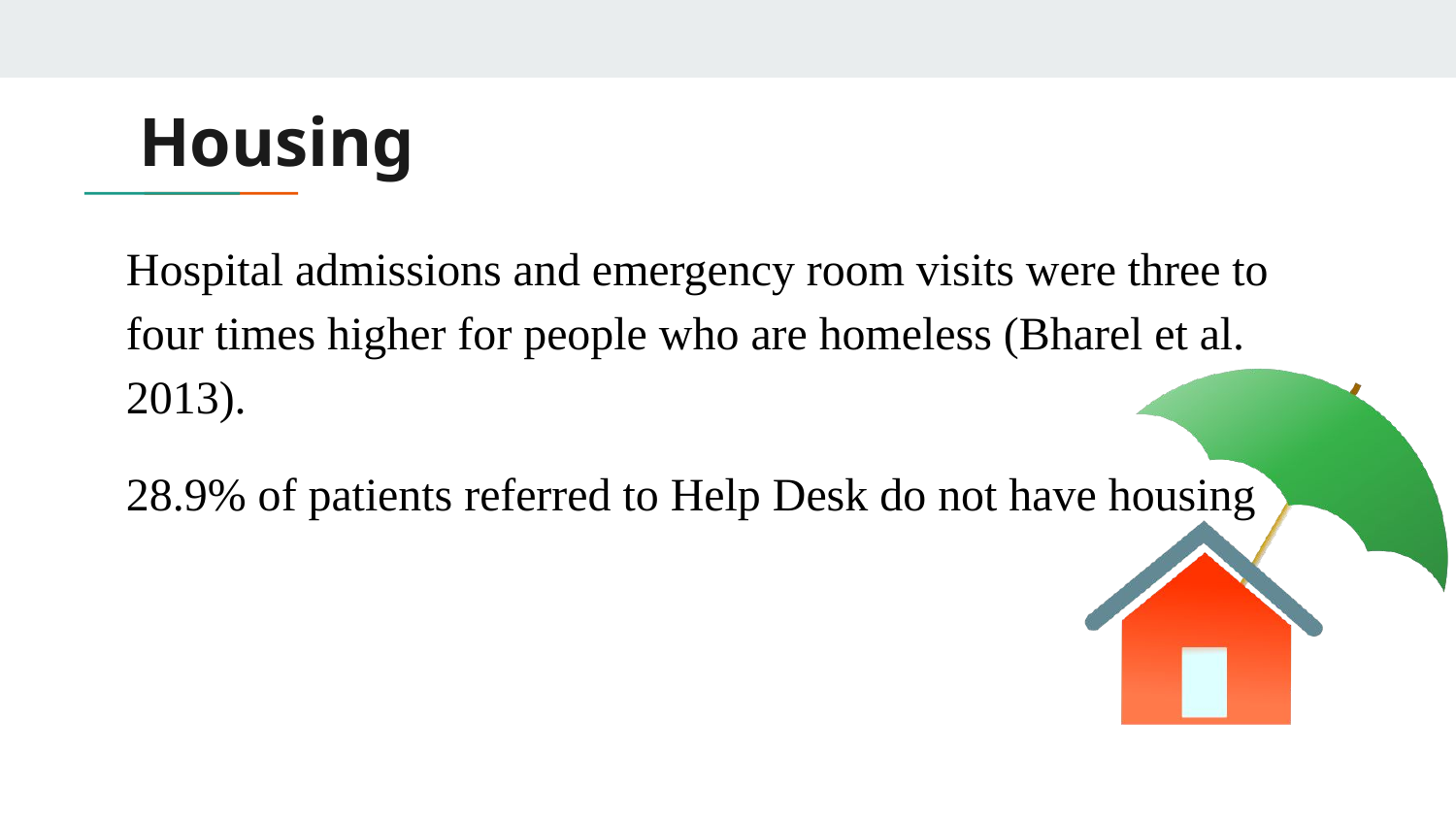

# Housing
Hospital admissions and emergency room visits were three to four times higher for people who are homeless (Bharel et al. 2013).
28.9% of patients referred to Help Desk do not have housing

## Slide 47
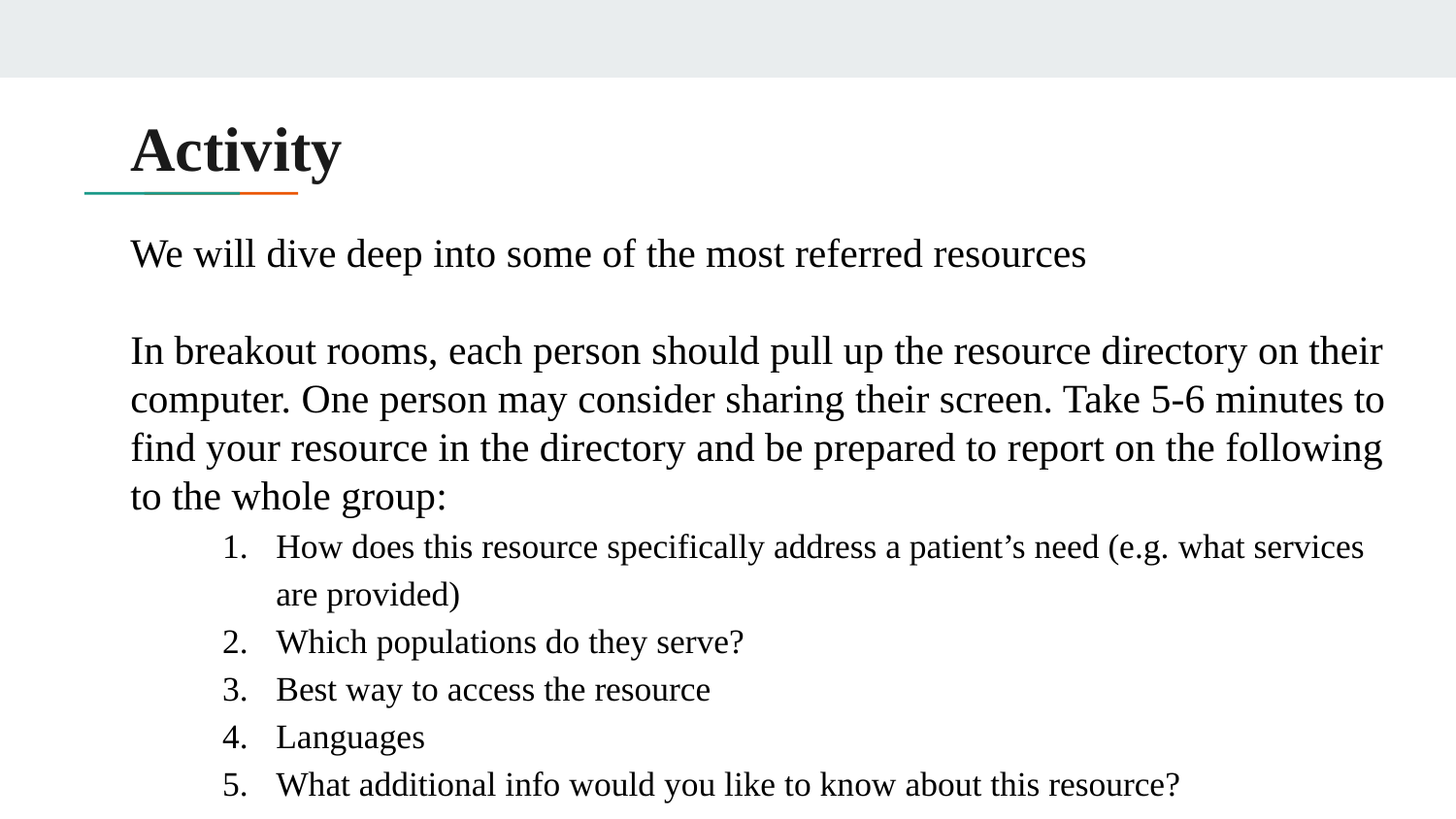

# Activity
We will dive deep into some of the most referred resources
In breakout rooms, each person should pull up the resource directory on their computer. One person may consider sharing their screen. Take 5-6 minutes to find your resource in the directory and be prepared to report on the following to the whole group:
How does this resource specifically address a patient’s need (e.g. what services are provided)
Which populations do they serve?
Best way to access the resource
Languages
What additional info would you like to know about this resource?

## Slide 48
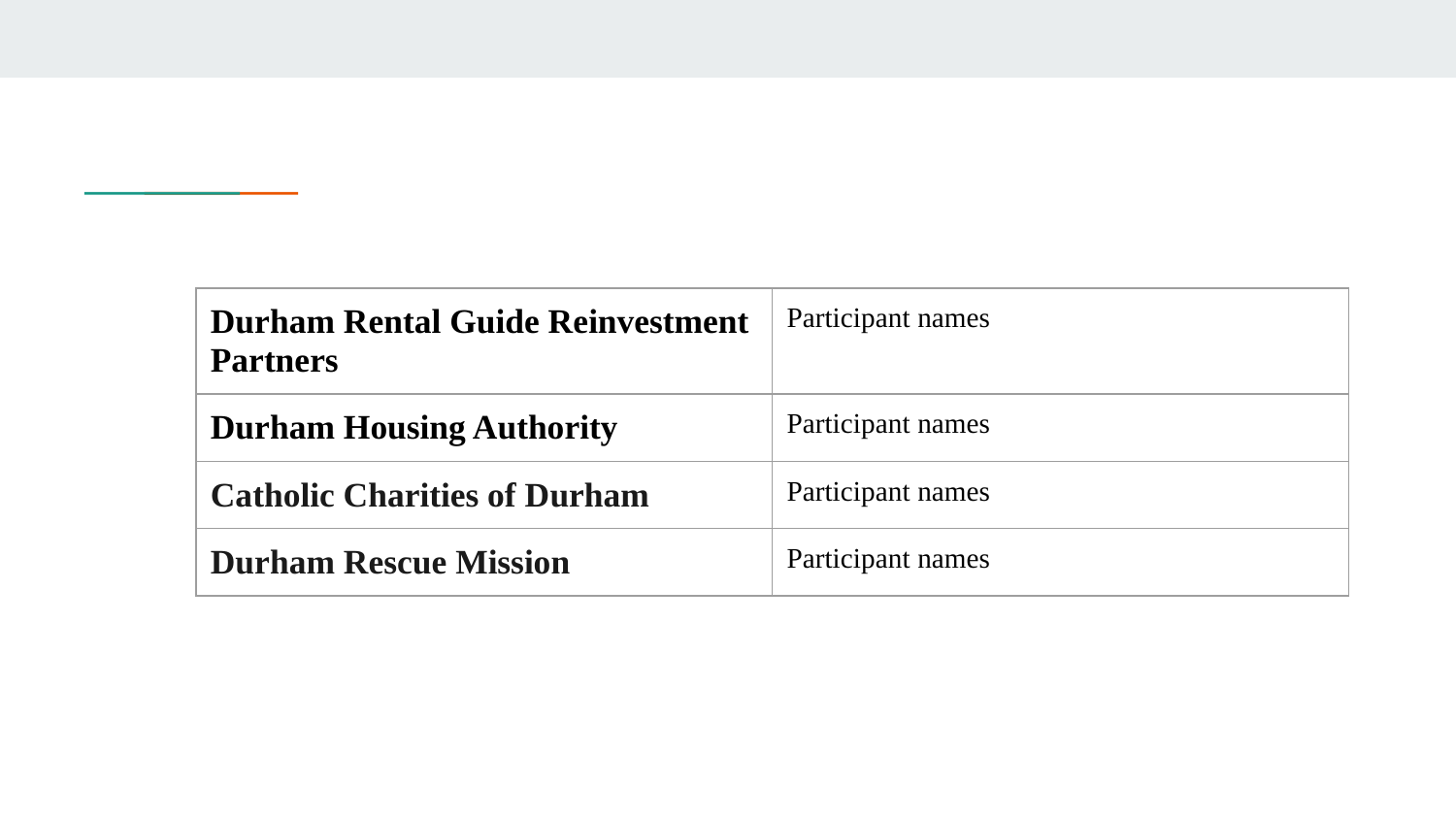

| Durham Rental Guide Reinvestment Partners | Participant names |
| --- | --- |
| Durham Housing Authority | Participant names |
| Catholic Charities of Durham | Participant names |
| Durham Rescue Mission | Participant names |

## Slide 49
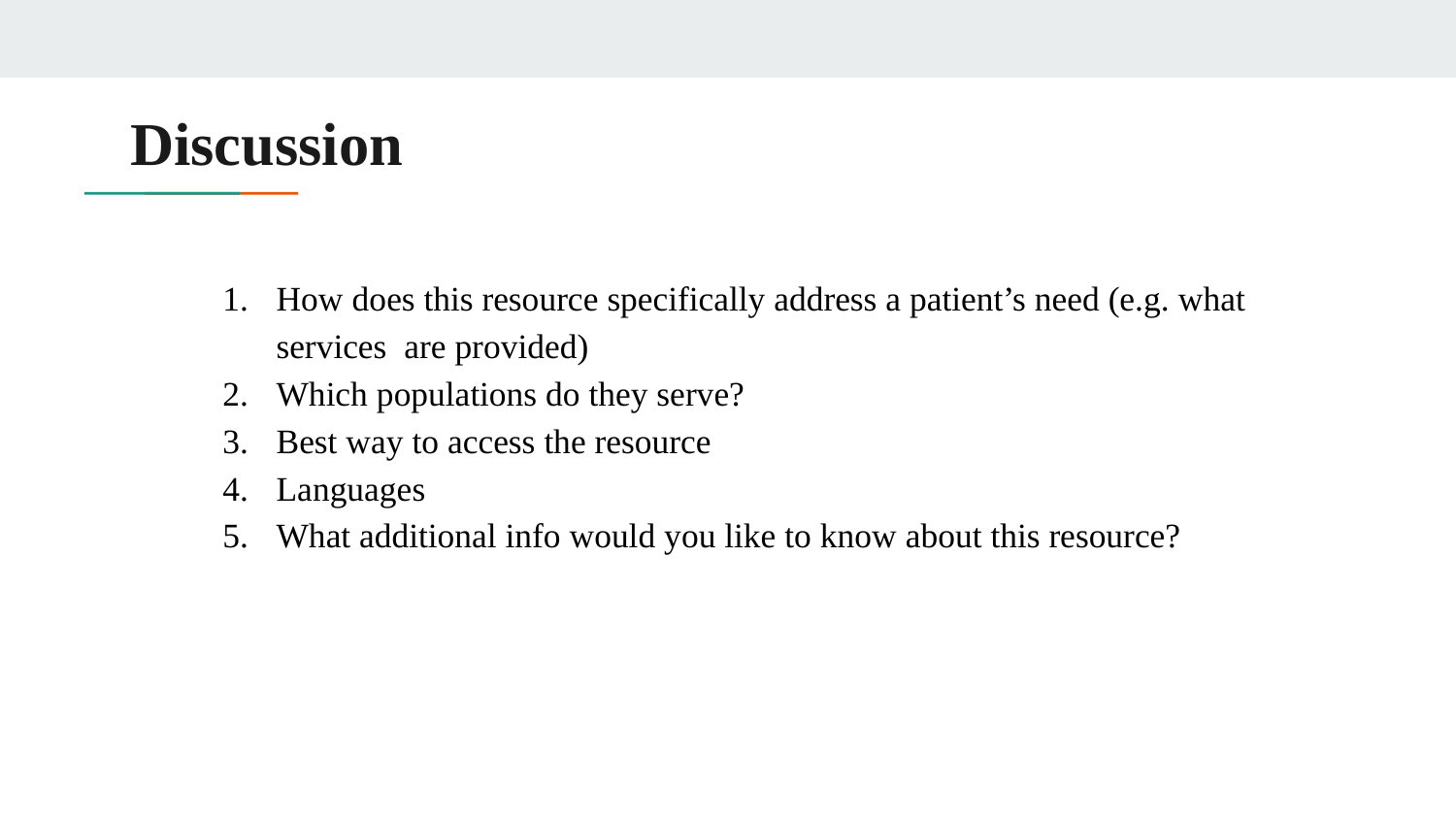

# Discussion
How does this resource specifically address a patient’s need (e.g. what services are provided)
Which populations do they serve?
Best way to access the resource
Languages
What additional info would you like to know about this resource?

## Slide 50
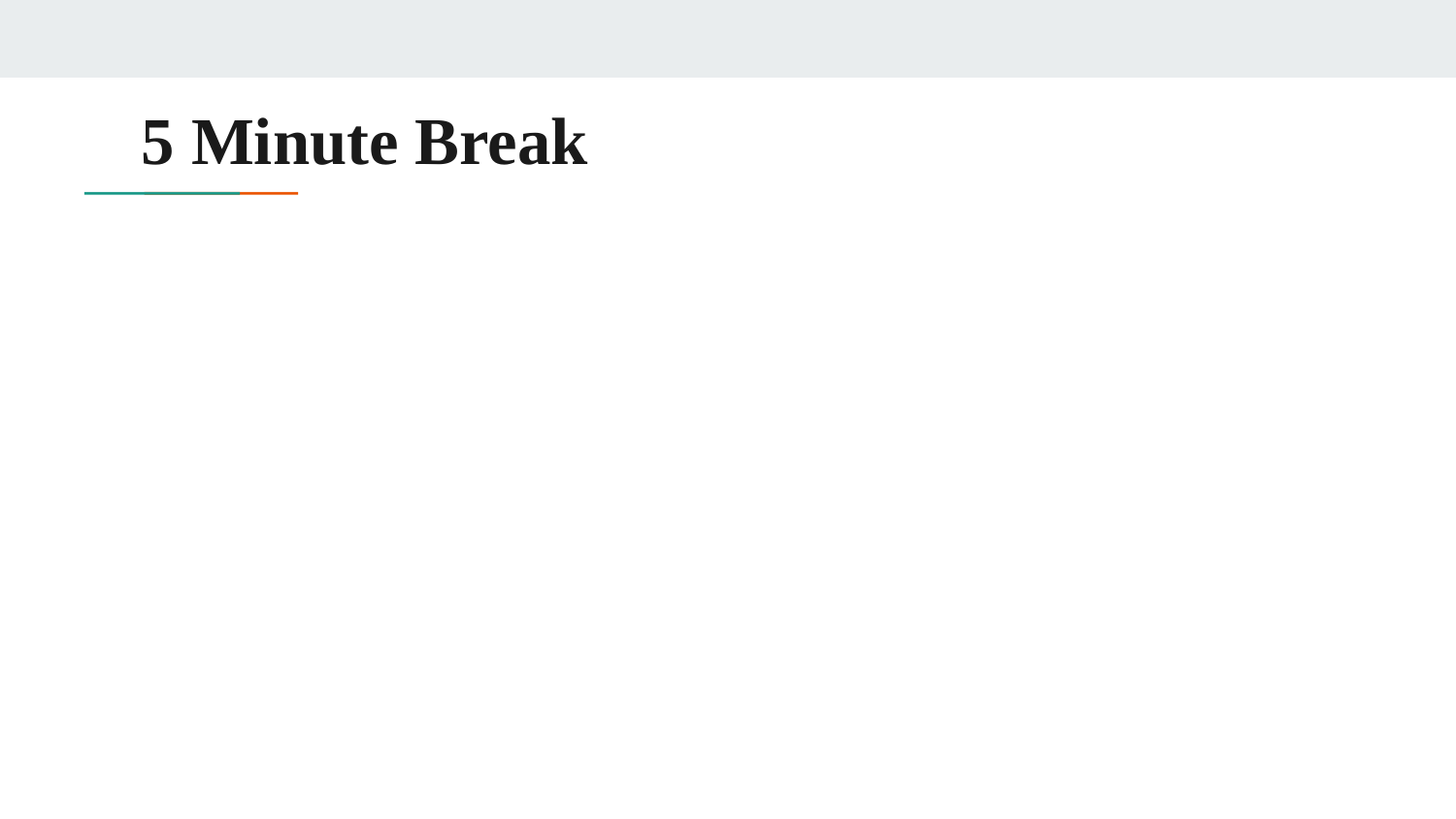

# 5 Minute Break

## Slide 51
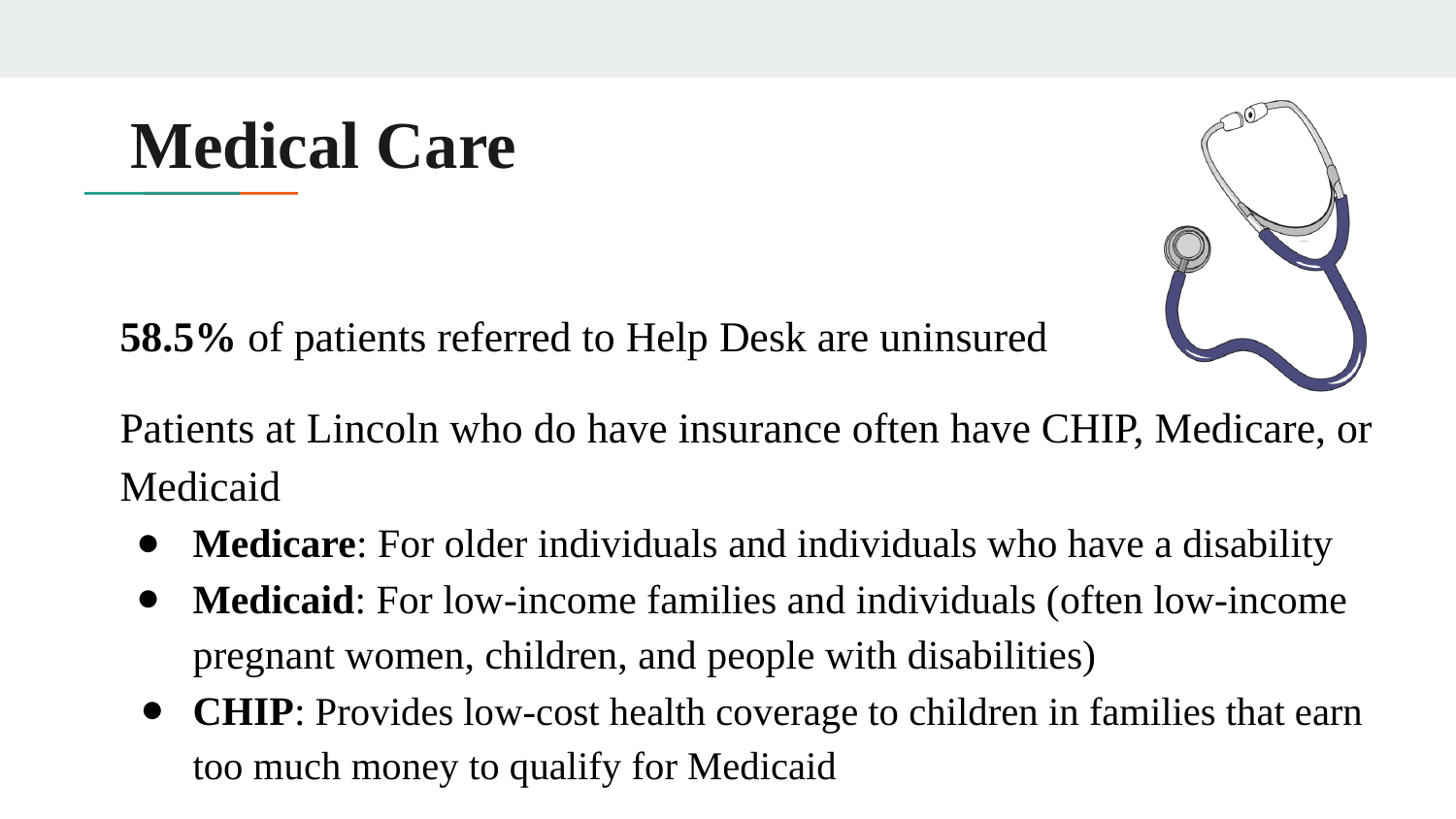

# Medical Care
58.5% of patients referred to Help Desk are uninsured
Patients at Lincoln who do have insurance often have CHIP, Medicare, or Medicaid
Medicare: For older individuals and individuals who have a disability
Medicaid: For low-income families and individuals (often low-income pregnant women, children, and people with disabilities)
CHIP: Provides low-cost health coverage to children in families that earn too much money to qualify for Medicaid

## Slide 52
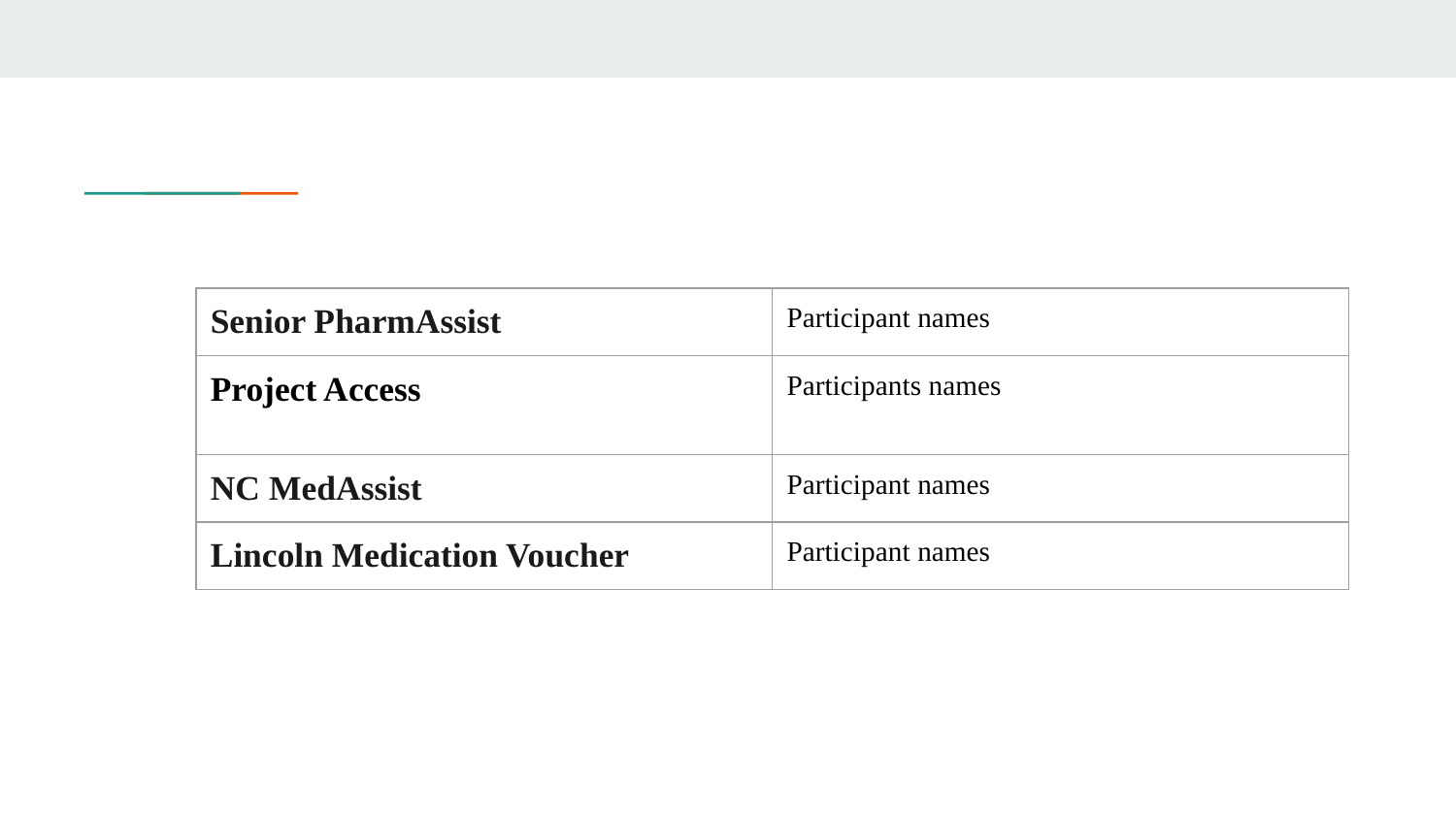

| Senior PharmAssist | Participant names |
| --- | --- |
| Project Access | Participants names |
| NC MedAssist | Participant names |
| Lincoln Medication Voucher | Participant names |

## Slide 53
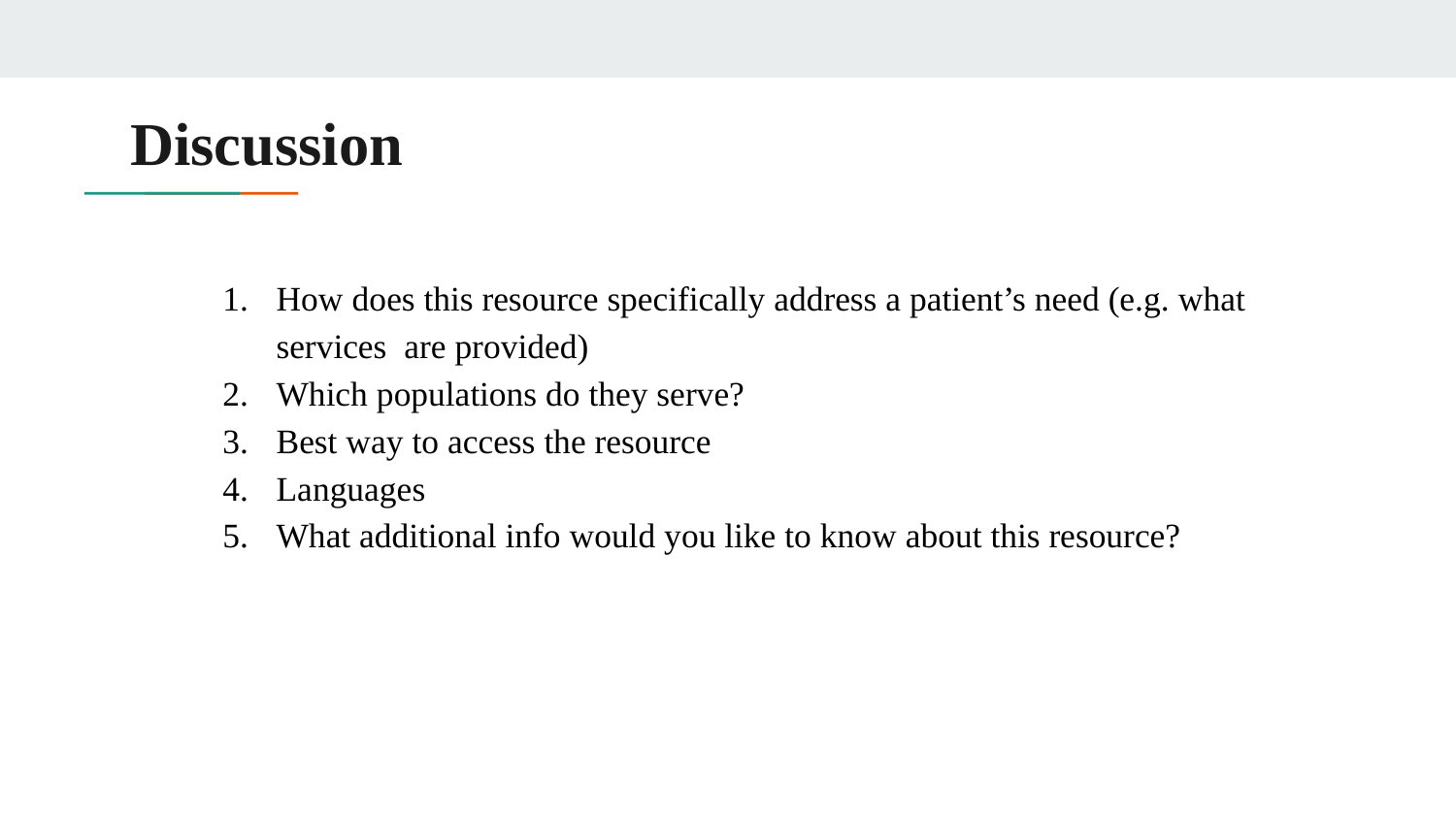

# Discussion
How does this resource specifically address a patient’s need (e.g. what services are provided)
Which populations do they serve?
Best way to access the resource
Languages
What additional info would you like to know about this resource?

## Slide 54
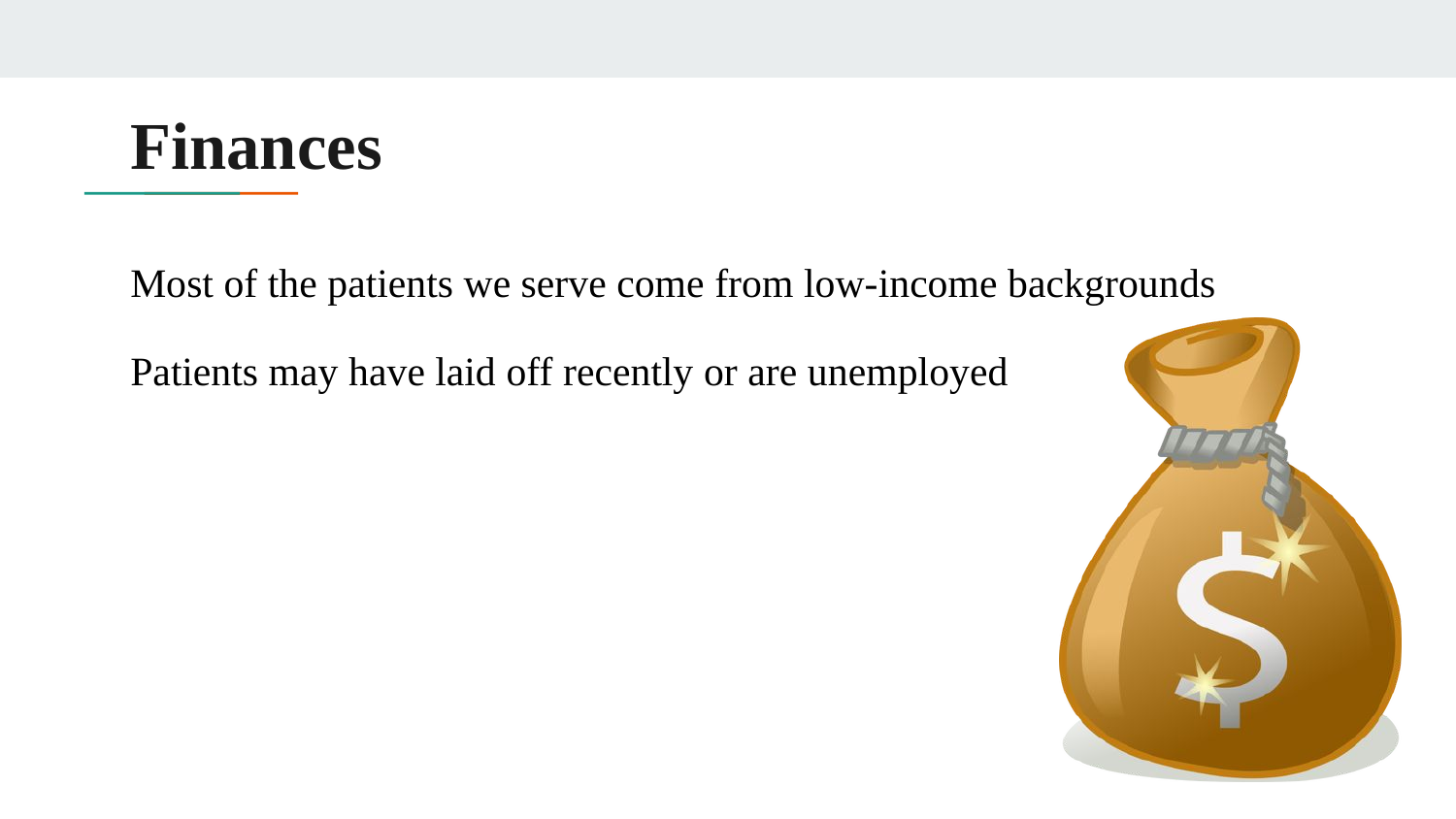

# Finances
Most of the patients we serve come from low-income backgrounds
Patients may have laid off recently or are unemployed

## Slide 55
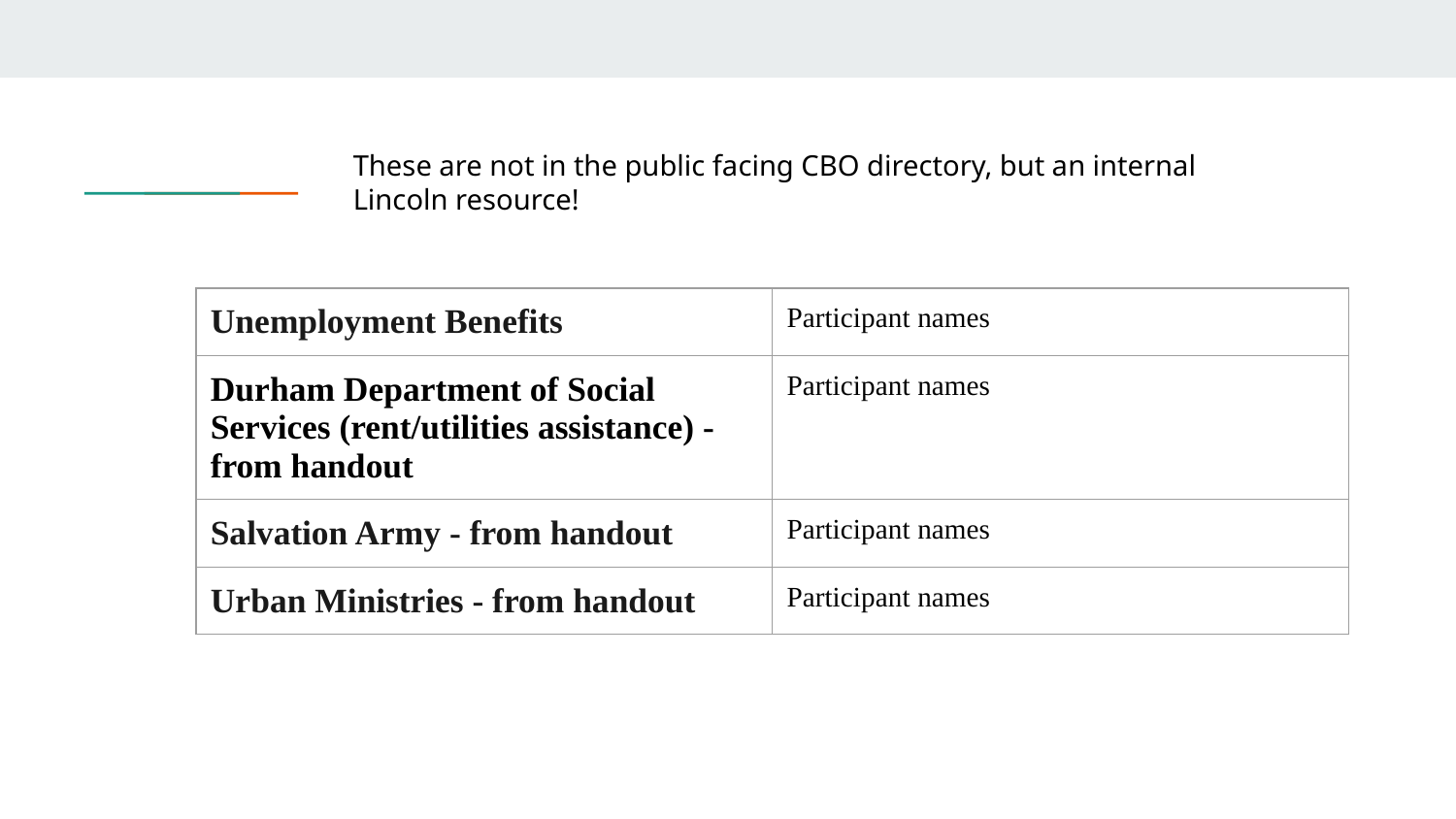

These are not in the public facing CBO directory, but an internal Lincoln resource!
| Unemployment Benefits | Participant names |
| --- | --- |
| Durham Department of Social Services (rent/utilities assistance) - from handout | Participant names |
| Salvation Army - from handout | Participant names |
| Urban Ministries - from handout | Participant names |

## Slide 56
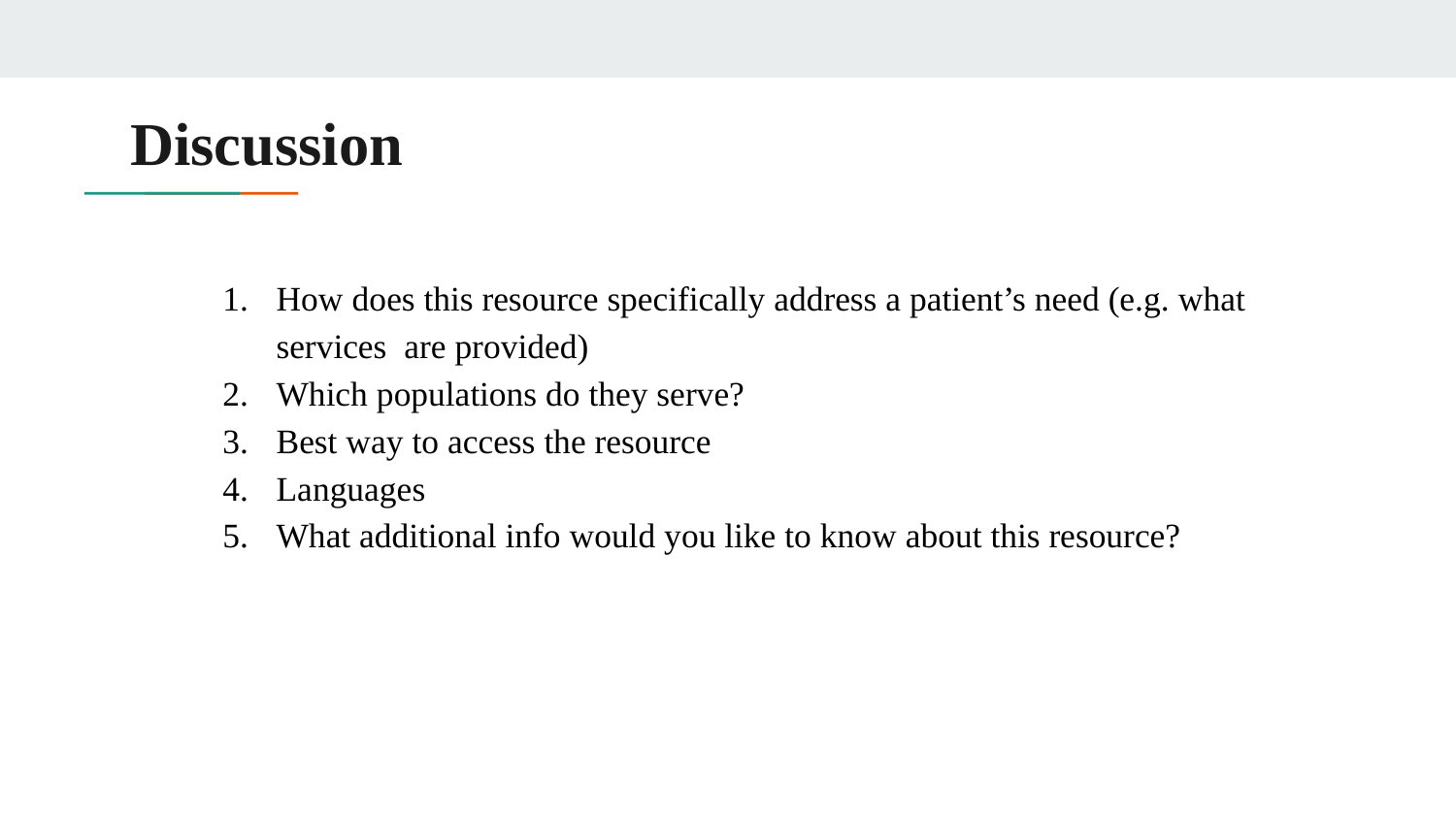

# Discussion
How does this resource specifically address a patient’s need (e.g. what services are provided)
Which populations do they serve?
Best way to access the resource
Languages
What additional info would you like to know about this resource?

## Slide 57
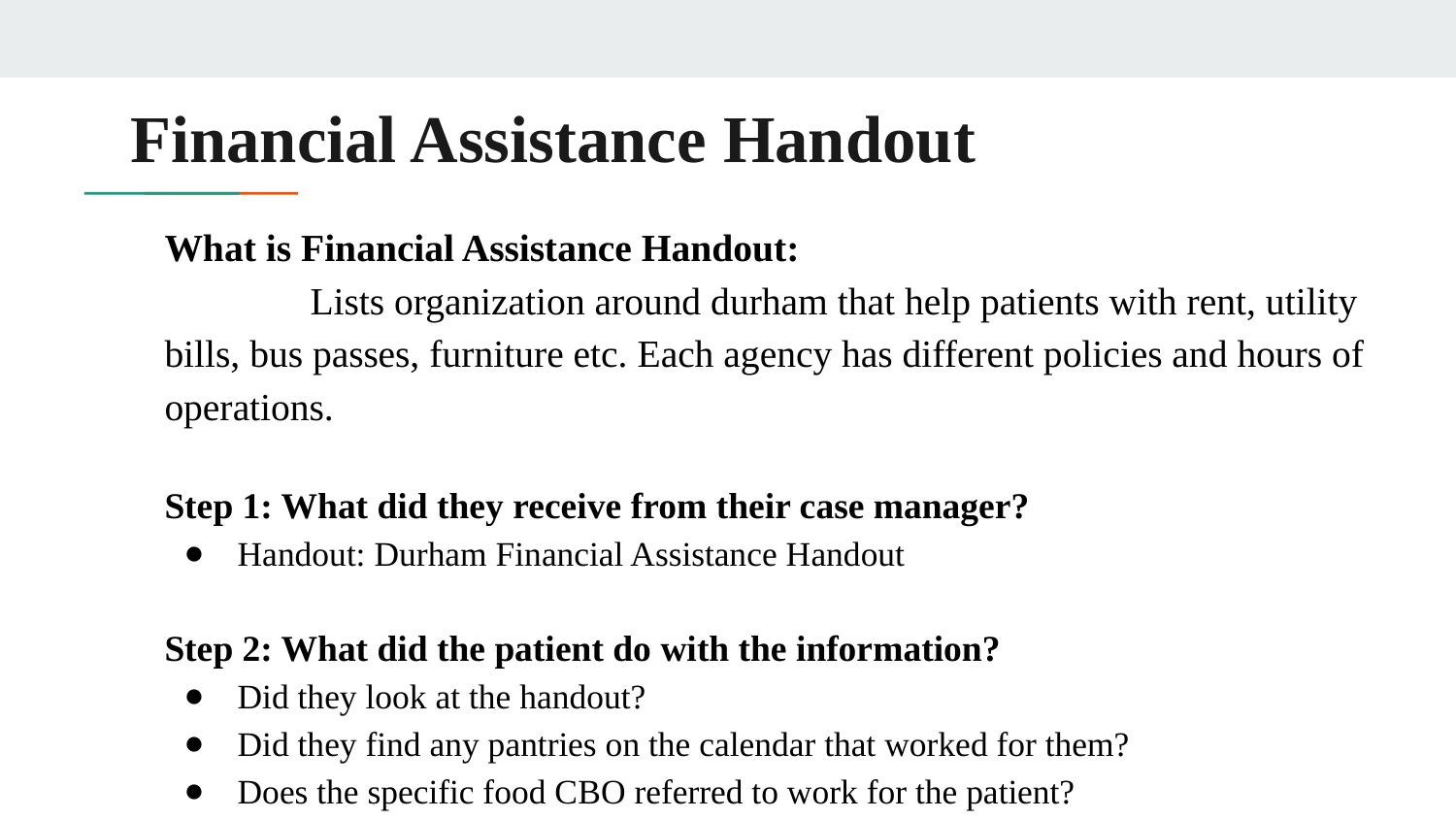

# Financial Assistance Handout
What is Financial Assistance Handout:
	Lists organization around durham that help patients with rent, utility bills, bus passes, furniture etc. Each agency has different policies and hours of operations.
Step 1: What did they receive from their case manager?
Handout: Durham Financial Assistance Handout
Step 2: What did the patient do with the information?
Did they look at the handout?
Did they find any pantries on the calendar that worked for them?
Does the specific food CBO referred to work for the patient?

## Slide 58
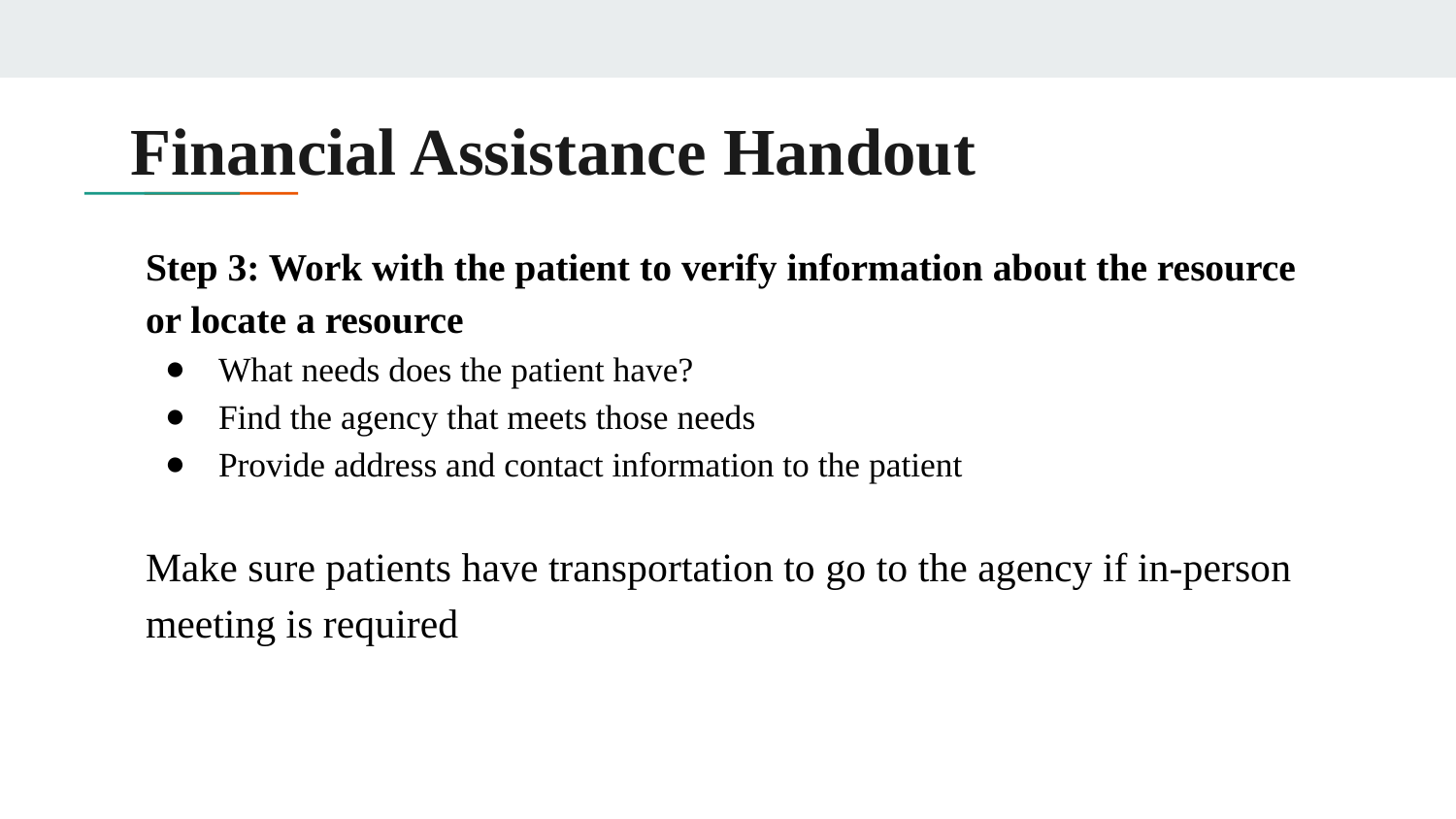

# Financial Assistance Handout
Step 3: Work with the patient to verify information about the resource or locate a resource
What needs does the patient have?
Find the agency that meets those needs
Provide address and contact information to the patient
Make sure patients have transportation to go to the agency if in-person meeting is required

## Slide 59
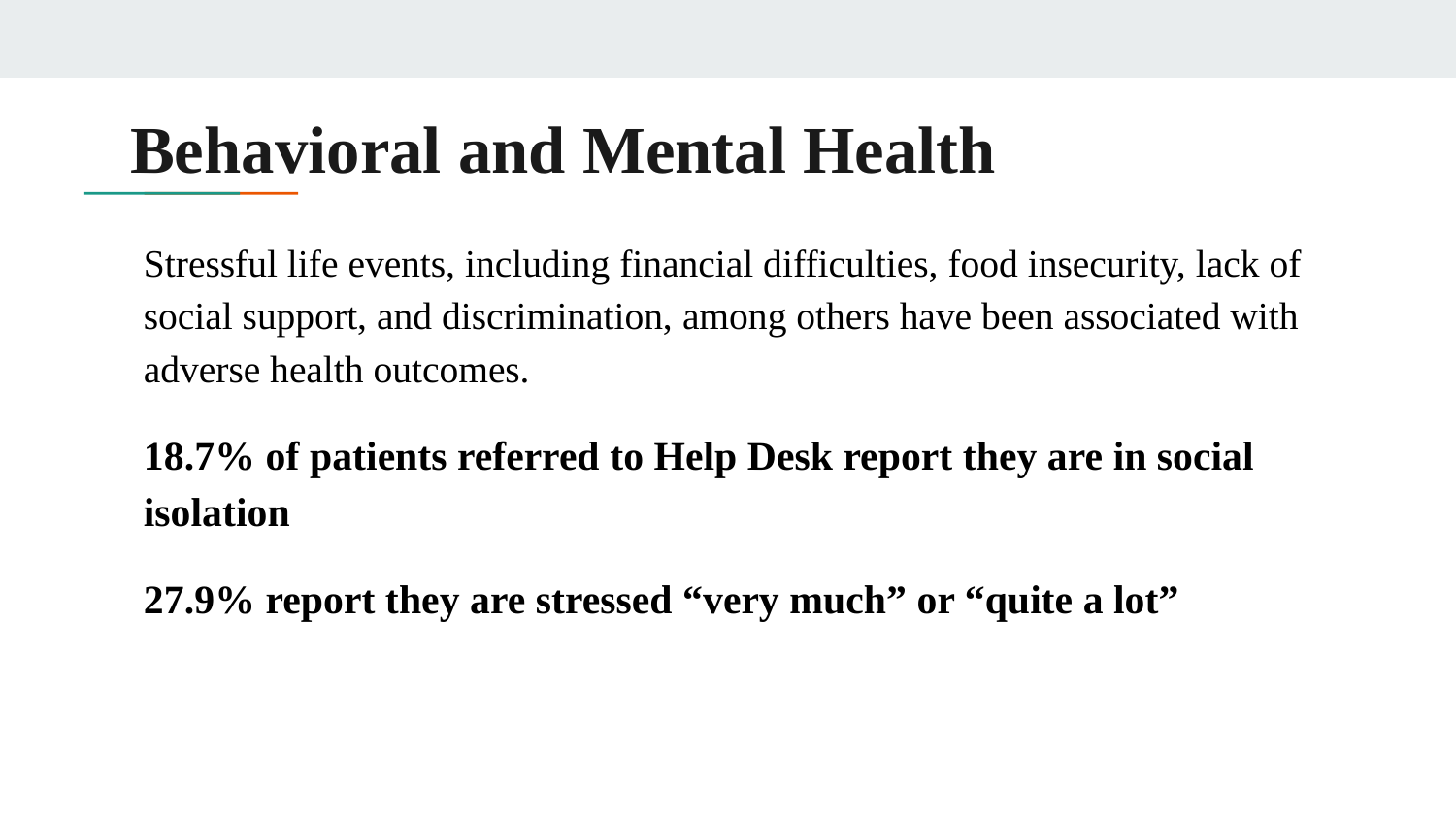

# Behavioral and Mental Health
Stressful life events, including financial difficulties, food insecurity, lack of social support, and discrimination, among others have been associated with adverse health outcomes.
18.7% of patients referred to Help Desk report they are in social isolation
27.9% report they are stressed “very much” or “quite a lot”

## Slide 60
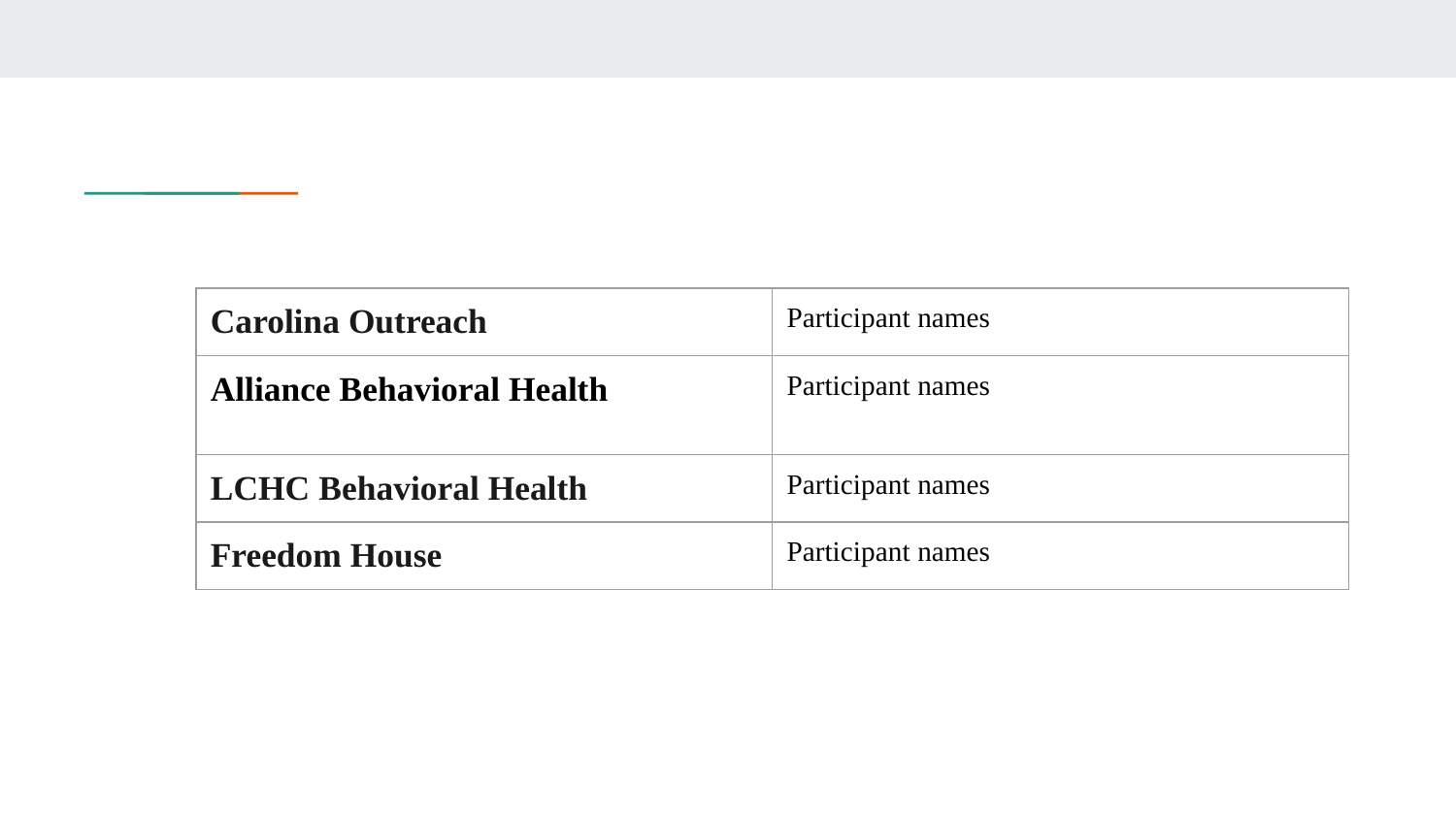

| Carolina Outreach | Participant names |
| --- | --- |
| Alliance Behavioral Health | Participant names |
| LCHC Behavioral Health | Participant names |
| Freedom House | Participant names |

## Slide 61
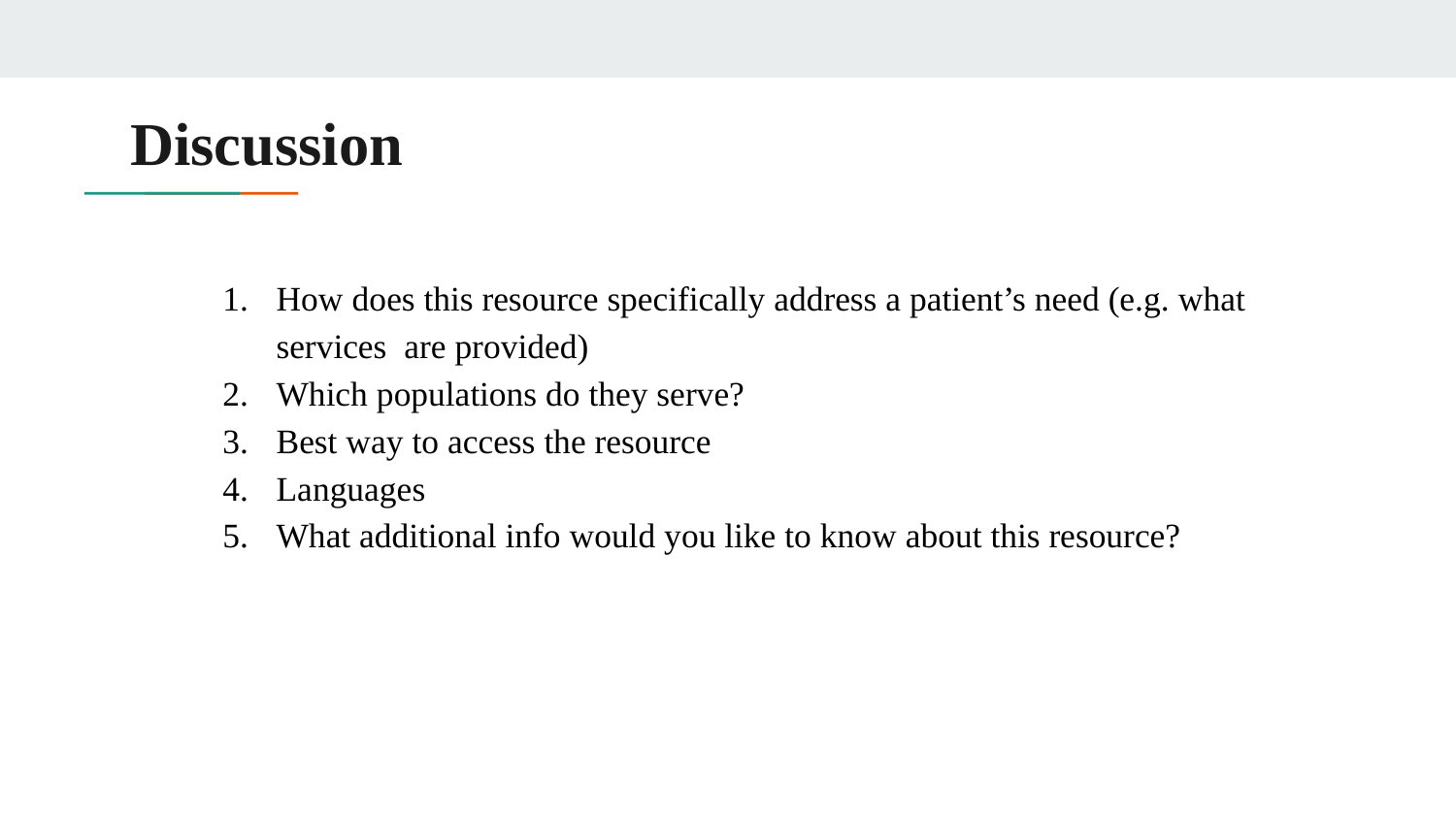

# Discussion
How does this resource specifically address a patient’s need (e.g. what services are provided)
Which populations do they serve?
Best way to access the resource
Languages
What additional info would you like to know about this resource?

## Slide 62
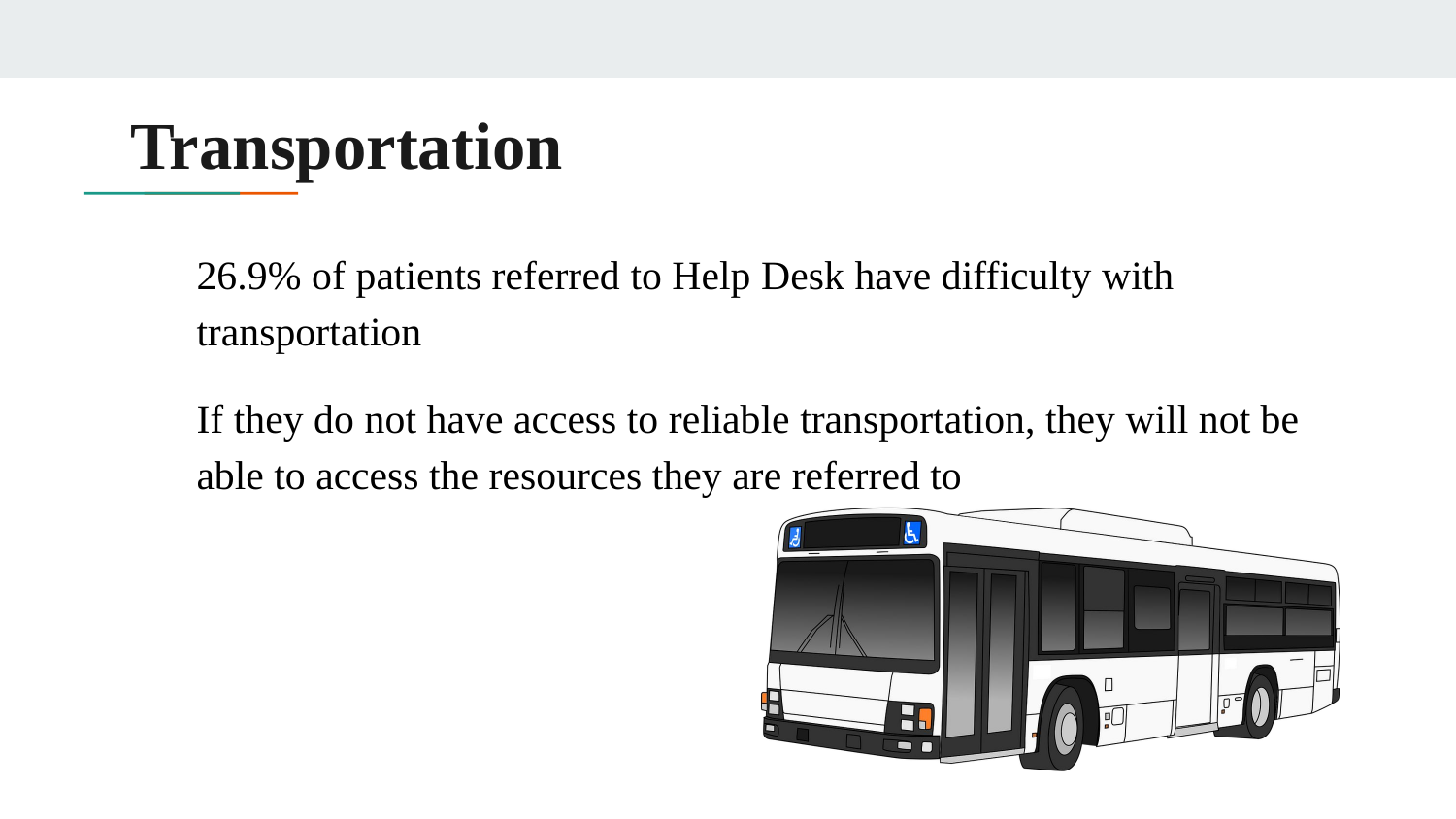

# Transportation
26.9% of patients referred to Help Desk have difficulty with transportation
If they do not have access to reliable transportation, they will not be able to access the resources they are referred to

## Slide 63
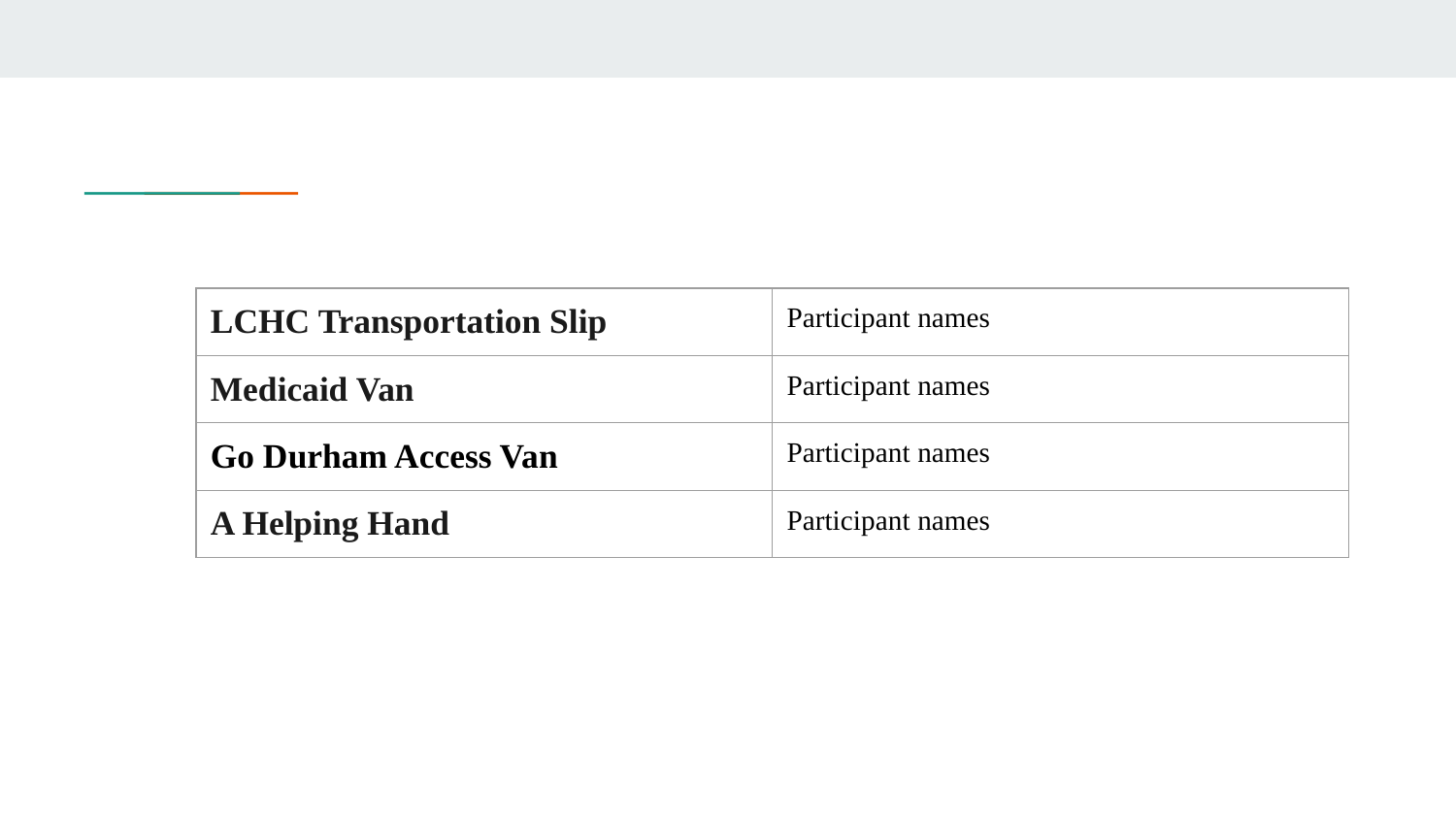

| LCHC Transportation Slip | Participant names |
| --- | --- |
| Medicaid Van | Participant names |
| Go Durham Access Van | Participant names |
| A Helping Hand | Participant names |

## Slide 64
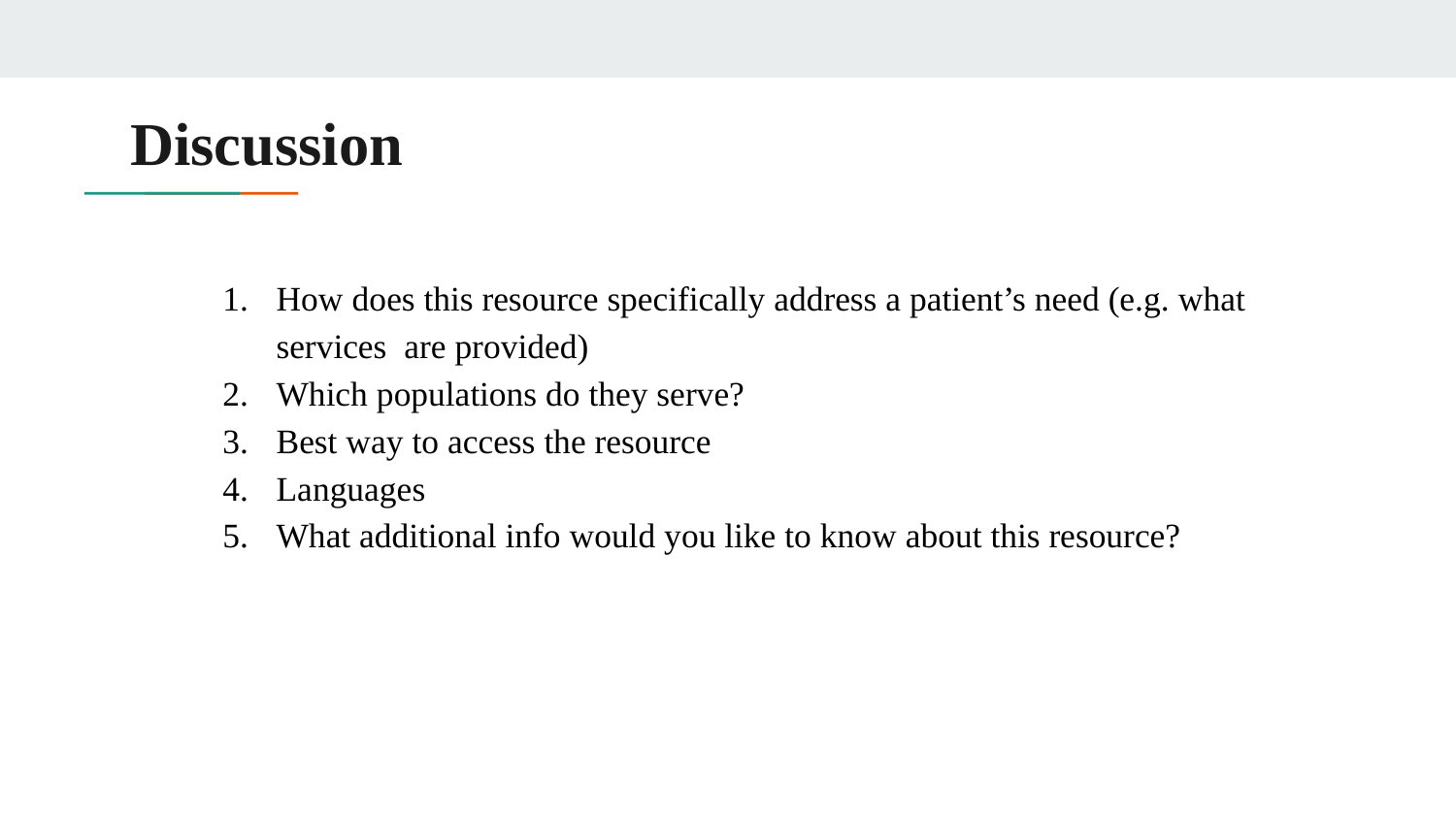

# Discussion
How does this resource specifically address a patient’s need (e.g. what services are provided)
Which populations do they serve?
Best way to access the resource
Languages
What additional info would you like to know about this resource?

## Slide 65
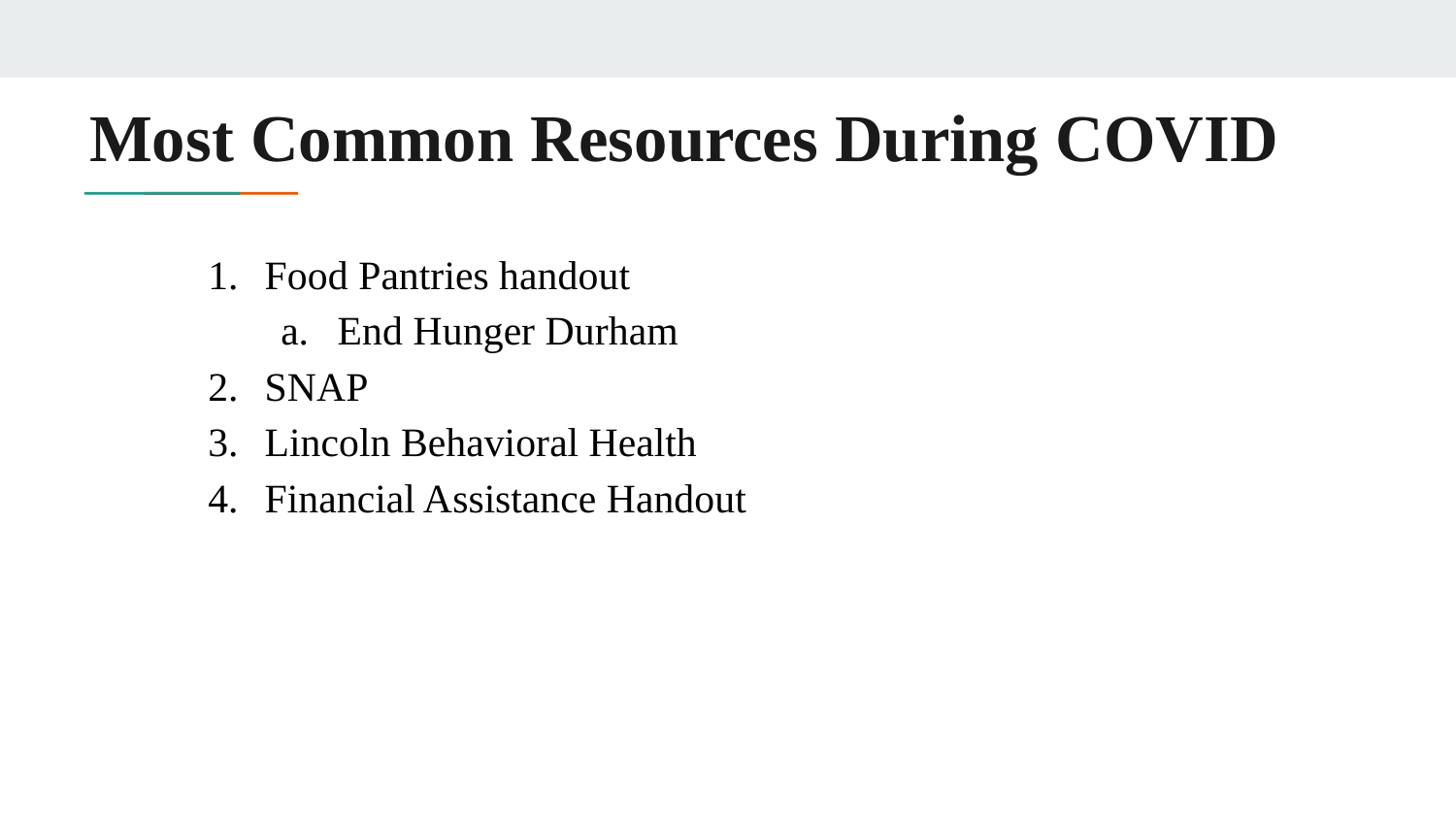

# Most Common Resources During COVID
Food Pantries handout
End Hunger Durham
SNAP
Lincoln Behavioral Health
Financial Assistance Handout

## Slide 66
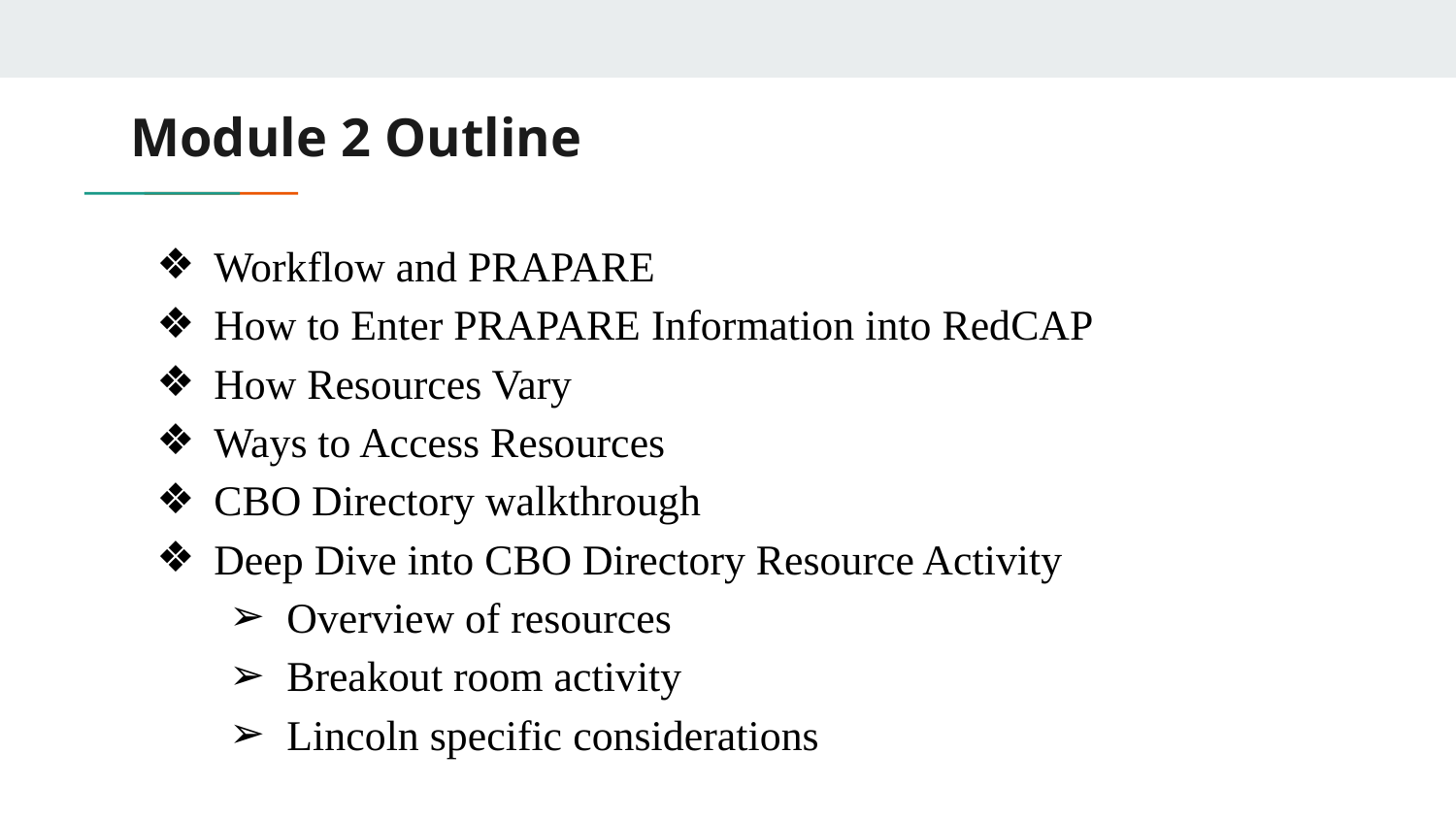

# Module 2 Outline
Workflow and PRAPARE
How to Enter PRAPARE Information into RedCAP
How Resources Vary
Ways to Access Resources
CBO Directory walkthrough
Deep Dive into CBO Directory Resource Activity
Overview of resources
Breakout room activity
Lincoln specific considerations
