# Supplementary material for: Training student volunteers as community resource navigators to address patients' social needs: A curriculum toolkit
Source: Front Public Health. 2022 Sep 20;10:966872. doi: 10.3389/fpubh.2022.966872 (PMC9531674; doi:10.3389/fpubh.2022.966872)
Supplement: Supplementary file 1 [file Data_Sheet_1.zip › Data Sheet 9.PPTX]

## Slide 1
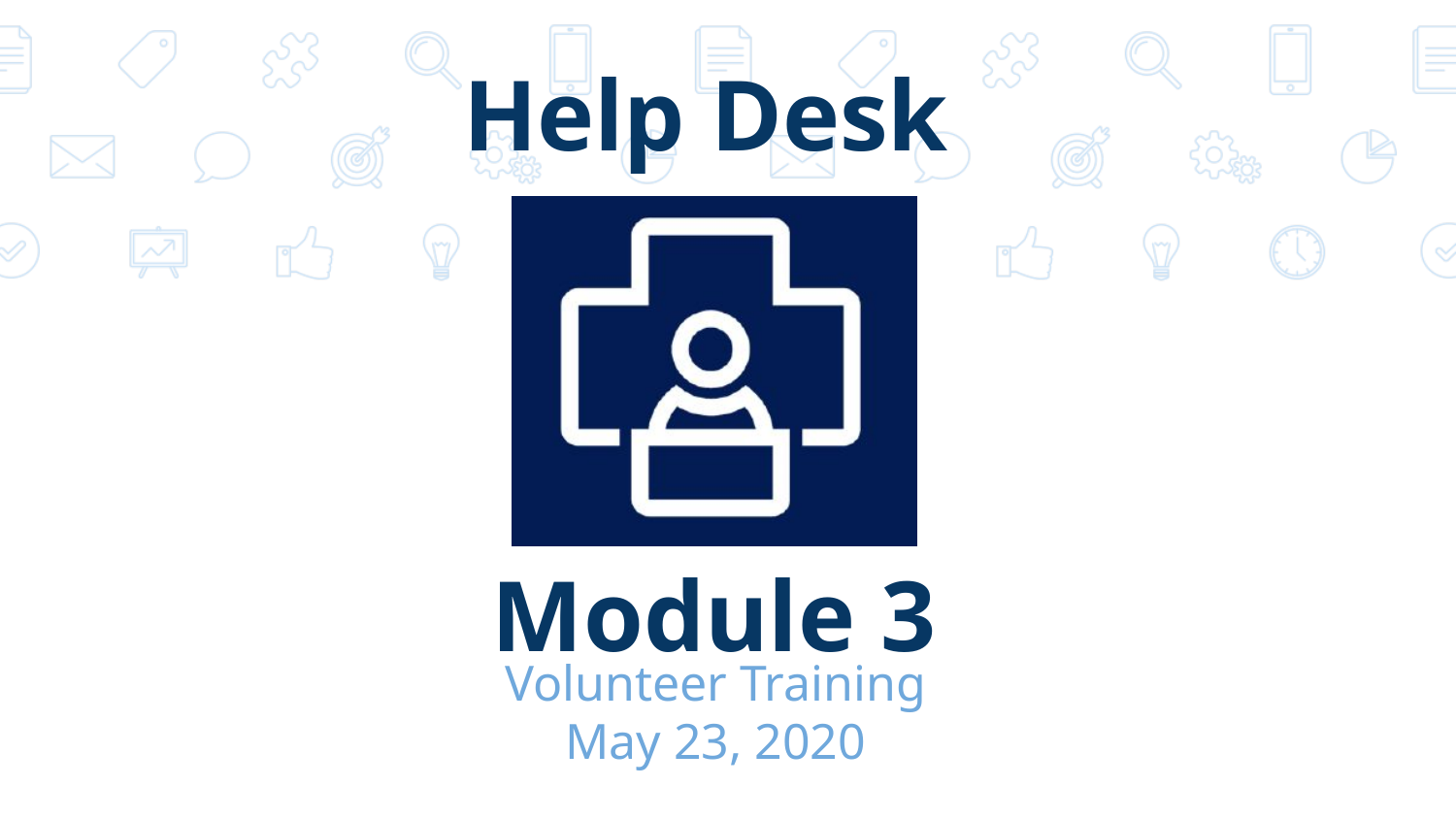

# Help Desk
Module 3
Volunteer Training
May 23, 2020

## Slide 2
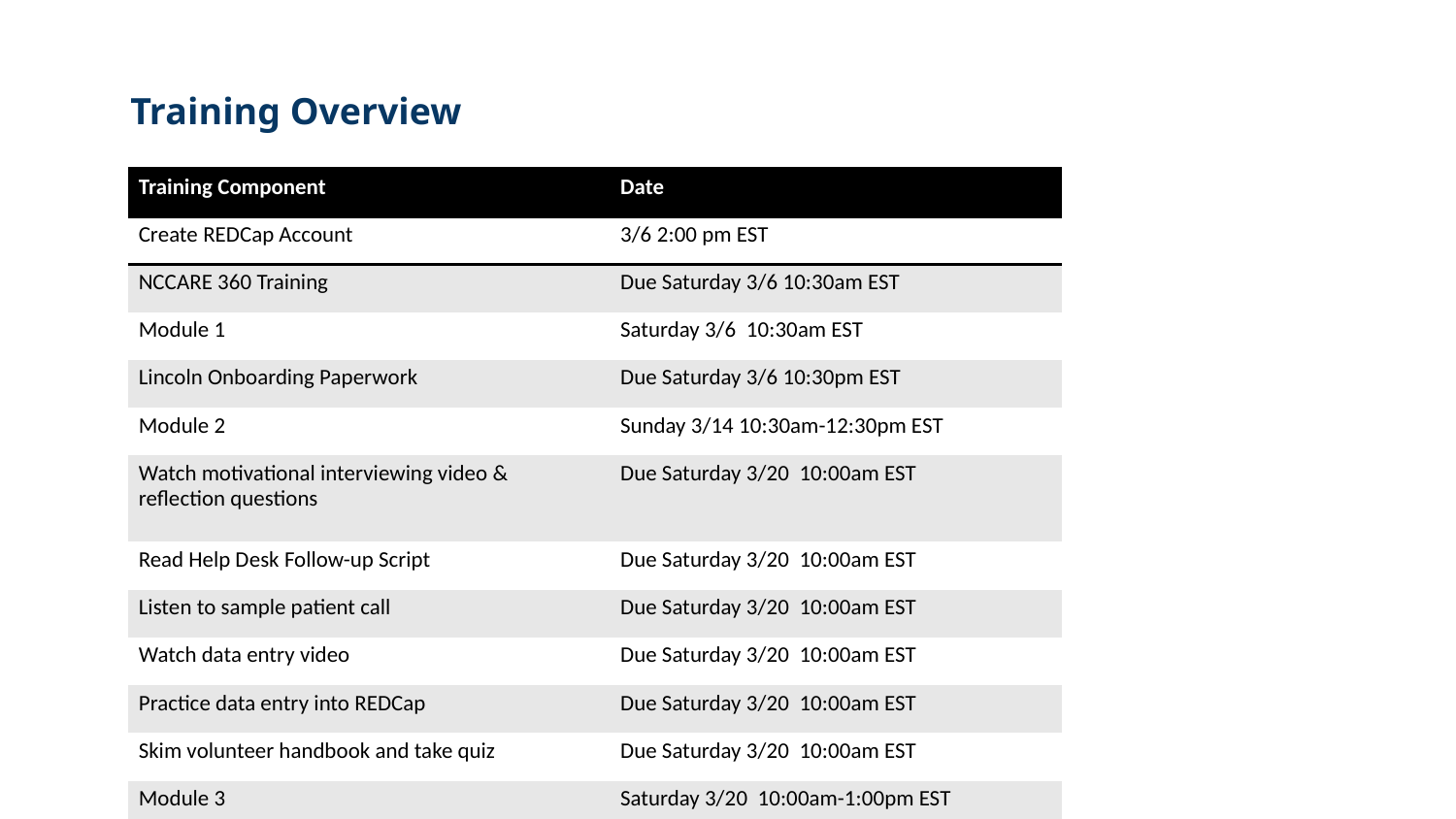

Training Overview
| Training Component | Date |
| --- | --- |
| Create REDCap Account | 3/6 2:00 pm EST |
| NCCARE 360 Training | Due Saturday 3/6 10:30am EST |
| Module 1 | Saturday 3/6 10:30am EST |
| Lincoln Onboarding Paperwork | Due Saturday 3/6 10:30pm EST |
| Module 2 | Sunday 3/14 10:30am-12:30pm EST |
| Watch motivational interviewing video & reflection questions | Due Saturday 3/20 10:00am EST |
| Read Help Desk Follow-up Script | Due Saturday 3/20 10:00am EST |
| Listen to sample patient call | Due Saturday 3/20 10:00am EST |
| Watch data entry video | Due Saturday 3/20 10:00am EST |
| Practice data entry into REDCap | Due Saturday 3/20 10:00am EST |
| Skim volunteer handbook and take quiz | Due Saturday 3/20 10:00am EST |
| Module 3 | Saturday 3/20 10:00am-1:00pm EST |

## Slide 3
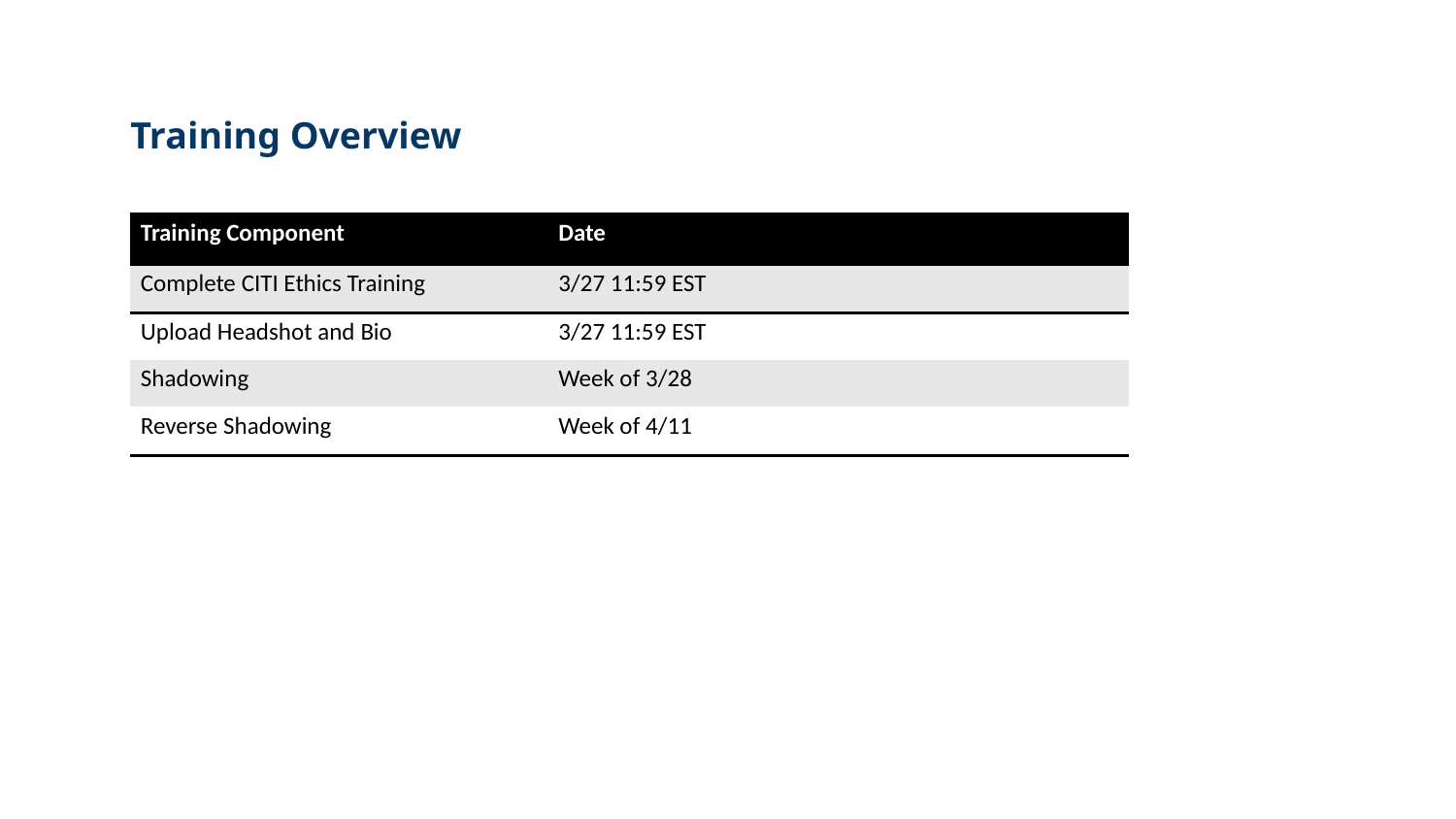

Training Overview
| Training Component | Date |
| --- | --- |
| Complete CITI Ethics Training | 3/27 11:59 EST |
| Upload Headshot and Bio | 3/27 11:59 EST |
| Shadowing | Week of 3/28 |
| Reverse Shadowing | Week of 4/11 |

## Slide 4
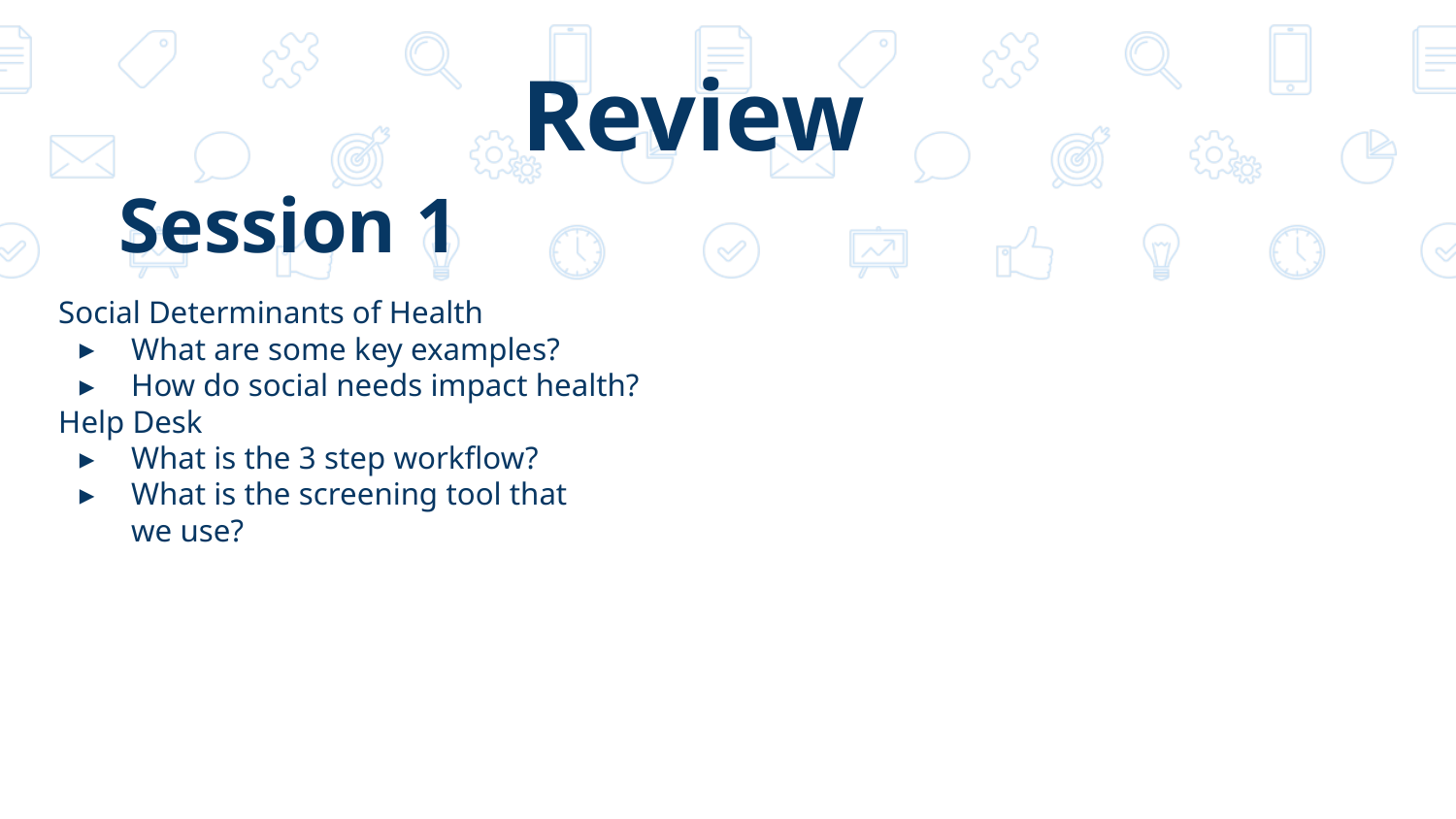

# Review
Session 1
Social Determinants of Health
What are some key examples?
How do social needs impact health?
Help Desk
What is the 3 step workflow?
What is the screening tool that
we use?

## Slide 5
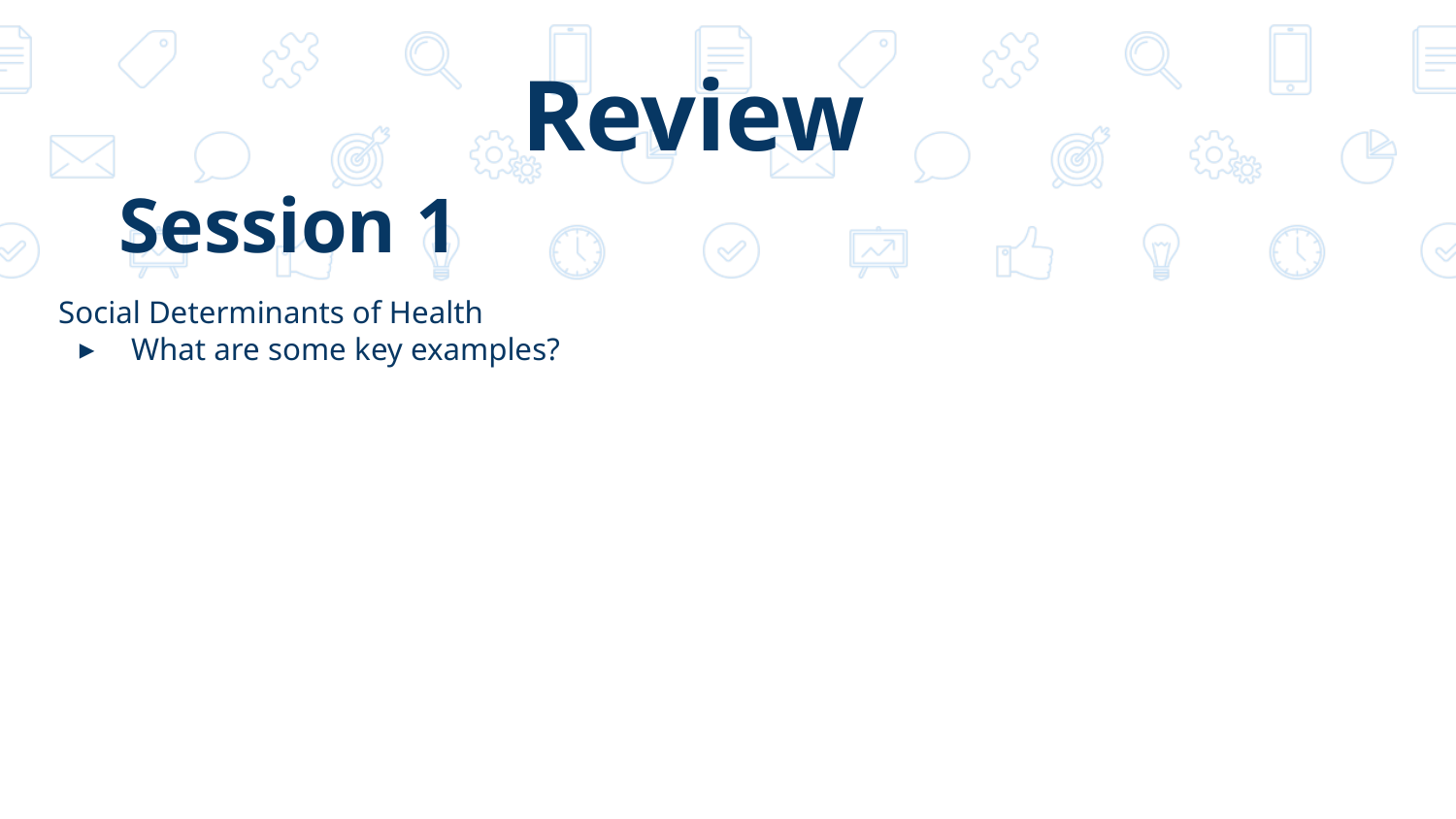

# Review
Session 1
Social Determinants of Health
What are some key examples?

## Slide 6
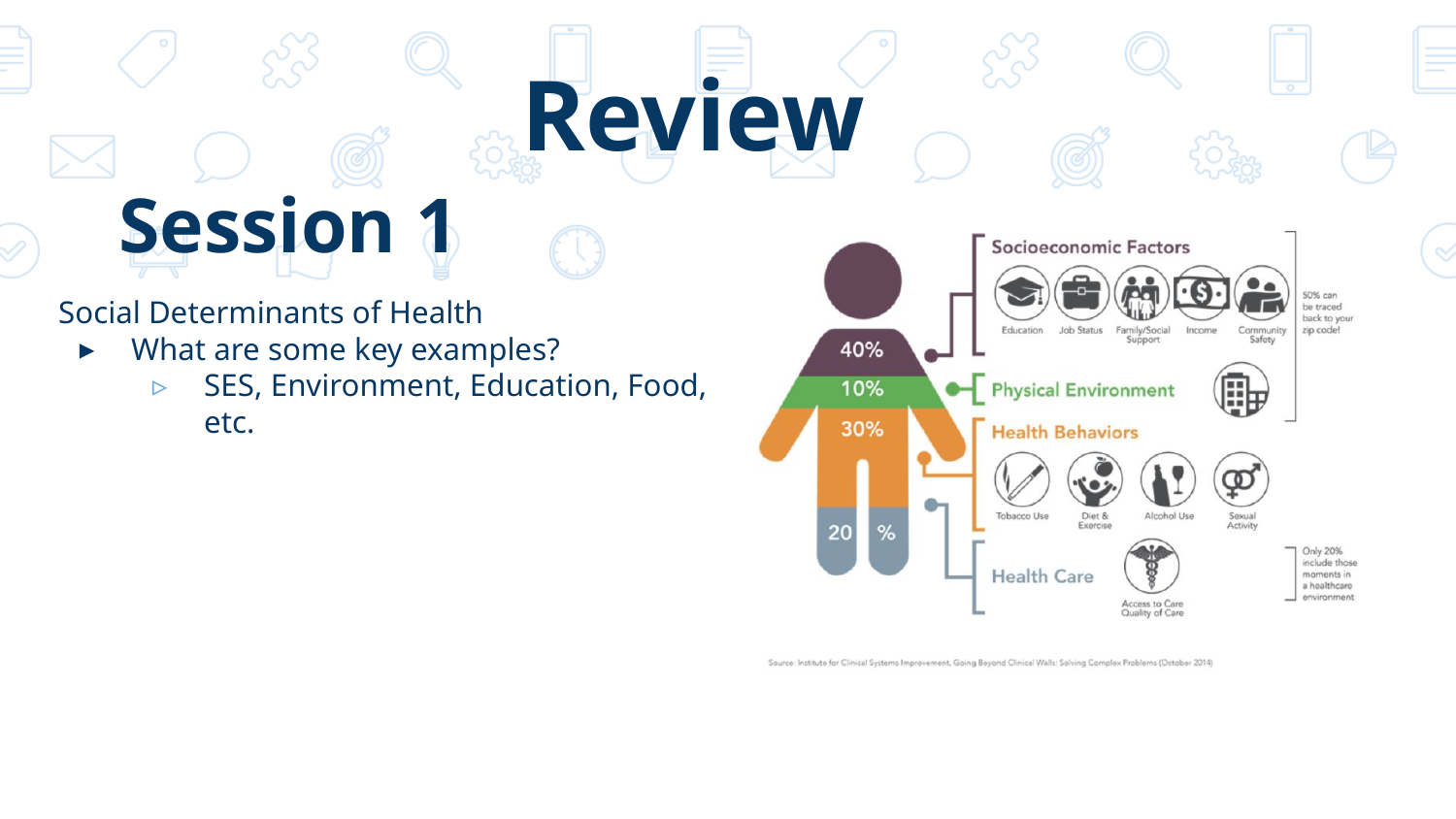

# Review
Session 1
Social Determinants of Health
What are some key examples?
SES, Environment, Education, Food, etc.

## Slide 7
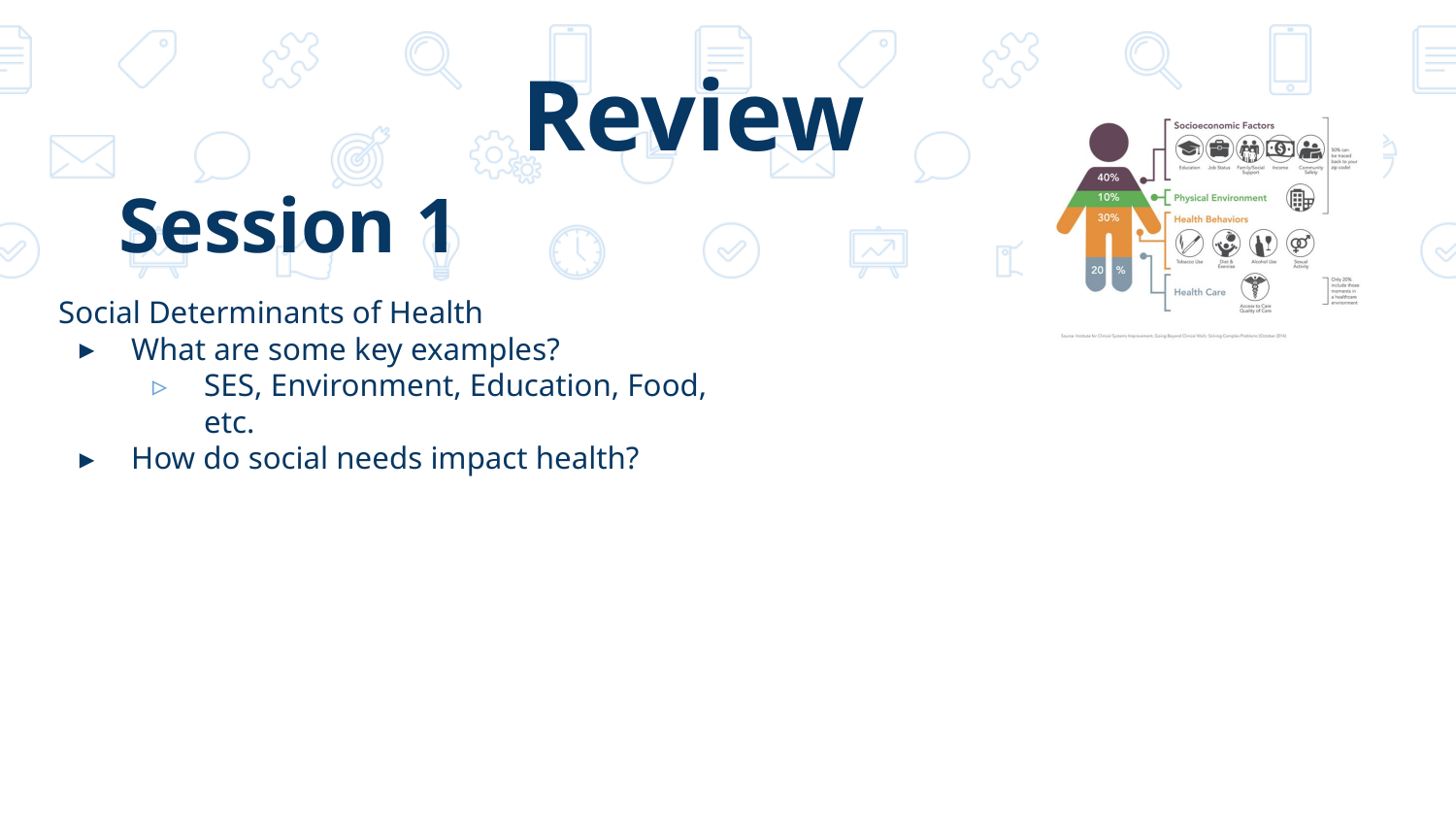

# Review
Session 1
Social Determinants of Health
What are some key examples?
SES, Environment, Education, Food, etc.
How do social needs impact health?

## Slide 8
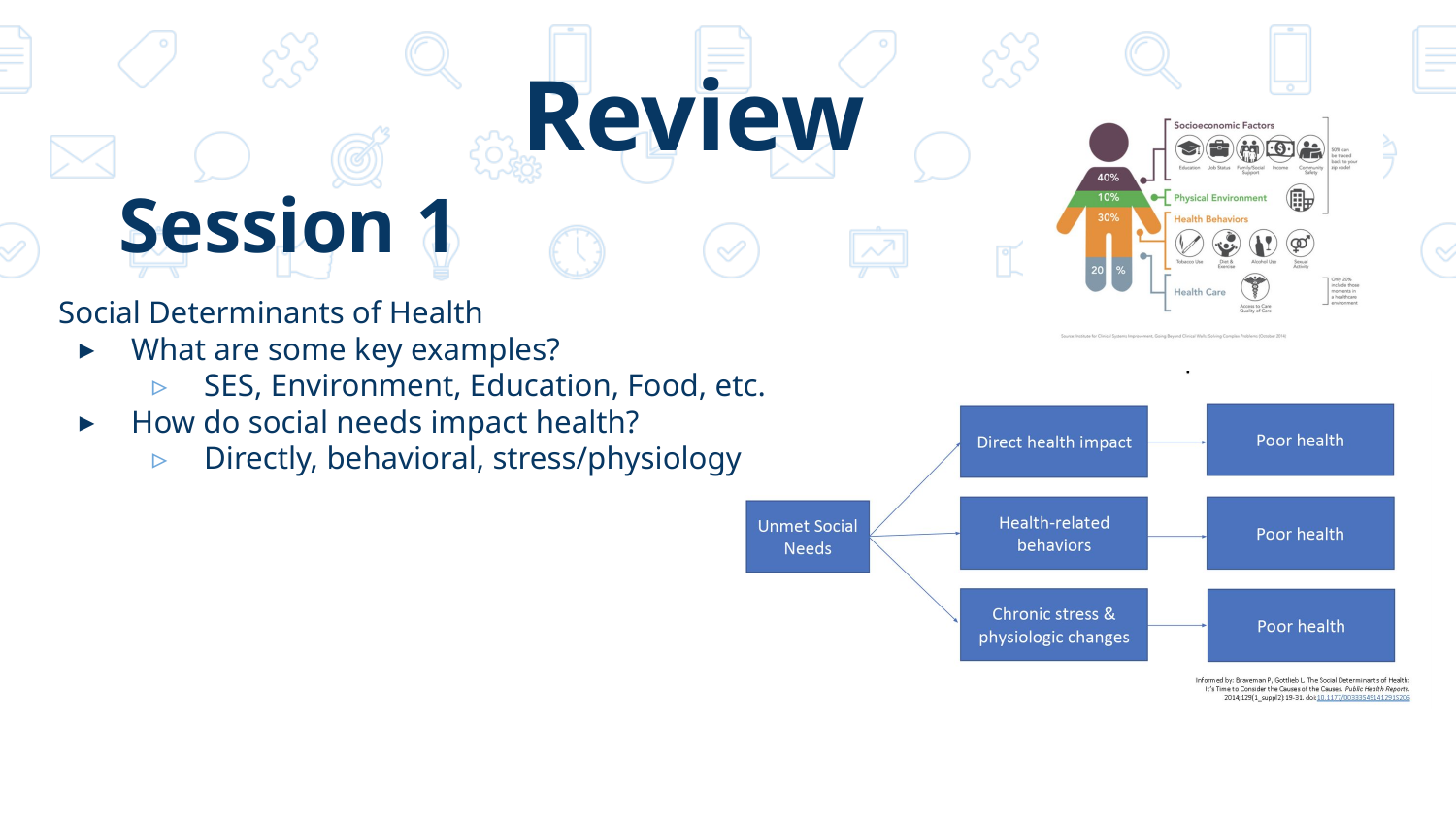

# Review
Session 1
Social Determinants of Health
What are some key examples?
SES, Environment, Education, Food, etc.
How do social needs impact health?
Directly, behavioral, stress/physiology

## Slide 9
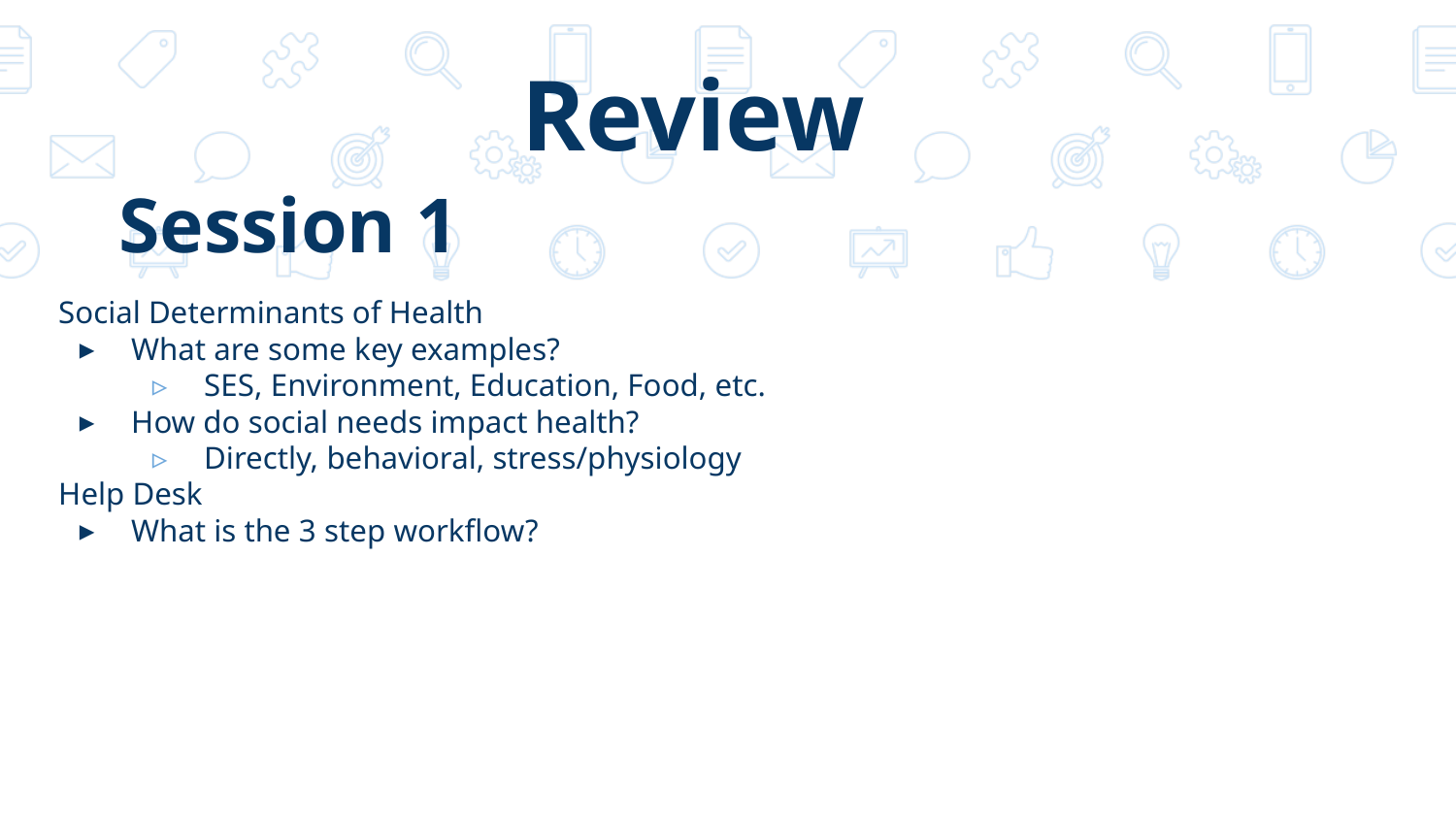

# Review
Session 1
Social Determinants of Health
What are some key examples?
SES, Environment, Education, Food, etc.
How do social needs impact health?
Directly, behavioral, stress/physiology
Help Desk
What is the 3 step workflow?

## Slide 10
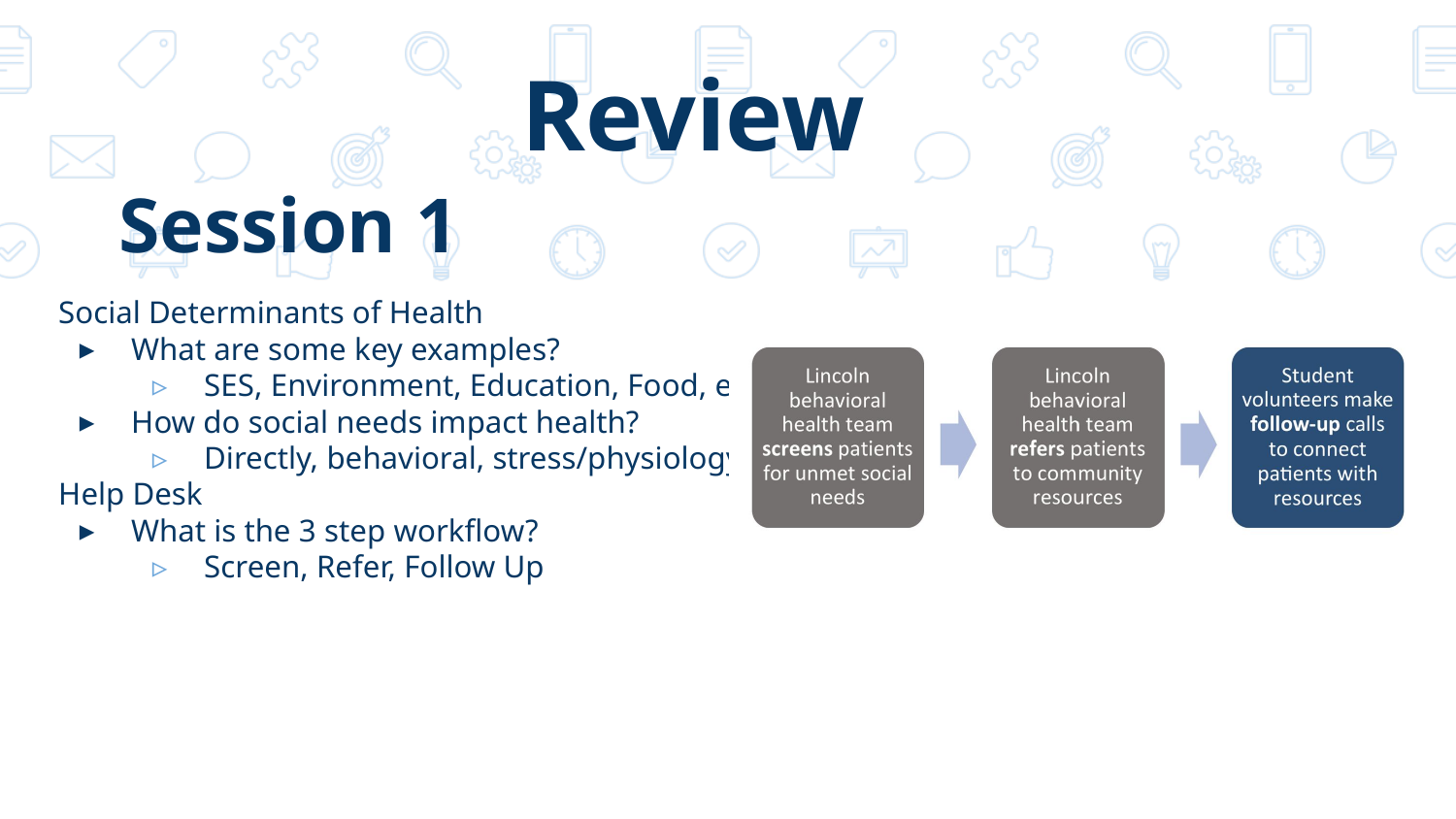

# Review
Session 1
Social Determinants of Health
What are some key examples?
SES, Environment, Education, Food, etc.
How do social needs impact health?
Directly, behavioral, stress/physiology
Help Desk
What is the 3 step workflow?
Screen, Refer, Follow Up

## Slide 11
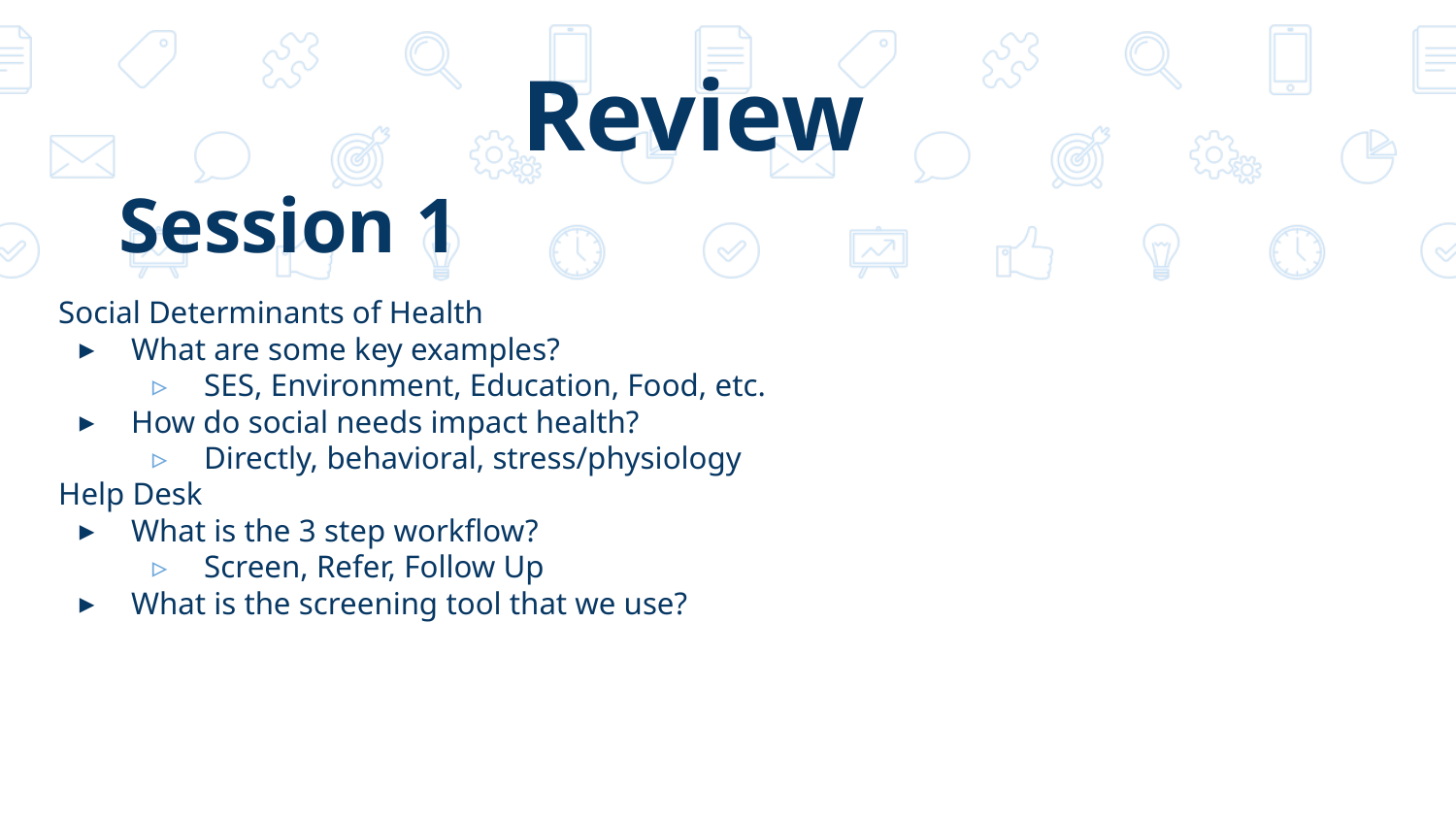

# Review
Session 1
Social Determinants of Health
What are some key examples?
SES, Environment, Education, Food, etc.
How do social needs impact health?
Directly, behavioral, stress/physiology
Help Desk
What is the 3 step workflow?
Screen, Refer, Follow Up
What is the screening tool that we use?

## Slide 12
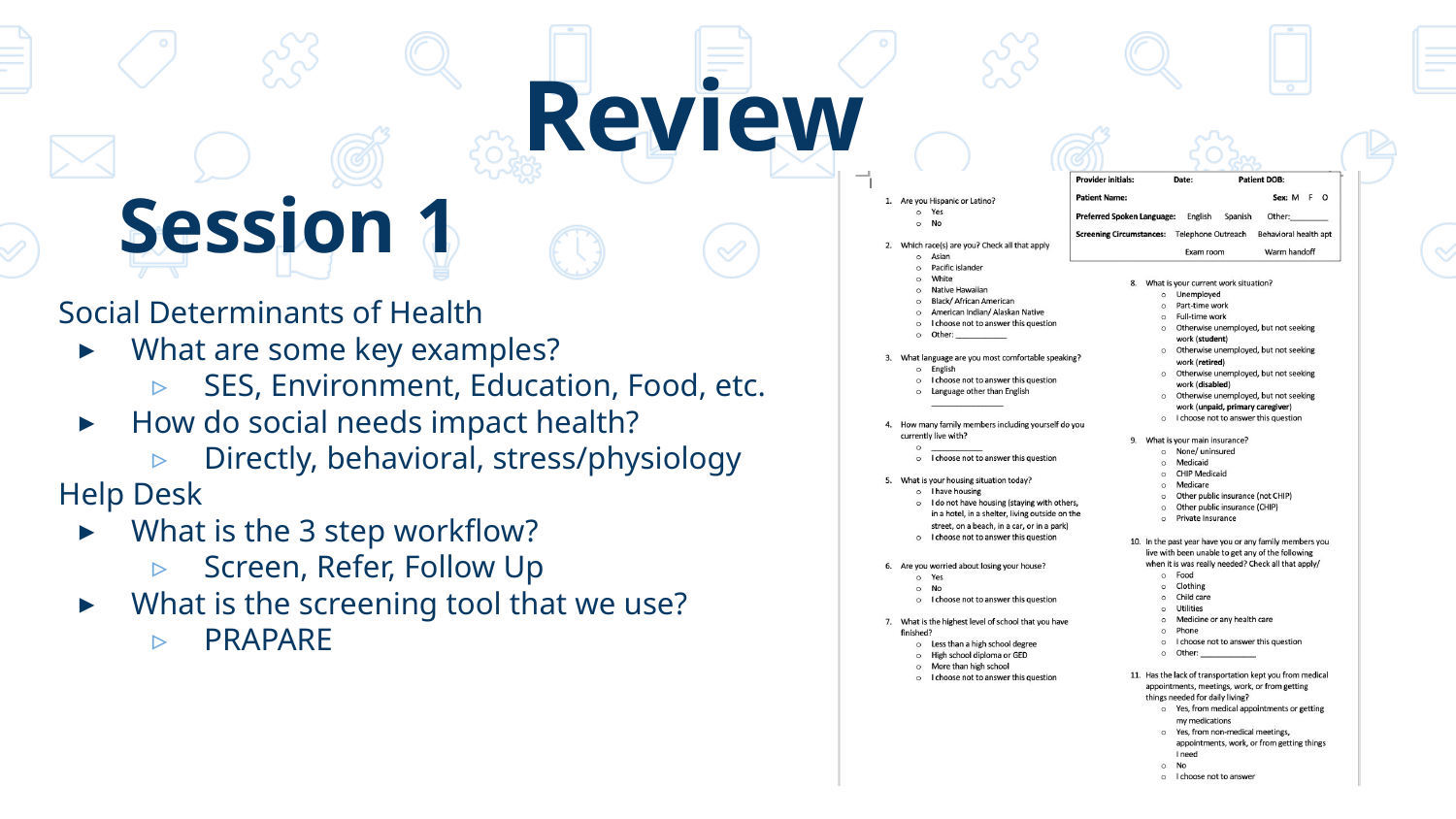

# Review
Session 1
Social Determinants of Health
What are some key examples?
SES, Environment, Education, Food, etc.
How do social needs impact health?
Directly, behavioral, stress/physiology
Help Desk
What is the 3 step workflow?
Screen, Refer, Follow Up
What is the screening tool that we use?
PRAPARE

## Slide 13
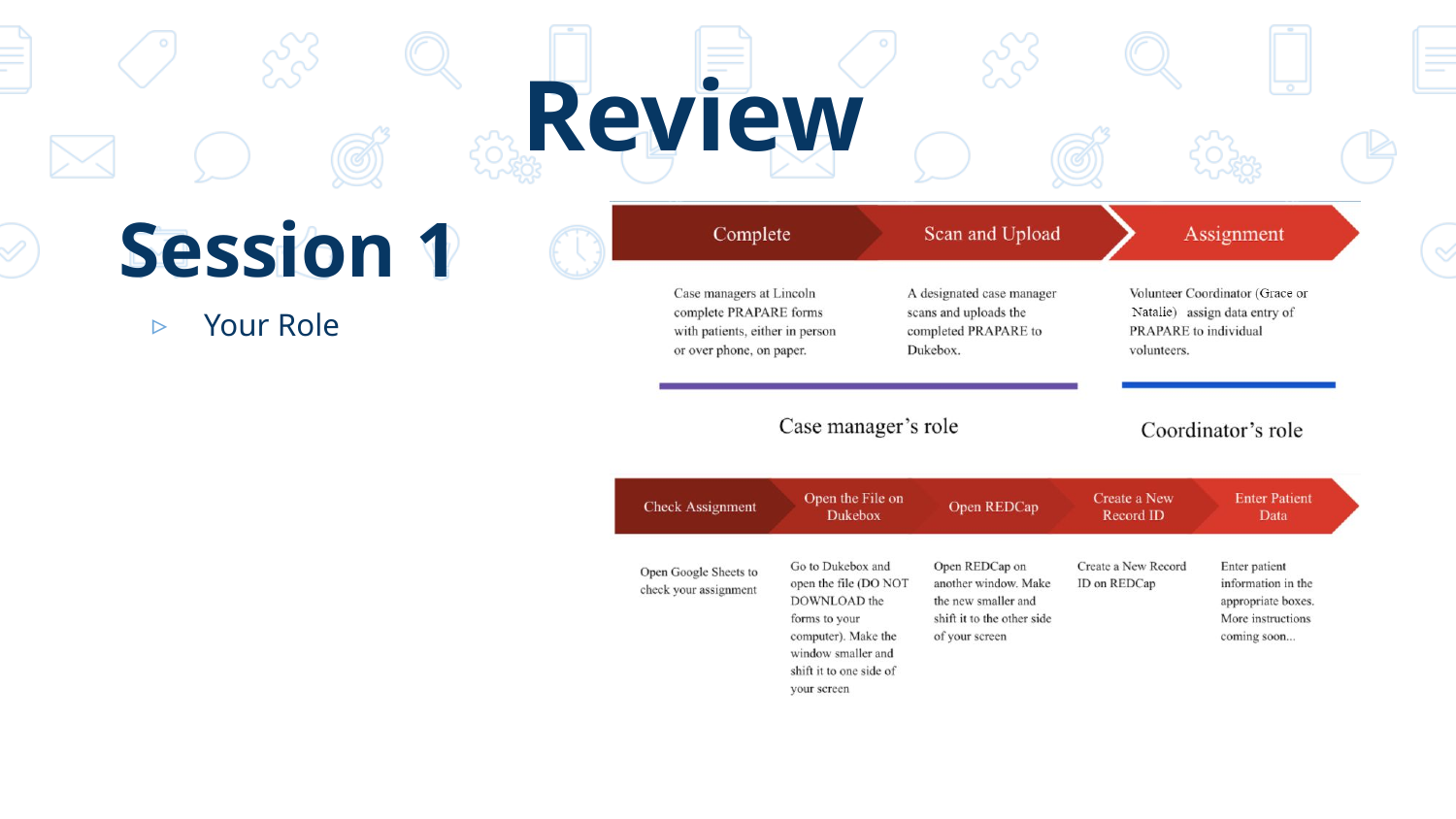

# Review
Session 1
Your Role

## Slide 14
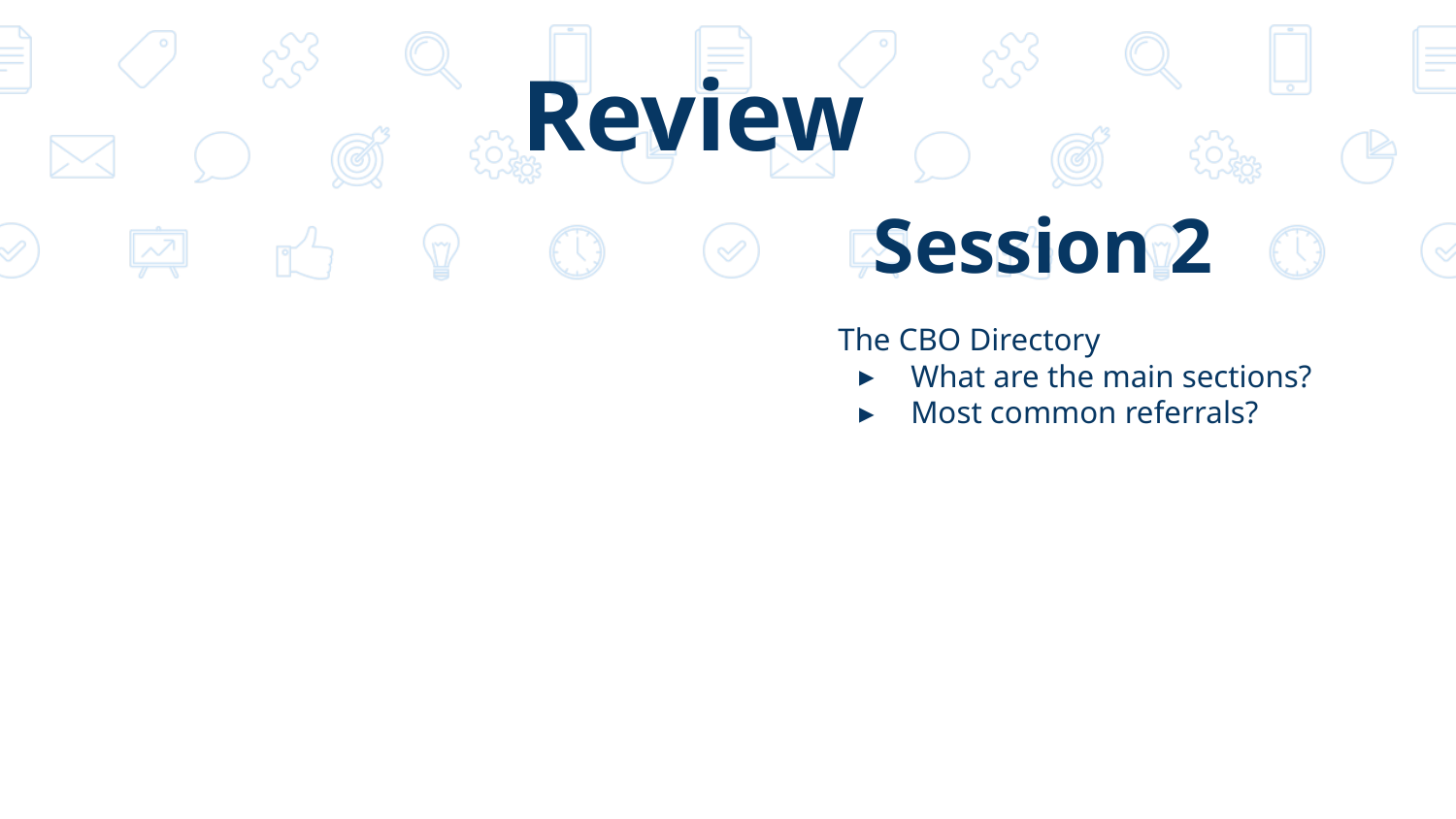

# Review
Session 2
The CBO Directory
What are the main sections?
Most common referrals?

## Slide 15
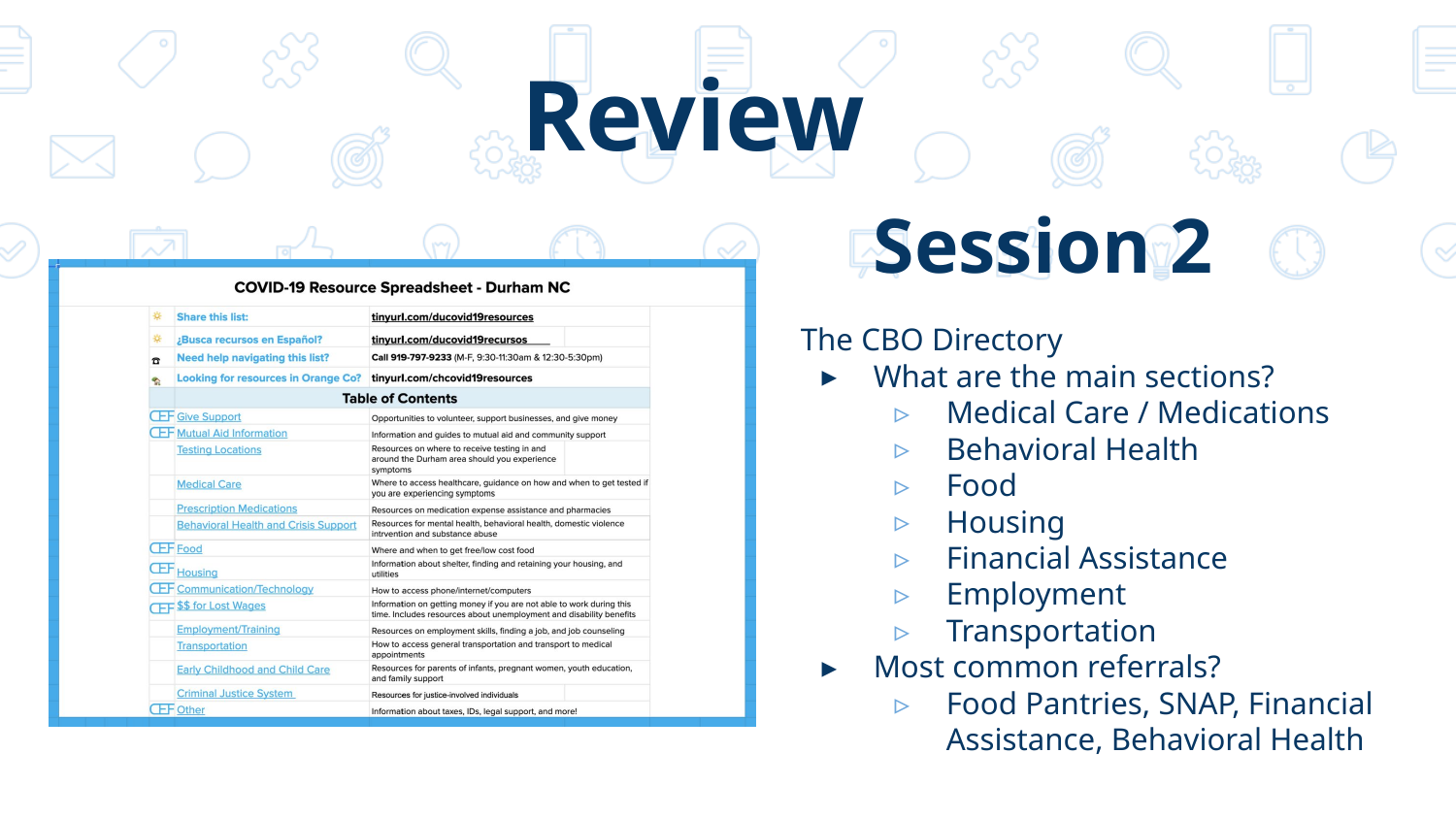

# Review
Session 2
The CBO Directory
What are the main sections?
Medical Care / Medications
Behavioral Health
Food
Housing
Financial Assistance
Employment
Transportation
Most common referrals?
Food Pantries, SNAP, Financial Assistance, Behavioral Health

## Slide 16
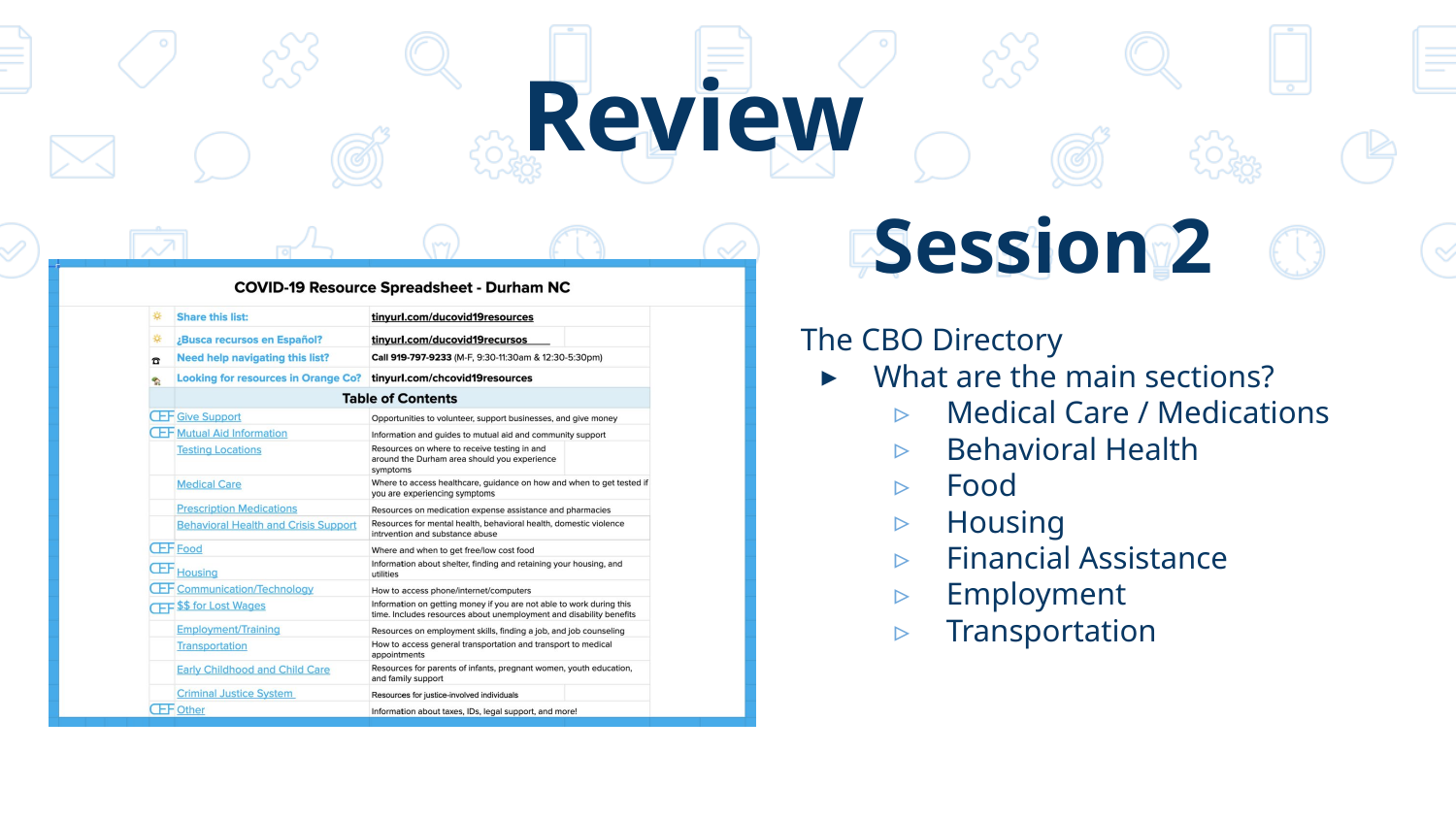

# Review
Session 2
The CBO Directory
What are the main sections?
Medical Care / Medications
Behavioral Health
Food
Housing
Financial Assistance
Employment
Transportation

## Slide 17
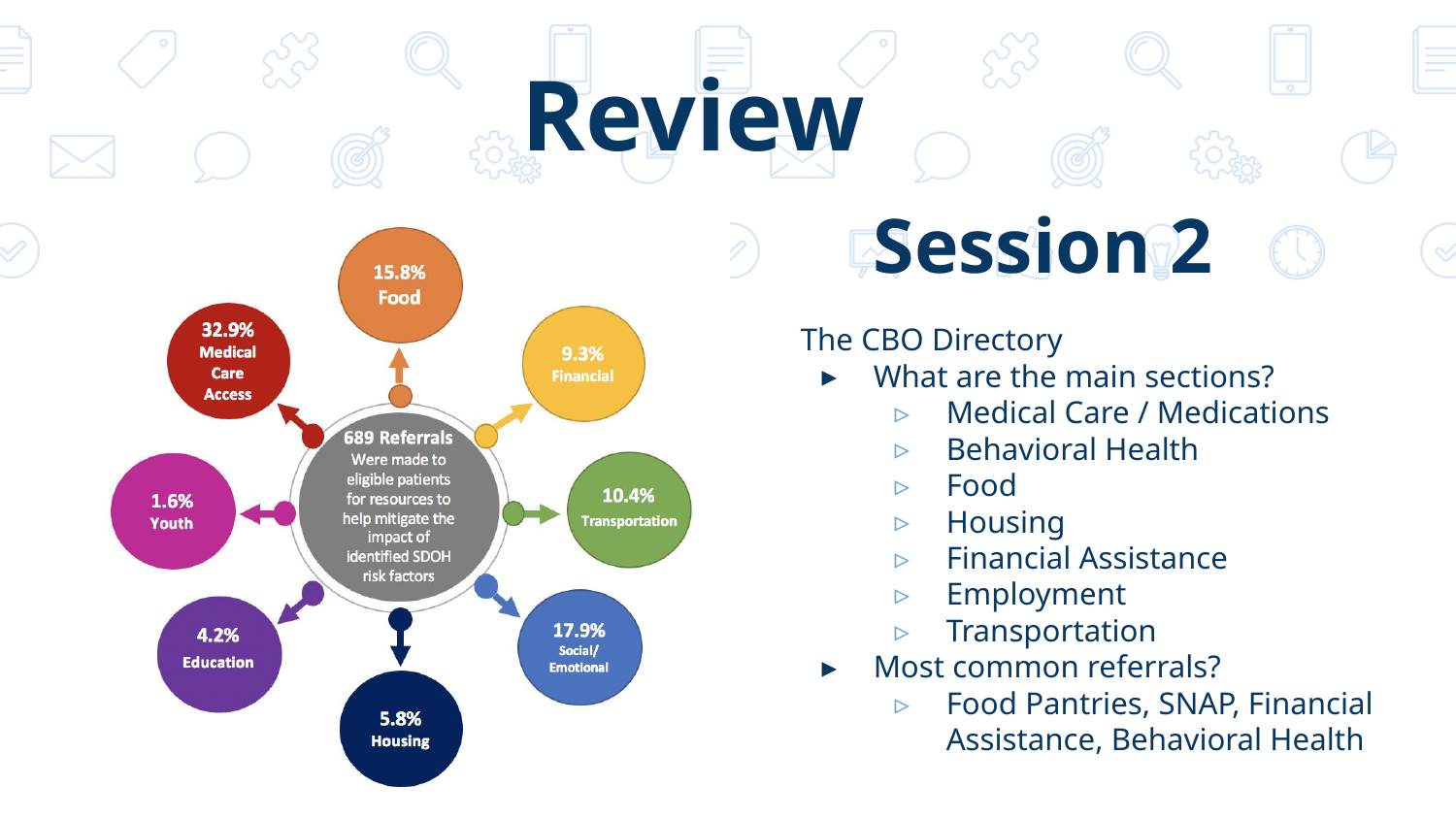

# Review
Session 2
The CBO Directory
What are the main sections?
Medical Care / Medications
Behavioral Health
Food
Housing
Financial Assistance
Employment
Transportation
Most common referrals?
Food Pantries, SNAP, Financial Assistance, Behavioral Health

## Slide 18
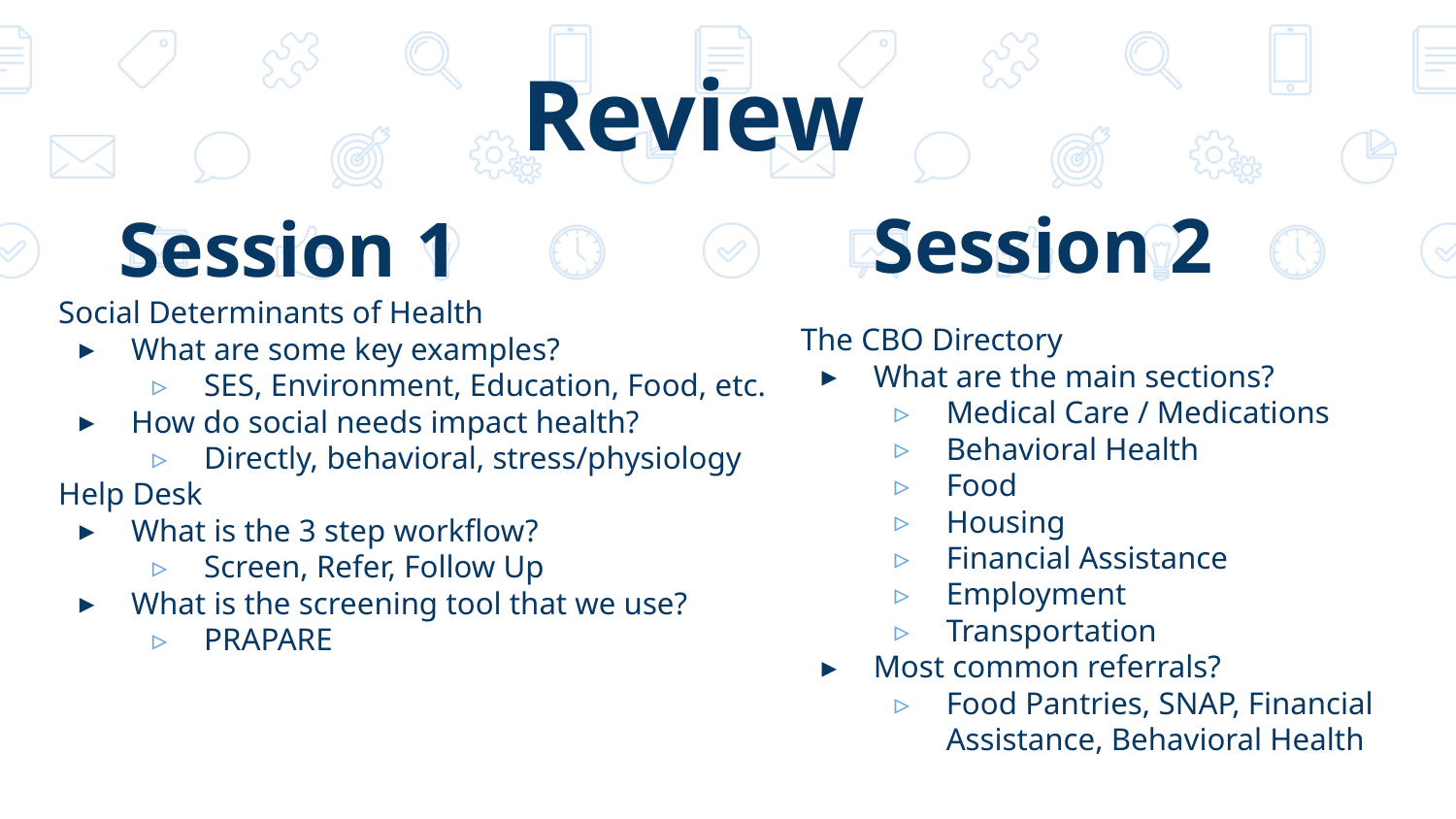

# Review
Session 2
Session 1
Social Determinants of Health
What are some key examples?
SES, Environment, Education, Food, etc.
How do social needs impact health?
Directly, behavioral, stress/physiology
Help Desk
What is the 3 step workflow?
Screen, Refer, Follow Up
What is the screening tool that we use?
PRAPARE
The CBO Directory
What are the main sections?
Medical Care / Medications
Behavioral Health
Food
Housing
Financial Assistance
Employment
Transportation
Most common referrals?
Food Pantries, SNAP, Financial Assistance, Behavioral Health

## Slide 19
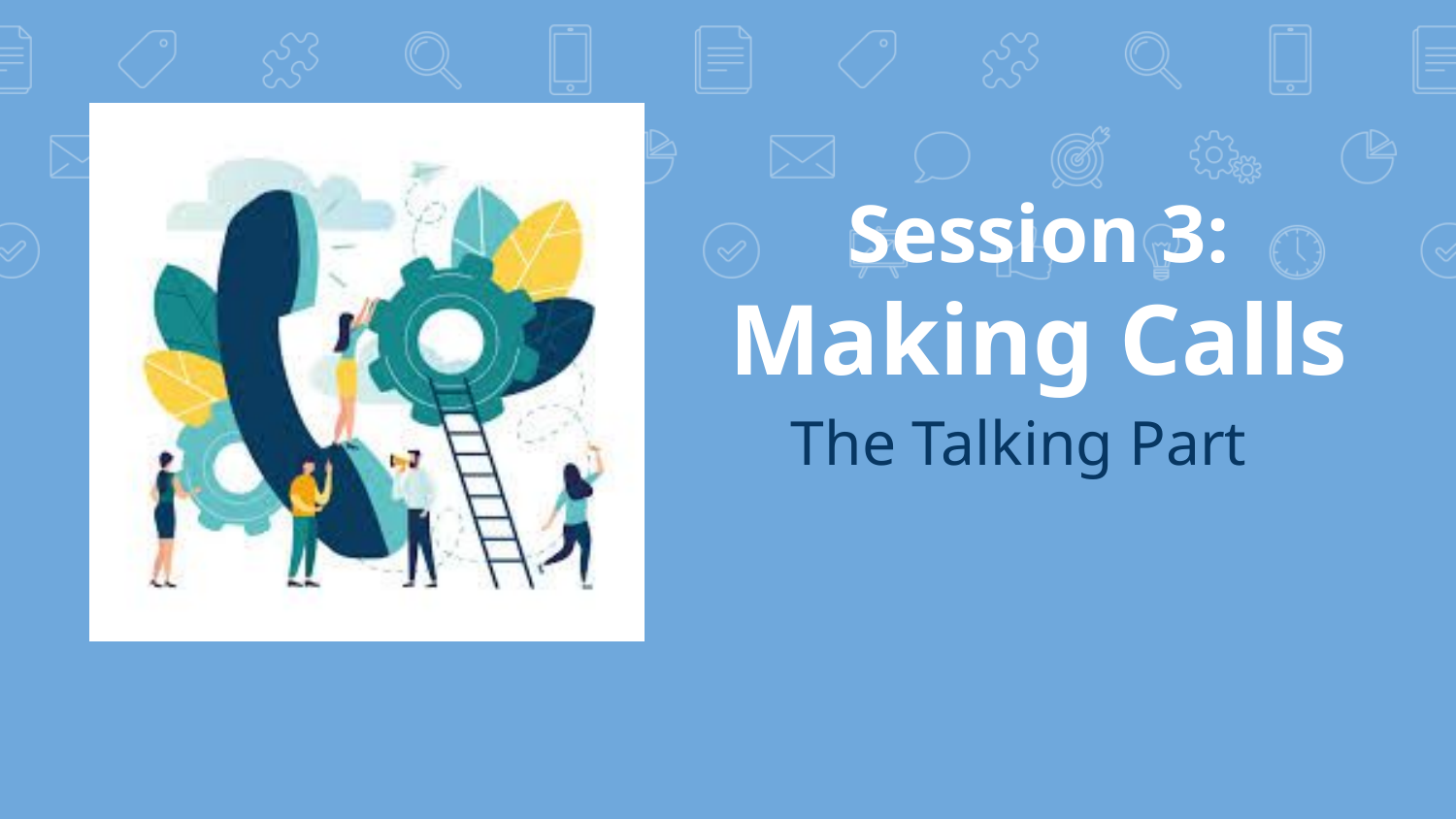

# Session 3:
Making Calls
The Talking Part

## Slide 20
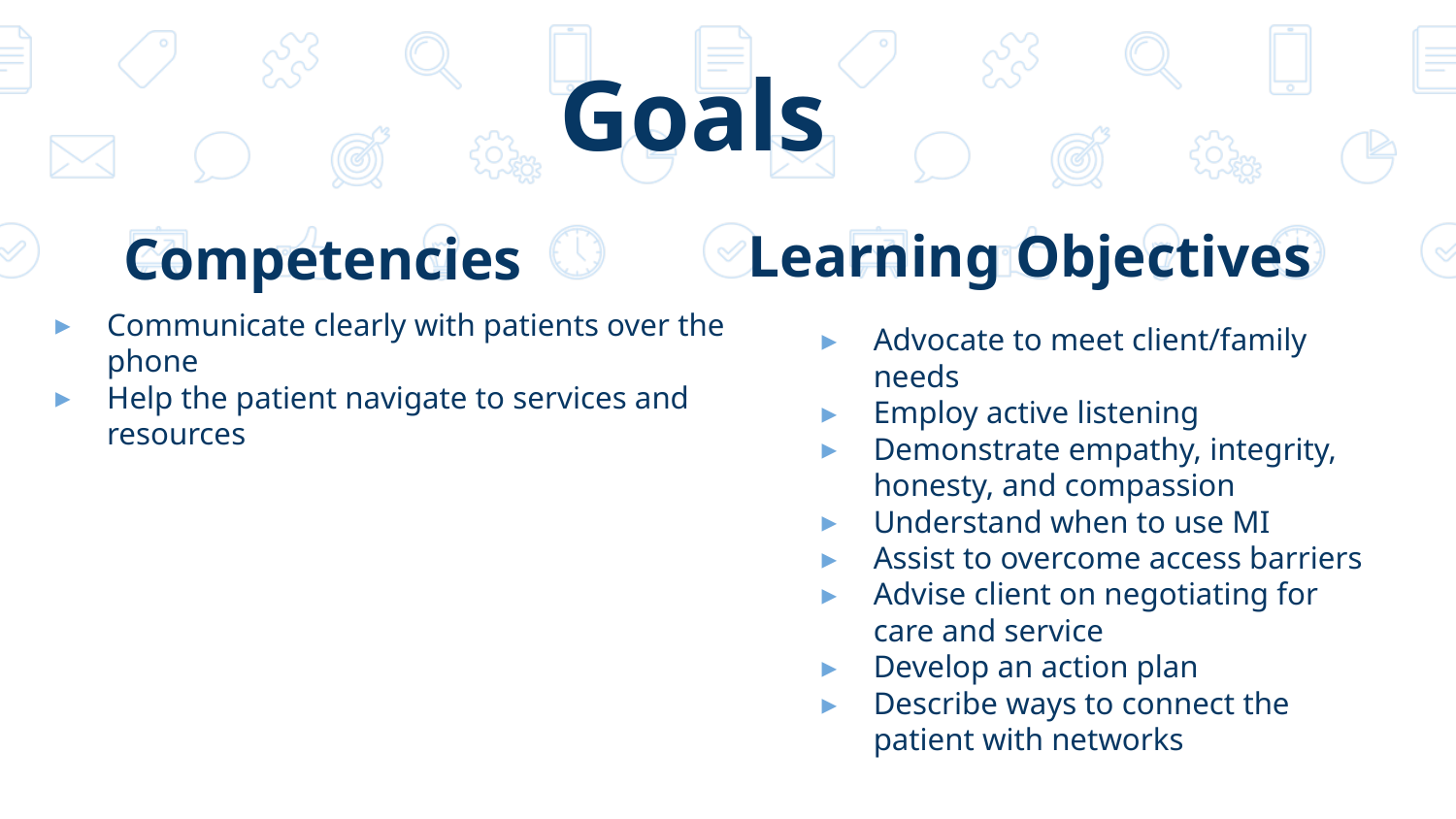

# Goals
Learning Objectives
Competencies
Communicate clearly with patients over the phone
Help the patient navigate to services and resources
Advocate to meet client/family needs
Employ active listening
Demonstrate empathy, integrity, honesty, and compassion
Understand when to use MI
Assist to overcome access barriers
Advise client on negotiating for care and service
Develop an action plan
Describe ways to connect the patient with networks

## Slide 21
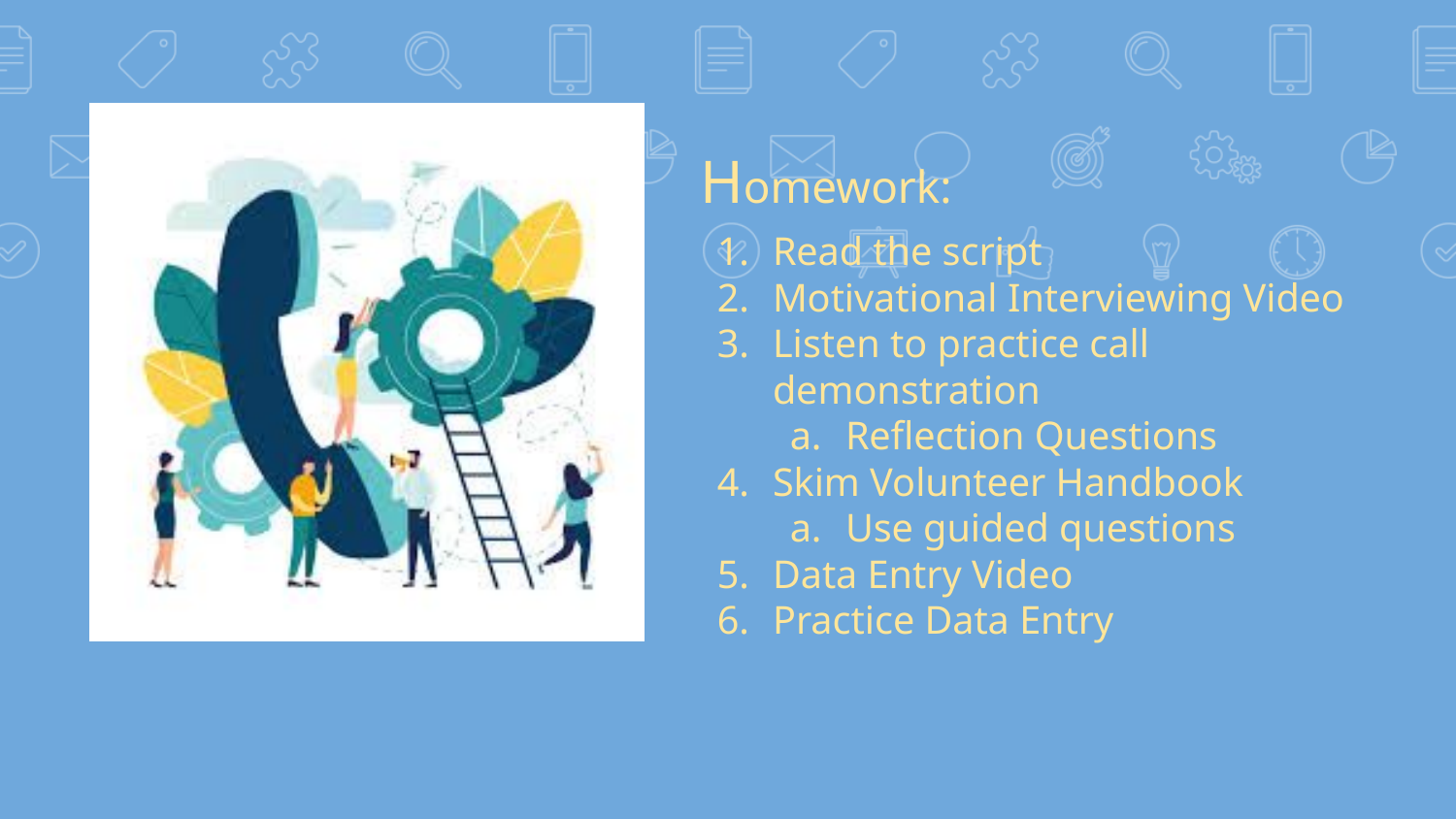

Homework:
Read the script
Motivational Interviewing Video
Listen to practice call demonstration
Reflection Questions
Skim Volunteer Handbook
Use guided questions
Data Entry Video
Practice Data Entry

## Slide 22
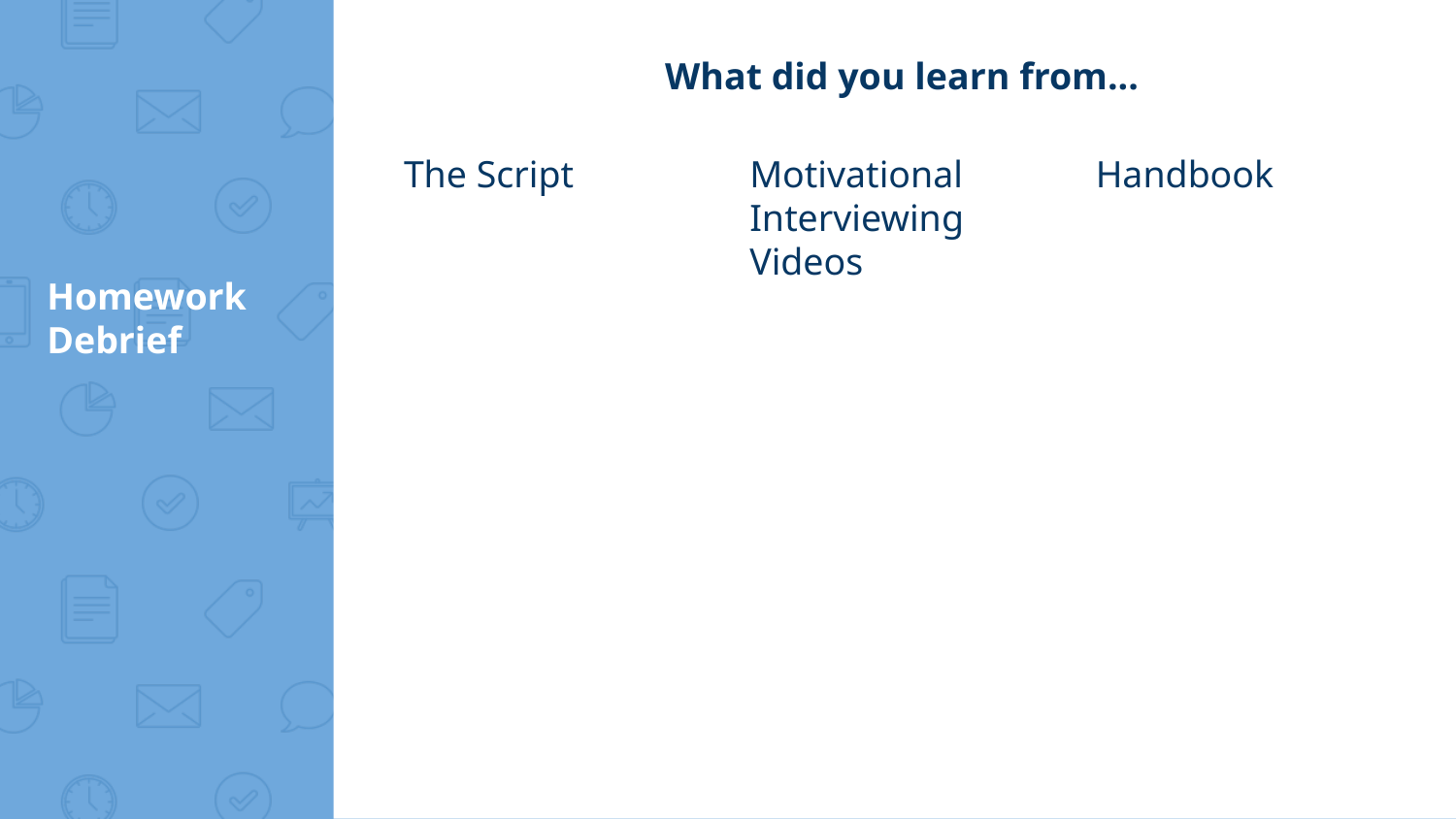

What did you learn from...
The Script
Motivational Interviewing Videos
Handbook
# Homework Debrief

## Slide 23
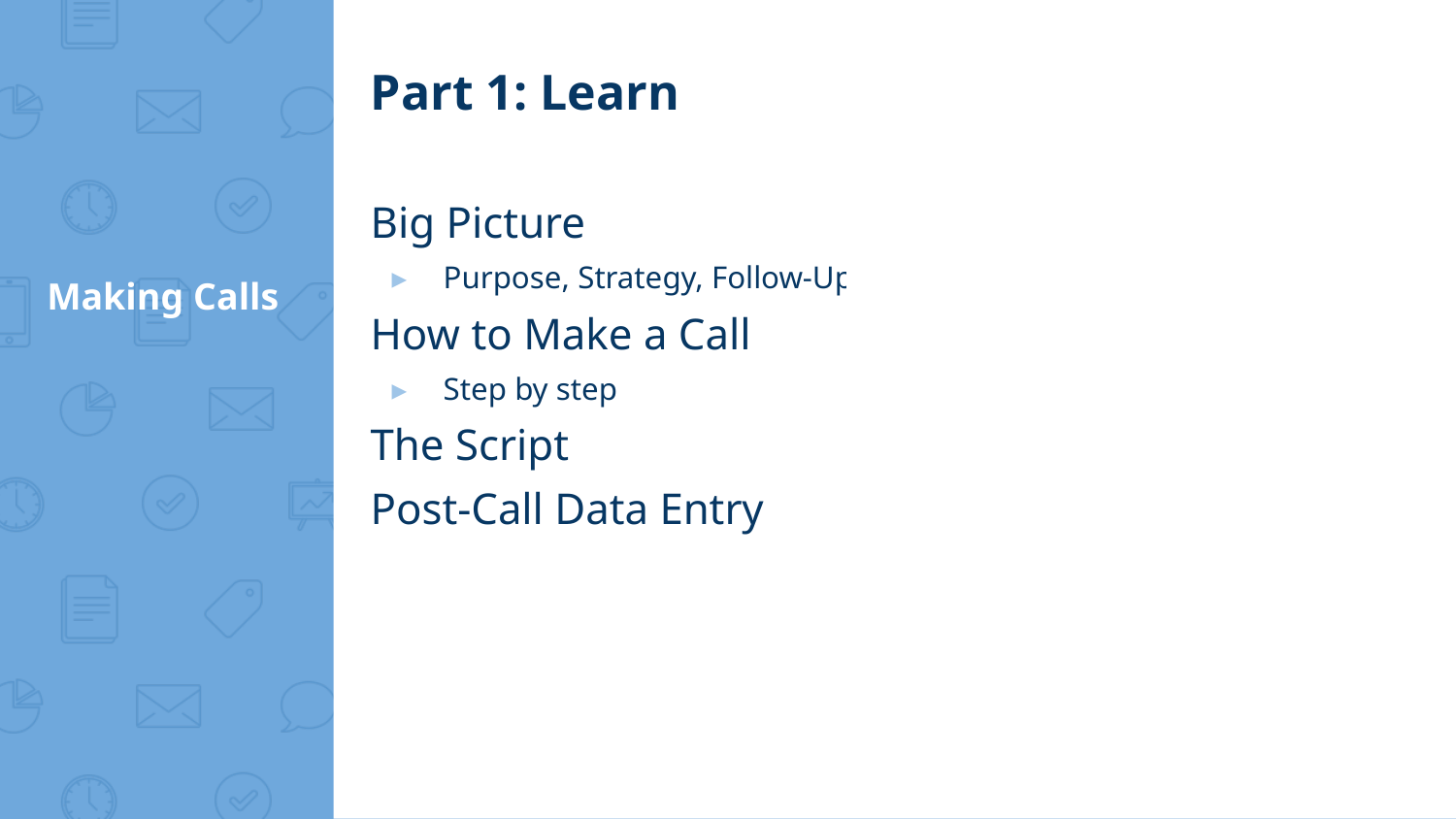

Part 1: Learn
Big Picture
Purpose, Strategy, Follow-Up
How to Make a Call
Step by step
The Script
Post-Call Data Entry
Part 2: Practice
PRAPARE data entry
Demonstration
Call Prep
Desktop management
Calling
Post Call Data Entry
# Making Calls

## Slide 24
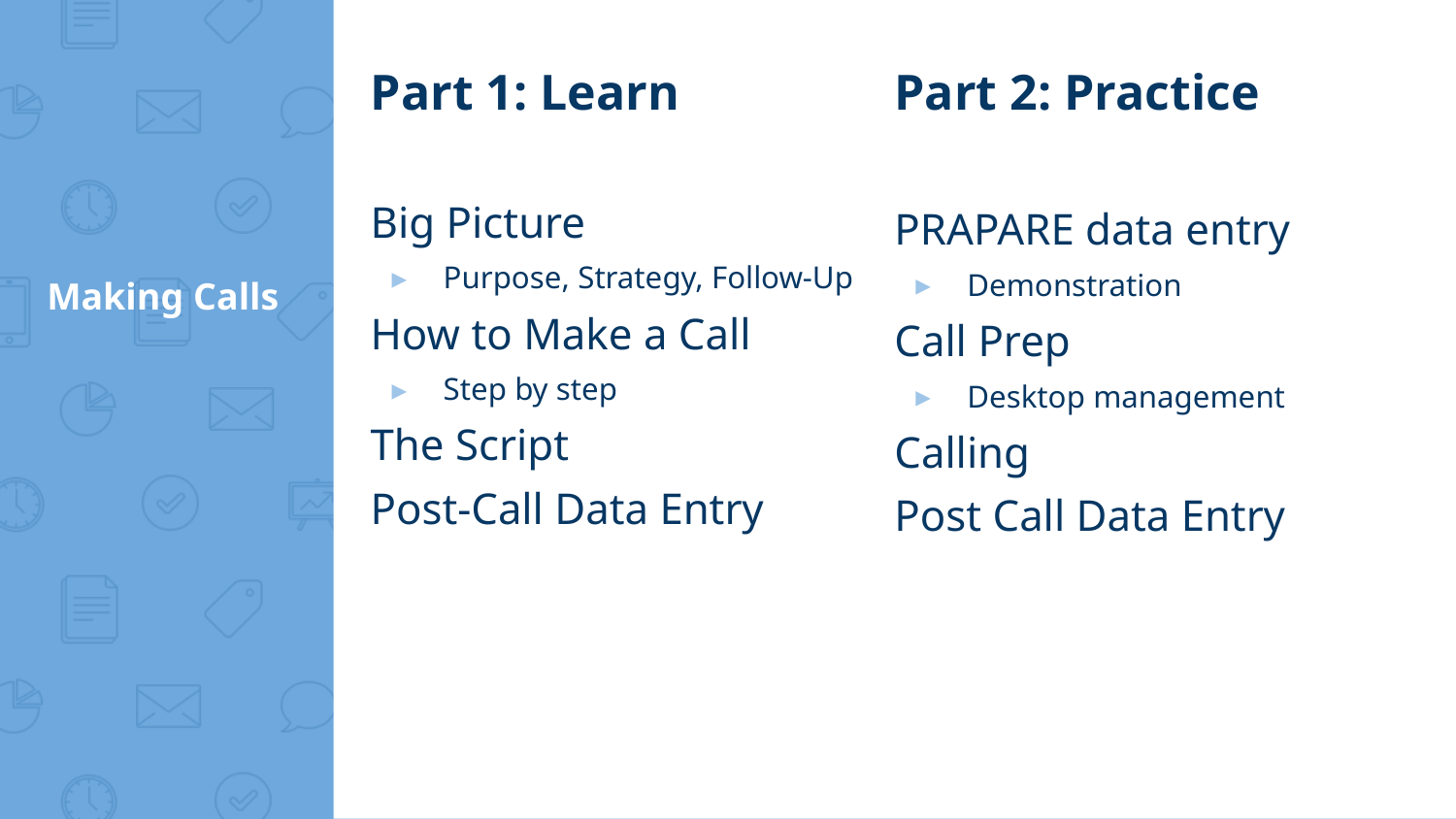

Part 1: Learn
Big Picture
Purpose, Strategy, Follow-Up
How to Make a Call
Step by step
The Script
Post-Call Data Entry
Part 2: Practice
PRAPARE data entry
Demonstration
Call Prep
Desktop management
Calling
Post Call Data Entry
# Making Calls

## Slide 25
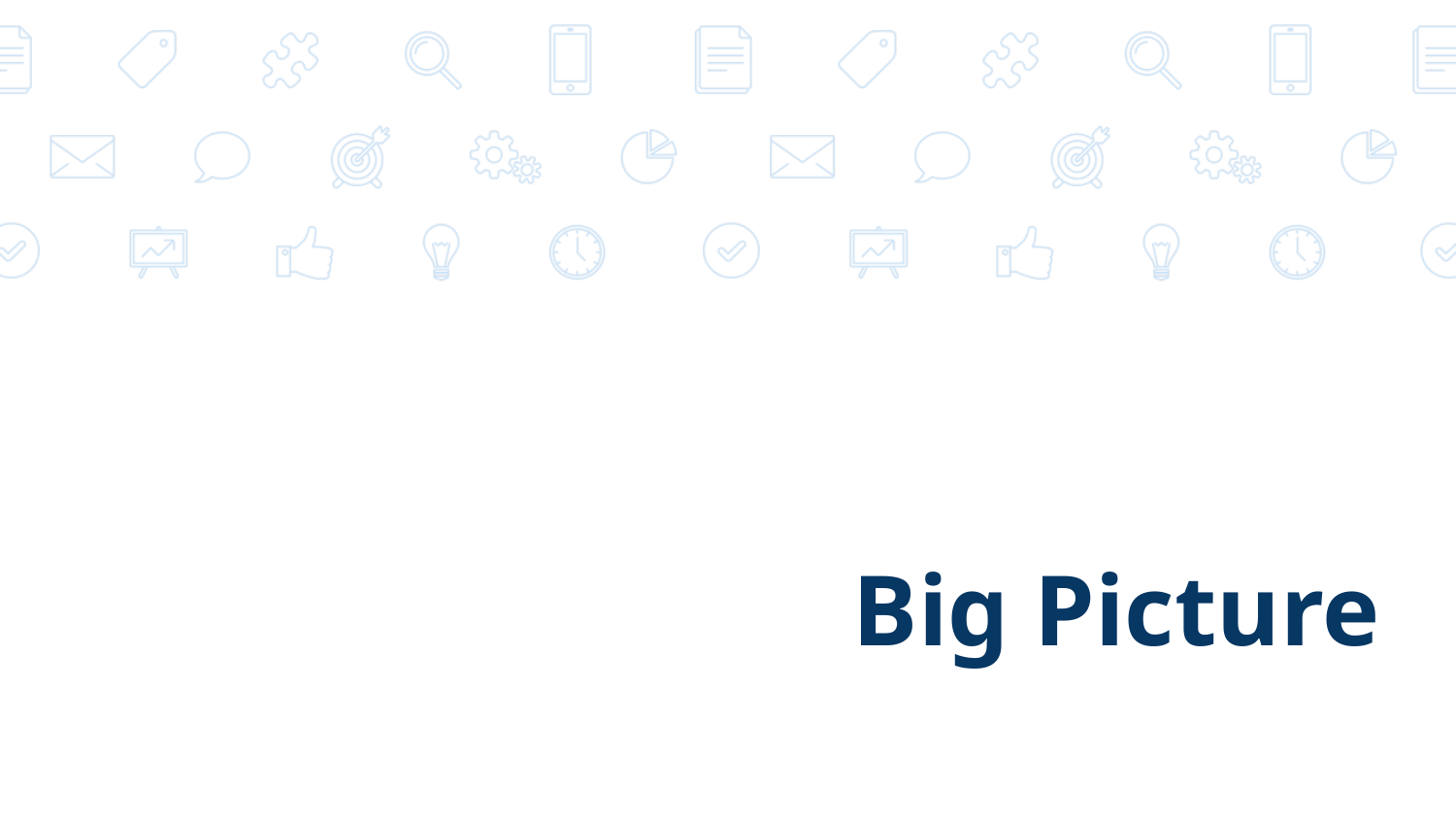

# Big Picture

## Slide 26
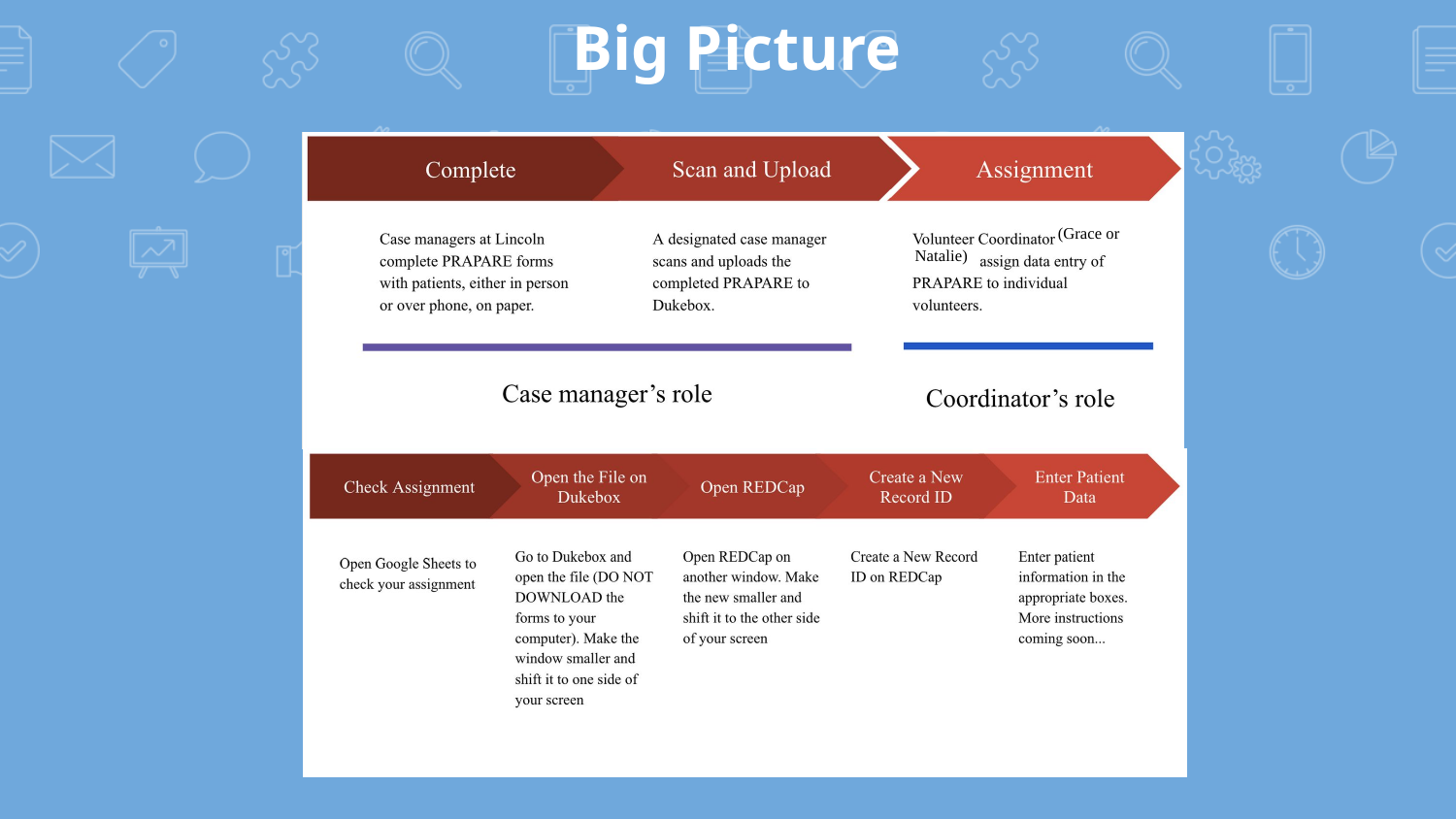

# Big Picture
(Grace or
Natalie)

## Slide 27
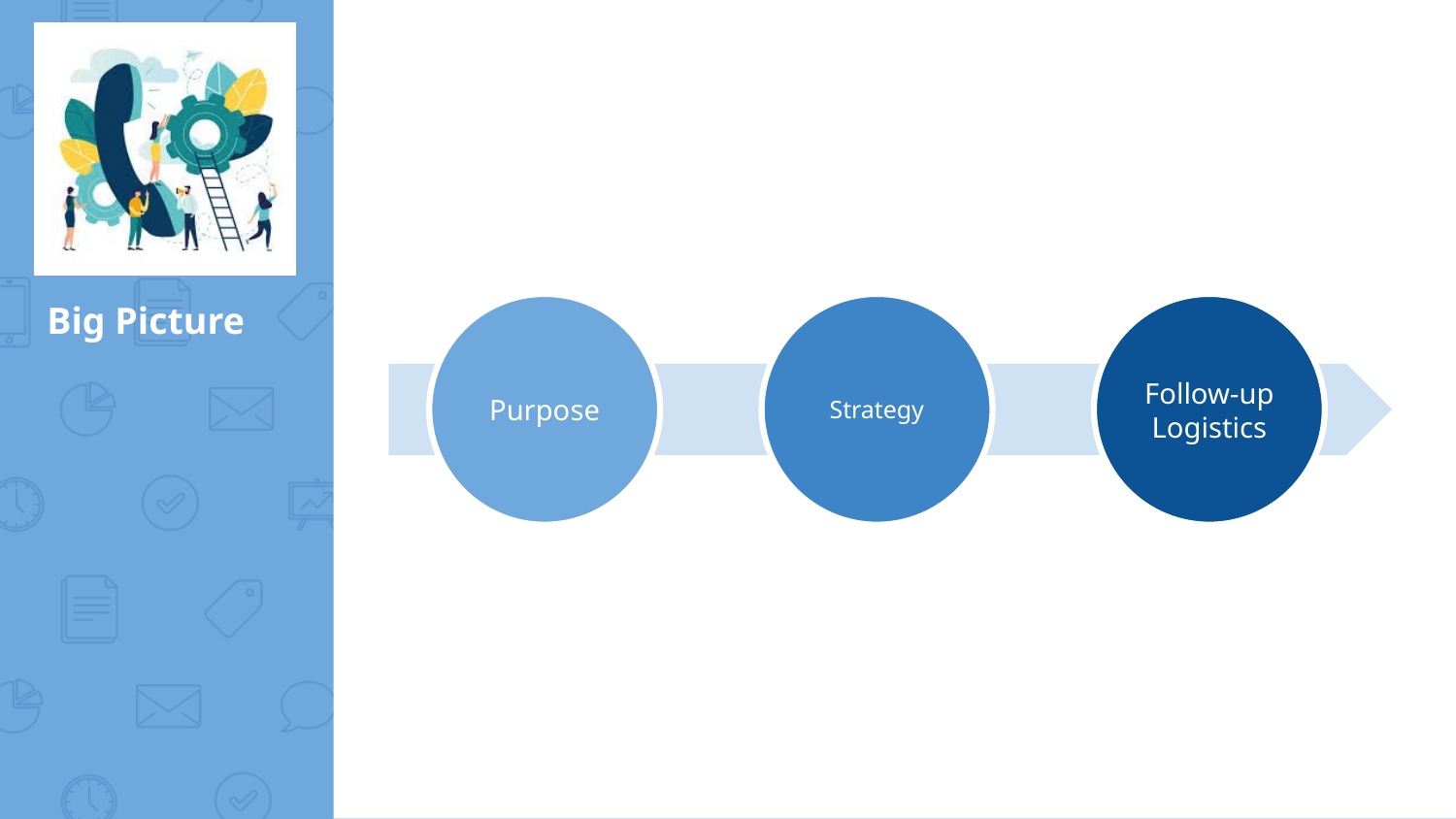

# Big Picture
Purpose
Strategy
Follow-up Logistics

## Slide 28
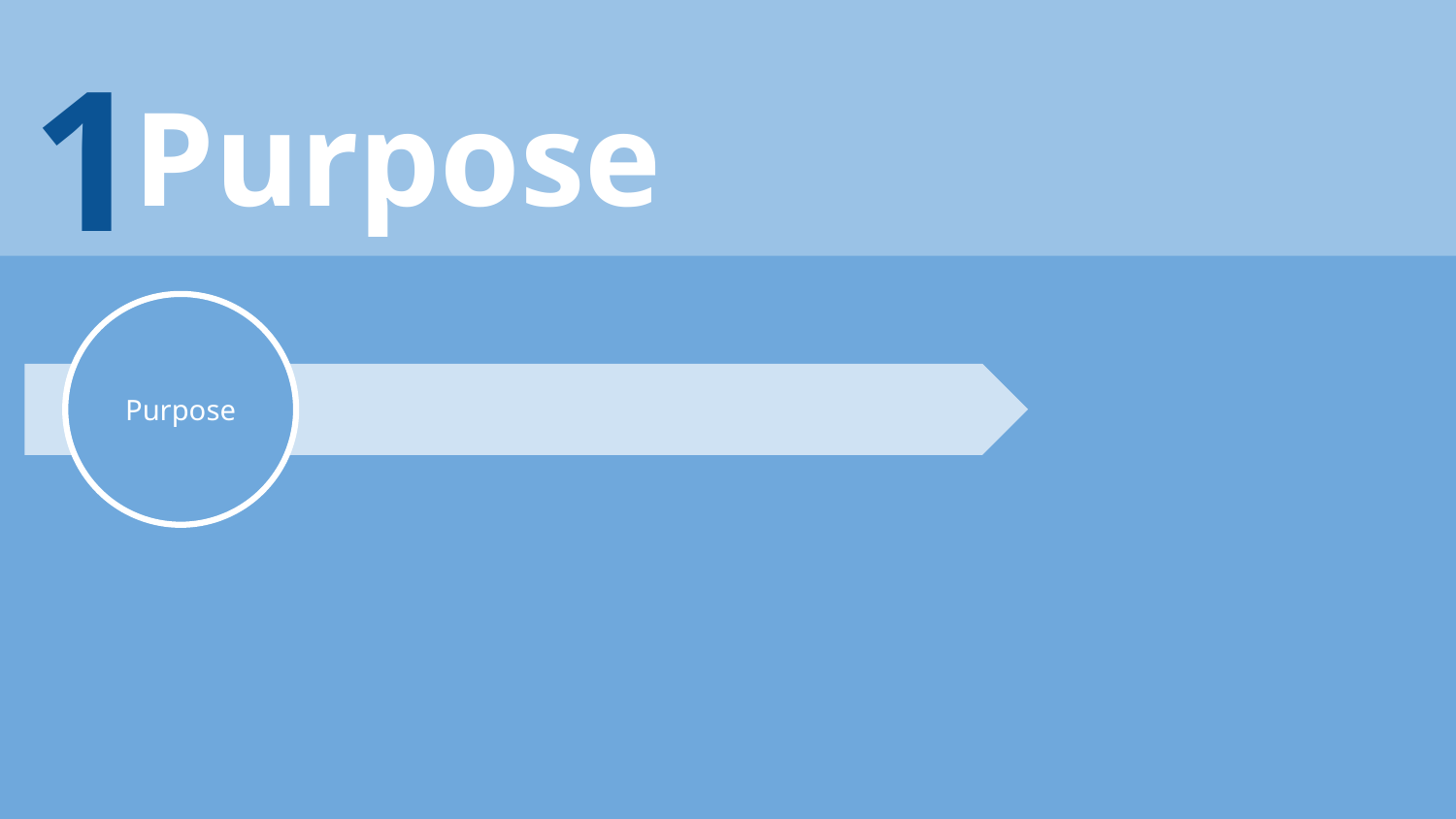

1
Purpose
Purpose

## Slide 29
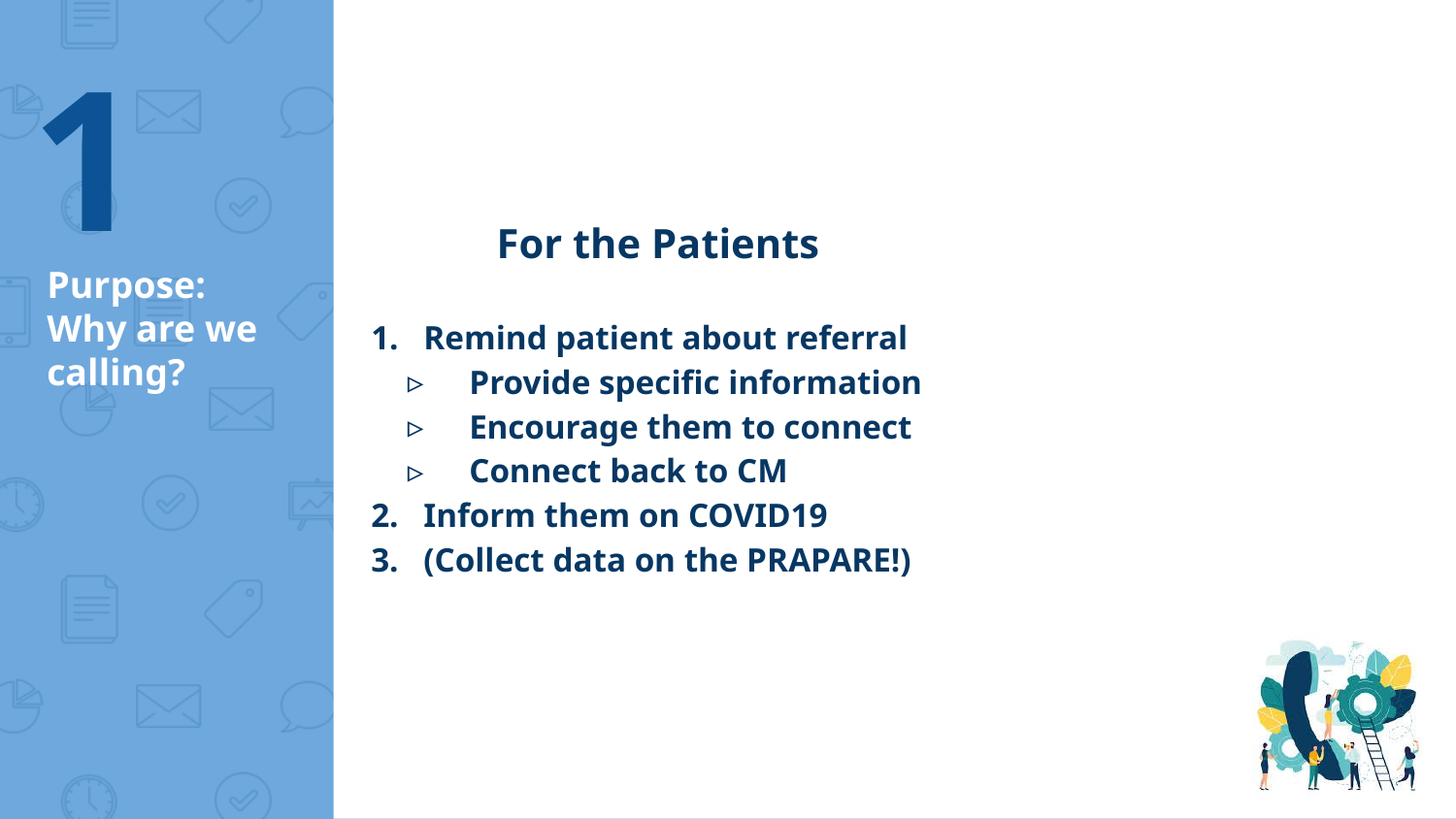

1
For the Patients
Remind patient about referral
Provide specific information
Encourage them to connect
Connect back to CM
Inform them on COVID19
(Collect data on the PRAPARE!)
# Purpose:
Why are we calling?

## Slide 30
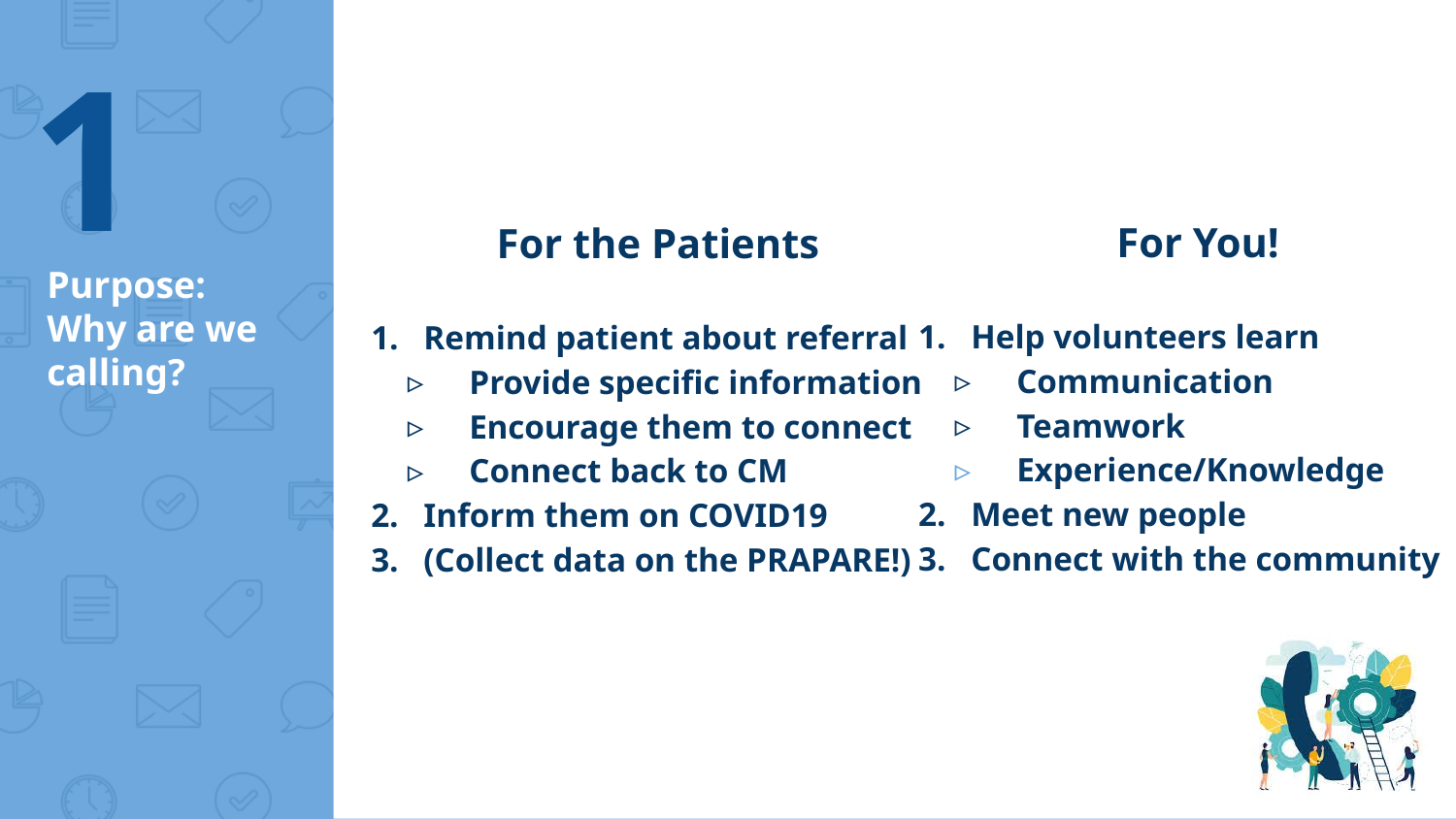

1
For You!
Help volunteers learn
Communication
Teamwork
Experience/Knowledge
Meet new people
Connect with the community
For the Patients
Remind patient about referral
Provide specific information
Encourage them to connect
Connect back to CM
Inform them on COVID19
(Collect data on the PRAPARE!)
# Purpose:
Why are we calling?

## Slide 31
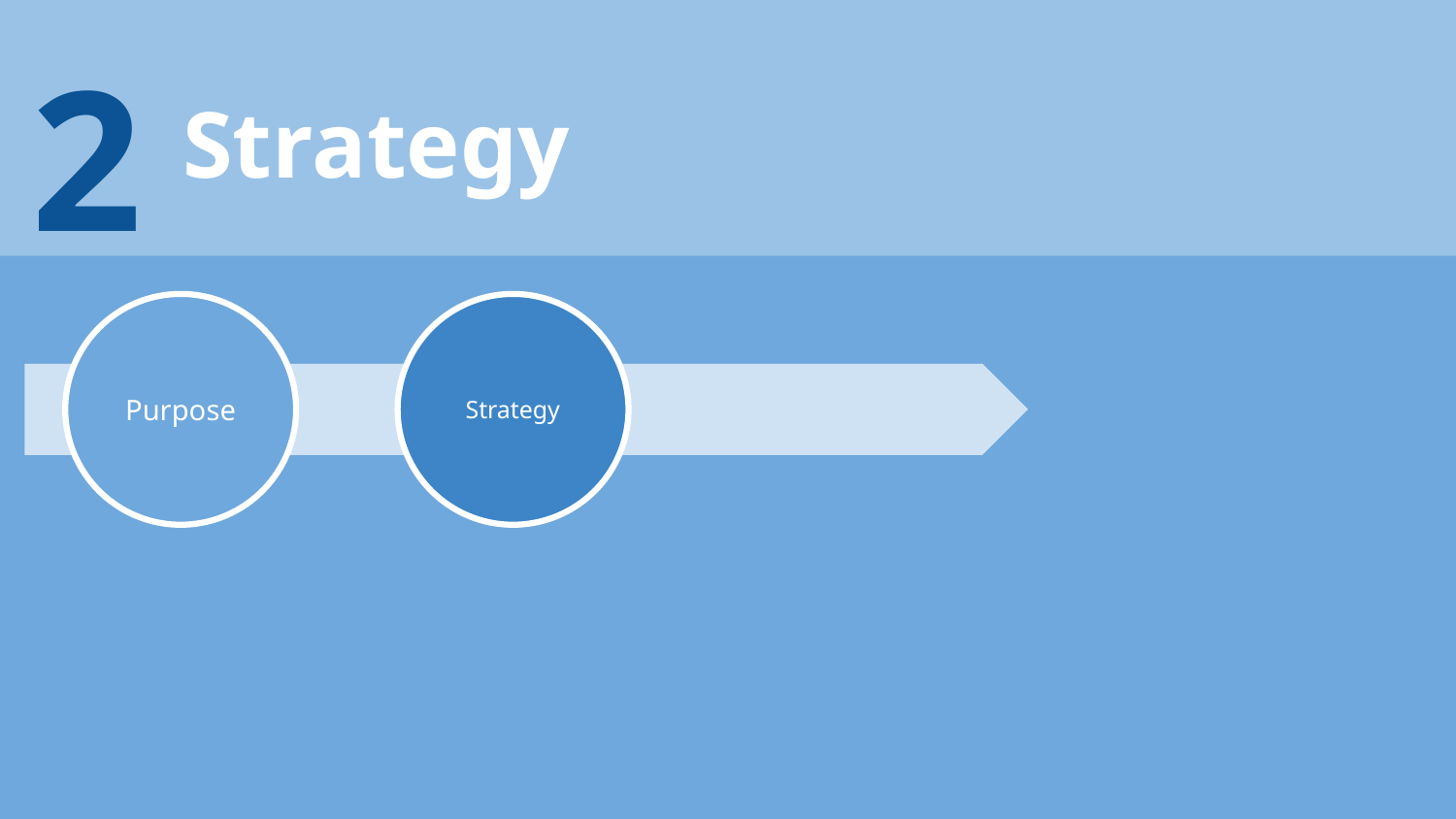

2
Strategy
Purpose
Strategy

## Slide 32
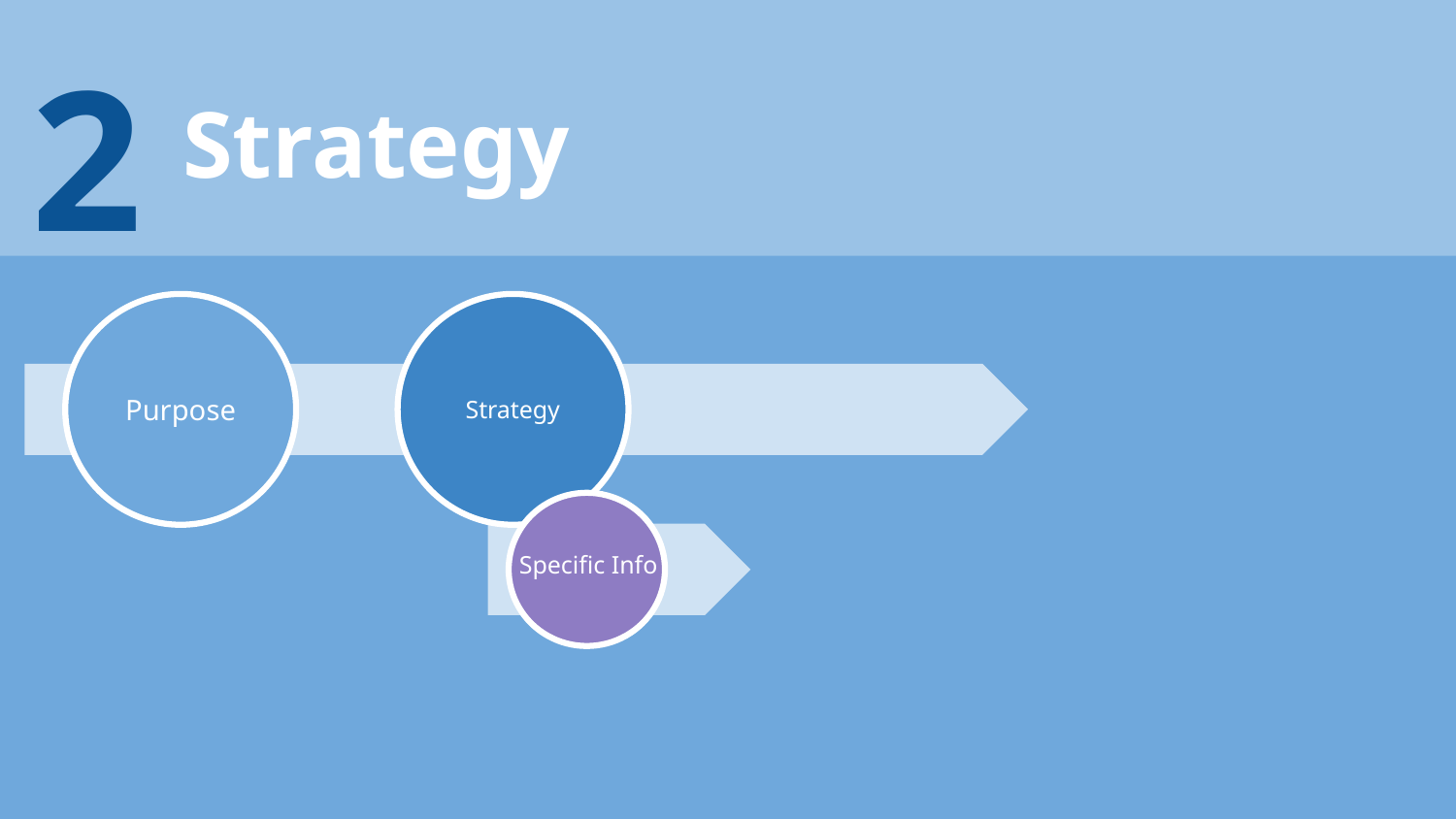

2
Strategy
Purpose
Strategy
Specific Info

## Slide 33
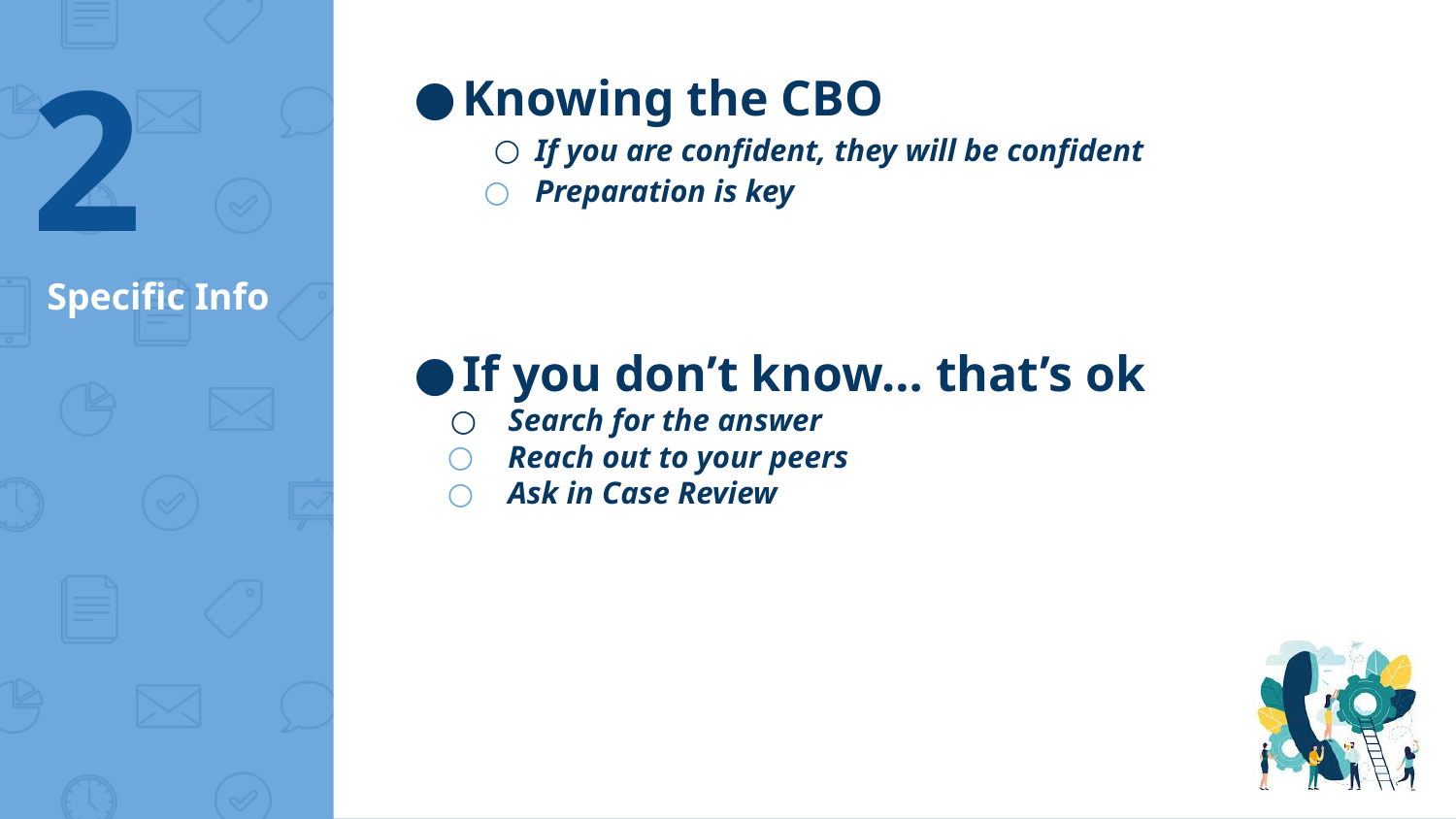

2
Knowing the CBO
If you are confident, they will be confident
Preparation is key
If you don’t know… that’s ok
Search for the answer
Reach out to your peers
Ask in Case Review
# Specific Info

## Slide 34
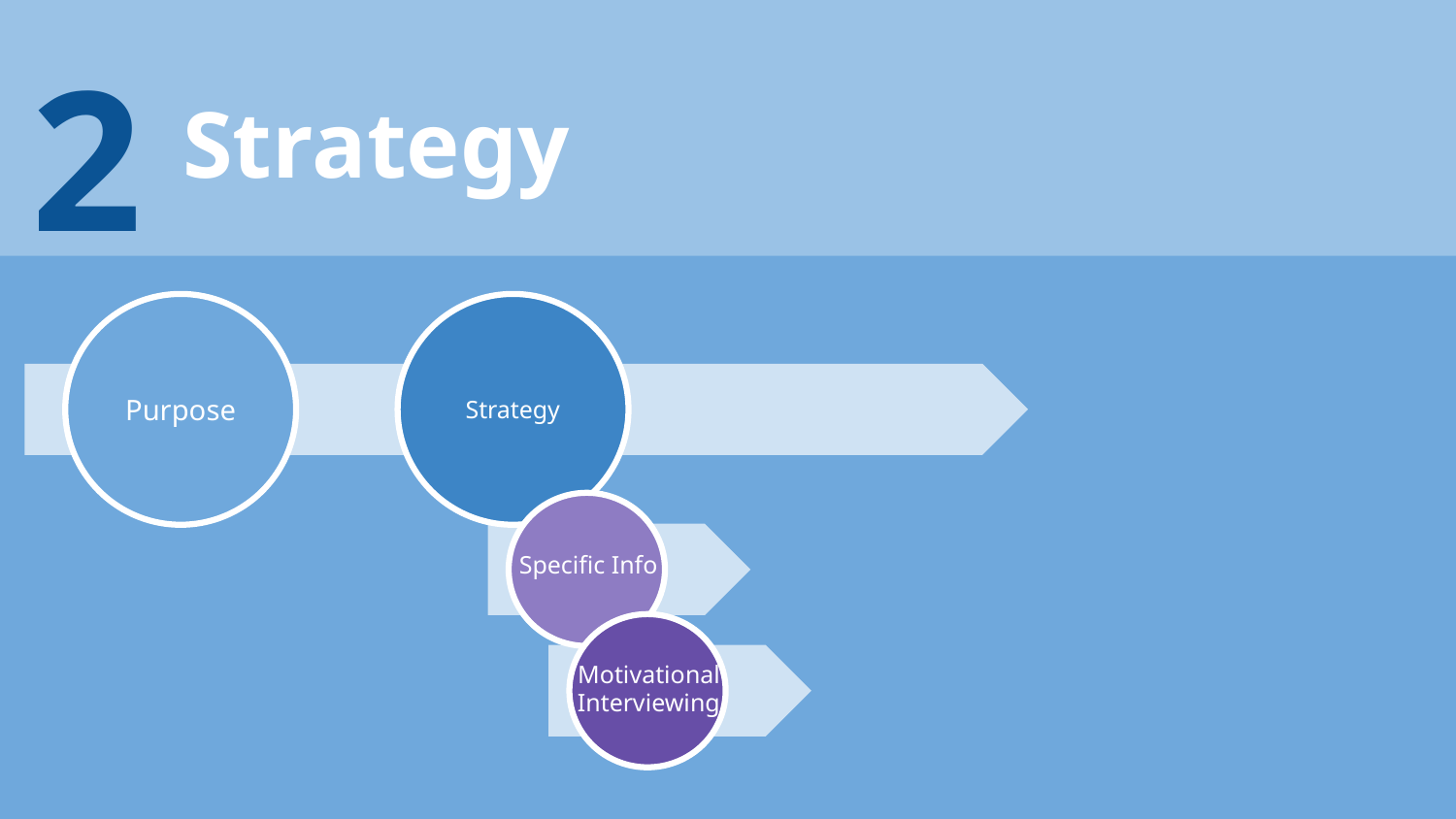

2
Strategy
Purpose
Strategy
Specific Info
Motivational
Interviewing

## Slide 35
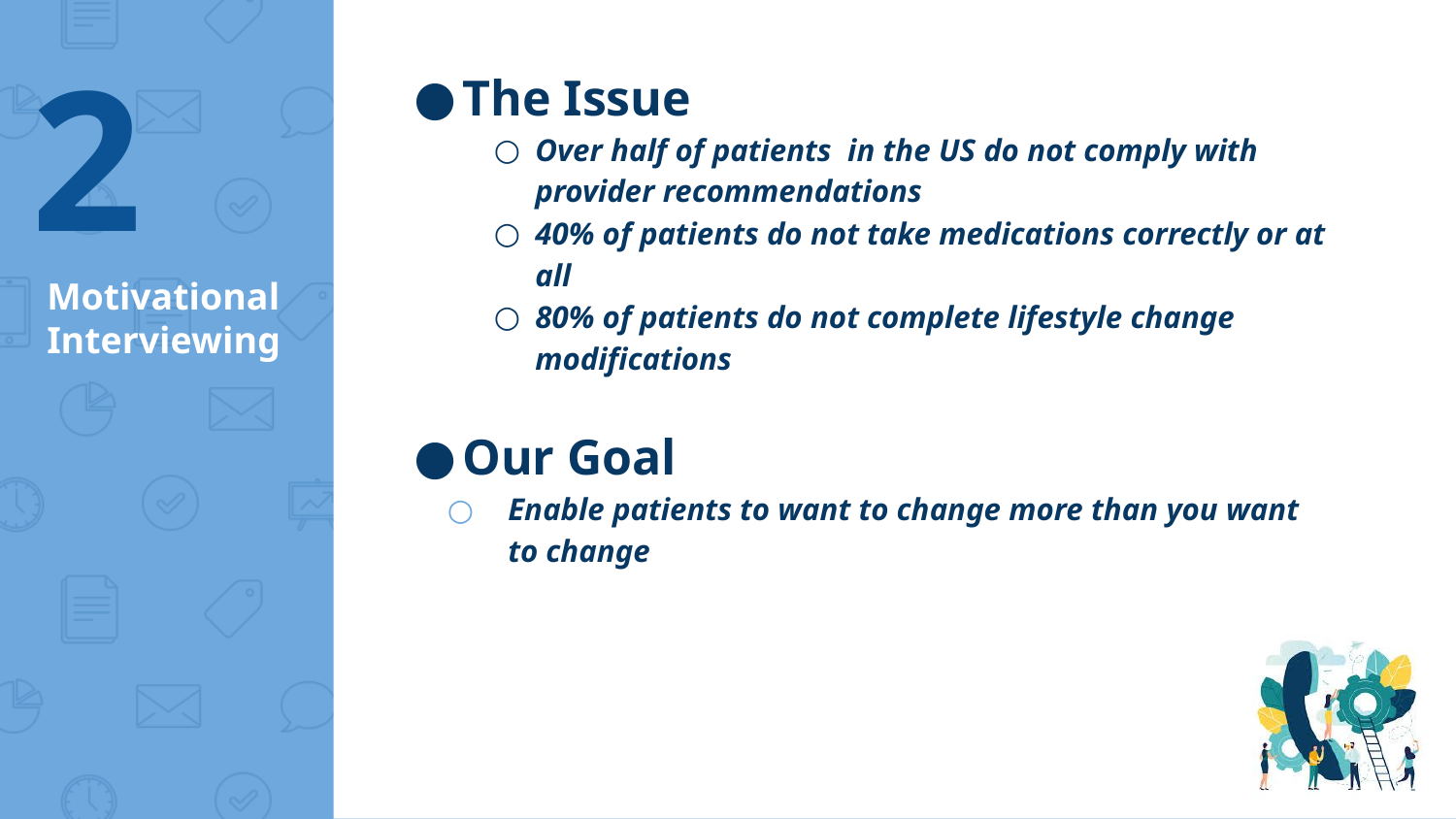

2
The Issue
Over half of patients in the US do not comply with provider recommendations
40% of patients do not take medications correctly or at all
80% of patients do not complete lifestyle change modifications
Our Goal
Enable patients to want to change more than you want to change
# Motivational Interviewing

## Slide 36
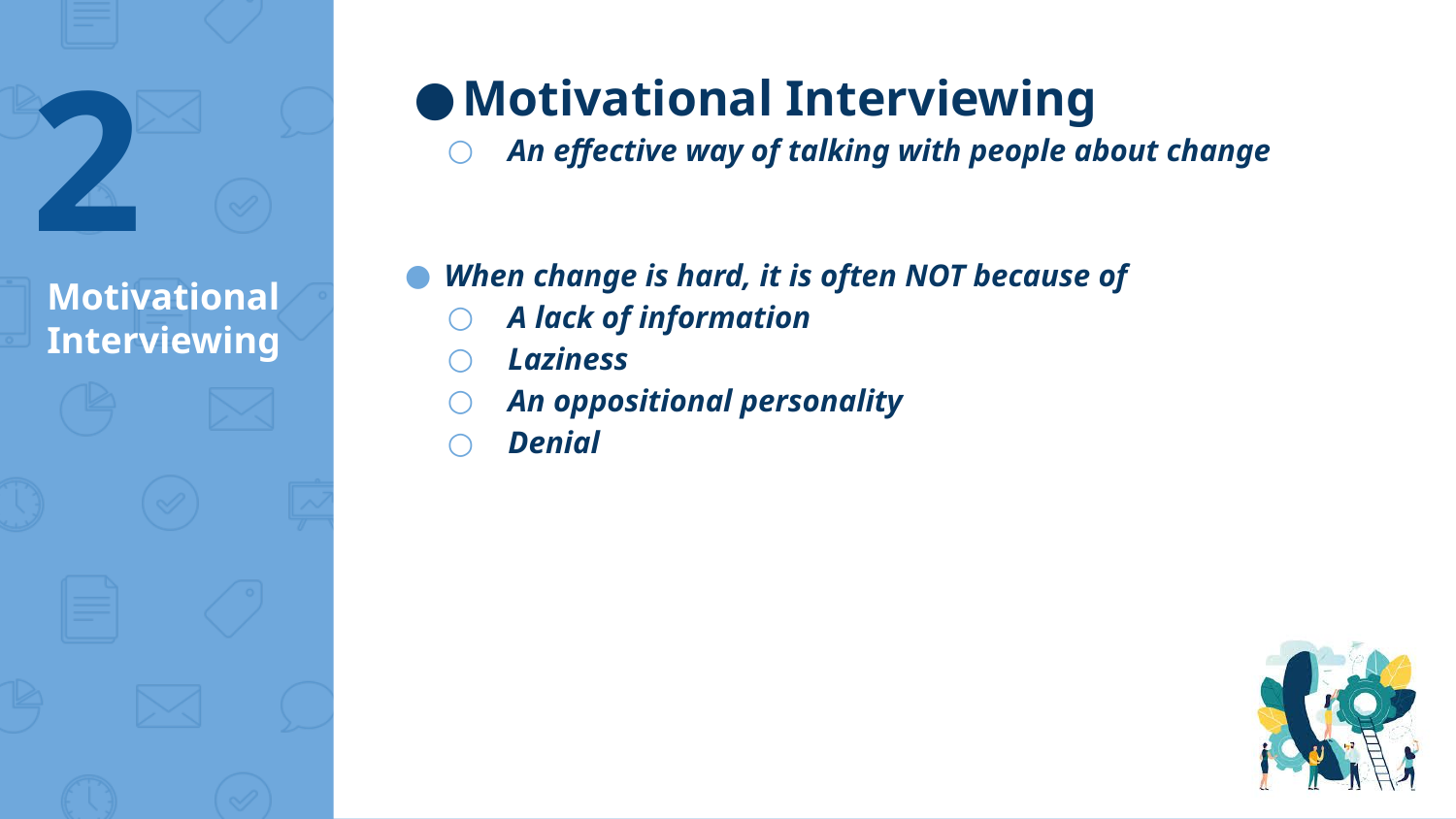

2
Motivational Interviewing
An effective way of talking with people about change
When change is hard, it is often NOT because of
A lack of information
Laziness
An oppositional personality
Denial
# Motivational Interviewing

## Slide 37
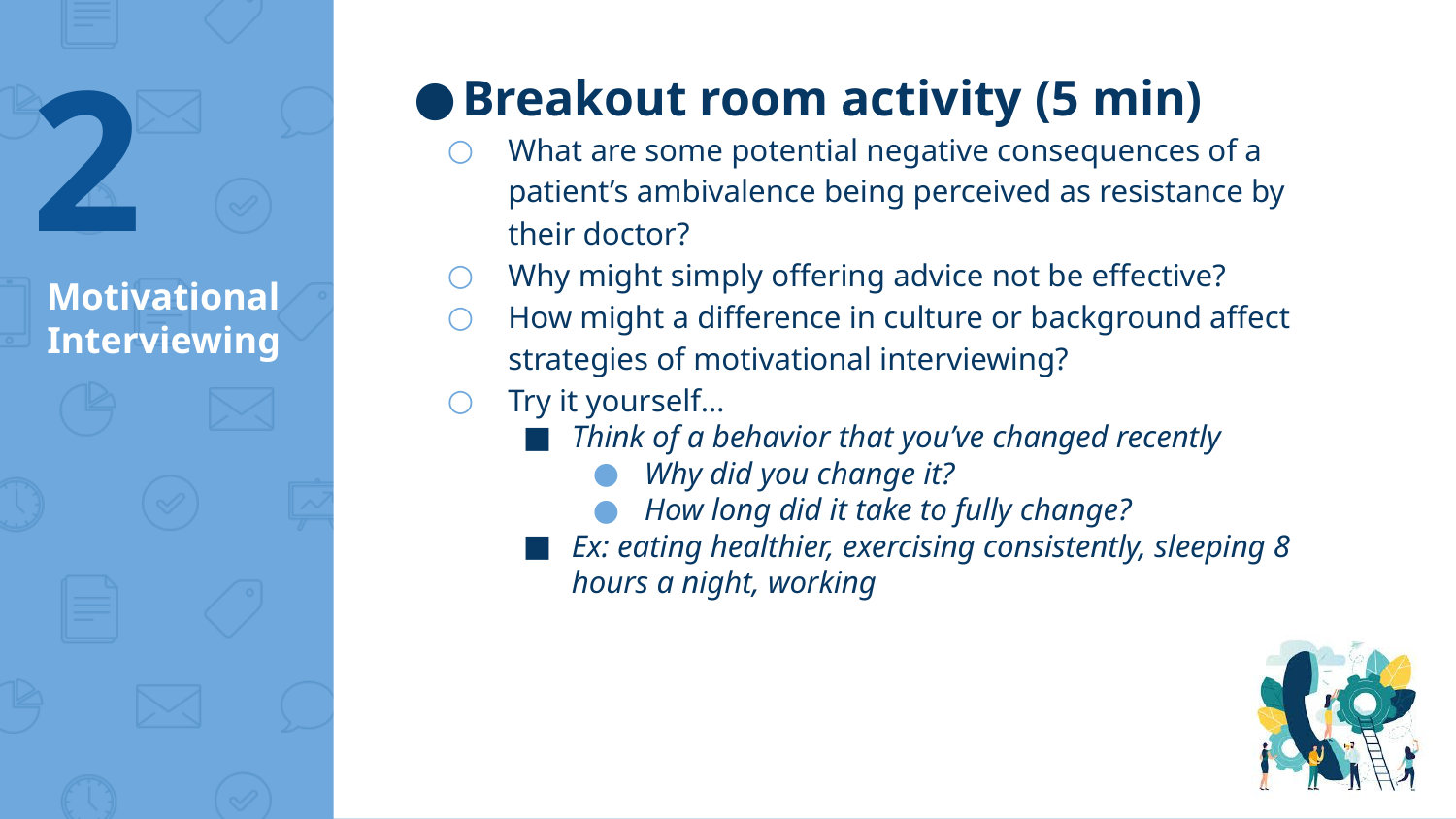

2
Breakout room activity (5 min)
What are some potential negative consequences of a patient’s ambivalence being perceived as resistance by their doctor?
Why might simply offering advice not be effective?
How might a difference in culture or background affect strategies of motivational interviewing?
Try it yourself…
Think of a behavior that you’ve changed recently
Why did you change it?
How long did it take to fully change?
Ex: eating healthier, exercising consistently, sleeping 8 hours a night, working
# Motivational Interviewing

## Slide 38
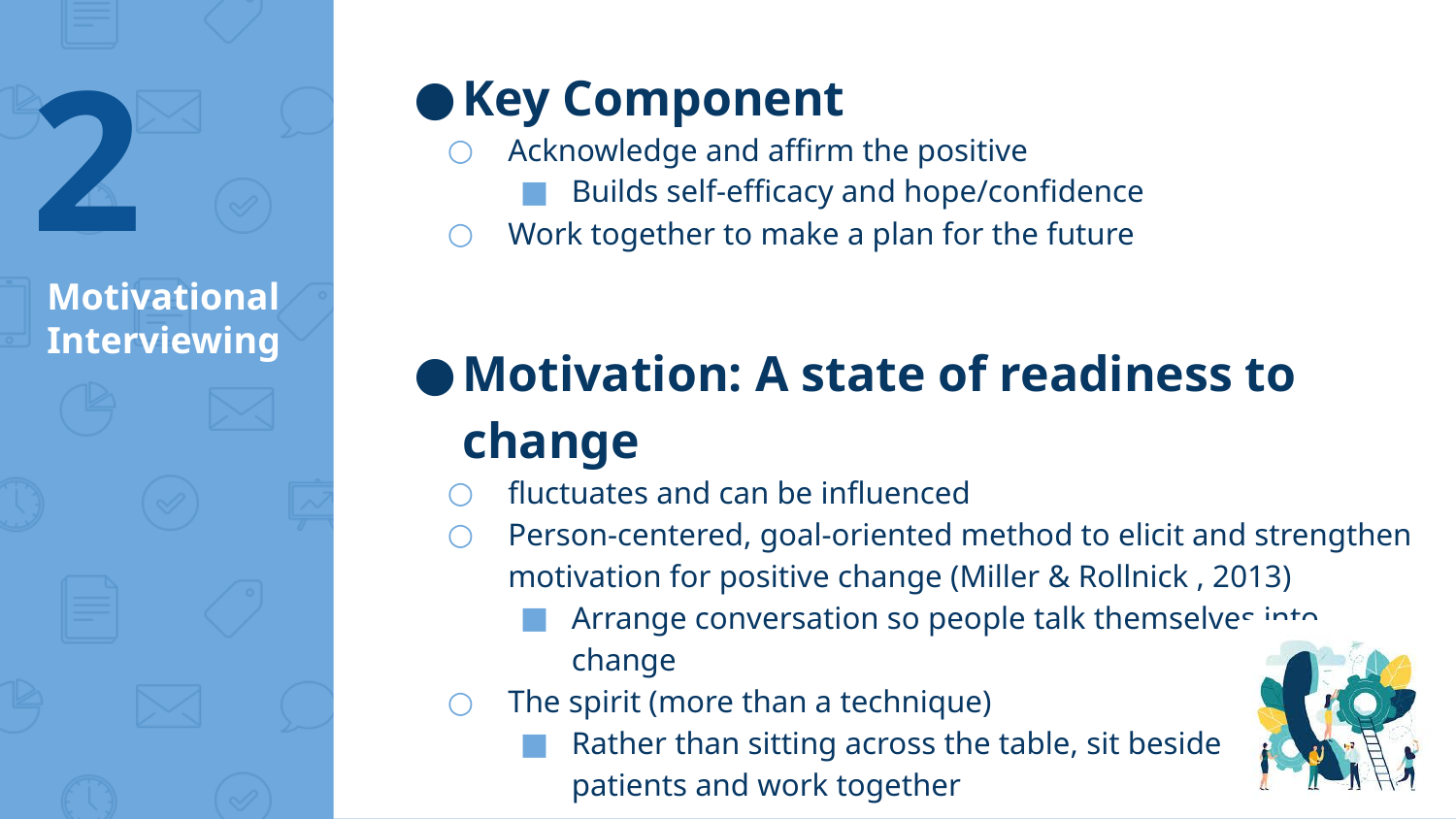

2
Key Component
Acknowledge and affirm the positive
Builds self-efficacy and hope/confidence
Work together to make a plan for the future
Motivation: A state of readiness to change
fluctuates and can be influenced
Person-centered, goal-oriented method to elicit and strengthen motivation for positive change (Miller & Rollnick , 2013)
Arrange conversation so people talk themselves into change
The spirit (more than a technique)
Rather than sitting across the table, sit beside
patients and work together
# Motivational Interviewing

## Slide 39
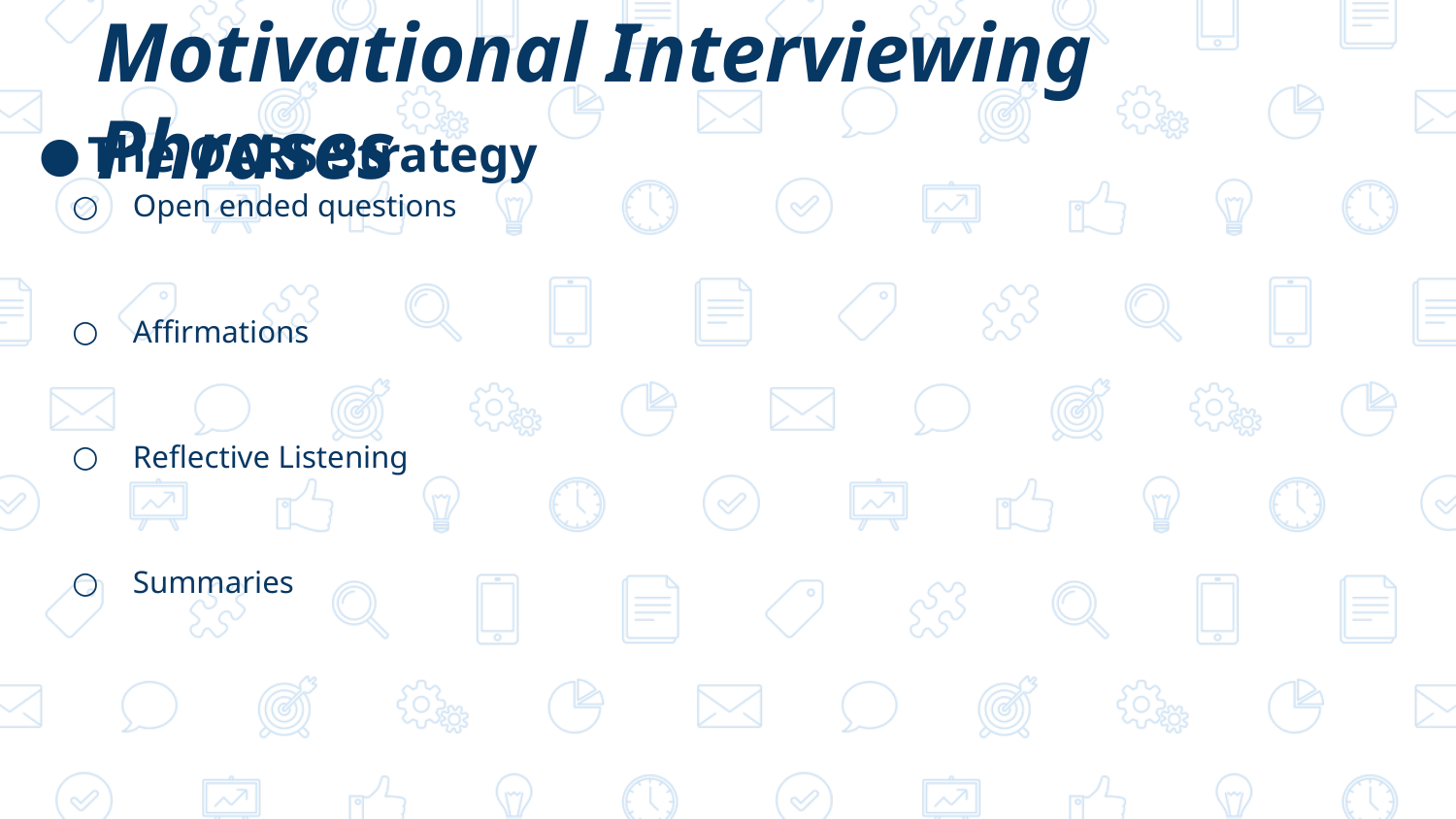

Motivational Interviewing Phrases
The OARS Strategy
Open ended questions
Affirmations
Reflective Listening
Summaries

## Slide 40
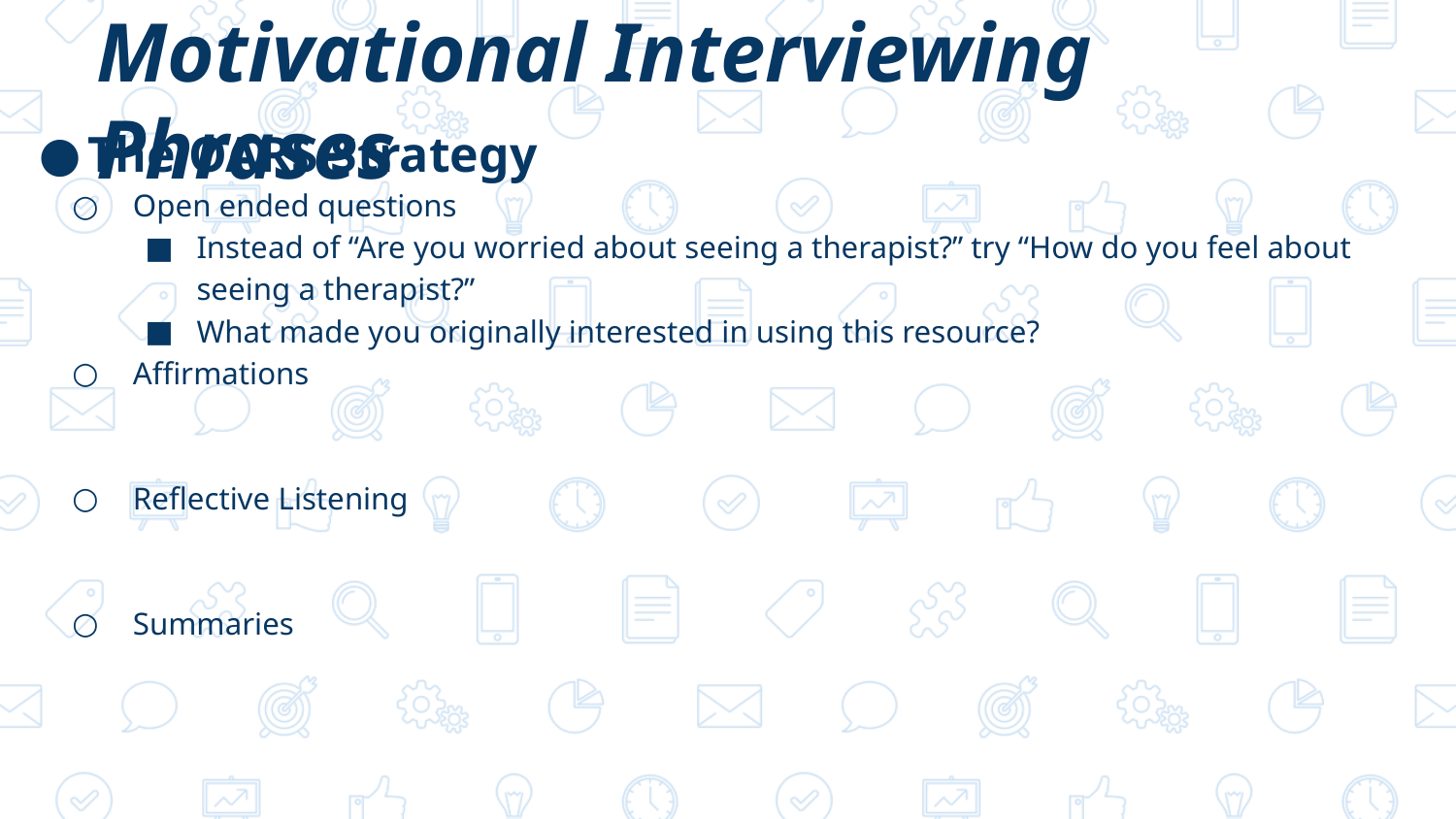

Motivational Interviewing Phrases
The OARS Strategy
Open ended questions
Instead of “Are you worried about seeing a therapist?” try “How do you feel about seeing a therapist?”
What made you originally interested in using this resource?
Affirmations
Reflective Listening
Summaries

## Slide 41
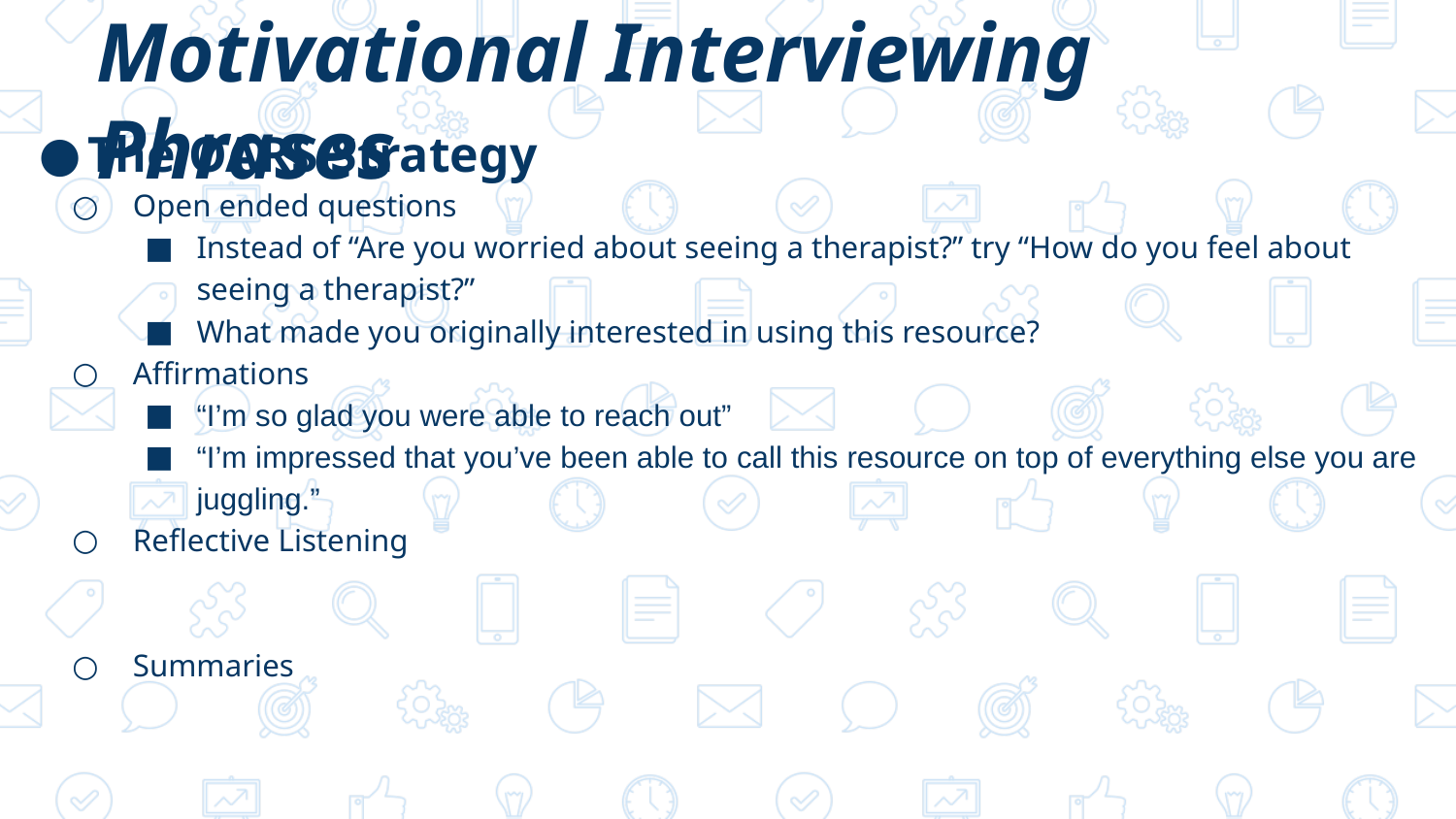

Motivational Interviewing Phrases
The OARS Strategy
Open ended questions
Instead of “Are you worried about seeing a therapist?” try “How do you feel about seeing a therapist?”
What made you originally interested in using this resource?
Affirmations
“I’m so glad you were able to reach out”
“I’m impressed that you’ve been able to call this resource on top of everything else you are juggling.”
Reflective Listening
Summaries

## Slide 42
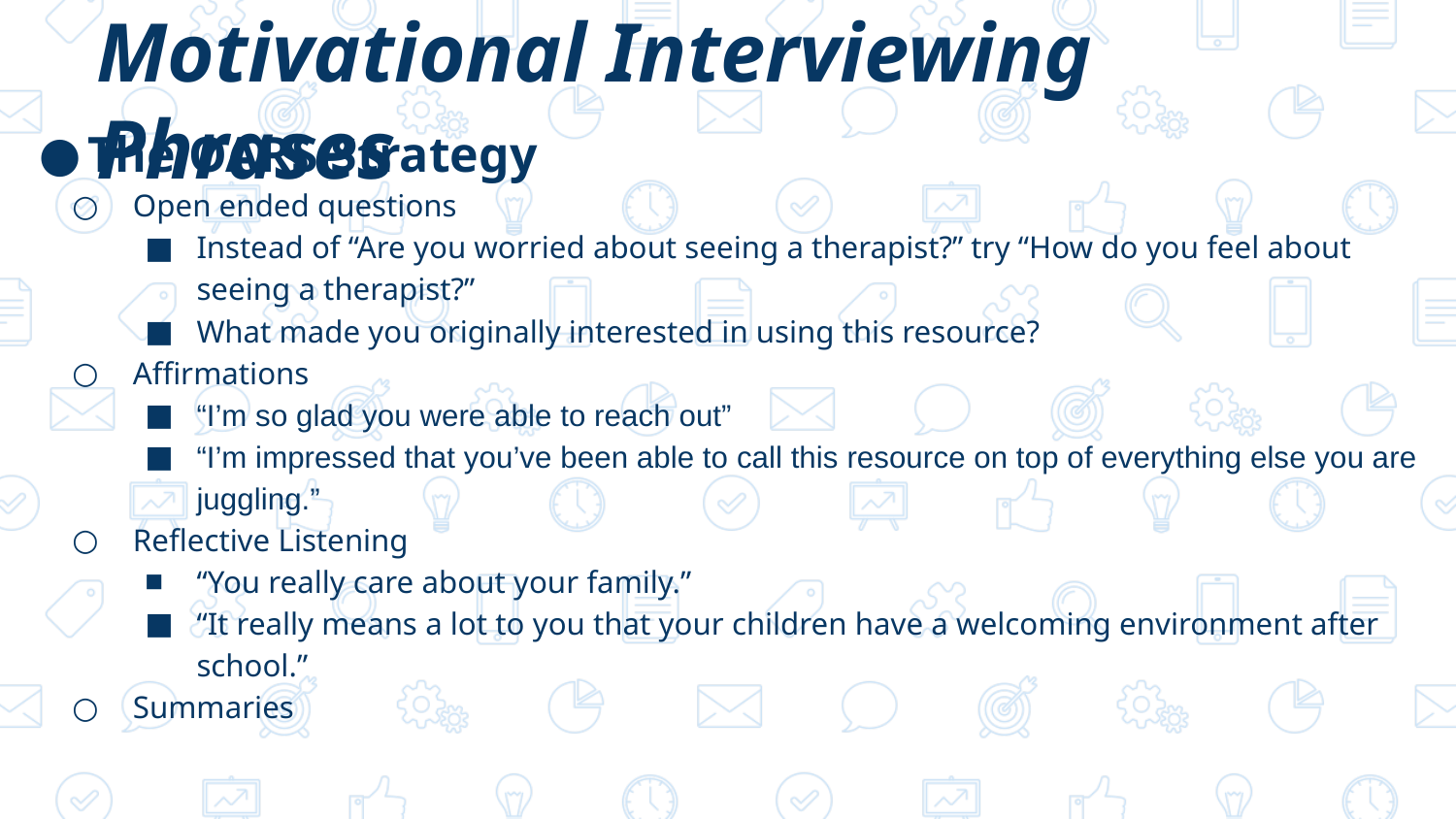

Motivational Interviewing Phrases
The OARS Strategy
Open ended questions
Instead of “Are you worried about seeing a therapist?” try “How do you feel about seeing a therapist?”
What made you originally interested in using this resource?
Affirmations
“I’m so glad you were able to reach out”
“I’m impressed that you’ve been able to call this resource on top of everything else you are juggling.”
Reflective Listening
“You really care about your family.”
“It really means a lot to you that your children have a welcoming environment after school.”
Summaries

## Slide 43
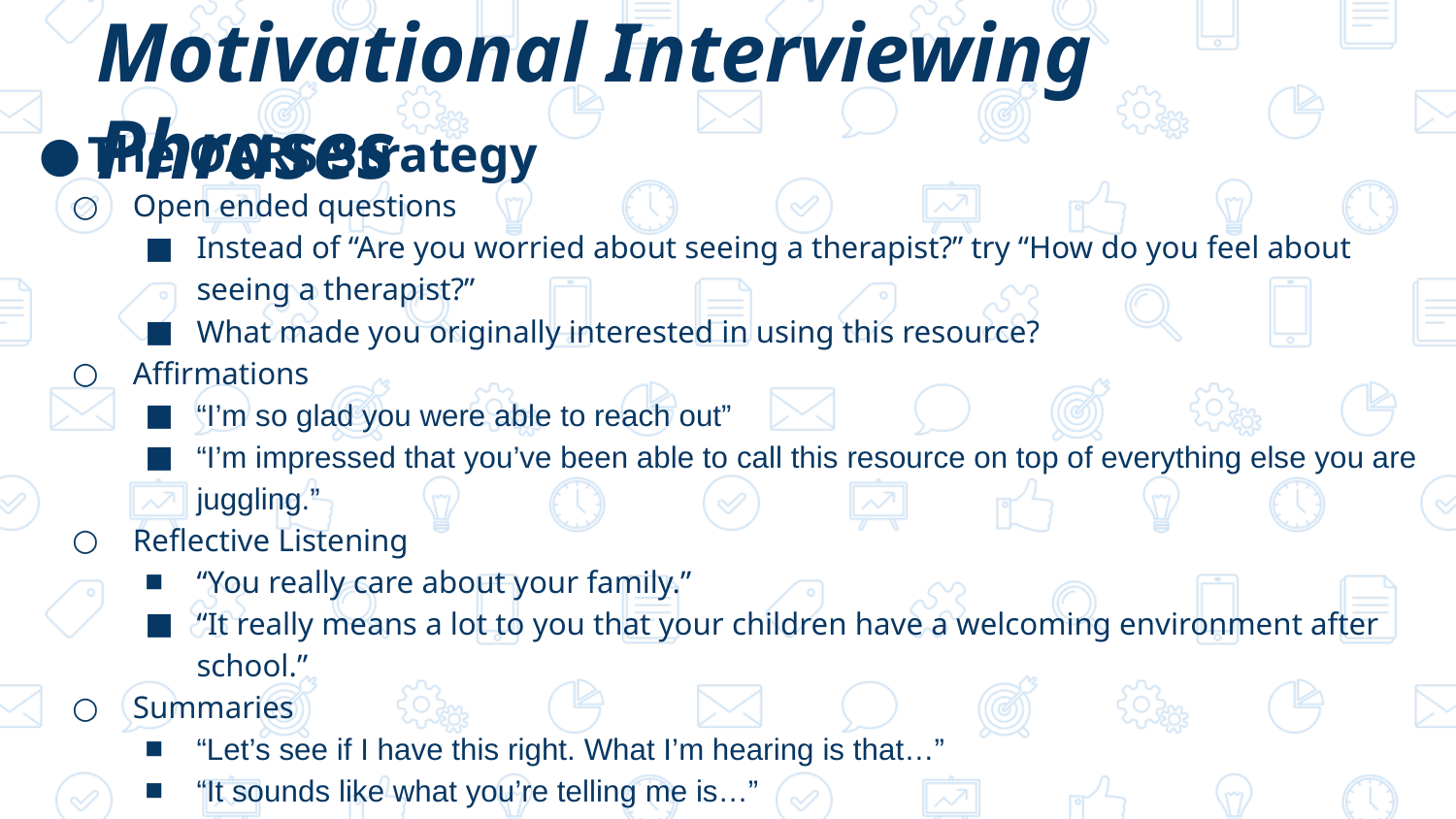

Motivational Interviewing Phrases
The OARS Strategy
Open ended questions
Instead of “Are you worried about seeing a therapist?” try “How do you feel about seeing a therapist?”
What made you originally interested in using this resource?
Affirmations
“I’m so glad you were able to reach out”
“I’m impressed that you’ve been able to call this resource on top of everything else you are juggling.”
Reflective Listening
“You really care about your family.”
“It really means a lot to you that your children have a welcoming environment after school.”
Summaries
“Let’s see if I have this right. What I’m hearing is that…”
“It sounds like what you’re telling me is…”

## Slide 44
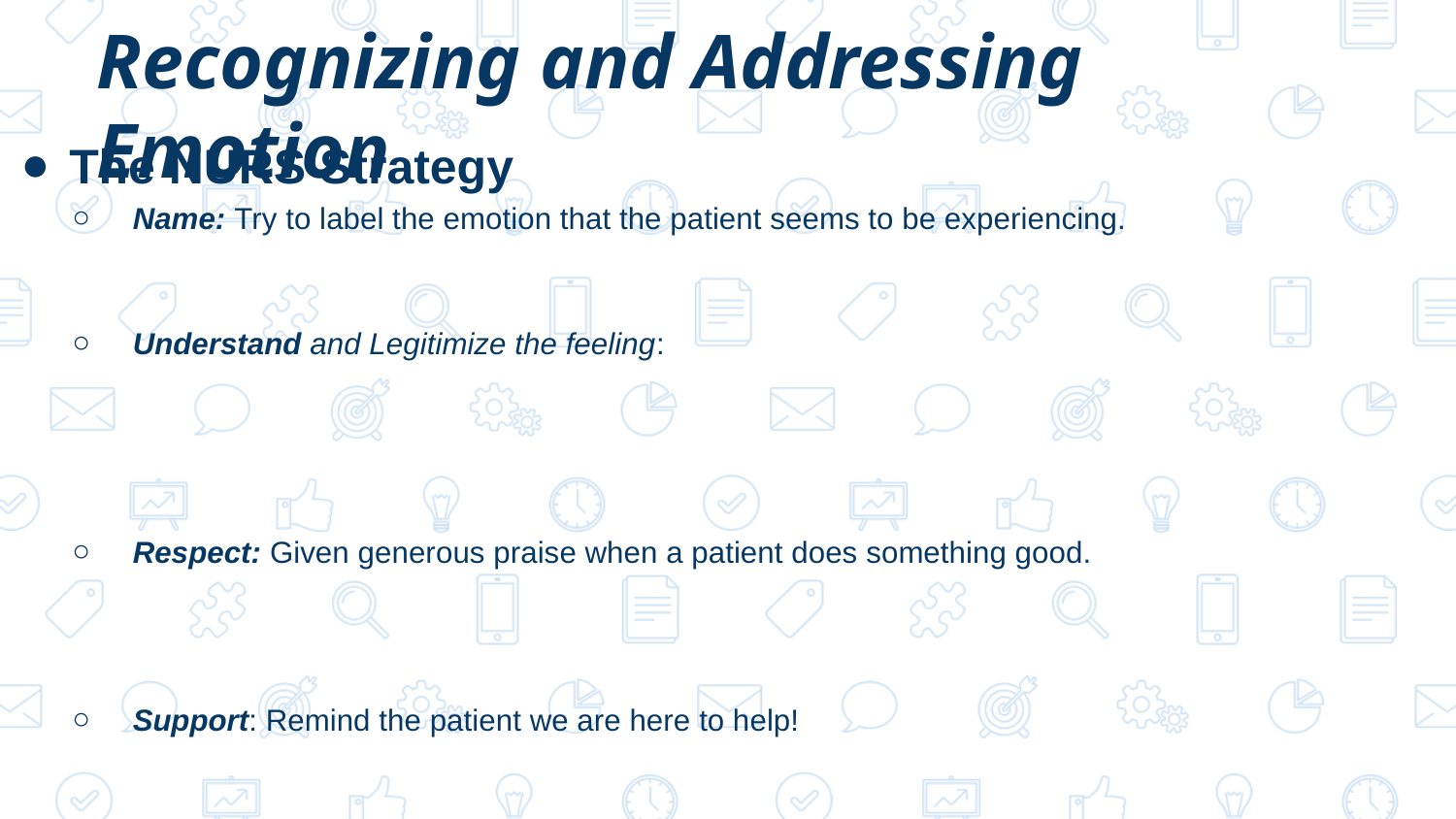

Recognizing and Addressing Emotion
The NURS Strategy
Name: Try to label the emotion that the patient seems to be experiencing.
Understand and Legitimize the feeling:
Respect: Given generous praise when a patient does something good.
Support: Remind the patient we are here to help!

## Slide 45
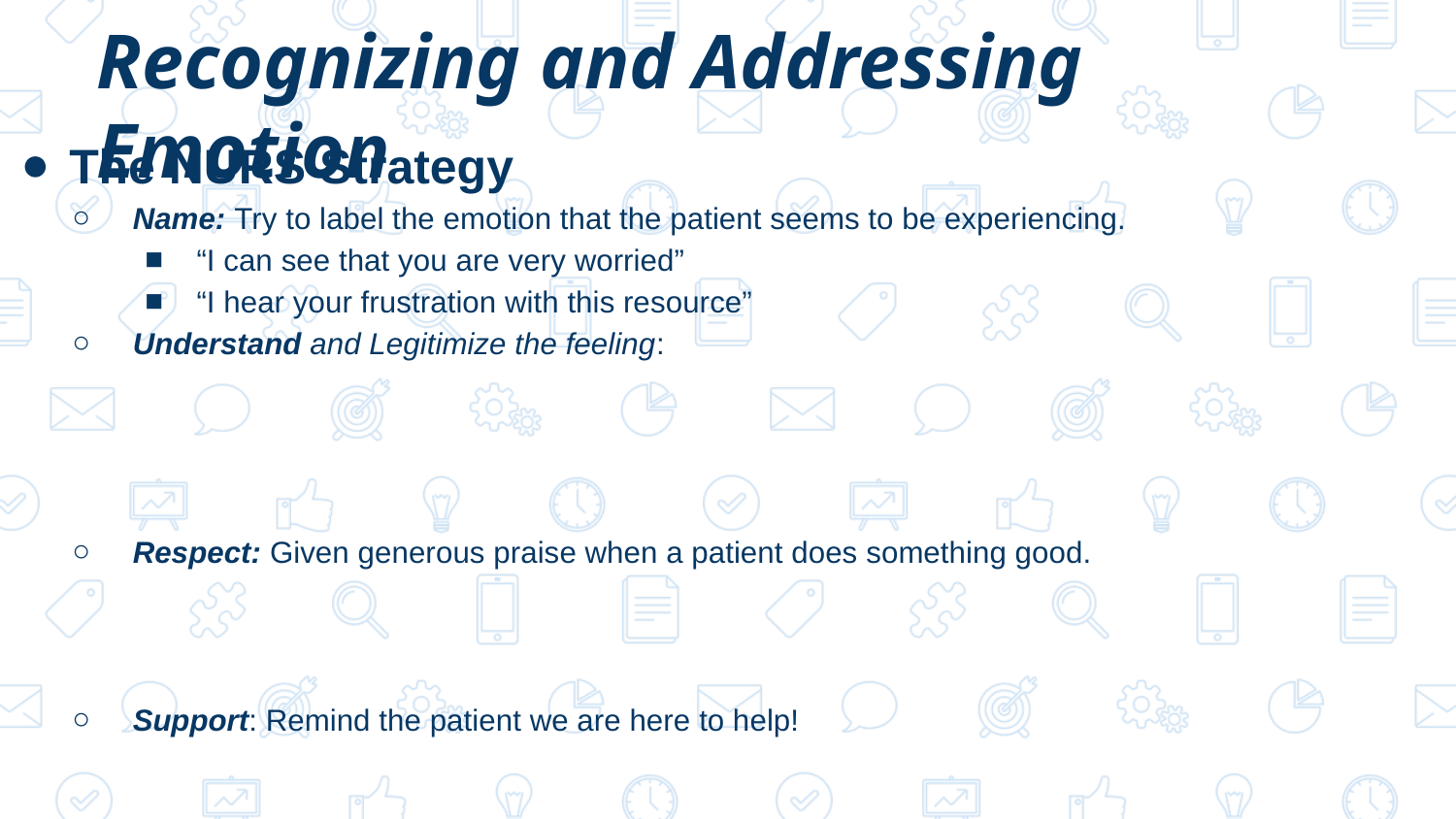

Recognizing and Addressing Emotion
The NURS Strategy
Name: Try to label the emotion that the patient seems to be experiencing.
“I can see that you are very worried”
“I hear your frustration with this resource”
Understand and Legitimize the feeling:
Respect: Given generous praise when a patient does something good.
Support: Remind the patient we are here to help!

## Slide 46
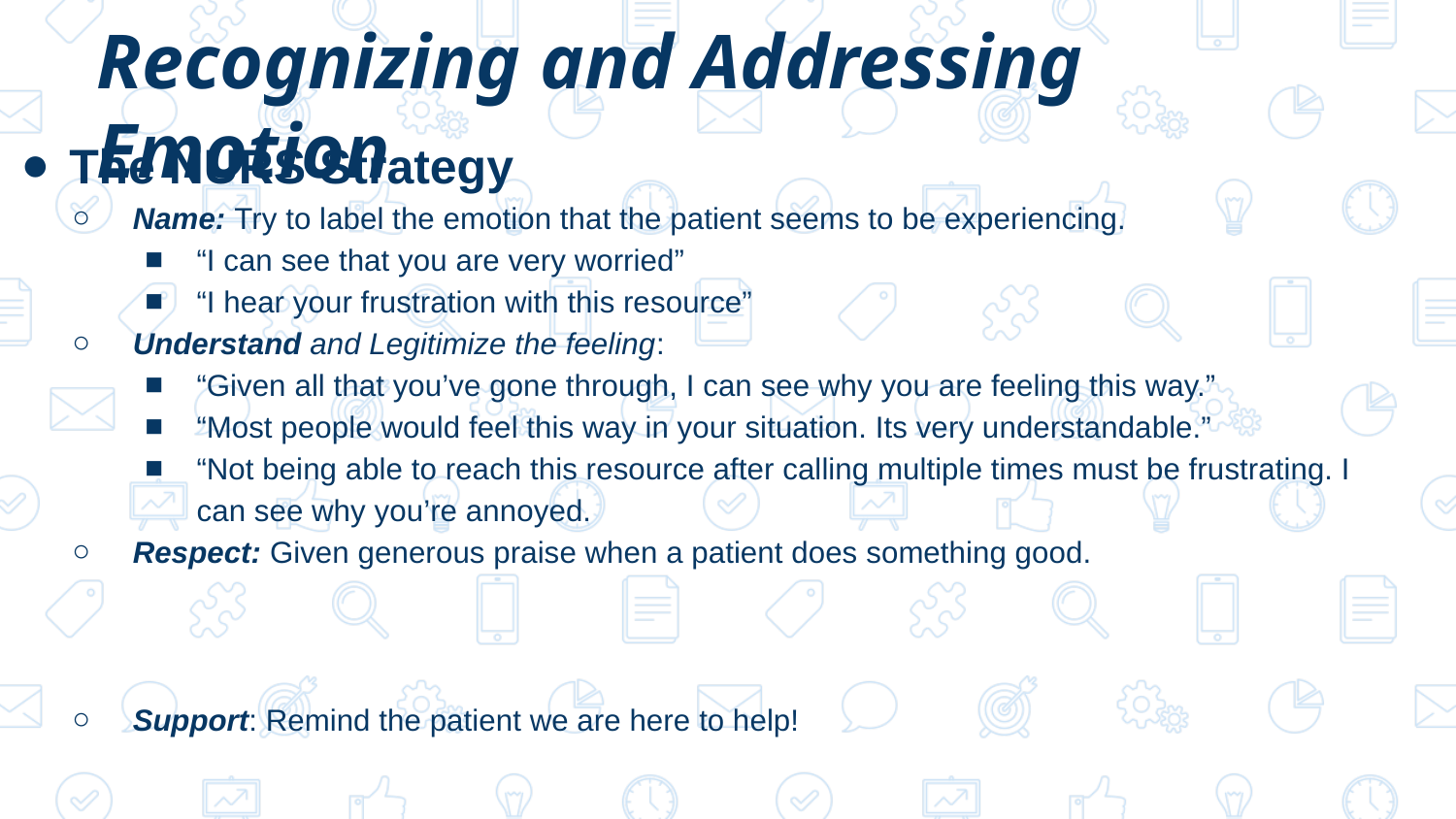

Recognizing and Addressing Emotion
The NURS Strategy
Name: Try to label the emotion that the patient seems to be experiencing.
“I can see that you are very worried”
“I hear your frustration with this resource”
Understand and Legitimize the feeling:
“Given all that you’ve gone through, I can see why you are feeling this way.”
“Most people would feel this way in your situation. Its very understandable.”
“Not being able to reach this resource after calling multiple times must be frustrating. I can see why you’re annoyed.
Respect: Given generous praise when a patient does something good.
Support: Remind the patient we are here to help!

## Slide 47
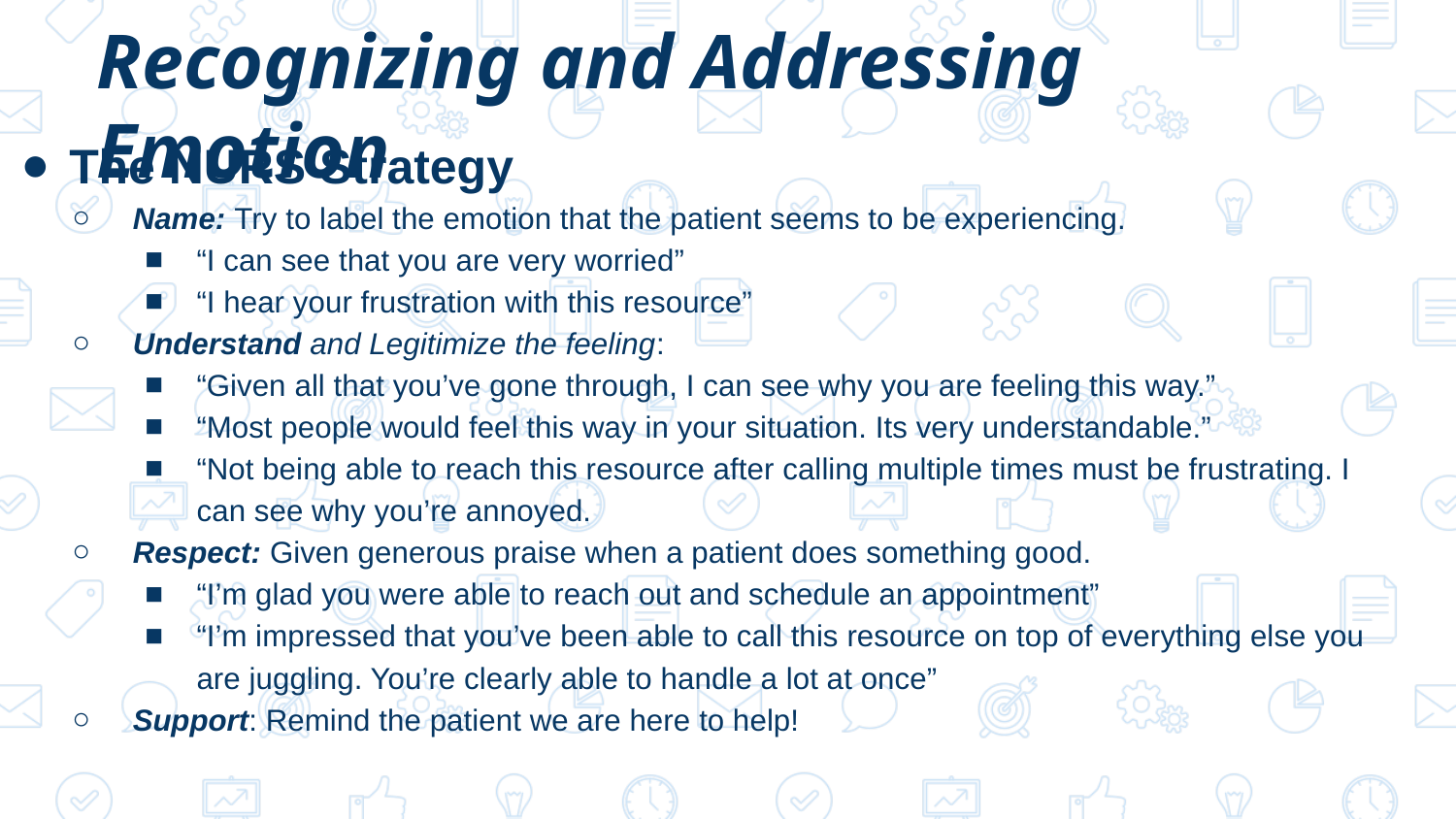

Recognizing and Addressing Emotion
The NURS Strategy
Name: Try to label the emotion that the patient seems to be experiencing.
“I can see that you are very worried”
“I hear your frustration with this resource”
Understand and Legitimize the feeling:
“Given all that you’ve gone through, I can see why you are feeling this way.”
“Most people would feel this way in your situation. Its very understandable.”
“Not being able to reach this resource after calling multiple times must be frustrating. I can see why you’re annoyed.
Respect: Given generous praise when a patient does something good.
“I’m glad you were able to reach out and schedule an appointment”
“I’m impressed that you’ve been able to call this resource on top of everything else you are juggling. You’re clearly able to handle a lot at once”
Support: Remind the patient we are here to help!

## Slide 48
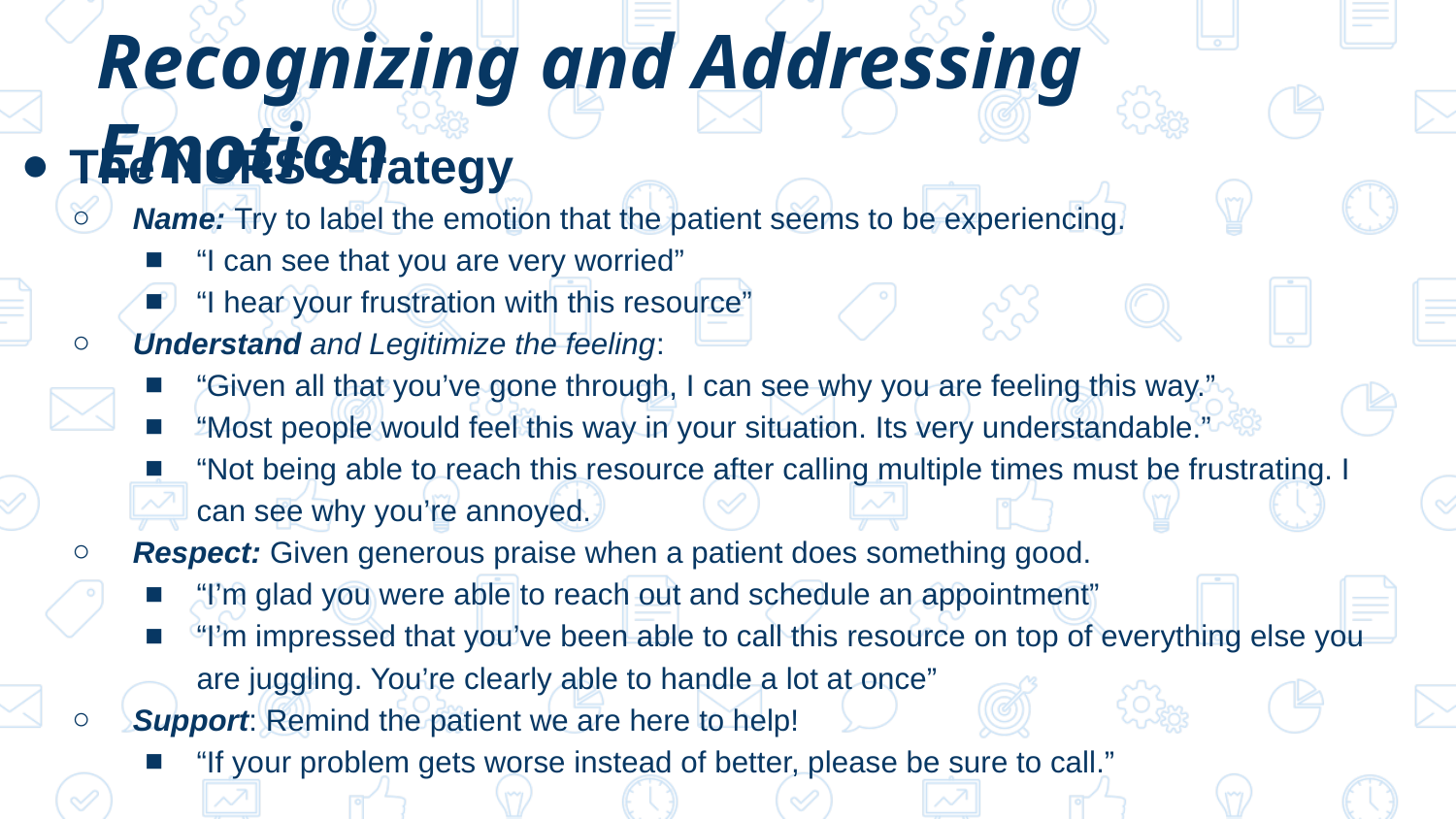

Recognizing and Addressing Emotion
The NURS Strategy
Name: Try to label the emotion that the patient seems to be experiencing.
“I can see that you are very worried”
“I hear your frustration with this resource”
Understand and Legitimize the feeling:
“Given all that you’ve gone through, I can see why you are feeling this way.”
“Most people would feel this way in your situation. Its very understandable.”
“Not being able to reach this resource after calling multiple times must be frustrating. I can see why you’re annoyed.
Respect: Given generous praise when a patient does something good.
“I’m glad you were able to reach out and schedule an appointment”
“I’m impressed that you’ve been able to call this resource on top of everything else you are juggling. You’re clearly able to handle a lot at once”
Support: Remind the patient we are here to help!
“If your problem gets worse instead of better, please be sure to call.”

## Slide 49
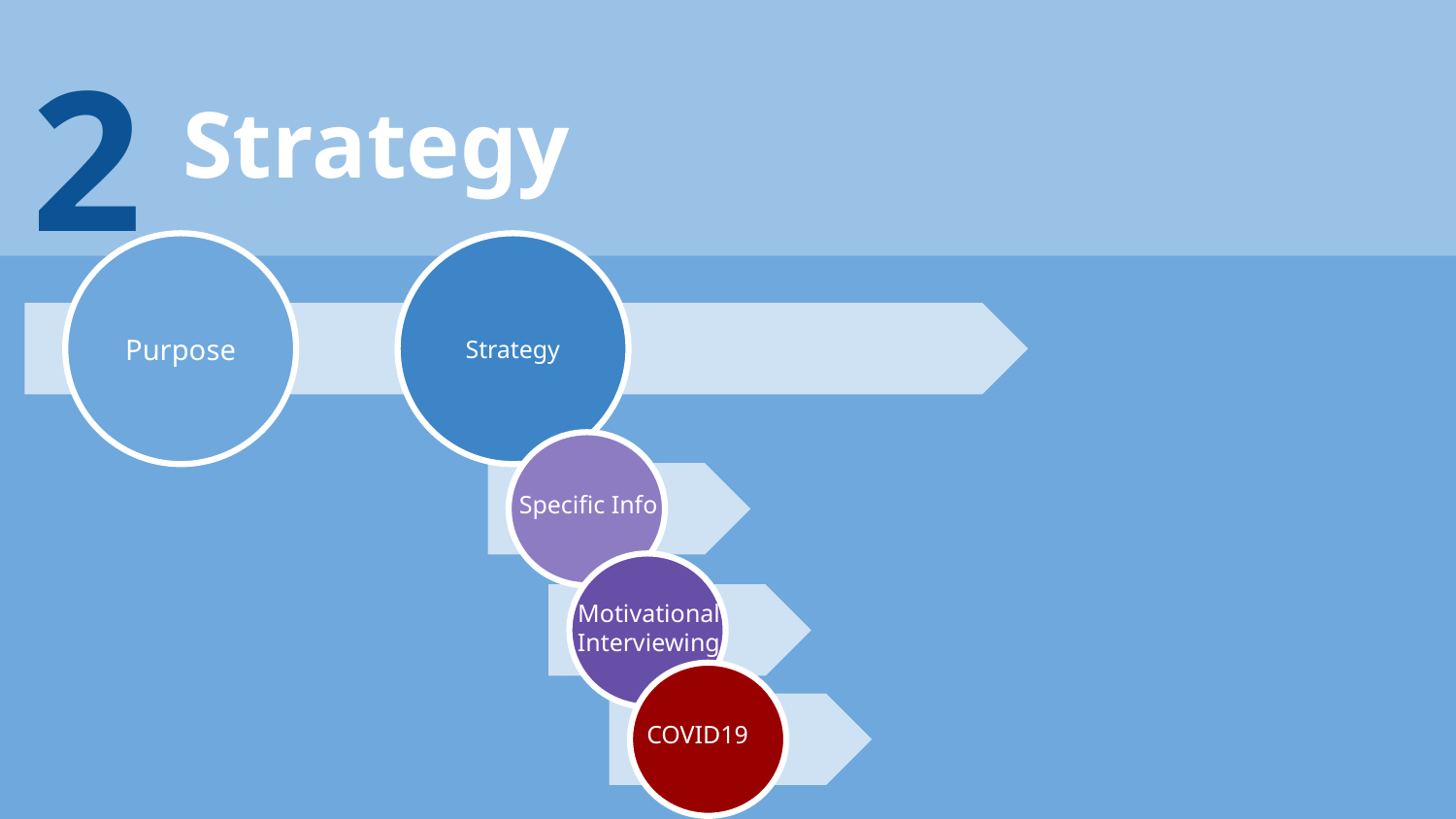

2
Strategy
Purpose
Strategy
Specific Info
Motivational
Interviewing
COVID19

## Slide 50
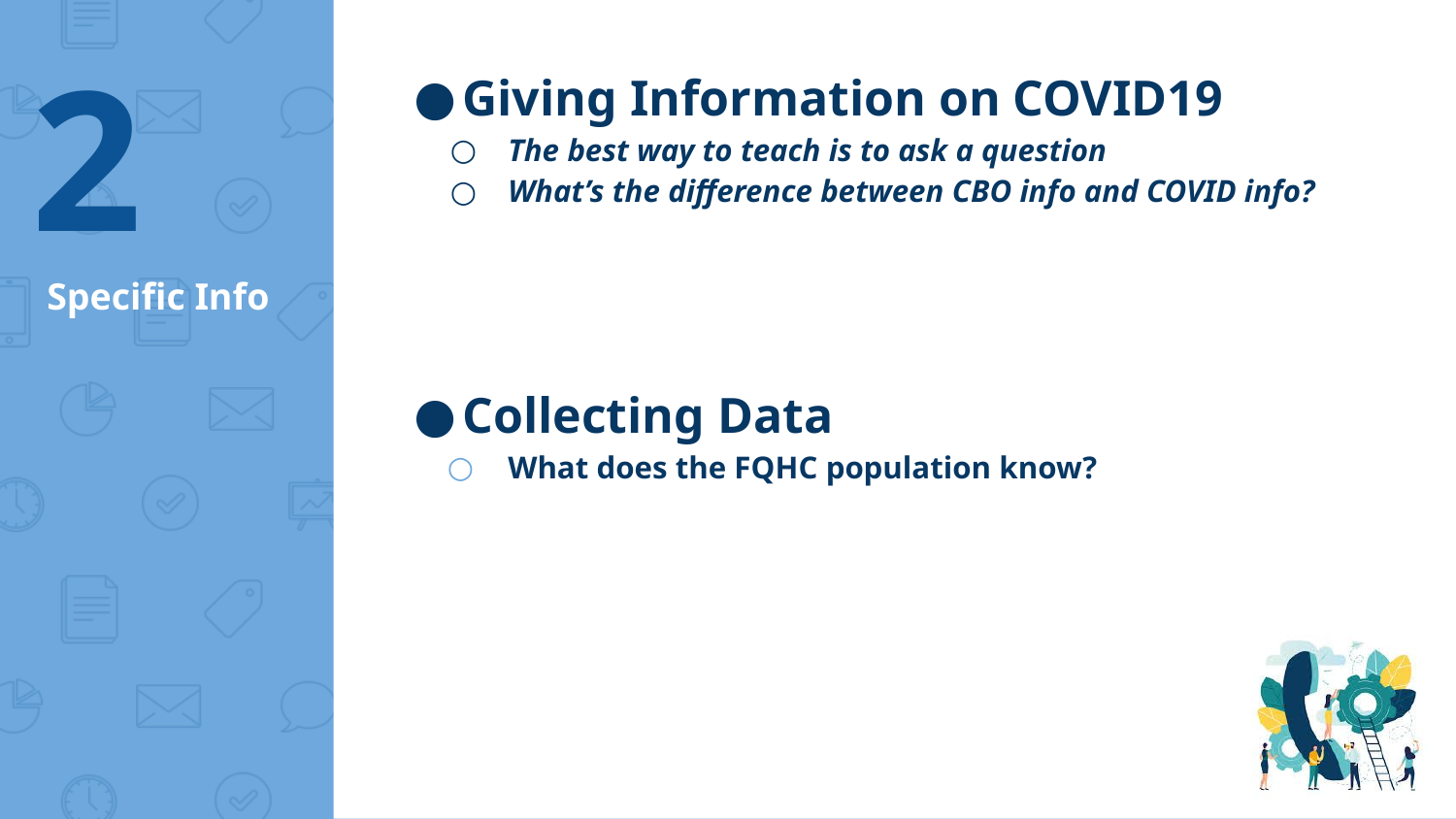

2
Giving Information on COVID19
The best way to teach is to ask a question
What’s the difference between CBO info and COVID info?
Collecting Data
What does the FQHC population know?
# Specific Info

## Slide 51
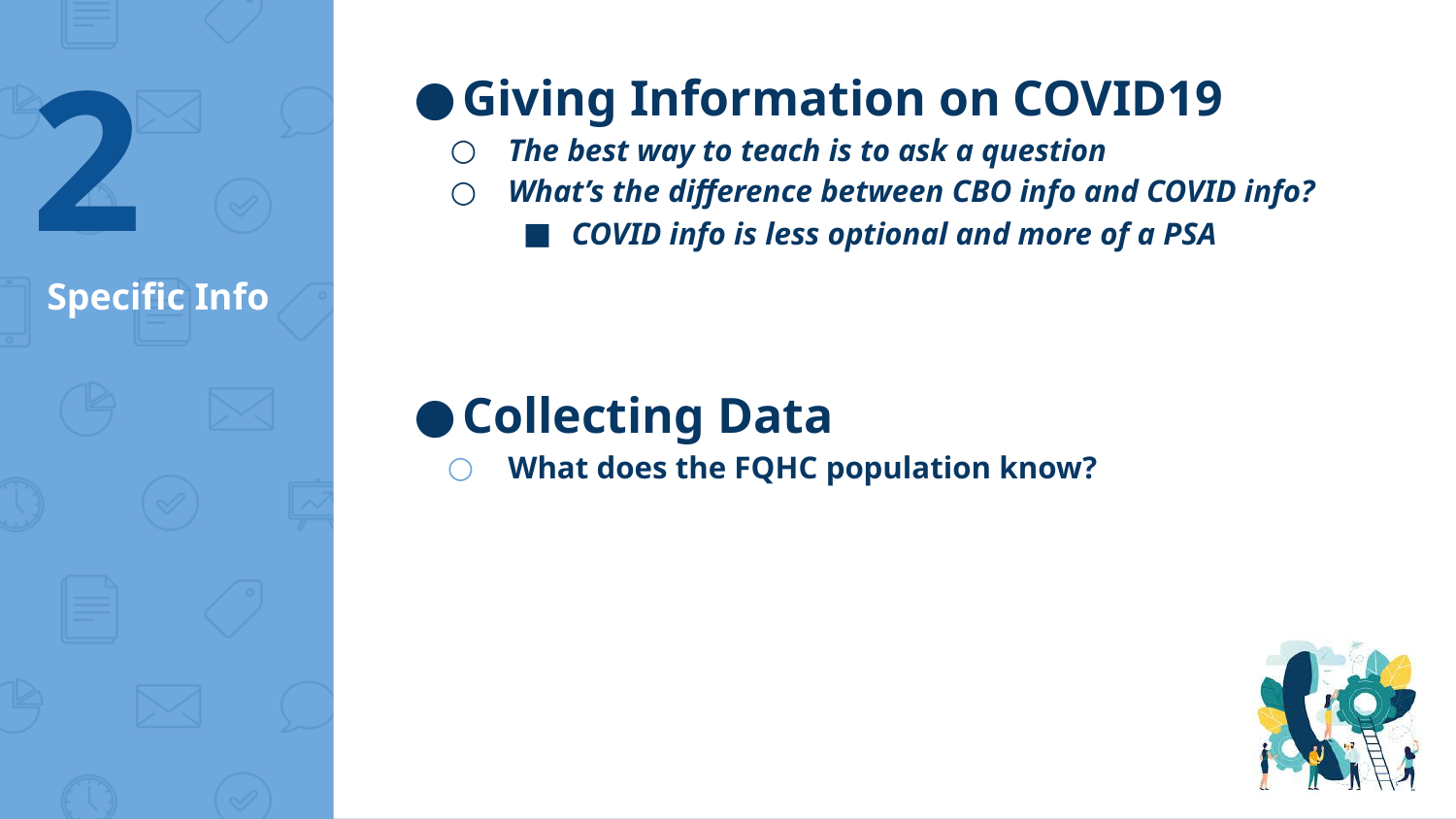

2
Giving Information on COVID19
The best way to teach is to ask a question
What’s the difference between CBO info and COVID info?
COVID info is less optional and more of a PSA
Collecting Data
What does the FQHC population know?
# Specific Info

## Slide 52
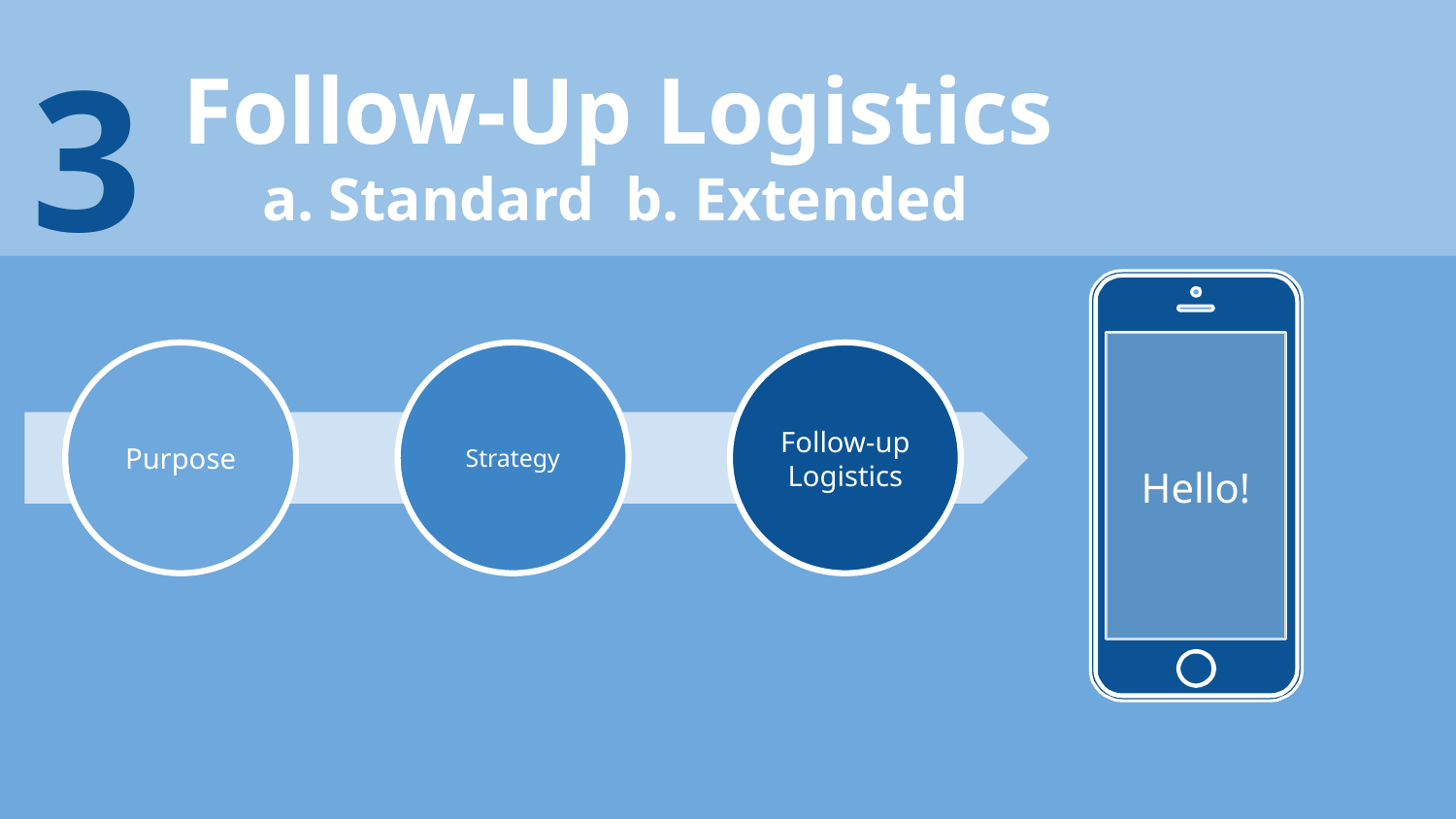

3
Follow-Up Logistics
Standard b. Extended
Hello!
Purpose
Strategy
Follow-up Logistics

## Slide 53
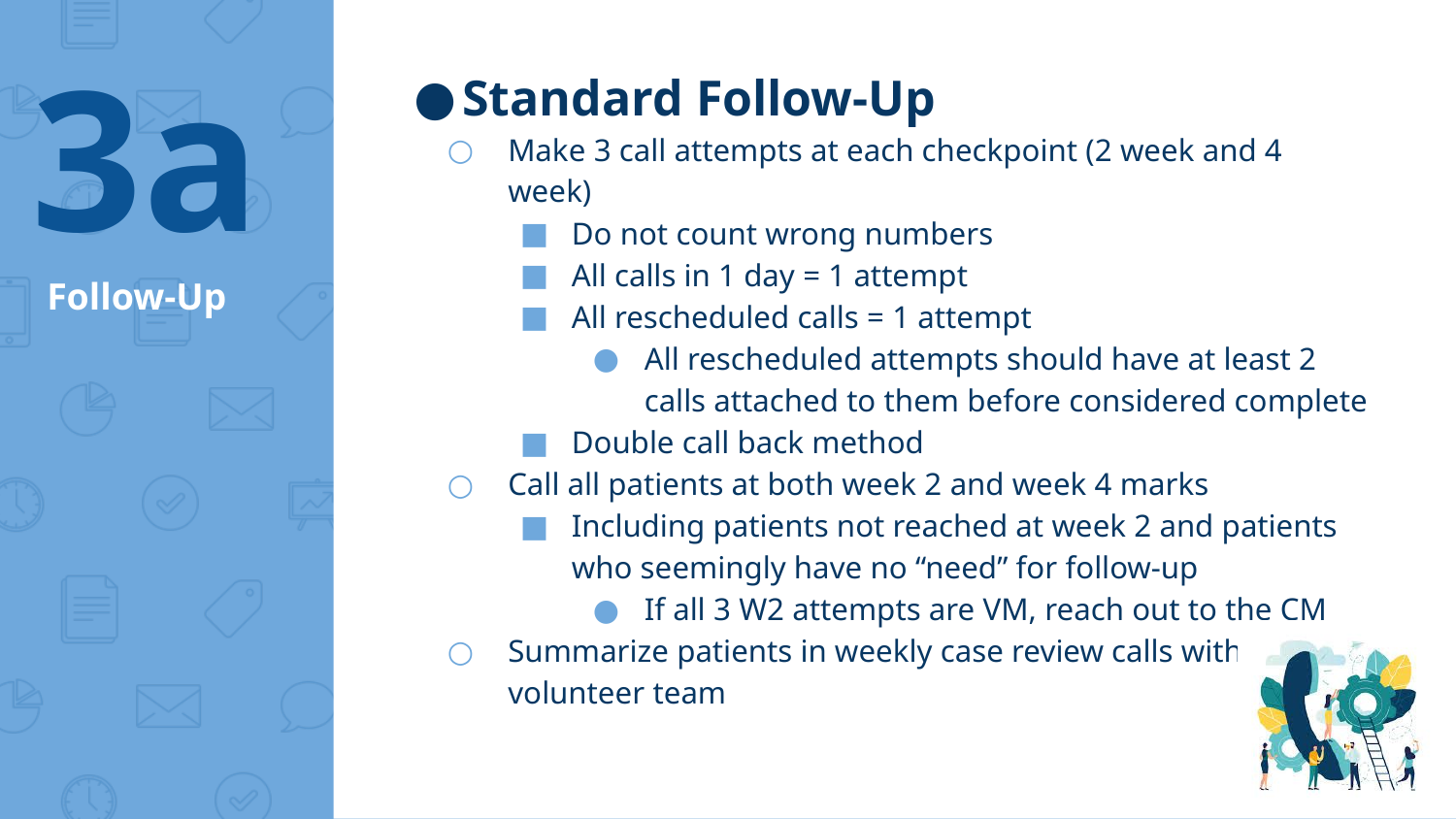

3a
Standard Follow-Up
Make 3 call attempts at each checkpoint (2 week and 4 week)
Do not count wrong numbers
All calls in 1 day = 1 attempt
All rescheduled calls = 1 attempt
All rescheduled attempts should have at least 2 calls attached to them before considered complete
Double call back method
Call all patients at both week 2 and week 4 marks
Including patients not reached at week 2 and patients who seemingly have no “need” for follow-up
If all 3 W2 attempts are VM, reach out to the CM
Summarize patients in weekly case review calls with the volunteer team
# Follow-Up

## Slide 54
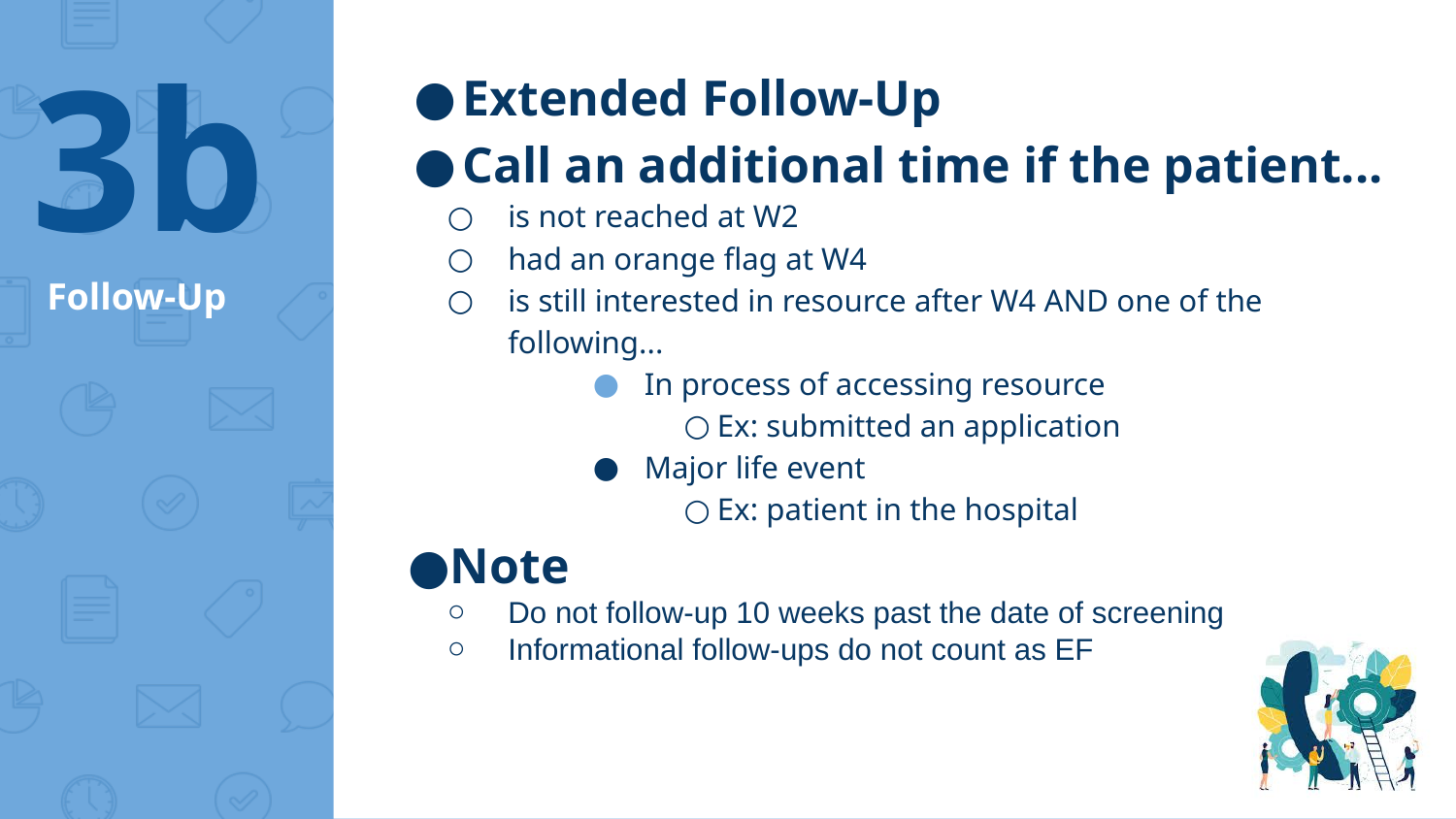

3b
Extended Follow-Up
Call an additional time if the patient...
is not reached at W2
had an orange flag at W4
is still interested in resource after W4 AND one of the following...
In process of accessing resource
Ex: submitted an application
Major life event
Ex: patient in the hospital
Note
Do not follow-up 10 weeks past the date of screening
Informational follow-ups do not count as EF
# Follow-Up

## Slide 55
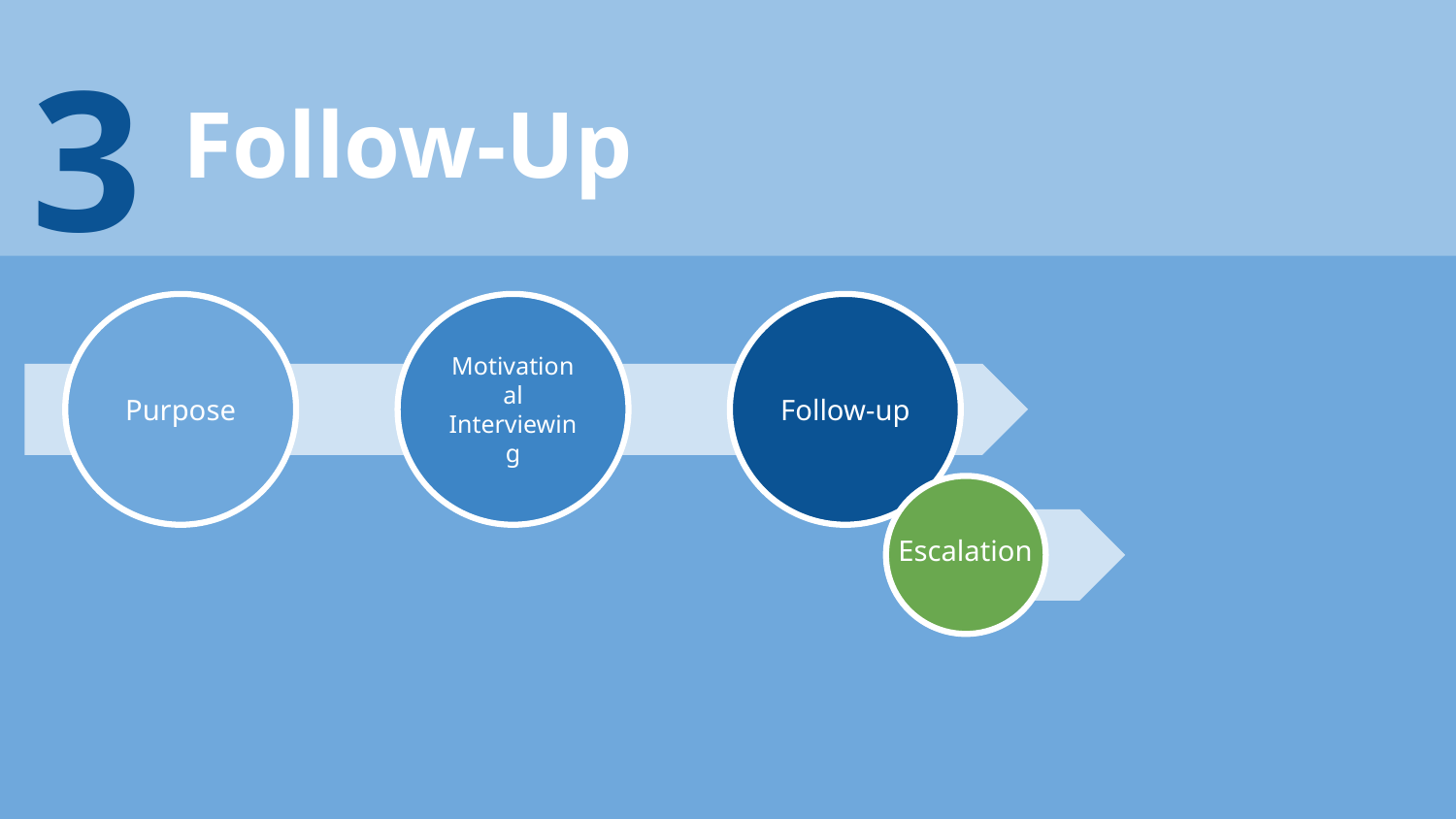

3
Follow-Up
Purpose
Motivational Interviewing
Follow-up
Escalation

## Slide 56
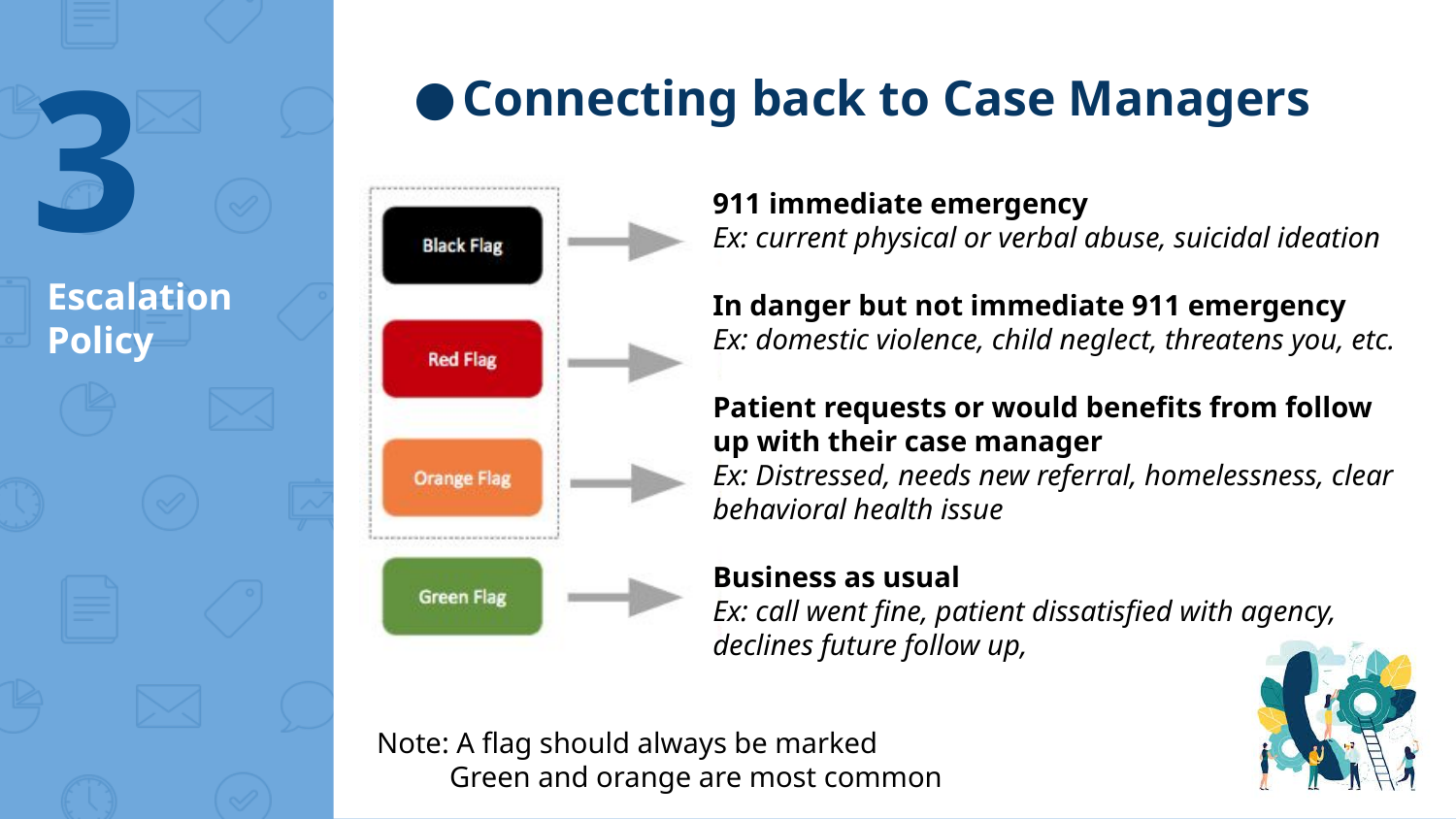

3
Connecting back to Case Managers
911 immediate emergency
Ex: current physical or verbal abuse, suicidal ideation
In danger but not immediate 911 emergency
Ex: domestic violence, child neglect, threatens you, etc.
Patient requests or would benefits from follow up with their case manager
Ex: Distressed, needs new referral, homelessness, clear behavioral health issue
Business as usual
Ex: call went fine, patient dissatisfied with agency,
declines future follow up,
# Escalation Policy
Note: A flag should always be marked
Green and orange are most common

## Slide 57
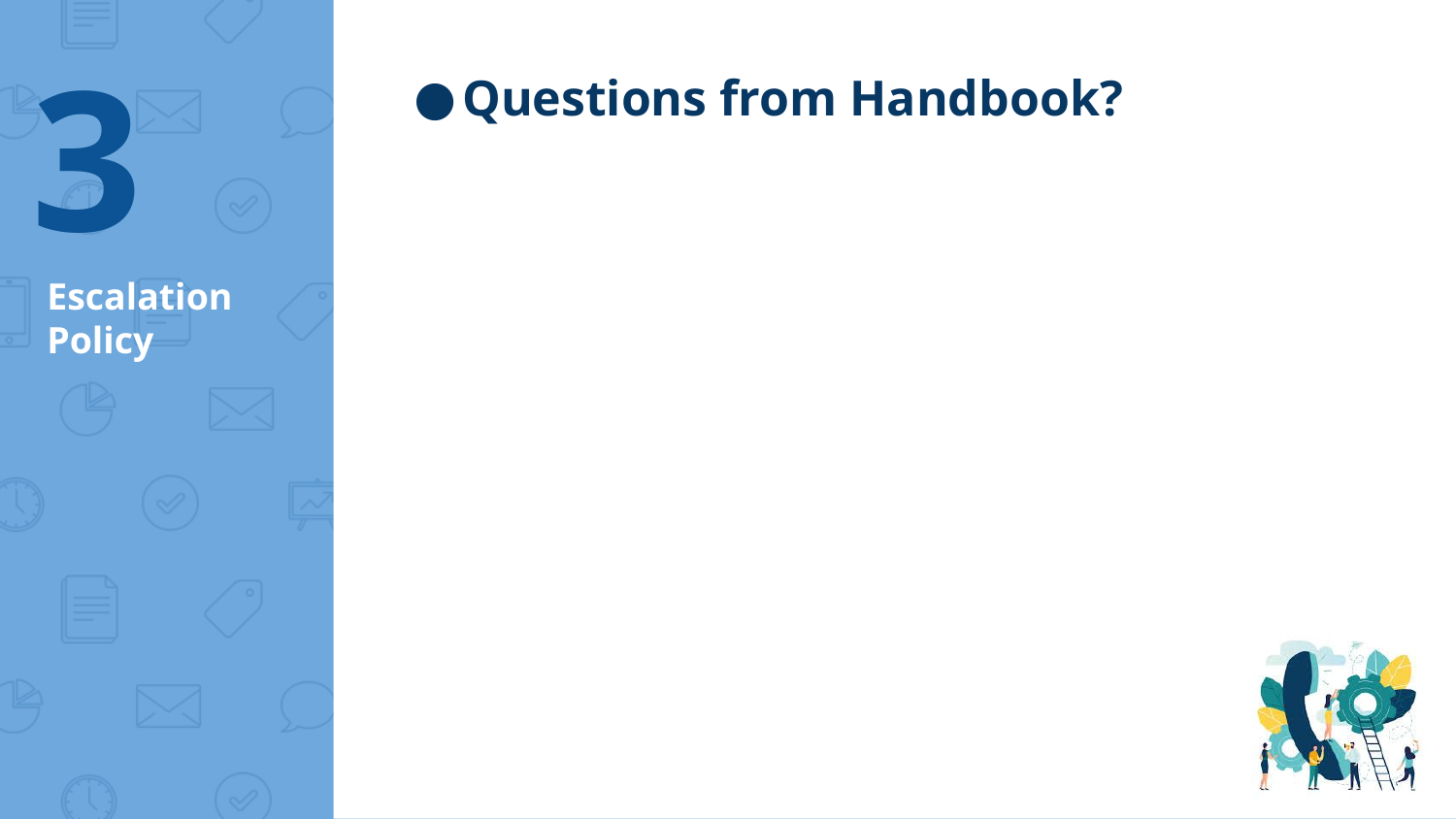

3
Questions from Handbook?
# Escalation Policy

## Slide 58
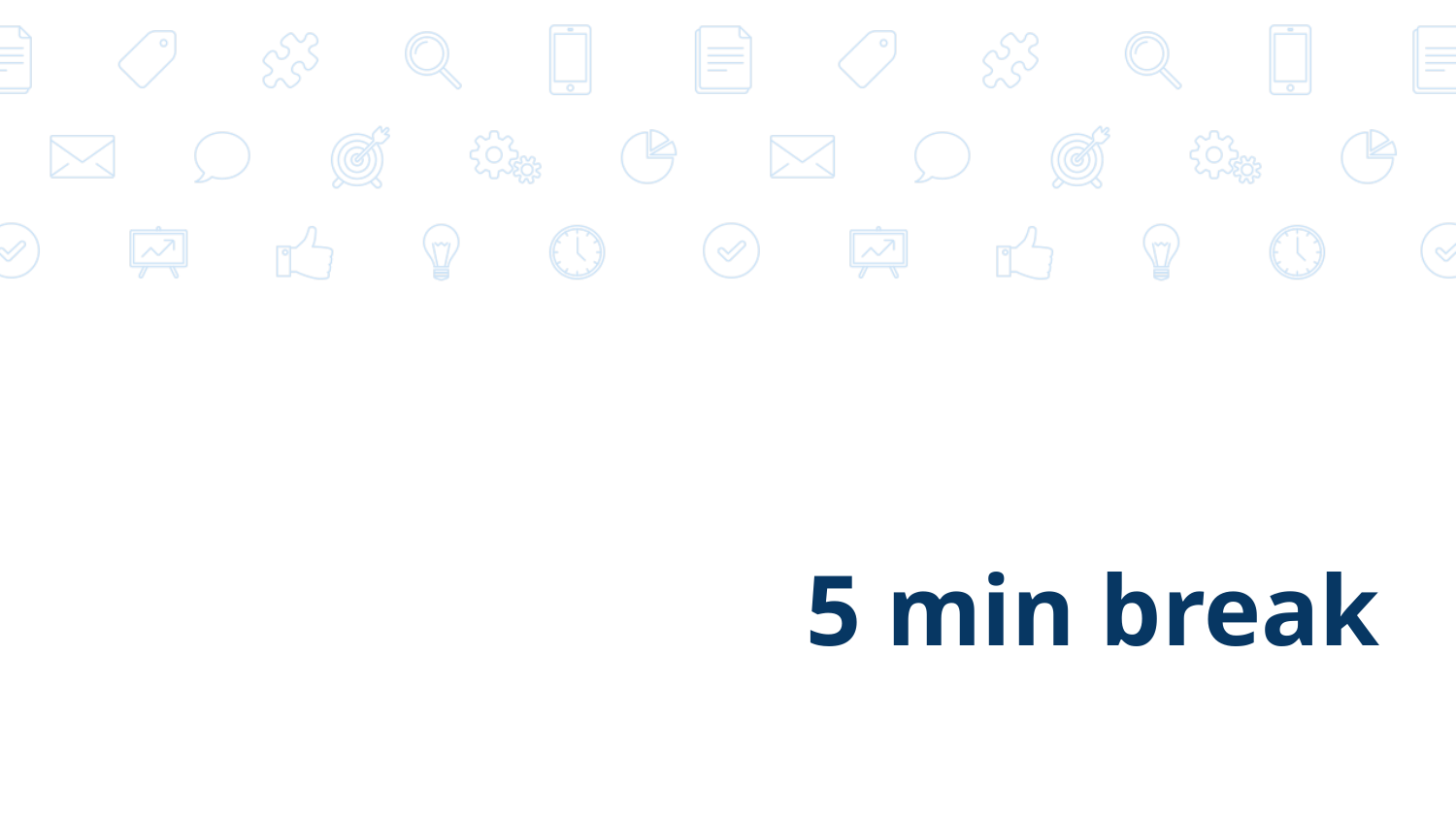

# 5 min break

## Slide 59
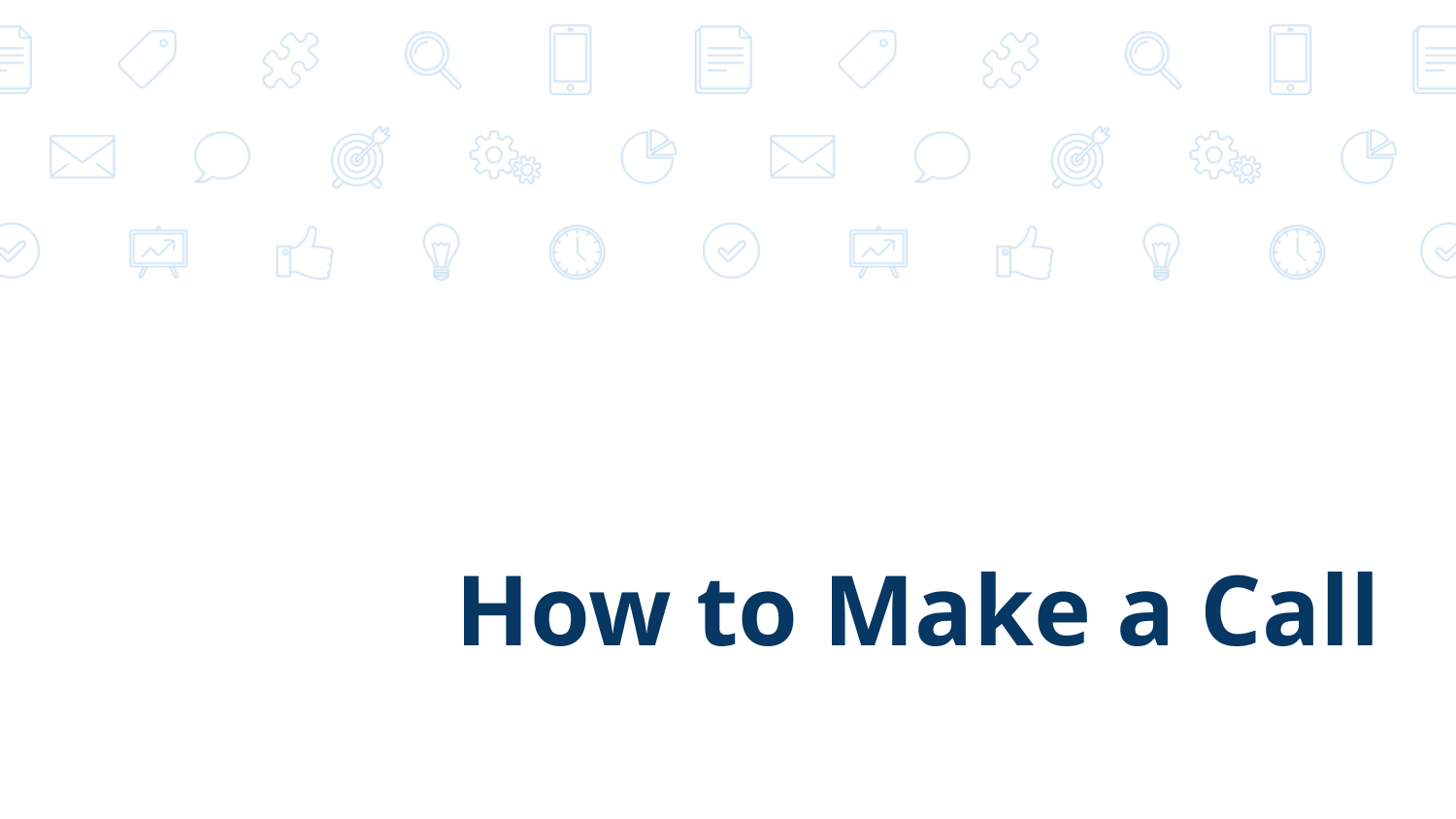

# How to Make a Call

## Slide 60
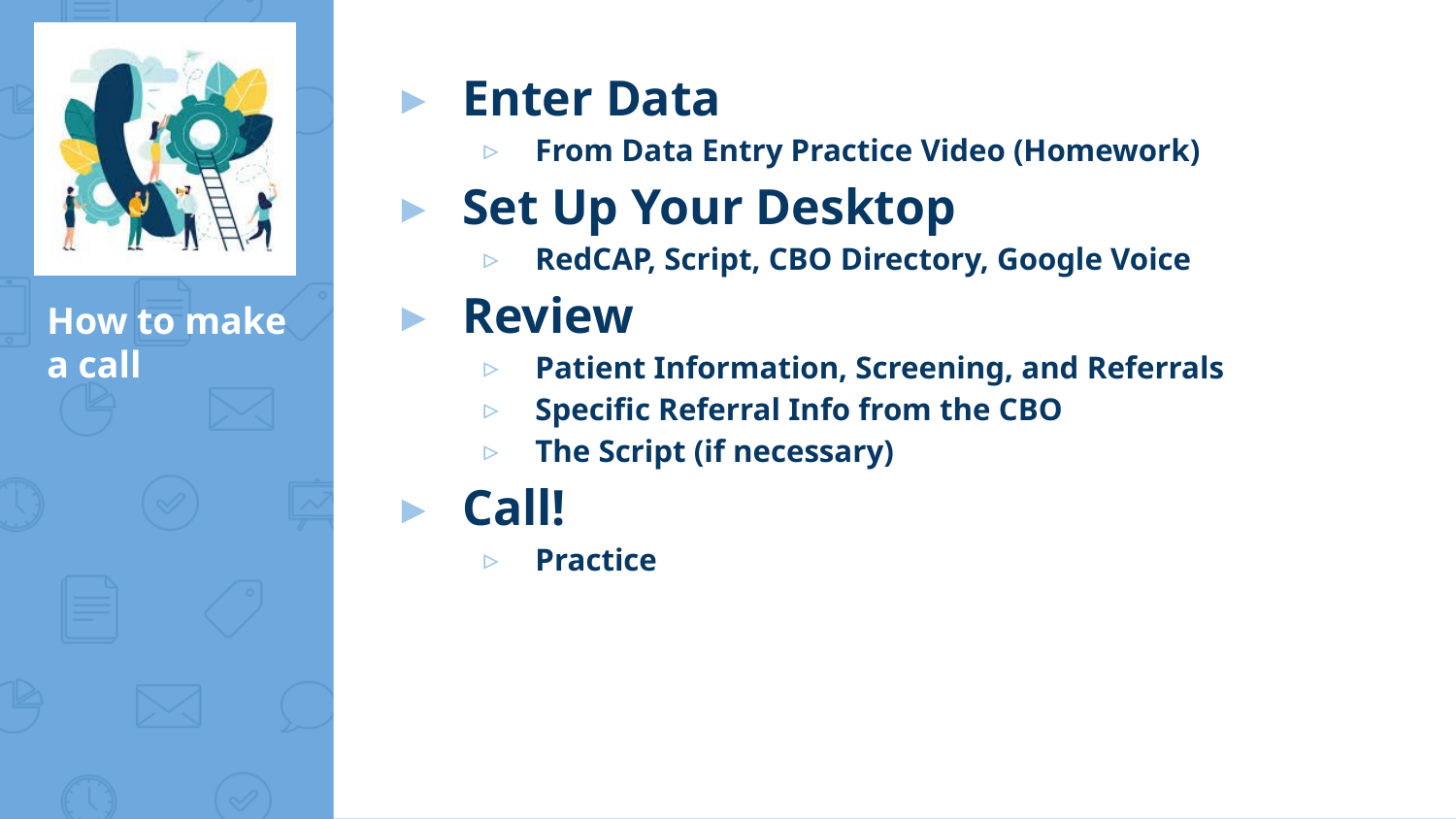

Enter Data
From Data Entry Practice Video (Homework)
Set Up Your Desktop
RedCAP, Script, CBO Directory, Google Voice
Review
Patient Information, Screening, and Referrals
Specific Referral Info from the CBO
The Script (if necessary)
Call!
Practice
# How to make a call

## Slide 61
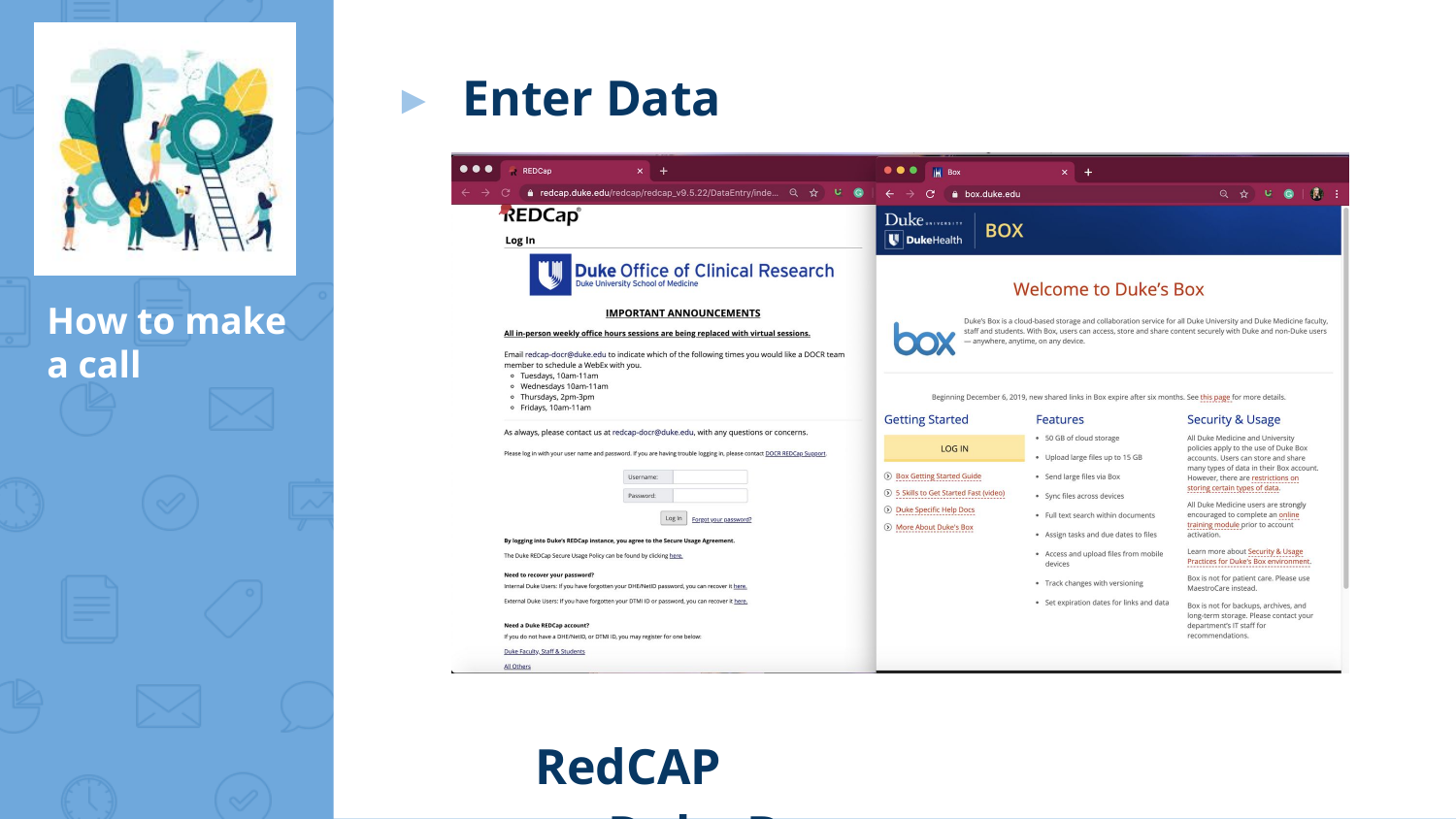

Enter Data
RedCAP					Duke Box
# How to make a call

## Slide 62
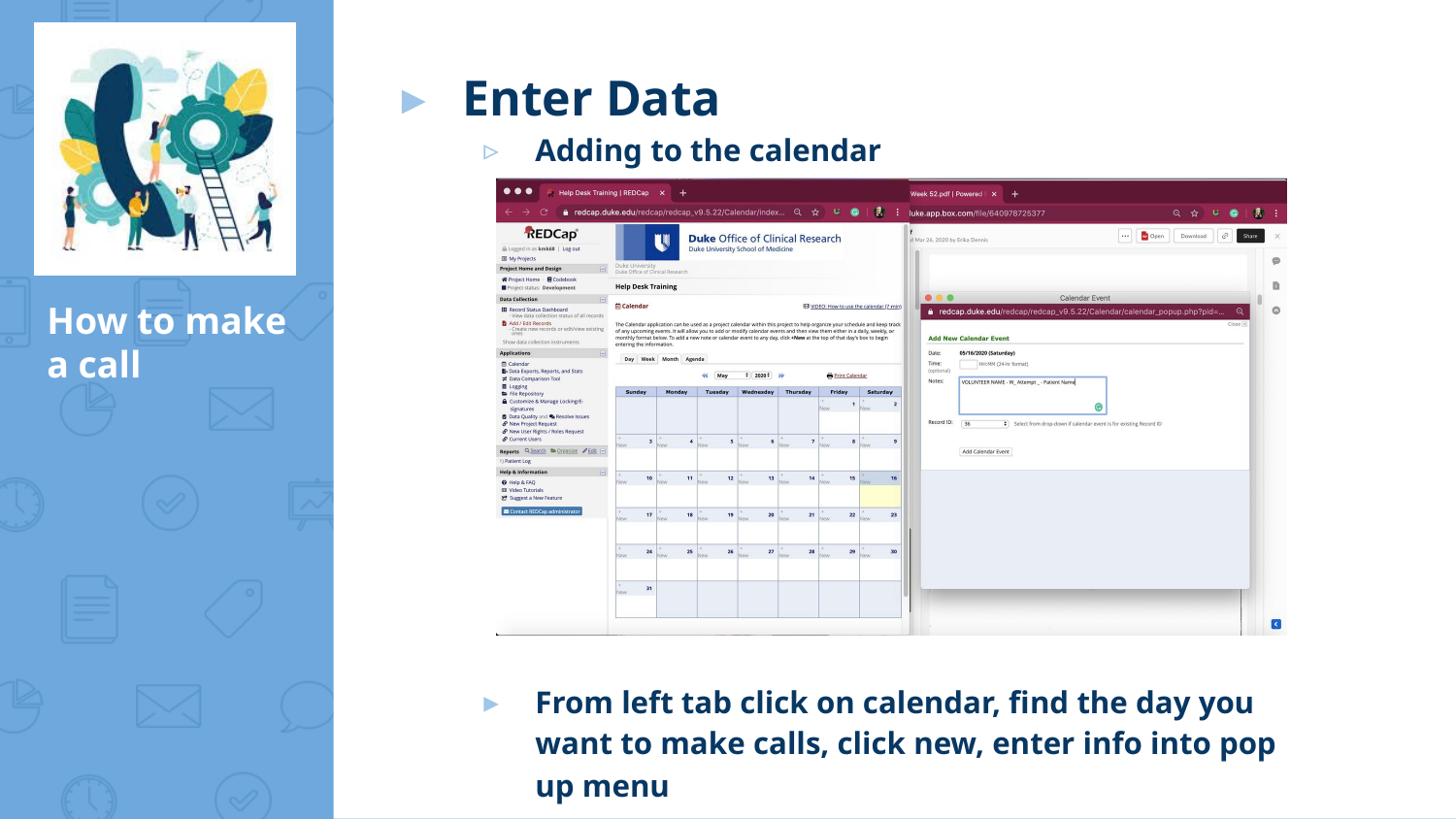

Enter Data
Adding to the calendar
From left tab click on calendar, find the day you want to make calls, click new, enter info into pop up menu
# How to make a call

## Slide 63
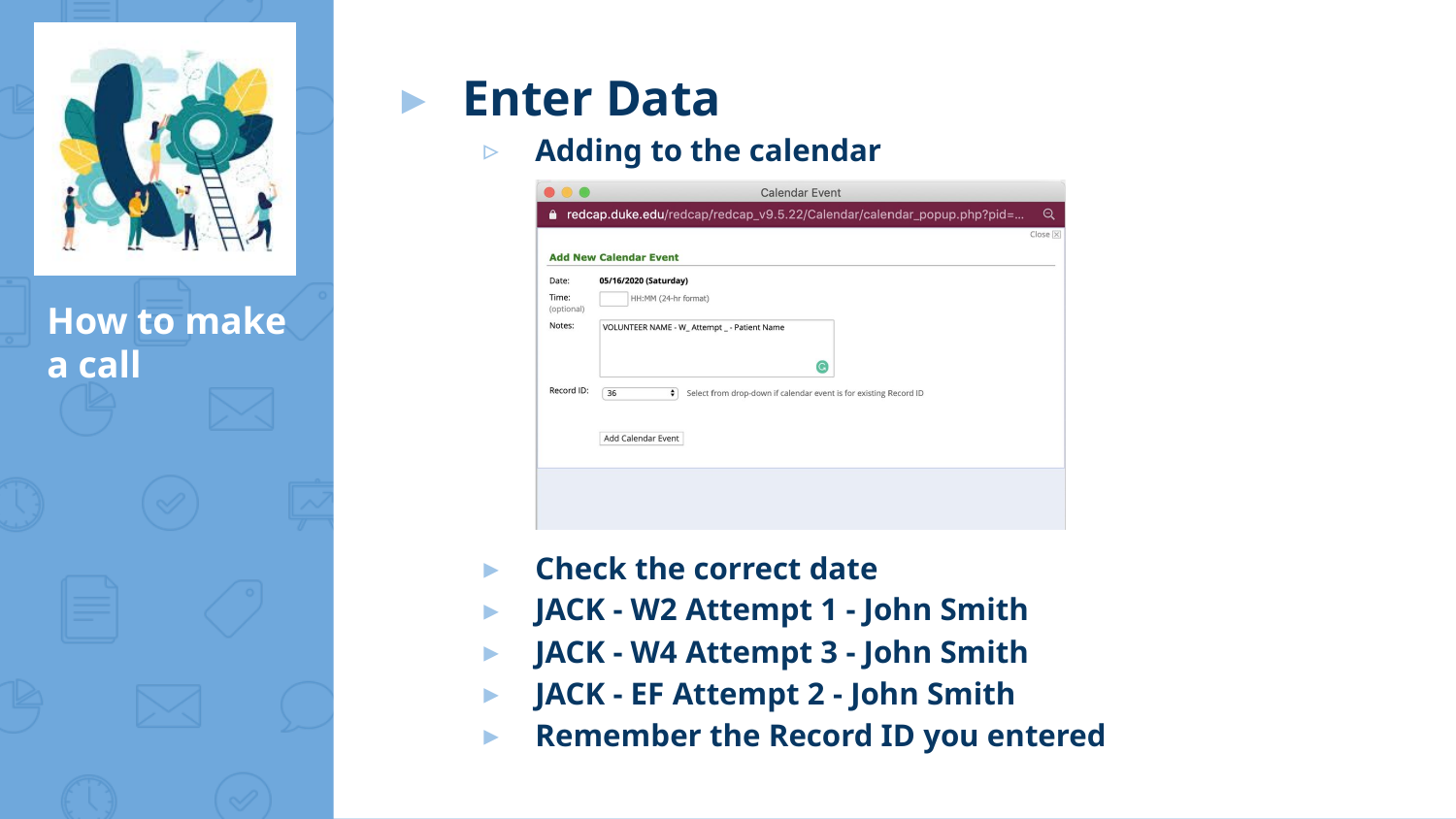

Enter Data
Adding to the calendar
Check the correct date
JACK - W2 Attempt 1 - John Smith
JACK - W4 Attempt 3 - John Smith
JACK - EF Attempt 2 - John Smith
Remember the Record ID you entered
# How to make a call

## Slide 64
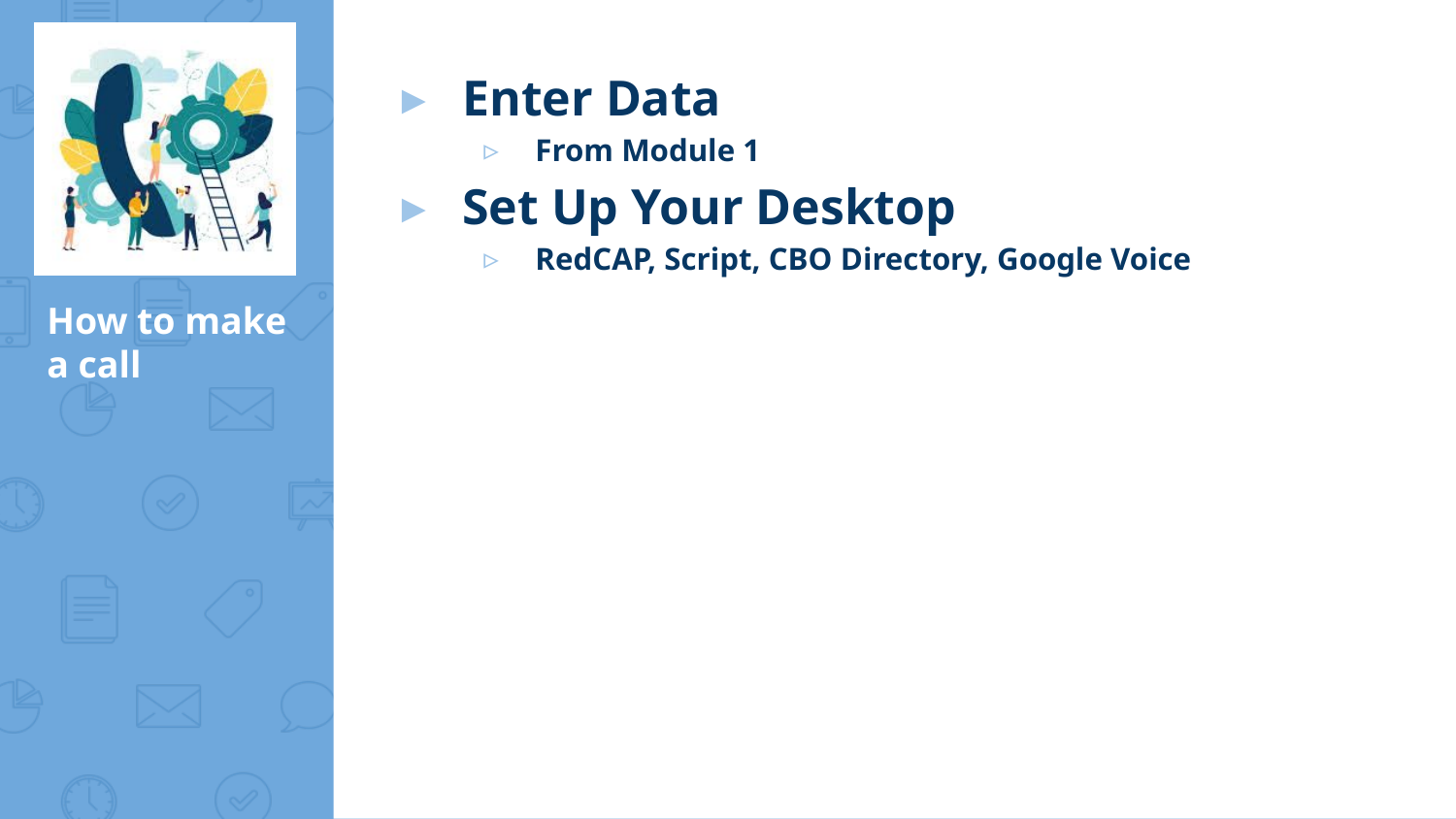

Enter Data
From Module 1
Set Up Your Desktop
RedCAP, Script, CBO Directory, Google Voice
# How to make a call

## Slide 65
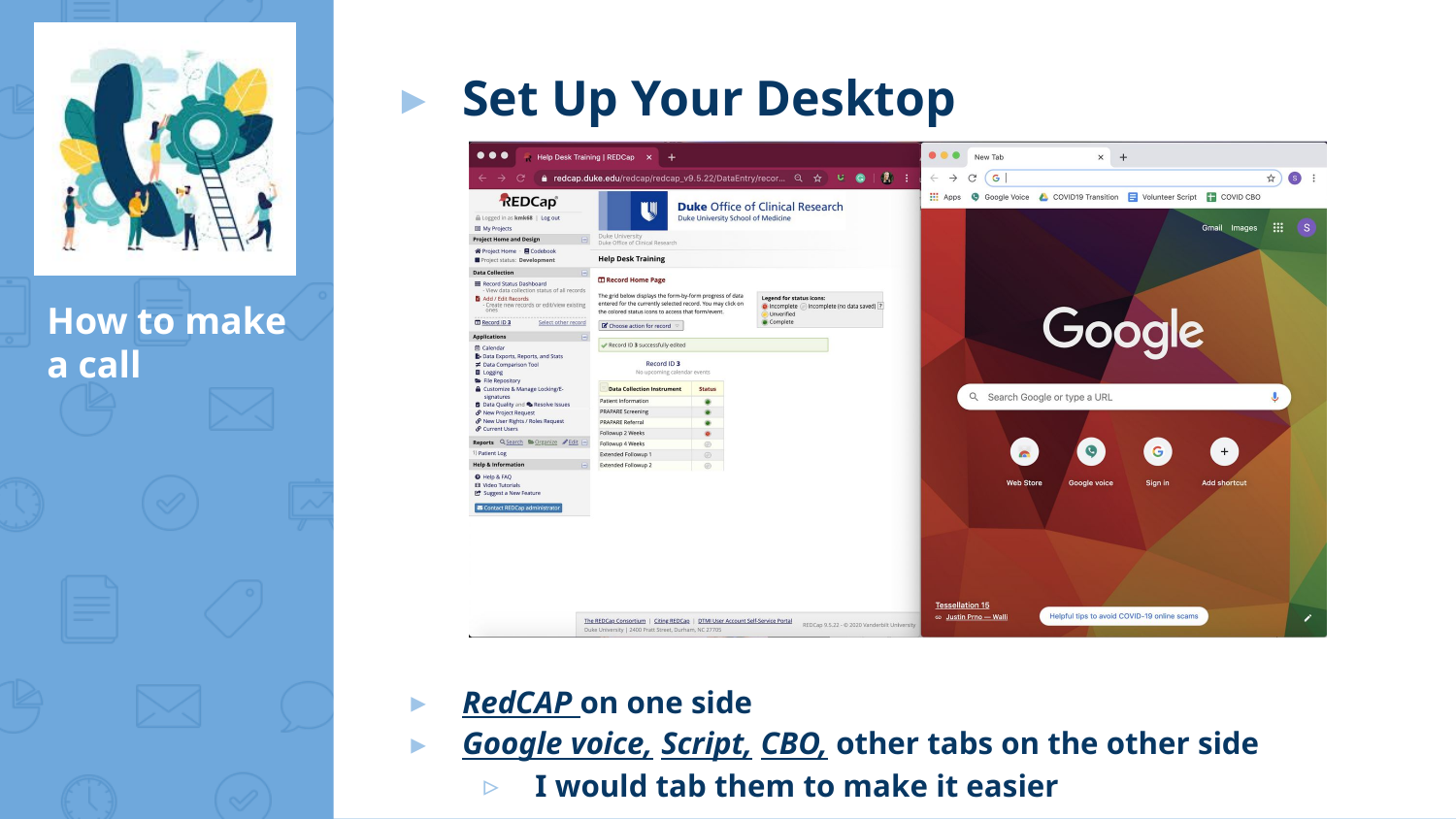

Set Up Your Desktop
RedCAP on one side
Google voice, Script, CBO, other tabs on the other side
I would tab them to make it easier
# How to make a call

## Slide 66
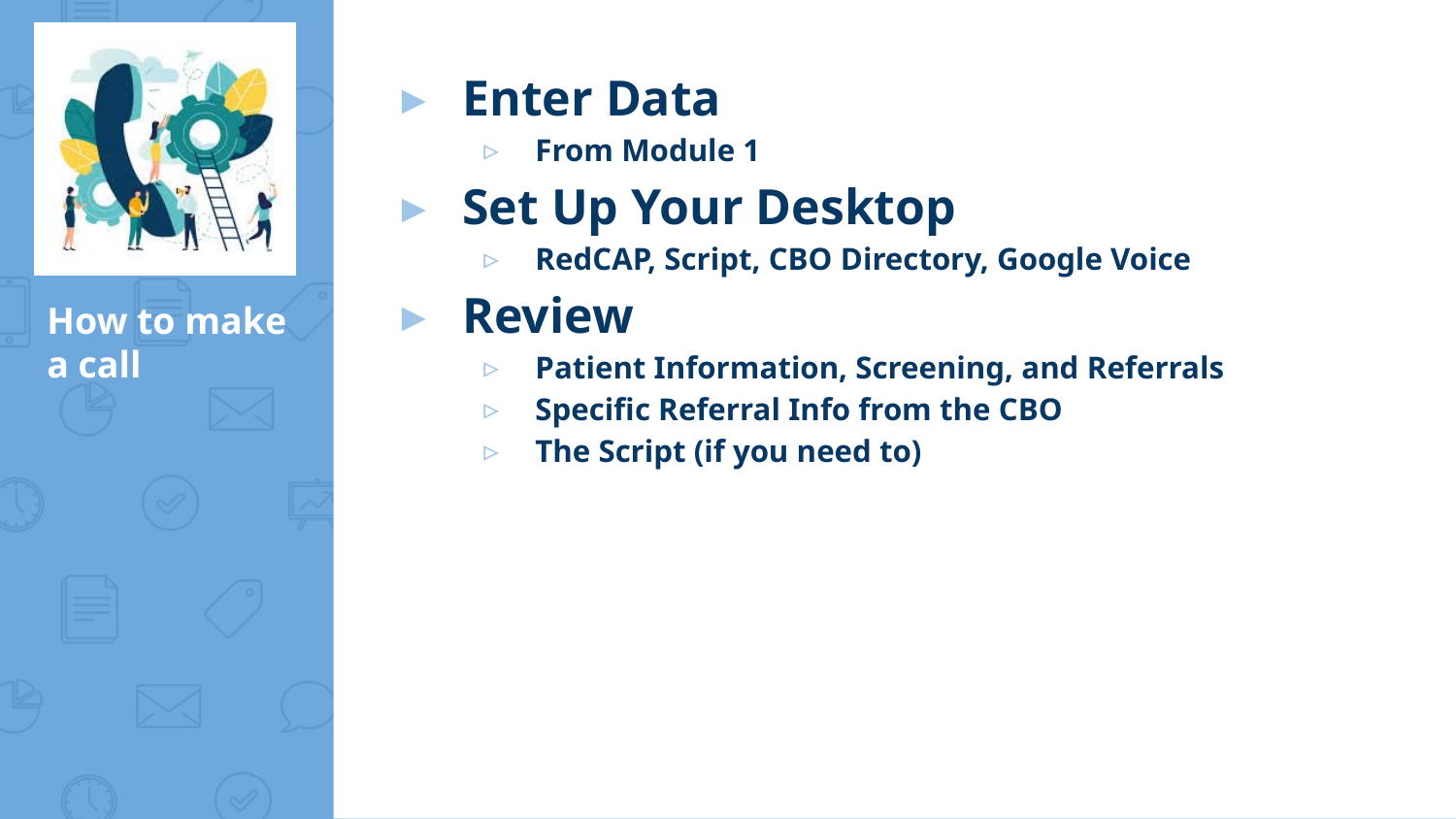

Enter Data
From Module 1
Set Up Your Desktop
RedCAP, Script, CBO Directory, Google Voice
Review
Patient Information, Screening, and Referrals
Specific Referral Info from the CBO
The Script (if you need to)
# How to make a call

## Slide 67
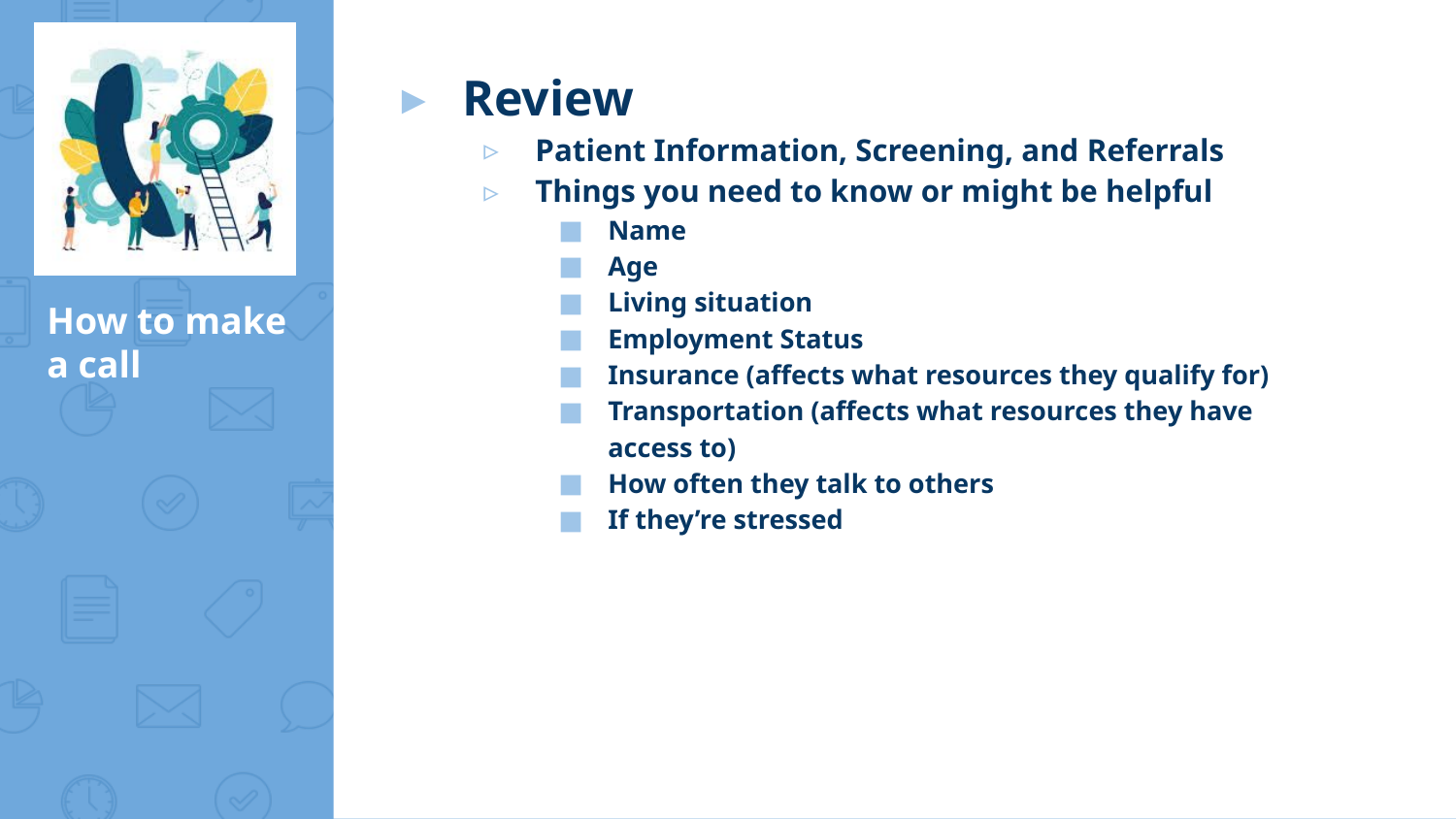

Review
Patient Information, Screening, and Referrals
Things you need to know or might be helpful
Name
Age
Living situation
Employment Status
Insurance (affects what resources they qualify for)
Transportation (affects what resources they have access to)
How often they talk to others
If they’re stressed
# How to make a call

## Slide 68
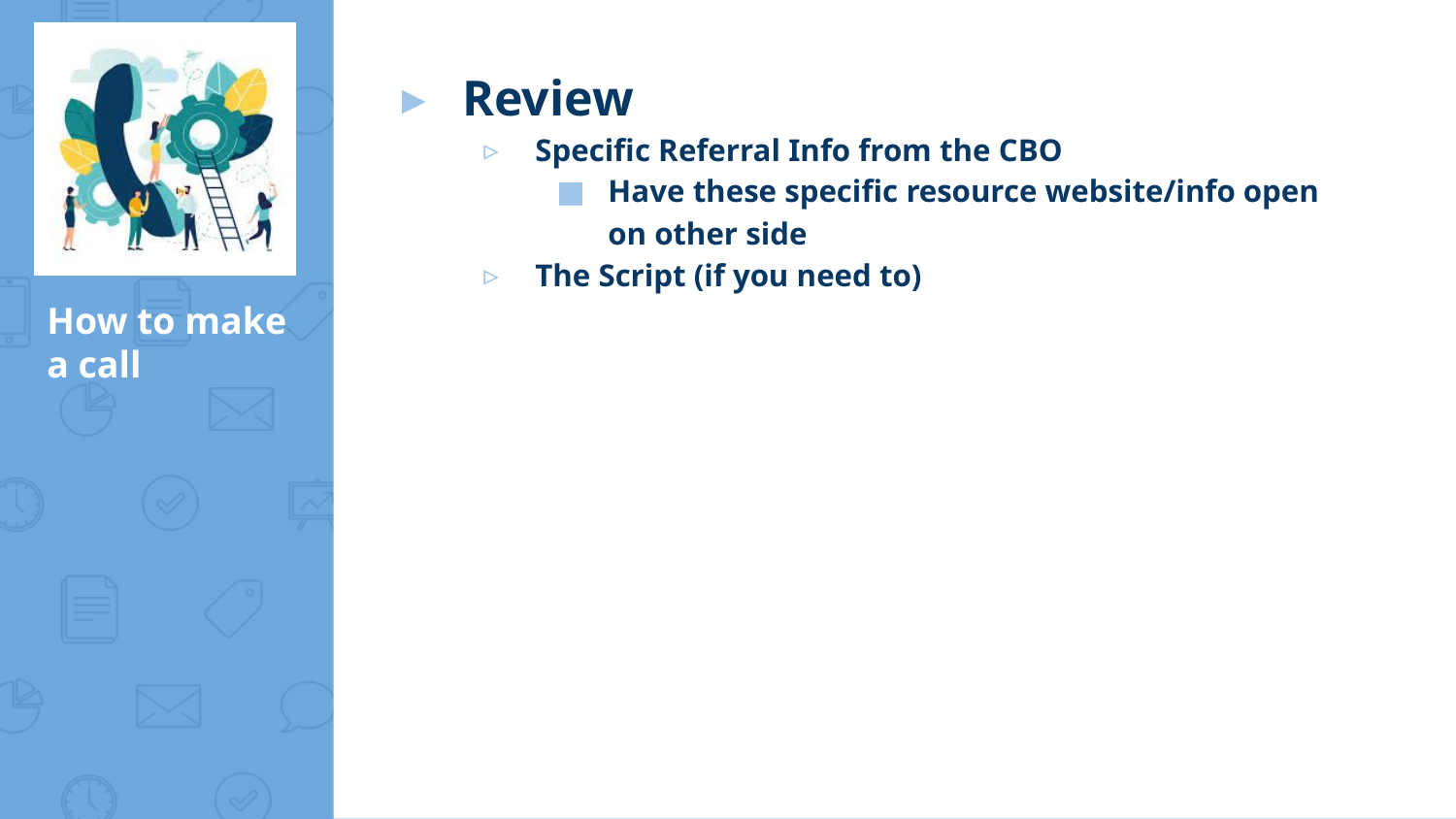

Review
Specific Referral Info from the CBO
Have these specific resource website/info open on other side
The Script (if you need to)
# How to make a call

## Slide 69
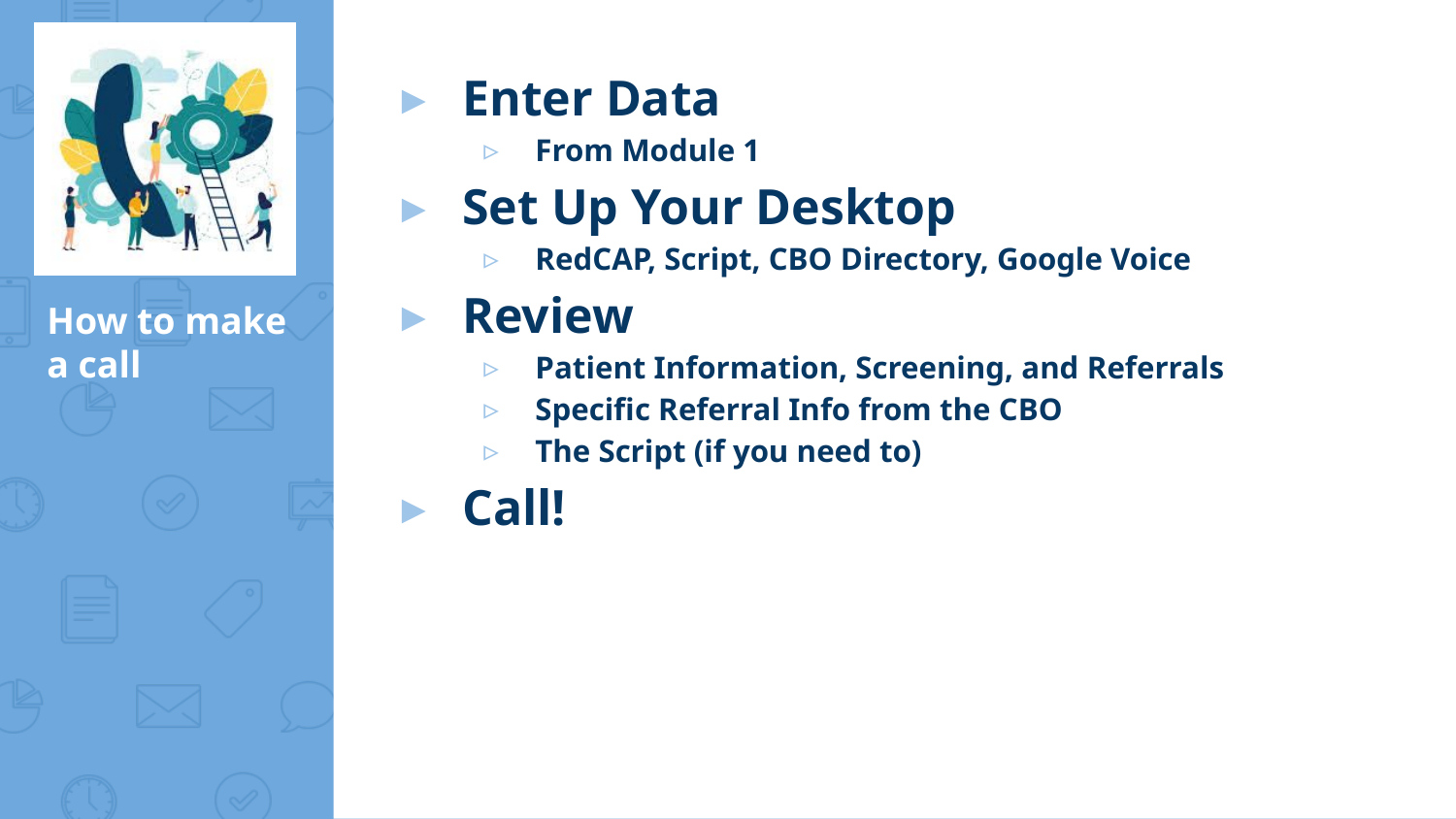

Enter Data
From Module 1
Set Up Your Desktop
RedCAP, Script, CBO Directory, Google Voice
Review
Patient Information, Screening, and Referrals
Specific Referral Info from the CBO
The Script (if you need to)
Call!
# How to make a call

## Slide 70
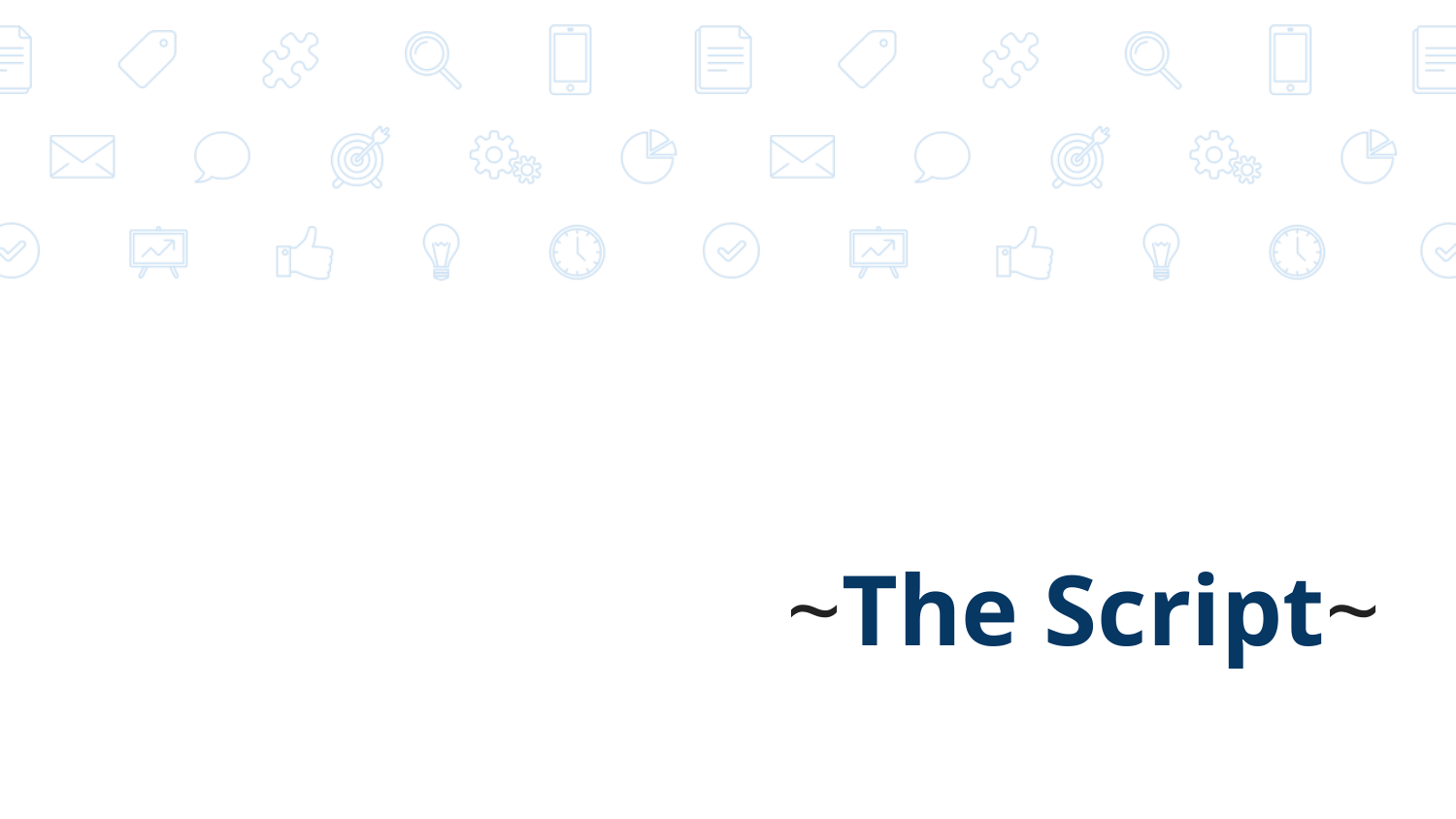

# ~The Script~

## Slide 71
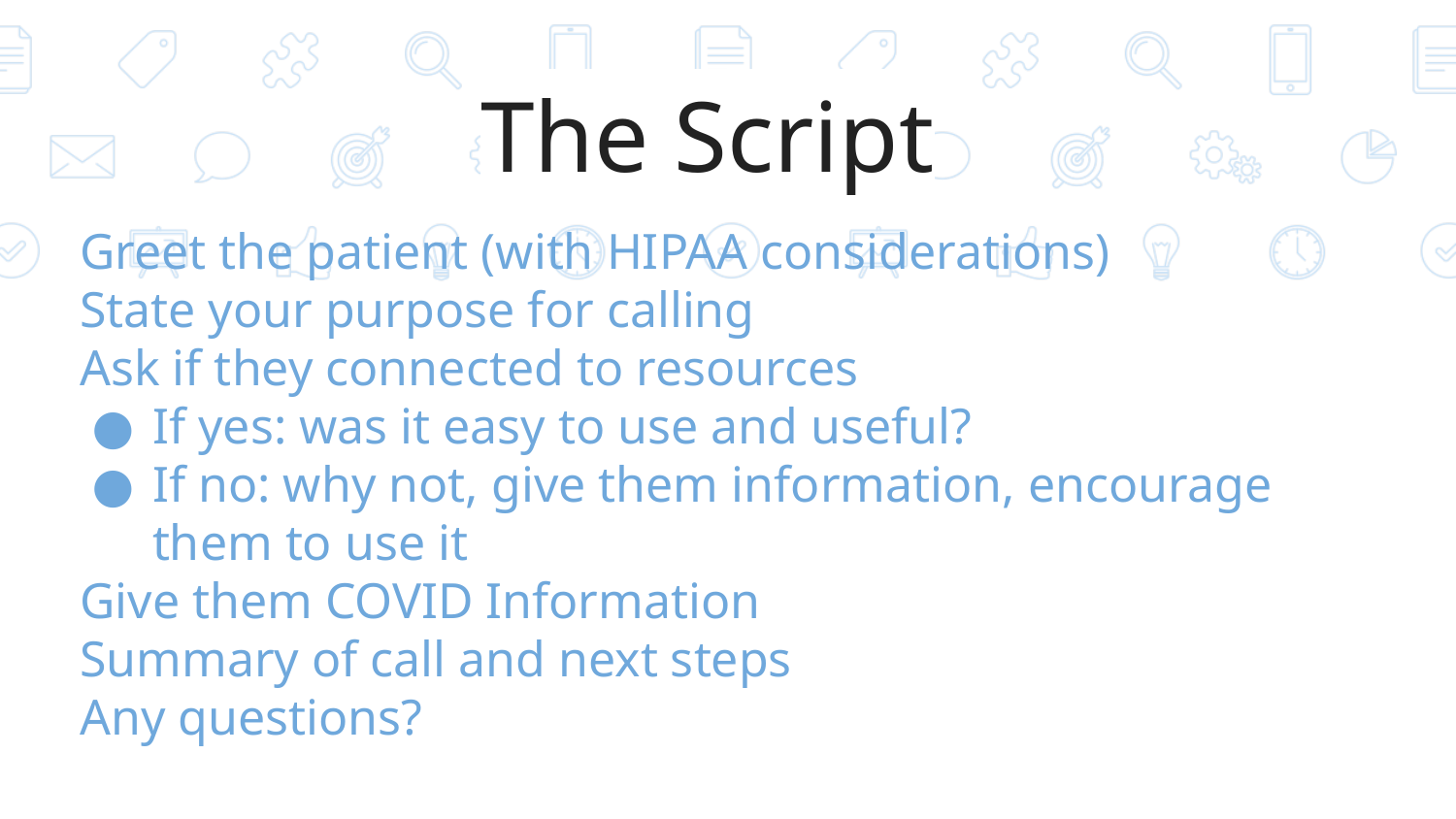

# The Script
Greet the patient (with HIPAA considerations)
State your purpose for calling
Ask if they connected to resources
If yes: was it easy to use and useful?
If no: why not, give them information, encourage them to use it
Give them COVID Information
Summary of call and next steps
Any questions?

## Slide 72
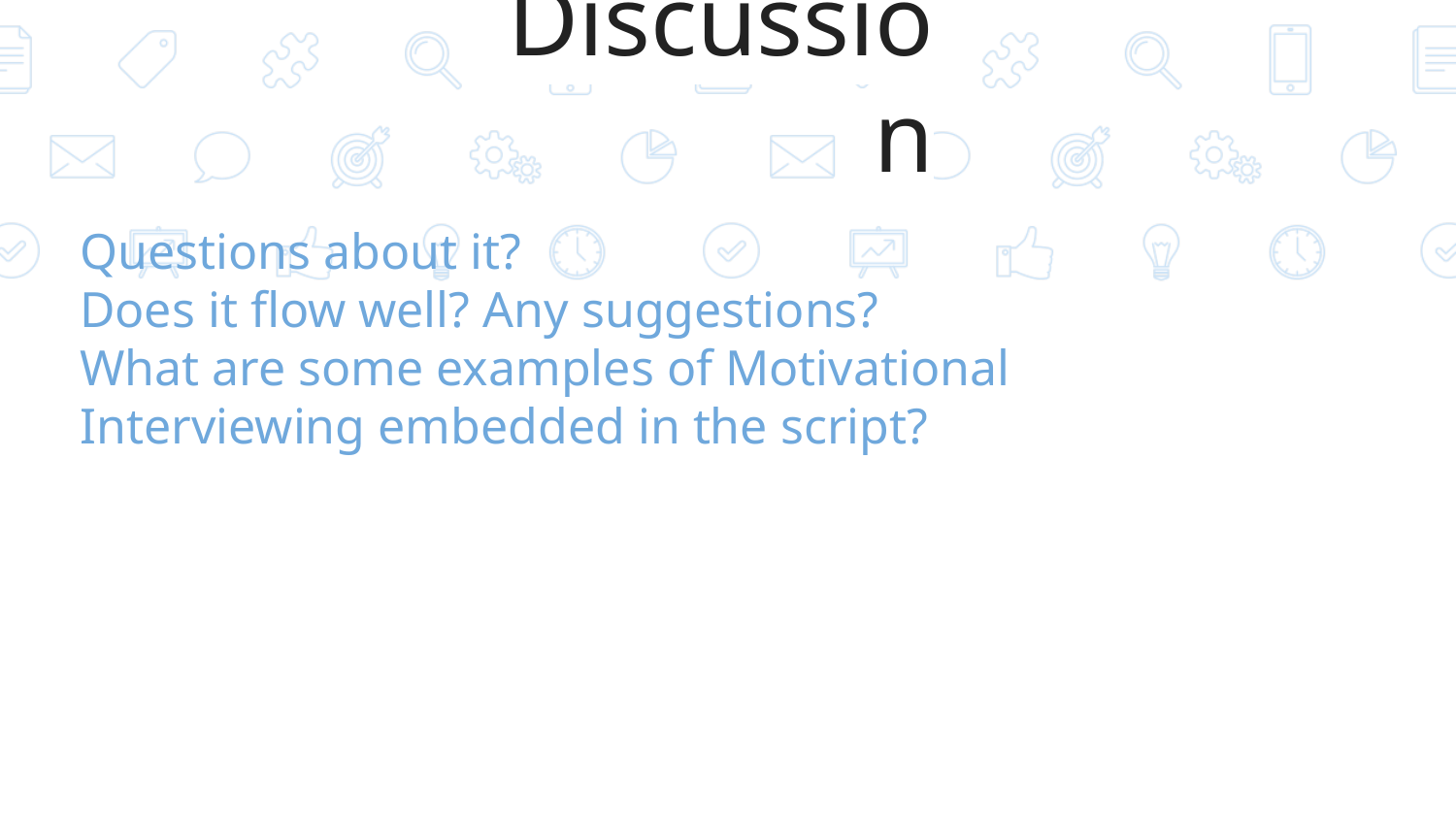

# Discussion
Questions about it?
Does it flow well? Any suggestions?
What are some examples of Motivational Interviewing embedded in the script?

## Slide 73
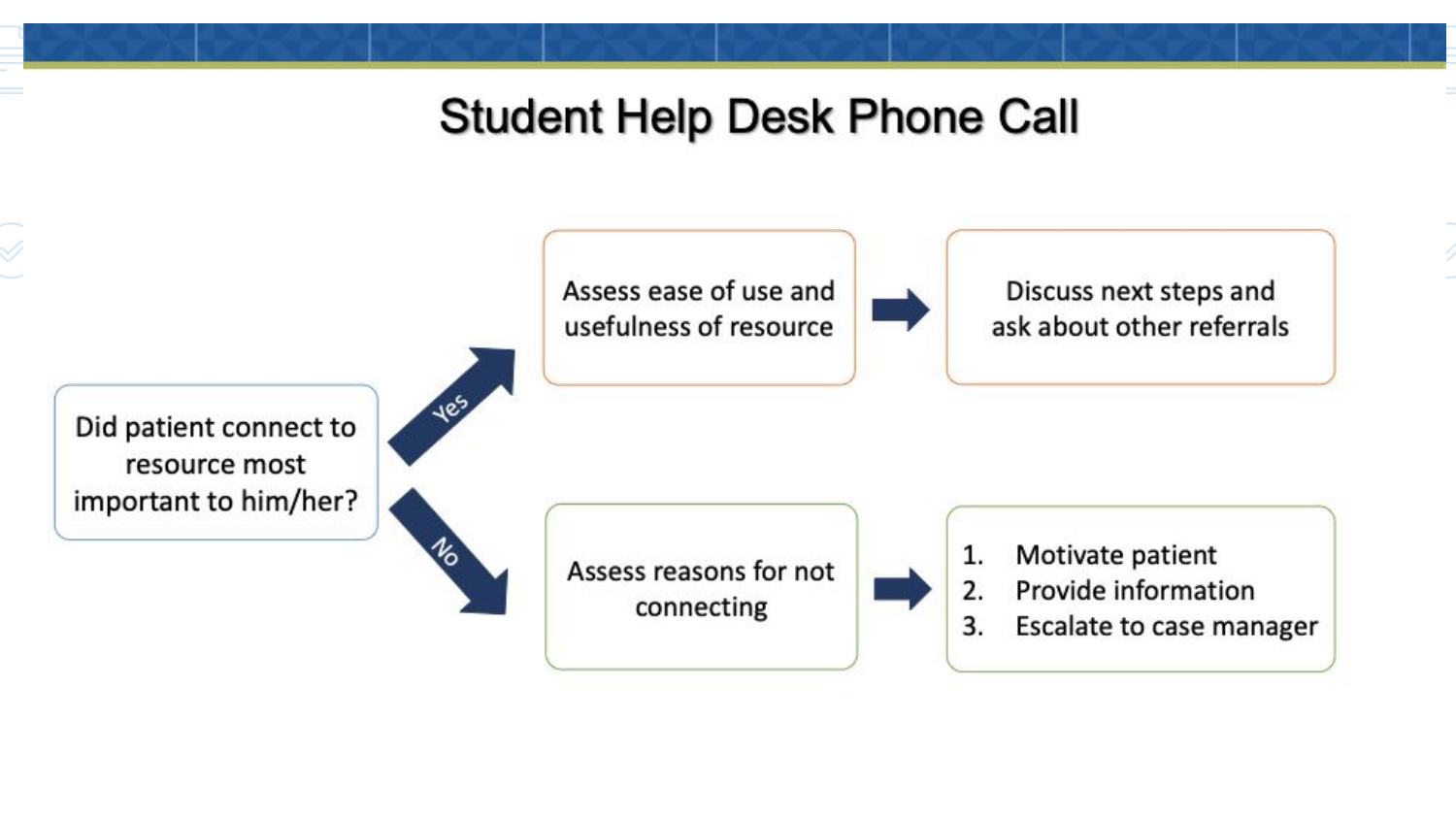

#

## Slide 74
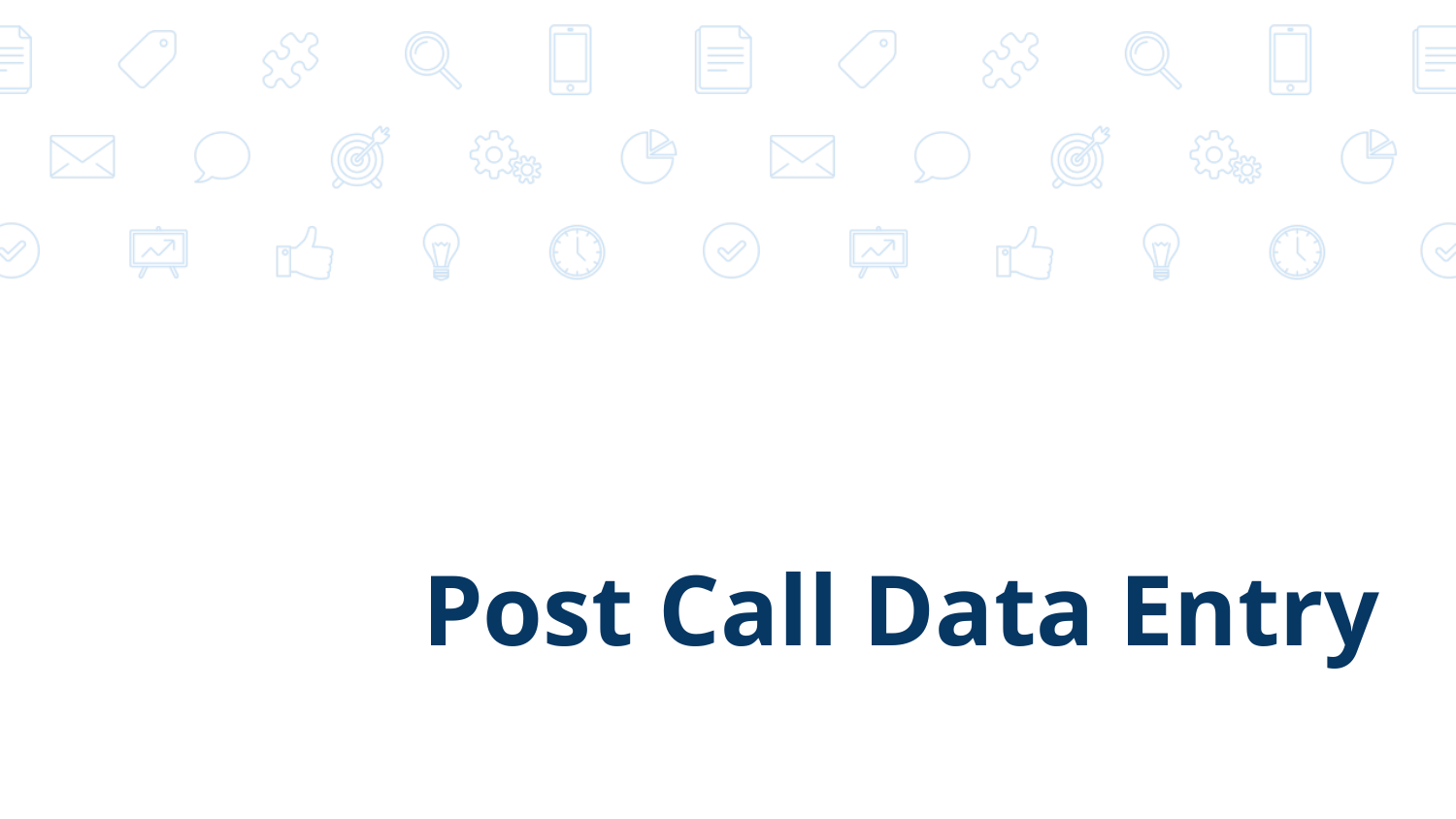

# Post Call Data Entry

## Slide 75
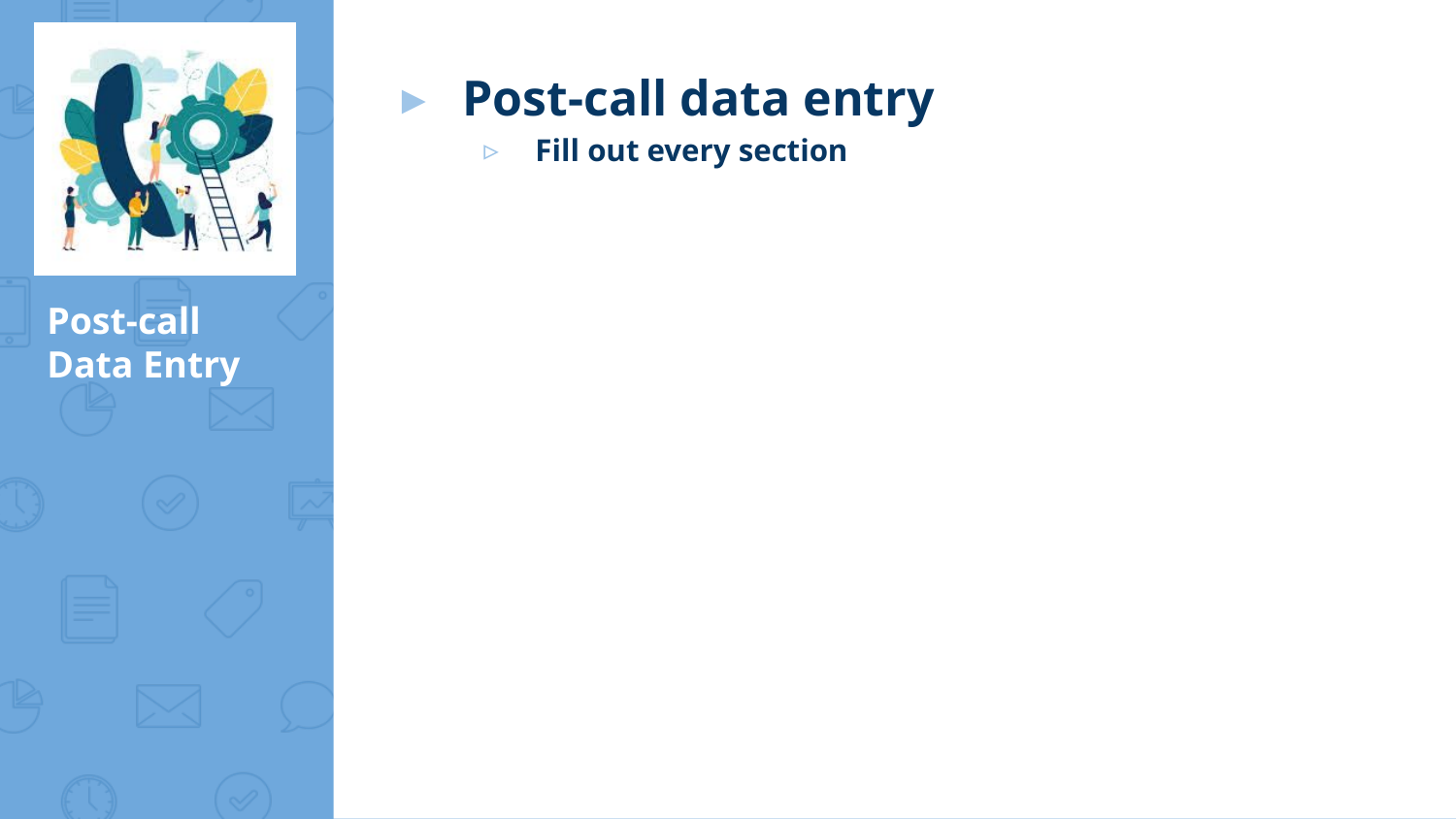

Post-call data entry
Fill out every section
# Post-call Data Entry

## Slide 76
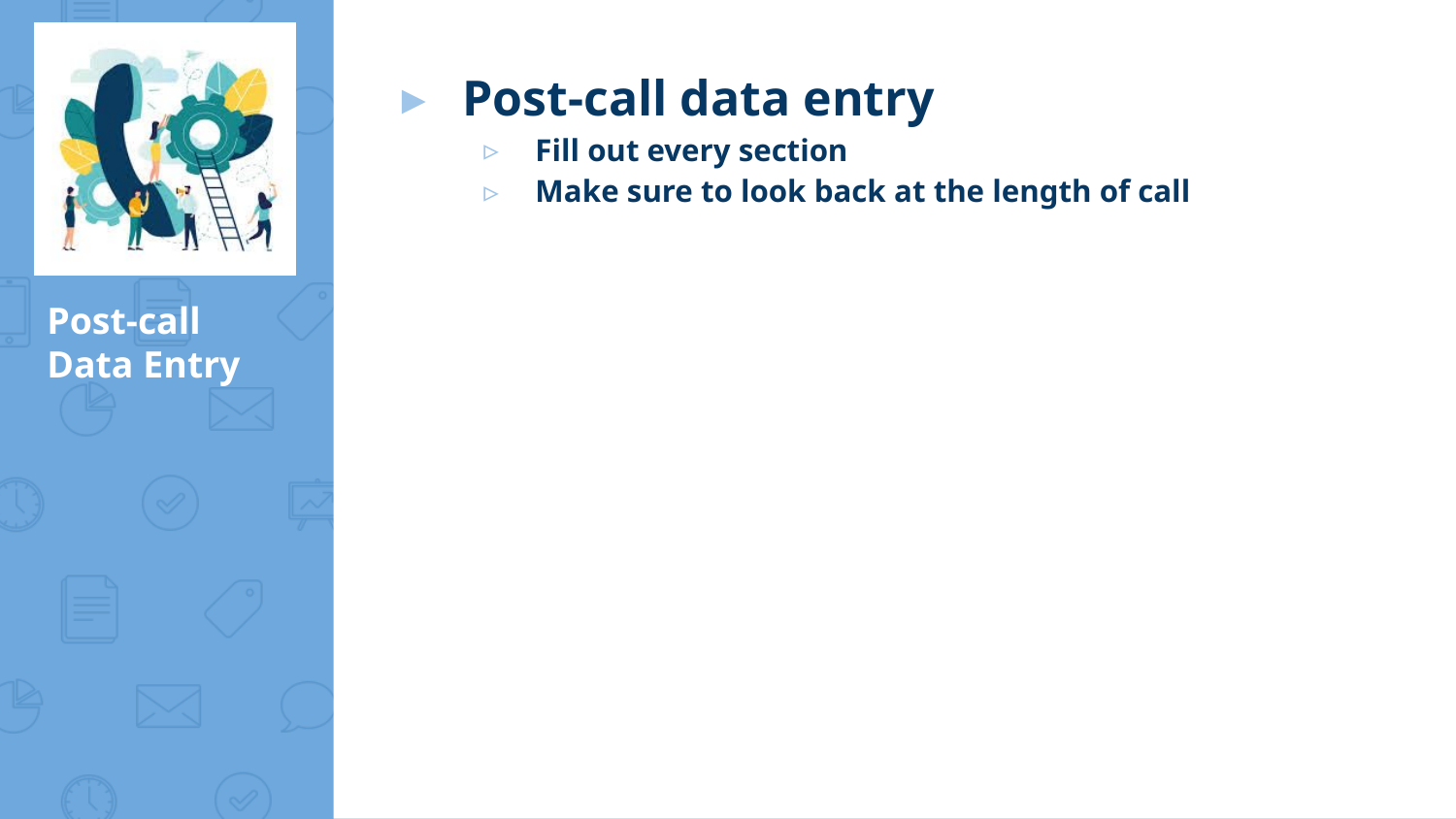

Post-call data entry
Fill out every section
Make sure to look back at the length of call
# Post-call Data Entry

## Slide 77
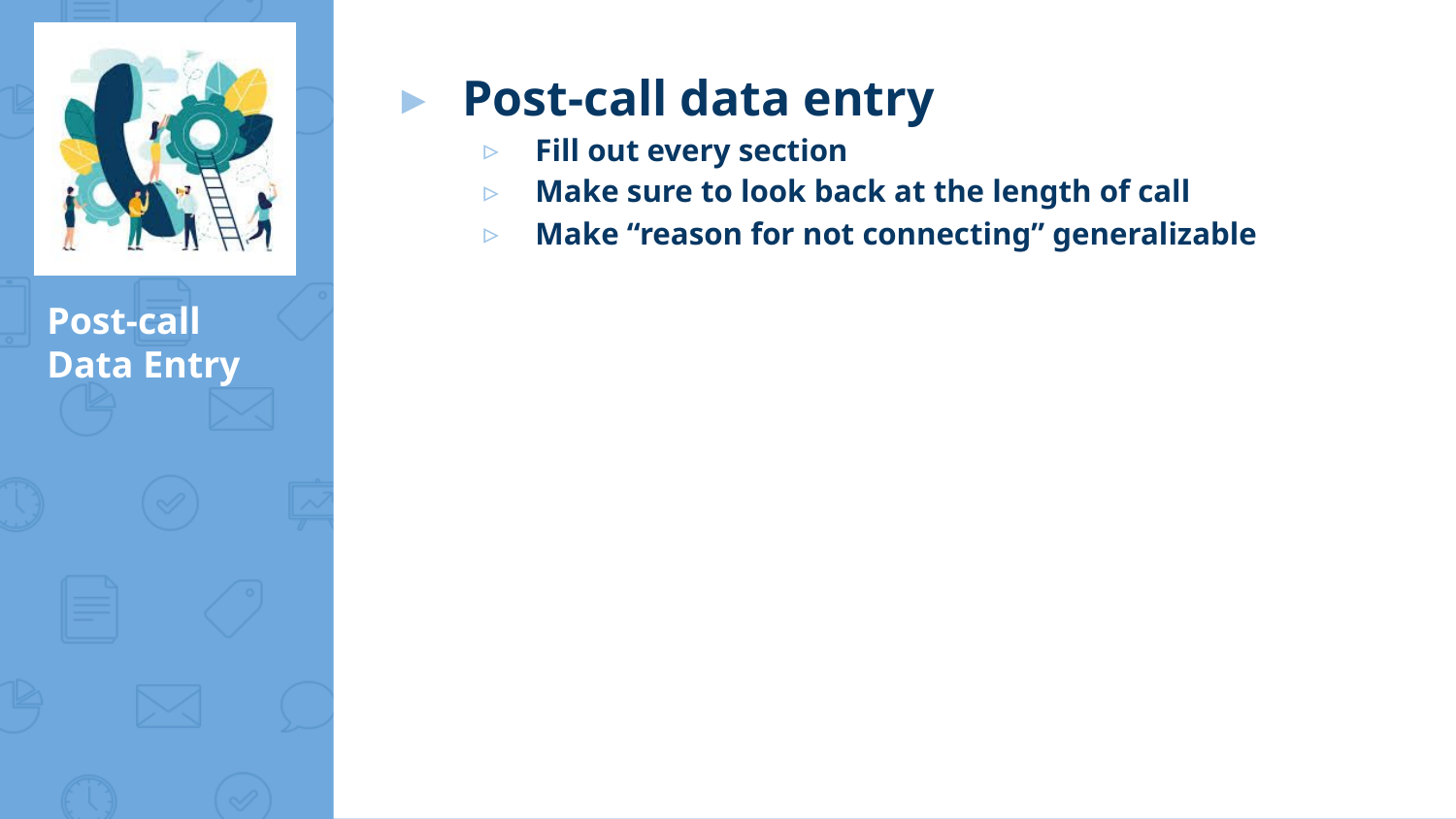

Post-call data entry
Fill out every section
Make sure to look back at the length of call
Make “reason for not connecting” generalizable
# Post-call Data Entry

## Slide 78
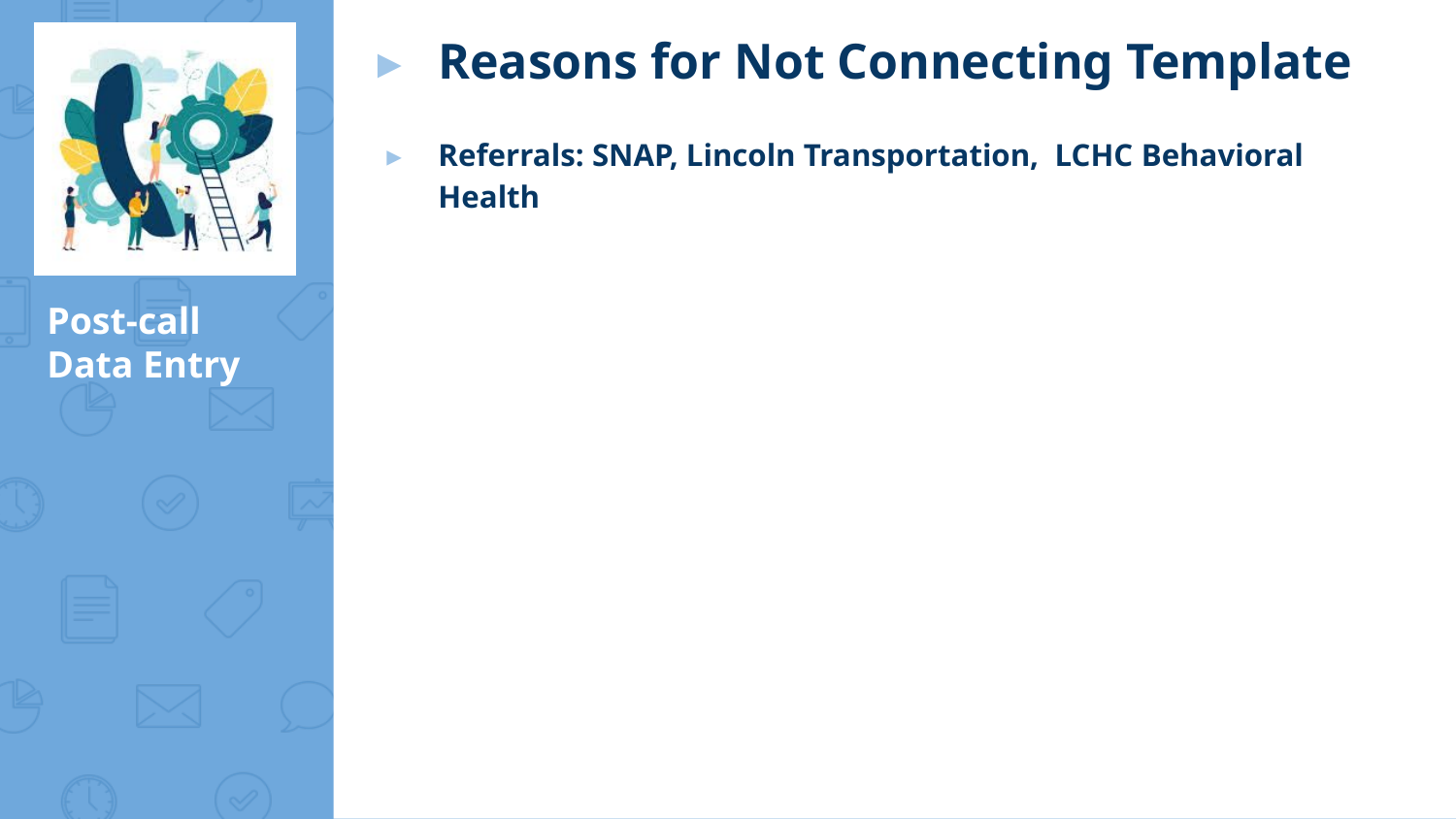

Reasons for Not Connecting Template
Referrals: SNAP, Lincoln Transportation, LCHC Behavioral Health
# Post-call Data Entry

## Slide 79
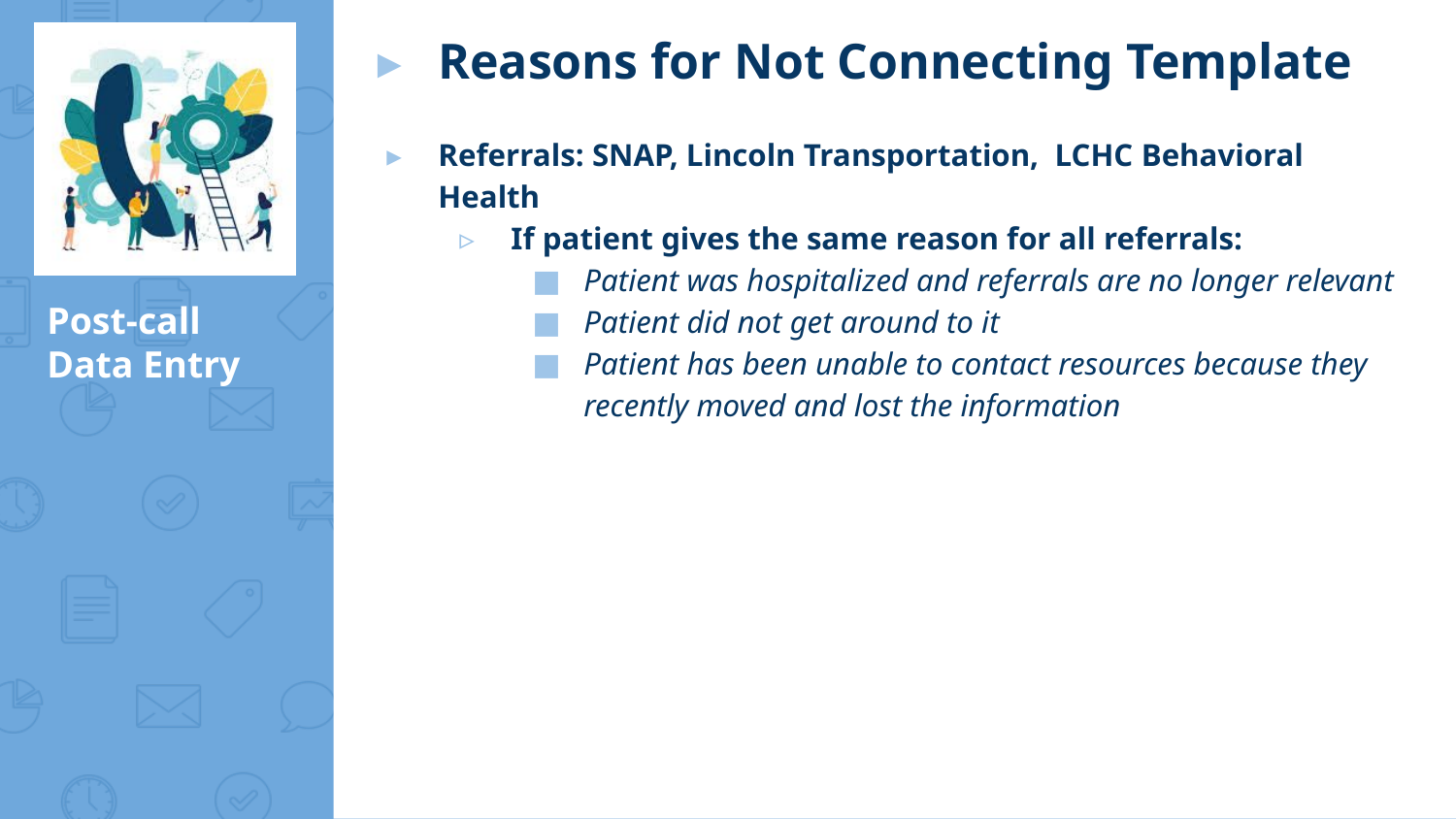

Reasons for Not Connecting Template
Referrals: SNAP, Lincoln Transportation, LCHC Behavioral Health
If patient gives the same reason for all referrals:
Patient was hospitalized and referrals are no longer relevant
Patient did not get around to it
Patient has been unable to contact resources because they recently moved and lost the information
# Post-call Data Entry

## Slide 80
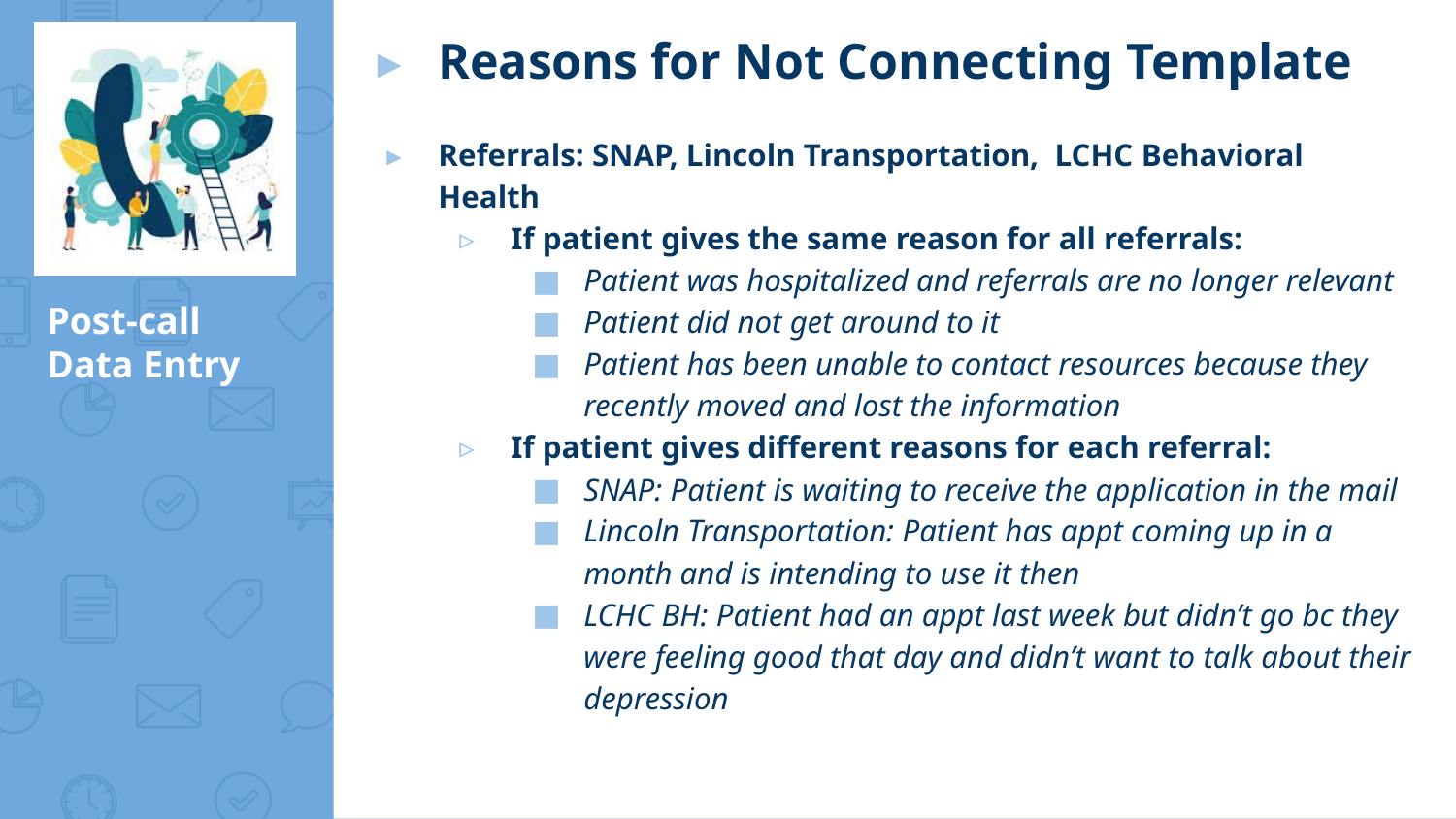

Reasons for Not Connecting Template
Referrals: SNAP, Lincoln Transportation, LCHC Behavioral Health
If patient gives the same reason for all referrals:
Patient was hospitalized and referrals are no longer relevant
Patient did not get around to it
Patient has been unable to contact resources because they recently moved and lost the information
If patient gives different reasons for each referral:
SNAP: Patient is waiting to receive the application in the mail
Lincoln Transportation: Patient has appt coming up in a month and is intending to use it then
LCHC BH: Patient had an appt last week but didn’t go bc they were feeling good that day and didn’t want to talk about their depression
# Post-call Data Entry

## Slide 81
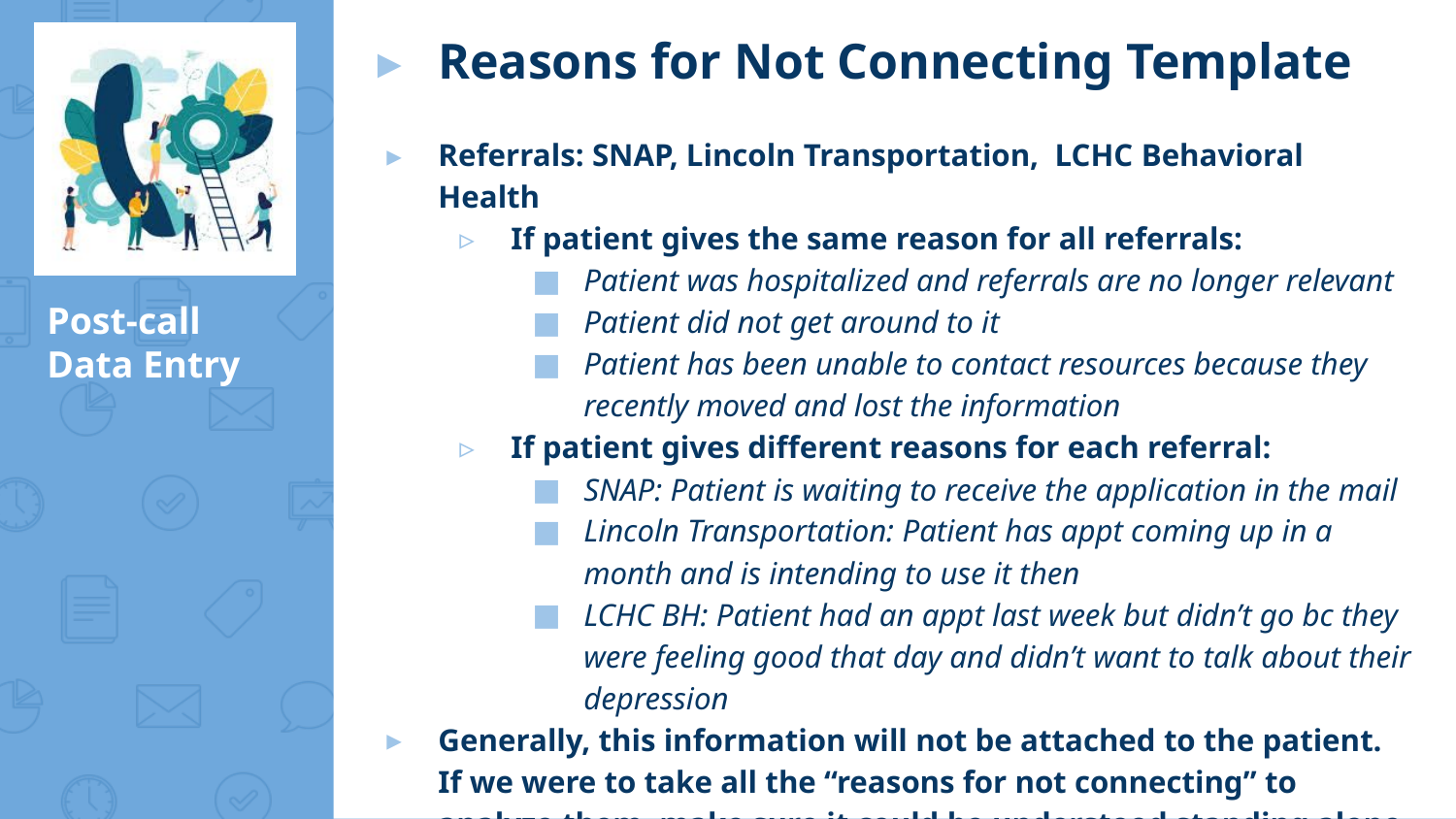

Reasons for Not Connecting Template
Referrals: SNAP, Lincoln Transportation, LCHC Behavioral Health
If patient gives the same reason for all referrals:
Patient was hospitalized and referrals are no longer relevant
Patient did not get around to it
Patient has been unable to contact resources because they recently moved and lost the information
If patient gives different reasons for each referral:
SNAP: Patient is waiting to receive the application in the mail
Lincoln Transportation: Patient has appt coming up in a month and is intending to use it then
LCHC BH: Patient had an appt last week but didn’t go bc they were feeling good that day and didn’t want to talk about their depression
Generally, this information will not be attached to the patient. If we were to take all the “reasons for not connecting” to analyze them, make sure it could be understood standing alone
# Post-call Data Entry

## Slide 82
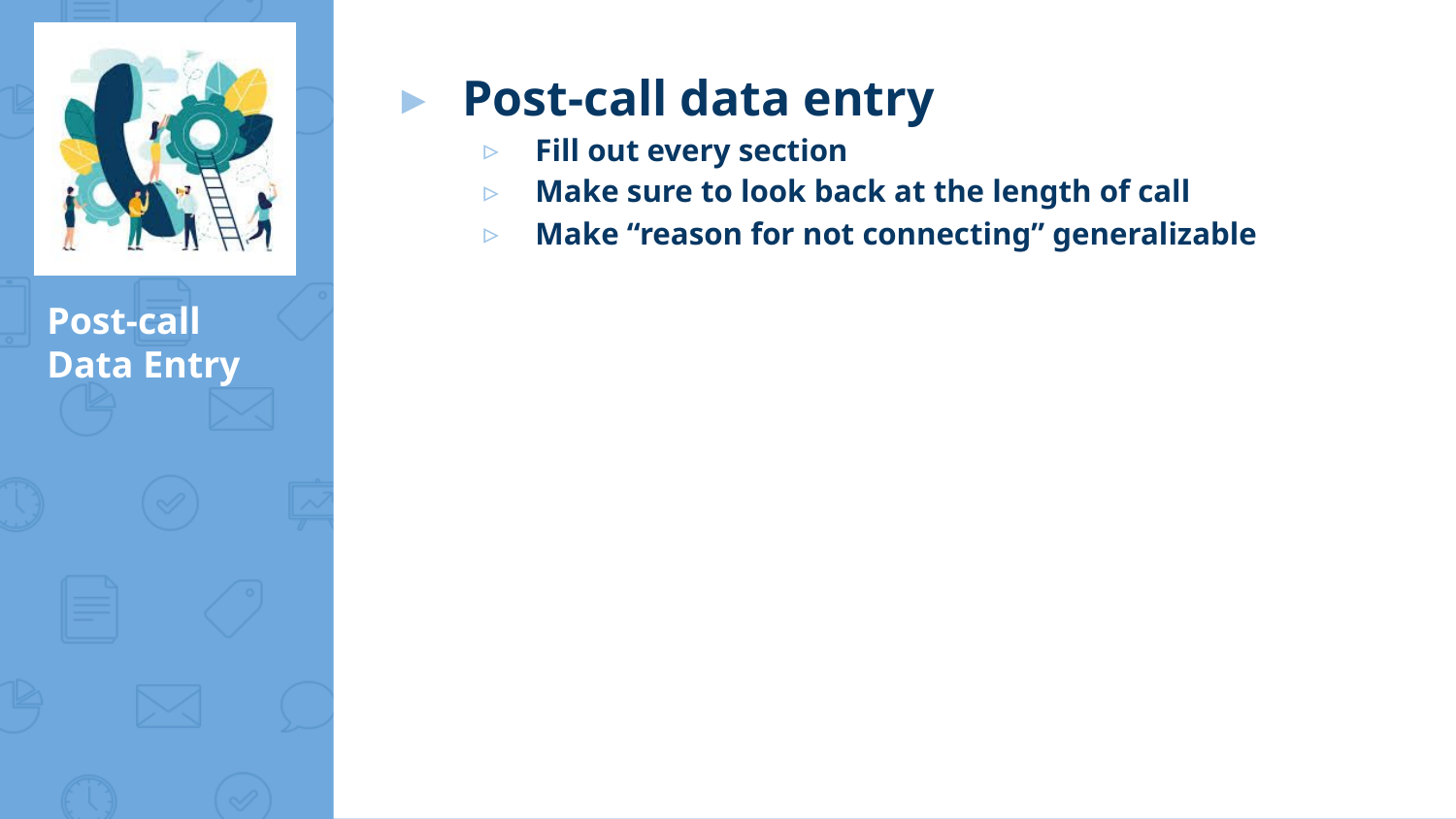

Post-call data entry
Fill out every section
Make sure to look back at the length of call
Make “reason for not connecting” generalizable
# Post-call Data Entry

## Slide 83
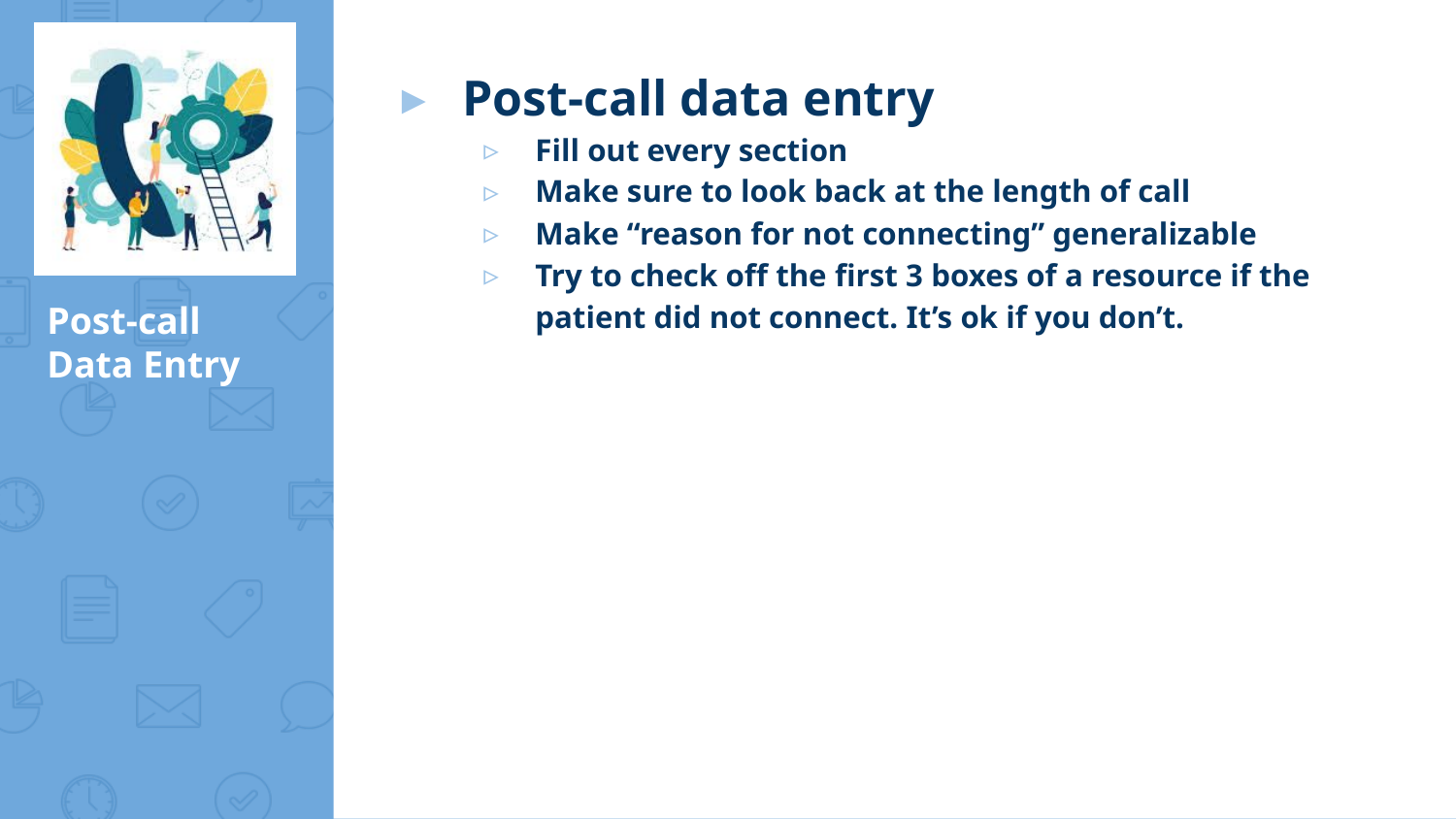

Post-call data entry
Fill out every section
Make sure to look back at the length of call
Make “reason for not connecting” generalizable
Try to check off the first 3 boxes of a resource if the patient did not connect. It’s ok if you don’t.
# Post-call Data Entry

## Slide 84
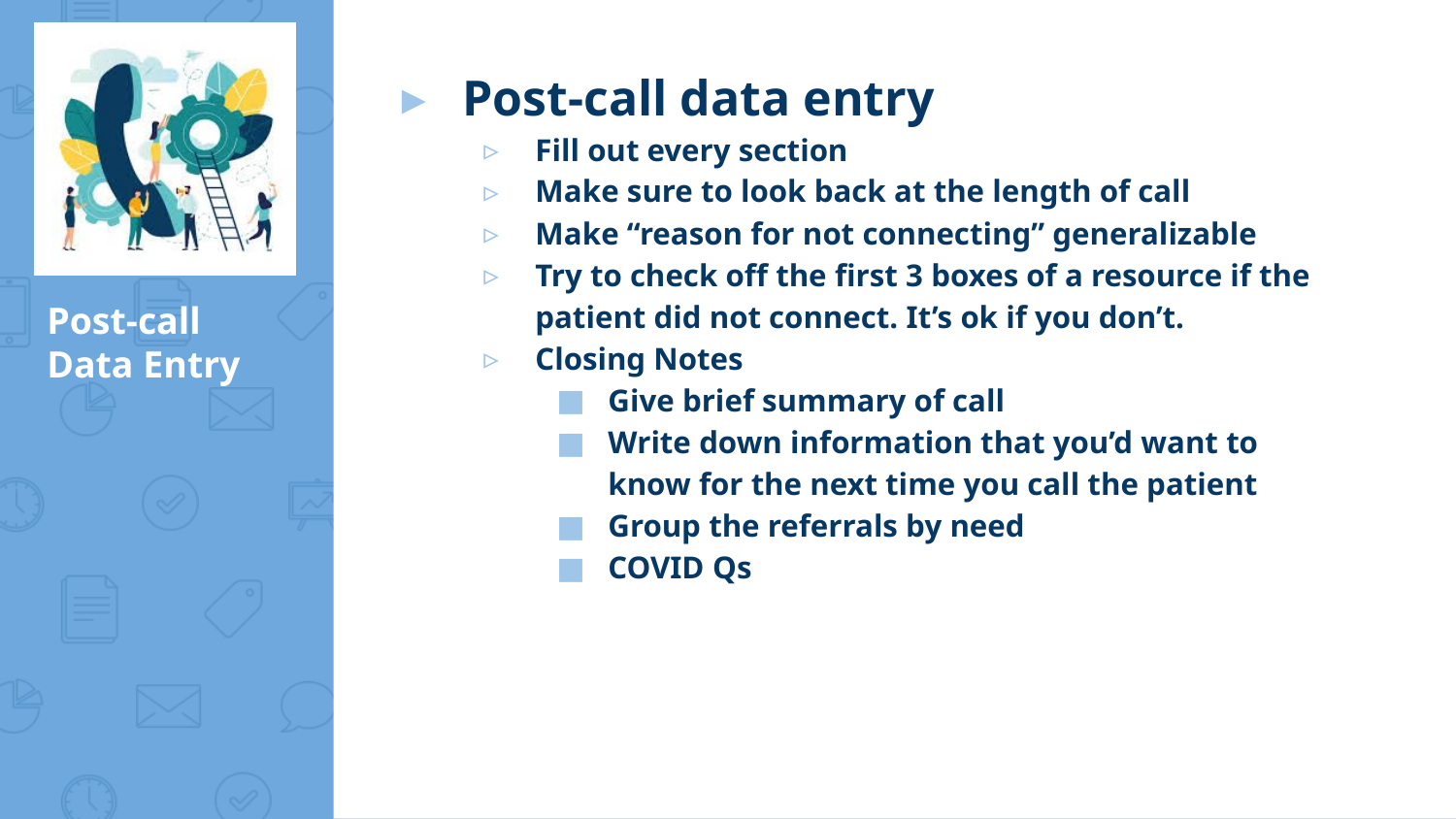

Post-call data entry
Fill out every section
Make sure to look back at the length of call
Make “reason for not connecting” generalizable
Try to check off the first 3 boxes of a resource if the patient did not connect. It’s ok if you don’t.
Closing Notes
Give brief summary of call
Write down information that you’d want to know for the next time you call the patient
Group the referrals by need
COVID Qs
# Post-call Data Entry

## Slide 85
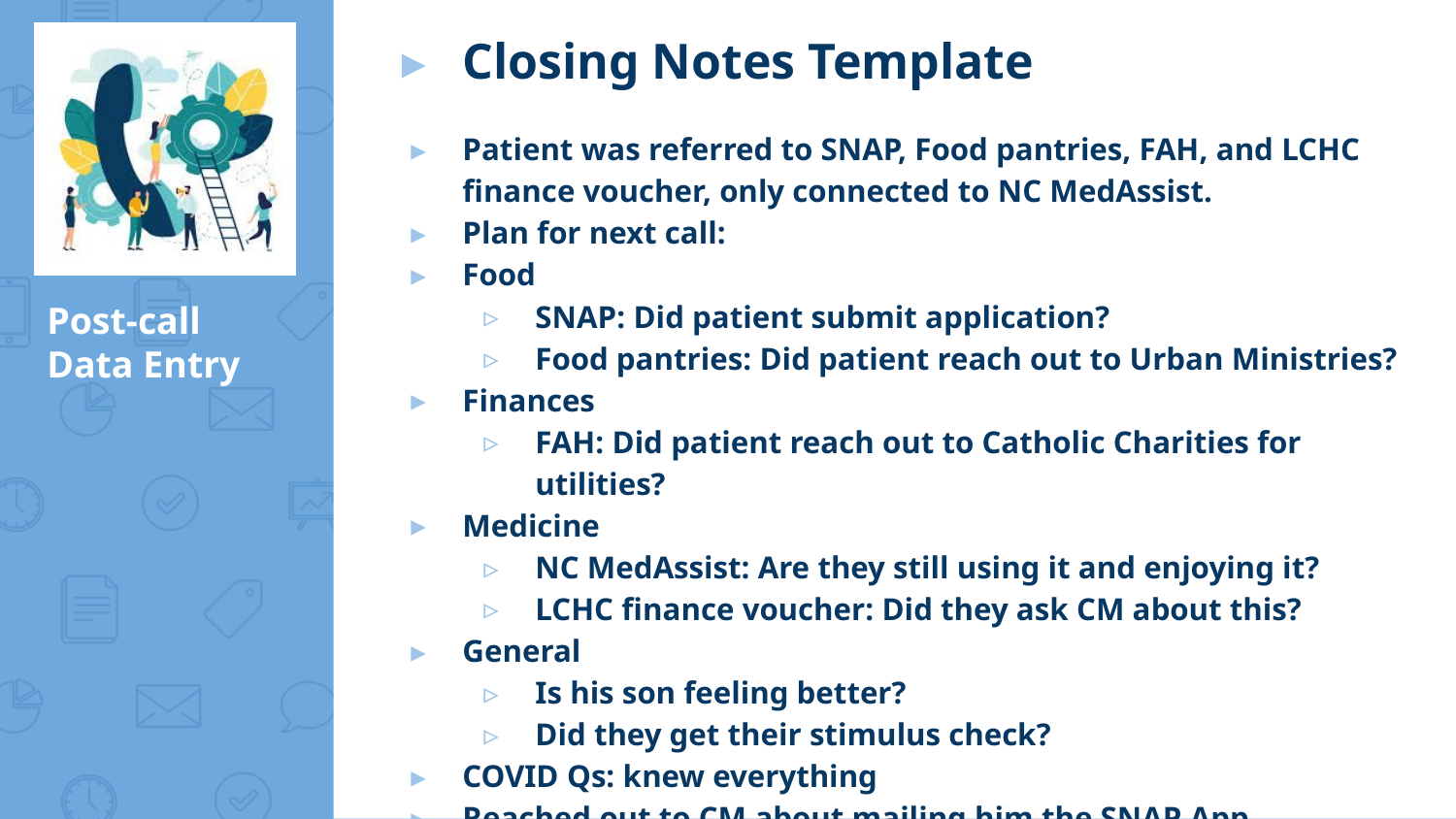

Closing Notes Template
Patient was referred to SNAP, Food pantries, FAH, and LCHC finance voucher, only connected to NC MedAssist.
Plan for next call:
Food
SNAP: Did patient submit application?
Food pantries: Did patient reach out to Urban Ministries?
Finances
FAH: Did patient reach out to Catholic Charities for utilities?
Medicine
NC MedAssist: Are they still using it and enjoying it?
LCHC finance voucher: Did they ask CM about this?
General
Is his son feeling better?
Did they get their stimulus check?
COVID Qs: knew everything
Reached out to CM about mailing him the SNAP App
# Post-call Data Entry

## Slide 86
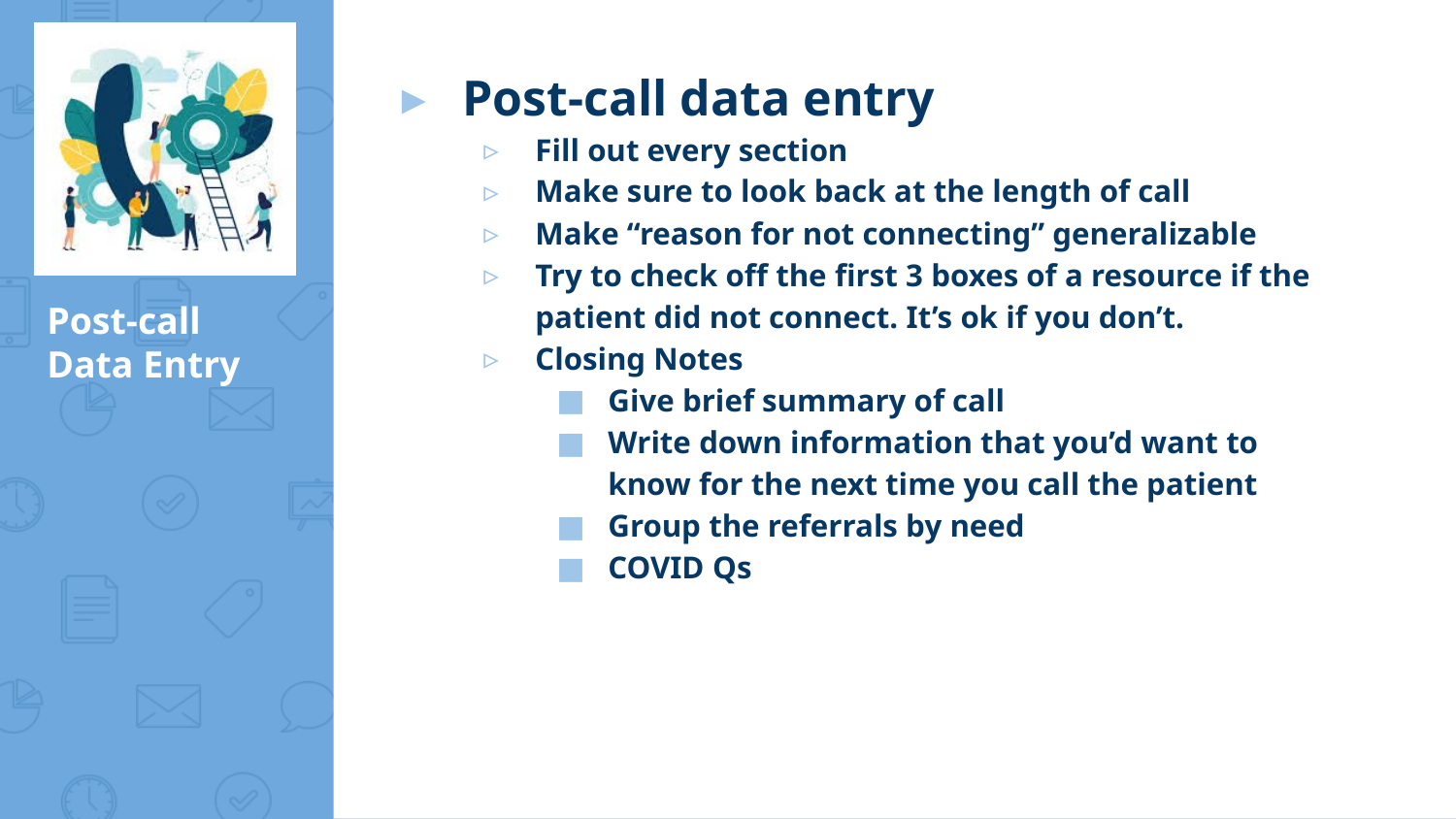

Post-call data entry
Fill out every section
Make sure to look back at the length of call
Make “reason for not connecting” generalizable
Try to check off the first 3 boxes of a resource if the patient did not connect. It’s ok if you don’t.
Closing Notes
Give brief summary of call
Write down information that you’d want to know for the next time you call the patient
Group the referrals by need
COVID Qs
# Post-call Data Entry

## Slide 87
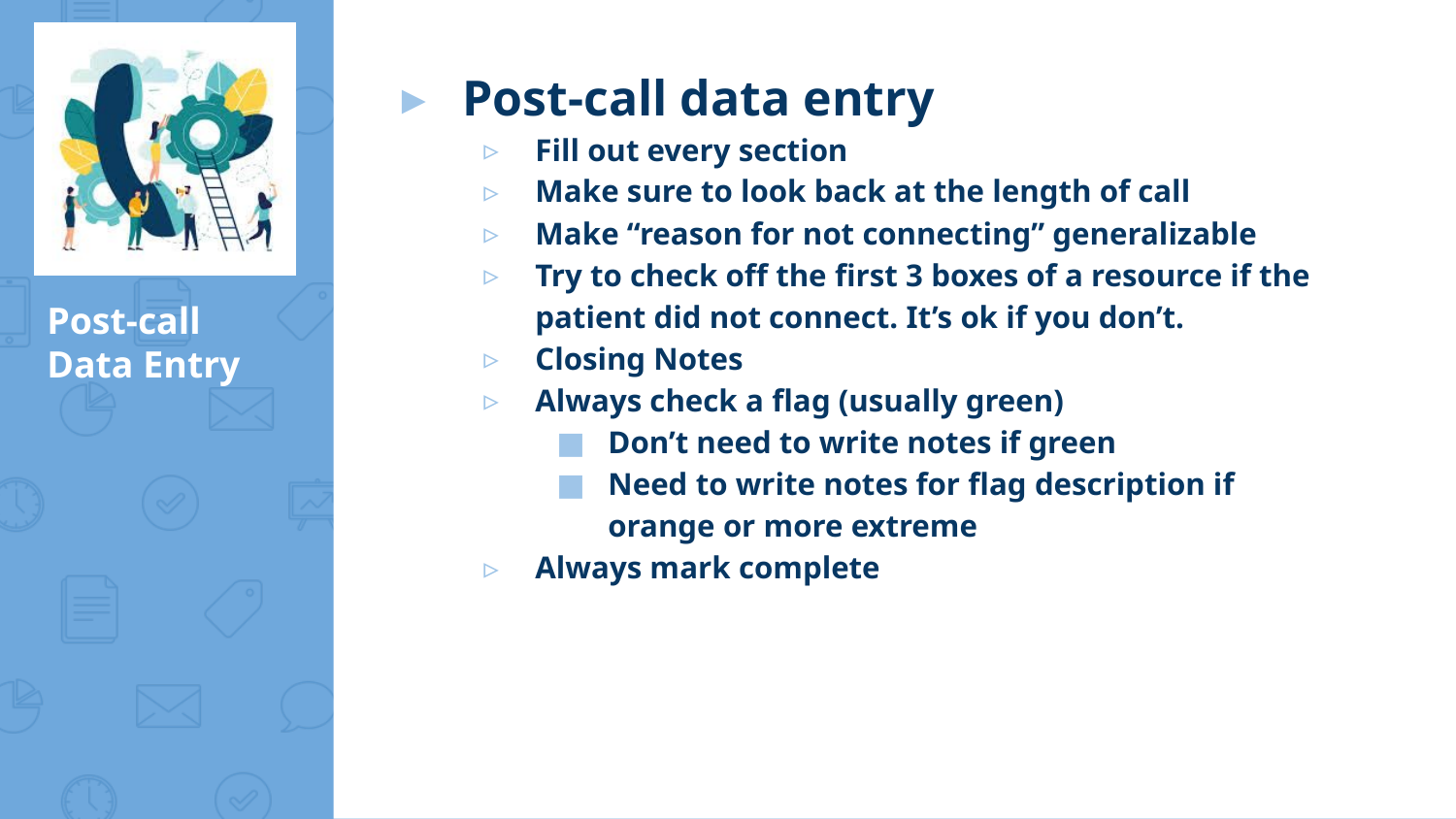

Post-call data entry
Fill out every section
Make sure to look back at the length of call
Make “reason for not connecting” generalizable
Try to check off the first 3 boxes of a resource if the patient did not connect. It’s ok if you don’t.
Closing Notes
Always check a flag (usually green)
Don’t need to write notes if green
Need to write notes for flag description if orange or more extreme
Always mark complete
# Post-call Data Entry

## Slide 88
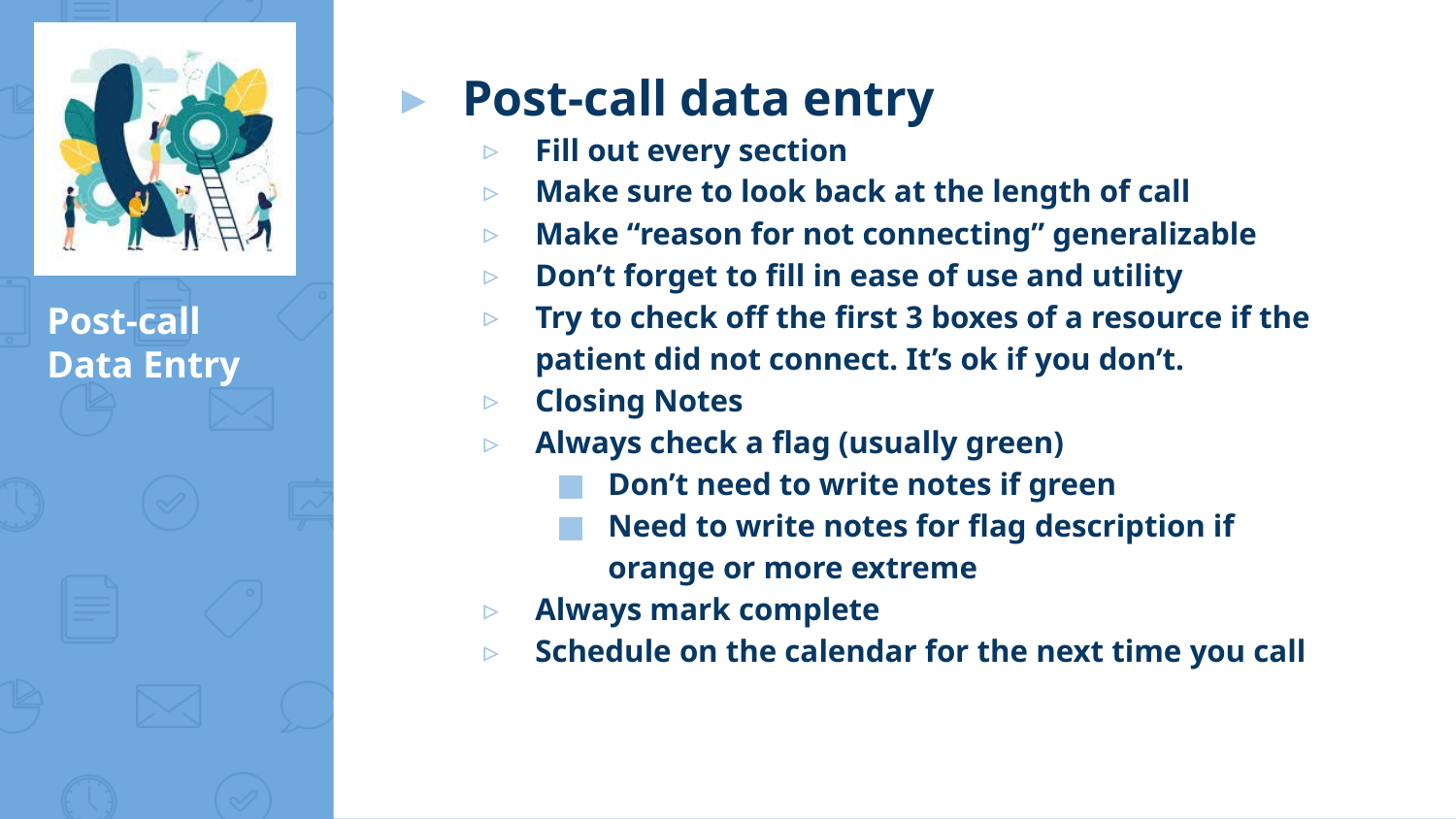

Post-call data entry
Fill out every section
Make sure to look back at the length of call
Make “reason for not connecting” generalizable
Don’t forget to fill in ease of use and utility
Try to check off the first 3 boxes of a resource if the patient did not connect. It’s ok if you don’t.
Closing Notes
Always check a flag (usually green)
Don’t need to write notes if green
Need to write notes for flag description if orange or more extreme
Always mark complete
Schedule on the calendar for the next time you call
# Post-call Data Entry

## Slide 89
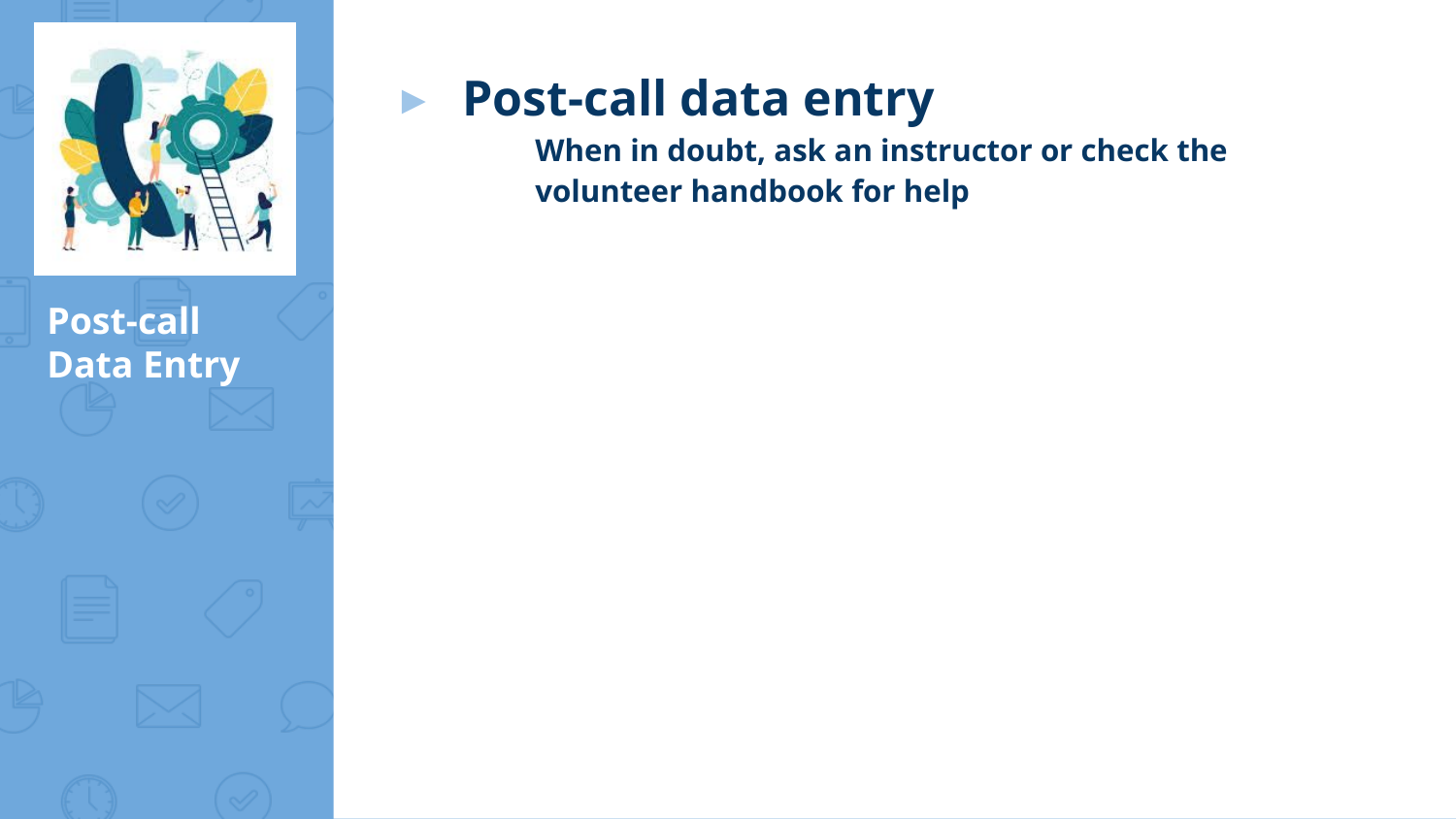

Post-call data entry
When in doubt, ask an instructor or check the volunteer handbook for help
# Post-call Data Entry

## Slide 90
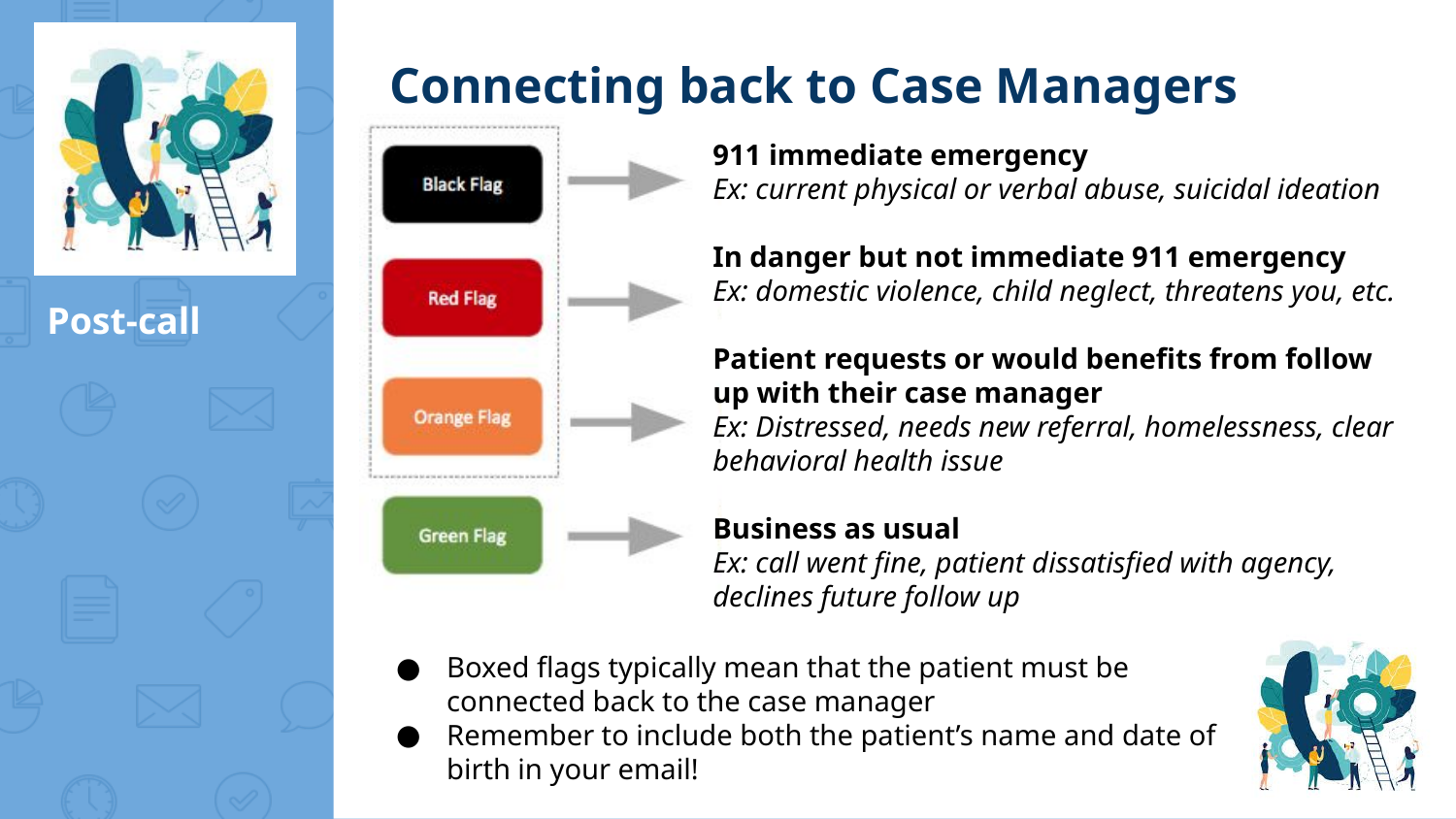

Connecting back to Case Managers
911 immediate emergency
Ex: current physical or verbal abuse, suicidal ideation
In danger but not immediate 911 emergency
Ex: domestic violence, child neglect, threatens you, etc.
Patient requests or would benefits from follow up with their case manager
Ex: Distressed, needs new referral, homelessness, clear behavioral health issue
Business as usual
Ex: call went fine, patient dissatisfied with agency,
declines future follow up
# Post-call
Boxed flags typically mean that the patient must be connected back to the case manager
Remember to include both the patient’s name and date of birth in your email!

## Slide 91
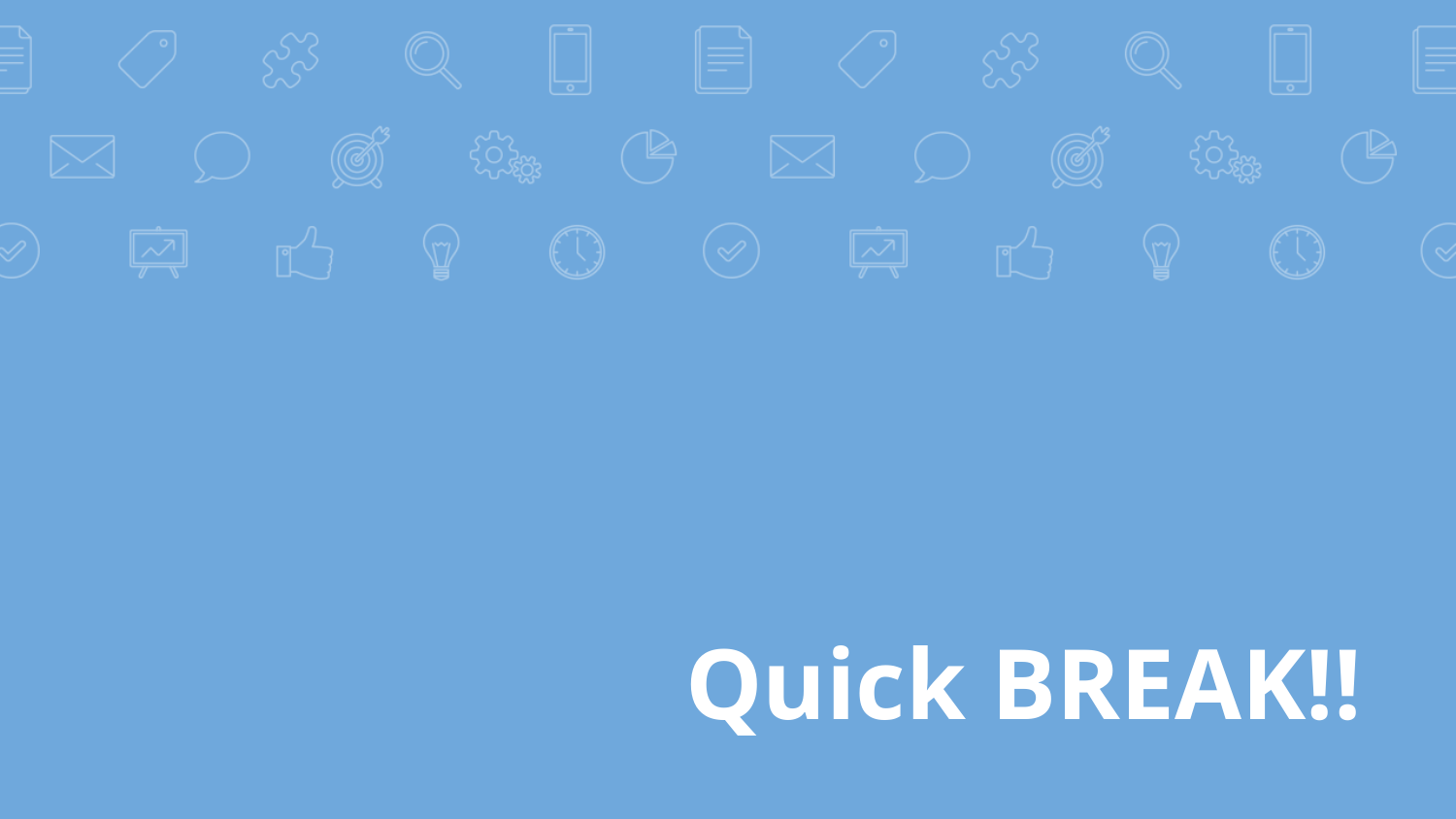

# Quick BREAK!!

## Slide 92
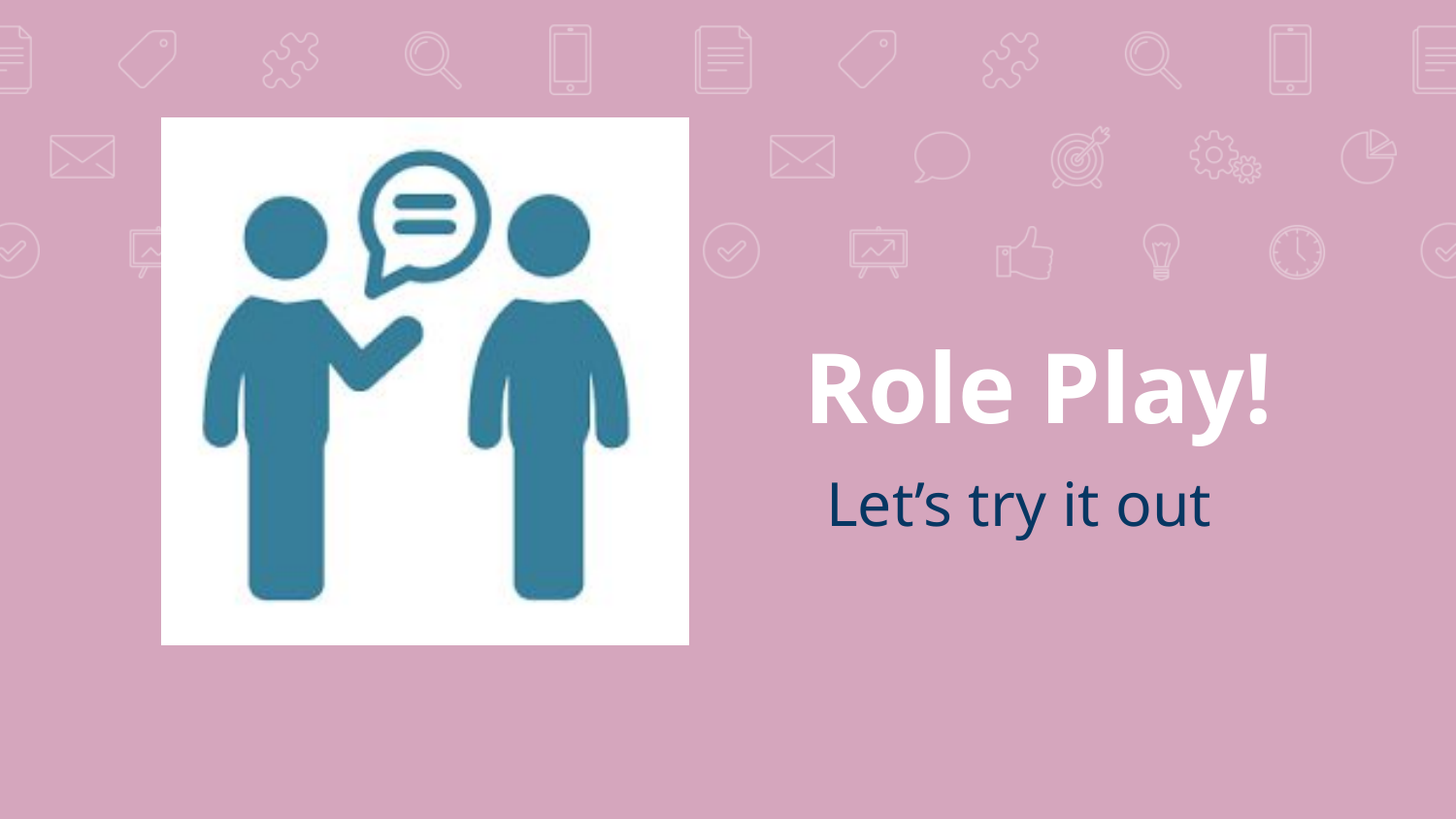

# Role Play!
Let’s try it out

## Slide 93
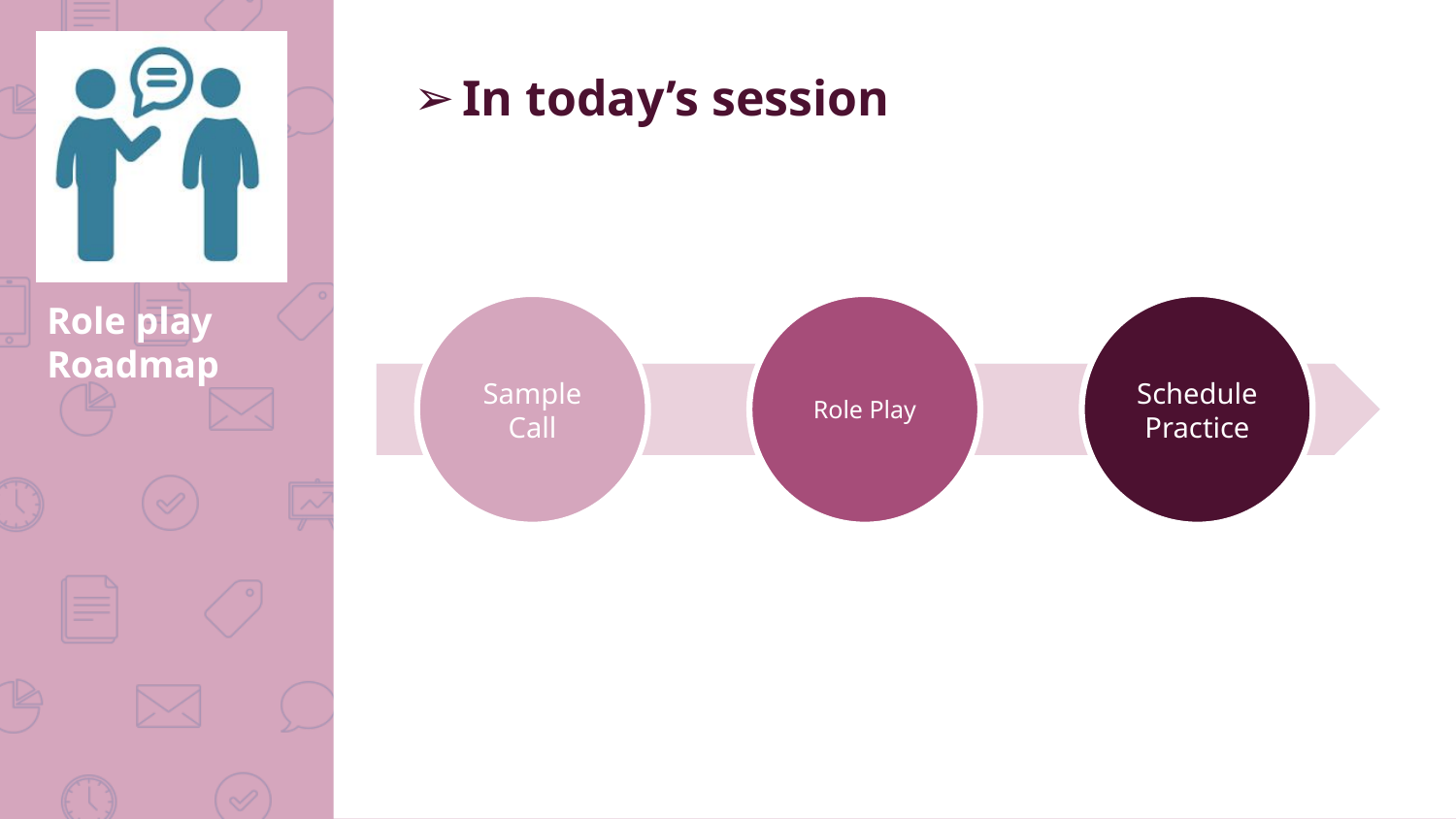

In today’s session
# Role play
Roadmap
Shadow
Sample Call
Role Play
Schedule Practice

## Slide 94
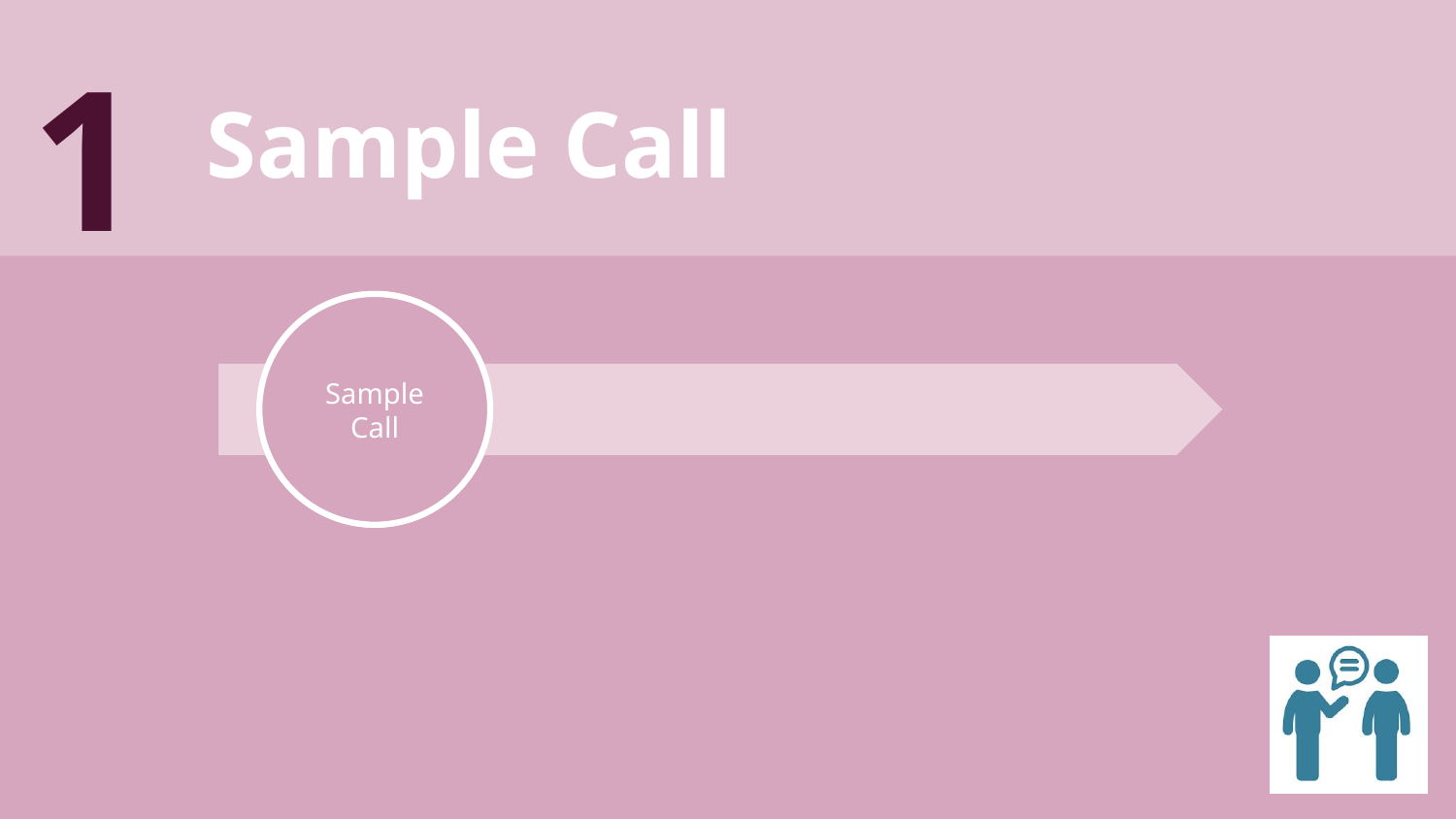

1
Sample Call
Shadow
Sample Call

## Slide 95
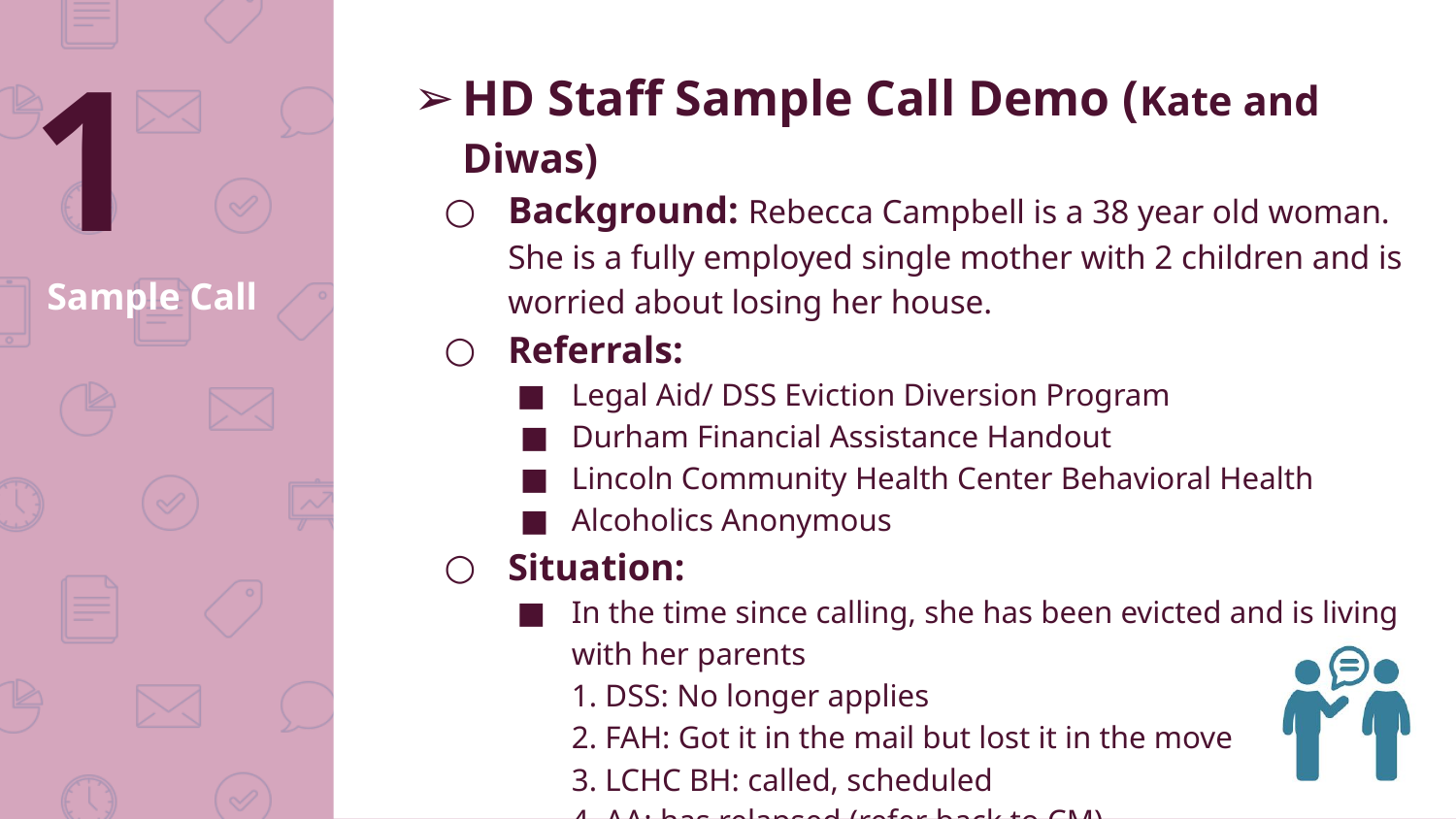

1
HD Staff Sample Call Demo (Kate and Diwas)
Background: Rebecca Campbell is a 38 year old woman. She is a fully employed single mother with 2 children and is worried about losing her house.
Referrals:
Legal Aid/ DSS Eviction Diversion Program
Durham Financial Assistance Handout
Lincoln Community Health Center Behavioral Health
Alcoholics Anonymous
Situation:
In the time since calling, she has been evicted and is living with her parents
1. DSS: No longer applies
2. FAH: Got it in the mail but lost it in the move
3. LCHC BH: called, scheduled
4. AA: has relapsed (refer back to CM)
# Sample Call

## Slide 96
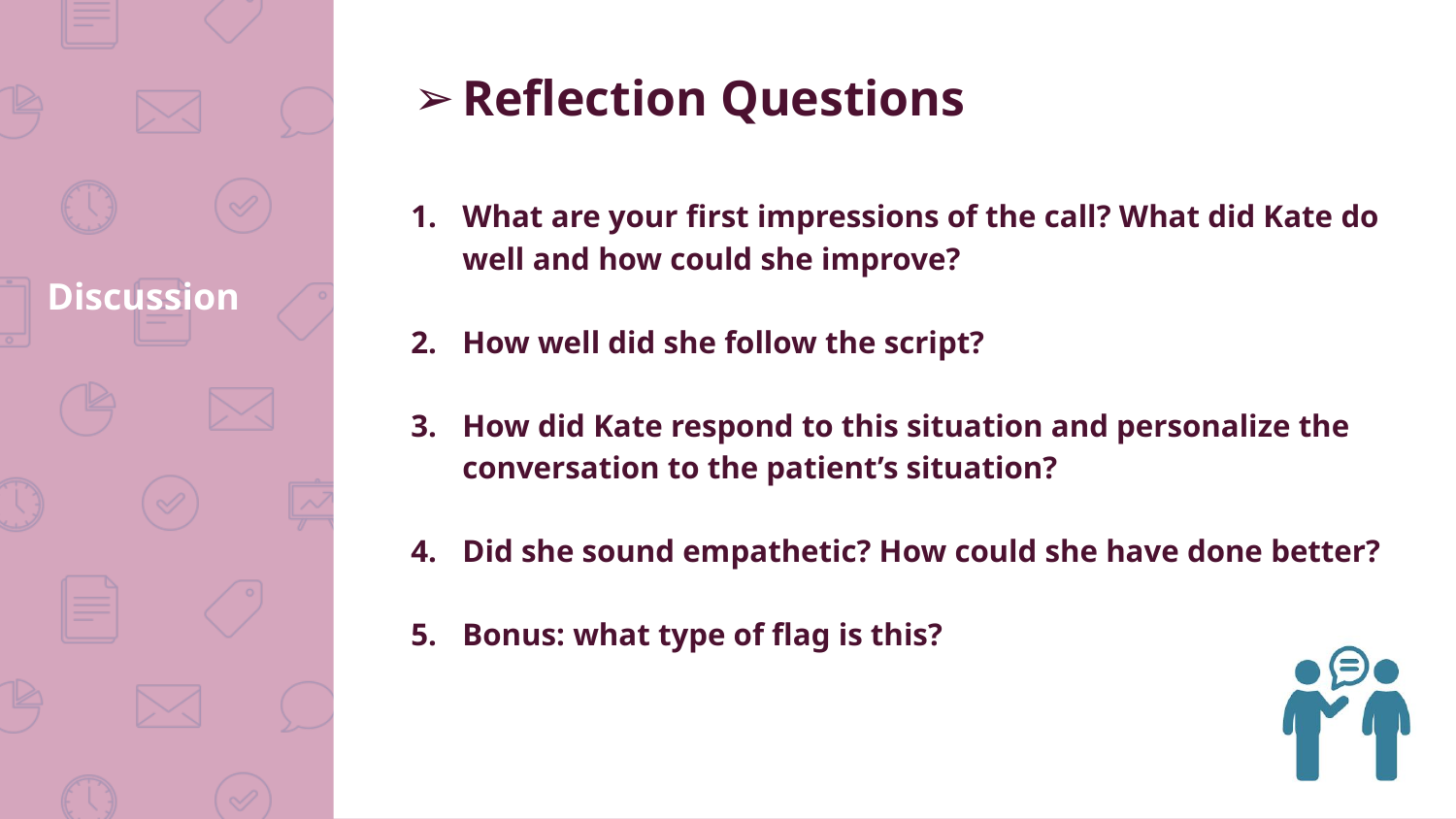

Reflection Questions
What are your first impressions of the call? What did Kate do well and how could she improve?
How well did she follow the script?
How did Kate respond to this situation and personalize the conversation to the patient’s situation?
Did she sound empathetic? How could she have done better?
Bonus: what type of flag is this?
# Discussion

## Slide 97
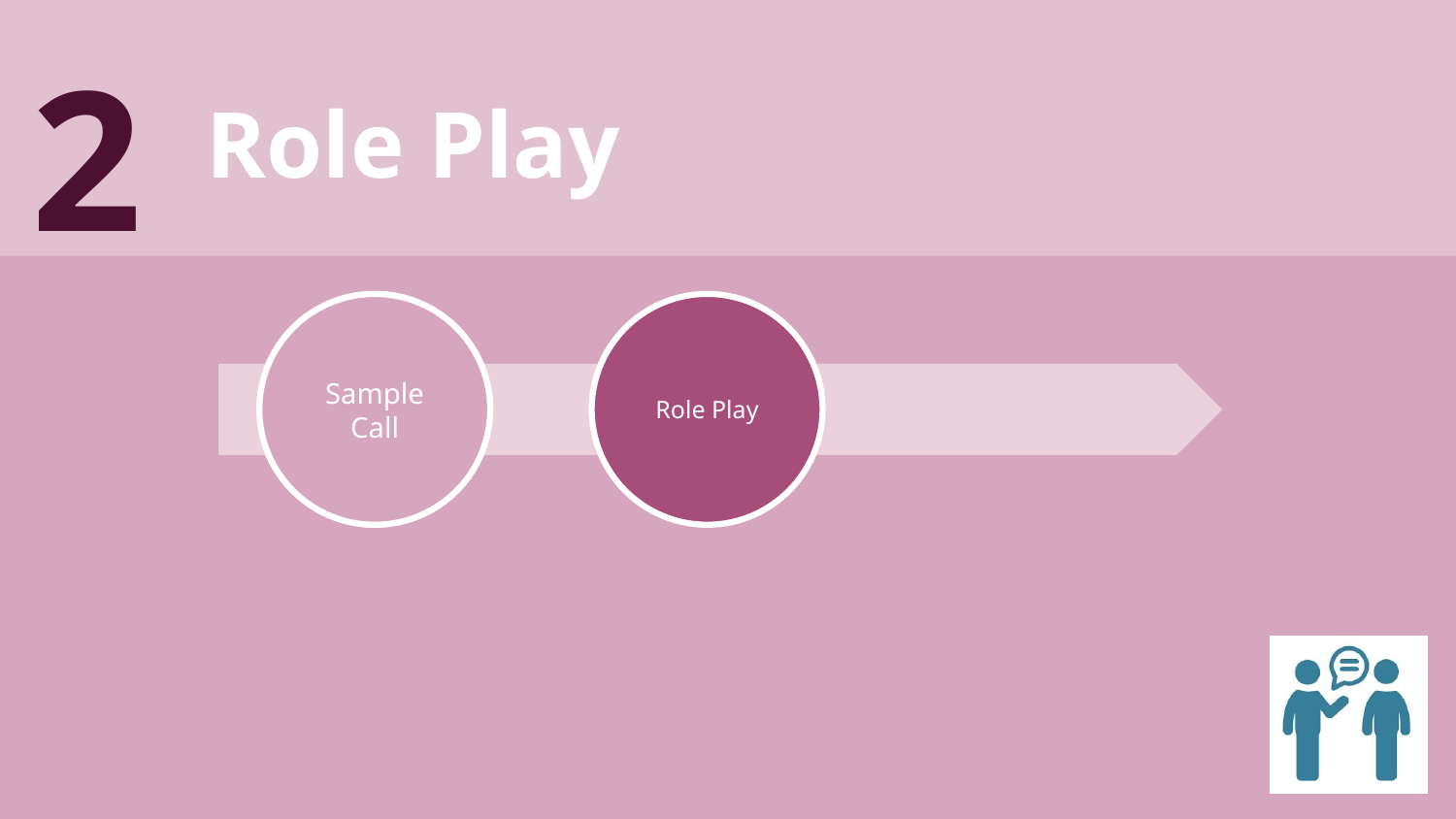

2
Role Play
Shadow
Sample Call
Role Play

## Slide 98
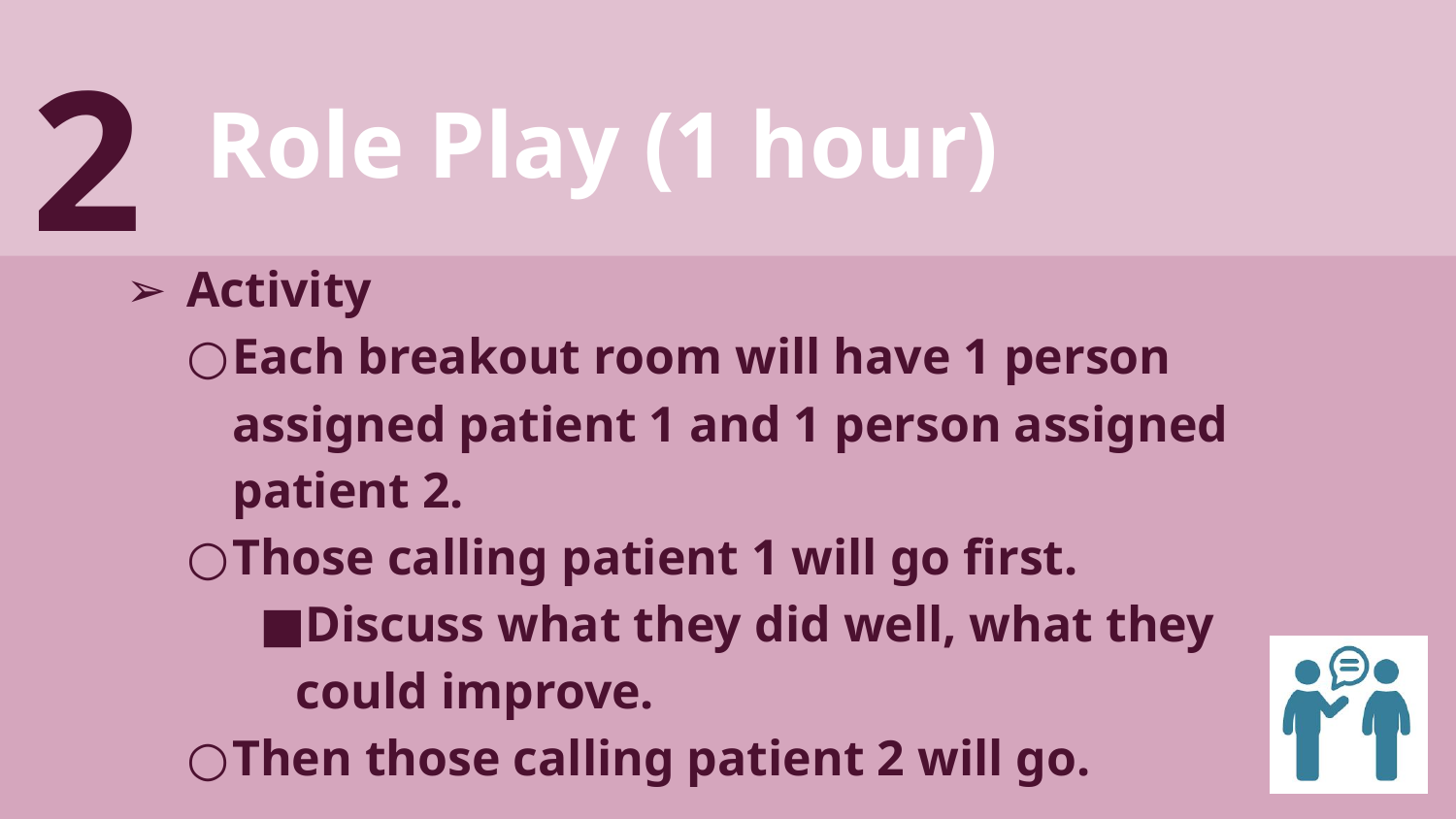

2
Role Play (1 hour)
Activity
Each breakout room will have 1 person assigned patient 1 and 1 person assigned patient 2.
Those calling patient 1 will go first.
Discuss what they did well, what they could improve.
Then those calling patient 2 will go.

## Slide 99
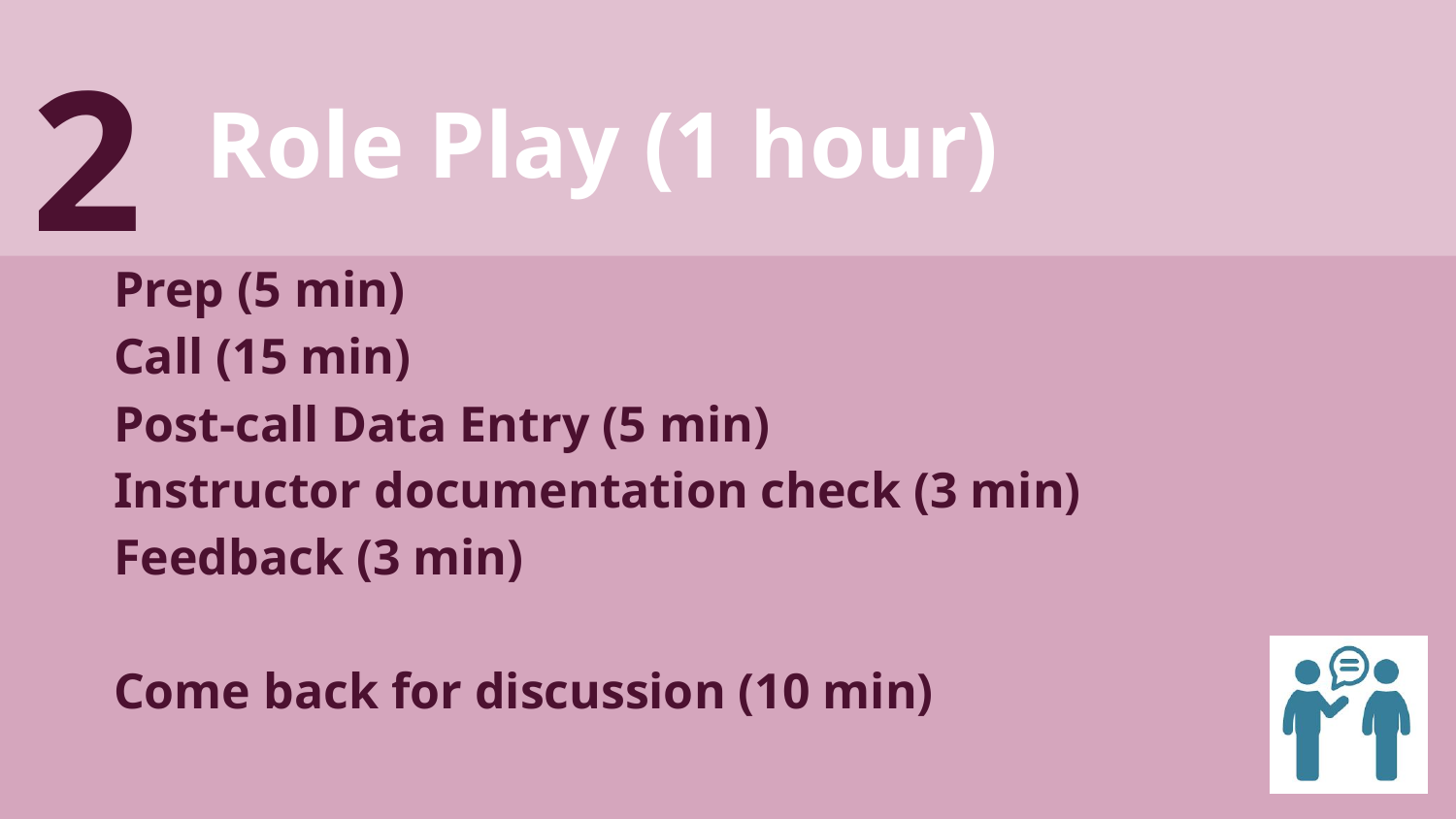

2
Role Play (1 hour)
Prep (5 min)
Call (15 min)
Post-call Data Entry (5 min)
Instructor documentation check (3 min)
Feedback (3 min)
Come back for discussion (10 min)

## Slide 100
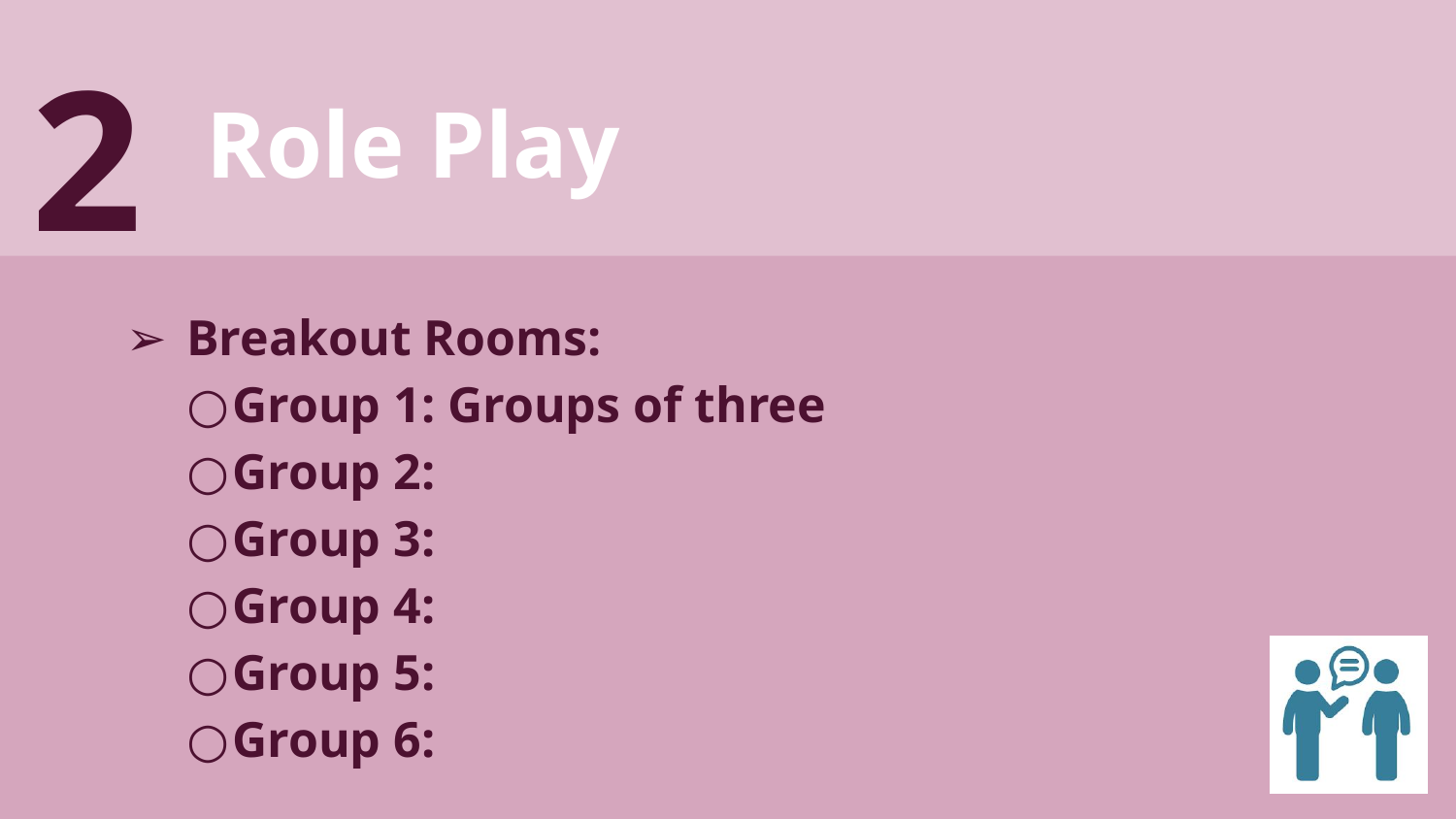

2
Role Play
Breakout Rooms:
Group 1: Groups of three
Group 2:
Group 3:
Group 4:
Group 5:
Group 6:

## Slide 101
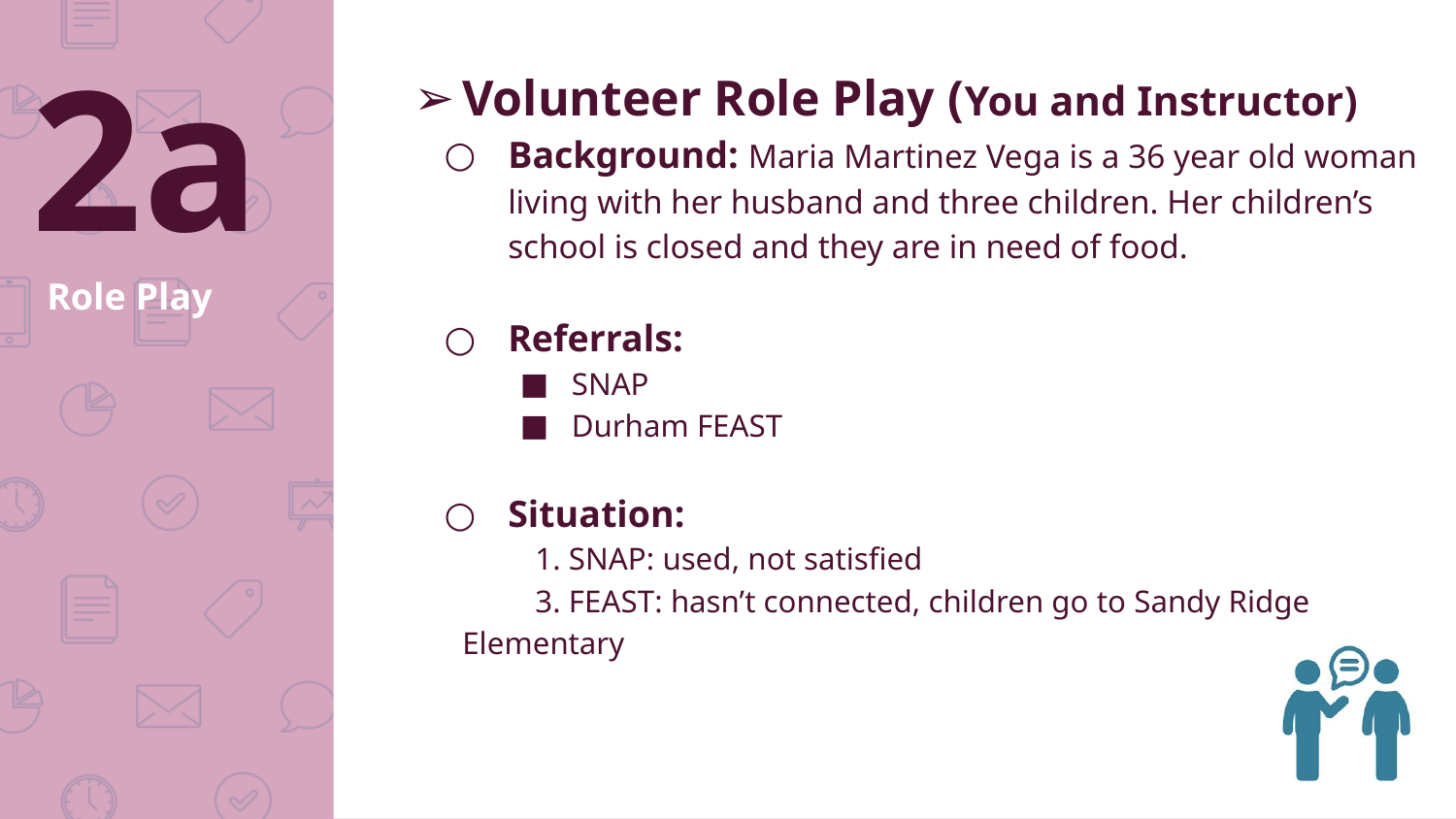

2a
Volunteer Role Play (You and Instructor)
Background: Maria Martinez Vega is a 36 year old woman living with her husband and three children. Her children’s school is closed and they are in need of food.
Referrals:
SNAP
Durham FEAST
Situation:
1. SNAP: used, not satisfied
3. FEAST: hasn’t connected, children go to Sandy Ridge Elementary
# Role Play

## Slide 102
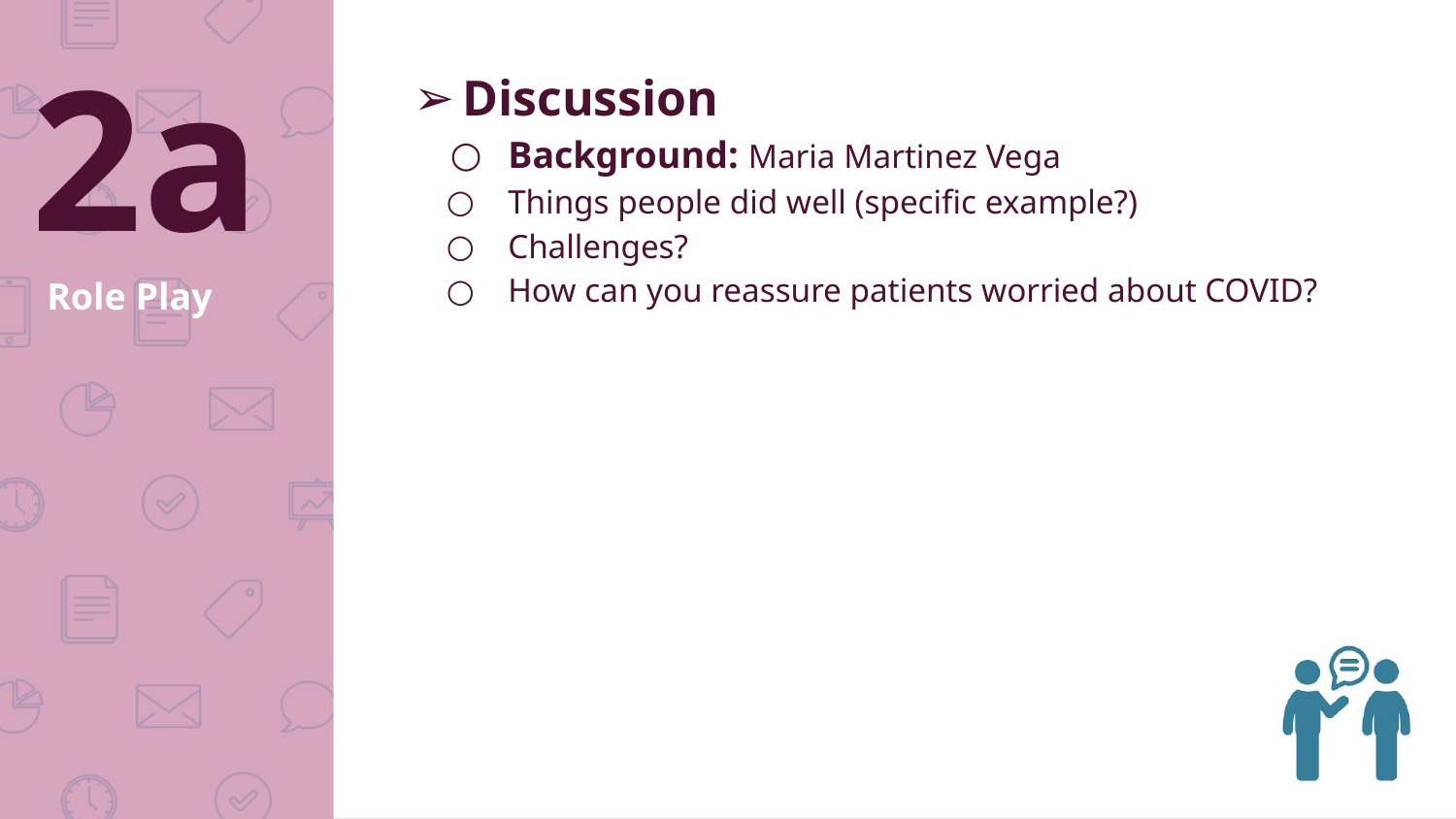

2a
Discussion
Background: Maria Martinez Vega
Things people did well (specific example?)
Challenges?
How can you reassure patients worried about COVID?
# Role Play

## Slide 103
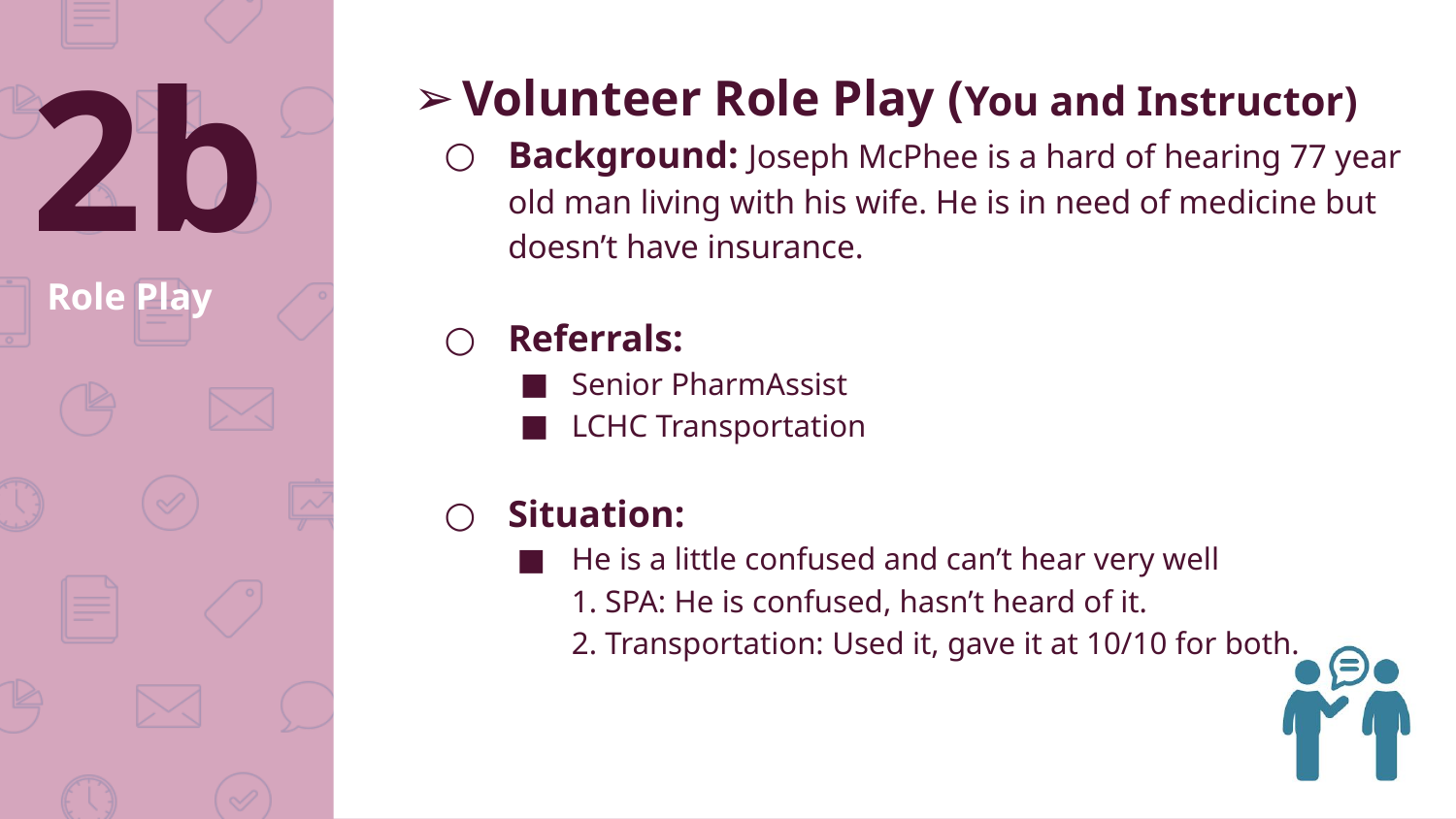

2b
Volunteer Role Play (You and Instructor)
Background: Joseph McPhee is a hard of hearing 77 year old man living with his wife. He is in need of medicine but doesn’t have insurance.
Referrals:
Senior PharmAssist
LCHC Transportation
Situation:
He is a little confused and can’t hear very well
1. SPA: He is confused, hasn’t heard of it.
2. Transportation: Used it, gave it at 10/10 for both.
# Role Play

## Slide 104
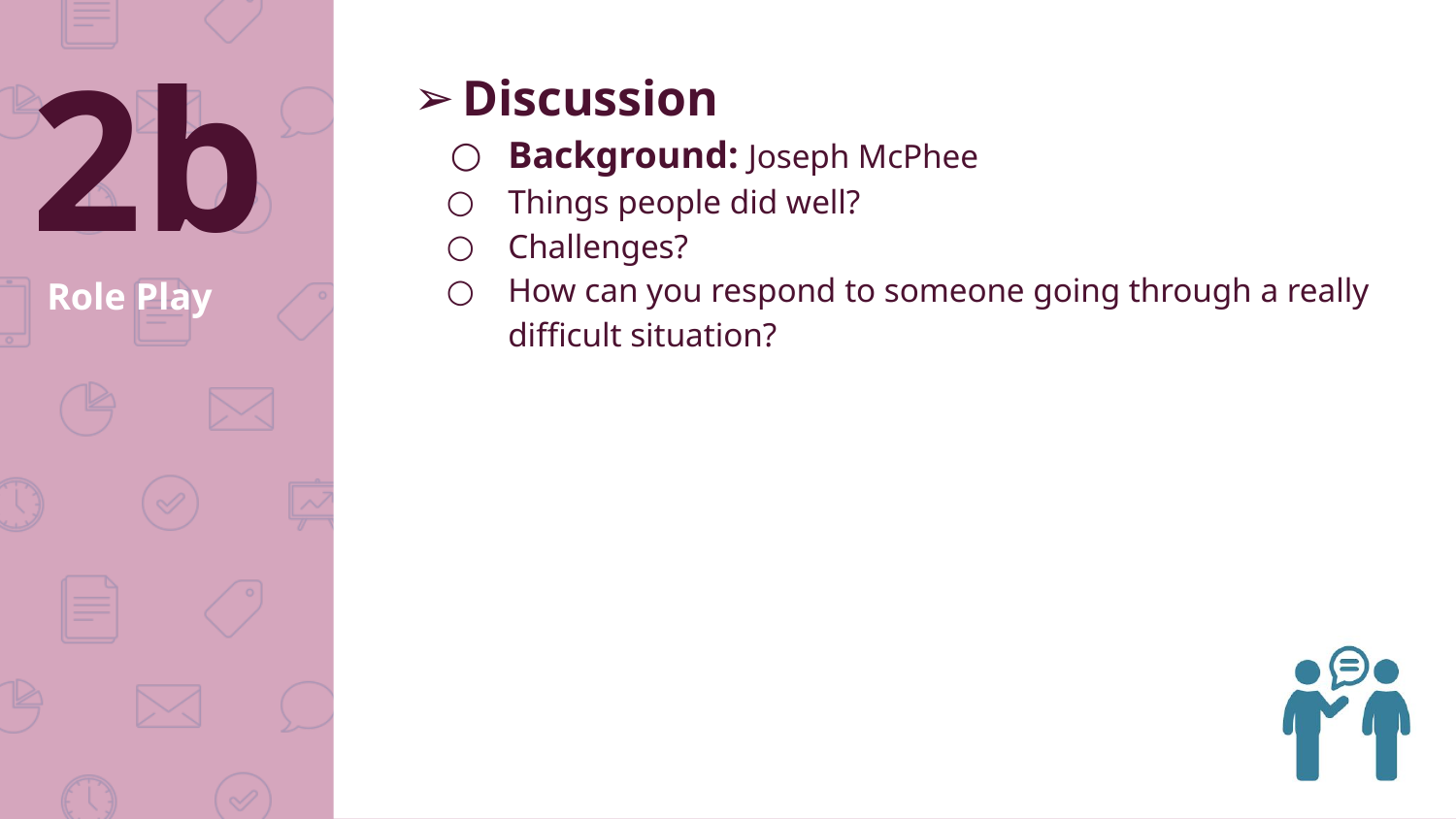

2b
Discussion
Background: Joseph McPhee
Things people did well?
Challenges?
How can you respond to someone going through a really difficult situation?
# Role Play

## Slide 105
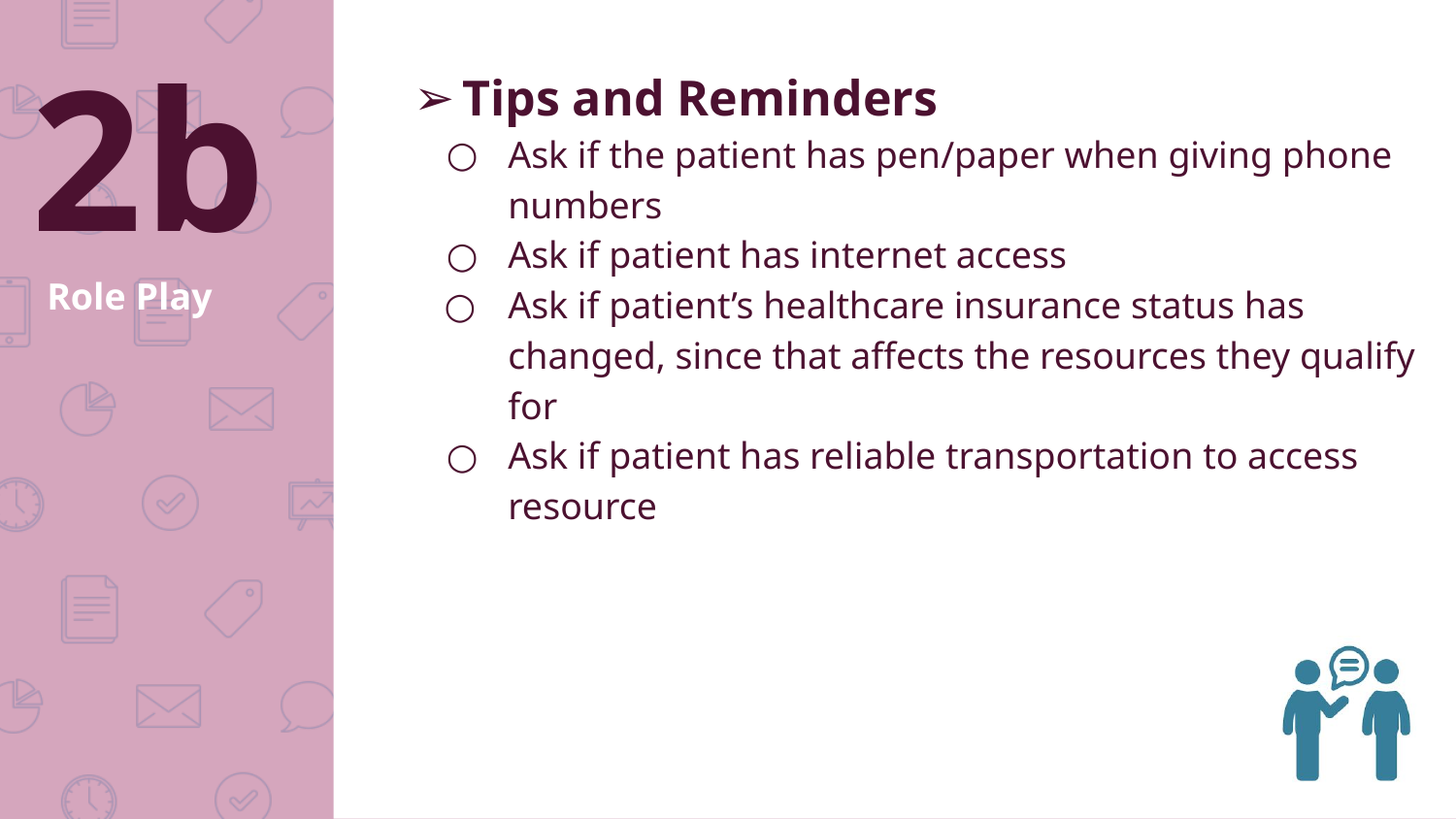

2b
Tips and Reminders
Ask if the patient has pen/paper when giving phone numbers
Ask if patient has internet access
Ask if patient’s healthcare insurance status has changed, since that affects the resources they qualify for
Ask if patient has reliable transportation to access resource
# Role Play

## Slide 106
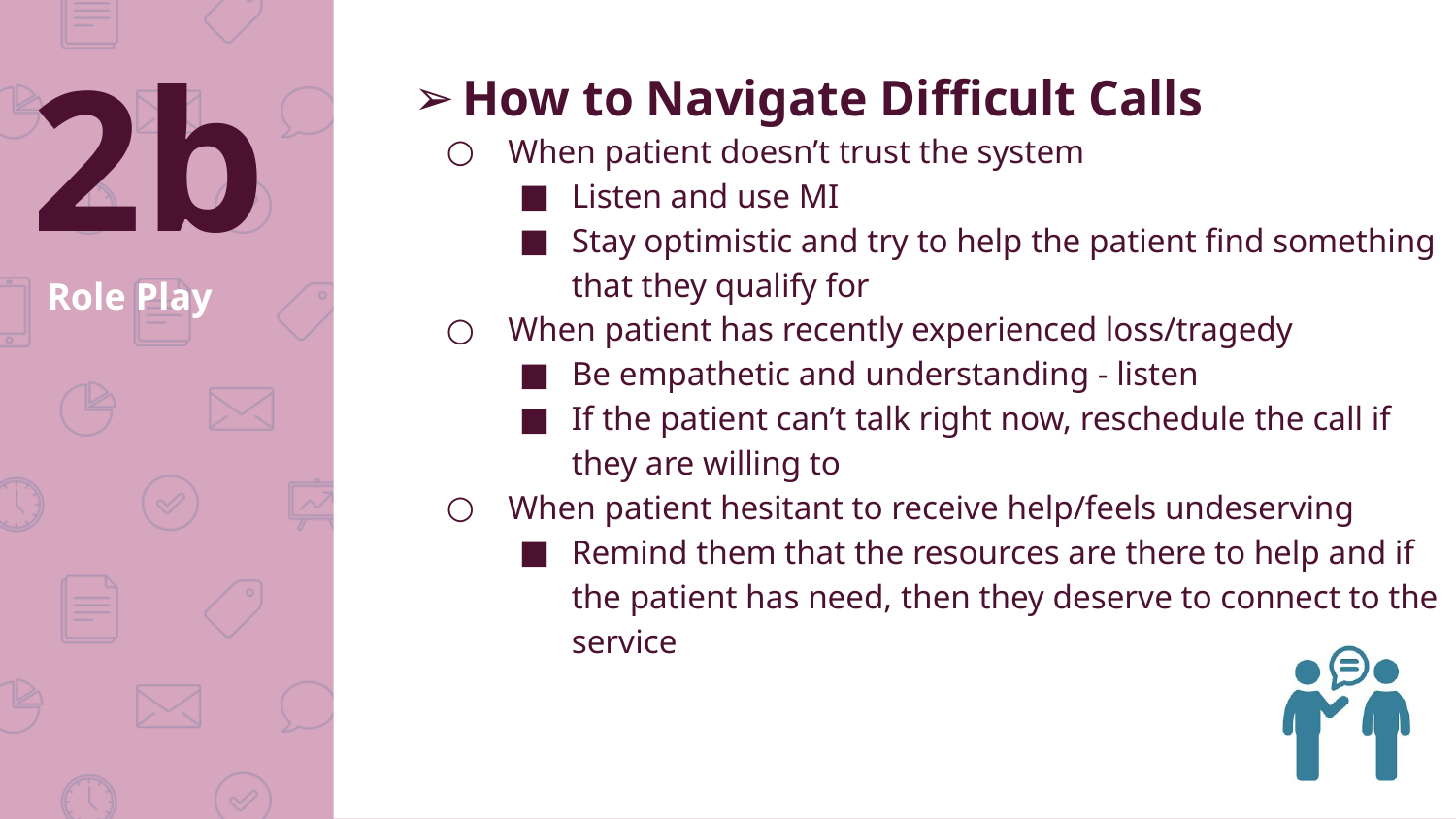

2b
How to Navigate Difficult Calls
When patient doesn’t trust the system
Listen and use MI
Stay optimistic and try to help the patient find something that they qualify for
When patient has recently experienced loss/tragedy
Be empathetic and understanding - listen
If the patient can’t talk right now, reschedule the call if they are willing to
When patient hesitant to receive help/feels undeserving
Remind them that the resources are there to help and if the patient has need, then they deserve to connect to the service
# Role Play

## Slide 107
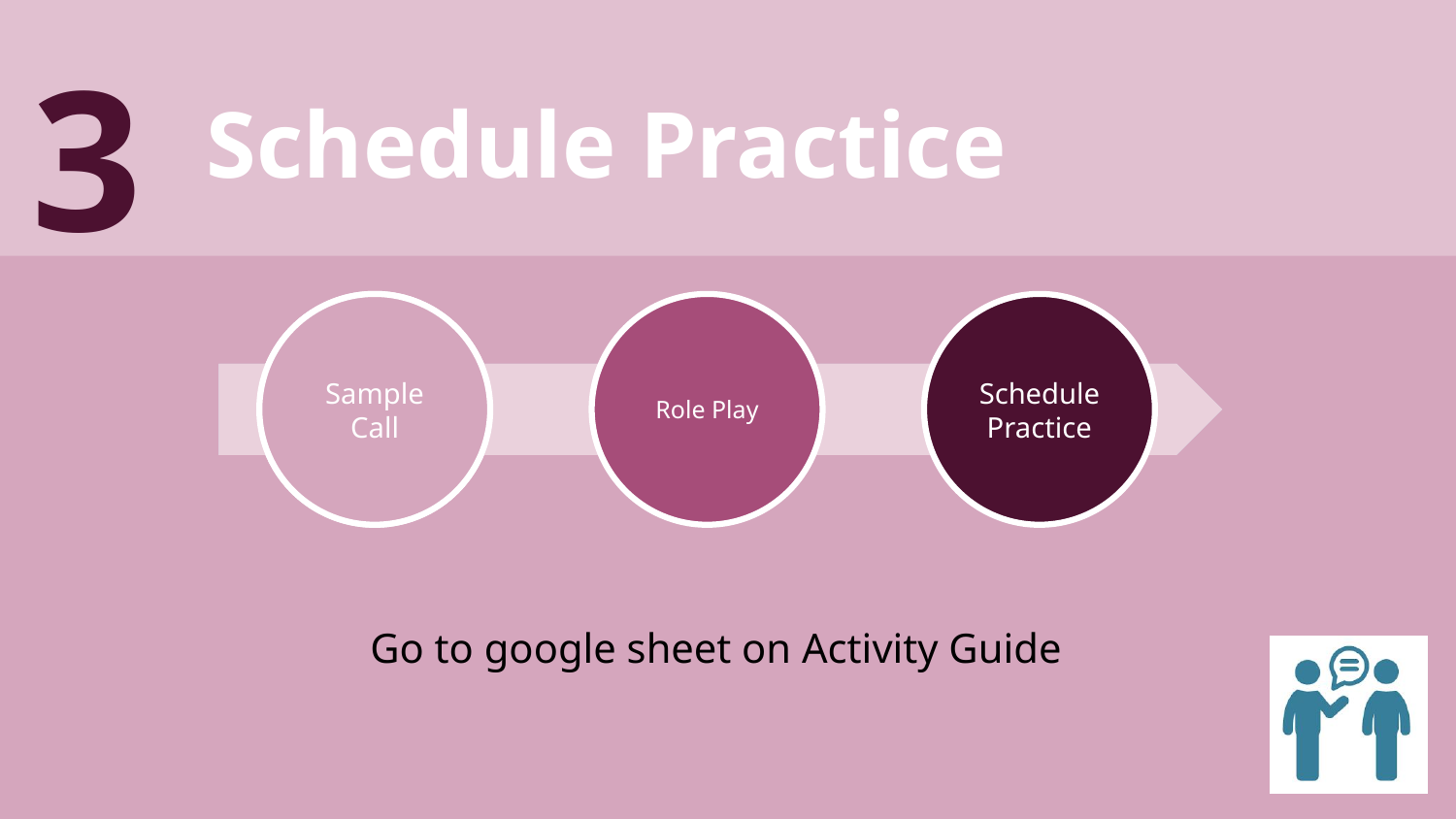

3
Schedule Practice
Shadow
Sample Call
Role Play
Schedule Practice
Go to google sheet on Activity Guide

## Slide 108
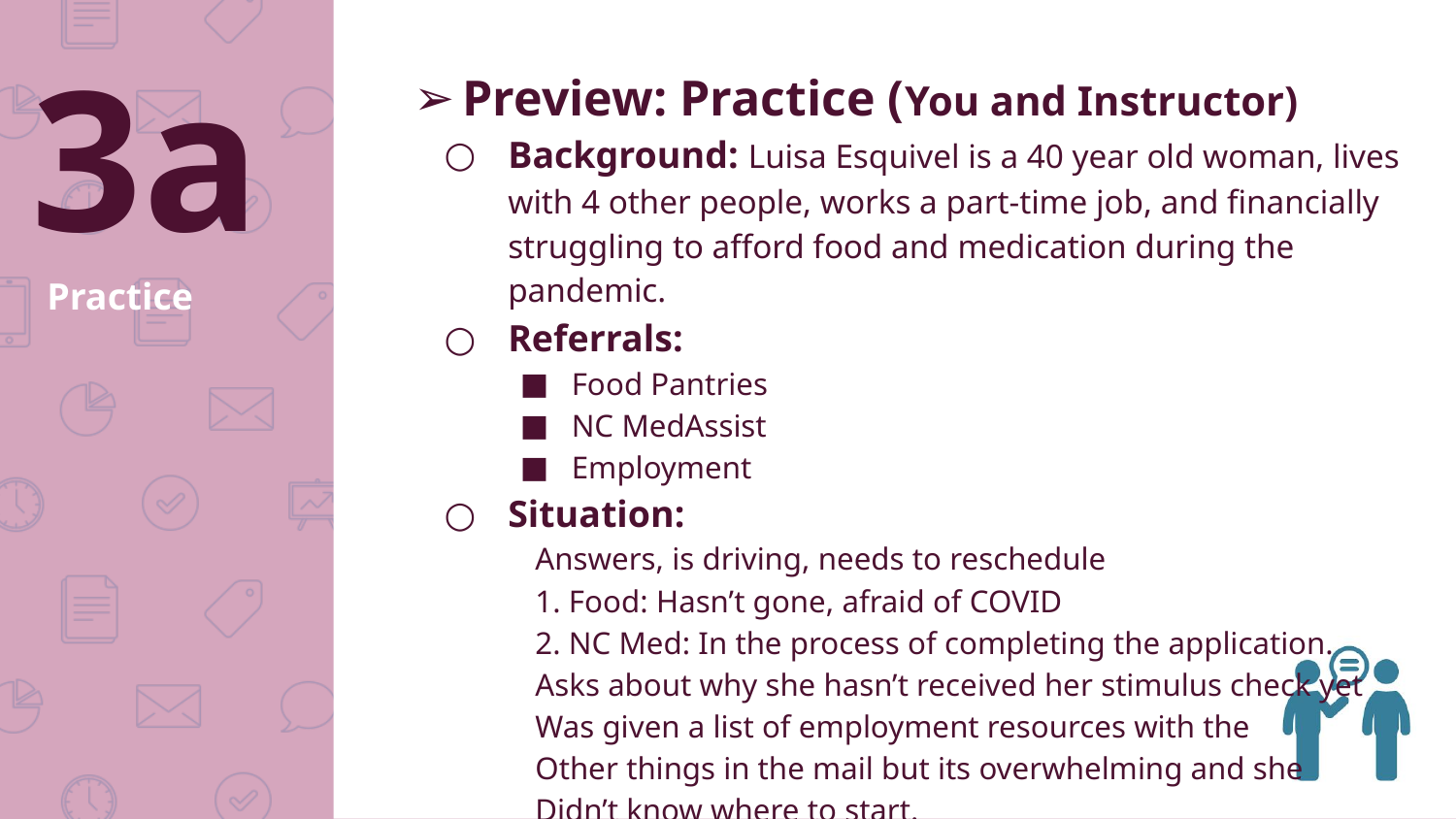

3a
Preview: Practice (You and Instructor)
Background: Luisa Esquivel is a 40 year old woman, lives with 4 other people, works a part-time job, and financially struggling to afford food and medication during the pandemic.
Referrals:
Food Pantries
NC MedAssist
Employment
Situation:
Answers, is driving, needs to reschedule
1. Food: Hasn’t gone, afraid of COVID
2. NC Med: In the process of completing the application.
Asks about why she hasn’t received her stimulus check yet
Was given a list of employment resources with the
Other things in the mail but its overwhelming and she
Didn’t know where to start.
# Practice

## Slide 109
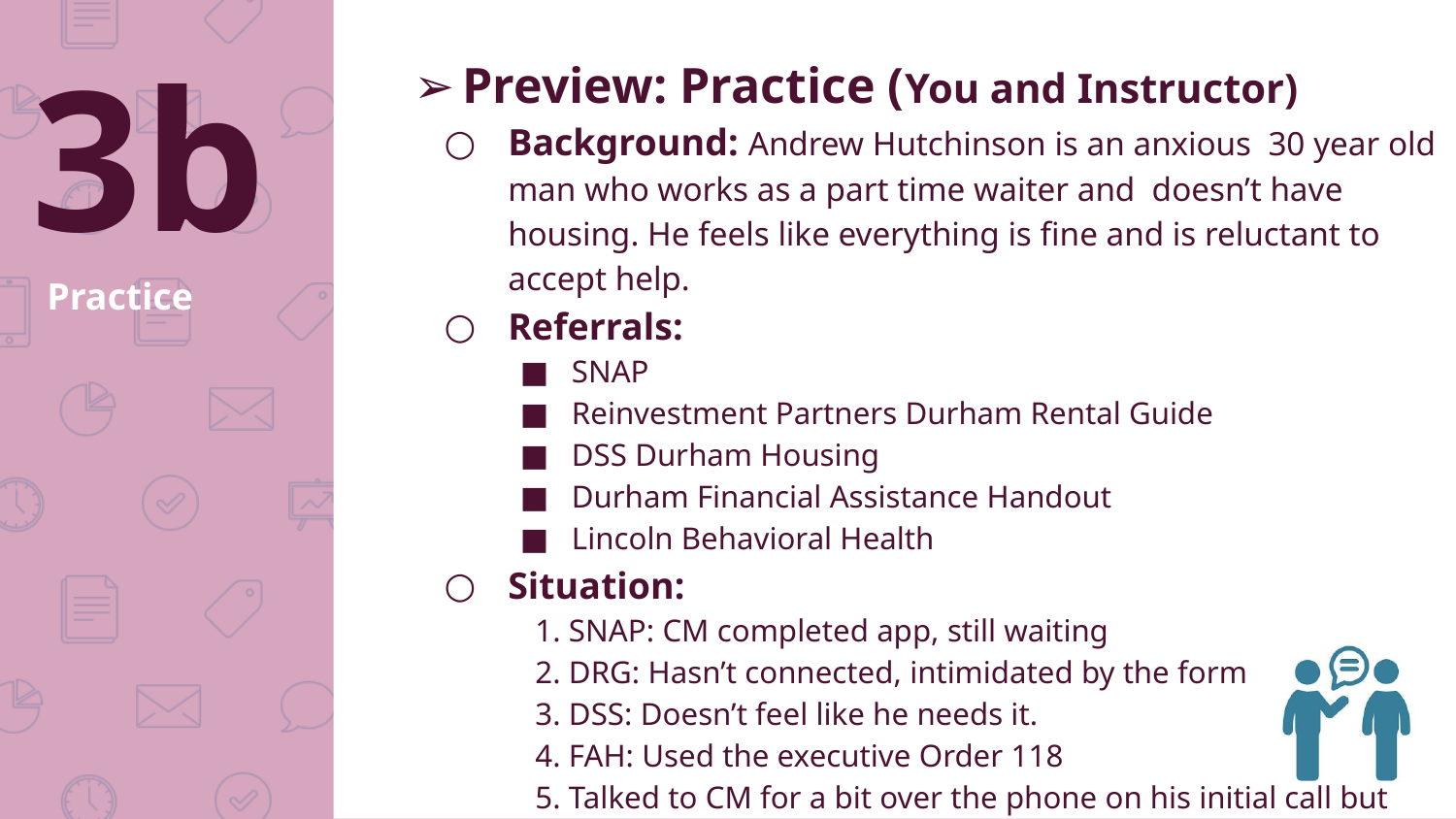

3b
Preview: Practice (You and Instructor)
Background: Andrew Hutchinson is an anxious 30 year old man who works as a part time waiter and doesn’t have housing. He feels like everything is fine and is reluctant to accept help.
Referrals:
SNAP
Reinvestment Partners Durham Rental Guide
DSS Durham Housing
Durham Financial Assistance Handout
Lincoln Behavioral Health
Situation:
1. SNAP: CM completed app, still waiting
2. DRG: Hasn’t connected, intimidated by the form
3. DSS: Doesn’t feel like he needs it.
4. FAH: Used the executive Order 118
5. Talked to CM for a bit over the phone on his initial call but doesn’t want more help
# Practice

## Slide 110
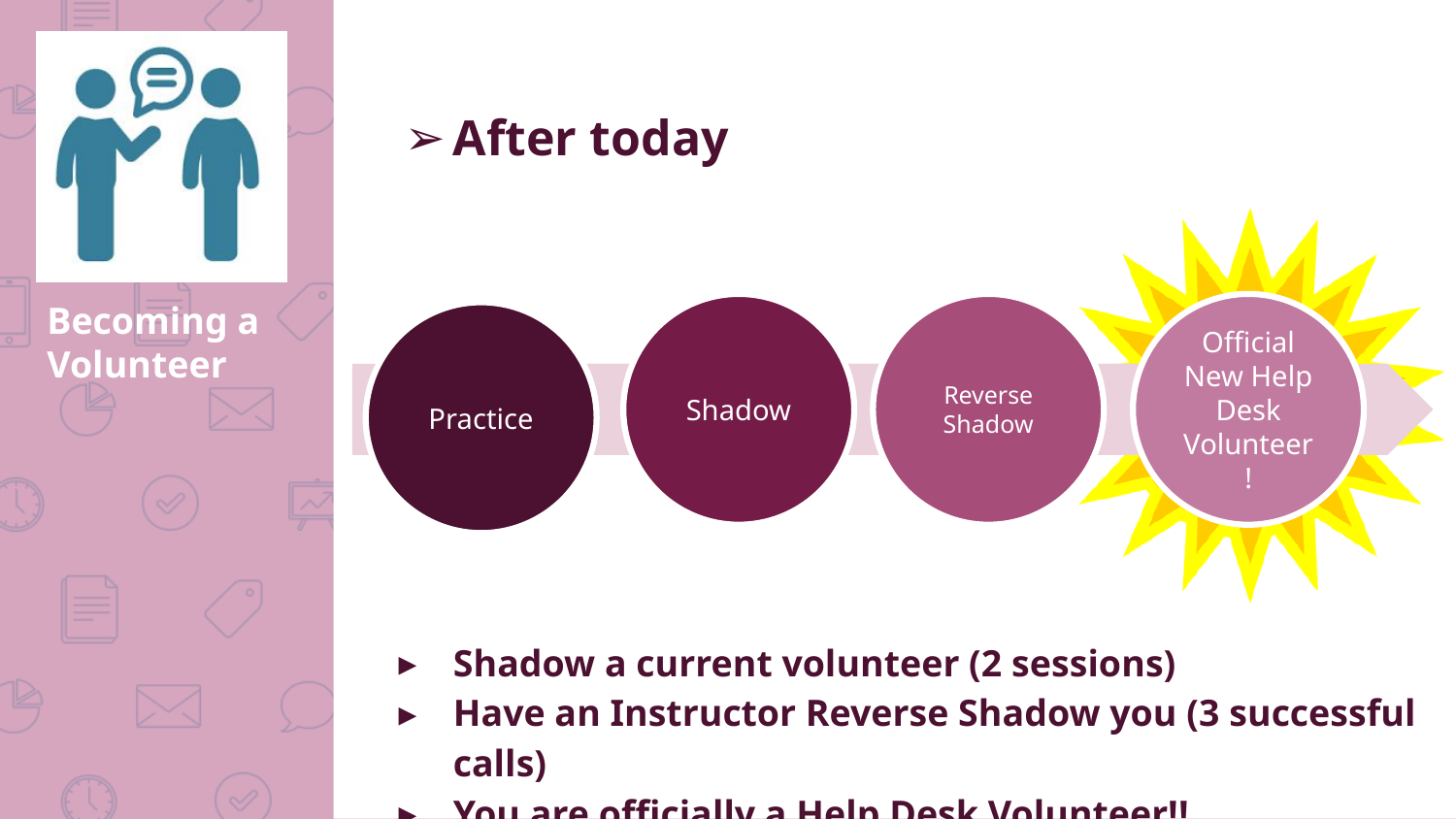

After today
Shadow a current volunteer (2 sessions)
Have an Instructor Reverse Shadow you (3 successful calls)
You are officially a Help Desk Volunteer!!
# Becoming a Volunteer
Shadow
Reverse Shadow
Official New Help Desk Volunteer!
Practice

## Slide 111
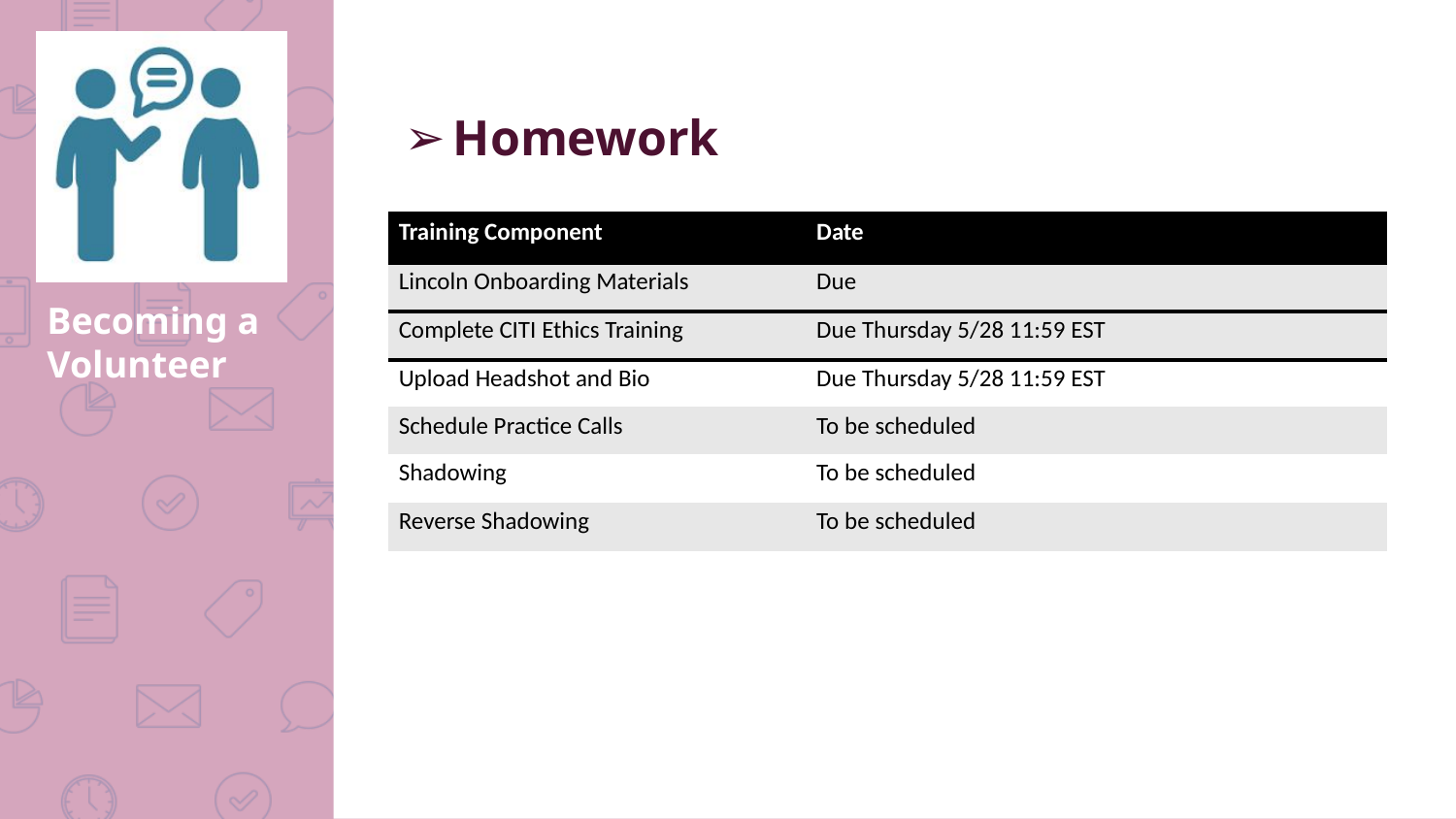

Homework
| Training Component | Date |
| --- | --- |
| Lincoln Onboarding Materials | Due |
| Complete CITI Ethics Training | Due Thursday 5/28 11:59 EST |
| Upload Headshot and Bio | Due Thursday 5/28 11:59 EST |
| Schedule Practice Calls | To be scheduled |
| Shadowing | To be scheduled |
| Reverse Shadowing | To be scheduled |
# Becoming a Volunteer
